# Identification of a urinary CD276 fragment for detecting resectable pancreatic cancer using a C-terminal proteomics strategy

**DOI:** 10.1038/s41598-024-65093-2

**Published:** 2024-06-20

**Authors:** Shuichi Mitsunaga, Nobuaki Okumura, Toshiki Takei, Toshifumi Takao, Hironobu Tsubouchi, Kohei Nakata, Masafumi Nakamura, Yuji Kitahata, Hideki Motobayashi, Masafumi Ikeda, Masamitsu Nakazato

**Affiliations:** 1 Division of Biomarker Discovery, Exploratory Oncology Research and Clinical Trial Center, National Cancer Center, Kashiwa, Japan; 2 Department of Hepatobiliary and Pancreatic Oncology, National Cancer Center Hospital East, Kashiwa, Japan; 3 Institute for Protein Research, Osaka University, Suita, Osaka Japan; 4 Division of Respirology, Rheumatology, Infectious Diseases, and Neurology, Department of Internal Medicine, Faculty of Medicine, University of Miyazaki, Kiyotake, Japan; 5 Department of Surgery and Oncology, Kyushu University, Fukuoka, Japan; 6 Second Department of Surgery, School of Medicine, Wakayama Medical University, Wakayama, Japan; 7 Department of Bioregulatory Science, Faculty of Medicine, University of Miyazaki, Kiyotake, Japan

**Keywords:** Paediatric cancer, Tumour biomarkers

## Abstract

This study aimed to confirm urinary protein fragments in relation to the presence of pancreatic ductal adenocarcinoma (PDAC) via a C-terminal proteomics strategy using exploratory and validation cohorts. Urinary fragments were examined by iTRAQ-labelling of tryptic peptides and concentrations of C-terminal fragments were evaluated. Only the urinary CD276 fragment showed a fold change (FC) of > 1.5 with a significant difference of P < 0.01 between healthy (H) and PDAC participants in both the exploratory (H, n = 42; PDAC, n = 39) and validation cohorts (H, n = 36; resectable PDAC, n = 28). The sensitivity and specificity of the CD276 fragment for diagnosing resectable PDAC were 75% and 89%, respectively, in the validation cohort. Postoperative urinary levels of the CD276 fragment were low as compared to those before surgery (n = 18, P < 0.01). Comprehensive C-terminus proteomics identified an increase in the urinary CD276 fragment level as a feature of patients with PDAC. The urinary CD276 fragment is a potential biomarker for detecting resectable PDAC.

## Introduction

Worldwide, pancreatic cancer is a leading cause of cancer-related death. In Japan, pancreatic cancer mortality and morbidity were reported as 37,677 patients in 2020 and 43,865 patients in 2019, respectively, and were associated with a poor prognosis, with a low 5-year survival rate of only 8.5% ^1^. The small minority of pancreatic cancer patients initially presenting with resectable disease (15–20%) ^2,3^ is one of the reasons for the poor survival rate, since surgical resection is the only curative treatment for pancreatic cancer. Serum carbohydrate antigen 19-9 (CA19-9) is a validated tumor marker in the management of pancreatic cancer, including for the detection of resectable disease. However, the sensitivity of CA19-9 for diagnosing resectable pancreatic cancer is unsatisfactory, at approximately 50–72% ^4,5^, indicating the urgent need for the development of specific and noninvasive biomarkers.

Disease-associated protein alterations, including protein synthesis, maturation and degradation, are reflected at the proteome level and might be detectable in relevant body fluids. Urine is an easily accessible bodily fluid that is used for clinical testing. A previous proteomics analysis reported that S100A9 protein was detected in 44% of urine samples, but not in plasma, of pancreatic cancer patients ^6^, which indicates that urine proteomics might provide tumor-specific biomarkers for pancreatic cancer. Urine contains C-terminally truncated proteins generated by proteolytic cleavage, such as by shedding of membrane proteins and degradation of protein C-termini. These protein fragments might reflect physiological proteolytic events, as well as disorders induced under disease conditions. Proteomic approaches intended for quantitation of protein C-termini are, thus, promising strategies for investing the changes in C-terminal protein processing ^7^. In the present study, we developed a new C-terminal proteomics workflow that quantitatively analyzes C-terminal peptides using iTRAQ technology. This workflow involves selective labelling of non-C-terminal peptides with ^18^O on trypsin digestion, and concentration of C-terminal peptides by SCX chromatography.

This study aimed to confirm urinary protein fragments in relation to the presence of pancreatic ductal adenocarcinoma (PDAC) through the C-terminal proteomics strategy, using both exploratory and validation cohorts. Further, the discriminative ability of PDAC-related urinary fragments was tested in a comparison of healthy and PDAC participants, as well as by an observed postoperative decrease in their levels in the validation cohort.

## Results

### Patient population

In total, 145 participants were analyzed in this study, including 42 healthy volunteers (mean age, 54 years; male, n = 21 [50.0%]) and 39 PDAC patients (mean age, 65 years; male, n = 27 [69.2%]) in the exploratory cohort, and 36 healthy volunteers (mean age, 57 years; male, n = 11 [30.6%]) and 28 patients with resectable PDAC (mean age, 70 years; male, n = 14 [50.0%]) in the validation cohort (Supplementary Table S1). All the PDACs were treatment naïve and were diagnosed pathologically.

### Measurement of C-terminal fragments

**Figure 1:**
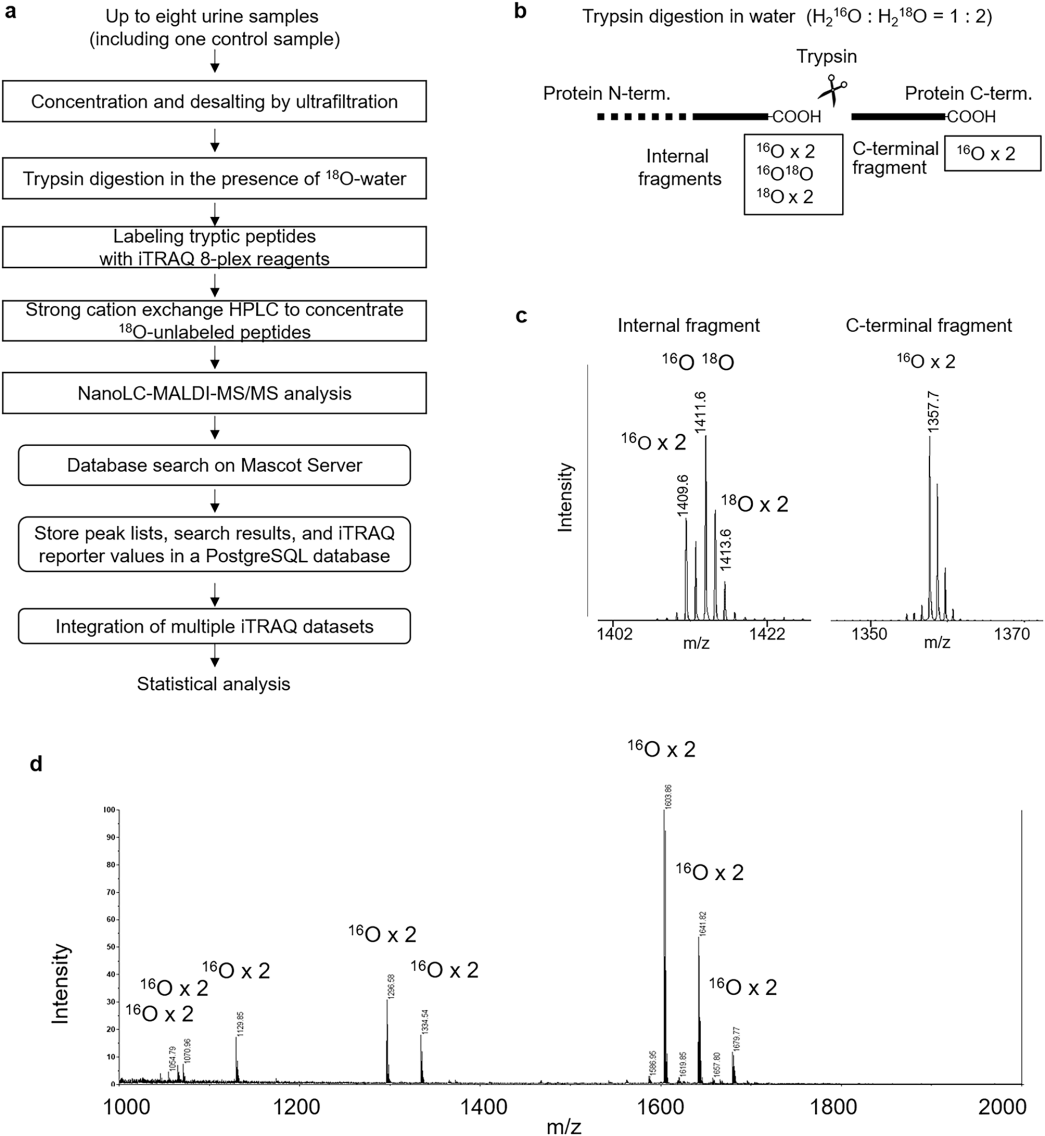
Measurement of C-terminal tryptic fragments. (**a**) Proteomic workflow for quantitative analysis of protein C-termini. (**b**) Trypsin digestion in the presence of ^18^O-water. Trypsin digestion results in ^18^O incorporation into internal fragments, but not into C-terminal fragments. (**c**) Distinction of C-terminal fragments from internal fragments by MALDI-TOF MS. Internal fragments can be recognized by their unusual isotopic distribution due to ^18^O. (**d**) Enrichment of C-terminal fragments by SCX chromatography. In the purified fraction, peptides from protein C-termini were predominant, as judged by isotope distribution. *MALDI-TOF MS* matrix-assisted laser desorption/ionization time-of-flight mass spectrometry, *SCX* strong cation exchange.

Urinary protein values were obtained from 145 evaluable participants and their C-termini were analyzed using a quantitative proteomics approach (Fig. 1a). In this workflow, the proteins were digested with trypsin in water (H_2_^16^O: H_2_^18^O = 1: 2), in which internal fragments were labeled with ^18^O, whereas C-terminal fragments remained unlabeled (Fig. 1b). This allowed us to distinguish C-terminal fragments from internal ones by MALDI-TOF MS (Fig. 1c). The peptides were then labeled with iTRAQ 8-plex reagents and applied on an SCX column. Since most C-terminal tryptic fragments have no lysine and arginine residue, whereas internal ones always have at least one such residue, C-terminal fragments tend to elute earlier from a Polysulfoethyl A column than internal ones ^8^ (Fig. 1d). A few internal peptides were present in the purified fraction, but were recognized by their isotope distribution and excluded from further analysis. Purified C-terminal peptides were analyzed by nanoLC-MALDI MS/MS analysis. Reporter ion intensities were obtained from the MS2 spectra, stored in a Postgresql database, and subjected to statistical analysis. Each iTRAQ signal was normalized with the ratio to the standard urine sample.

### PDAC-related urinary protein fragment

**Figure 2:**
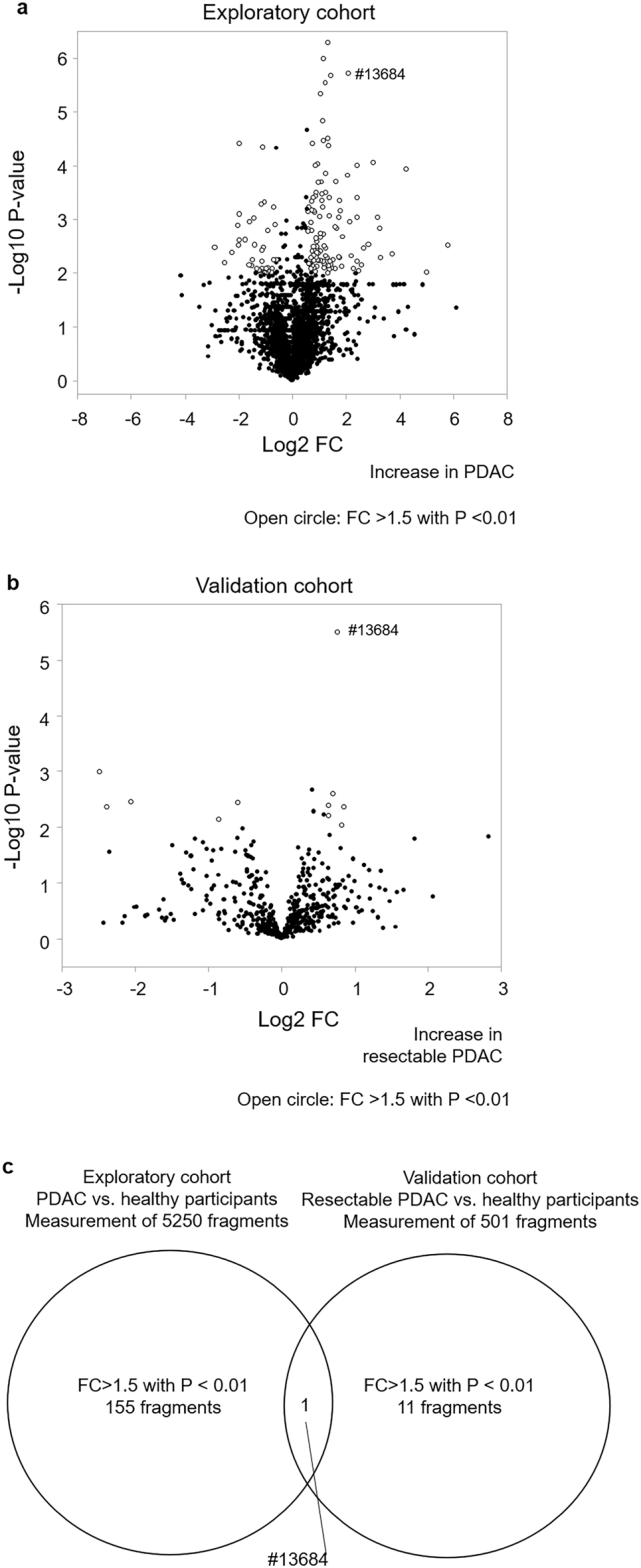
Urinary protein fragment profile in relation to the presence of PDAC. (**a**) Volcano plots of differential urinary protein fragment levels between PDAC and healthy participants, and (**b**) between resectable PDAC and healthy participants. (**c**) Venn diagram showing candidate PDAC-related urinary fragments in the exploratory cohort and in the validation cohort. Each point in the volcano plot represents a urinary protein fragment, the abscissa represents log2 FC, and the ordinate represents the significance level of differentially expressed urinary protein fragments (− log10 P value by the two-tailed Student’s t test). *PDAC* pancreatic ductal adenocarcinoma, *FC* fold change with respect to the mean value in healthy subjects.

A total of 5250 urinary protein fragments were evaluated in the exploratory cohort (Fig. 2a). Among them, 153 fragments showed an FC of > 1.5 with P < 0.01 (Supplementary Table S2). In the validation cohort, an FC of > 1.5 with P < 0.01 was found in 11 among 501 measurable fragments (Fig. 2b, Supplementary Table S3). Only the peptide number 13684 (#13,684) showed an FC of > 1.5 with P < 0.01 in both the exploratory and validation cohorts (Fig. 2c). In male and female population, #13,684 levels in PDAC patients were high as compared to healthy participants in exploratory cohort (P < 0.001 in both) and in validation cohort (P = 0.002 in male and P < 0.001 in female). These results prompted us to evaluate #13,684 as a PDAC-related urinary fragment.

**Figure 3:**
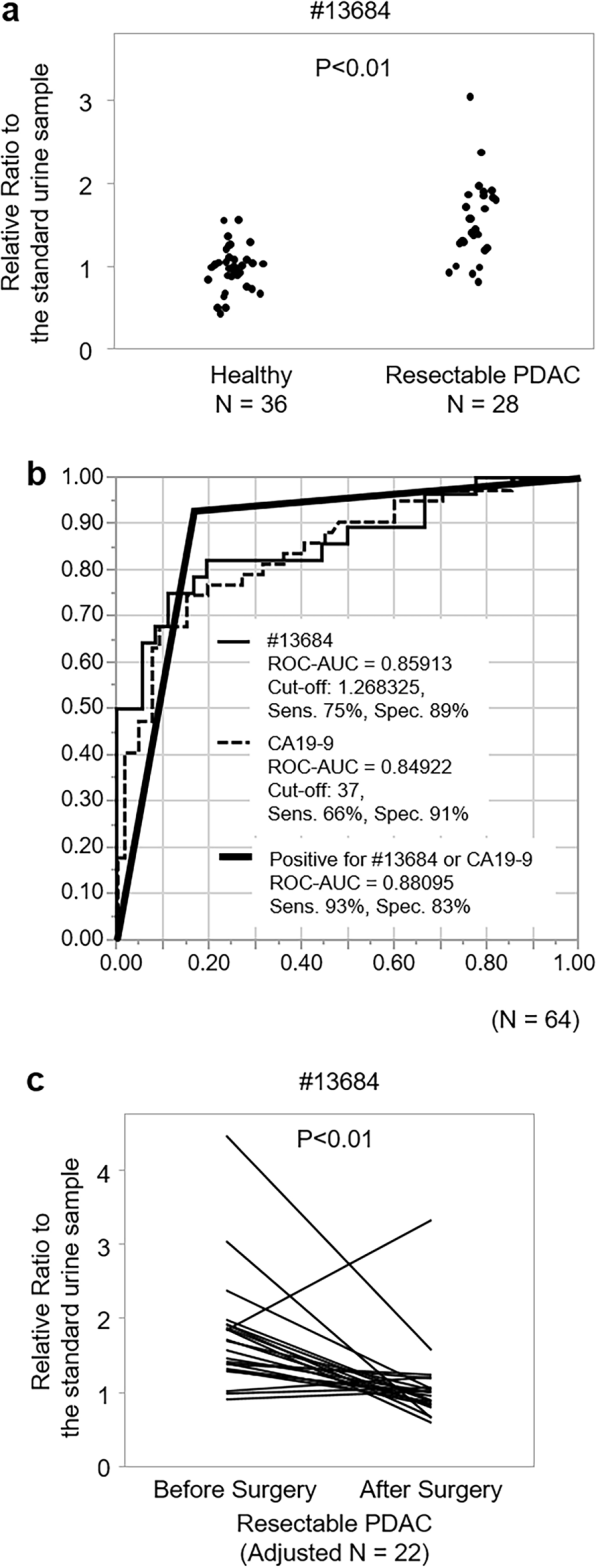
PDAC-related urinary fragment. (**a**) Distribution of urinary #13,684 levels in resectable PDAC and healthy participants in the validation cohort. (**b**) ROC curves of serum CA19-9 and urinary #13,684 levels for discriminating resectable PDAC patients from healthy participants in the validation cohort. (**c**) Postoperative changes in urinary #13,684 levels in 22 patients in the validation cohort. *PDAC* pancreatic ductal adenocarcinoma, *ROC curve* receiver operating characteristic curve, *CA19-9* Carbohydrate antigen 19-9, *AUC* area under the curve.

In the validation cohort, the mean value of #13,684 relative ratio to the standard urine sample was 0.96 (95% confidential interval [CI] 0.88, 1.05) in healthy volunteers and 1.64 (95% CI 1.35, 1.92) in patients with resectable PDAC (Fig. 3a), indicating a significant difference in #13,684 levels between healthy and resectable PDAC participants (P < 0.01). ROC-AUCs for discriminating resectable PDAC from healthy participants were 0.86 for #13,684 and 0.85 for CA19-9 (Fig. 3b). A diagnostic cut-off value of #13,684 was 1.27 which was determined on the maximum Youden index (sensitivity − [1 − specificity]) for detecting resectable PDAC. The sensitivity and specificity of a #13,684 value of ≥ 1.27 for diagnosing resectable PDAC were 75% and 89%, respectively. The sensitivity and specificity of a CA19-9 value of > 37 U/mL were 66% and 91%, respectively.

In the validation cohort, postoperative urinary collection was performed in 22 patients. The mean interval from the date of pancreatectomy to the date of postoperative urinary collection was 37.5 days (95% CI 31.8, 43.3). A postoperative decrease in urinary #13,684 was observed in 18 patients (81.8%) (Fig. 3c). Overall, urinary levels of #13,684 were lower after surgery (mean: 1.05 [95% CI 0.81, 1.29]) as compared to before surgery (mean: 1.74 [95% CI 1.40, 2.08]; P < 0.01).

### Identification of the #13,684 peptide as CD276 (242–258)

**Figure 4:**
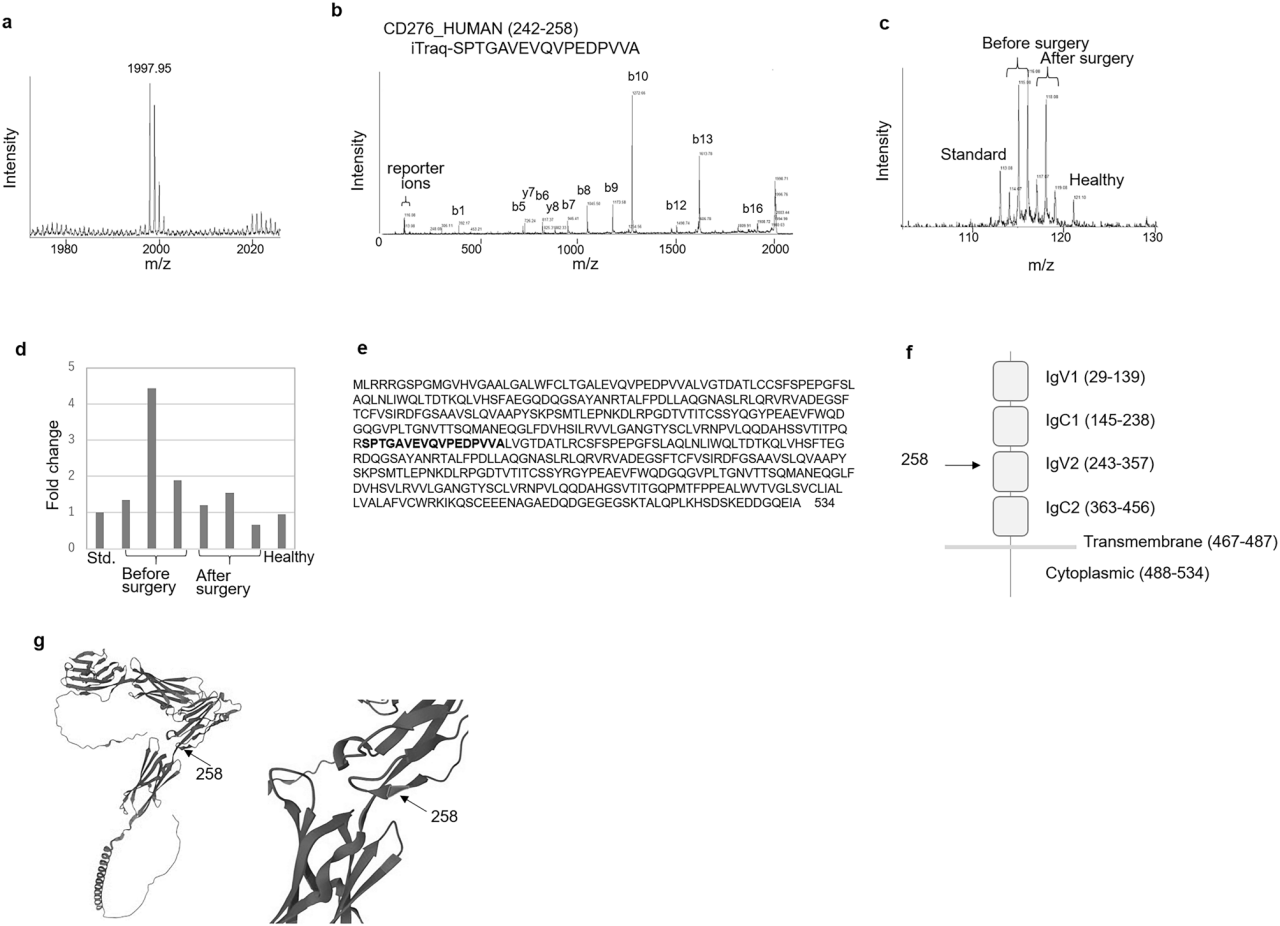
Detection of CD276_HUMAN (242–258) as a candidate biomarker for pancreatic cancer. (**a**) MS1 spectrum of tryptic peptide #13,684. The isotope distribution confirmed that it was derived from the C-terminal end of a protein. (**b**) MS2 spectrum of peptide #13,684. From this MS2 spectrum, the peptide was identified as the CD276 (242–258) fragment by a database search on a MASCOT server. The labeled MS2 peaks were those that matched the calculated values for the b- and y-series ions of CD276 (242–258). (**c**) Example of the reporter region of an MS2 spectrum for peptide #13,684. (**d**) Reporter ion intensities of CD276_HUMAN (242–258) in a single iTRAQ data set after normalization. (**e**) The full-length amino acid sequence of CD276 (4Ig form) obtained from the Uniprot database (accession number Q5ZPR3). The sequence highlighted is the #13,684 fragment. (**f**) Domain structure of CD276 (4Ig form). The arrow indicates the C-terminal end of #1368. (**g**) Predicted structure of CD 276 (4Ig form). Structural data for the protein (Uniprot number Q5ZPR3) were retrieved from the AlphaFold protein structure database and visualized using PyMOL 1.3 (Schrodinger). *MS1* mass spectrometry, *MS2* tandem mass spectrometry.

The mass spectra obtained above were further analyzed using the MASCOT database search program for peptide identification. Peptide #13,684 was observed as a peak at 1997.5 *m/z* [M + H]^+^ on MS1 spectra (Fig. 4a). Its isotope distribution confirmed that it was derived from the C-terminus of a protein (Fig. 4a). A database search using an MS2 peak list resulted in a significant match with fragment 242–258 of human CD276, with a peptide score of 92 (Fig. 4b, Supplementary Fig. S2). These results suggest that peptide #13,684 is the C-terminal region of a missing CD276 fragment after residue 269. The MS2 spectra also showed the relative amount of the peptide in eight different samples, including a standard sample (Fig. 4c). Each reporter ion was normalized (Fig. 4d) and used for statistical analysis, as described above. The cleavage site was located just below the second Ig domain of the CD276 extracellular region (Fig. 4e–g).

## Discussion

CD276 is a member of the B7 family ^9^, and is thought to serve as an accessory co-regulator of T-cell responses after initial antigen priming. In a previous study, CD276 was expressed in the cell membrane and cytoplasm of cancer cells in 93.2% of 55 pancreatic cancer tissue samples ^10^. In another study, CD276 expression by cancer tissue was higher than that by non-cancer tissue in each of 11 pancreatic cancer patients ^10^. In the present study, the urinary CD276 fragment level was increased in PDAC patients in the exploratory cohort and in resectable PDAC patients in the validation cohort, as compared to those in healthy participants. Surgical resection of the PDAC induced a reduction in urinary CD276 fragment levels. In this comprehensive C-terminus proteomics study, the urinary CD276 fragment was identified as the PDAC-related urinary protein fragment. Additionally, the discriminative ability of the urinary CD276 fragment between resectable PDAC and healthy participants was the same as that of CA19-9, which is a clinically validated biomarker for detecting PDAC (NCCH guideline). This suggests that the urinary CD276 fragment might also be a potential biomarker for detecting PDAC.

The ectodomain of CD276 has been identified as a single IgV- and IgC-like domain (2Ig form) or a duplicate of a IgV- and IgC-like domain (4Ig form), although the physiological differences between the 2Ig and 4Ig forms have not yet been elucidated ^11,12^. In this study, amino-acid sequence analysis revealed that the PDAC-related CD273 fragment was composed of IgV2 in 4IG form of CD276.

A soluble form of CD276 is produced by monocytes, activated T cells, dendritic cells, and B7-H3-positive cancer cells, including human pancreatic cancer cells ^13,14^. Soluble CD276 promotes the invasion of pancreatic cancer cells via the NF-kappaB pathway ^14^. Matrix metalloproteinase inhibitors caused an increase of cell-surface CD276 expression in cancer cells and a decrease in soluble CD276 levels in the supernatant ^13^, indicating that soluble CD276 is produced by the cleavage of cell-surface CD276 via the effect of matrix metalloproteinase. Elastase preferentially cleaves at the C-terminal amino-acid of alanine ^15^. In this study, the C-terminus amino-acid of the PDAC-related CD276 fragment was alanine (Fig. 4e), which meant that the cleavage due to elastase contributed the production of the PDAC-related CD276 fragment. The difference between matrix metalloproteinase-cleaved and PDAC-related soluble CD274 is unknown. The molecular weight of soluble CD274 cleaved by matrix metalloproteinase was previously reported as 16.5 kDa ^13^. Investigation of the molecular weight of the PDAC-related CD276 fragment might be the first step to realizing the role of the PDAC-related CD276 fragment as a biomarker of resectable PDAC.

The variety of urinary protein fragments was different between exploratory and validation cohorts in this study. The 5250 fragments were measured in exploratory cohort, and the 501 fragments in validation cohort. This discrepancy might be originated from the differences of tumor or sample status between two cohorts. The patients of exploratory cohort had stage I or II (17.9%), stage III (23.1%) and stage IV (59.0%). In validation cohort, the frequencies of stage I or II and stage III were 96.4% and 3.6%, respectively (Supplementary Table S1). Advanced stage-related protein fragmentation might affect the differences of urinary protein fragments between two cohorts. We were aware of the trade-off between storage time and urinary protein degradation in exploratory cohort analysis. The urinary sample with long storage time (more than 750 days), therefore, were excluded from validation cohort analysis. Median sample storage time in exploratory cohort (935 days, interquartile range: 894–971) was longer than that in validation cohort 574 days (507–705). Taken together, short storage time and high frequency of early stage PDAC in validation cohort might contribute a decrease of protein fragmentation, which could reduce the variety of urinary protein fragments as compared with that in exploratory cohort. The consistency of PDAC-related proteolytic cleavage across two cohorts was concerned in this study. In the respected to PDAC-related fragments, we plotted log2 FC for PDAC vs. healthy participants using data of exploratory and validation cohorts (Supplementary Fig. S2). The elevations of PDAC-related urinary fragments in PDAC patients were reproducible in two cohorts.

In this study, we intended to perform a quantitative C-terminal proteomic analysis using the iTRAQ technology, which requires free amino groups of peptides ^16^. However, many C-terminal proteomic strategies are unapplicable to iTRAQ because amino groups are derivatized to achieve good separation of C-terminal peptides on SCX chromatography ^7^. In our approach, internal peptides were labeled with ^18^O by trypsin treatment in the presence of ^18^O-water, allowing C-terminal fragments to be distinguished by isotope distribution on MALDI-TOF MS. The combination of ^18^O-labelling, iTRAQ derivatization and SCX chromatography provided a simple and robust solution for selective iTRAQ analysis of C-terminal fragments.

A limitation of this study is its small sample size, which could be a source of bias. Multicenter studies with larger sample sizes are needed to further validate the utility of urinary CD276 as a biomarker of PDAC.

In conclusion, comprehensive C-terminus proteomics identified an increase in urinary levels of the CD276 fragment as a clinical characteristic in patients with PDAC. This suggests that the urinary CD276 fragment might be a potential biomarker for detecting resectable PDAC.

## Methods

### Study design and participants

This prospective observational study was approved by the ethics review committee of National Cancer Center Hospital East and National Cancer Center Hospital (approval no. K2011-001) and the institutional review boards of other institutions, and complied with the Declaration of Helsinki and the Japanese Ethical Guidelines for Medical and Health Research Involving Human Subjects. This study consisted of two parts to meet the two study aims: establishing and confirming the PDAC-related urinary protein fragments in the exploratory and validation cohorts, and investigating the diagnostic ability and postoperative changes of PDAC-related urinary protein fragments in the validation cohort. Between 2013 and 2016, patients with PDAC and healthy volunteers at the National Cancer Center Hospital East Kashiwa and Miyazaki University were enrolled and allocated to the exploratory cohort. Patients with resectable PDAC and healthy volunteers at the National Cancer Center Hospital East Kashiwa, Kyusyu University, Wakayama Medical University and Miyazaki University between 2018 and 2019 were enrolled and allocated to the validation cohort. Written informed consent was obtained from patients before study enrollment. The study protocols were approved by the institutional review boards of all institutions before the study commenced. This study complied with the Declaration of Helsinki and the Japanese Ethical Guidelines for Medical and Health Research Involving Human Subjects.

Eligible patients included those with a clinical diagnosis of treatment-naïve PDAC or healthy participants. The key exclusion criteria were a urine dipstick test result for glucose, protein, or blood of ≥ 2+. When a pancreatic cancer patient was not pathologically diagnosed with PDAC after enrollment, the patient was excluded from analysis. In the validation cohort, patients in whom the urine sample was stored for a long time (more than 750 days), or with insufficient semi-quantification of the urinary fragment were excluded, since the validation cohort was established for confirming the value of the urinary protein fragment. Clinical data, including serum CA19-9 levels within 14 days after enrollment, were recorded. Tumors were staged according to the TNM classification ^1^ before the initiation of anti-cancer treatment.

### Sample collection and preparation

First morning urine samples were collected from pancreatic cancer patients before the starting date of the initial anti-cancer treatment and from healthy volunteers. The samples were immediately frozen and stored at − 80 °C. Using 20–45 mL urine samples, urinary proteins were concentrated around 100-fold by ultrafiltration using an Amicon ultra-15 (10 K cut off) centrifugal filter unit (Merck Millipore, Massachusetts, USA), followed by a buffer exchange to 100 mM triethylammonium bicarbonate buffer (pH 8.5, TEAB) on the same filter. The protein concentration in the centrifuged sample was determined using a microBCA Protein Assay Kit (ThermoFisher Scientific, Massachusetts, USA), using bovine serum albumin as the standard.

### iTRAQ-labelling of tryptic peptides and concentration of C-terminal fragments

Concentrated urinary proteins (400 μg protein) were incubated in 100 mM TEAB containing 90 mM dithiothreitol and 0.1% SDS for 60 min at 60 °C. Then, 1 M acrylamide was added to make a final concentration of 180 mM, and was incubated for 50 min at 37 °C. After the incubation, 0.5 M cysteine solution (3.2 μL, 1.6 μmol) was added to the mixture, and it was again incubated for 50 min at 37 °C. The proteins were digested in a reaction mixture containing 140 pmol trypsin (Roche, Basel, Switzerland), 100 mM TEAB, 61% H_2_^18^O and 0.01% SDS, and incubated for 6 h at 37 °C. This resulted in ^18^O incorporation into internal but not C-terminal fragments ^17^. At the end of the incubation, the reaction was terminated by adding 2.5 μL acetic acid. The samples were then desalted on a C_18_ cartridge column (HF Bond Elut, 100 mg, 1 mL, Agilent), dried on a vacuum centrifuge, redissolved in 20 μL of 1 M TEAB, and labeled with one of the eight iTRAQ 8-plex reagents (Sciex, Massachusetts, USA) according to the manufacturer’s protocol ^16^. The iTRAQ-113 reagent was used to label the standard sample, which was prepared from a mixture of urinary samples. Then, up to seven samples and one standard sample, each of which was labeled with a different isobaric tag, were mixed, desalted using HF Bond Elut, and applied on a cation-exchange column (Polysulfoethyl A, 50 × 4.6 mm, 5 μm, PolyLC inc. Columbia, USA) to concentrate monovalent cationic peptides ^8^. The peptides were eluted with 0.5 M potassium chloride containing 20% acetonitrile and 5 mM phosphoric acid (pH 2.5), and fractions containing ^18^O-unlabelled peptides were collected. The peptides were desalted on HF Bond Elut, dried on a vacuum concentrator, redissolved in 25 μL of 10% acetonitrile/0.1% TFA, and 10 μL aliquots were applied on a C_18_ capillary column (IntegraFrit, 150 mm × 75 μm, 3 μm, New Objective, Massachusetts, USA). Peptides were eluted with an acetonitrile gradient in 0.1% TFA (3–47% in 90 min) at a flow rate of 200 nL/min, and 30 s fractions were spotted on a 196-well MALDI target plate using an automatic MALDI plate spotter. Mass spectrometry and data processing have been described in the Supplementary Methods.

### Statistical analysis

To explore PDAC-related fragments, urinary fragment levels were compared between PDAC patients and healthy participants using missing value imputation in the exploratory cohort. In patients with a missing value of a fragment, ‘[mean value of the urinary protein fragment in the population] − [2 × standard deviation]’ was imputed. In the validation cohort, no value was imputed for missing fragments. Fold change (FC) with respect to the mean value in healthy subjects and p-value in two group comparisons of numerical data by a two-tailed Student’s t test were plotted as volcano plots in the exploratory and validation cohorts. Sensitivity, specificity and receiver operating characteristic (ROC) curves for discriminating resectable pancreatic cancer patients from healthy participants in the validation cohort were evaluated for each cancer-related fragment. All analyses were conducted using JMP® version 11 software (SAS Institute, Cary, NC, USA).

### Supplementary Information

Supplementary Information.

## Data Availability

The authors declare that the data supporting the findings of this study are available in the Supplementary Information. Source Data are also provided with this paper. MS proteomics data have been deposited with the ProteomeXchange Consortium (http://proteomecentral.proteomexchange.org) via the jPOST partner repository with the dataset identifier PXD041122 and JPST002103.
