# Supplementary material for: Identification of a urinary CD276 fragment for detecting resectable pancreatic cancer using a C-terminal proteomics strategy
Source: Sci Rep. 2024 Jun 20;14:14207. doi: 10.1038/s41598-024-65093-2 (PMC11190254; doi:10.1038/s41598-024-65093-2)
Supplement: Supplementary file 1 — Supplementary Information. [file 41598_2024_65093_MOESM1_ESM.docx]

# Supporting information

**Table of Contents**

| Supplementary methods | - Mass spectrometry and data processing |
| --- | --- |
| Supplementary figure legends | - Supplementary Figure S1 - Supplementary Figure S2 |
| Supplementary Figure S1. | Identification of #13684 peptide by a database search |
| Supplementary Figure S2. | Distribution of log2 FC for PDAC vs. healthy participants using PDAC-related fragments in exploratory and validation cohorts |
| Supplementary Table S1. | Patients’ characteristics |
| Supplementary Table S2. | Comparison of urinary protein fragments between healthy volunteers and patients with PDAC in the exploratory cohort |
| Supplementary Table S3. | Comparison of urinary protein fragments between healthy volunteers and patients with PDAC in the validation cohort |

## Supplementary methods

### Mass spectrometry and data processing

NanoLC fractions collected on a MALDI target plate were analyzed on an AB Sciex 4800 MALDI TOF/TOF mass spectrometer (Sciex). Samples were first subjected to MS (MS1) analysis in the reflector mode with α-cyano-4-hydroxycinnamic acid as the matrix. From each MS1 spectra, ^18^O-unlabelled peptides were listed and subjected to MS/MS (MS2) analysis. All MS2 spectra were saved as Data Explorer (AB Sciex) format files, from which a single peak list of MS2 peaks, including iTRAQ reporters, was created by Mascot distiller (Matrix Science) using Mascot daemon (Matrix Science) as the front end. The peak list was then subjected to a database search on MASCOT Server ver. 2.4 (Matrix Science) with the following parameters: Database: Swissprot, Taxonomy: Homo sapiens, Enzyme: none, Peptide charge: 1, Fixed modifications: propionamide (C), iTRAQ8plex (K) and iTRAQ8plex (N-term), Variable modifications: oxidation (M), oxidation (P), iTRAQ8plex (Y) and Label:18O [1] (C-term), Peptide tolerance: 0.3 Da, MS/MS tolerance: 0.3 Da. The search result was then combined with the MS2 peak list and stored in a PostgreSQL database (The PostgreSQL Global Development Group). The standard urine sample was a mixture of 105 urine samples from 45 non-cancer patients and 60 cancer patients and was labelled with the iTraq 113 reagent. The intensities of reporter ions were normalized first with the peptides in the standard urine sample. The reporter intensities were further standardized with the medians of each reporter ion (Supplemental Fig. S1). Each peptide was assigned peptide ID in a peptide library, as shown below, based on spectral similarity as judged by cosine distance [2]. Peptides that were ^18^O-labelled, having post-translational modification, that were not digested after K or R at N-termini, or failed to be identified were omitted from further analysis.

A library of tryptic peptides was created from all the MS/MS data in this study. In this library, each record had the precursor m/z value, top twenty MS2 m/z values and areas, retention time, and MASCOT search result. Peptide identity was judged by cosine distance, precursor m/z value and retention time. Each peptide record was given a non-overlapping identifier (ID), which was used to specify an individual peptide throughout this work. Data processing was carried out on Python 3.8 (Python Software Foundation, Beaverton, USA). To upload the MS/MS data to a data repository, MS/MS spectra of multiple .t2d format files were converted to a single .mzxml format file using PyMsXML 0.5.4 [3] running under Python 2.7.

1. Sobin, L., Wittekind, C. TNM classification of malignant tumors (ed 7) New York, NY, Wiley-Liss (2009).
2. Lam, H., et al. Development and validation of a spectral library searching method for peptide identification from MS/MS. *Proteomics*. **7**(5):655-667 (2007). DOI: 10.1002/pmic.200600625.
3. An, Y., Bekesova, S., Edwards, N., Goldman, R. Peptides in low molecular weight fraction of serum associated with hepatocellular carcinoma. *Dis. Markers.* **29**:11-20 (2010). DOI 10.3233/DMA-2010-0721

## Supplementary Figure Legends

### Supplementary Figure S1. Identification of #13684 peptide by a database search

An MS2 spectrum of the #13684 peptide ([M+H]^+^=1997.95) was analyzed by Mascot Distiller to create a peal list and perform a database search on a MASCOT server. The search results are represented as Protein Family Summary (a) and Peptide View (b). The search results showed a single significant hit, CD276 (242-258), with a peptide score of 92.

### Supplementary Figure S2. Distribution of log2 FC for PDAC vs. healthy participants using PDAC-related fragments in exploratory and validation cohorts

Depicted fragments have P<0.01 in comparison of their expressions between PDAC and healthy subjects in validation cohort. The x- and y-axes show the log2 FC in validation and exploratory cohorts, respectively.

Abbreviations: PDAC, pancreatic ductal adenocarcinoma; FC, fold change with respect to the mean value in healthy subjects.

## Supplementary Figure S1. Identification of #13684 peptide by a database search


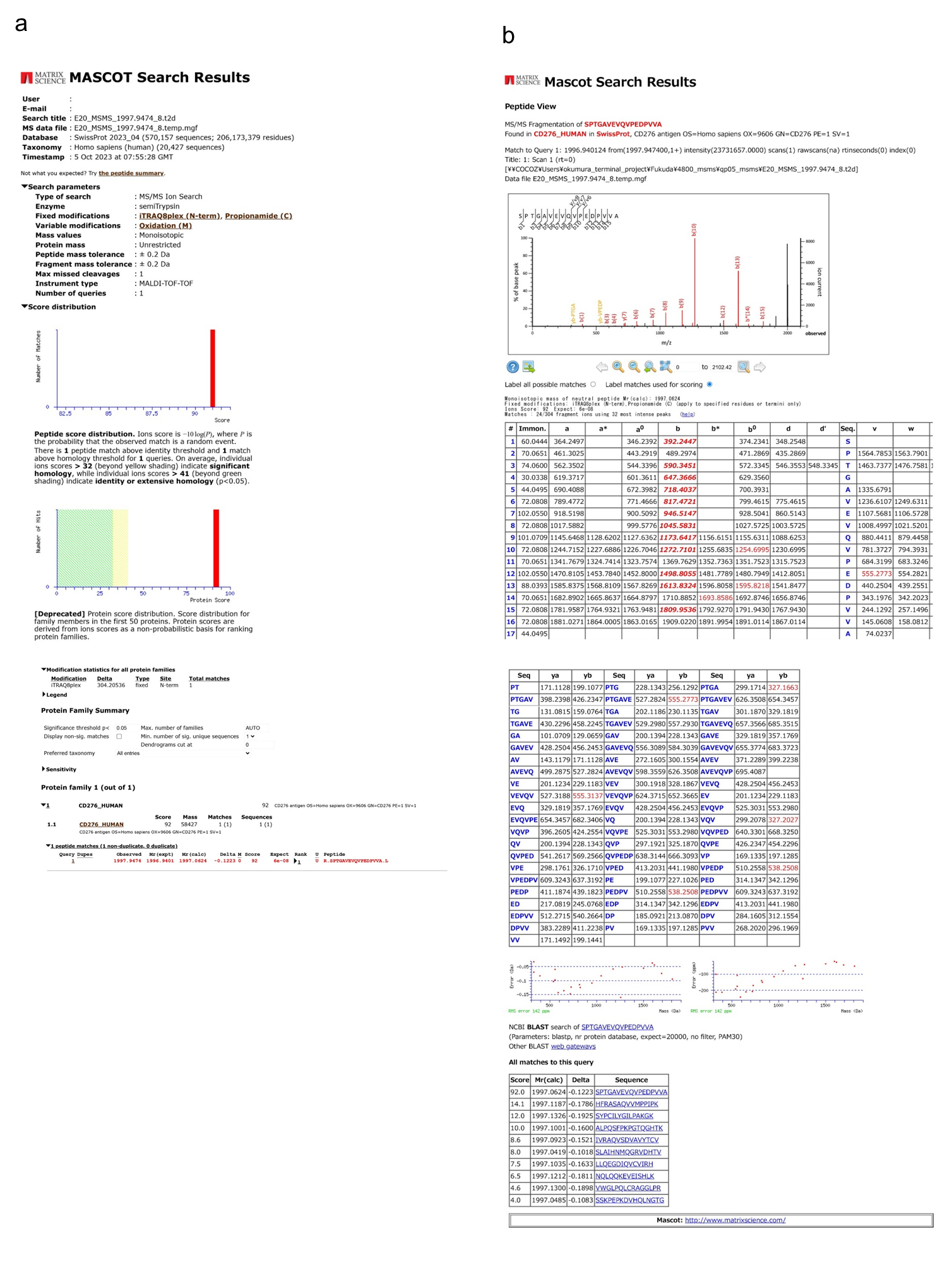


## Supplementary Figure S2. Distribution of log2 FC for PDAC vs. healthy participants using PDAC-related fragments in exploratory and validation cohorts


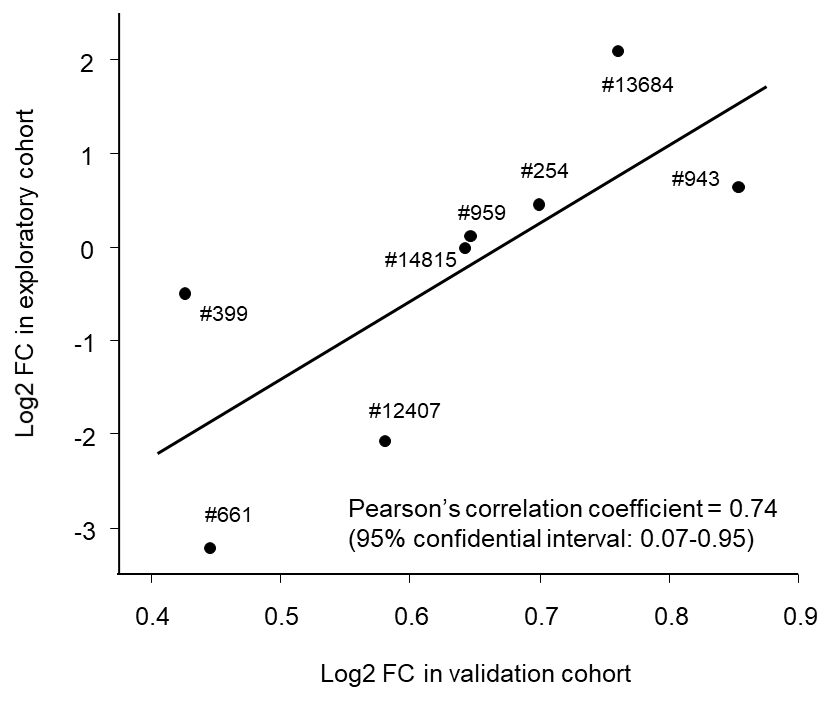


## Supplementary Table S1. Patients’ characteristics

| Variables | | Exploratory cohort | |  | Validation cohort | |
| --- | --- | --- | --- | --- | --- | --- |
|  |  | Healthy volunteers | PDAC |  | Healthy volunteers | Resectable PDAC |
|  |  | N=42 | N=39 |  | N=36 | N=28 |
|  |  | N (%) | |  | N (%) | |
| Age (years) | Median (IQR) | 54 (27-80) | 65 (22-79) |  | 57 (52-64) | 70 (66-74) |
| Gender | Male | 21 (50.0) | 27 (69.2) |  | 11 (30.6) | 14 (50.0) |
|  | Female | 21 (50.0) | 12 (30.8) |  | 25 (69.4) | 14 (50.0) |
| UICC Stage | I, II | - | 7 (17.9) |  | - | 27 (96.4) |
|  | III | - | 9 (23.1) |  | - | 1 (3.6) |
|  | IV | - | 23 (59.0) |  | - | 0 (0.0) |
| CA19-9 | Median (IQR) | 17.6 (6.8-31.7) | 329.2 (0.8-46543) |  | 9.5 (5.1-14.3) | 47.4 (12.2-215) |

Abbreviations: PDAC, pancreatic ductal adenocarcinoma; UICC, Union for International Cancer Control; IQR, interquartile range.

## Supplementary Table S2. Comparison of urinary protein fragments between healthy volunteers and patients with PDAC in the exploratory cohort

| Fragments | Healthy volunteers | | PDAC patients | | Log2 [FC] | -Log10 [P value] |
| --- | --- | --- | --- | --- | --- | --- |
|  | N | Mean (95%CI) | N | Mean (95%CI) |  |  |
| ｐ199 | 42 | 1 (0.61, 1.39) | 39 | 2.53 (2.13, 2.94) | 1.3411 | 6.2840 |
| ｐ191 | 42 | 1.83 (1.24, 2.42) | 39 | 4.08 (3.47, 4.69) | 1.1592 | 5.9918 |
| ｐ13684 | 42 | 0.31 (0.04, 0.58) | 39 | 1.31 (1.03, 1.59) | 2.0904 | 5.7247 |
| ｐ13341 | 42 | 0.51 (0.28, 0.74) | 39 | 1.37 (1.13, 1.61) | 1.4260 | 5.6844 |
| ｐ950 | 42 | 0.31 (0.2, 0.43) | 39 | 0.74 (0.62, 0.86) | 1.2332 | 5.5410 |
| ｐ14161 | 42 | 0.86 (0.6, 1.12) | 39 | 1.78 (1.51, 2.05) | 1.0454 | 5.3391 |
| ｐ22433 | 42 | 0.24 (0.15, 0.33) | 39 | 0.53 (0.44, 0.62) | 1.1449 | 4.8318 |
| ｐ2295 | 42 | 0.68 (0.58, 0.79) | 39 | 1.02 (0.91, 1.12) | 0.5760 | 4.6516 |
| ｐ3110 | 42 | 0.36 (0.19, 0.53) | 39 | 0.9 (0.73, 1.08) | 1.3193 | 4.5124 |
| ｐ271 | 42 | 0.56 (0.34, 0.78) | 39 | 1.26 (1.03, 1.49) | 1.1744 | 4.4615 |
| ｐ738 | 42 | 0.19 (0.15, 0.24) | 39 | 0.05 (0, 0.1) | -1.9718 | 4.4137 |
| ｐ22515 | 42 | 0.45 (0.35, 0.55) | 39 | 0.76 (0.66, 0.87) | 0.7593 | 4.4079 |
| ｐ166 | 42 | 1.57 (0.78, 2.36) | 39 | 4.04 (3.22, 4.86) | 1.3664 | 4.3698 |
| ｐ7498 | 42 | 0.39 (0.32, 0.45) | 39 | 0.18 (0.11, 0.25) | -1.0930 | 4.3448 |
| ｐ1769 | 42 | 0.49 (0.44, 0.54) | 39 | 0.33 (0.28, 0.38) | -0.5700 | 4.3242 |
| ｐ74 | 41 | 0.08 (-0.12, 0.29) | 39 | 0.68 (0.48, 0.89) | 3.0135 | 4.0540 |
| ｐ22430 | 42 | 0.44 (0.3, 0.57) | 39 | 0.84 (0.7, 0.98) | 0.9385 | 4.0318 |
| ｐ19994 | 42 | 0.08 (-0.03, 0.19) | 39 | 0.41 (0.3, 0.53) | 2.4151 | 4.0000 |
| ｐ15475 | 42 | 0.09 (-0.02, 0.2) | 39 | 0.47 (0.36, 0.59) | 2.3350 | 4.0000 |
| ｐ16292 | 42 | 0.25 (0.01, 0.49) | 39 | 0.98 (0.73, 1.23) | 1.9741 | 4.0000 |
| ｐ15039 | 42 | 0.66 (0.44, 0.89) | 39 | 1.44 (1.21, 1.67) | 1.1170 | 4.0000 |
| ｐ15057 | 42 | 0.27 (0.19, 0.36) | 39 | 0.58 (0.5, 0.67) | 1.0901 | 4.0000 |
| ｐ18290 | 42 | 0.51 (0.37, 0.66) | 39 | 0.93 (0.78, 1.08) | 0.8643 | 4.0000 |
| ｐ15120 | 42 | 0.39 (0.3, 0.48) | 39 | 0.68 (0.59, 0.78) | 0.8236 | 4.0000 |
| ｐ22414 | 42 | 0.02 (-0.09, 0.12) | 39 | 0.32 (0.21, 0.42) | 4.2423 | 3.9304 |
| ｐ13010 | 42 | 0.6 (0.32, 0.88) | 39 | 1.42 (1.13, 1.71) | 1.2383 | 3.8505 |
| ｐ20382 | 42 | 0.15 (-0.02, 0.32) | 39 | 0.63 (0.46, 0.8) | 2.0620 | 3.8194 |
| ｐ16203 | 42 | 0.21 (0.05, 0.36) | 39 | 0.64 (0.48, 0.8) | 1.6340 | 3.6990 |
| ｐ10210 | 42 | 0.57 (0.34, 0.8) | 39 | 1.22 (0.98, 1.46) | 1.0896 | 3.6963 |
| ｐ6038 | 42 | 0.44 (0.29, 0.59) | 39 | 0.86 (0.71, 1.02) | 0.9687 | 3.6925 |
| ｐ869 | 42 | 0.81 (0.4, 1.21) | 39 | 1.91 (1.49, 2.33) | 1.2438 | 3.4995 |
| ｐ13745 | 42 | 0.7 (0.48, 0.91) | 39 | 1.29 (1.06, 1.51) | 0.8887 | 3.4958 |
| ｐ20025 | 42 | 0.26 (0.15, 0.37) | 39 | 0.55 (0.44, 0.66) | 1.1026 | 3.4650 |
| ｐ10810 | 42 | 0.4 (0.28, 0.51) | 39 | 0.72 (0.59, 0.84) | 0.8605 | 3.4360 |
| ｐ13283 | 42 | 0.28 (0.2, 0.35) | 39 | 0.48 (0.4, 0.55) | 0.7766 | 3.3989 |
| ｐ22823 | 42 | 0.28 (0.2, 0.35) | 39 | 0.48 (0.4, 0.55) | 0.7766 | 3.3989 |
| ｐ18932 | 42 | 0.46 (-0.29, 1.2) | 39 | 2.46 (1.68, 3.23) | 2.4252 | 3.3979 |
| ｐ15912 | 42 | 0.24 (0.1, 0.39) | 39 | 0.64 (0.49, 0.79) | 1.3935 | 3.3979 |
| ｐ17430 | 42 | 0.29 (0.24, 0.34) | 39 | 0.42 (0.37, 0.47) | 0.5389 | 3.3979 |
| ｐ13677 | 42 | 0.52 (0.29, 0.75) | 39 | 1.14 (0.9, 1.37) | 1.1225 | 3.3533 |
| ｐ10954 | 42 | 0.3 (0.03, 0.57) | 39 | 1.02 (0.74, 1.31) | 1.7731 | 3.3521 |
| ｐ21725 | 42 | 0.57 (0.43, 0.72) | 39 | 0.96 (0.81, 1.11) | 0.7395 | 3.3320 |
| ｐ11692 | 42 | 0.45 (0.36, 0.54) | 39 | 0.22 (0.13, 0.31) | -1.0295 | 3.3268 |
| ｐ777 | 42 | 0.38 (0.3, 0.46) | 39 | 0.17 (0.09, 0.25) | -1.1581 | 3.2857 |
| ｐ21190 | 42 | 0.29 (0.23, 0.35) | 39 | 0.44 (0.38, 0.5) | 0.6105 | 3.2263 |
| ｐ15108 | 42 | 0.41 (0.22, 0.59) | 39 | 0.89 (0.7, 1.08) | 1.1335 | 3.2218 |
| ｐ2296 | 42 | 0.51 (0.43, 0.58) | 39 | 0.31 (0.24, 0.39) | -0.6899 | 3.2209 |
| ｐ22542 | 42 | 0.28 (0.23, 0.33) | 39 | 0.41 (0.35, 0.46) | 0.5570 | 3.1830 |
| ｐ804 | 42 | 1.08 (0.81, 1.35) | 39 | 1.78 (1.5, 2.06) | 0.7218 | 3.1722 |
| ｐ15993 | 42 | 0.27 (0.01, 0.53) | 39 | 0.94 (0.67, 1.21) | 1.7837 | 3.1549 |
| ｐ19713 | 42 | 0.71 (0.51, 0.92) | 39 | 1.24 (1.03, 1.45) | 0.7959 | 3.1549 |
| ｐ9533 | 42 | 0.3 (0.24, 0.35) | 39 | 0.45 (0.38, 0.51) | 0.5872 | 3.1488 |
| ｐ12298 | 42 | 0.69 (0.48, 0.9) | 39 | 1.23 (1.01, 1.45) | 0.8288 | 3.1298 |
| ｐ15335 | 42 | 0.35 (0.25, 0.46) | 39 | 0.09 (-0.02, 0.2) | -1.9658 | 3.0969 |
| ｐ22187 | 42 | 0.41 (0.15, 0.68) | 39 | 1.08 (0.8, 1.35) | 1.3876 | 3.0606 |
| ｐ18924 | 42 | 0.36 (0.21, 0.51) | 39 | 0.74 (0.58, 0.89) | 1.0234 | 3.0458 |
| ｐ7819 | 42 | 0.21 (0.01, 0.41) | 39 | 0.71 (0.5, 0.91) | 1.7459 | 3.0407 |
| ｐ22008 | 42 | 0.06 (-0.05, 0.17) | 39 | 0.34 (0.22, 0.45) | 2.4123 | 3.0330 |
| ｐ1021 | 40 | 0.98 (-2.19, 4.16) | 36 | 8.98 (5.63, 12.32) | 3.1895 | 3.0300 |
| ｐ4210 | 42 | 0.25 (0.19, 0.31) | 39 | 0.09 (0.03, 0.16) | -1.4165 | 3.0180 |
| ｐ14886 | 42 | 0.61 (0.58, 0.65) | 39 | 0.54 (0.5, 0.57) | -0.1974 | 2.9667 |
| ｐ16647 | 42 | 0.2 (-0.08, 0.49) | 39 | 0.9 (0.61, 1.19) | 2.1477 | 2.9586 |
| ｐ17947 | 42 | 0.36 (0.26, 0.45) | 39 | 0.12 (0.02, 0.22) | -1.5964 | 2.9586 |
| ｐ2418 | 42 | 0.76 (0.56, 0.96) | 39 | 1.25 (1.04, 1.45) | 0.7141 | 2.9431 |
| ｐ22727 | 42 | 0.42 (0.36, 0.48) | 39 | 0.57 (0.5, 0.63) | 0.4181 | 2.9075 |
| ｐ10581 | 42 | 0.43 (0.37, 0.49) | 39 | 0.28 (0.21, 0.34) | -0.6288 | 2.9029 |
| ｐ18384 | 42 | 0.12 (0.09, 0.16) | 39 | 0.03 (-0.01, 0.07) | -2.0294 | 2.8861 |
| ｐ22676 | 42 | 0.68 (0.57, 0.79) | 39 | 0.94 (0.83, 1.06) | 0.4755 | 2.8661 |
| ｐ15403 | 42 | 0.27 (0.04, 0.5) | 39 | 0.81 (0.58, 1.04) | 1.5900 | 2.8539 |
| ｐ14964 | 42 | 0.63 (0.55, 0.7) | 39 | 0.81 (0.73, 0.89) | 0.3682 | 2.8285 |
| ｐ1227 | 42 | 0.04 (-0.09, 0.17) | 39 | 0.35 (0.21, 0.48) | 3.2576 | 2.8285 |
| ｐ20246 | 42 | 0.29 (0.24, 0.34) | 39 | 0.41 (0.36, 0.46) | 0.5120 | 2.8285 |
| ｐ21657 | 42 | 0.07 (0.01, 0.14) | 39 | 0.23 (0.16, 0.29) | 1.6515 | 2.8285 |
| ｐ9019 | 42 | 0.48 (0.45, 0.51) | 39 | 0.56 (0.52, 0.59) | 0.2183 | 2.8285 |
| ｐ15527 | 42 | 0.24 (0.19, 0.29) | 39 | 0.36 (0.31, 0.42) | 0.6024 | 2.8239 |
| ｐ13831 | 42 | 0.48 (0.35, 0.62) | 39 | 0.8 (0.66, 0.94) | 0.7305 | 2.7954 |
| ｐ2291 | 42 | 0.27 (0.22, 0.32) | 39 | 0.14 (0.09, 0.2) | -0.9055 | 2.7850 |
| ｐ22373 | 42 | 0.57 (0.45, 0.7) | 39 | 0.86 (0.74, 0.99) | 0.5934 | 2.7738 |
| ｐ182 | 42 | 0.51 (0.27, 0.74) | 39 | 1.05 (0.81, 1.29) | 1.0479 | 2.7343 |
| ｐ20374 | 42 | 0.33 (0.15, 0.5) | 39 | 0.74 (0.56, 0.92) | 1.1737 | 2.7278 |
| ｐ19289 | 42 | 0.77 (0.72, 0.82) | 39 | 0.65 (0.6, 0.7) | -0.2339 | 2.7212 |
| ｐ8773 | 42 | 0.68 (0.61, 0.75) | 39 | 0.52 (0.45, 0.59) | -0.3848 | 2.7200 |
| ｐ14681 | 39 | 0.33 (-0.06, 0.72) | 39 | 1.2 (0.82, 1.59) | 1.8828 | 2.6756 |
| ｐ801 | 42 | 2.23 (1.18, 3.28) | 39 | 4.63 (3.55, 5.72) | 1.0569 | 2.6655 |
| ｐ22605 | 42 | 0.92 (0.58, 1.26) | 39 | 1.7 (1.35, 2.06) | 0.8874 | 2.6485 |
| ｐ18513 | 42 | 0 (-0.06, 0.06) | 39 | 0.15 (0.08, 0.21) | NA | 2.6383 |
| ｐ15213 | 42 | 0.42 (0.29, 0.55) | 39 | 0.13 (-0.01, 0.26) | -1.7443 | 2.6198 |
| ｐ11585 | 42 | 0.52 (0.35, 0.69) | 39 | 0.13 (-0.04, 0.31) | -1.9652 | 2.6091 |
| ｐ39 | 42 | 0.87 (0.52, 1.21) | 39 | 1.64 (1.29, 2) | 0.9193 | 2.5817 |
| ｐ2595 | 42 | 0.09 (-0.15, 0.33) | 39 | 0.62 (0.37, 0.87) | 2.8446 | 2.5334 |
| ｐ8495 | 42 | 0.52 (0.38, 0.67) | 39 | 0.2 (0.05, 0.35) | -1.4041 | 2.5256 |
| ｐ12606 | 42 | 0.25 (0.17, 0.34) | 39 | 0.06 (-0.03, 0.15) | -1.9988 | 2.5187 |
| ｐ4642 | 39 | 0.16 (-3.81, 4.12) | 39 | 8.76 (4.8, 12.73) | 5.8019 | 2.5119 |
| ｐ6293 | 42 | 0.72 (0.67, 0.77) | 39 | 0.61 (0.55, 0.66) | -0.2457 | 2.5014 |
| ｐ1899 | 42 | 0.43 (0.18, 0.68) | 39 | 0.99 (0.72, 1.25) | 1.2035 | 2.4987 |
| ｐ13098 | 42 | 0.44 (0.26, 0.61) | 39 | 0.83 (0.64, 1.01) | 0.9200 | 2.4965 |
| ｐ79 | 41 | 0.49 (0.32, 0.65) | 39 | 0.84 (0.68, 1.01) | 0.7981 | 2.4956 |
| ｐ21289 | 42 | 0.66 (0.59, 0.73) | 39 | 0.82 (0.74, 0.89) | 0.3113 | 2.4864 |
| ｐ15306 | 42 | 0.34 (0.26, 0.42) | 39 | 0.16 (0.08, 0.24) | -1.0771 | 2.4815 |
| ｐ13623 | 42 | 0.26 (0.16, 0.37) | 39 | 0.04 (-0.07, 0.14) | -2.8847 | 2.4800 |
| ｐ11516 | 42 | 0.33 (0.3, 0.36) | 39 | 0.27 (0.24, 0.3) | -0.2984 | 2.4800 |
| ｐ15509 | 41 | 1.75 (1.02, 2.49) | 39 | 3.34 (2.59, 4.1) | 0.9326 | 2.4685 |
| ｐ22729 | 42 | 0.3 (0.11, 0.49) | 39 | 0.71 (0.51, 0.91) | 1.2513 | 2.4597 |
| ｐ14846 | 42 | 0.24 (-0.35, 0.84) | 39 | 1.54 (0.92, 2.16) | 2.6593 | 2.4581 |
| ｐ736 | 42 | 0.19 (0.14, 0.24) | 39 | 0.09 (0.04, 0.14) | -1.1114 | 2.4202 |
| ｐ14531 | 42 | 0.56 (0.38, 0.73) | 39 | 0.93 (0.75, 1.11) | 0.7423 | 2.4147 |
| ｐ16652 | 42 | 0.34 (0.17, 0.52) | 39 | 0.71 (0.54, 0.89) | 1.0597 | 2.4089 |
| ｐ13242 | 42 | 0.35 (0.22, 0.49) | 39 | 0.08 (-0.06, 0.21) | -2.2409 | 2.3809 |
| ｐ11456 | 42 | 1.24 (0.72, 1.76) | 39 | 2.35 (1.81, 2.89) | 0.9260 | 2.3747 |
| ｐ15159 | 42 | 0.33 (0.18, 0.48) | 39 | 0.65 (0.49, 0.8) | 0.9723 | 2.3665 |
| ｐ22397 | 42 | 0.22 (-1.04, 1.48) | 39 | 2.89 (1.58, 4.19) | 3.7185 | 2.3558 |
| ｐ11259 | 42 | 1.91 (1.24, 2.58) | 39 | 3.32 (2.63, 4.02) | 0.7967 | 2.3314 |
| ｐ15431 | 42 | 0.68 (0.62, 0.74) | 39 | 0.81 (0.75, 0.87) | 0.2428 | 2.3279 |
| ｐ13680 | 42 | 0.44 (0.15, 0.72) | 39 | 1.04 (0.74, 1.34) | 1.2553 | 2.3209 |
| ｐ46 | 42 | 0.31 (-0.22, 0.84) | 39 | 1.43 (0.88, 1.98) | 2.1955 | 2.3208 |
| ｐ10160 | 42 | 0.22 (-0.03, 0.47) | 39 | 0.74 (0.48, 1) | 1.7364 | 2.3042 |
| ｐ3056 | 42 | 0.39 (0.23, 0.55) | 39 | 0.72 (0.56, 0.88) | 0.8859 | 2.2950 |
| ｐ16473 | 42 | 0.68 (0.58, 0.77) | 39 | 0.88 (0.78, 0.98) | 0.3767 | 2.2924 |
| ｐ118 | 42 | 0.08 (-0.25, 0.41) | 39 | 0.78 (0.43, 1.12) | 3.2845 | 2.2921 |
| ｐ22062 | 42 | 0.33 (0.1, 0.57) | 39 | 0.82 (0.58, 1.06) | 1.3035 | 2.2910 |
| ｐ22175 | 42 | 0.46 (0.43, 0.49) | 39 | 0.53 (0.49, 0.56) | 0.1954 | 2.2900 |
| ｐ22779 | 42 | 1.42 (1.35, 1.49) | 39 | 1.56 (1.49, 1.63) | 0.1355 | 2.2900 |
| ｐ20441 | 42 | 0.11 (-0.02, 0.23) | 39 | 0.37 (0.24, 0.5) | 1.7562 | 2.2900 |
| ｐ22763 | 42 | 0.53 (0.47, 0.58) | 39 | 0.64 (0.58, 0.69) | 0.2747 | 2.2900 |
| ｐ22678 | 42 | 0.31 (0.22, 0.41) | 39 | 0.51 (0.41, 0.6) | 0.7019 | 2.2900 |
| ｐ22778 | 42 | 0.78 (0.73, 0.84) | 39 | 0.9 (0.84, 0.96) | 0.2035 | 2.2900 |
| ｐ190 | 41 | 0.21 (0.02, 0.41) | 39 | 0.61 (0.41, 0.81) | 1.5245 | 2.2482 |
| ｐ13769 | 42 | 0.55 (0.52, 0.58) | 39 | 0.49 (0.46, 0.52) | -0.1580 | 2.2461 |
| ｐ14863 | 42 | 0.44 (0.37, 0.51) | 39 | 0.29 (0.22, 0.37) | -0.5902 | 2.2461 |
| ｐ21185 | 42 | 0.25 (0.18, 0.33) | 39 | 0.09 (0.01, 0.17) | -1.4752 | 2.2461 |
| ｐ11672 | 42 | 0.38 (0.33, 0.44) | 39 | 0.28 (0.22, 0.33) | -0.4793 | 2.2461 |
| ｐ842 | 42 | 0.42 (0.23, 0.62) | 39 | 0.82 (0.62, 1.02) | 0.9513 | 2.2448 |
| ｐ17991 | 42 | 0.3 (0.25, 0.36) | 39 | 0.19 (0.13, 0.25) | -0.6781 | 2.2441 |
| ｐ10347 | 42 | 0.47 (0.3, 0.63) | 39 | 0.81 (0.64, 0.99) | 0.8019 | 2.2365 |
| ｐ4140 | 42 | 0.12 (0.06, 0.18) | 39 | 0.24 (0.18, 0.3) | 0.9675 | 2.2327 |
| ｐ7815 | 42 | 0.28 (0.13, 0.43) | 39 | 0.58 (0.43, 0.73) | 1.0540 | 2.2292 |
| ｐ12109 | 42 | 0.52 (-0.59, 1.63) | 39 | 2.79 (1.64, 3.95) | 2.4228 | 2.2231 |
| ｐ9205 | 42 | 0.52 (0.4, 0.63) | 39 | 0.75 (0.63, 0.87) | 0.5355 | 2.2186 |
| ｐ21744 | 42 | 0.26 (0.05, 0.48) | 39 | 0.7 (0.48, 0.92) | 1.4047 | 2.2164 |
| ｐ15409 | 42 | 0.26 (0.05, 0.48) | 39 | 0.7 (0.48, 0.92) | 1.4047 | 2.2147 |
| ｐ17391 | 42 | 0.42 (0.16, 0.68) | 39 | 0.95 (0.68, 1.21) | 1.1673 | 2.2147 |
| ｐ15908 | 42 | 0.66 (0.48, 0.83) | 39 | 0.3 (0.12, 0.48) | -1.1268 | 2.2147 |
| ｐ21370 | 42 | 0.66 (0.48, 0.83) | 39 | 0.3 (0.12, 0.48) | -1.1268 | 2.2138 |
| ｐ13702 | 42 | 0.49 (0.41, 0.57) | 39 | 0.33 (0.25, 0.41) | -0.5800 | 2.1956 |
| ｐ12856 | 42 | 0.26 (0.15, 0.36) | 39 | 0.04 (-0.06, 0.15) | -2.5228 | 2.1920 |
| ｐ175 | 42 | 1.26 (0.92, 1.59) | 39 | 1.94 (1.59, 2.29) | 0.6255 | 2.1786 |
| ｐ17864 | 41 | 0.46 (0.16, 0.75) | 39 | 1.05 (0.75, 1.35) | 1.1932 | 2.1739 |
| ｐ15842 | 42 | 0.8 (0.56, 1.04) | 39 | 1.28 (1.03, 1.52) | 0.6789 | 2.1739 |
| ｐ230 | 42 | 0.73 (0.52, 0.94) | 39 | 1.16 (0.94, 1.38) | 0.6593 | 2.1697 |
| ｐ22916 | 42 | 0.66 (0.1, 1.22) | 39 | 1.79 (1.2, 2.37) | 1.4408 | 2.1588 |
| ｐ21368 | 42 | 0.79 (0.53, 1.05) | 39 | 0.26 (-0.01, 0.53) | -1.6024 | 2.1501 |
| ｐ3072 | 42 | 0.08 (-0.13, 0.3) | 39 | 0.51 (0.29, 0.73) | 2.5940 | 2.1492 |
| ｐ16641 | 42 | 0.24 (0.08, 0.41) | 39 | 0.57 (0.4, 0.74) | 1.2188 | 2.1487 |
| ｐ13050 | 42 | 0.33 (0.22, 0.43) | 39 | 0.11 (0, 0.22) | -1.5176 | 2.1415 |
| ｐ19288 | 42 | 0.25 (0.17, 0.34) | 39 | 0.43 (0.34, 0.52) | 0.7557 | 2.1367 |
| ｐ5033 | 42 | 0.09 (0.02, 0.17) | 39 | 0.24 (0.16, 0.31) | 1.3558 | 2.1343 |
| ｐ14782 | 42 | 2.02 (1.11, 2.94) | 39 | 3.84 (2.89, 4.79) | 0.9252 | 2.1271 |
| ｐ14129 | 42 | 0.35 (-0.11, 0.82) | 39 | 1.27 (0.79, 1.75) | 1.8480 | 2.1205 |
| ｐ22822 | 42 | 0.37 (0.34, 0.41) | 39 | 0.44 (0.4, 0.47) | 0.2328 | 2.1050 |
| ｐ15822 | 42 | 0.37 (0.04, 0.71) | 39 | 1.04 (0.69, 1.38) | 1.4680 | 2.0969 |
| ｐ16729 | 42 | 0.3 (0.11, 0.5) | 39 | 0.69 (0.48, 0.89) | 1.1898 | 2.0969 |
| ｐ4814 | 42 | 0.75 (0.65, 0.85) | 39 | 0.56 (0.46, 0.66) | -0.4292 | 2.0958 |
| ｐ20423 | 42 | 0.31 (0.24, 0.38) | 39 | 0.16 (0.09, 0.24) | -0.9171 | 2.0921 |
| ｐ12166 | 42 | 0.35 (0.22, 0.48) | 39 | 0.61 (0.47, 0.75) | 0.8041 | 2.0914 |
| ｐ11490 | 42 | 0.74 (0.54, 0.94) | 39 | 0.34 (0.13, 0.55) | -1.1189 | 2.0896 |
| ｐ12760 | 42 | 1.23 (0.83, 1.62) | 39 | 2 (1.59, 2.4) | 0.7042 | 2.0888 |
| ｐ329 | 42 | 0.81 (0.64, 0.97) | 39 | 0.48 (0.31, 0.65) | -0.7422 | 2.0870 |
| ｐ22055 | 42 | 0.25 (-0.24, 0.74) | 39 | 1.21 (0.7, 1.71) | 2.2716 | 2.0863 |
| ｐ4891 | 39 | 1.37 (-0.07, 2.8) | 39 | 4.13 (2.69, 5.56) | 1.5952 | 2.0847 |
| ｐ14974 | 42 | 0.3 (0.23, 0.38) | 39 | 0.15 (0.08, 0.23) | -0.9772 | 2.0836 |
| ｐ22335 | 42 | 0.35 (0.24, 0.46) | 39 | 0.14 (0.03, 0.25) | -1.3233 | 2.0791 |
| ｐ15677 | 42 | 0.29 (0.05, 0.53) | 39 | 0.75 (0.51, 1) | 1.3951 | 2.0655 |
| ｐ12241 | 42 | 0.86 (0.61, 1.12) | 39 | 0.37 (0.1, 0.63) | -1.2388 | 2.0536 |
| ｐ11766 | 42 | 0.43 (0.38, 0.48) | 39 | 0.52 (0.47, 0.57) | 0.2810 | 2.0519 |
| ｐ13088 | 42 | 0.54 (0.38, 0.7) | 39 | 0.23 (0.07, 0.4) | -1.1995 | 2.0486 |
| ｐ22522 | 42 | 0.59 (0.45, 0.73) | 39 | 0.32 (0.18, 0.47) | -0.8683 | 2.0478 |
| ｐ13252 | 42 | 0.11 (-0.14, 0.36) | 39 | 0.6 (0.34, 0.86) | 2.4627 | 2.0406 |
| ｐ7944 | 42 | 0.26 (0.15, 0.37) | 39 | 0.47 (0.36, 0.58) | 0.8611 | 2.0336 |
| ｐ11405 | 42 | 0.26 (0.22, 0.29) | 39 | 0.33 (0.29, 0.36) | 0.3484 | 2.0242 |
| ｐ19814 | 42 | 0.6 (0.55, 0.65) | 39 | 0.7 (0.65, 0.75) | 0.2082 | 2.0177 |
| ｐ16988 | 42 | 0.7 (0.69, 0.71) | 39 | 0.68 (0.67, 0.69) | -0.0495 | 2.0177 |
| ｐ15281 | 42 | 0.38 (0.3, 0.46) | 39 | 0.22 (0.14, 0.31) | -0.7824 | 2.0177 |
| ｐ15779 | 42 | 0.23 (0.16, 0.3) | 39 | 0.1 (0.03, 0.17) | -1.1967 | 2.0177 |
| ｐ16375 | 42 | 0.23 (0.16, 0.3) | 39 | 0.1 (0.03, 0.17) | -1.1967 | 2.0177 |
| ｐ14828 | 42 | 0.31 (0.23, 0.39) | 39 | 0.15 (0.07, 0.24) | -1.0165 | 2.0176 |
| ｐ11151 | 42 | 0.54 (0.51, 0.57) | 39 | 0.48 (0.45, 0.52) | -0.1633 | 2.0176 |
| ｐ12768 | 42 | 0.47 (0.39, 0.55) | 39 | 0.32 (0.24, 0.4) | -0.5613 | 2.0176 |
| ｐ11826 | 42 | 0.23 (0.16, 0.3) | 39 | 0.1 (0.03, 0.17) | -1.1967 | 2.0176 |
| ｐ20162 | 42 | 0.01 (-0.2, 0.22) | 39 | 0.42 (0.2, 0.64) | 5.0006 | 2.0101 |
| ｐ11063 | 42 | 0.38 (0.28, 0.49) | 39 | 0.19 (0.08, 0.29) | -1.0247 | 2.0055 |
| ｐ238 | 42 | 0.84 (0.63, 1.05) | 39 | 0.43 (0.21, 0.65) | -0.9536 | 2.0034 |
| ｐ19901 | 42 | 0.12 (0.02, 0.22) | 39 | 0.31 (0.21, 0.41) | 1.3375 | 2.0000 |
| ｐ235 | 42 | 0.76 (0.5, 1.02) | 39 | 1.25 (0.98, 1.52) | 0.7252 | 1.9999 |
| ｐ2384 | 42 | 0.45 (0.31, 0.6) | 39 | 0.18 (0.03, 0.33) | -1.3304 | 1.9937 |
| ｐ20602 | 42 | 0.11 (-0.14, 0.37) | 39 | 0.6 (0.33, 0.86) | 2.3818 | 1.9876 |
| ｐ13429 | 42 | 0.55 (0.45, 0.65) | 39 | 0.36 (0.26, 0.47) | -0.6069 | 1.9809 |
| ｐ14754 | 42 | 0.52 (0.38, 0.67) | 39 | 0.26 (0.11, 0.4) | -1.0297 | 1.9655 |
| ｐ16133 | 42 | 0.7 (0.54, 0.86) | 39 | 1 (0.84, 1.17) | 0.5180 | 1.9586 |
| ｐ15483 | 42 | 0.63 (0.61, 0.65) | 39 | 0.67 (0.65, 0.69) | 0.0800 | 1.9586 |
| ｐ9494 | 42 | 0.46 (0.39, 0.52) | 39 | 0.57 (0.51, 0.64) | 0.3365 | 1.9551 |
| ｐ17201 | 42 | 0.33 (0.25, 0.42) | 39 | 0.49 (0.4, 0.58) | 0.5665 | 1.9508 |
| ｐ172 | 42 | 0.44 (0.22, 0.66) | 39 | 0.03 (-0.2, 0.25) | -4.1391 | 1.9469 |
| ｐ20189 | 42 | 0.31 (0.24, 0.39) | 39 | 0.45 (0.37, 0.53) | 0.5246 | 1.9441 |
| ｐ10172 | 42 | 0.47 (0.25, 0.69) | 39 | 0.88 (0.65, 1.11) | 0.9007 | 1.9400 |
| ｐ13311 | 42 | 0.31 (0.23, 0.38) | 39 | 0.17 (0.1, 0.24) | -0.8424 | 1.9400 |
| ｐ19023 | 42 | 0.83 (0.65, 1) | 39 | 0.5 (0.32, 0.68) | -0.7211 | 1.9393 |
| ｐ22137 | 42 | 0.48 (0.16, 0.81) | 39 | 1.09 (0.75, 1.42) | 1.1733 | 1.9393 |
| ｐ1238 | 42 | 0.37 (0.26, 0.48) | 39 | 0.17 (0.05, 0.28) | -1.1523 | 1.9319 |
| ｐ22337 | 42 | 0.59 (0.56, 0.62) | 39 | 0.65 (0.61, 0.68) | 0.1356 | 1.9216 |
| ｐ4452 | 42 | 0.79 (0.7, 0.88) | 39 | 0.63 (0.54, 0.72) | -0.3333 | 1.9175 |
| ｐ15477 | 42 | 0.5 (0.38, 0.62) | 39 | 0.28 (0.15, 0.4) | -0.8565 | 1.9136 |
| ｐ17869 | 42 | 0.42 (0.26, 0.59) | 39 | 0.11 (-0.06, 0.29) | -1.8986 | 1.9136 |
| ｐ15006 | 42 | 1.33 (0.76, 1.91) | 39 | 0.26 (-0.34, 0.86) | -2.3563 | 1.9101 |
| ｐ18038 | 42 | 0.43 (0.28, 0.57) | 39 | 0.69 (0.54, 0.84) | 0.6931 | 1.9066 |
| ｐ20043 | 42 | 0.36 (0.23, 0.49) | 39 | 0.61 (0.47, 0.75) | 0.7579 | 1.9064 |
| ｐ21966 | 42 | 0.48 (0.41, 0.54) | 39 | 0.36 (0.29, 0.43) | -0.4129 | 1.9033 |
| ｐ4848 | 42 | 0 (-0.1, 0.1) | 39 | 0.18 (0.08, 0.27) | NA | 1.8829 |
| ｐ18750 | 42 | 0.41 (0.35, 0.46) | 39 | 0.51 (0.45, 0.57) | 0.3323 | 1.8794 |
| ｐ4705 | 42 | 0.43 (0.35, 0.5) | 39 | 0.56 (0.48, 0.63) | 0.3852 | 1.8784 |
| ｐ17527 | 42 | 0.72 (0.59, 0.85) | 39 | 0.48 (0.35, 0.62) | -0.5675 | 1.8665 |
| ｐ22739 | 42 | 0.64 (0.43, 0.84) | 39 | 1.01 (0.8, 1.23) | 0.6637 | 1.8578 |
| ｐ721 | 42 | 0.23 (0.17, 0.3) | 39 | 0.35 (0.28, 0.42) | 0.5931 | 1.8572 |
| ｐ15570 | 42 | 0.57 (0.51, 0.63) | 39 | 0.68 (0.62, 0.75) | 0.2594 | 1.8570 |
| ｐ1581 | 42 | 0.48 (0.3, 0.66) | 39 | 0.81 (0.62, 0.99) | 0.7430 | 1.8556 |
| ｐ7080 | 42 | 0.25 (0.07, 0.43) | 39 | 0.58 (0.39, 0.77) | 1.2150 | 1.8555 |
| ｐ15173 | 42 | 0.37 (0.23, 0.5) | 39 | 0.12 (-0.02, 0.26) | -1.5789 | 1.8477 |
| ｐ15569 | 42 | 0.6 (0.55, 0.64) | 39 | 0.68 (0.63, 0.72) | 0.1830 | 1.8386 |
| ｐ5603 | 42 | 0.18 (0.05, 0.32) | 39 | 0.43 (0.29, 0.58) | 1.2336 | 1.8379 |
| ｐ16638 | 42 | 0.18 (-0.25, 0.61) | 39 | 0.96 (0.51, 1.4) | 2.3980 | 1.8327 |
| ｐ14762 | 42 | 0.13 (0.01, 0.24) | 39 | 0.33 (0.22, 0.45) | 1.4041 | 1.8320 |
| ｐ14807 | 42 | 0.65 (0.57, 0.73) | 39 | 0.51 (0.43, 0.59) | -0.3459 | 1.8291 |
| ｐ21236 | 42 | 0.39 (0.14, 0.64) | 39 | 0.84 (0.58, 1.1) | 1.1020 | 1.8284 |
| ｐ21015 | 42 | 0.2 (0.09, 0.3) | 39 | 0 (-0.11, 0.11) | NA | 1.8274 |
| ｐ10170 | 42 | 0.28 (0.12, 0.43) | 39 | 0.55 (0.39, 0.71) | 0.9913 | 1.8122 |
| ｐ253 | 41 | 0.49 (0.24, 0.74) | 39 | 0.94 (0.68, 1.2) | 0.9425 | 1.8089 |
| ｐ14726 | 42 | 0.21 (0.11, 0.32) | 39 | 0.03 (-0.08, 0.14) | -3.0565 | 1.8057 |
| ｐ8602 | 42 | 0.9 (0.59, 1.22) | 39 | 0.35 (0.02, 0.67) | -1.3886 | 1.8043 |
| ｐ22786 | 42 | 0.35 (0.25, 0.45) | 39 | 0.53 (0.42, 0.64) | 0.6074 | 1.7976 |
| ｐ21302 | 42 | 0.72 (0.65, 0.8) | 39 | 0.86 (0.78, 0.94) | 0.2515 | 1.7969 |
| ｐ17552 | 42 | 0.45 (0.37, 0.53) | 39 | 0.59 (0.51, 0.68) | 0.4039 | 1.7959 |
| ｐ244 | 42 | 0.45 (0.37, 0.53) | 39 | 0.59 (0.51, 0.68) | 0.4039 | 1.7948 |
| ｐ13652 | 42 | 0.88 (0.86, 0.9) | 39 | 0.84 (0.82, 0.86) | -0.0672 | 1.7937 |
| ｐ734 | 42 | 0.21 (0.19, 0.24) | 39 | 0.17 (0.14, 0.19) | -0.3642 | 1.7937 |
| ｐ12744 | 42 | 0.55 (0.4, 0.71) | 39 | 0.28 (0.13, 0.44) | -0.9679 | 1.7937 |
| ｐ14422 | 42 | 0.39 (0.35, 0.43) | 39 | 0.32 (0.28, 0.36) | -0.2948 | 1.7937 |
| ｐ12807 | 42 | 0.44 (0.39, 0.49) | 39 | 0.34 (0.29, 0.4) | -0.3529 | 1.7937 |
| ｐ14696 | 42 | 0.73 (0.63, 0.82) | 39 | 0.56 (0.47, 0.66) | -0.3696 | 1.7937 |
| ｐ9980 | 42 | 0.32 (0.27, 0.38) | 39 | 0.22 (0.17, 0.28) | -0.5236 | 1.7937 |
| ｐ1565 | 42 | 0.43 (0.35, 0.52) | 39 | 0.29 (0.2, 0.37) | -0.5999 | 1.7937 |
| ｐ22868 | 42 | 0.33 (0.24, 0.42) | 39 | 0.17 (0.07, 0.26) | -0.9723 | 1.7937 |
| ｐ10365 | 42 | 0.74 (0.59, 0.89) | 39 | 0.47 (0.31, 0.63) | -0.6505 | 1.7937 |
| ｐ18252 | 42 | 0.92 (0.91, 0.93) | 39 | 0.9 (0.89, 0.91) | -0.0345 | 1.7932 |
| ｐ19041 | 42 | 0.68 (0.62, 0.75) | 39 | 0.57 (0.5, 0.64) | -0.2704 | 1.7932 |
| ｐ15806 | 42 | 0.52 (0.43, 0.61) | 39 | 0.36 (0.27, 0.45) | -0.5291 | 1.7932 |
| ｐ15241 | 42 | 0.41 (0.32, 0.51) | 39 | 0.24 (0.14, 0.34) | -0.7775 | 1.7932 |
| ｐ18229 | 42 | 0.17 (0.12, 0.22) | 39 | 0.09 (0.04, 0.14) | -0.9618 | 1.7932 |
| ｐ19327 | 42 | 0.51 (0.35, 0.66) | 39 | 0.23 (0.07, 0.39) | -1.1368 | 1.7932 |
| ｐ17320 | 42 | 0.16 (0.11, 0.21) | 39 | 0.07 (0.02, 0.12) | -1.1478 | 1.7932 |
| ｐ18276 | 42 | 0.35 (0.21, 0.48) | 39 | 0.11 (-0.03, 0.25) | -1.7103 | 1.7932 |
| ｐ15521 | 42 | 0.34 (0.19, 0.49) | 39 | 0.6 (0.45, 0.76) | 0.8311 | 1.7825 |
| ｐ2419 | 42 | 0.75 (0.65, 0.85) | 39 | 0.57 (0.46, 0.67) | -0.4027 | 1.7811 |
| ｐ13757 | 42 | 0.23 (0.14, 0.33) | 39 | 0.07 (-0.03, 0.16) | -1.8380 | 1.7809 |
| ｐ13467 | 42 | 0.38 (0.27, 0.5) | 39 | 0.18 (0.06, 0.3) | -1.1071 | 1.7797 |
| ｐ18499 | 42 | 0.01 (-0.11, 0.14) | 39 | 0.24 (0.11, 0.37) | 4.1868 | 1.7773 |
| ｐ15503 | 42 | 0.02 (-0.13, 0.17) | 39 | 0.28 (0.13, 0.44) | 4.1662 | 1.7773 |
| ｐ15548 | 42 | 0.03 (-0.2, 0.25) | 39 | 0.42 (0.19, 0.64) | 4.0354 | 1.7773 |
| ｐ15452 | 42 | 0.03 (-0.09, 0.14) | 39 | 0.23 (0.11, 0.35) | 3.0138 | 1.7773 |
| ｐ16716 | 42 | 0.05 (-0.14, 0.24) | 39 | 0.39 (0.19, 0.58) | 2.8785 | 1.7773 |
| ｐ15497 | 42 | 0.05 (-0.02, 0.12) | 39 | 0.18 (0.1, 0.26) | 1.8563 | 1.7773 |
| ｐ16681 | 42 | 0.03 (0, 0.06) | 39 | 0.08 (0.05, 0.11) | 1.3426 | 1.7773 |
| ｐ15472 | 42 | 0.11 (0.02, 0.2) | 39 | 0.27 (0.18, 0.37) | 1.3277 | 1.7773 |
| ｐ15558 | 42 | 0.13 (0.03, 0.24) | 39 | 0.32 (0.21, 0.43) | 1.2674 | 1.7773 |
| ｐ15559 | 42 | 0.14 (0.04, 0.24) | 39 | 0.31 (0.21, 0.41) | 1.1663 | 1.7773 |
| ｐ19660 | 42 | 0.11 (0.04, 0.18) | 39 | 0.24 (0.16, 0.31) | 1.1247 | 1.7773 |
| ｐ15468 | 42 | 0.07 (0.03, 0.1) | 39 | 0.13 (0.09, 0.16) | 0.9519 | 1.7773 |
| ｐ15520 | 42 | 0.4 (0.19, 0.6) | 39 | 0.76 (0.55, 0.98) | 0.9516 | 1.7773 |
| ｐ15474 | 42 | 0.16 (0.09, 0.23) | 39 | 0.29 (0.21, 0.37) | 0.8576 | 1.7773 |
| ｐ17976 | 42 | 0.06 (0.04, 0.09) | 39 | 0.11 (0.08, 0.14) | 0.8047 | 1.7773 |
| ｐ15344 | 42 | 0.11 (0.07, 0.16) | 39 | 0.2 (0.15, 0.25) | 0.7930 | 1.7773 |
| ｐ16727 | 42 | 0.28 (0.16, 0.39) | 39 | 0.48 (0.36, 0.6) | 0.7910 | 1.7773 |
| ｐ15479 | 42 | 0.3 (0.18, 0.42) | 39 | 0.51 (0.38, 0.63) | 0.7812 | 1.7773 |
| ｐ15510 | 42 | 0.31 (0.22, 0.41) | 39 | 0.49 (0.39, 0.58) | 0.6235 | 1.7773 |
| ｐ15535 | 42 | 0.09 (0.06, 0.11) | 39 | 0.13 (0.1, 0.16) | 0.5916 | 1.7773 |
| ｐ15529 | 42 | 0.29 (0.23, 0.36) | 39 | 0.41 (0.34, 0.49) | 0.4962 | 1.7773 |
| ｐ15536 | 42 | 0.32 (0.25, 0.38) | 39 | 0.43 (0.36, 0.5) | 0.4560 | 1.7773 |
| ｐ15482 | 42 | 0.21 (0.16, 0.25) | 39 | 0.28 (0.24, 0.33) | 0.4456 | 1.7773 |
| ｐ15378 | 42 | 0.17 (0.14, 0.2) | 39 | 0.23 (0.19, 0.27) | 0.4361 | 1.7773 |
| ｐ15504 | 42 | 0.37 (0.3, 0.45) | 39 | 0.5 (0.43, 0.58) | 0.4356 | 1.7773 |
| ｐ15560 | 42 | 0.38 (0.31, 0.45) | 39 | 0.5 (0.43, 0.57) | 0.4052 | 1.7773 |
| ｐ19645 | 42 | 0.38 (0.32, 0.44) | 39 | 0.49 (0.43, 0.56) | 0.3688 | 1.7773 |
| ｐ15424 | 42 | 0.43 (0.36, 0.5) | 39 | 0.55 (0.48, 0.62) | 0.3537 | 1.7773 |
| ｐ15585 | 42 | 0.52 (0.44, 0.59) | 39 | 0.66 (0.57, 0.74) | 0.3456 | 1.7773 |
| ｐ15377 | 42 | 0.26 (0.22, 0.29) | 39 | 0.32 (0.28, 0.35) | 0.3121 | 1.7773 |
| ｐ15364 | 42 | 0.42 (0.36, 0.47) | 39 | 0.52 (0.46, 0.58) | 0.3100 | 1.7773 |
| ｐ15584 | 42 | 0.43 (0.38, 0.49) | 39 | 0.53 (0.47, 0.59) | 0.2980 | 1.7773 |
| ｐ15463 | 42 | 0.2 (0.18, 0.23) | 39 | 0.25 (0.22, 0.28) | 0.2959 | 1.7773 |
| ｐ15496 | 42 | 0.49 (0.43, 0.55) | 39 | 0.59 (0.53, 0.65) | 0.2726 | 1.7773 |
| ｐ18441 | 42 | 0.41 (0.37, 0.46) | 39 | 0.5 (0.45, 0.55) | 0.2669 | 1.7773 |
| ｐ15538 | 42 | 0.43 (0.38, 0.48) | 39 | 0.51 (0.46, 0.56) | 0.2628 | 1.7773 |
| ｐ15494 | 42 | 0.82 (0.73, 0.91) | 39 | 0.97 (0.88, 1.07) | 0.2495 | 1.7773 |
| ｐ15419 | 42 | 0.47 (0.42, 0.52) | 39 | 0.56 (0.51, 0.61) | 0.2441 | 1.7773 |
| ｐ15498 | 42 | 0.42 (0.37, 0.46) | 39 | 0.49 (0.45, 0.54) | 0.2431 | 1.7773 |
| ｐ15450 | 42 | 0.56 (0.5, 0.61) | 39 | 0.66 (0.6, 0.72) | 0.2366 | 1.7773 |
| ｐ15578 | 42 | 0.62 (0.56, 0.68) | 39 | 0.73 (0.66, 0.79) | 0.2303 | 1.7773 |
| ｐ15586 | 42 | 0.91 (0.83, 1) | 39 | 1.07 (0.98, 1.15) | 0.2230 | 1.7773 |
| ｐ15574 | 42 | 0.75 (0.68, 0.82) | 39 | 0.87 (0.8, 0.94) | 0.2212 | 1.7773 |
| ｐ15522 | 42 | 0.84 (0.77, 0.92) | 39 | 0.98 (0.9, 1.06) | 0.2197 | 1.7773 |
| ｐ15491 | 42 | 0.51 (0.47, 0.56) | 39 | 0.6 (0.55, 0.65) | 0.2195 | 1.7773 |
| ｐ15502 | 42 | 0.31 (0.28, 0.34) | 39 | 0.36 (0.33, 0.39) | 0.2157 | 1.7773 |
| ｐ15462 | 42 | 0.58 (0.52, 0.63) | 39 | 0.67 (0.62, 0.73) | 0.2154 | 1.7773 |
| ｐ19235 | 42 | 0.59 (0.53, 0.64) | 39 | 0.68 (0.62, 0.73) | 0.2128 | 1.7773 |
| ｐ16709 | 42 | 1.72 (1.56, 1.87) | 39 | 1.99 (1.83, 2.15) | 0.2121 | 1.7773 |
| ｐ15495 | 42 | 0.45 (0.41, 0.49) | 39 | 0.52 (0.48, 0.56) | 0.2120 | 1.7773 |
| ｐ15565 | 42 | 0.52 (0.47, 0.56) | 39 | 0.6 (0.55, 0.65) | 0.2103 | 1.7773 |
| ｐ15526 | 42 | 0.26 (0.24, 0.28) | 39 | 0.3 (0.28, 0.32) | 0.2059 | 1.7773 |
| ｐ15533 | 42 | 0.47 (0.44, 0.51) | 39 | 0.54 (0.5, 0.58) | 0.1969 | 1.7773 |
| ｐ19207 | 42 | 1.51 (1.39, 1.64) | 39 | 1.73 (1.6, 1.86) | 0.1968 | 1.7773 |
| ｐ15464 | 42 | 0.24 (0.22, 0.26) | 39 | 0.28 (0.26, 0.3) | 0.1944 | 1.7773 |
| ｐ15577 | 42 | 0.64 (0.6, 0.69) | 39 | 0.73 (0.68, 0.78) | 0.1767 | 1.7773 |
| ｐ15493 | 42 | 0.86 (0.8, 0.93) | 39 | 0.97 (0.91, 1.04) | 0.1762 | 1.7773 |
| ｐ15580 | 42 | 0.46 (0.42, 0.49) | 39 | 0.51 (0.48, 0.55) | 0.1687 | 1.7773 |
| ｐ15525 | 42 | 0.29 (0.27, 0.31) | 39 | 0.33 (0.31, 0.35) | 0.1614 | 1.7773 |
| ｐ15537 | 42 | 0.51 (0.48, 0.55) | 39 | 0.57 (0.54, 0.6) | 0.1543 | 1.7773 |
| ｐ15539 | 42 | 0.39 (0.37, 0.42) | 39 | 0.44 (0.41, 0.46) | 0.1509 | 1.7773 |
| ｐ15550 | 42 | 0.39 (0.37, 0.41) | 39 | 0.43 (0.41, 0.45) | 0.1408 | 1.7773 |
| ｐ19887 | 42 | 0.47 (0.44, 0.5) | 39 | 0.52 (0.49, 0.55) | 0.1372 | 1.7773 |
| ｐ15457 | 42 | 0.45 (0.42, 0.47) | 39 | 0.49 (0.47, 0.52) | 0.1347 | 1.7773 |
| ｐ15465 | 42 | 0.69 (0.65, 0.73) | 39 | 0.76 (0.72, 0.79) | 0.1297 | 1.7773 |
| ｐ15505 | 42 | 0.67 (0.63, 0.7) | 39 | 0.73 (0.69, 0.76) | 0.1245 | 1.7773 |
| ｐ15530 | 42 | 0.73 (0.69, 0.76) | 39 | 0.79 (0.75, 0.83) | 0.1180 | 1.7773 |
| ｐ15566 | 42 | 0.79 (0.75, 0.83) | 39 | 0.85 (0.82, 0.89) | 0.1142 | 1.7773 |
| ｐ15571 | 42 | 0.66 (0.63, 0.69) | 39 | 0.71 (0.68, 0.74) | 0.1117 | 1.7773 |
| ｐ15561 | 42 | 0.97 (0.93, 1.01) | 39 | 1.04 (1, 1.09) | 0.1055 | 1.7773 |
| ｐ15551 | 42 | 1.1 (1.05, 1.14) | 39 | 1.18 (1.13, 1.23) | 0.1045 | 1.7773 |
| ｐ16238 | 42 | 0.5 (0.48, 0.53) | 39 | 0.54 (0.52, 0.56) | 0.1035 | 1.7773 |
| ｐ15459 | 42 | 0.87 (0.84, 0.91) | 39 | 0.94 (0.9, 0.97) | 0.1007 | 1.7773 |
| ｐ18477 | 42 | 0.67 (0.65, 0.7) | 39 | 0.72 (0.69, 0.75) | 0.0995 | 1.7773 |
| ｐ15379 | 42 | 0.61 (0.59, 0.64) | 39 | 0.65 (0.63, 0.68) | 0.0943 | 1.7773 |
| ｐ16884 | 42 | 0.75 (0.72, 0.78) | 39 | 0.8 (0.77, 0.83) | 0.0936 | 1.7773 |
| ｐ15506 | 42 | 0.77 (0.74, 0.8) | 39 | 0.82 (0.79, 0.85) | 0.0917 | 1.7773 |
| ｐ15544 | 42 | 0.84 (0.81, 0.87) | 39 | 0.9 (0.86, 0.93) | 0.0904 | 1.7773 |
| ｐ15426 | 42 | 0.59 (0.57, 0.61) | 39 | 0.63 (0.61, 0.65) | 0.0895 | 1.7773 |
| ｐ15513 | 42 | 0.55 (0.53, 0.57) | 39 | 0.58 (0.56, 0.6) | 0.0883 | 1.7773 |
| ｐ18030 | 42 | 0.57 (0.55, 0.59) | 39 | 0.61 (0.59, 0.63) | 0.0827 | 1.7773 |
| ｐ15473 | 42 | 0.81 (0.78, 0.84) | 39 | 0.86 (0.83, 0.88) | 0.0802 | 1.7773 |
| ｐ15583 | 42 | 1.08 (1.04, 1.11) | 39 | 1.14 (1.1, 1.17) | 0.0765 | 1.7773 |
| ｐ15507 | 42 | 0.65 (0.63, 0.67) | 39 | 0.68 (0.66, 0.7) | 0.0687 | 1.7773 |
| ｐ15460 | 42 | 0.53 (0.51, 0.54) | 39 | 0.55 (0.54, 0.57) | 0.0632 | 1.7773 |
| ｐ15486 | 42 | 0.73 (0.72, 0.75) | 39 | 0.76 (0.75, 0.78) | 0.0528 | 1.7773 |
| ｐ16339 | 42 | 0.62 (0.61, 0.64) | 39 | 0.65 (0.63, 0.66) | 0.0514 | 1.7773 |
| ｐ16927 | 42 | 0.62 (0.61, 0.64) | 39 | 0.65 (0.63, 0.66) | 0.0514 | 1.7773 |
| ｐ396 | 42 | 0.96 (0.94, 0.97) | 39 | 0.98 (0.97, 0.99) | 0.0365 | 1.7766 |
| ｐ790 | 42 | 0.83 (0.83, 0.84) | 39 | 0.85 (0.84, 0.85) | 0.0211 | 1.7766 |
| ｐ3076 | 42 | 0.57 (0.55, 0.59) | 39 | 0.61 (0.59, 0.63) | 0.0975 | 1.7766 |
| ｐ1749 | 42 | 0.71 (0.63, 0.79) | 39 | 0.84 (0.76, 0.92) | 0.2514 | 1.7766 |
| ｐ3539 | 42 | 0.65 (0.6, 0.71) | 39 | 0.75 (0.7, 0.81) | 0.2040 | 1.7766 |
| ｐ21445 | 42 | 0.26 (0.24, 0.28) | 39 | 0.29 (0.27, 0.31) | 0.1751 | 1.7766 |
| ｐ20783 | 42 | 1.22 (1.07, 1.36) | 39 | 1.47 (1.32, 1.62) | 0.2746 | 1.7766 |
| ｐ22403 | 42 | 0.02 (-0.25, 0.28) | 39 | 0.48 (0.21, 0.75) | 4.8810 | 1.7766 |
| ｐ13531 | 42 | 0.43 (0.36, 0.5) | 39 | 0.55 (0.48, 0.62) | 0.3537 | 1.7766 |
| ｐ20297 | 42 | 0.43 (0.36, 0.5) | 39 | 0.55 (0.48, 0.62) | 0.3537 | 1.7766 |
| ｐ22440 | 42 | 0.06 (-0.03, 0.16) | 39 | 0.23 (0.13, 0.33) | 1.9209 | 1.7766 |
| ｐ21655 | 42 | 0.25 (0.13, 0.38) | 39 | 0.47 (0.34, 0.6) | 0.8949 | 1.7766 |
| ｐ22418 | 42 | 0.11 (0.06, 0.16) | 39 | 0.2 (0.15, 0.26) | 0.8558 | 1.7766 |
| ｐ22448 | 42 | 0.45 (0.38, 0.53) | 39 | 0.59 (0.51, 0.67) | 0.3721 | 1.7766 |
| ｐ14401 | 42 | 0.39 (0.34, 0.44) | 39 | 0.48 (0.43, 0.53) | 0.2809 | 1.7766 |
| ｐ1733 | 42 | 0.75 (0.72, 0.78) | 39 | 0.8 (0.77, 0.83) | 0.0936 | 1.7766 |
| ｐ22423 | 42 | 0.01 (-0.08, 0.1) | 39 | 0.17 (0.08, 0.26) | 3.7837 | 1.7766 |
| ｐ22437 | 42 | 0.04 (-0.05, 0.14) | 39 | 0.22 (0.11, 0.32) | 2.3325 | 1.7766 |
| ｐ943 | 42 | 0.22 (0.15, 0.29) | 39 | 0.35 (0.27, 0.42) | 0.6383 | 1.7766 |
| ｐ22402 | 42 | 0.76 (0.69, 0.82) | 39 | 0.87 (0.8, 0.94) | 0.2112 | 1.7766 |
| ｐ21476 | 42 | 0.57 (0.55, 0.59) | 39 | 0.61 (0.59, 0.63) | 0.0975 | 1.7766 |
| ｐ13056 | 42 | 0.61 (0.59, 0.63) | 39 | 0.64 (0.62, 0.67) | 0.0830 | 1.7766 |
| ｐ21144 | 42 | 0.88 (0.84, 0.93) | 39 | 0.97 (0.92, 1.02) | 0.1296 | 1.7766 |
| ｐ21437 | 42 | 0.8 (0.77, 0.84) | 39 | 0.87 (0.83, 0.9) | 0.1040 | 1.7766 |
| ｐ22406 | 42 | 0.2 (0.03, 0.36) | 39 | 0.49 (0.32, 0.66) | 1.3112 | 1.7766 |
| ｐ14432 | 42 | 0.22 (0.14, 0.31) | 39 | 0.38 (0.29, 0.46) | 0.7539 | 1.7766 |
| ｐ818 | 42 | 0.38 (0.32, 0.44) | 39 | 0.49 (0.43, 0.56) | 0.3688 | 1.7766 |
| ｐ1239 | 42 | 0.81 (0.77, 0.86) | 39 | 0.89 (0.85, 0.94) | 0.1353 | 1.7766 |
| ｐ22431 | 42 | 0.03 (-0.03, 0.1) | 39 | 0.15 (0.08, 0.21) | 2.0688 | 1.7766 |
| ｐ22446 | 42 | 0.14 (-0.08, 0.36) | 39 | 0.53 (0.3, 0.76) | 1.9283 | 1.7766 |
| ｐ1179 | 42 | 0.21 (0.05, 0.37) | 39 | 0.5 (0.33, 0.67) | 1.2386 | 1.7766 |
| ｐ3538 | 42 | 0.65 (0.43, 0.86) | 39 | 1.03 (0.81, 1.25) | 0.6645 | 1.7766 |
| ｐ20222 | 42 | 0.7 (0.61, 0.8) | 39 | 0.87 (0.77, 0.97) | 0.3092 | 1.7766 |
| ｐ22434 | 42 | 0.55 (0.48, 0.61) | 39 | 0.67 (0.6, 0.74) | 0.2864 | 1.7766 |
| ｐ22429 | 42 | 0.63 (0.57, 0.69) | 39 | 0.74 (0.67, 0.8) | 0.2250 | 1.7766 |
| ｐ7602 | 42 | 0.72 (0.69, 0.76) | 39 | 0.79 (0.75, 0.83) | 0.1263 | 1.7766 |
| ｐ22424 | 42 | 0.02 (-0.11, 0.14) | 39 | 0.24 (0.11, 0.37) | 3.8884 | 1.7766 |
| ｐ22416 | 42 | 0.3 (0.12, 0.48) | 39 | 0.62 (0.43, 0.81) | 1.0521 | 1.7766 |
| ｐ22408 | 42 | 0.27 (0.18, 0.36) | 39 | 0.43 (0.33, 0.52) | 0.6815 | 1.7766 |
| ｐ20368 | 42 | 0.63 (0.58, 0.67) | 39 | 0.71 (0.66, 0.76) | 0.1797 | 1.7766 |
| ｐ21110 | 42 | 0.13 (0.12, 0.14) | 39 | 0.14 (0.13, 0.15) | 0.1647 | 1.7766 |
| ｐ22420 | 42 | 0.58 (0.56, 0.6) | 39 | 0.62 (0.6, 0.64) | 0.0802 | 1.7766 |
| ｐ21828 | 42 | 0.39 (0.38, 0.4) | 39 | 0.41 (0.4, 0.42) | 0.0726 | 1.7766 |
| ｐ22417 | 42 | 0.36 (0.3, 0.41) | 39 | 0.45 (0.4, 0.51) | 0.3353 | 1.7766 |
| ｐ20844 | 42 | 0.37 (0.36, 0.38) | 39 | 0.39 (0.38, 0.41) | 0.0821 | 1.7766 |
| ｐ22405 | 42 | 0.1 (-0.07, 0.26) | 39 | 0.39 (0.22, 0.56) | 2.0277 | 1.7766 |
| ｐ20387 | 42 | 0.49 (0.44, 0.54) | 39 | 0.57 (0.52, 0.62) | 0.2238 | 1.7766 |
| ｐ22421 | 42 | 0.15 (-0.01, 0.31) | 39 | 0.44 (0.27, 0.61) | 1.5476 | 1.7766 |
| ｐ13378 | 42 | 0.43 (0.38, 0.48) | 39 | 0.51 (0.46, 0.56) | 0.2530 | 1.7766 |
| ｐ22444 | 42 | 0.47 (0.36, 0.57) | 39 | 0.65 (0.54, 0.75) | 0.4760 | 1.7766 |
| ｐ7853 | 42 | 0.21 (0.18, 0.23) | 39 | 0.25 (0.22, 0.27) | 0.2654 | 1.7766 |
| ｐ12374 | 42 | 0.21 (0.19, 0.24) | 39 | 0.26 (0.23, 0.29) | 0.2933 | 1.7766 |
| ｐ21459 | 42 | 0.43 (0.42, 0.45) | 39 | 0.46 (0.45, 0.48) | 0.0958 | 1.7766 |
| ｐ825 | 42 | 0.41 (0.36, 0.46) | 39 | 0.49 (0.44, 0.54) | 0.2659 | 1.7766 |
| ｐ14494 | 42 | 0.44 (0.32, 0.55) | 39 | 0.23 (0.11, 0.35) | -0.9168 | 1.7765 |
| ｐ19157 | 42 | 0.36 (0.18, 0.54) | 39 | 0.67 (0.49, 0.85) | 0.8969 | 1.7721 |
| ｐ15563 | 42 | 0.77 (0.61, 0.93) | 39 | 1.05 (0.88, 1.22) | 0.4564 | 1.7696 |
| ｐ56 | 40 | 0.83 (0.4, 1.26) | 39 | 0.09 (-0.35, 0.52) | -3.2893 | 1.7655 |
| ｐ10051 | 42 | 0.7 (0.69, 0.72) | 39 | 0.68 (0.66, 0.69) | -0.0486 | 1.7550 |
| ｐ15799 | 42 | 0.77 (0.23, 1.31) | 39 | 1.72 (1.16, 2.28) | 1.1630 | 1.7545 |
| ｐ14106 | 42 | 0.12 (0.01, 0.24) | 39 | 0.32 (0.2, 0.44) | 1.4121 | 1.7515 |
| ｐ15164 | 42 | 0 (-0.25, 0.25) | 39 | 0.44 (0.18, 0.69) | NA | 1.7496 |
| ｐ16849 | 42 | 0.21 (0.11, 0.31) | 39 | 0.38 (0.28, 0.48) | 0.8892 | 1.7496 |
| ｐ2169 | 42 | 0.27 (0.21, 0.33) | 39 | 0.16 (0.09, 0.22) | -0.7582 | 1.7490 |
| ｐ833 | 42 | 1.17 (0.53, 1.81) | 39 | 2.28 (1.62, 2.95) | 0.9669 | 1.7474 |
| ｐ14399 | 42 | 0.53 (0.43, 0.64) | 39 | 0.72 (0.61, 0.84) | 0.4388 | 1.7462 |
| ｐ21482 | 42 | 0.39 (0.19, 0.59) | 39 | 0.74 (0.53, 0.95) | 0.9240 | 1.7435 |
| ｐ22705 | 42 | 0.35 (0.21, 0.49) | 39 | 0.59 (0.45, 0.74) | 0.7771 | 1.7425 |
| ｐ15454 | 42 | 0.65 (0.59, 0.7) | 39 | 0.75 (0.69, 0.81) | 0.2137 | 1.7423 |
| ｐ179 | 41 | 0.49 (0.04, 0.93) | 39 | 1.25 (0.8, 1.71) | 1.3653 | 1.7416 |
| ｐ254 | 42 | 0.84 (0.66, 1.02) | 39 | 1.15 (0.97, 1.33) | 0.4511 | 1.7410 |
| ｐ17051 | 42 | 0.32 (0.24, 0.4) | 39 | 0.18 (0.1, 0.27) | -0.8235 | 1.7399 |
| ｐ9197 | 42 | 0.37 (0.29, 0.45) | 39 | 0.23 (0.14, 0.31) | -0.7000 | 1.7378 |
| ｐ3053 | 42 | 0.23 (0.12, 0.33) | 39 | 0.41 (0.3, 0.52) | 0.8631 | 1.7376 |
| ｐ15564 | 42 | 0.47 (0.32, 0.62) | 39 | 0.73 (0.58, 0.89) | 0.6352 | 1.7375 |
| ｐ1180 | 42 | 3.06 (1.86, 4.26) | 33 | 5.24 (3.89, 6.59) | 0.7784 | 1.7342 |
| ｐ7740 | 42 | 0.45 (0.32, 0.59) | 39 | 0.69 (0.55, 0.83) | 0.6026 | 1.7282 |
| ｐ7206 | 42 | 0.34 (0.21, 0.48) | 39 | 0.58 (0.44, 0.72) | 0.7566 | 1.7240 |
| ｐ13052 | 42 | 0.49 (0.46, 0.52) | 39 | 0.54 (0.51, 0.58) | 0.1563 | 1.7221 |
| ｐ264 | 42 | 1 (0.86, 1.14) | 39 | 0.75 (0.61, 0.9) | -0.4079 | 1.7194 |
| ｐ17181 | 42 | 0.17 (0.07, 0.26) | 39 | 0.32 (0.23, 0.42) | 0.9641 | 1.7167 |
| ｐ22757 | 42 | 0.26 (0.16, 0.35) | 39 | 0.42 (0.32, 0.52) | 0.7203 | 1.7041 |
| ｐ13402 | 42 | 0.33 (0.15, 0.52) | 39 | 0.65 (0.46, 0.84) | 0.9685 | 1.7039 |
| ｐ15312 | 42 | 0.16 (0.08, 0.24) | 39 | 0.3 (0.21, 0.38) | 0.9070 | 1.7033 |
| ｐ22415 | 42 | 0.11 (-0.04, 0.26) | 39 | 0.36 (0.21, 0.52) | 1.7177 | 1.7014 |
| ｐ16412 | 42 | 0.37 (0.24, 0.49) | 39 | 0.59 (0.45, 0.72) | 0.6820 | 1.7011 |
| ｐ15456 | 42 | 0.2 (0.15, 0.25) | 39 | 0.29 (0.23, 0.34) | 0.5135 | 1.6946 |
| ｐ13591 | 41 | 0.92 (-1.09, 2.93) | 39 | 4.35 (2.28, 6.41) | 2.2423 | 1.6931 |
| ｐ22701 | 42 | 0.39 (0.25, 0.53) | 39 | 0.63 (0.49, 0.78) | 0.6902 | 1.6929 |
| ｐ17421 | 42 | 0.17 (0.12, 0.22) | 39 | 0.09 (0.04, 0.14) | -0.9430 | 1.6882 |
| ｐ16491 | 41 | 0.54 (0.39, 0.69) | 39 | 0.8 (0.64, 0.95) | 0.5633 | 1.6861 |
| ｐ19828 | 42 | 0.56 (0.53, 0.59) | 39 | 0.61 (0.58, 0.64) | 0.1155 | 1.6840 |
| ｐ920 | 42 | 0.25 (0.11, 0.39) | 39 | 0.49 (0.35, 0.64) | 0.9830 | 1.6821 |
| ｐ11735 | 42 | 0.58 (0.24, 0.92) | 39 | 1.16 (0.81, 1.51) | 0.9959 | 1.6799 |
| ｐ15899 | 42 | 0.39 (0.24, 0.54) | 39 | 0.65 (0.49, 0.8) | 0.7198 | 1.6799 |
| ｐ16726 | 42 | 0.33 (-0.15, 0.8) | 39 | 1.14 (0.64, 1.63) | 1.7993 | 1.6778 |
| ｐ16733 | 42 | 0.23 (-0.06, 0.53) | 39 | 0.74 (0.43, 1.05) | 1.6676 | 1.6778 |
| ｐ19664 | 42 | 0.71 (0.61, 0.81) | 39 | 0.88 (0.78, 0.99) | 0.3106 | 1.6778 |
| ｐ423 | 42 | 0.73 (0.6, 0.87) | 39 | 0.96 (0.82, 1.1) | 0.3945 | 1.6743 |
| ｐ2393 | 42 | 0.42 (-0.47, 1.3) | 39 | 1.93 (1.01, 2.84) | 2.2005 | 1.6741 |
| ｐ14363 | 42 | 0.5 (0.23, 0.76) | 39 | 0.95 (0.67, 1.23) | 0.9438 | 1.6729 |
| ｐ20439 | 42 | 0.34 (0.02, 0.66) | 39 | 0.88 (0.55, 1.22) | 1.3709 | 1.6719 |
| ｐ20169 | 41 | 0.25 (0.15, 0.34) | 39 | 0.08 (-0.02, 0.18) | -1.6096 | 1.6710 |
| ｐ21767 | 42 | 0.39 (0.34, 0.45) | 39 | 0.3 (0.25, 0.36) | -0.3727 | 1.6696 |
| ｐ22035 | 42 | 0.45 (0.32, 0.57) | 39 | 0.66 (0.53, 0.8) | 0.5761 | 1.6675 |
| ｐ12076 | 42 | 0.33 (0.21, 0.44) | 39 | 0.13 (0, 0.25) | -1.3817 | 1.6493 |
| ｐ16389 | 42 | 0.1 (0.04, 0.16) | 39 | 0.19 (0.14, 0.25) | 0.9689 | 1.6459 |
| ｐ16711 | 42 | 0.81 (0.78, 0.84) | 39 | 0.86 (0.83, 0.88) | 0.0777 | 1.6421 |
| ｐ10333 | 42 | 0.45 (0.3, 0.61) | 39 | 0.72 (0.55, 0.88) | 0.6586 | 1.6375 |
| ｐ21791 | 42 | 0.66 (0.47, 0.85) | 39 | 0.98 (0.78, 1.18) | 0.5731 | 1.6356 |
| ｐ16762 | 42 | 0.28 (-0.35, 0.9) | 39 | 1.32 (0.67, 1.97) | 2.2512 | 1.6308 |
| ｐ11198 | 42 | 0.41 (0.27, 0.55) | 39 | 0.65 (0.5, 0.8) | 0.6592 | 1.6253 |
| ｐ12192 | 42 | 0.51 (0.38, 0.63) | 39 | 0.3 (0.17, 0.43) | -0.7469 | 1.6187 |
| ｐ16472 | 42 | 0.16 (0.1, 0.23) | 39 | 0.27 (0.2, 0.34) | 0.7148 | 1.6144 |
| ｐ13559 | 42 | 0.27 (0.15, 0.38) | 39 | 0.08 (-0.04, 0.2) | -1.7763 | 1.6130 |
| ｐ22828 | 42 | 0 (-0.18, 0.18) | 39 | 0.29 (0.11, 0.48) | NA | 1.6090 |
| ｐ14967 | 42 | 0.41 (0.3, 0.52) | 39 | 0.59 (0.48, 0.7) | 0.5243 | 1.6063 |
| ｐ1237 | 42 | 0.19 (0.1, 0.28) | 39 | 0.34 (0.24, 0.44) | 0.8611 | 1.6048 |
| ｐ12196 | 42 | 0.51 (0.34, 0.67) | 39 | 0.24 (0.07, 0.41) | -1.1061 | 1.6043 |
| ｐ9177 | 42 | 0.66 (0.47, 0.85) | 39 | 0.35 (0.16, 0.54) | -0.9080 | 1.6031 |
| ｐ15725 | 42 | 0.32 (0.23, 0.42) | 39 | 0.17 (0.08, 0.27) | -0.9150 | 1.5986 |
| ｐ10972 | 42 | 0.57 (0.35, 0.79) | 39 | 0.21 (-0.02, 0.44) | -1.4466 | 1.5972 |
| ｐ17782 | 42 | 0.76 (0.25, 1.27) | 39 | 1.6 (1.07, 2.13) | 1.0716 | 1.5918 |
| ｐ21085 | 42 | 0.49 (0.38, 0.6) | 39 | 0.67 (0.55, 0.78) | 0.4586 | 1.5901 |
| ｐ11838 | 42 | 0.6 (0.49, 0.72) | 39 | 0.79 (0.67, 0.91) | 0.3910 | 1.5891 |
| ｐ15438 | 42 | 0.27 (0.16, 0.38) | 39 | 0.45 (0.33, 0.56) | 0.7342 | 1.5884 |
| ｐ9138 | 42 | 0.38 (0.29, 0.46) | 39 | 0.23 (0.14, 0.32) | -0.6822 | 1.5879 |
| ｐ14309 | 42 | 0.49 (0.24, 0.75) | 39 | 0.91 (0.65, 1.18) | 0.8898 | 1.5854 |
| ｐ16035 | 42 | 0.31 (0.22, 0.4) | 39 | 0.46 (0.36, 0.55) | 0.5679 | 1.5817 |
| ｐ13703 | 42 | 0.04 (-0.07, 0.15) | 39 | 0.22 (0.11, 0.34) | 2.4831 | 1.5780 |
| ｐ11217 | 42 | 0.44 (-0.65, 1.52) | 39 | 2.21 (1.08, 3.33) | 2.3355 | 1.5740 |
| ｐ21327 | 42 | 0.57 (0.53, 0.61) | 39 | 0.5 (0.46, 0.54) | -0.1847 | 1.5737 |
| ｐ22157 | 42 | 0.7 (0.66, 0.73) | 39 | 0.64 (0.6, 0.67) | -0.1305 | 1.5737 |
| ｐ20758 | 42 | 0.51 (0.46, 0.57) | 39 | 0.42 (0.36, 0.48) | -0.2887 | 1.5737 |
| ｐ20686 | 42 | 0.32 (0.28, 0.36) | 39 | 0.25 (0.2, 0.29) | -0.3612 | 1.5737 |
| ｐ13234 | 42 | 0.34 (0.29, 0.38) | 39 | 0.26 (0.21, 0.31) | -0.3830 | 1.5737 |
| ｐ10223 | 42 | 0.45 (0.37, 0.52) | 39 | 0.33 (0.25, 0.4) | -0.4572 | 1.5737 |
| ｐ14721 | 42 | 0.25 (0.22, 0.27) | 39 | 0.21 (0.19, 0.23) | -0.2317 | 1.5737 |
| ｐ17123 | 42 | 0.69 (0.68, 0.7) | 39 | 0.67 (0.66, 0.69) | -0.0420 | 1.5735 |
| ｐ16929 | 42 | 0.5 (0.48, 0.52) | 39 | 0.46 (0.43, 0.48) | -0.1204 | 1.5735 |
| ｐ16604 | 42 | 0.44 (0.41, 0.46) | 39 | 0.4 (0.37, 0.42) | -0.1381 | 1.5735 |
| ｐ15690 | 42 | 0.3 (0.28, 0.32) | 39 | 0.27 (0.24, 0.29) | -0.1784 | 1.5735 |
| ｐ16542 | 42 | 0.51 (0.47, 0.54) | 39 | 0.45 (0.41, 0.48) | -0.1842 | 1.5735 |
| ｐ19500 | 42 | 0.46 (0.42, 0.5) | 39 | 0.4 (0.36, 0.44) | -0.2040 | 1.5735 |
| ｐ19325 | 42 | 0.36 (0.32, 0.39) | 39 | 0.3 (0.26, 0.34) | -0.2552 | 1.5735 |
| ｐ15243 | 42 | 0.64 (0.55, 0.72) | 39 | 0.49 (0.41, 0.58) | -0.3614 | 1.5735 |
| ｐ17866 | 42 | 0.7 (0.61, 0.8) | 39 | 0.55 (0.45, 0.65) | -0.3635 | 1.5735 |
| ｐ17093 | 42 | 0.48 (0.39, 0.58) | 39 | 0.33 (0.23, 0.43) | -0.5657 | 1.5735 |
| ｐ15338 | 42 | 0.35 (0.24, 0.45) | 39 | 0.18 (0.07, 0.29) | -0.9527 | 1.5735 |
| ｐ15188 | 42 | 0.3 (0.2, 0.41) | 39 | 0.14 (0.03, 0.24) | -1.1612 | 1.5735 |
| ｐ17740 | 42 | 0.16 (0.1, 0.21) | 39 | 0.07 (0.01, 0.13) | -1.1790 | 1.5735 |
| ｐ19835 | 42 | 0.36 (0.22, 0.5) | 39 | 0.13 (-0.02, 0.27) | -1.5040 | 1.5735 |
| ｐ16844 | 42 | 0.26 (0.11, 0.41) | 39 | 0.02 (-0.14, 0.17) | -4.1054 | 1.5735 |
| ｐ22409 | 42 | 0.2 (0.14, 0.26) | 39 | 0.3 (0.24, 0.37) | 0.6038 | 1.5707 |
| ｐ10510 | 42 | 0.43 (-0.07, 0.92) | 39 | 1.23 (0.72, 1.75) | 1.5355 | 1.5701 |
| ｐ15490 | 42 | 0.14 (0, 0.28) | 39 | 0.37 (0.22, 0.52) | 1.4273 | 1.5686 |
| ｐ10272 | 42 | 0.65 (0.54, 0.76) | 39 | 0.47 (0.36, 0.59) | -0.4567 | 1.5681 |
| ｐ3059 | 42 | 0.27 (0.18, 0.37) | 39 | 0.42 (0.33, 0.52) | 0.6298 | 1.5665 |
| ｐ22436 | 42 | 0.23 (0.17, 0.29) | 39 | 0.33 (0.27, 0.4) | 0.5293 | 1.5645 |
| ｐ17677 | 42 | 0.17 (0.09, 0.24) | 39 | 0.04 (-0.04, 0.12) | -2.0113 | 1.5638 |
| ｐ14829 | 42 | 0.47 (0.33, 0.61) | 39 | 0.25 (0.1, 0.39) | -0.9320 | 1.5627 |
| ｐ14183 | 42 | 0.21 (0.12, 0.31) | 39 | 0.37 (0.27, 0.47) | 0.7994 | 1.5604 |
| ｐ9578 | 42 | 0.46 (-0.06, 0.98) | 39 | 1.31 (0.77, 1.85) | 1.5045 | 1.5601 |
| ｐ10689 | 42 | 0.56 (0.3, 0.82) | 39 | 0.13 (-0.14, 0.41) | -2.0760 | 1.5596 |
| ｐ12407 | 42 | 0.56 (0.3, 0.82) | 39 | 0.13 (-0.14, 0.41) | -2.0760 | 1.5596 |
| ｐ15341 | 42 | 0.29 (0.21, 0.37) | 39 | 0.42 (0.34, 0.5) | 0.5338 | 1.5575 |
| ｐ21670 | 42 | 0.16 (0.06, 0.26) | 39 | 0 (-0.1, 0.1) | NA | 1.5550 |
| ｐ14871 | 42 | 0.73 (0.45, 1.01) | 39 | 0.28 (-0.01, 0.57) | -1.4022 | 1.5522 |
| ｐ19723 | 42 | 0.26 (-0.14, 0.66) | 39 | 0.91 (0.49, 1.33) | 1.8071 | 1.5436 |
| ｐ22368 | 42 | 0.43 (0.33, 0.53) | 39 | 0.26 (0.16, 0.37) | -0.6998 | 1.5366 |
| ｐ14745 | 42 | 0.81 (0.56, 1.05) | 39 | 1.2 (0.95, 1.46) | 0.5750 | 1.5313 |
| ｐ12129 | 42 | 0.12 (0, 0.24) | 39 | 0.31 (0.19, 0.43) | 1.3742 | 1.5273 |
| ｐ22447 | 42 | 0.11 (0.01, 0.2) | 39 | 0.25 (0.16, 0.35) | 1.2511 | 1.5245 |
| ｐ15500 | 42 | 0.51 (0.24, 0.79) | 39 | 0.95 (0.67, 1.24) | 0.8886 | 1.5157 |
| ｐ22435 | 42 | 0.3 (0.17, 0.42) | 39 | 0.5 (0.37, 0.62) | 0.7348 | 1.5151 |
| ｐ12353 | 42 | 0.07 (-0.05, 0.19) | 39 | 0.26 (0.13, 0.38) | 1.9363 | 1.5148 |
| ｐ20619 | 42 | 0.27 (0.15, 0.39) | 39 | 0.47 (0.34, 0.59) | 0.7873 | 1.5147 |
| ｐ22438 | 42 | 0.15 (0.06, 0.23) | 39 | 0.29 (0.19, 0.38) | 0.9742 | 1.5112 |
| ｐ6075 | 42 | 0.75 (0.67, 0.83) | 39 | 0.63 (0.55, 0.71) | -0.2589 | 1.5110 |
| ｐ21632 | 42 | 0.32 (0.26, 0.38) | 39 | 0.23 (0.17, 0.29) | -0.5031 | 1.5098 |
| ｐ20975 | 42 | 0.13 (-0.1, 0.35) | 39 | 0.49 (0.25, 0.72) | 1.9316 | 1.5089 |
| ｐ15517 | 42 | 0.3 (0.17, 0.44) | 39 | 0.51 (0.38, 0.65) | 0.7509 | 1.5072 |
| ｐ14226 | 42 | 1.32 (0.9, 1.75) | 39 | 2 (1.56, 2.45) | 0.5979 | 1.5070 |
| ｐ14724 | 42 | 1 (0.57, 1.44) | 39 | 1.7 (1.24, 2.15) | 0.7586 | 1.5062 |
| ｐ20019 | 42 | 0.24 (0.14, 0.33) | 39 | 0.39 (0.29, 0.49) | 0.7117 | 1.5054 |
| ｐ4843 | 42 | 0.24 (0.14, 0.33) | 39 | 0.39 (0.29, 0.49) | 0.7117 | 1.5054 |
| ｐ22030 | 42 | 0.61 (0.46, 0.76) | 39 | 0.85 (0.7, 1.01) | 0.4827 | 1.5049 |
| ｐ15567 | 42 | 0.42 (0.33, 0.5) | 39 | 0.55 (0.46, 0.63) | 0.3993 | 1.5045 |
| ｐ147 | 40 | 0.33 (0.07, 0.58) | 39 | 0.73 (0.47, 0.99) | 1.1559 | 1.5025 |
| ｐ13485 | 42 | 0.43 (-0.94, 1.8) | 39 | 2.6 (1.18, 4.02) | 2.5972 | 1.5021 |
| ｐ18810 | 42 | 0 (-0.15, 0.15) | 39 | 0.24 (0.08, 0.4) | NA | 1.5017 |
| ｐ15518 | 42 | 0.33 (0.26, 0.4) | 39 | 0.44 (0.37, 0.52) | 0.4223 | 1.5017 |
| ｐ12408 | 42 | 0.34 (0.17, 0.51) | 39 | 0.62 (0.44, 0.8) | 0.8528 | 1.5013 |
| ｐ11384 | 42 | 0.09 (-0.02, 0.19) | 39 | 0.26 (0.15, 0.37) | 1.5406 | 1.4967 |
| ｐ22425 | 42 | 0.58 (0.29, 0.88) | 39 | 1.05 (0.75, 1.36) | 0.8498 | 1.4967 |
| ｐ13829 | 42 | 0.22 (0.13, 0.31) | 39 | 0.08 (-0.01, 0.17) | -1.4678 | 1.4957 |
| ｐ12377 | 42 | 0.89 (0.75, 1.03) | 39 | 0.67 (0.53, 0.82) | -0.4099 | 1.4956 |
| ｐ10640 | 41 | 1.74 (0.96, 2.51) | 39 | 2.95 (2.16, 3.75) | 0.7669 | 1.4955 |
| ｐ22699 | 42 | 0.29 (0.11, 0.46) | 39 | 0.56 (0.38, 0.74) | 0.9676 | 1.4918 |
| ｐ22441 | 42 | 0 (-0.15, 0.15) | 39 | 0.24 (0.08, 0.39) | NA | 1.4869 |
| ｐ15557 | 42 | 0.46 (0.22, 0.7) | 39 | 0.84 (0.59, 1.09) | 0.8564 | 1.4841 |
| ｐ22413 | 42 | 0 (-0.37, 0.37) | 39 | 0.58 (0.2, 0.96) | NA | 1.4826 |
| ｐ15532 | 42 | 0.15 (0.05, 0.26) | 39 | 0.31 (0.21, 0.42) | 1.0286 | 1.4802 |
| ｐ16764 | 42 | 0.52 (0.28, 0.75) | 39 | 0.89 (0.64, 1.13) | 0.7725 | 1.4789 |
| ｐ22442 | 42 | 0.06 (0.02, 0.09) | 39 | 0.11 (0.07, 0.15) | 1.0302 | 1.4777 |
| ｐ17451 | 42 | 0.55 (0.51, 0.59) | 39 | 0.49 (0.45, 0.53) | -0.1704 | 1.4750 |
| ｐ13671 | 42 | 0.66 (0.42, 0.89) | 39 | 1.03 (0.78, 1.27) | 0.6418 | 1.4725 |
| ｐ16504 | 42 | 0.66 (0.42, 0.89) | 39 | 1.03 (0.78, 1.27) | 0.6418 | 1.4724 |
| ｐ13861 | 42 | 0.11 (0.01, 0.21) | 39 | 0.26 (0.16, 0.37) | 1.3018 | 1.4722 |
| ｐ21749 | 42 | 0.63 (0.03, 1.23) | 39 | 1.57 (0.95, 2.2) | 1.3209 | 1.4687 |
| ｐ15093 | 42 | 0.63 (0.03, 1.23) | 39 | 1.57 (0.95, 2.2) | 1.3209 | 1.4685 |
| ｐ15516 | 42 | 0.59 (0.56, 0.61) | 39 | 0.62 (0.6, 0.65) | 0.0874 | 1.4685 |
| ｐ15553 | 42 | 0.4 (0.28, 0.52) | 39 | 0.59 (0.46, 0.71) | 0.5629 | 1.4672 |
| ｐ17126 | 42 | 0.12 (0.02, 0.23) | 39 | 0.29 (0.18, 0.4) | 1.2251 | 1.4622 |
| ｐ14915 | 42 | 0.03 (0.02, 0.05) | 39 | 0.06 (0.04, 0.07) | 0.8240 | 1.4591 |
| ｐ10747 | 42 | 0.87 (0.49, 1.25) | 39 | 0.28 (-0.12, 0.67) | -1.6556 | 1.4563 |
| ｐ220 | 42 | 0.79 (0.51, 1.06) | 39 | 1.22 (0.93, 1.5) | 0.6299 | 1.4556 |
| ｐ14218 | 42 | 0.3 (0.17, 0.43) | 39 | 0.51 (0.37, 0.64) | 0.7546 | 1.4549 |
| ｐ313 | 42 | 0.47 (0.38, 0.57) | 39 | 0.33 (0.23, 0.43) | -0.5263 | 1.4512 |
| ｐ15146 | 42 | 0.28 (0.1, 0.47) | 39 | 0.57 (0.38, 0.76) | 1.0053 | 1.4498 |
| ｐ20700 | 42 | 0.35 (0.27, 0.44) | 39 | 0.23 (0.14, 0.31) | -0.6356 | 1.4492 |
| ｐ22058 | 42 | 0.08 (-0.06, 0.21) | 39 | 0.29 (0.15, 0.43) | 1.9206 | 1.4470 |
| ｐ4813 | 42 | 0.25 (0.21, 0.28) | 39 | 0.2 (0.16, 0.23) | -0.3317 | 1.4466 |
| ｐ15614 | 42 | 0.49 (0.37, 0.61) | 39 | 0.3 (0.17, 0.43) | -0.7004 | 1.4461 |
| ｐ15575 | 42 | 0.16 (0.08, 0.25) | 39 | 0.29 (0.2, 0.38) | 0.8546 | 1.4377 |
| ｐ22118 | 42 | 0.16 (0.05, 0.26) | 39 | 0 (-0.11, 0.11) | NA | 1.4349 |
| ｐ47 | 42 | 0.33 (0.04, 0.62) | 39 | 0.78 (0.48, 1.08) | 1.2425 | 1.4284 |
| ｐ22072 | 42 | 0.77 (0.71, 0.82) | 39 | 0.85 (0.79, 0.9) | 0.1445 | 1.4284 |
| ｐ14752 | 42 | 0.7 (0.52, 0.88) | 39 | 0.43 (0.25, 0.61) | -0.7073 | 1.4283 |
| ｐ225 | 42 | 0.55 (0.39, 0.7) | 39 | 0.79 (0.62, 0.95) | 0.5318 | 1.4261 |
| ｐ22509 | 42 | 0.24 (0.16, 0.32) | 39 | 0.12 (0.04, 0.2) | -1.0033 | 1.4214 |
| ｐ17005 | 42 | 0.77 (0.73, 0.81) | 39 | 0.83 (0.79, 0.88) | 0.1155 | 1.4214 |
| ｐ22454 | 42 | 0 (-0.01, 0.01) | 39 | 0.02 (0.01, 0.04) | NA | 1.4158 |
| ｐ4518 | 42 | 0 (-0.07, 0.07) | 39 | 0.11 (0.04, 0.19) | NA | 1.4131 |
| ｐ17366 | 42 | 0.58 (0.53, 0.62) | 39 | 0.5 (0.45, 0.55) | -0.1917 | 1.4123 |
| ｐ17774 | 42 | 0.33 (0.19, 0.47) | 39 | 0.54 (0.4, 0.69) | 0.7218 | 1.4101 |
| ｐ22453 | 42 | 0 (-0.07, 0.07) | 39 | 0.11 (0.03, 0.18) | NA | 1.4098 |
| ｐ6388 | 42 | 0.22 (0.11, 0.34) | 39 | 0.05 (-0.08, 0.17) | -2.3063 | 1.4089 |
| ｐ10287 | 42 | 0.38 (0.2, 0.56) | 39 | 0.1 (-0.08, 0.29) | -1.8670 | 1.4077 |
| ｐ11024 | 42 | 0.29 (-0.58, 1.17) | 39 | 1.62 (0.71, 2.53) | 2.4720 | 1.4036 |
| ｐ18224 | 42 | 0.89 (0.61, 1.16) | 39 | 0.46 (0.18, 0.75) | -0.9301 | 1.4023 |
| ｐ15547 | 42 | 0.1 (0.08, 0.13) | 39 | 0.14 (0.12, 0.17) | 0.4612 | 1.4001 |
| ｐ22445 | 42 | 0 (-0.14, 0.14) | 39 | 0.22 (0.07, 0.37) | NA | 1.3971 |
| ｐ15214 | 42 | 0.51 (0.16, 0.86) | 39 | 1.04 (0.67, 1.4) | 1.0274 | 1.3969 |
| ｐ19094 | 42 | 0.28 (0.17, 0.39) | 39 | 0.44 (0.33, 0.55) | 0.6744 | 1.3925 |
| ｐ22411 | 42 | 0.38 (0.13, 0.64) | 39 | 0.77 (0.5, 1.03) | 0.9999 | 1.3920 |
| ｐ16557 | 42 | 0.33 (0.09, 0.58) | 39 | 0.71 (0.45, 0.96) | 1.0773 | 1.3915 |
| ｐ13355 | 42 | 0.14 (0.1, 0.18) | 39 | 0.19 (0.16, 0.23) | 0.4736 | 1.3895 |
| ｐ21666 | 42 | 0.64 (0.52, 0.76) | 39 | 0.46 (0.33, 0.58) | -0.4892 | 1.3855 |
| ｐ22450 | 42 | 0.05 (0.02, 0.08) | 39 | 0.09 (0.07, 0.12) | 0.8415 | 1.3844 |
| ｐ722 | 41 | 0.31 (0.24, 0.38) | 39 | 0.2 (0.13, 0.27) | -0.6233 | 1.3832 |
| ｐ18249 | 42 | 0.24 (0.15, 0.34) | 39 | 0.1 (0, 0.2) | -1.2867 | 1.3830 |
| ｐ1532 | 42 | 0.17 (0.09, 0.24) | 39 | 0.28 (0.2, 0.35) | 0.7434 | 1.3828 |
| ｐ13855 | 42 | 0.34 (0.28, 0.4) | 39 | 0.25 (0.19, 0.31) | -0.4350 | 1.3768 |
| ｐ19775 | 42 | 0.26 (-0.01, 0.53) | 39 | 0.67 (0.39, 0.95) | 1.3524 | 1.3757 |
| ｐ17002 | 42 | 0.55 (0.43, 0.67) | 39 | 0.37 (0.24, 0.49) | -0.5854 | 1.3747 |
| ｐ4802 | 42 | 0.66 (-0.33, 1.65) | 39 | 2.14 (1.12, 3.17) | 1.6904 | 1.3744 |
| ｐ15266 | 42 | 0.1 (0.03, 0.17) | 39 | 0 (-0.07, 0.07) | NA | 1.3716 |
| ｐ141 | 42 | 0.41 (0.2, 0.62) | 39 | 0.72 (0.5, 0.93) | 0.8096 | 1.3714 |
| ｐ12991 | 42 | 0 (-0.18, 0.18) | 39 | 0.27 (0.08, 0.45) | NA | 1.3709 |
| ｐ20692 | 42 | 0.32 (0.15, 0.5) | 39 | 0.58 (0.4, 0.76) | 0.8547 | 1.3699 |
| ｐ17732 | 42 | 0.17 (0.01, 0.32) | 39 | 0.39 (0.24, 0.55) | 1.2424 | 1.3696 |
| ｐ10790 | 42 | 0.26 (0.15, 0.38) | 39 | 0.43 (0.32, 0.55) | 0.7087 | 1.3673 |
| ｐ6366 | 42 | 0.21 (0.16, 0.26) | 39 | 0.29 (0.23, 0.34) | 0.4371 | 1.3673 |
| ｐ22410 | 42 | 0.11 (-0.2, 0.42) | 39 | 0.56 (0.25, 0.88) | 2.3678 | 1.3659 |
| ｐ15601 | 42 | 0.44 (0.32, 0.57) | 39 | 0.63 (0.5, 0.77) | 0.5117 | 1.3575 |
| ｐ15446 | 42 | 0.47 (0.41, 0.54) | 39 | 0.57 (0.5, 0.63) | 0.2638 | 1.3565 |
| ｐ19138 | 42 | 0.52 (0.51, 0.54) | 39 | 0.51 (0.5, 0.52) | -0.0457 | 1.3565 |
| ｐ17412 | 42 | 0.69 (0.67, 0.71) | 39 | 0.66 (0.64, 0.68) | -0.0653 | 1.3565 |
| ｐ15595 | 42 | 0.83 (0.81, 0.86) | 39 | 0.79 (0.77, 0.82) | -0.0692 | 1.3565 |
| ｐ15320 | 42 | 0.59 (0.57, 0.61) | 39 | 0.56 (0.54, 0.58) | -0.0781 | 1.3565 |
| ｐ19115 | 42 | 1.41 (1.34, 1.47) | 39 | 1.31 (1.24, 1.38) | -0.1009 | 1.3565 |
| ｐ17602 | 42 | 0.72 (0.68, 0.77) | 39 | 0.66 (0.62, 0.7) | -0.1318 | 1.3565 |
| ｐ15794 | 42 | 0.47 (0.43, 0.5) | 39 | 0.42 (0.39, 0.45) | -0.1526 | 1.3565 |
| ｐ16591 | 42 | 0.44 (0.41, 0.48) | 39 | 0.39 (0.35, 0.43) | -0.1902 | 1.3565 |
| ｐ17307 | 42 | 0.34 (0.31, 0.37) | 39 | 0.29 (0.26, 0.33) | -0.2164 | 1.3565 |
| ｐ15818 | 42 | 0.4 (0.36, 0.45) | 39 | 0.34 (0.29, 0.38) | -0.2606 | 1.3565 |
| ｐ18911 | 42 | 0.72 (0.63, 0.82) | 39 | 0.58 (0.48, 0.68) | -0.3110 | 1.3565 |
| ｐ19633 | 42 | 0.59 (0.51, 0.66) | 39 | 0.47 (0.39, 0.55) | -0.3171 | 1.3565 |
| ｐ16623 | 42 | 0.39 (0.33, 0.45) | 39 | 0.3 (0.24, 0.37) | -0.3652 | 1.3565 |
| ｐ19796 | 42 | 0.2 (0.17, 0.23) | 39 | 0.15 (0.12, 0.19) | -0.3808 | 1.3565 |
| ｐ18279 | 42 | 0.36 (0.3, 0.42) | 39 | 0.28 (0.21, 0.34) | -0.4006 | 1.3565 |
| ｐ18955 | 42 | 0.46 (0.38, 0.54) | 39 | 0.34 (0.26, 0.42) | -0.4177 | 1.3565 |
| ｐ15315 | 42 | 0.69 (0.56, 0.82) | 39 | 0.5 (0.36, 0.63) | -0.4709 | 1.3565 |
| ｐ15280 | 42 | 0.27 (0.21, 0.33) | 39 | 0.19 (0.13, 0.25) | -0.5351 | 1.3565 |
| ｐ19732 | 42 | 0.4 (0.31, 0.49) | 39 | 0.27 (0.18, 0.36) | -0.5724 | 1.3565 |
| ｐ17188 | 42 | 0.25 (0.19, 0.31) | 39 | 0.16 (0.1, 0.22) | -0.6457 | 1.3565 |
| ｐ19075 | 42 | 0.24 (0.17, 0.31) | 39 | 0.13 (0.06, 0.2) | -0.8497 | 1.3565 |
| ｐ15258 | 42 | 0.18 (0.12, 0.24) | 39 | 0.1 (0.03, 0.16) | -0.9494 | 1.3565 |
| ｐ19341 | 42 | 0.68 (0.4, 0.97) | 39 | 0.26 (-0.04, 0.56) | -1.3912 | 1.3565 |
| ｐ14653 | 42 | 1.66 (1.53, 1.79) | 39 | 1.47 (1.33, 1.61) | -0.1784 | 1.3564 |
| ｐ9530 | 42 | 0.46 (0.41, 0.51) | 39 | 0.38 (0.33, 0.43) | -0.2679 | 1.3564 |
| ｐ10405 | 42 | 0.64 (0.61, 0.67) | 39 | 0.6 (0.57, 0.63) | -0.0958 | 1.3564 |
| ｐ14888 | 42 | 0.45 (0.39, 0.51) | 39 | 0.36 (0.3, 0.42) | -0.3244 | 1.3564 |
| ｐ6331 | 42 | 0.35 (0.28, 0.41) | 39 | 0.25 (0.18, 0.32) | -0.4696 | 1.3564 |
| ｐ22621 | 42 | 0.6 (0.54, 0.66) | 39 | 0.52 (0.46, 0.58) | -0.2154 | 1.3564 |
| ｐ9186 | 42 | 0.33 (0.26, 0.41) | 39 | 0.23 (0.16, 0.3) | -0.5407 | 1.3564 |
| ｐ14823 | 42 | 0.54 (0.52, 0.57) | 39 | 0.5 (0.48, 0.53) | -0.1111 | 1.3564 |
| ｐ13809 | 42 | 0.6 (0.56, 0.64) | 39 | 0.54 (0.51, 0.58) | -0.1421 | 1.3564 |
| ｐ11517 | 42 | 0.65 (0.6, 0.7) | 39 | 0.57 (0.52, 0.63) | -0.1814 | 1.3564 |
| ｐ13451 | 42 | 0.44 (0.4, 0.47) | 39 | 0.38 (0.35, 0.42) | -0.1858 | 1.3564 |
| ｐ21145 | 42 | 0.38 (0.33, 0.42) | 39 | 0.32 (0.27, 0.36) | -0.2501 | 1.3564 |
| ｐ14760 | 42 | 0.4 (0.35, 0.44) | 39 | 0.33 (0.28, 0.38) | -0.2639 | 1.3564 |
| ｐ14274 | 42 | 0.5 (0.44, 0.57) | 39 | 0.4 (0.33, 0.47) | -0.3230 | 1.3564 |
| ｐ21754 | 42 | 1.32 (1.12, 1.53) | 39 | 1.02 (0.8, 1.23) | -0.3789 | 1.3564 |
| ｐ12481 | 42 | 0.32 (0.27, 0.37) | 39 | 0.24 (0.19, 0.3) | -0.4000 | 1.3564 |
| ｐ7672 | 42 | 0.23 (0.16, 0.31) | 39 | 0.12 (0.04, 0.2) | -0.9659 | 1.3564 |
| ｐ12694 | 42 | 0.22 (0.08, 0.35) | 39 | 0.02 (-0.12, 0.16) | -3.4554 | 1.3564 |
| ｐ20062 | 42 | 0.29 (0.28, 0.31) | 39 | 0.27 (0.26, 0.29) | -0.1149 | 1.3564 |
| ｐ9502 | 42 | 0.43 (0.39, 0.47) | 39 | 0.37 (0.32, 0.41) | -0.2272 | 1.3564 |
| ｐ20929 | 42 | 0.2 (0.17, 0.23) | 39 | 0.16 (0.13, 0.19) | -0.3339 | 1.3564 |
| ｐ12803 | 42 | 0.23 (0.16, 0.29) | 39 | 0.13 (0.07, 0.2) | -0.7849 | 1.3564 |
| ｐ14043 | 42 | 0.23 (0.16, 0.3) | 39 | 0.13 (0.06, 0.2) | -0.8387 | 1.3564 |
| ｐ8024 | 42 | 0.49 (0.46, 0.53) | 39 | 0.44 (0.4, 0.48) | -0.1724 | 1.3564 |
| ｐ21183 | 42 | 0.33 (0.24, 0.42) | 39 | 0.2 (0.11, 0.29) | -0.7348 | 1.3564 |
| ｐ8173 | 42 | 0.47 (0.43, 0.5) | 39 | 0.42 (0.39, 0.45) | -0.1526 | 1.3564 |
| ｐ22455 | 42 | 0 (-0.07, 0.07) | 39 | 0.1 (0.03, 0.17) | NA | 1.3543 |
| ｐ5372 | 42 | 0.22 (-2.61, 3.06) | 39 | 4.42 (1.48, 7.36) | 4.3171 | 1.3542 |
| ｐ15381 | 42 | 0.48 (0.32, 0.65) | 39 | 0.73 (0.56, 0.9) | 0.5967 | 1.3536 |
| ｐ12303 | 42 | 0.79 (0.71, 0.87) | 39 | 0.91 (0.83, 1) | 0.2043 | 1.3524 |
| ｐ13069 | 42 | 0.37 (0.22, 0.52) | 39 | 0.59 (0.43, 0.74) | 0.6727 | 1.3520 |
| ｐ13324 | 42 | 0.25 (0.18, 0.32) | 39 | 0.36 (0.28, 0.43) | 0.4983 | 1.3510 |
| ｐ851 | 42 | 0.68 (0.63, 0.72) | 39 | 0.75 (0.7, 0.79) | 0.1340 | 1.3508 |
| ｐ16765 | 42 | 0.15 (-0.6, 0.9) | 39 | 1.26 (0.48, 2.04) | 3.0900 | 1.3478 |
| ｐ14425 | 42 | 0.85 (0.57, 1.12) | 39 | 0.44 (0.15, 0.73) | -0.9500 | 1.3466 |
| ｐ15296 | 42 | 0.2 (0.11, 0.29) | 39 | 0.07 (-0.02, 0.16) | -1.4714 | 1.3439 |
| ｐ868 | 42 | 0.78 (0.66, 0.91) | 39 | 0.6 (0.47, 0.73) | -0.3830 | 1.3427 |
| ｐ22933 | 42 | 0 (-0.11, 0.11) | 39 | 0.17 (0.05, 0.28) | 6.1377 | 1.3426 |
| ｐ22427 | 42 | 0 (-0.12, 0.12) | 39 | 0.18 (0.05, 0.31) | NA | 1.3401 |
| ｐ22404 | 42 | 0.27 (0.13, 0.4) | 39 | 0.47 (0.33, 0.61) | 0.7946 | 1.3400 |
| ｐ23049 | 42 | 0.63 (0.6, 0.66) | 39 | 0.67 (0.64, 0.7) | 0.0899 | 1.3398 |
| ｐ16722 | 42 | 0.42 (0.24, 0.6) | 39 | 0.68 (0.5, 0.86) | 0.6954 | 1.3372 |
| ｐ15625 | 42 | 0.32 (0.17, 0.46) | 39 | 0.52 (0.38, 0.67) | 0.7253 | 1.3354 |
| ｐ16179 | 42 | 0.11 (-0.12, 0.34) | 39 | 0.44 (0.21, 0.68) | 1.9950 | 1.3335 |
| ｐ15471 | 42 | 0.09 (0.01, 0.18) | 39 | 0.22 (0.13, 0.31) | 1.2025 | 1.3325 |
| ｐ15404 | 42 | 0.41 (0.26, 0.57) | 39 | 0.64 (0.48, 0.8) | 0.6294 | 1.3307 |
| ｐ20190 | 42 | 0.35 (0.28, 0.42) | 39 | 0.25 (0.17, 0.32) | -0.5026 | 1.3239 |
| ｐ15856 | 42 | 0.28 (0.18, 0.37) | 39 | 0.14 (0.04, 0.24) | -1.0006 | 1.3233 |
| ｐ6861 | 42 | 0.26 (0.2, 0.32) | 39 | 0.17 (0.11, 0.23) | -0.6174 | 1.3211 |
| ｐ12685 | 42 | 0.57 (0.07, 1.08) | 39 | 1.31 (0.78, 1.83) | 1.1923 | 1.3166 |
| ｐ18008 | 42 | 0.24 (0.05, 0.44) | 39 | 0.53 (0.32, 0.73) | 1.1253 | 1.3161 |
| ｐ11130 | 42 | 0.44 (0.31, 0.57) | 39 | 0.63 (0.49, 0.76) | 0.5137 | 1.3159 |
| ｐ17916 | 42 | 0.49 (0.45, 0.52) | 39 | 0.54 (0.5, 0.58) | 0.1448 | 1.3152 |
| ｐ14909 | 42 | 0.44 (0.29, 0.58) | 39 | 0.64 (0.5, 0.79) | 0.5643 | 1.3149 |
| ｐ20219 | 42 | 0.44 (0.29, 0.58) | 39 | 0.64 (0.5, 0.79) | 0.5643 | 1.3149 |
| ｐ16832 | 42 | 1.07 (0.63, 1.51) | 39 | 1.71 (1.25, 2.16) | 0.6739 | 1.3125 |
| ｐ10084 | 42 | 0.38 (0.02, 0.74) | 39 | 0.91 (0.53, 1.28) | 1.2444 | 1.3111 |
| ｐ16142 | 42 | 0.39 (0.29, 0.48) | 39 | 0.53 (0.43, 0.63) | 0.4433 | 1.3089 |
| ｐ18422 | 42 | 0.38 (0.34, 0.42) | 39 | 0.43 (0.39, 0.47) | 0.2053 | 1.3080 |
| ｐ15190 | 42 | 0.41 (0.29, 0.53) | 39 | 0.23 (0.11, 0.36) | -0.8050 | 1.3072 |
| ｐ331 | 42 | 1.58 (1.34, 1.82) | 39 | 1.23 (0.98, 1.48) | -0.3580 | 1.3015 |
| ｐ15444 | 42 | 0.46 (0.35, 0.58) | 39 | 0.62 (0.51, 0.74) | 0.4242 | 1.3002 |
| ｐ16990 | 42 | 1.33 (1.32, 1.35) | 39 | 1.35 (1.34, 1.37) | 0.0212 | 1.3002 |
| ｐ12438 | 42 | 0.34 (0.23, 0.45) | 39 | 0.5 (0.38, 0.61) | 0.5544 | 1.2990 |
| ｐ12786 | 42 | 1.16 (1.11, 1.22) | 39 | 1.25 (1.19, 1.31) | 0.0997 | 1.2966 |
| ｐ12985 | 42 | 0.1 (0.01, 0.18) | 39 | 0.22 (0.13, 0.3) | 1.1502 | 1.2949 |
| ｐ13386 | 42 | 0.63 (0.52, 0.75) | 39 | 0.8 (0.68, 0.92) | 0.3386 | 1.2944 |
| ｐ11247 | 42 | 1.17 (0.57, 1.77) | 33 | 0.26 (-0.42, 0.94) | -2.1479 | 1.2925 |
| ｐ18057 | 42 | 0.45 (0.32, 0.58) | 39 | 0.63 (0.5, 0.76) | 0.4879 | 1.2924 |
| ｐ15150 | 42 | 3.22 (1.4, 5.04) | 39 | 0.62 (-1.27, 2.5) | -2.3869 | 1.2874 |
| ｐ15733 | 42 | 0.91 (0.36, 1.46) | 39 | 0.13 (-0.44, 0.7) | -2.8432 | 1.2874 |
| ｐ19116 | 42 | 0.91 (0.36, 1.46) | 39 | 0.13 (-0.44, 0.7) | -2.8432 | 1.2874 |
| ｐ14566 | 41 | 0.21 (-0.05, 0.46) | 39 | 0.56 (0.31, 0.82) | 1.4503 | 1.2871 |
| ｐ20962 | 41 | 0.21 (-0.05, 0.46) | 39 | 0.56 (0.31, 0.82) | 1.4503 | 1.2871 |
| ｐ150 | 42 | 0.58 (0.32, 0.84) | 39 | 0.96 (0.68, 1.23) | 0.7159 | 1.2861 |
| ｐ15485 | 42 | 0.09 (-0.01, 0.18) | 39 | 0.22 (0.12, 0.32) | 1.3439 | 1.2848 |
| ｐ8512 | 42 | 0.29 (0.19, 0.4) | 39 | 0.14 (0.04, 0.25) | -1.0177 | 1.2833 |
| ｐ16660 | 42 | 0.99 (-0.11, 2.09) | 39 | 2.56 (1.41, 3.7) | 1.3749 | 1.2832 |
| ｐ2367 | 42 | 0.57 (0.22, 0.92) | 39 | 1.07 (0.71, 1.44) | 0.9043 | 1.2817 |
| ｐ428 | 42 | 0.97 (0.82, 1.12) | 39 | 1.19 (1.03, 1.35) | 0.2925 | 1.2802 |
| ｐ184 | 42 | 0.61 (0.33, 0.89) | 39 | 1.01 (0.72, 1.3) | 0.7269 | 1.2791 |
| ｐ22681 | 42 | 1 (0.99, 1.01) | 39 | 1.02 (1.01, 1.03) | 0.0240 | 1.2791 |
| ｐ20937 | 42 | 0.49 (0.48, 0.5) | 39 | 0.5 (0.49, 0.51) | 0.0358 | 1.2791 |
| ｐ22770 | 42 | 0.72 (0.71, 0.73) | 39 | 0.74 (0.73, 0.75) | 0.0257 | 1.2791 |
| ｐ22860 | 42 | 0.33 (0.26, 0.4) | 39 | 0.43 (0.36, 0.51) | 0.3832 | 1.2791 |
| ｐ21328 | 42 | 0.6 (0.57, 0.64) | 39 | 0.66 (0.62, 0.7) | 0.1205 | 1.2791 |
| ｐ22728 | 42 | 0.02 (-0.03, 0.08) | 39 | 0.11 (0.05, 0.17) | 2.1302 | 1.2791 |
| ｐ20644 | 42 | 0.42 (0.33, 0.52) | 39 | 0.56 (0.46, 0.66) | 0.4020 | 1.2791 |
| ｐ21278 | 42 | 0.42 (0.33, 0.52) | 39 | 0.56 (0.46, 0.66) | 0.4020 | 1.2791 |
| ｐ22726 | 42 | 0.36 (0.34, 0.37) | 39 | 0.38 (0.36, 0.4) | 0.0923 | 1.2791 |
| ｐ22704 | 42 | 0.58 (0.56, 0.6) | 39 | 0.61 (0.59, 0.63) | 0.0620 | 1.2791 |
| ｐ22677 | 42 | 0.61 (0.6, 0.62) | 39 | 0.63 (0.62, 0.64) | 0.0387 | 1.2791 |
| ｐ20604 | 42 | 0.24 (0.16, 0.32) | 39 | 0.36 (0.27, 0.44) | 0.5617 | 1.2791 |
| ｐ22708 | 42 | 0.21 (0.14, 0.28) | 39 | 0.31 (0.24, 0.39) | 0.5590 | 1.2791 |
| ｐ14602 | 42 | 0.38 (0.27, 0.5) | 39 | 0.55 (0.43, 0.67) | 0.5200 | 1.2791 |
| ｐ21013 | 42 | 0.13 (0.09, 0.16) | 39 | 0.17 (0.14, 0.2) | 0.4382 | 1.2791 |
| ｐ21235 | 42 | 0.31 (0.25, 0.37) | 39 | 0.4 (0.33, 0.46) | 0.3560 | 1.2791 |
| ｐ22802 | 42 | 0.21 (0.18, 0.25) | 39 | 0.26 (0.22, 0.29) | 0.2975 | 1.2791 |
| ｐ22762 | 42 | 0.55 (0.49, 0.61) | 39 | 0.63 (0.57, 0.69) | 0.1960 | 1.2791 |
| ｐ20608 | 42 | 0.57 (0.51, 0.62) | 39 | 0.65 (0.59, 0.71) | 0.1932 | 1.2791 |
| ｐ13796 | 42 | 0.45 (0.42, 0.49) | 39 | 0.5 (0.47, 0.54) | 0.1570 | 1.2791 |
| ｐ22738 | 42 | 0.11 (-0.01, 0.23) | 39 | 0.28 (0.16, 0.41) | 1.3718 | 1.2791 |
| ｐ21287 | 42 | 0.26 (0.12, 0.41) | 39 | 0.47 (0.32, 0.62) | 0.8340 | 1.2791 |
| ｐ22772 | 42 | 0.09 (0.05, 0.14) | 39 | 0.16 (0.11, 0.21) | 0.7647 | 1.2791 |
| ｐ20527 | 42 | 0.2 (0.11, 0.29) | 39 | 0.33 (0.23, 0.42) | 0.7134 | 1.2791 |
| ｐ22743 | 42 | 0.33 (0.23, 0.42) | 39 | 0.46 (0.36, 0.56) | 0.5076 | 1.2791 |
| ｐ22687 | 42 | 0.57 (0.44, 0.69) | 39 | 0.74 (0.61, 0.86) | 0.3858 | 1.2791 |
| ｐ22692 | 42 | 0.79 (0.74, 0.83) | 39 | 0.85 (0.8, 0.9) | 0.1117 | 1.2791 |
| ｐ13470 | 42 | 0.69 (0.68, 0.7) | 39 | 0.7 (0.69, 0.71) | 0.0262 | 1.2791 |
| ｐ22706 | 42 | 0.31 (0.24, 0.38) | 39 | 0.41 (0.34, 0.48) | 0.4005 | 1.2791 |
| ｐ22797 | 42 | 0.65 (0.55, 0.75) | 39 | 0.8 (0.69, 0.9) | 0.2902 | 1.2791 |
| ｐ22719 | 42 | 0.24 (0.2, 0.27) | 39 | 0.28 (0.25, 0.31) | 0.2477 | 1.2791 |
| ｐ13005 | 42 | 0.36 (0.32, 0.4) | 39 | 0.42 (0.38, 0.46) | 0.2129 | 1.2791 |
| ｐ20611 | 42 | 0.36 (0.32, 0.4) | 39 | 0.42 (0.38, 0.46) | 0.2129 | 1.2791 |
| ｐ22789 | 42 | 0.59 (0.53, 0.66) | 39 | 0.68 (0.62, 0.75) | 0.2099 | 1.2791 |
| ｐ22808 | 42 | 0.42 (0.4, 0.44) | 39 | 0.45 (0.43, 0.48) | 0.1143 | 1.2791 |
| ｐ22696 | 42 | 0.59 (0.56, 0.62) | 39 | 0.63 (0.6, 0.66) | 0.0929 | 1.2791 |
| ｐ22749 | 42 | 0.76 (0.75, 0.77) | 39 | 0.78 (0.77, 0.79) | 0.0366 | 1.2791 |
| ｐ22735 | 42 | 0.8 (0.74, 0.87) | 39 | 0.9 (0.83, 0.97) | 0.1610 | 1.2791 |
| ｐ22689 | 42 | 0.37 (0.34, 0.39) | 39 | 0.4 (0.38, 0.43) | 0.1338 | 1.2791 |
| ｐ21440 | 42 | 0.35 (0.33, 0.37) | 39 | 0.38 (0.36, 0.4) | 0.1099 | 1.2791 |
| ｐ10925 | 42 | 0.83 (0.79, 0.88) | 39 | 0.89 (0.85, 0.94) | 0.1013 | 1.2791 |
| ｐ3508 | 42 | 0.5 (0.48, 0.53) | 39 | 0.53 (0.51, 0.56) | 0.0894 | 1.2791 |
| ｐ22716 | 42 | 0.56 (0.54, 0.59) | 39 | 0.6 (0.57, 0.62) | 0.0869 | 1.2791 |
| ｐ22718 | 42 | 0.4 (0.39, 0.42) | 39 | 0.42 (0.41, 0.44) | 0.0779 | 1.2791 |
| ｐ22804 | 42 | 0.94 (0.9, 0.98) | 39 | 0.99 (0.96, 1.03) | 0.0778 | 1.2791 |
| ｐ13784 | 42 | 0.62 (0.59, 0.64) | 39 | 0.65 (0.63, 0.68) | 0.0766 | 1.2791 |
| ｐ6957 | 42 | 0.83 (0.82, 0.85) | 39 | 0.85 (0.84, 0.86) | 0.0300 | 1.2791 |
| ｐ22787 | 42 | 0.17 (0.07, 0.28) | 39 | 0.32 (0.21, 0.42) | 0.8738 | 1.2791 |
| ｐ22693 | 42 | 0.64 (0.5, 0.77) | 39 | 0.83 (0.69, 0.97) | 0.3814 | 1.2791 |
| ｐ20066 | 42 | 0.24 (0.2, 0.28) | 39 | 0.3 (0.26, 0.34) | 0.3033 | 1.2791 |
| ｐ22725 | 42 | 0.53 (0.5, 0.57) | 39 | 0.58 (0.55, 0.62) | 0.1273 | 1.2791 |
| ｐ22742 | 42 | 0.38 (0.37, 0.4) | 39 | 0.4 (0.39, 0.41) | 0.0672 | 1.2791 |
| ｐ22684 | 42 | 0.31 (0.3, 0.31) | 39 | 0.32 (0.31, 0.33) | 0.0595 | 1.2791 |
| ｐ22791 | 42 | 0.15 (0.06, 0.24) | 39 | 0.28 (0.19, 0.38) | 0.8805 | 1.2791 |
| ｐ22769 | 42 | 0.19 (0.16, 0.22) | 39 | 0.23 (0.2, 0.26) | 0.2650 | 1.2791 |
| ｐ22767 | 42 | 0.46 (0.43, 0.5) | 39 | 0.52 (0.48, 0.56) | 0.1605 | 1.2791 |
| ｐ22695 | 42 | 0.48 (0.44, 0.51) | 39 | 0.52 (0.49, 0.56) | 0.1349 | 1.2791 |
| ｐ21295 | 42 | 0.72 (0.67, 0.77) | 39 | 0.79 (0.74, 0.84) | 0.1313 | 1.2791 |
| ｐ22715 | 42 | 0.51 (0.48, 0.54) | 39 | 0.55 (0.52, 0.58) | 0.1066 | 1.2791 |
| ｐ22711 | 42 | 0.53 (0.51, 0.55) | 39 | 0.56 (0.54, 0.58) | 0.0693 | 1.2791 |
| ｐ13280 | 42 | 0.04 (-0.01, 0.09) | 39 | 0.12 (0.06, 0.17) | 1.4584 | 1.2791 |
| ｐ22799 | 42 | 0.09 (0.02, 0.17) | 39 | 0.21 (0.12, 0.29) | 1.1265 | 1.2791 |
| ｐ20768 | 42 | 0.15 (0.1, 0.21) | 39 | 0.23 (0.17, 0.29) | 0.6068 | 1.2791 |
| ｐ22759 | 42 | 0.86 (0.69, 1.03) | 39 | 1.1 (0.93, 1.28) | 0.3636 | 1.2791 |
| ｐ22707 | 42 | 0.53 (0.46, 0.59) | 39 | 0.62 (0.55, 0.69) | 0.2416 | 1.2791 |
| ｐ22760 | 42 | 0.45 (0.4, 0.5) | 39 | 0.52 (0.47, 0.58) | 0.2241 | 1.2791 |
| ｐ10049 | 42 | 0.44 (0.41, 0.48) | 39 | 0.49 (0.46, 0.53) | 0.1580 | 1.2791 |
| ｐ13015 | 42 | 0.4 (0.37, 0.43) | 39 | 0.44 (0.41, 0.47) | 0.1399 | 1.2791 |
| ｐ22720 | 42 | 0.26 (0.24, 0.28) | 39 | 0.28 (0.26, 0.3) | 0.1349 | 1.2791 |
| ｐ22753 | 42 | 0.9 (0.84, 0.96) | 39 | 0.98 (0.92, 1.05) | 0.1321 | 1.2791 |
| ｐ22807 | 42 | 1.22 (1.16, 1.29) | 39 | 1.31 (1.25, 1.38) | 0.1052 | 1.2791 |
| ｐ13496 | 42 | 0.63 (0.6, 0.66) | 39 | 0.67 (0.64, 0.71) | 0.0924 | 1.2791 |
| ｐ22754 | 42 | 0.36 (0.34, 0.37) | 39 | 0.37 (0.36, 0.39) | 0.0732 | 1.2791 |
| ｐ22688 | 42 | 0.78 (0.77, 0.8) | 39 | 0.81 (0.79, 0.82) | 0.0384 | 1.2791 |
| ｐ21191 | 42 | 0.15 (-0.19, 0.48) | 39 | 0.62 (0.28, 0.97) | 2.0729 | 1.2791 |
| ｐ11149 | 42 | 0.86 (0.84, 0.87) | 39 | 0.87 (0.86, 0.89) | 0.0278 | 1.2791 |
| ｐ11395 | 42 | 0.2 (0.1, 0.3) | 39 | 0.35 (0.24, 0.45) | 0.7842 | 1.2791 |
| ｐ22806 | 42 | 0.36 (0.28, 0.44) | 39 | 0.48 (0.39, 0.56) | 0.4066 | 1.2791 |
| ｐ13007 | 42 | 0.69 (0.64, 0.74) | 39 | 0.76 (0.71, 0.81) | 0.1382 | 1.2791 |
| ｐ21318 | 42 | 0.72 (0.68, 0.75) | 39 | 0.77 (0.73, 0.81) | 0.1057 | 1.2791 |
| ｐ22776 | 42 | 0.5 (0.47, 0.52) | 39 | 0.53 (0.51, 0.56) | 0.1057 | 1.2791 |
| ｐ22774 | 42 | 0.41 (0.39, 0.43) | 39 | 0.44 (0.42, 0.46) | 0.0954 | 1.2791 |
| ｐ22798 | 42 | 0.82 (0.79, 0.86) | 39 | 0.87 (0.84, 0.91) | 0.0826 | 1.2791 |
| ｐ8961 | 42 | 0.09 (0.02, 0.15) | 39 | 0.18 (0.11, 0.24) | 1.0292 | 1.2791 |
| ｐ22965 | 42 | 0.11 (0.04, 0.19) | 39 | 0.22 (0.14, 0.3) | 0.9613 | 1.2791 |
| ｐ22747 | 42 | 0.19 (0.09, 0.28) | 39 | 0.32 (0.22, 0.42) | 0.7932 | 1.2791 |
| ｐ22766 | 42 | 0.4 (0.35, 0.46) | 39 | 0.48 (0.43, 0.54) | 0.2500 | 1.2791 |
| ｐ22712 | 42 | 0.07 (0, 0.15) | 39 | 0.18 (0.1, 0.26) | 1.2745 | 1.2791 |
| ｐ22751 | 42 | 0.63 (0.62, 0.63) | 39 | 0.63 (0.63, 0.64) | 0.0208 | 1.2791 |
| ｐ22746 | 42 | 0.57 (0.57, 0.58) | 39 | 0.58 (0.57, 0.58) | 0.0188 | 1.2791 |
| ｐ18185 | 42 | 0.04 (-0.03, 0.1) | 39 | 0.12 (0.06, 0.19) | 1.7949 | 1.2790 |
| ｐ16270 | 42 | 0.04 (0.01, 0.07) | 39 | 0.08 (0.05, 0.11) | 1.0975 | 1.2790 |
| ｐ19808 | 42 | 0.15 (0.06, 0.24) | 39 | 0.28 (0.19, 0.38) | 0.8770 | 1.2790 |
| ｐ16233 | 42 | 0.33 (0.26, 0.4) | 39 | 0.43 (0.36, 0.51) | 0.3832 | 1.2790 |
| ｐ16124 | 42 | 0.38 (0.3, 0.46) | 39 | 0.49 (0.41, 0.58) | 0.3824 | 1.2790 |
| ｐ16307 | 42 | 0.38 (0.3, 0.46) | 39 | 0.49 (0.41, 0.58) | 0.3824 | 1.2790 |
| ｐ18267 | 42 | 0.49 (0.41, 0.57) | 39 | 0.6 (0.52, 0.68) | 0.2897 | 1.2790 |
| ｐ19480 | 42 | 0.49 (0.42, 0.56) | 39 | 0.59 (0.52, 0.66) | 0.2647 | 1.2790 |
| ｐ19350 | 42 | 1.52 (1.31, 1.74) | 39 | 1.83 (1.61, 2.05) | 0.2633 | 1.2790 |
| ｐ18106 | 42 | 0.2 (0.17, 0.22) | 39 | 0.23 (0.21, 0.26) | 0.2410 | 1.2790 |
| ｐ18521 | 42 | 0.21 (0.18, 0.23) | 39 | 0.24 (0.22, 0.26) | 0.2154 | 1.2790 |
| ｐ15032 | 42 | 0.33 (0.29, 0.36) | 39 | 0.37 (0.34, 0.41) | 0.1946 | 1.2790 |
| ｐ16434 | 42 | 0.66 (0.6, 0.73) | 39 | 0.75 (0.69, 0.82) | 0.1809 | 1.2790 |
| ｐ18169 | 42 | 0.69 (0.63, 0.75) | 39 | 0.77 (0.71, 0.84) | 0.1689 | 1.2790 |
| ｐ18296 | 42 | 0.36 (0.33, 0.39) | 39 | 0.41 (0.37, 0.44) | 0.1614 | 1.2790 |
| ｐ18058 | 42 | 0.45 (0.41, 0.48) | 39 | 0.49 (0.46, 0.52) | 0.1399 | 1.2790 |
| ｐ18758 | 42 | 0.69 (0.64, 0.74) | 39 | 0.76 (0.71, 0.81) | 0.1382 | 1.2790 |
| ｐ18081 | 42 | 0.54 (0.51, 0.57) | 39 | 0.58 (0.55, 0.62) | 0.1158 | 1.2790 |
| ｐ17400 | 42 | 0.68 (0.64, 0.72) | 39 | 0.74 (0.7, 0.78) | 0.1133 | 1.2790 |
| ｐ18682 | 42 | 0.28 (0.27, 0.3) | 39 | 0.3 (0.29, 0.32) | 0.1049 | 1.2790 |
| ｐ17439 | 42 | 0.99 (0.94, 1.04) | 39 | 1.06 (1.01, 1.11) | 0.0960 | 1.2790 |
| ｐ16679 | 42 | 0.63 (0.6, 0.66) | 39 | 0.67 (0.64, 0.7) | 0.0949 | 1.2790 |
| ｐ17565 | 42 | 0.46 (0.45, 0.48) | 39 | 0.48 (0.47, 0.5) | 0.0642 | 1.2790 |
| ｐ16725 | 42 | 0.53 (0.52, 0.54) | 39 | 0.54 (0.53, 0.55) | 0.0397 | 1.2790 |
| ｐ17246 | 42 | 0.78 (0.76, 0.79) | 39 | 0.79 (0.78, 0.8) | 0.0299 | 1.2790 |
| ｐ14937 | 42 | 0.27 (0.19, 0.35) | 39 | 0.15 (0.06, 0.24) | -0.8318 | 1.2773 |
| ｐ14771 | 42 | 0.57 (0.45, 0.69) | 39 | 0.4 (0.27, 0.52) | -0.5201 | 1.2759 |
| ｐ15501 | 42 | 0.14 (0, 0.28) | 39 | 0.34 (0.2, 0.49) | 1.2528 | 1.2733 |
| ｐ13760 | 42 | 0.35 (0.16, 0.54) | 39 | 0.62 (0.42, 0.82) | 0.8198 | 1.2730 |
| ｐ16671 | 42 | 0.48 (-4.48, 5.44) | 39 | 7.52 (2.37, 12.67) | 3.9737 | 1.2725 |
| ｐ15488 | 42 | 0.42 (0.38, 0.45) | 39 | 0.47 (0.43, 0.51) | 0.1610 | 1.2716 |
| ｐ20656 | 42 | 0.14 (-0.15, 0.43) | 39 | 0.55 (0.25, 0.85) | 1.9530 | 1.2688 |
| ｐ91 | 42 | 0.63 (0.48, 0.77) | 39 | 0.42 (0.27, 0.57) | -0.5758 | 1.2647 |
| ｐ17177 | 42 | 0.57 (-0.28, 1.42) | 39 | 1.76 (0.89, 2.64) | 1.6316 | 1.2612 |
| ｐ13890 | 42 | 0.1 (0.04, 0.17) | 39 | 0.19 (0.13, 0.26) | 0.8882 | 1.2597 |
| ｐ22800 | 42 | 0.14 (0.07, 0.21) | 39 | 0.24 (0.17, 0.32) | 0.7946 | 1.2587 |
| ｐ12114 | 42 | 0.07 (0.05, 0.09) | 39 | 0.05 (0.03, 0.07) | -0.6369 | 1.2568 |
| ｐ19782 | 42 | 0.43 (0.38, 0.47) | 39 | 0.49 (0.44, 0.53) | 0.1903 | 1.2557 |
| ｐ22451 | 42 | 0 (-0.09, 0.09) | 39 | 0.12 (0.03, 0.21) | NA | 1.2527 |
| ｐ22459 | 42 | 0 (-0.17, 0.17) | 39 | 0.24 (0.06, 0.41) | NA | 1.2505 |
| ｐ12552 | 42 | 0.87 (0.81, 0.93) | 39 | 0.95 (0.89, 1.01) | 0.1327 | 1.2502 |
| ｐ22412 | 42 | 0 (-0.04, 0.04) | 39 | 0.06 (0.02, 0.11) | NA | 1.2502 |
| ｐ14438 | 42 | 1.18 (0.93, 1.44) | 39 | 0.83 (0.57, 1.09) | -0.5123 | 1.2494 |
| ｐ15441 | 42 | 0.24 (0.17, 0.31) | 39 | 0.14 (0.07, 0.22) | -0.7578 | 1.2472 |
| ｐ13072 | 42 | 0.27 (0.2, 0.34) | 39 | 0.36 (0.29, 0.43) | 0.4460 | 1.2441 |
| ｐ22443 | 42 | 0.88 (0.12, 1.63) | 39 | 1.93 (1.15, 2.72) | 1.1383 | 1.2425 |
| ｐ15531 | 42 | 0.51 (0.39, 0.63) | 39 | 0.67 (0.55, 0.79) | 0.4007 | 1.2396 |
| ｐ13792 | 42 | 0.52 (0.4, 0.64) | 39 | 0.68 (0.56, 0.81) | 0.3921 | 1.2383 |
| ｐ14723 | 42 | 0.39 (0.29, 0.48) | 39 | 0.25 (0.15, 0.35) | -0.6135 | 1.2381 |
| ｐ8510 | 42 | 0.34 (0.29, 0.4) | 39 | 0.42 (0.36, 0.48) | 0.2970 | 1.2368 |
| ｐ22937 | 42 | 0.29 (0.02, 0.56) | 39 | 0.67 (0.39, 0.95) | 1.2113 | 1.2352 |
| ｐ12767 | 40 | 0.25 (0.19, 0.3) | 39 | 0.32 (0.27, 0.38) | 0.3859 | 1.2322 |
| ｐ16030 | 42 | 0.33 (0.22, 0.43) | 39 | 0.18 (0.07, 0.29) | -0.8787 | 1.2291 |
| ｐ17078 | 42 | 0.28 (0.16, 0.39) | 39 | 0.12 (0, 0.24) | -1.1938 | 1.2291 |
| ｐ276 | 42 | 0.36 (0.26, 0.45) | 39 | 0.22 (0.12, 0.32) | -0.6746 | 1.2290 |
| ｐ17810 | 42 | 0.42 (0.31, 0.54) | 39 | 0.26 (0.14, 0.38) | -0.6919 | 1.2284 |
| ｐ14206 | 42 | 0.95 (0.39, 1.51) | 39 | 1.72 (1.14, 2.3) | 0.8594 | 1.2253 |
| ｐ13786 | 42 | 1.06 (0.58, 1.54) | 39 | 0.39 (-0.11, 0.89) | -1.4345 | 1.2246 |
| ｐ15611 | 42 | 0.11 (0.04, 0.17) | 39 | 0.2 (0.13, 0.26) | 0.8753 | 1.2226 |
| ｐ15345 | 42 | 0.17 (0.12, 0.21) | 39 | 0.23 (0.18, 0.28) | 0.4761 | 1.2226 |
| ｐ20663 | 42 | 0.48 (0.3, 0.65) | 39 | 0.72 (0.54, 0.91) | 0.6038 | 1.2199 |
| ｐ16207 | 42 | 0.25 (0.2, 0.3) | 39 | 0.18 (0.13, 0.23) | -0.4550 | 1.2197 |
| ｐ14642 | 42 | 0.2 (0.13, 0.28) | 39 | 0.1 (0.03, 0.18) | -0.9679 | 1.2179 |
| ｐ18572 | 42 | 0.31 (0.22, 0.4) | 39 | 0.19 (0.09, 0.28) | -0.7406 | 1.2154 |
| ｐ16627 | 42 | 0.25 (0.23, 0.26) | 39 | 0.27 (0.25, 0.28) | 0.1149 | 1.2147 |
| ｐ4522 | 42 | 0.68 (0.65, 0.71) | 39 | 0.64 (0.6, 0.67) | -0.0965 | 1.2141 |
| ｐ336 | 42 | 0.69 (0.68, 0.71) | 39 | 0.71 (0.7, 0.73) | 0.0441 | 1.2116 |
| ｐ9196 | 42 | 0.04 (-0.05, 0.13) | 39 | 0.16 (0.07, 0.26) | 2.0223 | 1.2065 |
| ｐ15582 | 42 | 0.38 (0.28, 0.49) | 39 | 0.52 (0.42, 0.63) | 0.4538 | 1.2062 |
| ｐ15546 | 42 | 0.6 (0.59, 0.62) | 39 | 0.63 (0.61, 0.64) | 0.0530 | 1.2013 |
| ｐ15178 | 42 | 0.31 (0.24, 0.38) | 39 | 0.22 (0.15, 0.29) | -0.5157 | 1.2013 |
| ｐ14879 | 42 | 0.62 (0.43, 0.8) | 39 | 0.37 (0.18, 0.56) | -0.7483 | 1.2008 |
| ｐ18822 | 42 | 0.15 (0.02, 0.28) | 39 | 0.33 (0.19, 0.47) | 1.1537 | 1.2000 |
| ｐ19700 | 42 | 1.1 (0.66, 1.54) | 39 | 1.71 (1.25, 2.16) | 0.6286 | 1.2000 |
| ｐ12919 | 42 | 0.55 (0.26, 0.84) | 39 | 0.95 (0.65, 1.25) | 0.7790 | 1.1997 |
| ｐ5978 | 42 | 0.41 (0.26, 0.56) | 39 | 0.2 (0.05, 0.36) | -1.0067 | 1.1994 |
| ｐ2408 | 42 | 0.32 (0.24, 0.39) | 39 | 0.22 (0.14, 0.29) | -0.5497 | 1.1961 |
| ｐ836 | 42 | 0.62 (0.5, 0.74) | 39 | 0.45 (0.33, 0.58) | -0.4414 | 1.1945 |
| ｐ2 | 42 | 0.44 (0.3, 0.58) | 39 | 0.25 (0.1, 0.39) | -0.8145 | 1.1935 |
| ｐ5031 | 42 | 0.27 (0.22, 0.32) | 39 | 0.2 (0.14, 0.25) | -0.4375 | 1.1916 |
| ｐ12715 | 42 | 0.34 (0.28, 0.4) | 39 | 0.25 (0.19, 0.32) | -0.4181 | 1.1897 |
| ｐ2407 | 42 | 0.6 (0.5, 0.71) | 39 | 0.46 (0.35, 0.57) | -0.3934 | 1.1869 |
| ｐ13758 | 42 | 1.24 (0.98, 1.5) | 39 | 0.89 (0.62, 1.16) | -0.4799 | 1.1867 |
| ｐ16309 | 42 | 0.28 (0.2, 0.36) | 39 | 0.39 (0.31, 0.47) | 0.4726 | 1.1831 |
| ｐ14925 | 42 | 0.48 (0.42, 0.54) | 39 | 0.56 (0.5, 0.62) | 0.2243 | 1.1812 |
| ｐ12647 | 41 | 0.61 (0.44, 0.78) | 39 | 0.38 (0.2, 0.55) | -0.6957 | 1.1784 |
| ｐ15202 | 42 | 0.67 (0.55, 0.79) | 39 | 0.51 (0.38, 0.64) | -0.4031 | 1.1778 |
| ｐ2985 | 42 | 0.16 (0.04, 0.27) | 39 | 0 (-0.12, 0.12) | NA | 1.1768 |
| ｐ16688 | 42 | 0.35 (0.12, 0.59) | 39 | 0.67 (0.42, 0.91) | 0.9286 | 1.1752 |
| ｐ17080 | 42 | 3.06 (1.24, 4.88) | 39 | 0.62 (-1.27, 2.5) | -2.3149 | 1.1752 |
| ｐ22399 | 42 | 0 (-0.17, 0.17) | 39 | 0.23 (0.05, 0.41) | NA | 1.1748 |
| ｐ6004 | 42 | 0.32 (0.21, 0.42) | 39 | 0.46 (0.35, 0.57) | 0.5394 | 1.1737 |
| ｐ16146 | 42 | 0.25 (0.18, 0.32) | 39 | 0.15 (0.08, 0.23) | -0.6869 | 1.1733 |
| ｐ15365 | 42 | 0.36 (0.21, 0.52) | 39 | 0.57 (0.41, 0.73) | 0.6581 | 1.1726 |
| ｐ20412 | 42 | 0.34 (0.29, 0.39) | 39 | 0.41 (0.36, 0.47) | 0.2764 | 1.1716 |
| ｐ17657 | 42 | 0.23 (-0.04, 0.5) | 39 | 0.6 (0.31, 0.88) | 1.3734 | 1.1713 |
| ｐ22780 | 42 | 0.2 (0.04, 0.35) | 39 | 0.4 (0.24, 0.56) | 1.0333 | 1.1703 |
| ｐ22240 | 42 | 0.11 (0.05, 0.17) | 39 | 0.03 (-0.03, 0.09) | -1.8022 | 1.1693 |
| ｐ17210 | 42 | 0.33 (0.25, 0.4) | 39 | 0.23 (0.15, 0.31) | -0.5247 | 1.1675 |
| ｐ11257 | 42 | 0.39 (0.26, 0.51) | 39 | 0.21 (0.08, 0.35) | -0.8545 | 1.1672 |
| ｐ16897 | 42 | 0.44 (0.28, 0.61) | 39 | 0.66 (0.49, 0.84) | 0.5876 | 1.1669 |
| ｐ16704 | 42 | 0.07 (-0.33, 0.47) | 39 | 0.61 (0.19, 1.02) | 3.0800 | 1.1656 |
| ｐ17000 | 42 | 0.53 (0.36, 0.71) | 39 | 0.3 (0.12, 0.48) | -0.8295 | 1.1656 |
| ｐ22777 | 42 | 0.39 (0.18, 0.59) | 39 | 0.66 (0.45, 0.88) | 0.7816 | 1.1655 |
| ｐ6210 | 42 | 0.94 (0.56, 1.33) | 39 | 0.43 (0.02, 0.83) | -1.1478 | 1.1648 |
| ｐ14981 | 42 | 0.99 (0.6, 1.38) | 39 | 0.47 (0.07, 0.88) | -1.0615 | 1.1636 |
| ｐ14730 | 42 | 0.27 (0.11, 0.43) | 39 | 0.48 (0.32, 0.64) | 0.8379 | 1.1635 |
| ｐ17410 | 42 | 0.28 (0.22, 0.35) | 39 | 0.37 (0.3, 0.44) | 0.3939 | 1.1630 |
| ｐ19083 | 42 | 0.19 (0.15, 0.23) | 39 | 0.24 (0.2, 0.28) | 0.3525 | 1.1630 |
| ｐ22025 | 42 | 0.22 (0.15, 0.28) | 39 | 0.31 (0.24, 0.37) | 0.4882 | 1.1629 |
| ｐ4942 | 42 | 0.15 (0.09, 0.21) | 39 | 0.07 (0.01, 0.13) | -1.1288 | 1.1601 |
| ｐ22669 | 42 | 0.49 (0.38, 0.6) | 39 | 0.63 (0.52, 0.74) | 0.3749 | 1.1595 |
| ｐ14242 | 42 | 0.72 (0.04, 1.4) | 39 | 1.63 (0.92, 2.34) | 1.1710 | 1.1562 |
| ｐ15366 | 42 | 0.43 (0.21, 0.66) | 39 | 0.74 (0.5, 0.97) | 0.7639 | 1.1561 |
| ｐ19026 | 42 | 0.61 (0.45, 0.77) | 39 | 0.4 (0.23, 0.56) | -0.6154 | 1.1543 |
| ｐ15771 | 42 | 0.2 (0.16, 0.24) | 39 | 0.15 (0.11, 0.19) | -0.4210 | 1.1537 |
| ｐ22792 | 42 | 0.07 (0.05, 0.1) | 39 | 0.1 (0.08, 0.13) | 0.5071 | 1.1524 |
| ｐ21955 | 42 | 0.12 (0.1, 0.14) | 39 | 0.14 (0.12, 0.16) | 0.2442 | 1.1508 |
| ｐ8839 | 42 | 2.94 (1.17, 4.71) | 39 | 0.6 (-1.24, 2.43) | -2.3017 | 1.1500 |
| ｐ9517 | 42 | 0.18 (-0.45, 0.8) | 39 | 1 (0.35, 1.65) | 2.5167 | 1.1479 |
| ｐ20657 | 42 | 0.3 (0.22, 0.38) | 39 | 0.41 (0.32, 0.5) | 0.4536 | 1.1476 |
| ｐ15383 | 42 | 0.47 (-0.39, 1.32) | 39 | 1.59 (0.71, 2.48) | 1.7761 | 1.1475 |
| ｐ2382 | 41 | 2.17 (0.95, 3.38) | 39 | 3.77 (2.52, 5.01) | 0.7974 | 1.1475 |
| ｐ21004 | 42 | 0.03 (-0.2, 0.26) | 39 | 0.33 (0.1, 0.56) | 3.4314 | 1.1473 |
| ｐ12386 | 42 | 0.33 (0.26, 0.4) | 39 | 0.42 (0.35, 0.49) | 0.3515 | 1.1470 |
| ｐ20845 | 42 | 0.33 (0.26, 0.4) | 39 | 0.42 (0.35, 0.49) | 0.3515 | 1.1470 |
| ｐ1166 | 42 | 0.57 (0.39, 0.75) | 39 | 0.33 (0.15, 0.52) | -0.7706 | 1.1468 |
| ｐ22685 | 42 | 0.51 (0.39, 0.63) | 39 | 0.67 (0.54, 0.79) | 0.3887 | 1.1467 |
| ｐ16075 | 42 | 0.34 (0.09, 0.59) | 39 | 0.67 (0.41, 0.93) | 0.9780 | 1.1445 |
| ｐ12255 | 42 | 0.32 (0.22, 0.41) | 39 | 0.19 (0.1, 0.29) | -0.7023 | 1.1434 |
| ｐ15492 | 42 | 0.52 (0.38, 0.66) | 39 | 0.7 (0.56, 0.85) | 0.4337 | 1.1433 |
| ｐ15309 | 42 | 0.38 (0.25, 0.51) | 39 | 0.21 (0.07, 0.34) | -0.8638 | 1.1415 |
| ｐ20913 | 42 | 0.96 (0.9, 1.02) | 39 | 0.88 (0.82, 0.94) | -0.1268 | 1.1404 |
| ｐ13722 | 42 | 0.08 (0.04, 0.12) | 39 | 0.03 (0, 0.07) | -1.2995 | 1.1404 |
| ｐ14734 | 42 | 0.41 (0.39, 0.43) | 39 | 0.38 (0.36, 0.4) | -0.1069 | 1.1404 |
| ｐ22214 | 42 | 0.42 (0.3, 0.53) | 39 | 0.27 (0.15, 0.39) | -0.6383 | 1.1404 |
| ｐ916 | 42 | 0.19 (0.13, 0.25) | 39 | 0.11 (0.05, 0.17) | -0.7900 | 1.1404 |
| ｐ22526 | 42 | 0.43 (0.41, 0.45) | 39 | 0.4 (0.38, 0.42) | -0.0913 | 1.1404 |
| ｐ11854 | 42 | 0.35 (0.33, 0.37) | 39 | 0.32 (0.3, 0.34) | -0.1342 | 1.1404 |
| ｐ20556 | 42 | 0.18 (0.16, 0.21) | 39 | 0.15 (0.12, 0.18) | -0.2978 | 1.1404 |
| ｐ12774 | 42 | 0.53 (0.45, 0.61) | 39 | 0.43 (0.35, 0.51) | -0.3056 | 1.1404 |
| ｐ22478 | 42 | 0.4 (0.34, 0.46) | 39 | 0.32 (0.26, 0.39) | -0.3159 | 1.1404 |
| ｐ21813 | 42 | 0.67 (0.65, 0.7) | 39 | 0.64 (0.62, 0.67) | -0.0679 | 1.1404 |
| ｐ3510 | 42 | 0.44 (0.41, 0.46) | 39 | 0.41 (0.38, 0.43) | -0.0984 | 1.1404 |
| ｐ14793 | 42 | 0.54 (0.47, 0.61) | 39 | 0.44 (0.37, 0.52) | -0.2798 | 1.1404 |
| ｐ1554 | 42 | 0.41 (0.37, 0.46) | 39 | 0.35 (0.3, 0.4) | -0.2294 | 1.1404 |
| ｐ20671 | 42 | 0.14 (0.09, 0.19) | 39 | 0.08 (0.03, 0.13) | -0.8845 | 1.1404 |
| ｐ20910 | 42 | 0.45 (0.34, 0.56) | 39 | 0.3 (0.19, 0.42) | -0.5670 | 1.1404 |
| ｐ13169 | 42 | 0.22 (0.2, 0.25) | 39 | 0.19 (0.17, 0.22) | -0.2262 | 1.1404 |
| ｐ10060 | 42 | 0.55 (0.51, 0.58) | 39 | 0.5 (0.47, 0.54) | -0.1104 | 1.1404 |
| ｐ11479 | 42 | 0.52 (0.49, 0.55) | 39 | 0.48 (0.44, 0.51) | -0.1194 | 1.1404 |
| ｐ22246 | 42 | 0.48 (0.43, 0.52) | 39 | 0.42 (0.37, 0.47) | -0.1889 | 1.1404 |
| ｐ12222 | 42 | 0.54 (0.48, 0.61) | 39 | 0.46 (0.39, 0.52) | -0.2439 | 1.1404 |
| ｐ5275 | 42 | 0.23 (0.18, 0.28) | 39 | 0.16 (0.11, 0.22) | -0.5132 | 1.1404 |
| ｐ12738 | 42 | 0.53 (0.51, 0.54) | 39 | 0.51 (0.5, 0.52) | -0.0510 | 1.1404 |
| ｐ22474 | 42 | 0.49 (0.46, 0.51) | 39 | 0.46 (0.43, 0.48) | -0.0914 | 1.1404 |
| ｐ22553 | 42 | 0.27 (0.25, 0.29) | 39 | 0.24 (0.23, 0.26) | -0.1365 | 1.1404 |
| ｐ13708 | 42 | 0.4 (0.34, 0.47) | 39 | 0.32 (0.25, 0.39) | -0.3422 | 1.1404 |
| ｐ21275 | 42 | 0.36 (0.35, 0.37) | 39 | 0.35 (0.34, 0.36) | -0.0541 | 1.1404 |
| ｐ20112 | 42 | 0.25 (0.2, 0.3) | 39 | 0.19 (0.14, 0.24) | -0.4280 | 1.1404 |
| ｐ21950 | 42 | 0.35 (0.34, 0.36) | 39 | 0.34 (0.33, 0.35) | -0.0551 | 1.1404 |
| ｐ22525 | 42 | 0.51 (0.49, 0.53) | 39 | 0.48 (0.47, 0.5) | -0.0685 | 1.1404 |
| ｐ22863 | 42 | 0.3 (0.25, 0.34) | 39 | 0.24 (0.19, 0.28) | -0.3293 | 1.1404 |
| ｐ20418 | 42 | 0.27 (0.2, 0.34) | 39 | 0.18 (0.1, 0.25) | -0.6069 | 1.1404 |
| ｐ13198 | 42 | 0.23 (0.18, 0.28) | 39 | 0.17 (0.12, 0.22) | -0.4563 | 1.1404 |
| ｐ12724 | 42 | 0.32 (0.2, 0.44) | 39 | 0.17 (0.04, 0.29) | -0.9544 | 1.1404 |
| ｐ14813 | 42 | 0.2 (0.14, 0.25) | 39 | 0.12 (0.06, 0.18) | -0.6973 | 1.1404 |
| ｐ12793 | 42 | 0.08 (0.03, 0.12) | 39 | 0.02 (-0.03, 0.06) | -2.0695 | 1.1404 |
| ｐ21680 | 42 | 0.57 (0.47, 0.66) | 39 | 0.44 (0.34, 0.54) | -0.3712 | 1.1404 |
| ｐ18988 | 42 | 0.52 (0.5, 0.53) | 39 | 0.5 (0.48, 0.51) | -0.0493 | 1.1403 |
| ｐ18193 | 42 | 0.99 (0.97, 1.02) | 39 | 0.96 (0.93, 0.99) | -0.0554 | 1.1403 |
| ｐ18238 | 42 | 0.57 (0.54, 0.59) | 39 | 0.54 (0.51, 0.56) | -0.0757 | 1.1403 |
| ｐ16205 | 42 | 0.7 (0.67, 0.73) | 39 | 0.66 (0.63, 0.69) | -0.0817 | 1.1403 |
| ｐ16811 | 42 | 0.52 (0.5, 0.55) | 39 | 0.49 (0.47, 0.52) | -0.0855 | 1.1403 |
| ｐ16302 | 42 | 0.64 (0.61, 0.67) | 39 | 0.6 (0.57, 0.63) | -0.0967 | 1.1403 |
| ｐ15310 | 42 | 0.5 (0.48, 0.53) | 39 | 0.47 (0.45, 0.5) | -0.0973 | 1.1403 |
| ｐ19117 | 42 | 0.7 (0.66, 0.74) | 39 | 0.65 (0.61, 0.69) | -0.1034 | 1.1403 |
| ｐ16915 | 42 | 0.31 (0.29, 0.33) | 39 | 0.29 (0.27, 0.31) | -0.1290 | 1.1403 |
| ｐ16743 | 42 | 0.53 (0.49, 0.57) | 39 | 0.48 (0.44, 0.52) | -0.1452 | 1.1403 |
| ｐ17469 | 42 | 0.5 (0.46, 0.53) | 39 | 0.45 (0.41, 0.49) | -0.1462 | 1.1403 |
| ｐ19152 | 42 | 0.45 (0.41, 0.48) | 39 | 0.4 (0.36, 0.44) | -0.1619 | 1.1403 |
| ｐ15218 | 42 | 0.43 (0.39, 0.46) | 39 | 0.38 (0.34, 0.42) | -0.1666 | 1.1403 |
| ｐ16224 | 42 | 0.51 (0.46, 0.55) | 39 | 0.45 (0.41, 0.5) | -0.1743 | 1.1403 |
| ｐ15786 | 42 | 0.38 (0.34, 0.42) | 39 | 0.33 (0.29, 0.37) | -0.2062 | 1.1403 |
| ｐ16376 | 42 | 0.38 (0.34, 0.42) | 39 | 0.33 (0.29, 0.37) | -0.2062 | 1.1403 |
| ｐ18357 | 42 | 0.38 (0.34, 0.42) | 39 | 0.33 (0.29, 0.37) | -0.2062 | 1.1403 |
| ｐ19309 | 42 | 0.38 (0.34, 0.42) | 39 | 0.33 (0.29, 0.37) | -0.2062 | 1.1403 |
| ｐ16737 | 42 | 0.41 (0.37, 0.46) | 39 | 0.35 (0.3, 0.4) | -0.2294 | 1.1403 |
| ｐ15103 | 42 | 0.58 (0.51, 0.65) | 39 | 0.49 (0.43, 0.56) | -0.2319 | 1.1403 |
| ｐ18919 | 42 | 0.49 (0.43, 0.55) | 39 | 0.41 (0.35, 0.47) | -0.2431 | 1.1403 |
| ｐ17860 | 42 | 0.41 (0.36, 0.47) | 39 | 0.35 (0.29, 0.4) | -0.2553 | 1.1403 |
| ｐ18191 | 42 | 0.25 (0.21, 0.29) | 39 | 0.2 (0.16, 0.24) | -0.3129 | 1.1403 |
| ｐ19394 | 42 | 0.57 (0.47, 0.66) | 39 | 0.44 (0.34, 0.54) | -0.3712 | 1.1403 |
| ｐ15207 | 42 | 0.37 (0.29, 0.44) | 39 | 0.27 (0.19, 0.35) | -0.4518 | 1.1403 |
| ｐ18973 | 42 | 0.35 (0.26, 0.44) | 39 | 0.23 (0.14, 0.32) | -0.6034 | 1.1403 |
| ｐ18782 | 42 | 0.42 (0.31, 0.54) | 39 | 0.27 (0.15, 0.39) | -0.6408 | 1.1403 |
| ｐ15304 | 42 | 0.41 (0.28, 0.53) | 39 | 0.24 (0.1, 0.37) | -0.7753 | 1.1403 |
| ｐ18359 | 42 | 0.19 (0.13, 0.26) | 39 | 0.11 (0.04, 0.17) | -0.8613 | 1.1403 |
| ｐ19198 | 42 | 0.22 (0.14, 0.3) | 39 | 0.11 (0.03, 0.2) | -0.9811 | 1.1403 |
| ｐ19425 | 42 | 0.26 (0.16, 0.36) | 39 | 0.12 (0.02, 0.23) | -1.0769 | 1.1403 |
| ｐ15862 | 42 | 0.13 (0.07, 0.2) | 39 | 0.04 (-0.03, 0.11) | -1.5964 | 1.1403 |
| ｐ15333 | 42 | 0.08 (0.03, 0.12) | 39 | 0.01 (-0.04, 0.06) | -2.4388 | 1.1403 |
| ｐ14086 | 42 | 0.28 (0.18, 0.39) | 39 | 0.42 (0.31, 0.53) | 0.5647 | 1.1375 |
| ｐ16516 | 42 | 0.78 (0.65, 0.91) | 39 | 0.95 (0.82, 1.08) | 0.2848 | 1.1373 |
| ｐ22781 | 42 | 0.5 (0.4, 0.61) | 39 | 0.64 (0.53, 0.75) | 0.3437 | 1.1358 |
| ｐ16183 | 42 | 0.67 (0.35, 0.98) | 39 | 1.08 (0.76, 1.41) | 0.6938 | 1.1343 |
| ｐ13580 | 42 | 0.48 (0.36, 0.6) | 39 | 0.32 (0.19, 0.45) | -0.5808 | 1.1334 |
| ｐ16573 | 42 | 0 (-0.05, 0.05) | 39 | 0.07 (0.01, 0.12) | NA | 1.1325 |
| ｐ14736 | 42 | 0.41 (0.3, 0.52) | 39 | 0.27 (0.15, 0.38) | -0.6193 | 1.1297 |
| ｐ12316 | 42 | 0.26 (0.17, 0.36) | 39 | 0.14 (0.04, 0.24) | -0.9508 | 1.1269 |
| ｐ22009 | 42 | 0 (-0.04, 0.04) | 39 | 0.06 (0.01, 0.11) | NA | 1.1264 |
| ｐ22953 | 42 | 0.31 (0.29, 0.33) | 39 | 0.34 (0.31, 0.36) | 0.1191 | 1.1239 |
| ｐ17285 | 42 | 0.63 (0.56, 0.71) | 39 | 0.73 (0.65, 0.81) | 0.2107 | 1.1232 |
| ｐ11256 | 42 | 0.45 (0.35, 0.54) | 39 | 0.32 (0.22, 0.42) | -0.4703 | 1.1217 |
| ｐ20087 | 42 | 0.06 (0.01, 0.11) | 39 | 0 (-0.05, 0.05) | NA | 1.1212 |
| ｐ22078 | 42 | 0 (-0.06, 0.06) | 39 | 0.08 (0.02, 0.15) | NA | 1.1200 |
| ｐ19945 | 42 | 0.49 (0.36, 0.62) | 39 | 0.66 (0.53, 0.8) | 0.4363 | 1.1198 |
| ｐ8918 | 42 | 0.36 (0.31, 0.41) | 39 | 0.29 (0.24, 0.35) | -0.2913 | 1.1180 |
| ｐ15572 | 42 | 0.14 (0.08, 0.2) | 39 | 0.21 (0.15, 0.28) | 0.6342 | 1.1169 |
| ｐ16624 | 42 | 0.17 (0.09, 0.26) | 39 | 0.07 (-0.01, 0.15) | -1.3299 | 1.1169 |
| ｐ20198 | 42 | 0.56 (0.3, 0.81) | 39 | 0.22 (-0.05, 0.49) | -1.3345 | 1.1167 |
| ｐ22764 | 42 | 0.57 (0.33, 0.81) | 39 | 0.88 (0.63, 1.14) | 0.6371 | 1.1166 |
| ｐ16255 | 42 | 0.63 (0.34, 0.92) | 39 | 1 (0.71, 1.3) | 0.6713 | 1.1146 |
| ｐ15576 | 42 | 0.33 (0.24, 0.42) | 39 | 0.44 (0.35, 0.53) | 0.4281 | 1.1141 |
| ｐ98 | 42 | 0.38 (0.16, 0.6) | 39 | 0.67 (0.44, 0.89) | 0.8079 | 1.1137 |
| ｐ15901 | 42 | 0.3 (0.22, 0.38) | 39 | 0.41 (0.32, 0.49) | 0.4417 | 1.1113 |
| ｐ21292 | 42 | 0.31 (0.14, 0.47) | 39 | 0.52 (0.35, 0.7) | 0.7757 | 1.1088 |
| ｐ16736 | 42 | 0.45 (0.24, 0.67) | 39 | 0.73 (0.51, 0.96) | 0.6927 | 1.1062 |
| ｐ6404 | 42 | 0.23 (0.19, 0.27) | 39 | 0.18 (0.14, 0.22) | -0.3641 | 1.1060 |
| ｐ15319 | 42 | 0.28 (0.18, 0.39) | 39 | 0.15 (0.04, 0.26) | -0.9604 | 1.1057 |
| ｐ17777 | 42 | 0.57 (0.5, 0.64) | 39 | 0.48 (0.41, 0.55) | -0.2422 | 1.1007 |
| ｐ22006 | 42 | 0.12 (-0.02, 0.26) | 39 | 0.3 (0.16, 0.45) | 1.3092 | 1.0953 |
| ｐ72 | 42 | 0.27 (-0.05, 0.6) | 39 | 0.7 (0.36, 1.04) | 1.3432 | 1.0944 |
| ｐ16640 | 42 | 0.19 (-0.14, 0.51) | 39 | 0.6 (0.27, 0.94) | 1.6932 | 1.0942 |
| ｐ14795 | 42 | 0.07 (-0.02, 0.17) | 39 | 0.19 (0.1, 0.29) | 1.3681 | 1.0897 |
| ｐ10415 | 42 | 0.42 (0.22, 0.63) | 39 | 0.68 (0.47, 0.89) | 0.6922 | 1.0894 |
| ｐ22755 | 42 | 0.14 (-0.51, 0.78) | 39 | 0.96 (0.29, 1.64) | 2.8177 | 1.0889 |
| ｐ15313 | 42 | 0.16 (0.09, 0.23) | 39 | 0.07 (0, 0.14) | -1.1578 | 1.0888 |
| ｐ19351 | 42 | 0.33 (0.28, 0.39) | 39 | 0.4 (0.35, 0.46) | 0.2723 | 1.0883 |
| ｐ15581 | 42 | 0.86 (0.81, 0.9) | 39 | 0.92 (0.87, 0.96) | 0.0960 | 1.0883 |
| ｐ22691 | 42 | 0.35 (0.26, 0.44) | 39 | 0.47 (0.37, 0.56) | 0.4105 | 1.0850 |
| ｐ16796 | 42 | 0.24 (0.09, 0.39) | 39 | 0.04 (-0.11, 0.2) | -2.4120 | 1.0846 |
| ｐ12722 | 42 | 0.13 (0.08, 0.17) | 39 | 0.07 (0.03, 0.12) | -0.7935 | 1.0842 |
| ｐ16188 | 42 | 0.9 (0.05, 1.76) | 39 | 1.99 (1.11, 2.88) | 1.1411 | 1.0841 |
| ｐ13343 | 42 | 0.13 (0.09, 0.16) | 39 | 0.08 (0.05, 0.12) | -0.6074 | 1.0838 |
| ｐ16154 | 42 | 0.28 (0.22, 0.35) | 39 | 0.37 (0.3, 0.43) | 0.3654 | 1.0835 |
| ｐ16752 | 42 | 0.13 (0.09, 0.16) | 39 | 0.08 (0.05, 0.12) | -0.6074 | 1.0835 |
| ｐ15461 | 42 | 0.47 (0.45, 0.49) | 39 | 0.5 (0.48, 0.52) | 0.0880 | 1.0820 |
| ｐ240 | 42 | 0.4 (0.13, 0.67) | 39 | 0.74 (0.46, 1.02) | 0.8884 | 1.0777 |
| ｐ13067 | 42 | 0.23 (0.16, 0.3) | 39 | 0.14 (0.06, 0.21) | -0.7557 | 1.0741 |
| ｐ6370 | 42 | 0.57 (0.34, 0.81) | 39 | 0.87 (0.63, 1.11) | 0.5967 | 1.0739 |
| ｐ10036 | 42 | 0.42 (0.33, 0.51) | 39 | 0.3 (0.21, 0.4) | -0.4637 | 1.0712 |
| ｐ20171 | 41 | 0.56 (0.54, 0.58) | 39 | 0.59 (0.56, 0.61) | 0.0691 | 1.0708 |
| ｐ1241 | 42 | 0.7 (0.53, 0.87) | 39 | 0.49 (0.31, 0.66) | -0.5192 | 1.0697 |
| ｐ304 | 42 | 5.26 (-1.21, 11.74) | 39 | 13.43 (6.71, 20.15) | 1.3516 | 1.0683 |
| ｐ12335 | 42 | 1.94 (0.21, 3.67) | 39 | 4.12 (2.33, 5.91) | 1.0847 | 1.0663 |
| ｐ15224 | 42 | 0.5 (0.39, 0.61) | 39 | 0.36 (0.24, 0.48) | -0.4788 | 1.0630 |
| ｐ374 | 42 | 0.65 (0.53, 0.76) | 39 | 0.51 (0.39, 0.62) | -0.3522 | 1.0616 |
| ｐ346 | 42 | 0.63 (0.59, 0.67) | 39 | 0.68 (0.64, 0.72) | 0.1095 | 1.0613 |
| ｐ15678 | 42 | 0.49 (0.37, 0.6) | 39 | 0.63 (0.51, 0.74) | 0.3651 | 1.0595 |
| ｐ21151 | 42 | 0.19 (0.06, 0.33) | 39 | 0.36 (0.22, 0.5) | 0.8992 | 1.0592 |
| ｐ19333 | 42 | 0.24 (0.11, 0.38) | 39 | 0.41 (0.27, 0.55) | 0.7498 | 1.0575 |
| ｐ22407 | 42 | 0.11 (0.04, 0.18) | 39 | 0.2 (0.12, 0.28) | 0.8866 | 1.0572 |
| ｐ11680 | 42 | 0.48 (0.33, 0.63) | 39 | 0.67 (0.51, 0.83) | 0.4857 | 1.0569 |
| ｐ19597 | 42 | 0.51 (0.33, 0.7) | 39 | 0.28 (0.09, 0.47) | -0.8756 | 1.0565 |
| ｐ22662 | 42 | 1.07 (-0.28, 2.42) | 39 | 2.76 (1.36, 4.16) | 1.3689 | 1.0564 |
| ｐ15871 | 42 | 0.24 (0.18, 0.29) | 39 | 0.17 (0.11, 0.22) | -0.5165 | 1.0555 |
| ｐ17090 | 42 | 0.82 (0.74, 0.9) | 39 | 0.92 (0.84, 1.01) | 0.1657 | 1.0540 |
| ｐ22439 | 42 | 0 (-0.07, 0.07) | 39 | 0.08 (0.01, 0.15) | NA | 1.0538 |
| ｐ22419 | 42 | 0.38 (-0.02, 0.77) | 39 | 0.87 (0.46, 1.27) | 1.2011 | 1.0538 |
| ｐ12893 | 42 | 0.44 (0.27, 0.61) | 39 | 0.23 (0.05, 0.4) | -0.9637 | 1.0528 |
| ｐ19510 | 42 | 0.52 (0.17, 0.86) | 33 | 0.96 (0.58, 1.35) | 0.8989 | 1.0521 |
| ｐ7954 | 42 | 0.45 (0.26, 0.63) | 39 | 0.21 (0.02, 0.41) | -1.0645 | 1.0512 |
| ｐ14 | 42 | 0.2 (0.14, 0.26) | 39 | 0.27 (0.21, 0.33) | 0.4571 | 1.0507 |
| ｐ16625 | 42 | 1.01 (0.73, 1.29) | 39 | 0.67 (0.38, 0.95) | -0.6059 | 1.0506 |
| ｐ9975 | 42 | 0.26 (0.17, 0.35) | 39 | 0.15 (0.06, 0.24) | -0.7625 | 1.0483 |
| ｐ22012 | 42 | 0 (-0.1, 0.1) | 39 | 0.13 (0.02, 0.23) | NA | 1.0475 |
| ｐ10472 | 42 | 0.14 (0.04, 0.23) | 39 | 0.26 (0.16, 0.36) | 0.9349 | 1.0463 |
| ｐ22740 | 42 | 0.7 (0.58, 0.82) | 39 | 0.85 (0.72, 0.98) | 0.2823 | 1.0457 |
| ｐ11386 | 42 | 0.44 (0.33, 0.54) | 39 | 0.57 (0.46, 0.68) | 0.3817 | 1.0436 |
| ｐ22723 | 42 | 0.59 (0.5, 0.68) | 39 | 0.7 (0.61, 0.8) | 0.2543 | 1.0426 |
| ｐ14357 | 42 | 0.22 (0.14, 0.3) | 39 | 0.13 (0.05, 0.2) | -0.7968 | 1.0423 |
| ｐ12477 | 42 | 0.17 (0.15, 0.19) | 39 | 0.14 (0.12, 0.16) | -0.2265 | 1.0414 |
| ｐ16773 | 42 | 0.51 (-0.12, 1.13) | 39 | 1.28 (0.63, 1.93) | 1.3335 | 1.0410 |
| ｐ22698 | 42 | 0.16 (0.08, 0.23) | 39 | 0.25 (0.17, 0.33) | 0.6962 | 1.0397 |
| ｐ16956 | 42 | 0.07 (0.01, 0.13) | 39 | 0 (-0.06, 0.06) | NA | 1.0391 |
| ｐ22775 | 42 | 0.85 (0.43, 1.27) | 39 | 1.37 (0.93, 1.8) | 0.6877 | 1.0384 |
| ｐ23033 | 42 | 0.56 (0.48, 0.65) | 39 | 0.67 (0.58, 0.76) | 0.2526 | 1.0363 |
| ｐ18090 | 41 | 0.43 (0.27, 0.59) | 39 | 0.63 (0.46, 0.79) | 0.5464 | 1.0353 |
| ｐ19128 | 42 | 0.25 (0.18, 0.32) | 39 | 0.34 (0.27, 0.41) | 0.4212 | 1.0353 |
| ｐ22064 | 42 | 0 (-0.11, 0.11) | 39 | 0.14 (0.02, 0.25) | NA | 1.0332 |
| ｐ22724 | 42 | 0.2 (0.17, 0.23) | 39 | 0.24 (0.21, 0.28) | 0.2600 | 1.0328 |
| ｐ16837 | 42 | 0.32 (0.12, 0.52) | 39 | 0.56 (0.36, 0.77) | 0.8268 | 1.0306 |
| ｐ15628 | 42 | 0.41 (0.28, 0.53) | 39 | 0.56 (0.43, 0.69) | 0.4600 | 1.0292 |
| ｐ18171 | 42 | 0.61 (0.46, 0.76) | 39 | 0.79 (0.64, 0.94) | 0.3755 | 1.0287 |
| ｐ17558 | 42 | 0.36 (0.24, 0.48) | 39 | 0.21 (0.08, 0.34) | -0.7829 | 1.0283 |
| ｐ14850 | 42 | 0.5 (0.27, 0.73) | 39 | 0.22 (-0.02, 0.45) | -1.2040 | 1.0282 |
| ｐ22101 | 42 | 0 (-0.05, 0.05) | 39 | 0.06 (0.01, 0.11) | NA | 1.0272 |
| ｐ22768 | 42 | 0.69 (0.56, 0.82) | 39 | 0.85 (0.71, 0.98) | 0.3009 | 1.0268 |
| ｐ10921 | 42 | 0.27 (0.19, 0.34) | 39 | 0.17 (0.09, 0.25) | -0.6239 | 1.0267 |
| ｐ12034 | 42 | 0.22 (0.13, 0.31) | 39 | 0.33 (0.23, 0.42) | 0.5907 | 1.0263 |
| ｐ8595 | 42 | 0.46 (0.23, 0.68) | 39 | 0.73 (0.5, 0.96) | 0.6781 | 1.0256 |
| ｐ21504 | 42 | 0.19 (0.08, 0.29) | 39 | 0.31 (0.21, 0.42) | 0.7433 | 1.0255 |
| ｐ18072 | 42 | 0.33 (0.27, 0.39) | 39 | 0.26 (0.2, 0.32) | -0.3595 | 1.0237 |
| ｐ14855 | 42 | 1.06 (0.56, 1.57) | 39 | 0.44 (-0.09, 0.97) | -1.2711 | 1.0236 |
| ｐ22954 | 42 | 0.57 (0.52, 0.63) | 39 | 0.64 (0.58, 0.7) | 0.1632 | 1.0224 |
| ｐ14348 | 42 | 0.23 (0.19, 0.28) | 39 | 0.18 (0.13, 0.23) | -0.4172 | 1.0220 |
| ｐ14345 | 42 | 0.8 (0.36, 1.24) | 39 | 0.26 (-0.19, 0.72) | -1.6006 | 1.0216 |
| ｐ22690 | 42 | 0.75 (0.25, 1.25) | 39 | 1.36 (0.84, 1.87) | 0.8560 | 1.0196 |
| ｐ11783 | 42 | 0.86 (0.24, 1.48) | 39 | 0.11 (-0.53, 0.75) | -2.9900 | 1.0194 |
| ｐ17988 | 42 | 0.34 (0.23, 0.45) | 39 | 0.47 (0.36, 0.58) | 0.4643 | 1.0182 |
| ｐ16715 | 42 | 0.26 (0.19, 0.33) | 39 | 0.34 (0.27, 0.41) | 0.3895 | 1.0182 |
| ｐ852 | 42 | 0.46 (0.26, 0.66) | 39 | 0.71 (0.5, 0.92) | 0.6241 | 1.0155 |
| ｐ15969 | 42 | 0.28 (0.14, 0.43) | 39 | 0.46 (0.31, 0.61) | 0.6993 | 1.0137 |
| ｐ19113 | 42 | 0.4 (0.31, 0.49) | 39 | 0.51 (0.41, 0.6) | 0.3552 | 1.0132 |
| ｐ10297 | 42 | 0.35 (0.27, 0.43) | 39 | 0.26 (0.18, 0.34) | -0.4563 | 1.0129 |
| ｐ10442 | 42 | 0.5 (0.44, 0.55) | 39 | 0.43 (0.38, 0.49) | -0.2061 | 1.0128 |
| ｐ22401 | 42 | 0.37 (0.26, 0.49) | 39 | 0.52 (0.4, 0.64) | 0.4694 | 1.0125 |
| ｐ19035 | 42 | 0.38 (0.15, 0.62) | 39 | 0.67 (0.43, 0.92) | 0.8043 | 1.0114 |
| ｐ8603 | 42 | 0.35 (0.29, 0.4) | 39 | 0.28 (0.21, 0.34) | -0.3314 | 1.0109 |
| ｐ15270 | 42 | 0.27 (0.22, 0.31) | 39 | 0.21 (0.17, 0.26) | -0.3082 | 1.0101 |
| ｐ18781 | 42 | 0.69 (0.58, 0.79) | 39 | 0.56 (0.45, 0.67) | -0.2933 | 1.0097 |
| ｐ16040 | 42 | 0.38 (0.32, 0.43) | 39 | 0.45 (0.39, 0.5) | 0.2416 | 1.0083 |
| ｐ14570 | 42 | 0.21 (0.08, 0.34) | 39 | 0.36 (0.23, 0.49) | 0.7928 | 1.0072 |
| ｐ17685 | 42 | 0.5 (0.39, 0.62) | 39 | 0.64 (0.52, 0.76) | 0.3532 | 1.0057 |
| ｐ15652 | 42 | 0.65 (0.51, 0.8) | 39 | 0.83 (0.68, 0.98) | 0.3414 | 1.0057 |
| ｐ15470 | 42 | 0.64 (0.56, 0.72) | 39 | 0.73 (0.65, 0.81) | 0.1945 | 1.0052 |
| ｐ15248 | 42 | 0.25 (0.14, 0.36) | 39 | 0.38 (0.27, 0.5) | 0.6125 | 1.0048 |
| ｐ937 | 42 | 0.16 (0.13, 0.19) | 39 | 0.13 (0.1, 0.16) | -0.3524 | 1.0032 |
| ｐ22758 | 42 | 0.97 (0.8, 1.13) | 39 | 1.17 (0.99, 1.34) | 0.2733 | 1.0028 |
| ｐ9549 | 42 | 0.23 (0.13, 0.32) | 39 | 0.34 (0.24, 0.44) | 0.5895 | 1.0016 |
| ｐ19377 | 42 | 0.16 (0.09, 0.22) | 39 | 0.24 (0.17, 0.3) | 0.5894 | 1.0009 |
| ｐ20610 | 42 | 0.21 (0.13, 0.29) | 39 | 0.31 (0.22, 0.4) | 0.5624 | 1.0002 |
| ｐ16995 | 42 | 0.35 (0.29, 0.4) | 39 | 0.28 (0.22, 0.34) | -0.3274 | 1.0000 |
| ｐ19774 | 42 | 0.29 (0.22, 0.35) | 39 | 0.21 (0.15, 0.28) | -0.4415 | 0.9974 |
| ｐ16742 | 42 | 0.3 (0.24, 0.36) | 39 | 0.23 (0.18, 0.29) | -0.3617 | 0.9970 |
| ｐ19708 | 42 | 0.48 (0.33, 0.62) | 39 | 0.3 (0.15, 0.45) | -0.6619 | 0.9970 |
| ｐ22269 | 42 | 0.57 (0.56, 0.59) | 39 | 0.59 (0.58, 0.6) | 0.0328 | 0.9957 |
| ｐ16749 | 42 | 0.52 (0.25, 0.78) | 39 | 0.84 (0.56, 1.11) | 0.6943 | 0.9944 |
| ｐ13564 | 42 | 0.7 (0.51, 0.89) | 39 | 0.48 (0.28, 0.67) | -0.5575 | 0.9933 |
| ｐ22790 | 42 | 0.4 (0.3, 0.5) | 39 | 0.52 (0.41, 0.62) | 0.3817 | 0.9931 |
| ｐ14777 | 42 | 0.9 (0.46, 1.33) | 39 | 0.37 (-0.08, 0.83) | -1.2683 | 0.9919 |
| ｐ8653 | 42 | 0.33 (-0.19, 0.84) | 39 | 0.94 (0.41, 1.47) | 1.5289 | 0.9910 |
| ｐ15870 | 42 | 0.33 (-0.19, 0.84) | 39 | 0.94 (0.41, 1.47) | 1.5289 | 0.9910 |
| ｐ20978 | 42 | 0.28 (0.14, 0.41) | 39 | 0.44 (0.3, 0.58) | 0.6539 | 0.9873 |
| ｐ12573 | 42 | 0.55 (0.39, 0.71) | 39 | 0.35 (0.18, 0.52) | -0.6338 | 0.9861 |
| ｐ15195 | 42 | 0.73 (0.55, 0.9) | 39 | 0.93 (0.75, 1.11) | 0.3610 | 0.9855 |
| ｐ16074 | 42 | 0.17 (0.12, 0.21) | 39 | 0.11 (0.07, 0.16) | -0.5431 | 0.9851 |
| ｐ16594 | 42 | 0.43 (0.15, 0.7) | 39 | 0.1 (-0.18, 0.38) | -2.0892 | 0.9851 |
| ｐ22168 | 42 | 0.49 (0.42, 0.55) | 39 | 0.56 (0.5, 0.63) | 0.2083 | 0.9843 |
| ｐ22803 | 42 | 0.32 (0.23, 0.41) | 39 | 0.42 (0.33, 0.51) | 0.4029 | 0.9833 |
| ｐ15276 | 42 | 0.19 (0.14, 0.24) | 39 | 0.13 (0.08, 0.18) | -0.5231 | 0.9830 |
| ｐ16314 | 42 | 0.1 (0.06, 0.14) | 39 | 0.15 (0.11, 0.19) | 0.5533 | 0.9825 |
| ｐ17487 | 42 | 1.95 (1.36, 2.54) | 39 | 1.24 (0.63, 1.86) | -0.6464 | 0.9821 |
| ｐ365 | 42 | 0.23 (0.12, 0.35) | 39 | 0.37 (0.25, 0.48) | 0.6449 | 0.9800 |
| ｐ15185 | 42 | 0.39 (0.31, 0.46) | 39 | 0.3 (0.22, 0.38) | -0.3792 | 0.9792 |
| ｐ9554 | 42 | 0.26 (0.2, 0.33) | 39 | 0.19 (0.12, 0.25) | -0.4955 | 0.9792 |
| ｐ15209 | 42 | 1.55 (0.88, 2.22) | 39 | 0.76 (0.06, 1.45) | -1.0346 | 0.9788 |
| ｐ71 | 42 | 0.04 (0, 0.07) | 39 | 0.08 (0.04, 0.12) | 1.0992 | 0.9780 |
| ｐ13774 | 42 | 0.42 (0.09, 0.75) | 39 | 0.81 (0.47, 1.15) | 0.9444 | 0.9778 |
| ｐ2370 | 42 | 0.61 (0.52, 0.71) | 39 | 0.5 (0.41, 0.6) | -0.2879 | 0.9772 |
| ｐ21257 | 42 | 0.42 (0.37, 0.47) | 39 | 0.48 (0.43, 0.54) | 0.1912 | 0.9762 |
| ｐ10225 | 42 | 0.89 (0.7, 1.08) | 39 | 0.67 (0.48, 0.86) | -0.4095 | 0.9731 |
| ｐ22067 | 42 | 0.06 (0.01, 0.1) | 39 | 0.11 (0.06, 0.16) | 0.9890 | 0.9721 |
| ｐ15264 | 42 | 0.2 (0.15, 0.26) | 39 | 0.14 (0.08, 0.19) | -0.5602 | 0.9718 |
| ｐ15372 | 42 | 0.43 (0.36, 0.51) | 39 | 0.52 (0.44, 0.6) | 0.2768 | 0.9714 |
| ｐ15089 | 42 | 2.62 (1.66, 3.57) | 39 | 1.49 (0.5, 2.48) | -0.8139 | 0.9706 |
| ｐ14835 | 42 | 0.46 (0.3, 0.62) | 39 | 0.65 (0.48, 0.81) | 0.5007 | 0.9704 |
| ｐ21833 | 42 | 0.51 (0.37, 0.65) | 39 | 0.67 (0.53, 0.81) | 0.3959 | 0.9702 |
| ｐ15780 | 42 | 0.63 (0.21, 1.06) | 39 | 1.14 (0.69, 1.58) | 0.8415 | 0.9690 |
| ｐ22984 | 42 | 2.36 (0.68, 4.04) | 39 | 4.34 (2.6, 6.08) | 0.8797 | 0.9689 |
| ｐ14652 | 42 | 0.32 (0.18, 0.46) | 39 | 0.15 (0.01, 0.3) | -1.0669 | 0.9686 |
| ｐ15380 | 42 | 0.6 (0.43, 0.78) | 39 | 0.39 (0.21, 0.58) | -0.6127 | 0.9682 |
| ｐ22039 | 42 | 0.19 (0.1, 0.28) | 39 | 0.29 (0.2, 0.39) | 0.6316 | 0.9633 |
| ｐ17341 | 42 | 0.72 (0.48, 0.96) | 39 | 0.44 (0.19, 0.69) | -0.7102 | 0.9630 |
| ｐ11912 | 42 | 0.26 (0.12, 0.41) | 39 | 0.43 (0.28, 0.58) | 0.7207 | 0.9625 |
| ｐ18271 | 42 | 0.61 (0.4, 0.82) | 39 | 0.85 (0.64, 1.07) | 0.4867 | 0.9614 |
| ｐ11749 | 42 | 0.65 (0.35, 0.95) | 39 | 1 (0.69, 1.31) | 0.6186 | 0.9595 |
| ｐ14333 | 42 | 0.61 (0.46, 0.76) | 39 | 0.43 (0.27, 0.59) | -0.4997 | 0.9595 |
| ｐ18343 | 42 | 0.75 (0.52, 0.98) | 39 | 0.48 (0.24, 0.72) | -0.6374 | 0.9594 |
| ｐ22761 | 42 | 0.31 (0.08, 0.54) | 39 | 0.58 (0.34, 0.81) | 0.9032 | 0.9572 |
| ｐ12954 | 42 | 0.14 (0.02, 0.26) | 39 | 0 (-0.13, 0.13) | NA | 0.9544 |
| ｐ22737 | 42 | 0.77 (0.52, 1.01) | 39 | 1.05 (0.8, 1.31) | 0.4576 | 0.9542 |
| ｐ22218 | 42 | 0.18 (0.11, 0.25) | 39 | 0.26 (0.19, 0.34) | 0.5546 | 0.9533 |
| ｐ19668 | 42 | 0.18 (0.11, 0.25) | 39 | 0.26 (0.19, 0.34) | 0.5546 | 0.9531 |
| ｐ14950 | 42 | 0.27 (0.23, 0.31) | 39 | 0.22 (0.18, 0.26) | -0.2873 | 0.9529 |
| ｐ22091 | 42 | 0.02 (0.01, 0.03) | 39 | 0.03 (0.02, 0.05) | 0.7612 | 0.9489 |
| ｐ19741 | 42 | 0.62 (0.51, 0.72) | 39 | 0.49 (0.38, 0.6) | -0.3251 | 0.9465 |
| ｐ16959 | 42 | 0.32 (0.29, 0.35) | 39 | 0.36 (0.32, 0.4) | 0.1688 | 0.9462 |
| ｐ14105 | 42 | 1.14 (0.87, 1.41) | 39 | 0.83 (0.55, 1.11) | -0.4629 | 0.9413 |
| ｐ22615 | 42 | 0.44 (0.24, 0.64) | 39 | 0.21 (0.01, 0.42) | -1.0610 | 0.9412 |
| ｐ14856 | 42 | 0.28 (0.21, 0.35) | 39 | 0.19 (0.12, 0.27) | -0.5255 | 0.9412 |
| ｐ11544 | 42 | 0.09 (0.03, 0.15) | 39 | 0.02 (-0.04, 0.08) | -2.1155 | 0.9409 |
| ｐ23002 | 42 | 0.13 (-0.04, 0.3) | 39 | 0.33 (0.15, 0.51) | 1.3537 | 0.9407 |
| ｐ22383 | 42 | 0.18 (0.11, 0.24) | 39 | 0.1 (0.04, 0.17) | -0.7538 | 0.9390 |
| ｐ22619 | 42 | 0.52 (0.5, 0.55) | 39 | 0.55 (0.53, 0.58) | 0.0786 | 0.9379 |
| ｐ67 | 42 | 0.01 (-0.15, 0.17) | 39 | 0.19 (0.03, 0.35) | 4.2597 | 0.9371 |
| ｐ17216 | 42 | 0.35 (0.27, 0.44) | 39 | 0.26 (0.18, 0.34) | -0.4439 | 0.9370 |
| ｐ14101 | 42 | 0.73 (0.63, 0.83) | 39 | 0.61 (0.51, 0.72) | -0.2511 | 0.9369 |
| ｐ13278 | 42 | 1.71 (0.68, 2.75) | 39 | 0.52 (-0.55, 1.59) | -1.7125 | 0.9367 |
| ｐ17040 | 42 | 0.42 (0.05, 0.79) | 39 | 0.85 (0.46, 1.23) | 1.0081 | 0.9344 |
| ｐ19033 | 42 | 0.49 (0.36, 0.61) | 39 | 0.34 (0.21, 0.47) | -0.5213 | 0.9337 |
| ｐ13556 | 42 | 0.76 (0.48, 1.04) | 39 | 0.44 (0.16, 0.73) | -0.7798 | 0.9318 |
| ｐ273 | 42 | 0.81 (0.64, 0.98) | 39 | 1 (0.83, 1.18) | 0.3103 | 0.9317 |
| ｐ15841 | 42 | 0.6 (0.55, 0.65) | 39 | 0.54 (0.49, 0.59) | -0.1412 | 0.9311 |
| ｐ17680 | 41 | 0.43 (0.3, 0.55) | 39 | 0.57 (0.44, 0.7) | 0.4131 | 0.9307 |
| ｐ22795 | 42 | 0.72 (0.57, 0.87) | 39 | 0.9 (0.74, 1.05) | 0.3084 | 0.9296 |
| ｐ17446 | 42 | 0.12 (0.03, 0.22) | 39 | 0.24 (0.14, 0.34) | 0.9296 | 0.9289 |
| ｐ11382 | 42 | 0.63 (0.43, 0.83) | 39 | 0.85 (0.65, 1.06) | 0.4385 | 0.9263 |
| ｐ22085 | 42 | 0.81 (0.69, 0.93) | 39 | 0.94 (0.82, 1.07) | 0.2201 | 0.9255 |
| ｐ12134 | 42 | 1.84 (0.2, 3.48) | 39 | 3.71 (2.01, 5.4) | 1.0104 | 0.9249 |
| ｐ15212 | 42 | 0.75 (0.75, 0.75) | 39 | 0.75 (0.75, 0.75) | -0.0016 | 0.9237 |
| ｐ15831 | 42 | 1.16 (1.15, 1.17) | 39 | 1.15 (1.14, 1.16) | -0.0127 | 0.9237 |
| ｐ19649 | 42 | 0.97 (0.96, 0.98) | 39 | 0.96 (0.95, 0.97) | -0.0139 | 0.9237 |
| ｐ15286 | 42 | 0.69 (0.68, 0.69) | 39 | 0.68 (0.67, 0.69) | -0.0141 | 0.9237 |
| ｐ16973 | 42 | 0.57 (0.56, 0.58) | 39 | 0.56 (0.55, 0.57) | -0.0234 | 0.9237 |
| ｐ19021 | 42 | 0.69 (0.68, 0.7) | 39 | 0.68 (0.67, 0.69) | -0.0237 | 0.9237 |
| ｐ15234 | 42 | 0.98 (0.96, 0.99) | 39 | 0.96 (0.95, 0.98) | -0.0243 | 0.9237 |
| ｐ15358 | 42 | 0.43 (0.42, 0.44) | 39 | 0.42 (0.41, 0.43) | -0.0247 | 0.9237 |
| ｐ15317 | 42 | 0.57 (0.56, 0.57) | 39 | 0.56 (0.55, 0.56) | -0.0268 | 0.9237 |
| ｐ15321 | 42 | 0.37 (0.36, 0.38) | 39 | 0.36 (0.36, 0.37) | -0.0290 | 0.9237 |
| ｐ15305 | 42 | 0.58 (0.57, 0.6) | 39 | 0.57 (0.56, 0.58) | -0.0307 | 0.9237 |
| ｐ16601 | 42 | 0.61 (0.6, 0.62) | 39 | 0.6 (0.59, 0.61) | -0.0316 | 0.9237 |
| ｐ18488 | 42 | 0.56 (0.55, 0.57) | 39 | 0.55 (0.53, 0.56) | -0.0358 | 0.9237 |
| ｐ16992 | 42 | 0.71 (0.69, 0.72) | 39 | 0.69 (0.67, 0.71) | -0.0395 | 0.9237 |
| ｐ18184 | 42 | 0.97 (0.94, 0.99) | 39 | 0.94 (0.91, 0.96) | -0.0403 | 0.9237 |
| ｐ16964 | 42 | 0.9 (0.87, 0.92) | 39 | 0.87 (0.84, 0.89) | -0.0492 | 0.9237 |
| ｐ15238 | 42 | 0.78 (0.76, 0.81) | 39 | 0.76 (0.73, 0.78) | -0.0499 | 0.9237 |
| ｐ15274 | 42 | 1.48 (1.43, 1.52) | 39 | 1.42 (1.37, 1.47) | -0.0556 | 0.9237 |
| ｐ16115 | 42 | 1.08 (1.04, 1.12) | 39 | 1.04 (1, 1.08) | -0.0566 | 0.9237 |
| ｐ15114 | 42 | 0.62 (0.6, 0.64) | 39 | 0.59 (0.57, 0.62) | -0.0576 | 0.9237 |
| ｐ15288 | 42 | 0.37 (0.36, 0.38) | 39 | 0.36 (0.34, 0.37) | -0.0580 | 0.9237 |
| ｐ15194 | 42 | 0.74 (0.72, 0.77) | 39 | 0.71 (0.68, 0.74) | -0.0625 | 0.9237 |
| ｐ15311 | 42 | 0.52 (0.49, 0.54) | 39 | 0.49 (0.47, 0.51) | -0.0777 | 0.9237 |
| ｐ15793 | 42 | 0.73 (0.69, 0.76) | 39 | 0.69 (0.65, 0.72) | -0.0788 | 0.9237 |
| ｐ15165 | 42 | 0.41 (0.39, 0.44) | 39 | 0.39 (0.37, 0.41) | -0.0819 | 0.9237 |
| ｐ15181 | 42 | 0.35 (0.33, 0.37) | 39 | 0.33 (0.31, 0.35) | -0.0836 | 0.9237 |
| ｐ19676 | 42 | 0.89 (0.85, 0.94) | 39 | 0.84 (0.8, 0.89) | -0.0856 | 0.9237 |
| ｐ15180 | 42 | 1.82 (1.73, 1.92) | 39 | 1.71 (1.61, 1.81) | -0.0918 | 0.9237 |
| ｐ15256 | 42 | 0.55 (0.51, 0.58) | 39 | 0.51 (0.48, 0.54) | -0.1000 | 0.9237 |
| ｐ15251 | 42 | 0.45 (0.42, 0.48) | 39 | 0.42 (0.39, 0.45) | -0.1037 | 0.9237 |
| ｐ15261 | 42 | 0.34 (0.32, 0.36) | 39 | 0.32 (0.29, 0.34) | -0.1066 | 0.9237 |
| ｐ16354 | 42 | 0.59 (0.55, 0.63) | 39 | 0.54 (0.5, 0.58) | -0.1169 | 0.9237 |
| ｐ15272 | 42 | 0.59 (0.55, 0.63) | 39 | 0.54 (0.5, 0.58) | -0.1194 | 0.9237 |
| ｐ15196 | 42 | 0.31 (0.29, 0.34) | 39 | 0.29 (0.27, 0.31) | -0.1197 | 0.9237 |
| ｐ17854 | 42 | 0.68 (0.63, 0.72) | 39 | 0.62 (0.57, 0.67) | -0.1208 | 0.9237 |
| ｐ18402 | 42 | 0.43 (0.4, 0.46) | 39 | 0.4 (0.37, 0.43) | -0.1219 | 0.9237 |
| ｐ15249 | 42 | 0.42 (0.39, 0.45) | 39 | 0.39 (0.35, 0.42) | -0.1236 | 0.9237 |
| ｐ16972 | 42 | 0.54 (0.5, 0.58) | 39 | 0.49 (0.45, 0.53) | -0.1256 | 0.9237 |
| ｐ18784 | 42 | 0.62 (0.58, 0.67) | 39 | 0.57 (0.52, 0.62) | -0.1329 | 0.9237 |
| ｐ15228 | 42 | 0.73 (0.67, 0.78) | 39 | 0.66 (0.6, 0.72) | -0.1341 | 0.9237 |
| ｐ15232 | 42 | 0.32 (0.29, 0.35) | 39 | 0.29 (0.26, 0.32) | -0.1389 | 0.9237 |
| ｐ15009 | 42 | 0.52 (0.48, 0.57) | 39 | 0.47 (0.43, 0.52) | -0.1469 | 0.9237 |
| ｐ18037 | 42 | 0.7 (0.64, 0.76) | 39 | 0.63 (0.57, 0.69) | -0.1477 | 0.9237 |
| ｐ17220 | 42 | 0.25 (0.23, 0.27) | 39 | 0.23 (0.2, 0.25) | -0.1501 | 0.9237 |
| ｐ15792 | 42 | 0.75 (0.68, 0.81) | 39 | 0.67 (0.6, 0.74) | -0.1503 | 0.9237 |
| ｐ16225 | 42 | 0.49 (0.44, 0.53) | 39 | 0.44 (0.39, 0.48) | -0.1575 | 0.9237 |
| ｐ16058 | 42 | 0.47 (0.42, 0.51) | 39 | 0.42 (0.37, 0.46) | -0.1577 | 0.9237 |
| ｐ15206 | 42 | 0.93 (0.84, 1.01) | 39 | 0.83 (0.74, 0.92) | -0.1602 | 0.9237 |
| ｐ15291 | 42 | 0.29 (0.26, 0.31) | 39 | 0.26 (0.23, 0.28) | -0.1680 | 0.9237 |
| ｐ15318 | 42 | 0.22 (0.2, 0.24) | 39 | 0.2 (0.18, 0.22) | -0.1689 | 0.9237 |
| ｐ18205 | 42 | 0.52 (0.47, 0.57) | 39 | 0.46 (0.4, 0.51) | -0.1802 | 0.9237 |
| ｐ17452 | 42 | 0.99 (0.88, 1.09) | 39 | 0.87 (0.76, 0.98) | -0.1863 | 0.9237 |
| ｐ19098 | 42 | 0.99 (0.88, 1.09) | 39 | 0.87 (0.76, 0.98) | -0.1863 | 0.9237 |
| ｐ19302 | 42 | 0.3 (0.26, 0.33) | 39 | 0.26 (0.22, 0.29) | -0.1952 | 0.9237 |
| ｐ15384 | 42 | 0.19 (0.17, 0.22) | 39 | 0.17 (0.15, 0.19) | -0.1969 | 0.9237 |
| ｐ15208 | 42 | 0.44 (0.39, 0.49) | 39 | 0.38 (0.33, 0.44) | -0.2086 | 0.9237 |
| ｐ17759 | 42 | 0.27 (0.24, 0.3) | 39 | 0.23 (0.2, 0.27) | -0.2086 | 0.9237 |
| ｐ15240 | 42 | 2.44 (2.15, 2.73) | 39 | 2.11 (1.81, 2.41) | -0.2090 | 0.9237 |
| ｐ15301 | 42 | 0.17 (0.14, 0.19) | 39 | 0.14 (0.12, 0.16) | -0.2293 | 0.9237 |
| ｐ15295 | 42 | 0.38 (0.33, 0.43) | 39 | 0.32 (0.27, 0.37) | -0.2384 | 0.9237 |
| ｐ17902 | 42 | 0.33 (0.29, 0.38) | 39 | 0.28 (0.23, 0.33) | -0.2442 | 0.9237 |
| ｐ15247 | 42 | 0.33 (0.29, 0.38) | 39 | 0.28 (0.24, 0.33) | -0.2450 | 0.9237 |
| ｐ15203 | 42 | 0.87 (0.75, 1) | 39 | 0.73 (0.61, 0.86) | -0.2502 | 0.9237 |
| ｐ15275 | 42 | 0.24 (0.21, 0.28) | 39 | 0.2 (0.17, 0.24) | -0.2557 | 0.9237 |
| ｐ15210 | 42 | 0.36 (0.31, 0.41) | 39 | 0.3 (0.25, 0.36) | -0.2573 | 0.9237 |
| ｐ16610 | 42 | 0.4 (0.34, 0.46) | 39 | 0.33 (0.27, 0.39) | -0.2613 | 0.9237 |
| ｐ15410 | 42 | 0.28 (0.24, 0.32) | 39 | 0.23 (0.19, 0.27) | -0.2621 | 0.9237 |
| ｐ15269 | 42 | 0.45 (0.38, 0.52) | 39 | 0.37 (0.3, 0.45) | -0.2718 | 0.9237 |
| ｐ15225 | 42 | 0.21 (0.18, 0.24) | 39 | 0.17 (0.14, 0.21) | -0.2726 | 0.9237 |
| ｐ17645 | 42 | 0.47 (0.4, 0.54) | 39 | 0.39 (0.31, 0.46) | -0.2822 | 0.9237 |
| ｐ15253 | 42 | 0.74 (0.63, 0.86) | 39 | 0.61 (0.49, 0.73) | -0.2901 | 0.9237 |
| ｐ15290 | 42 | 0.16 (0.13, 0.19) | 39 | 0.13 (0.1, 0.16) | -0.3059 | 0.9237 |
| ｐ15186 | 42 | 0.29 (0.24, 0.34) | 39 | 0.23 (0.18, 0.29) | -0.3193 | 0.9237 |
| ｐ15255 | 42 | 0.24 (0.19, 0.29) | 39 | 0.19 (0.14, 0.24) | -0.3815 | 0.9237 |
| ｐ15287 | 42 | 0.27 (0.22, 0.33) | 39 | 0.21 (0.15, 0.27) | -0.3825 | 0.9237 |
| ｐ15189 | 42 | 0.51 (0.4, 0.62) | 39 | 0.39 (0.28, 0.5) | -0.3944 | 0.9237 |
| ｐ17187 | 42 | 0.02 (0.02, 0.03) | 39 | 0.02 (0.01, 0.02) | -0.4190 | 0.9237 |
| ｐ15197 | 42 | 0.2 (0.16, 0.25) | 39 | 0.15 (0.1, 0.2) | -0.4450 | 0.9237 |
| ｐ15850 | 42 | 0.17 (0.13, 0.21) | 39 | 0.13 (0.08, 0.17) | -0.4586 | 0.9237 |
| ｐ15299 | 42 | 0.44 (0.33, 0.55) | 39 | 0.32 (0.21, 0.43) | -0.4623 | 0.9237 |
| ｐ15222 | 42 | 0.18 (0.13, 0.22) | 39 | 0.13 (0.08, 0.17) | -0.4665 | 0.9237 |
| ｐ15324 | 42 | 0.03 (0.02, 0.03) | 39 | 0.02 (0.01, 0.03) | -0.4771 | 0.9237 |
| ｐ15179 | 42 | 0.25 (0.18, 0.32) | 39 | 0.17 (0.1, 0.24) | -0.5388 | 0.9237 |
| ｐ15226 | 42 | 0.18 (0.13, 0.23) | 39 | 0.12 (0.07, 0.17) | -0.5502 | 0.9237 |
| ｐ15336 | 42 | 0.17 (0.13, 0.22) | 39 | 0.12 (0.07, 0.17) | -0.5538 | 0.9237 |
| ｐ15285 | 42 | 0.25 (0.17, 0.32) | 39 | 0.16 (0.09, 0.24) | -0.6027 | 0.9237 |
| ｐ15252 | 42 | 0.16 (0.1, 0.21) | 39 | 0.1 (0.04, 0.15) | -0.7042 | 0.9237 |
| ｐ15316 | 42 | 0.13 (0.09, 0.18) | 39 | 0.08 (0.03, 0.13) | -0.7197 | 0.9237 |
| ｐ15676 | 42 | 0.13 (0.08, 0.17) | 39 | 0.07 (0.02, 0.12) | -0.8016 | 0.9237 |
| ｐ15956 | 42 | 0.16 (0.1, 0.23) | 39 | 0.09 (0.03, 0.16) | -0.8247 | 0.9237 |
| ｐ15230 | 42 | 0.11 (0.07, 0.15) | 39 | 0.06 (0.02, 0.11) | -0.8305 | 0.9237 |
| ｐ17190 | 42 | 0.19 (0.11, 0.27) | 39 | 0.1 (0.02, 0.18) | -0.9060 | 0.9237 |
| ｐ17399 | 42 | 0.13 (0.07, 0.2) | 39 | 0.06 (-0.01, 0.13) | -1.1748 | 0.9237 |
| ｐ18800 | 42 | 0.11 (0.06, 0.17) | 39 | 0.05 (-0.01, 0.11) | -1.1918 | 0.9237 |
| ｐ15200 | 42 | 0.11 (0.06, 0.17) | 39 | 0.05 (-0.01, 0.1) | -1.2157 | 0.9237 |
| ｐ18220 | 42 | 0.19 (0.08, 0.3) | 39 | 0.07 (-0.05, 0.18) | -1.5119 | 0.9237 |
| ｐ19981 | 42 | 0.05 (0.02, 0.09) | 39 | 0.02 (-0.01, 0.05) | -1.5359 | 0.9237 |
| ｐ17539 | 42 | 0.08 (0.03, 0.13) | 39 | 0.02 (-0.04, 0.07) | -2.1385 | 0.9237 |
| ｐ15217 | 42 | 0.05 (0.02, 0.08) | 39 | 0.01 (-0.02, 0.05) | -2.1476 | 0.9237 |
| ｐ19039 | 42 | 0.18 (0.06, 0.31) | 39 | 0.04 (-0.09, 0.17) | -2.2042 | 0.9237 |
| ｐ15606 | 42 | 0.25 (0.07, 0.43) | 39 | 0.05 (-0.14, 0.23) | -2.3761 | 0.9237 |
| ｐ19737 | 42 | 0.07 (0.02, 0.12) | 39 | 0.01 (-0.04, 0.06) | -2.4767 | 0.9237 |
| ｐ15233 | 42 | 0.09 (0.02, 0.15) | 39 | 0.02 (-0.05, 0.08) | -2.5174 | 0.9237 |
| ｐ17424 | 42 | 0.05 (0.01, 0.09) | 39 | 0.01 (-0.03, 0.05) | -2.6986 | 0.9237 |
| ｐ18370 | 42 | 0.05 (0.01, 0.09) | 39 | 0.01 (-0.03, 0.05) | -2.6986 | 0.9237 |
| ｐ22783 | 42 | 0.49 (0.35, 0.64) | 39 | 0.66 (0.51, 0.8) | 0.4098 | 0.9237 |
| ｐ22473 | 42 | 1.29 (1.28, 1.3) | 39 | 1.28 (1.27, 1.29) | -0.0116 | 0.9235 |
| ｐ10387 | 42 | 0.63 (0.62, 0.64) | 39 | 0.62 (0.61, 0.63) | -0.0245 | 0.9235 |
| ｐ12037 | 42 | 0.38 (0.38, 0.39) | 39 | 0.38 (0.37, 0.38) | -0.0320 | 0.9235 |
| ｐ7120 | 42 | 0.52 (0.48, 0.56) | 39 | 0.47 (0.43, 0.52) | -0.1444 | 0.9235 |
| ｐ20454 | 42 | 0.57 (0.54, 0.61) | 39 | 0.53 (0.49, 0.57) | -0.1091 | 0.9235 |
| ｐ22543 | 42 | 0.37 (0.37, 0.38) | 39 | 0.37 (0.36, 0.37) | -0.0275 | 0.9235 |
| ｐ21250 | 42 | 0.62 (0.6, 0.65) | 39 | 0.6 (0.57, 0.62) | -0.0615 | 0.9235 |
| ｐ13235 | 42 | 0.56 (0.54, 0.59) | 39 | 0.54 (0.52, 0.56) | -0.0659 | 0.9235 |
| ｐ14504 | 42 | 0.1 (0.05, 0.14) | 39 | 0.05 (0.01, 0.09) | -0.9814 | 0.9235 |
| ｐ14453 | 42 | 1.98 (1.91, 2.06) | 39 | 1.9 (1.82, 1.97) | -0.0635 | 0.9235 |
| ｐ13183 | 42 | 1.2 (1.17, 1.23) | 39 | 1.16 (1.13, 1.19) | -0.0420 | 0.9235 |
| ｐ22562 | 42 | 0.72 (0.69, 0.74) | 39 | 0.69 (0.66, 0.71) | -0.0562 | 0.9235 |
| ｐ14919 | 42 | 0.6 (0.58, 0.62) | 39 | 0.57 (0.55, 0.6) | -0.0668 | 0.9235 |
| ｐ13058 | 42 | 0.64 (0.63, 0.64) | 39 | 0.63 (0.62, 0.64) | -0.0235 | 0.9235 |
| ｐ10332 | 42 | 0.53 (0.52, 0.54) | 39 | 0.52 (0.51, 0.53) | -0.0294 | 0.9235 |
| ｐ14875 | 42 | 0.52 (0.51, 0.53) | 39 | 0.5 (0.49, 0.52) | -0.0425 | 0.9235 |
| ｐ11090 | 42 | 0.89 (0.86, 0.92) | 39 | 0.85 (0.82, 0.88) | -0.0539 | 0.9235 |
| ｐ22527 | 42 | 0.6 (0.56, 0.65) | 39 | 0.55 (0.5, 0.6) | -0.1373 | 0.9235 |
| ｐ14004 | 42 | 0.4 (0.36, 0.43) | 39 | 0.36 (0.32, 0.39) | -0.1468 | 0.9235 |
| ｐ12495 | 42 | 0.2 (0.18, 0.22) | 39 | 0.17 (0.15, 0.2) | -0.2052 | 0.9235 |
| ｐ20207 | 42 | 0.23 (0.19, 0.27) | 39 | 0.18 (0.15, 0.22) | -0.3029 | 0.9235 |
| ｐ13834 | 42 | 0.12 (0.09, 0.15) | 39 | 0.09 (0.05, 0.12) | -0.4809 | 0.9235 |
| ｐ20634 | 42 | 0.53 (0.4, 0.66) | 39 | 0.38 (0.24, 0.52) | -0.4841 | 0.9235 |
| ｐ21927 | 42 | 0.26 (0.07, 0.46) | 39 | 0.04 (-0.16, 0.24) | -2.6165 | 0.9235 |
| ｐ3582 | 42 | 0.58 (0.55, 0.6) | 39 | 0.55 (0.53, 0.57) | -0.0624 | 0.9235 |
| ｐ22532 | 42 | 0.62 (0.58, 0.66) | 39 | 0.58 (0.53, 0.62) | -0.1071 | 0.9235 |
| ｐ12194 | 42 | 0.38 (0.35, 0.4) | 39 | 0.35 (0.32, 0.38) | -0.1120 | 0.9235 |
| ｐ10706 | 42 | 0.36 (0.31, 0.4) | 39 | 0.3 (0.25, 0.35) | -0.2322 | 0.9235 |
| ｐ20751 | 42 | 0.2 (0.12, 0.29) | 39 | 0.1 (0.01, 0.2) | -0.9839 | 0.9235 |
| ｐ20315 | 42 | 0.91 (0.86, 0.95) | 39 | 0.85 (0.81, 0.9) | -0.0825 | 0.9235 |
| ｐ13420 | 42 | 0.19 (0.16, 0.22) | 39 | 0.16 (0.13, 0.19) | -0.2811 | 0.9235 |
| ｐ4765 | 42 | 0.19 (0.14, 0.23) | 39 | 0.13 (0.09, 0.18) | -0.4715 | 0.9235 |
| ｐ14715 | 42 | 0.12 (0.08, 0.17) | 39 | 0.07 (0.02, 0.12) | -0.8090 | 0.9235 |
| ｐ14770 | 42 | 0.18 (0.08, 0.28) | 39 | 0.07 (-0.03, 0.17) | -1.3682 | 0.9235 |
| ｐ20716 | 42 | 0.08 (0.04, 0.13) | 39 | 0.03 (-0.02, 0.08) | -1.5368 | 0.9235 |
| ｐ20401 | 42 | 0.67 (0.65, 0.68) | 39 | 0.65 (0.63, 0.67) | -0.0431 | 0.9235 |
| ｐ21011 | 42 | 1.29 (1.23, 1.34) | 39 | 1.23 (1.17, 1.28) | -0.0687 | 0.9235 |
| ｐ12551 | 42 | 0.46 (0.43, 0.49) | 39 | 0.43 (0.39, 0.46) | -0.1110 | 0.9235 |
| ｐ11472 | 42 | 0.74 (0.69, 0.79) | 39 | 0.69 (0.64, 0.74) | -0.1125 | 0.9235 |
| ｐ10520 | 42 | 0.4 (0.36, 0.44) | 39 | 0.35 (0.31, 0.39) | -0.1816 | 0.9235 |
| ｐ6506 | 42 | 0.53 (0.46, 0.6) | 39 | 0.45 (0.37, 0.52) | -0.2452 | 0.9235 |
| ｐ22283 | 42 | 0.71 (0.7, 0.71) | 39 | 0.7 (0.69, 0.7) | -0.0153 | 0.9235 |
| ｐ11468 | 42 | 0.74 (0.74, 0.75) | 39 | 0.73 (0.73, 0.74) | -0.0187 | 0.9235 |
| ｐ11755 | 42 | 1.63 (1.52, 1.74) | 39 | 1.5 (1.39, 1.62) | -0.1140 | 0.9235 |
| ｐ6445 | 42 | 0.48 (0.44, 0.51) | 39 | 0.44 (0.41, 0.47) | -0.1142 | 0.9235 |
| ｐ22929 | 42 | 0.17 (0.14, 0.19) | 39 | 0.14 (0.12, 0.16) | -0.2360 | 0.9235 |
| ｐ14806 | 42 | 0.44 (0.39, 0.5) | 39 | 0.38 (0.32, 0.44) | -0.2373 | 0.9235 |
| ｐ14847 | 42 | 0.23 (0.19, 0.28) | 39 | 0.19 (0.14, 0.23) | -0.3399 | 0.9235 |
| ｐ12346 | 42 | 0.23 (0.15, 0.31) | 39 | 0.14 (0.05, 0.22) | -0.7574 | 0.9235 |
| ｐ20806 | 42 | 0.05 (0.01, 0.09) | 39 | 0.01 (-0.03, 0.05) | -2.6987 | 0.9235 |
| ｐ20953 | 42 | 0.49 (0.46, 0.52) | 39 | 0.46 (0.43, 0.49) | -0.1009 | 0.9235 |
| ｐ12017 | 42 | 0.82 (0.77, 0.87) | 39 | 0.76 (0.7, 0.81) | -0.1072 | 0.9235 |
| ｐ22477 | 42 | 0.13 (0.07, 0.19) | 39 | 0.06 (0, 0.13) | -1.0310 | 0.9235 |
| ｐ12695 | 42 | 0.44 (0.4, 0.48) | 39 | 0.39 (0.35, 0.44) | -0.1584 | 0.9235 |
| ｐ20417 | 42 | 0.51 (0.44, 0.58) | 39 | 0.43 (0.36, 0.5) | -0.2453 | 0.9235 |
| ｐ22332 | 42 | 0.65 (0.56, 0.74) | 39 | 0.54 (0.45, 0.64) | -0.2577 | 0.9235 |
| ｐ20086 | 42 | 0.29 (0.26, 0.32) | 39 | 0.25 (0.22, 0.28) | -0.1928 | 0.9235 |
| ｐ22972 | 42 | 0.4 (0.34, 0.47) | 39 | 0.33 (0.27, 0.4) | -0.2737 | 0.9235 |
| ｐ2722 | 42 | 0.65 (0.62, 0.68) | 39 | 0.62 (0.59, 0.65) | -0.0753 | 0.9235 |
| ｐ1188 | 42 | 0.84 (0.77, 0.91) | 39 | 0.76 (0.69, 0.83) | -0.1383 | 0.9235 |
| ｐ14881 | 42 | 0.18 (0.15, 0.22) | 39 | 0.14 (0.1, 0.18) | -0.3771 | 0.9235 |
| ｐ12773 | 42 | 0.82 (0.79, 0.85) | 39 | 0.79 (0.76, 0.82) | -0.0564 | 0.9235 |
| ｐ13965 | 42 | 0.29 (0.26, 0.32) | 39 | 0.26 (0.23, 0.29) | -0.1812 | 0.9235 |
| ｐ20484 | 42 | 0.29 (0.26, 0.32) | 39 | 0.26 (0.23, 0.29) | -0.1812 | 0.9235 |
| ｐ14883 | 42 | 0.23 (0.19, 0.26) | 39 | 0.19 (0.15, 0.23) | -0.2743 | 0.9235 |
| ｐ11302 | 42 | 0.5 (0.49, 0.52) | 39 | 0.49 (0.47, 0.5) | -0.0451 | 0.9235 |
| ｐ22638 | 42 | 0.94 (0.91, 0.98) | 39 | 0.9 (0.86, 0.94) | -0.0637 | 0.9235 |
| ｐ1734 | 42 | 0.3 (0.27, 0.34) | 39 | 0.26 (0.23, 0.3) | -0.2098 | 0.9235 |
| ｐ13715 | 42 | 0.47 (0.45, 0.48) | 39 | 0.45 (0.44, 0.47) | -0.0500 | 0.9235 |
| ｐ22528 | 42 | 1.09 (1.07, 1.11) | 39 | 1.07 (1.05, 1.09) | -0.0254 | 0.9235 |
| ｐ14253 | 42 | 0.48 (0.47, 0.5) | 39 | 0.47 (0.45, 0.48) | -0.0450 | 0.9235 |
| ｐ6939 | 42 | 0.88 (0.87, 0.9) | 39 | 0.87 (0.85, 0.88) | -0.0240 | 0.9235 |
| ｐ15481 | 42 | 0.62 (0.51, 0.73) | 39 | 0.49 (0.38, 0.61) | -0.3316 | 0.9226 |
| ｐ15591 | 42 | 0.18 (0.01, 0.36) | 39 | 0.38 (0.2, 0.56) | 1.0437 | 0.9215 |
| ｐ17077 | 42 | 0.27 (0.16, 0.38) | 39 | 0.39 (0.28, 0.51) | 0.5595 | 0.9208 |
| ｐ8837 | 42 | 0.23 (0.17, 0.28) | 39 | 0.17 (0.11, 0.22) | -0.4463 | 0.9183 |
| ｐ8465 | 42 | 0.56 (0.36, 0.76) | 39 | 0.79 (0.58, 1) | 0.4873 | 0.9172 |
| ｐ16295 | 42 | 0.29 (0.04, 0.53) | 39 | 0.57 (0.31, 0.82) | 0.9873 | 0.9169 |
| ｐ22938 | 42 | 0.6 (0.43, 0.76) | 39 | 0.79 (0.61, 0.96) | 0.3987 | 0.9168 |
| ｐ20105 | 42 | 0.32 (0.25, 0.39) | 39 | 0.24 (0.17, 0.31) | -0.4048 | 0.9159 |
| ｐ14259 | 42 | 0.26 (0.21, 0.31) | 39 | 0.2 (0.15, 0.25) | -0.3419 | 0.9130 |
| ｐ14824 | 42 | 0.25 (0.11, 0.4) | 39 | 0.42 (0.27, 0.57) | 0.7202 | 0.9129 |
| ｐ12560 | 42 | 0.39 (0.32, 0.45) | 39 | 0.31 (0.24, 0.38) | -0.3221 | 0.9105 |
| ｐ22773 | 42 | 0.8 (0.48, 1.11) | 39 | 1.16 (0.83, 1.49) | 0.5371 | 0.9102 |
| ｐ22948 | 42 | 0.38 (0.32, 0.45) | 39 | 0.46 (0.39, 0.53) | 0.2555 | 0.9097 |
| ｐ20584 | 42 | 0.31 (0.22, 0.41) | 39 | 0.2 (0.1, 0.31) | -0.6285 | 0.9096 |
| ｐ21409 | 42 | 0.53 (0.51, 0.56) | 39 | 0.56 (0.53, 0.58) | 0.0692 | 0.9064 |
| ｐ22709 | 42 | 0.38 (0.17, 0.59) | 39 | 0.61 (0.4, 0.83) | 0.6879 | 0.9063 |
| ｐ10288 | 42 | 0.41 (0.3, 0.53) | 39 | 0.29 (0.17, 0.4) | -0.5304 | 0.9057 |
| ｐ12455 | 42 | 0.29 (0.19, 0.39) | 39 | 0.18 (0.07, 0.28) | -0.7060 | 0.9057 |
| ｐ21843 | 42 | 0.29 (0.19, 0.39) | 39 | 0.18 (0.07, 0.28) | -0.7060 | 0.9057 |
| ｐ4405 | 42 | 0.47 (0.24, 0.69) | 39 | 0.72 (0.49, 0.96) | 0.6322 | 0.9050 |
| ｐ17309 | 42 | 0.34 (0.25, 0.43) | 39 | 0.44 (0.34, 0.53) | 0.3767 | 0.9024 |
| ｐ13085 | 42 | 0.45 (0.25, 0.66) | 39 | 0.68 (0.47, 0.9) | 0.5925 | 0.8997 |
| ｐ18791 | 42 | 0.06 (0.03, 0.09) | 39 | 0.03 (0, 0.06) | -0.9405 | 0.8989 |
| ｐ797 | 42 | 0.35 (0.19, 0.51) | 39 | 0.53 (0.36, 0.69) | 0.5975 | 0.8960 |
| ｐ21356 | 42 | 0.32 (0.2, 0.43) | 39 | 0.45 (0.33, 0.57) | 0.5016 | 0.8948 |
| ｐ13644 | 42 | 0.32 (0.25, 0.39) | 39 | 0.39 (0.32, 0.46) | 0.3076 | 0.8940 |
| ｐ14746 | 39 | 0.23 (-0.09, 0.55) | 39 | 0.58 (0.26, 0.9) | 1.3350 | 0.8930 |
| ｐ22041 | 42 | 0.2 (0.16, 0.25) | 39 | 0.25 (0.21, 0.29) | 0.2996 | 0.8919 |
| ｐ12333 | 42 | 0.29 (0.12, 0.46) | 39 | 0.48 (0.31, 0.66) | 0.7194 | 0.8911 |
| ｐ15958 | 42 | 0.42 (0.28, 0.56) | 39 | 0.58 (0.43, 0.73) | 0.4638 | 0.8908 |
| ｐ14237 | 42 | 0.39 (0.23, 0.54) | 39 | 0.56 (0.4, 0.73) | 0.5384 | 0.8891 |
| ｐ15038 | 42 | 0.24 (0.17, 0.3) | 39 | 0.31 (0.24, 0.37) | 0.3723 | 0.8887 |
| ｐ16513 | 42 | 0.04 (-0.07, 0.15) | 39 | 0.17 (0.05, 0.29) | 2.0579 | 0.8874 |
| ｐ21653 | 42 | 0.04 (-0.07, 0.15) | 39 | 0.17 (0.05, 0.29) | 2.0579 | 0.8874 |
| ｐ15752 | 42 | 0.37 (0.3, 0.44) | 39 | 0.3 (0.23, 0.37) | -0.3231 | 0.8867 |
| ｐ22022 | 42 | 0.46 (0.14, 0.78) | 39 | 0.82 (0.48, 1.15) | 0.8217 | 0.8864 |
| ｐ20792 | 42 | 0.14 (0.1, 0.18) | 39 | 0.1 (0.06, 0.14) | -0.5019 | 0.8855 |
| ｐ19780 | 42 | 0.22 (0.2, 0.25) | 39 | 0.25 (0.23, 0.28) | 0.1723 | 0.8854 |
| ｐ14317 | 42 | 0.26 (0.2, 0.32) | 39 | 0.19 (0.13, 0.26) | -0.4240 | 0.8851 |
| ｐ15515 | 42 | 0.87 (0.73, 1.02) | 39 | 1.03 (0.88, 1.18) | 0.2401 | 0.8851 |
| ｐ22703 | 42 | 0.39 (0.17, 0.61) | 39 | 0.63 (0.4, 0.86) | 0.7002 | 0.8835 |
| ｐ14830 | 42 | 0.49 (0.34, 0.63) | 39 | 0.33 (0.18, 0.48) | -0.5759 | 0.8792 |
| ｐ21683 | 42 | 0.84 (0.76, 0.91) | 39 | 0.92 (0.84, 0.99) | 0.1315 | 0.8765 |
| ｐ962 | 42 | 0.47 (0.35, 0.58) | 39 | 0.34 (0.23, 0.46) | -0.4415 | 0.8758 |
| ｐ22143 | 42 | 0.47 (0.38, 0.55) | 39 | 0.38 (0.29, 0.46) | -0.3158 | 0.8747 |
| ｐ7098 | 42 | 0.19 (0.12, 0.26) | 39 | 0.12 (0.05, 0.19) | -0.7131 | 0.8744 |
| ｐ22744 | 42 | 0.61 (0.1, 1.13) | 39 | 1.18 (0.64, 1.72) | 0.9452 | 0.8734 |
| ｐ14217 | 42 | 0.76 (0.72, 0.8) | 39 | 0.8 (0.76, 0.84) | 0.0778 | 0.8717 |
| ｐ21259 | 42 | 1.61 (-5.24, 8.45) | 39 | 9.08 (1.98, 16.19) | 2.4976 | 0.8684 |
| ｐ4197 | 42 | 1.61 (-5.24, 8.45) | 39 | 9.08 (1.98, 16.19) | 2.4976 | 0.8684 |
| ｐ20822 | 42 | 0.8 (-0.1, 1.7) | 39 | 1.79 (0.85, 2.72) | 1.1595 | 0.8675 |
| ｐ22683 | 42 | 0.15 (-0.07, 0.37) | 39 | 0.39 (0.16, 0.62) | 1.3813 | 0.8674 |
| ｐ4915 | 42 | 0.62 (0.47, 0.76) | 39 | 0.78 (0.62, 0.93) | 0.3356 | 0.8659 |
| ｐ14265 | 42 | 0.44 (0.31, 0.57) | 39 | 0.3 (0.16, 0.43) | -0.5647 | 0.8658 |
| ｐ22675 | 42 | 0.19 (0.05, 0.33) | 39 | 0.34 (0.2, 0.48) | 0.8306 | 0.8656 |
| ｐ22741 | 42 | 0.43 (0.09, 0.76) | 39 | 0.79 (0.44, 1.14) | 0.8944 | 0.8649 |
| ｐ16714 | 42 | 0.35 (-0.6, 1.3) | 39 | 1.38 (0.39, 2.36) | 1.9870 | 0.8611 |
| ｐ18045 | 42 | 0.6 (0.13, 1.07) | 39 | 1.11 (0.62, 1.6) | 0.8914 | 0.8611 |
| ｐ10262 | 42 | 0.34 (0.27, 0.41) | 39 | 0.27 (0.2, 0.34) | -0.3480 | 0.8598 |
| ｐ16310 | 42 | 0.26 (0.17, 0.36) | 39 | 0.37 (0.27, 0.47) | 0.4818 | 0.8592 |
| ｐ19718 | 42 | 0.51 (0.42, 0.6) | 39 | 0.42 (0.32, 0.51) | -0.2994 | 0.8576 |
| ｐ22574 | 42 | 0.6 (-0.13, 1.33) | 39 | 1.39 (0.63, 2.15) | 1.2087 | 0.8548 |
| ｐ15573 | 42 | 0.3 (0, 0.6) | 39 | 0.63 (0.31, 0.94) | 1.0657 | 0.8533 |
| ｐ364 | 42 | 0.42 (0.27, 0.57) | 39 | 0.58 (0.43, 0.74) | 0.4684 | 0.8530 |
| ｐ22857 | 42 | 0.6 (0.57, 0.63) | 39 | 0.64 (0.6, 0.67) | 0.0813 | 0.8530 |
| ｐ22674 | 42 | 0 (-0.08, 0.08) | 39 | 0.09 (0, 0.17) | NA | 0.8529 |
| ｐ22942 | 42 | 0.63 (0.48, 0.77) | 39 | 0.78 (0.63, 0.94) | 0.3251 | 0.8521 |
| ｐ22957 | 42 | 0.52 (0.39, 0.65) | 39 | 0.66 (0.52, 0.79) | 0.3455 | 0.8516 |
| ｐ16728 | 42 | 0.58 (0.24, 0.91) | 39 | 0.94 (0.59, 1.29) | 0.7001 | 0.8508 |
| ｐ11120 | 42 | 1.85 (1.04, 2.66) | 39 | 0.98 (0.14, 1.82) | -0.9157 | 0.8502 |
| ｐ15244 | 42 | 0.5 (0.41, 0.58) | 39 | 0.41 (0.32, 0.49) | -0.2868 | 0.8492 |
| ｐ20379 | 42 | 0.34 (0.17, 0.51) | 39 | 0.53 (0.35, 0.71) | 0.6286 | 0.8492 |
| ｐ16873 | 42 | 0.03 (-0.51, 0.56) | 39 | 0.6 (0.04, 1.15) | 4.5732 | 0.8489 |
| ｐ20298 | 42 | 0.45 (0.4, 0.51) | 39 | 0.51 (0.45, 0.57) | 0.1768 | 0.8482 |
| ｐ15235 | 42 | 0.2 (0.16, 0.25) | 39 | 0.15 (0.11, 0.2) | -0.3924 | 0.8477 |
| ｐ11187 | 42 | 0.31 (0.2, 0.42) | 39 | 0.43 (0.32, 0.55) | 0.4582 | 0.8448 |
| ｐ16384 | 42 | 0.31 (0.2, 0.42) | 39 | 0.43 (0.32, 0.55) | 0.4582 | 0.8447 |
| ｐ15277 | 42 | 0.36 (0.28, 0.45) | 39 | 0.27 (0.18, 0.36) | -0.4260 | 0.8444 |
| ｐ15034 | 42 | 1.61 (1.3, 1.92) | 39 | 1.28 (0.95, 1.6) | -0.3337 | 0.8422 |
| ｐ15227 | 42 | 0.14 (0.1, 0.17) | 39 | 0.1 (0.07, 0.14) | -0.4328 | 0.8422 |
| ｐ22765 | 42 | 0.52 (0.36, 0.67) | 39 | 0.69 (0.52, 0.85) | 0.3987 | 0.8411 |
| ｐ22004 | 42 | 0.58 (0.4, 0.77) | 39 | 0.79 (0.59, 0.98) | 0.4263 | 0.8389 |
| ｐ14859 | 42 | 0.78 (0.6, 0.96) | 39 | 0.59 (0.4, 0.77) | -0.4067 | 0.8358 |
| ｐ15292 | 42 | 0.22 (0.17, 0.27) | 39 | 0.17 (0.12, 0.22) | -0.3891 | 0.8353 |
| ｐ21087 | 42 | 0.13 (0.1, 0.16) | 39 | 0.1 (0.06, 0.13) | -0.4312 | 0.8337 |
| ｐ10548 | 42 | 0.32 (0.26, 0.38) | 39 | 0.38 (0.32, 0.45) | 0.2675 | 0.8326 |
| ｐ21258 | 42 | 0.18 (0.14, 0.22) | 39 | 0.23 (0.18, 0.27) | 0.3267 | 0.8323 |
| ｐ15191 | 42 | 0.39 (0.29, 0.49) | 39 | 0.28 (0.17, 0.39) | -0.4789 | 0.8321 |
| ｐ22658 | 42 | 0.43 (0.37, 0.5) | 39 | 0.36 (0.29, 0.43) | -0.2554 | 0.8306 |
| ｐ17838 | 42 | 0.63 (0.49, 0.77) | 39 | 0.49 (0.34, 0.63) | -0.3797 | 0.8300 |
| ｐ19838 | 42 | 0.36 (0.19, 0.53) | 39 | 0.18 (0, 0.36) | -0.9988 | 0.8300 |
| ｐ21649 | 42 | 0.52 (0.36, 0.68) | 39 | 0.35 (0.19, 0.52) | -0.5668 | 0.8294 |
| ｐ20565 | 42 | 1.26 (1.02, 1.51) | 39 | 1 (0.75, 1.26) | -0.3330 | 0.8292 |
| ｐ15204 | 42 | 0.2 (0.16, 0.23) | 39 | 0.16 (0.12, 0.2) | -0.3015 | 0.8262 |
| ｐ2807 | 42 | 0.5 (0.2, 0.79) | 39 | 0.19 (-0.12, 0.49) | -1.4060 | 0.8261 |
| ｐ17897 | 42 | 1.2 (0.42, 1.99) | 39 | 2.03 (1.22, 2.85) | 0.7571 | 0.8259 |
| ｐ4643 | 42 | 0.51 (0.25, 0.76) | 39 | 0.78 (0.51, 1.04) | 0.6175 | 0.8258 |
| ｐ15449 | 42 | 0.48 (-0.06, 1.02) | 39 | 1.05 (0.49, 1.61) | 1.1313 | 0.8256 |
| ｐ15568 | 42 | 0.39 (0.12, 0.66) | 39 | 0.67 (0.39, 0.95) | 0.7941 | 0.8256 |
| ｐ20904 | 42 | 0.3 (0.18, 0.43) | 39 | 0.43 (0.3, 0.56) | 0.5267 | 0.8251 |
| ｐ16550 | 42 | 0.23 (0.19, 0.26) | 39 | 0.18 (0.14, 0.23) | -0.2891 | 0.8245 |
| ｐ14783 | 42 | 0.57 (0.11, 1.03) | 39 | 0.09 (-0.39, 0.57) | -2.6751 | 0.8224 |
| ｐ22031 | 42 | 0.76 (0.58, 0.94) | 39 | 0.95 (0.76, 1.13) | 0.3228 | 0.8222 |
| ｐ13608 | 42 | 0.16 (0.04, 0.29) | 39 | 0.3 (0.17, 0.43) | 0.8536 | 0.8222 |
| ｐ14735 | 42 | 0.28 (0.18, 0.38) | 39 | 0.38 (0.28, 0.49) | 0.4671 | 0.8219 |
| ｐ15963 | 42 | 0.38 (0.26, 0.49) | 39 | 0.49 (0.38, 0.61) | 0.3936 | 0.8216 |
| ｐ17765 | 42 | 0.15 (-0.03, 0.32) | 39 | 0.33 (0.15, 0.51) | 1.1784 | 0.8207 |
| ｐ17569 | 42 | 0.21 (0.15, 0.27) | 39 | 0.14 (0.08, 0.2) | -0.5300 | 0.8207 |
| ｐ10231 | 42 | 0.35 (0.21, 0.5) | 39 | 0.5 (0.36, 0.65) | 0.5048 | 0.8204 |
| ｐ22745 | 42 | 0.38 (0.23, 0.53) | 39 | 0.54 (0.39, 0.7) | 0.4954 | 0.8200 |
| ｐ15174 | 42 | 0.17 (0.13, 0.2) | 39 | 0.13 (0.09, 0.17) | -0.3856 | 0.8196 |
| ｐ22001 | 42 | 0.11 (0.02, 0.19) | 39 | 0.01 (-0.07, 0.1) | -2.8320 | 0.8182 |
| ｐ10878 | 42 | 0.61 (0.33, 0.89) | 39 | 0.9 (0.61, 1.19) | 0.5659 | 0.8182 |
| ｐ22552 | 42 | 0.14 (0.11, 0.18) | 39 | 0.18 (0.14, 0.22) | 0.3454 | 0.8178 |
| ｐ14999 | 42 | 0.29 (0.2, 0.38) | 39 | 0.19 (0.1, 0.29) | -0.5678 | 0.8170 |
| ｐ16564 | 42 | 0.32 (0.25, 0.39) | 39 | 0.39 (0.32, 0.46) | 0.2956 | 0.8164 |
| ｐ14805 | 42 | 0.42 (0.22, 0.61) | 39 | 0.62 (0.42, 0.82) | 0.5695 | 0.8160 |
| ｐ17079 | 42 | 0.41 (0.38, 0.45) | 39 | 0.45 (0.41, 0.48) | 0.1099 | 0.8156 |
| ｐ17192 | 42 | 0.37 (0.29, 0.46) | 39 | 0.28 (0.19, 0.37) | -0.4114 | 0.8156 |
| ｐ16189 | 42 | 0.55 (0.36, 0.73) | 39 | 0.36 (0.16, 0.55) | -0.6235 | 0.8150 |
| ｐ10746 | 42 | 0.12 (0.1, 0.14) | 39 | 0.1 (0.07, 0.12) | -0.3178 | 0.8147 |
| ｐ22784 | 42 | 0.36 (0.15, 0.56) | 39 | 0.57 (0.36, 0.78) | 0.6779 | 0.8140 |
| ｐ15995 | 42 | 0.33 (0.28, 0.39) | 39 | 0.28 (0.22, 0.33) | -0.2669 | 0.8139 |
| ｐ2288 | 42 | 0.22 (0.18, 0.26) | 39 | 0.18 (0.13, 0.22) | -0.3196 | 0.8132 |
| ｐ15661 | 42 | 0.22 (0.18, 0.26) | 39 | 0.18 (0.13, 0.22) | -0.3196 | 0.8130 |
| ｐ14741 | 42 | 0.18 (0.01, 0.35) | 39 | 0 (-0.18, 0.18) | NA | 0.8126 |
| ｐ22890 | 42 | 0.18 (0.14, 0.22) | 39 | 0.14 (0.1, 0.18) | -0.3778 | 0.8126 |
| ｐ17910 | 42 | 0.49 (0.37, 0.61) | 39 | 0.62 (0.49, 0.74) | 0.3222 | 0.8125 |
| ｐ15322 | 42 | 0.21 (0.17, 0.25) | 39 | 0.17 (0.12, 0.21) | -0.3388 | 0.8119 |
| ｐ19349 | 42 | 0.68 (0.63, 0.72) | 39 | 0.72 (0.68, 0.77) | 0.0957 | 0.8116 |
| ｐ19074 | 42 | 0.4 (0.34, 0.45) | 39 | 0.34 (0.29, 0.4) | -0.2133 | 0.8116 |
| ｐ148 | 42 | 0.54 (0.41, 0.67) | 39 | 0.41 (0.27, 0.54) | -0.4168 | 0.8115 |
| ｐ17881 | 42 | 0.37 (0.31, 0.43) | 39 | 0.31 (0.25, 0.37) | -0.2610 | 0.8114 |
| ｐ17642 | 42 | 0.15 (0.1, 0.21) | 39 | 0.1 (0.04, 0.15) | -0.6653 | 0.8097 |
| ｐ16546 | 42 | 0.07 (-0.84, 0.99) | 39 | 1.03 (0.07, 1.98) | 3.8060 | 0.8094 |
| ｐ4360 | 42 | 1.02 (0.83, 1.22) | 39 | 0.82 (0.62, 1.02) | -0.3179 | 0.8092 |
| ｐ19525 | 42 | 0.66 (0.58, 0.74) | 39 | 0.57 (0.49, 0.66) | -0.2029 | 0.8091 |
| ｐ15323 | 42 | 0.15 (0.11, 0.2) | 39 | 0.11 (0.06, 0.15) | -0.5350 | 0.8085 |
| ｐ18905 | 42 | 0.37 (0.06, 0.67) | 39 | 0.05 (-0.26, 0.37) | -2.7775 | 0.8085 |
| ｐ21266 | 42 | 0.41 (0.31, 0.52) | 39 | 0.53 (0.41, 0.64) | 0.3478 | 0.8082 |
| ｐ16508 | 42 | 0.77 (0.66, 0.89) | 39 | 0.65 (0.53, 0.77) | -0.2444 | 0.8080 |
| ｐ22697 | 42 | 0.06 (0.01, 0.1) | 39 | 0.11 (0.06, 0.16) | 0.9204 | 0.8080 |
| ｐ15443 | 42 | 0.41 (0.34, 0.49) | 39 | 0.49 (0.41, 0.57) | 0.2521 | 0.8074 |
| ｐ14936 | 42 | 0.38 (0.31, 0.45) | 39 | 0.31 (0.24, 0.38) | -0.3007 | 0.8055 |
| ｐ17301 | 42 | 0.43 (0.37, 0.49) | 39 | 0.37 (0.31, 0.43) | -0.2188 | 0.8052 |
| ｐ15042 | 42 | 0.37 (0.31, 0.44) | 39 | 0.3 (0.24, 0.37) | -0.2875 | 0.8049 |
| ｐ22092 | 42 | 0 (-0.1, 0.1) | 39 | 0.11 (0, 0.21) | NA | 0.8041 |
| ｐ22680 | 42 | 0.64 (0.55, 0.72) | 39 | 0.72 (0.63, 0.81) | 0.1819 | 0.8040 |
| ｐ10001 | 42 | 0.63 (0.5, 0.75) | 39 | 0.49 (0.36, 0.63) | -0.3387 | 0.8039 |
| ｐ18954 | 42 | 0.34 (0.27, 0.4) | 39 | 0.27 (0.2, 0.34) | -0.3170 | 0.8038 |
| ｐ15229 | 42 | 0.09 (0.05, 0.13) | 39 | 0.05 (0.02, 0.09) | -0.7733 | 0.8022 |
| ｐ22513 | 42 | 0.37 (0.3, 0.44) | 39 | 0.3 (0.22, 0.37) | -0.3205 | 0.8012 |
| ｐ19735 | 42 | 1.31 (1.26, 1.37) | 39 | 1.25 (1.2, 1.31) | -0.0656 | 0.8002 |
| ｐ14767 | 42 | 0.84 (0.74, 0.94) | 39 | 0.74 (0.63, 0.84) | -0.1944 | 0.8001 |
| ｐ22334 | 42 | 0.81 (0.73, 0.89) | 39 | 0.9 (0.81, 0.98) | 0.1401 | 0.7998 |
| ｐ18354 | 42 | 0.38 (0.26, 0.49) | 39 | 0.26 (0.14, 0.38) | -0.5356 | 0.7989 |
| ｐ15198 | 42 | 0.34 (0.23, 0.44) | 39 | 0.23 (0.12, 0.34) | -0.5625 | 0.7986 |
| ｐ22657 | 42 | 0.23 (0.14, 0.32) | 39 | 0.14 (0.04, 0.23) | -0.7502 | 0.7979 |
| ｐ17979 | 42 | 0.22 (0.13, 0.31) | 39 | 0.31 (0.22, 0.41) | 0.5067 | 0.7967 |
| ｐ22048 | 42 | 0.33 (0.31, 0.35) | 39 | 0.35 (0.33, 0.37) | 0.0960 | 0.7962 |
| ｐ15887 | 42 | 0.21 (0.15, 0.26) | 39 | 0.15 (0.09, 0.21) | -0.4965 | 0.7951 |
| ｐ20858 | 42 | 0.3 (0.24, 0.36) | 39 | 0.24 (0.17, 0.3) | -0.3431 | 0.7949 |
| ｐ19151 | 42 | 0.33 (0.25, 0.42) | 39 | 0.25 (0.16, 0.34) | -0.4363 | 0.7924 |
| ｐ13538 | 42 | 0.33 (0.25, 0.42) | 39 | 0.25 (0.16, 0.34) | -0.4363 | 0.7923 |
| ｐ20037 | 42 | 0.61 (0.57, 0.66) | 39 | 0.66 (0.61, 0.71) | 0.1067 | 0.7913 |
| ｐ36 | 42 | 0.38 (0.12, 0.63) | 39 | 0.64 (0.38, 0.9) | 0.7544 | 0.7912 |
| ｐ15211 | 42 | 0.37 (0.21, 0.54) | 39 | 0.2 (0.03, 0.37) | -0.8796 | 0.7899 |
| ｐ11545 | 42 | 0.36 (0.28, 0.44) | 39 | 0.28 (0.2, 0.36) | -0.3517 | 0.7899 |
| ｐ204 | 42 | 0.45 (0.33, 0.58) | 39 | 0.33 (0.2, 0.46) | -0.4761 | 0.7896 |
| ｐ12422 | 42 | 0.19 (0.13, 0.26) | 39 | 0.12 (0.06, 0.19) | -0.6309 | 0.7881 |
| ｐ15271 | 42 | 0.44 (0.37, 0.52) | 39 | 0.37 (0.29, 0.44) | -0.2769 | 0.7878 |
| ｐ14377 | 42 | 0.21 (0.12, 0.3) | 39 | 0.3 (0.21, 0.39) | 0.5087 | 0.7876 |
| ｐ22734 | 42 | 0 (-0.26, 0.26) | 39 | 0.26 (-0.01, 0.53) | NA | 0.7861 |
| ｐ15914 | 42 | 0.33 (0.22, 0.43) | 39 | 0.43 (0.32, 0.54) | 0.4134 | 0.7849 |
| ｐ776 | 42 | 1.86 (1.54, 2.19) | 39 | 1.53 (1.19, 1.87) | -0.2817 | 0.7843 |
| ｐ15254 | 42 | 0.36 (0.3, 0.42) | 39 | 0.3 (0.23, 0.36) | -0.2806 | 0.7830 |
| ｐ16731 | 42 | 0.34 (0.32, 0.36) | 39 | 0.36 (0.34, 0.37) | 0.0778 | 0.7809 |
| ｐ14840 | 42 | 0.21 (0.13, 0.28) | 39 | 0.13 (0.05, 0.21) | -0.7012 | 0.7791 |
| ｐ14068 | 42 | 0.14 (0.09, 0.2) | 39 | 0.09 (0.03, 0.15) | -0.7108 | 0.7790 |
| ｐ22079 | 42 | 0.17 (0.12, 0.22) | 39 | 0.22 (0.17, 0.27) | 0.3652 | 0.7775 |
| ｐ14775 | 42 | 0.09 (0.03, 0.15) | 39 | 0.15 (0.09, 0.21) | 0.7749 | 0.7772 |
| ｐ16503 | 42 | 0.26 (0.18, 0.33) | 39 | 0.18 (0.1, 0.26) | -0.5113 | 0.7770 |
| ｐ19809 | 42 | 0.34 (0.29, 0.38) | 39 | 0.38 (0.33, 0.42) | 0.1677 | 0.7768 |
| ｐ17857 | 42 | 0.32 (0.26, 0.38) | 39 | 0.26 (0.21, 0.32) | -0.2786 | 0.7768 |
| ｐ14971 | 42 | 0.55 (0.41, 0.7) | 39 | 0.4 (0.25, 0.56) | -0.4483 | 0.7767 |
| ｐ200 | 42 | 1.57 (0.88, 2.26) | 39 | 0.87 (0.15, 1.59) | -0.8491 | 0.7762 |
| ｐ10409 | 42 | 0.88 (0.55, 1.22) | 39 | 1.22 (0.87, 1.56) | 0.4641 | 0.7758 |
| ｐ15201 | 42 | 0.4 (0.27, 0.53) | 39 | 0.27 (0.13, 0.4) | -0.5776 | 0.7752 |
| ｐ75 | 42 | 0.13 (-0.09, 0.34) | 39 | 0.34 (0.12, 0.56) | 1.4252 | 0.7746 |
| ｐ22046 | 42 | 0.21 (0.13, 0.29) | 39 | 0.29 (0.21, 0.38) | 0.4881 | 0.7743 |
| ｐ14751 | 42 | 1.55 (0.63, 2.47) | 39 | 0.62 (-0.34, 1.58) | -1.3209 | 0.7742 |
| ｐ15307 | 42 | 0.17 (0.07, 0.26) | 39 | 0.07 (-0.03, 0.17) | -1.2696 | 0.7739 |
| ｐ432 | 42 | 0.86 (0.68, 1.03) | 39 | 1.03 (0.85, 1.21) | 0.2657 | 0.7737 |
| ｐ17880 | 42 | 0.51 (0.38, 0.64) | 39 | 0.38 (0.25, 0.52) | -0.4176 | 0.7719 |
| ｐ15259 | 42 | 0.36 (0.29, 0.42) | 39 | 0.29 (0.23, 0.36) | -0.2867 | 0.7708 |
| ｐ15334 | 42 | 0.13 (0.03, 0.24) | 39 | 0.03 (-0.07, 0.14) | -2.0320 | 0.7693 |
| ｐ7481 | 42 | 0.06 (0.01, 0.11) | 39 | 0.01 (-0.04, 0.06) | -2.2377 | 0.7688 |
| ｐ387 | 42 | 1.07 (-0.87, 3.01) | 39 | 3.01 (1, 5.02) | 1.4938 | 0.7687 |
| ｐ12592 | 42 | 0.58 (0.38, 0.78) | 39 | 0.78 (0.57, 0.99) | 0.4325 | 0.7686 |
| ｐ15175 | 42 | 0.27 (0.21, 0.33) | 39 | 0.33 (0.27, 0.39) | 0.2791 | 0.7683 |
| ｐ3054 | 42 | 0.28 (0.14, 0.42) | 39 | 0.42 (0.27, 0.56) | 0.5859 | 0.7666 |
| ｐ13860 | 42 | 0.17 (0.07, 0.27) | 39 | 0.27 (0.17, 0.38) | 0.6602 | 0.7664 |
| ｐ17256 | 42 | 0.68 (0.46, 0.89) | 39 | 0.46 (0.23, 0.68) | -0.5563 | 0.7652 |
| ｐ15169 | 42 | 0.06 (0, 0.12) | 39 | 0 (-0.06, 0.06) | NA | 0.7650 |
| ｐ14742 | 42 | 0.06 (0, 0.12) | 39 | 0 (-0.06, 0.06) | NA | 0.7649 |
| ｐ15545 | 42 | 0.42 (0.4, 0.45) | 39 | 0.45 (0.42, 0.47) | 0.0800 | 0.7645 |
| ｐ17767 | 42 | 0.47 (0.36, 0.58) | 39 | 0.36 (0.25, 0.47) | -0.3710 | 0.7645 |
| ｐ15184 | 42 | 0.06 (0.03, 0.08) | 39 | 0.03 (0.01, 0.06) | -0.7414 | 0.7645 |
| ｐ22748 | 42 | 0.86 (0.52, 1.19) | 39 | 1.19 (0.85, 1.54) | 0.4736 | 0.7643 |
| ｐ13570 | 42 | 0.27 (0.15, 0.38) | 39 | 0.15 (0.03, 0.27) | -0.8229 | 0.7643 |
| ｐ15331 | 42 | 0.34 (0, 0.69) | 39 | 0 (-0.36, 0.36) | NA | 0.7642 |
| ｐ22946 | 42 | 0.64 (0.41, 0.86) | 39 | 0.86 (0.63, 1.1) | 0.4382 | 0.7637 |
| ｐ10027 | 42 | 0.14 (0.08, 0.19) | 39 | 0.08 (0.03, 0.14) | -0.6865 | 0.7637 |
| ｐ11236 | 42 | 0.7 (-0.17, 1.57) | 39 | 1.56 (0.66, 2.47) | 1.1620 | 0.7633 |
| ｐ2289 | 42 | 0.2 (0.09, 0.31) | 39 | 0.09 (-0.02, 0.2) | -1.1386 | 0.7632 |
| ｐ21924 | 42 | 0.26 (0.1, 0.42) | 39 | 0.1 (-0.06, 0.26) | -1.3401 | 0.7611 |
| ｐ17110 | 42 | 0.34 (0.19, 0.48) | 39 | 0.48 (0.33, 0.63) | 0.5134 | 0.7607 |
| ｐ22733 | 42 | 0.46 (0.42, 0.5) | 39 | 0.5 (0.46, 0.54) | 0.1212 | 0.7604 |
| ｐ15162 | 42 | 0.1 (0, 0.2) | 39 | 0 (-0.11, 0.11) | NA | 0.7602 |
| ｐ18481 | 42 | 0.35 (0.28, 0.41) | 39 | 0.41 (0.35, 0.48) | 0.2449 | 0.7602 |
| ｐ15645 | 42 | 0.91 (0.35, 1.48) | 39 | 0.35 (-0.23, 0.94) | -1.3659 | 0.7590 |
| ｐ9214 | 42 | 0.52 (0.44, 0.59) | 39 | 0.44 (0.36, 0.52) | -0.2367 | 0.7585 |
| ｐ18287 | 42 | 0.06 (0, 0.12) | 39 | 0 (-0.06, 0.06) | NA | 0.7575 |
| ｐ11304 | 42 | 0.4 (0.27, 0.53) | 39 | 0.27 (0.13, 0.41) | -0.5684 | 0.7574 |
| ｐ22294 | 42 | 0.34 (0.2, 0.48) | 39 | 0.48 (0.34, 0.63) | 0.4889 | 0.7568 |
| ｐ22671 | 42 | 0 (-0.04, 0.04) | 39 | 0.04 (0, 0.08) | NA | 0.7565 |
| ｐ13137 | 42 | 0.23 (0.15, 0.32) | 39 | 0.15 (0.06, 0.24) | -0.6280 | 0.7564 |
| ｐ14785 | 42 | 0.27 (0.22, 0.32) | 39 | 0.22 (0.17, 0.27) | -0.2907 | 0.7563 |
| ｐ15540 | 42 | 0.51 (0.34, 0.68) | 39 | 0.68 (0.5, 0.86) | 0.4139 | 0.7552 |
| ｐ22196 | 42 | 0.35 (0.28, 0.41) | 39 | 0.28 (0.22, 0.35) | -0.2986 | 0.7552 |
| ｐ14880 | 42 | 0.26 (0.22, 0.29) | 39 | 0.22 (0.18, 0.26) | -0.2231 | 0.7543 |
| ｐ20877 | 42 | 0.21 (0.13, 0.3) | 39 | 0.13 (0.05, 0.22) | -0.6997 | 0.7542 |
| ｐ14827 | 42 | 0.04 (0, 0.08) | 39 | 0 (-0.04, 0.04) | NA | 0.7541 |
| ｐ22010 | 42 | 0.15 (0.08, 0.22) | 39 | 0.22 (0.15, 0.3) | 0.5680 | 0.7534 |
| ｐ2963 | 42 | 0.4 (-0.01, 0.8) | 39 | 0 (-0.42, 0.42) | NA | 0.7524 |
| ｐ15328 | 42 | 0.45 (0.38, 0.51) | 39 | 0.39 (0.32, 0.45) | -0.2224 | 0.7515 |
| ｐ21897 | 42 | 0.67 (0.53, 0.81) | 39 | 0.53 (0.38, 0.68) | -0.3398 | 0.7503 |
| ｐ15242 | 42 | 0.05 (0, 0.09) | 39 | 0 (-0.05, 0.05) | NA | 0.7496 |
| ｐ18332 | 42 | 0.4 (0.02, 0.78) | 39 | 0.77 (0.38, 1.16) | 0.9508 | 0.7488 |
| ｐ13547 | 42 | 0.47 (0.35, 0.59) | 39 | 0.35 (0.22, 0.48) | -0.4299 | 0.7456 |
| ｐ20770 | 42 | 0.47 (0.35, 0.59) | 39 | 0.35 (0.22, 0.48) | -0.4299 | 0.7456 |
| ｐ15718 | 42 | 0.02 (-0.05, 0.09) | 39 | 0.09 (0.02, 0.16) | 2.0988 | 0.7450 |
| ｐ11570 | 42 | 0.37 (0.31, 0.44) | 39 | 0.31 (0.24, 0.38) | -0.2732 | 0.7443 |
| ｐ21353 | 42 | 0.25 (0.15, 0.34) | 39 | 0.16 (0.06, 0.25) | -0.6530 | 0.7442 |
| ｐ18832 | 42 | 0.57 (0.49, 0.65) | 39 | 0.49 (0.41, 0.58) | -0.2128 | 0.7426 |
| ｐ22702 | 42 | 0.39 (0.06, 0.73) | 39 | 0.72 (0.37, 1.07) | 0.8743 | 0.7416 |
| ｐ14910 | 42 | 1.22 (1.02, 1.42) | 39 | 1.03 (0.82, 1.24) | -0.2509 | 0.7416 |
| ｐ15172 | 42 | 0.07 (0.03, 0.1) | 39 | 0.03 (0, 0.07) | -1.0064 | 0.7411 |
| ｐ15332 | 42 | 0.05 (0, 0.11) | 39 | 0 (-0.06, 0.06) | NA | 0.7380 |
| ｐ10017 | 42 | 0.55 (0.39, 0.7) | 39 | 0.4 (0.24, 0.56) | -0.4647 | 0.7371 |
| ｐ10338 | 42 | 0.25 (0.16, 0.34) | 39 | 0.33 (0.24, 0.42) | 0.4251 | 0.7370 |
| ｐ14353 | 42 | 0.66 (0.51, 0.81) | 39 | 0.51 (0.36, 0.67) | -0.3562 | 0.7369 |
| ｐ10193 | 42 | 1.02 (0.66, 1.37) | 39 | 0.67 (0.3, 1.04) | -0.6026 | 0.7365 |
| ｐ19468 | 42 | 0.49 (0.04, 0.95) | 39 | 0.94 (0.46, 1.41) | 0.9247 | 0.7364 |
| ｐ987 | 42 | 1.59 (0.67, 2.51) | 39 | 2.49 (1.53, 3.44) | 0.6441 | 0.7363 |
| ｐ17357 | 42 | 0.56 (0.47, 0.64) | 39 | 0.47 (0.38, 0.56) | -0.2359 | 0.7361 |
| ｐ20415 | 42 | 0.75 (0.64, 0.85) | 39 | 0.84 (0.74, 0.95) | 0.1784 | 0.7356 |
| ｐ15447 | 42 | 0.41 (0.06, 0.76) | 39 | 0.75 (0.39, 1.12) | 0.8650 | 0.7354 |
| ｐ22054 | 42 | 0 (-0.02, 0.02) | 39 | 0.02 (0, 0.05) | NA | 0.7351 |
| ｐ18715 | 42 | 0.06 (0.03, 0.09) | 39 | 0.03 (-0.01, 0.06) | -1.0810 | 0.7349 |
| ｐ10722 | 42 | 0.68 (0.58, 0.78) | 39 | 0.59 (0.48, 0.69) | -0.2207 | 0.7307 |
| ｐ15015 | 42 | 0.35 (0.29, 0.41) | 39 | 0.29 (0.23, 0.36) | -0.2713 | 0.7300 |
| ｐ15273 | 42 | 0.45 (0.38, 0.52) | 39 | 0.38 (0.31, 0.46) | -0.2348 | 0.7291 |
| ｐ21296 | 42 | 0.37 (0.22, 0.52) | 39 | 0.51 (0.36, 0.67) | 0.4731 | 0.7274 |
| ｐ13145 | 42 | 0.53 (0.33, 0.72) | 39 | 0.34 (0.14, 0.54) | -0.6314 | 0.7260 |
| ｐ15298 | 42 | 0.11 (0.08, 0.13) | 39 | 0.08 (0.06, 0.11) | -0.3204 | 0.7247 |
| ｐ14946 | 42 | 0.44 (0.32, 0.56) | 39 | 0.55 (0.43, 0.68) | 0.3355 | 0.7241 |
| ｐ20442 | 42 | 0.38 (0.34, 0.42) | 39 | 0.42 (0.37, 0.46) | 0.1434 | 0.7230 |
| ｐ22338 | 42 | 0.34 (0.22, 0.47) | 39 | 0.22 (0.09, 0.35) | -0.6345 | 0.7227 |
| ｐ22545 | 42 | 0.35 (0.27, 0.43) | 39 | 0.28 (0.2, 0.36) | -0.3342 | 0.7223 |
| ｐ14774 | 42 | 0.21 (0.13, 0.29) | 39 | 0.28 (0.2, 0.36) | 0.4343 | 0.7220 |
| ｐ15302 | 42 | 0.03 (0, 0.05) | 39 | 0 (-0.03, 0.03) | NA | 0.7217 |
| ｐ22679 | 42 | 0.08 (-0.24, 0.4) | 39 | 0.39 (0.05, 0.72) | 2.2996 | 0.7207 |
| ｐ15168 | 39 | 0.24 (0.13, 0.34) | 39 | 0.14 (0.04, 0.24) | -0.7547 | 0.7206 |
| ｐ14220 | 42 | 0.48 (0.3, 0.65) | 39 | 0.65 (0.46, 0.83) | 0.4370 | 0.7205 |
| ｐ22422 | 42 | 0.18 (0.13, 0.23) | 39 | 0.23 (0.17, 0.28) | 0.3399 | 0.7201 |
| ｐ15278 | 42 | 0.41 (0.33, 0.48) | 39 | 0.34 (0.26, 0.41) | -0.2755 | 0.7183 |
| ｐ13450 | 42 | 0.4 (0.25, 0.55) | 39 | 0.26 (0.11, 0.41) | -0.6282 | 0.7166 |
| ｐ11134 | 42 | 0.42 (0.33, 0.51) | 39 | 0.33 (0.24, 0.43) | -0.3362 | 0.7164 |
| ｐ20924 | 42 | 0.24 (0.18, 0.31) | 39 | 0.18 (0.11, 0.25) | -0.4208 | 0.7149 |
| ｐ19712 | 42 | 0.08 (-0.05, 0.2) | 39 | 0.2 (0.07, 0.33) | 1.3631 | 0.7144 |
| ｐ19695 | 41 | 0.68 (0.68, 0.69) | 39 | 0.68 (0.67, 0.68) | -0.0111 | 0.7142 |
| ｐ11702 | 42 | 0.13 (0.08, 0.18) | 39 | 0.18 (0.13, 0.23) | 0.4481 | 0.7142 |
| ｐ18825 | 42 | 0.52 (0.12, 0.91) | 39 | 0.89 (0.48, 1.31) | 0.7933 | 0.7138 |
| ｐ16443 | 42 | 0.5 (0.36, 0.65) | 39 | 0.64 (0.49, 0.79) | 0.3457 | 0.7126 |
| ｐ430 | 42 | 0.65 (0.5, 0.8) | 39 | 0.51 (0.36, 0.66) | -0.3447 | 0.7109 |
| ｐ9791 | 42 | 0.46 (0.3, 0.62) | 39 | 0.31 (0.15, 0.48) | -0.5719 | 0.7109 |
| ｐ12776 | 42 | 0.22 (0.16, 0.27) | 39 | 0.17 (0.11, 0.22) | -0.3884 | 0.7103 |
| ｐ22158 | 42 | 0.27 (0.13, 0.4) | 39 | 0.4 (0.25, 0.54) | 0.5741 | 0.7091 |
| ｐ15231 | 42 | 0.85 (0.55, 1.15) | 39 | 0.57 (0.26, 0.88) | -0.5854 | 0.7089 |
| ｐ19279 | 42 | 0.25 (0.12, 0.39) | 39 | 0.38 (0.24, 0.53) | 0.5944 | 0.7058 |
| ｐ15257 | 42 | 0.44 (0.3, 0.57) | 39 | 0.31 (0.16, 0.45) | -0.5140 | 0.7038 |
| ｐ17990 | 42 | 0.69 (0.46, 0.93) | 39 | 0.91 (0.67, 1.15) | 0.3966 | 0.7036 |
| ｐ13042 | 42 | 0.42 (0.04, 0.79) | 39 | 0.77 (0.38, 1.16) | 0.8916 | 0.7027 |
| ｐ15886 | 42 | 0.76 (0.76, 0.76) | 39 | 0.76 (0.76, 0.76) | -0.0003 | 0.7022 |
| ｐ16607 | 42 | 0.93 (0.93, 0.93) | 39 | 0.93 (0.93, 0.93) | -0.0007 | 0.7022 |
| ｐ17444 | 42 | 0.8 (0.8, 0.81) | 39 | 0.8 (0.8, 0.8) | -0.0019 | 0.7022 |
| ｐ19745 | 42 | 0.65 (0.64, 0.65) | 39 | 0.64 (0.64, 0.65) | -0.0022 | 0.7022 |
| ｐ17786 | 42 | 0.9 (0.9, 0.9) | 39 | 0.9 (0.9, 0.9) | -0.0024 | 0.7022 |
| ｐ15350 | 42 | 1.02 (1.02, 1.03) | 39 | 1.02 (1.02, 1.02) | -0.0034 | 0.7022 |
| ｐ15989 | 42 | 0.82 (0.82, 0.83) | 39 | 0.82 (0.82, 0.82) | -0.0036 | 0.7022 |
| ｐ17433 | 42 | 0.59 (0.59, 0.59) | 39 | 0.59 (0.58, 0.59) | -0.0044 | 0.7022 |
| ｐ15808 | 42 | 0.41 (0.41, 0.42) | 39 | 0.41 (0.41, 0.41) | -0.0049 | 0.7022 |
| ｐ18923 | 42 | 0.64 (0.64, 0.65) | 39 | 0.64 (0.64, 0.64) | -0.0066 | 0.7022 |
| ｐ18977 | 42 | 0.74 (0.74, 0.75) | 39 | 0.74 (0.74, 0.74) | -0.0074 | 0.7022 |
| ｐ16234 | 42 | 0.46 (0.46, 0.47) | 39 | 0.46 (0.46, 0.46) | -0.0077 | 0.7022 |
| ｐ16385 | 42 | 0.46 (0.46, 0.47) | 39 | 0.46 (0.46, 0.46) | -0.0077 | 0.7022 |
| ｐ16699 | 42 | 0.81 (0.81, 0.82) | 39 | 0.81 (0.8, 0.81) | -0.0082 | 0.7022 |
| ｐ17039 | 42 | 0.81 (0.81, 0.82) | 39 | 0.81 (0.8, 0.81) | -0.0082 | 0.7022 |
| ｐ16758 | 42 | 0.69 (0.68, 0.69) | 39 | 0.68 (0.68, 0.69) | -0.0110 | 0.7022 |
| ｐ17546 | 42 | 0.78 (0.77, 0.79) | 39 | 0.77 (0.77, 0.78) | -0.0111 | 0.7022 |
| ｐ16432 | 42 | 1.18 (1.17, 1.19) | 39 | 1.17 (1.16, 1.18) | -0.0114 | 0.7022 |
| ｐ15767 | 42 | 0.23 (0.23, 0.23) | 39 | 0.23 (0.23, 0.23) | -0.0120 | 0.7022 |
| ｐ16744 | 42 | 0.77 (0.76, 0.77) | 39 | 0.76 (0.75, 0.77) | -0.0134 | 0.7022 |
| ｐ19357 | 42 | 0.74 (0.73, 0.75) | 39 | 0.73 (0.72, 0.74) | -0.0135 | 0.7022 |
| ｐ17485 | 42 | 0.73 (0.73, 0.74) | 39 | 0.73 (0.72, 0.73) | -0.0135 | 0.7022 |
| ｐ16782 | 42 | 0.77 (0.76, 0.78) | 39 | 0.76 (0.76, 0.77) | -0.0137 | 0.7022 |
| ｐ19755 | 42 | 1.06 (1.05, 1.07) | 39 | 1.05 (1.04, 1.06) | -0.0142 | 0.7022 |
| ｐ19156 | 42 | 0.83 (0.82, 0.84) | 39 | 0.83 (0.82, 0.83) | -0.0147 | 0.7022 |
| ｐ17922 | 42 | 0.74 (0.73, 0.74) | 39 | 0.73 (0.72, 0.74) | -0.0165 | 0.7022 |
| ｐ17230 | 42 | 0.66 (0.65, 0.67) | 39 | 0.65 (0.64, 0.66) | -0.0166 | 0.7022 |
| ｐ17057 | 42 | 0.75 (0.75, 0.76) | 39 | 0.75 (0.74, 0.76) | -0.0168 | 0.7022 |
| ｐ18005 | 42 | 1.41 (1.39, 1.43) | 39 | 1.4 (1.38, 1.42) | -0.0189 | 0.7022 |
| ｐ15385 | 42 | 1.18 (1.16, 1.2) | 39 | 1.16 (1.14, 1.18) | -0.0213 | 0.7022 |
| ｐ16059 | 42 | 1.31 (1.29, 1.33) | 39 | 1.29 (1.27, 1.31) | -0.0222 | 0.7022 |
| ｐ17475 | 42 | 0.48 (0.48, 0.49) | 39 | 0.48 (0.47, 0.49) | -0.0228 | 0.7022 |
| ｐ15830 | 42 | 1.01 (0.99, 1.03) | 39 | 1 (0.98, 1.01) | -0.0229 | 0.7022 |
| ｐ16407 | 42 | 1.92 (1.89, 1.96) | 39 | 1.89 (1.86, 1.93) | -0.0229 | 0.7022 |
| ｐ17174 | 42 | 0.55 (0.54, 0.55) | 39 | 0.54 (0.53, 0.55) | -0.0233 | 0.7022 |
| ｐ17140 | 42 | 0.69 (0.68, 0.7) | 39 | 0.68 (0.67, 0.69) | -0.0237 | 0.7022 |
| ｐ17074 | 42 | 0.56 (0.55, 0.57) | 39 | 0.55 (0.54, 0.56) | -0.0252 | 0.7022 |
| ｐ17813 | 42 | 0.41 (0.4, 0.41) | 39 | 0.4 (0.39, 0.41) | -0.0281 | 0.7022 |
| ｐ19778 | 42 | 0.76 (0.74, 0.77) | 39 | 0.74 (0.72, 0.76) | -0.0289 | 0.7022 |
| ｐ15636 | 42 | 0.7 (0.68, 0.72) | 39 | 0.69 (0.67, 0.7) | -0.0310 | 0.7022 |
| ｐ16338 | 42 | 0.28 (0.27, 0.28) | 39 | 0.27 (0.27, 0.28) | -0.0316 | 0.7022 |
| ｐ18748 | 42 | 0.97 (0.95, 0.99) | 39 | 0.95 (0.92, 0.97) | -0.0329 | 0.7022 |
| ｐ19234 | 42 | 0.44 (0.43, 0.46) | 39 | 0.43 (0.42, 0.45) | -0.0363 | 0.7022 |
| ｐ16705 | 42 | 0.95 (0.93, 0.98) | 39 | 0.93 (0.9, 0.95) | -0.0363 | 0.7022 |
| ｐ15852 | 42 | 0.46 (0.45, 0.47) | 39 | 0.45 (0.44, 0.46) | -0.0369 | 0.7022 |
| ｐ15906 | 42 | 0.36 (0.35, 0.37) | 39 | 0.35 (0.34, 0.36) | -0.0375 | 0.7022 |
| ｐ16999 | 42 | 0.64 (0.62, 0.66) | 39 | 0.62 (0.6, 0.64) | -0.0418 | 0.7022 |
| ｐ15660 | 42 | 0.65 (0.63, 0.67) | 39 | 0.63 (0.61, 0.65) | -0.0422 | 0.7022 |
| ｐ19657 | 42 | 0.55 (0.53, 0.56) | 39 | 0.53 (0.51, 0.55) | -0.0422 | 0.7022 |
| ｐ16918 | 42 | 0.69 (0.67, 0.71) | 39 | 0.67 (0.65, 0.69) | -0.0443 | 0.7022 |
| ｐ18066 | 42 | 0.52 (0.5, 0.53) | 39 | 0.5 (0.48, 0.52) | -0.0450 | 0.7022 |
| ｐ15802 | 42 | 0.53 (0.51, 0.55) | 39 | 0.52 (0.5, 0.53) | -0.0469 | 0.7022 |
| ｐ15717 | 42 | 0.63 (0.6, 0.65) | 39 | 0.61 (0.58, 0.63) | -0.0488 | 0.7022 |
| ｐ16152 | 42 | 1.96 (1.89, 2.03) | 39 | 1.89 (1.82, 1.97) | -0.0488 | 0.7022 |
| ｐ18168 | 42 | 0.61 (0.59, 0.64) | 39 | 0.59 (0.57, 0.62) | -0.0489 | 0.7022 |
| ｐ17557 | 42 | 0.79 (0.76, 0.82) | 39 | 0.76 (0.73, 0.79) | -0.0501 | 0.7022 |
| ｐ17001 | 42 | 0.51 (0.49, 0.53) | 39 | 0.5 (0.47, 0.52) | -0.0534 | 0.7022 |
| ｐ19606 | 42 | 1.03 (0.99, 1.08) | 39 | 0.99 (0.95, 1.04) | -0.0556 | 0.7022 |
| ｐ16320 | 42 | 0.41 (0.39, 0.43) | 39 | 0.39 (0.38, 0.41) | -0.0588 | 0.7022 |
| ｐ18650 | 42 | 0.41 (0.39, 0.43) | 39 | 0.39 (0.38, 0.41) | -0.0588 | 0.7022 |
| ｐ19031 | 42 | 0.41 (0.39, 0.43) | 39 | 0.39 (0.38, 0.41) | -0.0588 | 0.7022 |
| ｐ17851 | 42 | 0.53 (0.51, 0.55) | 39 | 0.51 (0.49, 0.53) | -0.0603 | 0.7022 |
| ｐ16029 | 42 | 0.78 (0.75, 0.82) | 39 | 0.75 (0.71, 0.79) | -0.0612 | 0.7022 |
| ｐ18836 | 42 | 0.73 (0.69, 0.76) | 39 | 0.7 (0.66, 0.73) | -0.0622 | 0.7022 |
| ｐ15147 | 42 | 0.91 (0.87, 0.95) | 39 | 0.87 (0.83, 0.92) | -0.0624 | 0.7022 |
| ｐ16332 | 42 | 0.4 (0.38, 0.42) | 39 | 0.38 (0.36, 0.4) | -0.0632 | 0.7022 |
| ｐ15007 | 42 | 0.3 (0.28, 0.31) | 39 | 0.28 (0.27, 0.3) | -0.0674 | 0.7022 |
| ｐ18125 | 42 | 0.4 (0.38, 0.42) | 39 | 0.38 (0.36, 0.4) | -0.0699 | 0.7022 |
| ｐ15688 | 42 | 0.24 (0.23, 0.25) | 39 | 0.23 (0.22, 0.24) | -0.0727 | 0.7022 |
| ｐ15624 | 42 | 0.37 (0.35, 0.38) | 39 | 0.35 (0.33, 0.37) | -0.0727 | 0.7022 |
| ｐ16006 | 42 | 0.31 (0.3, 0.33) | 39 | 0.3 (0.28, 0.31) | -0.0729 | 0.7022 |
| ｐ19550 | 42 | 0.93 (0.88, 0.98) | 39 | 0.88 (0.83, 0.93) | -0.0731 | 0.7022 |
| ｐ18757 | 42 | 1.67 (1.58, 1.76) | 39 | 1.59 (1.5, 1.68) | -0.0749 | 0.7022 |
| ｐ16595 | 42 | 0.33 (0.31, 0.35) | 39 | 0.31 (0.29, 0.33) | -0.0750 | 0.7022 |
| ｐ17840 | 42 | 0.66 (0.62, 0.7) | 39 | 0.62 (0.59, 0.66) | -0.0783 | 0.7022 |
| ｐ16456 | 42 | 0.39 (0.37, 0.42) | 39 | 0.37 (0.35, 0.4) | -0.0828 | 0.7022 |
| ｐ15869 | 42 | 0.96 (0.9, 1.02) | 39 | 0.91 (0.85, 0.97) | -0.0853 | 0.7022 |
| ｐ17013 | 42 | 0.82 (0.77, 0.87) | 39 | 0.77 (0.72, 0.82) | -0.0858 | 0.7022 |
| ｐ16049 | 42 | 0.55 (0.52, 0.59) | 39 | 0.52 (0.48, 0.55) | -0.0862 | 0.7022 |
| ｐ15704 | 42 | 0.63 (0.59, 0.67) | 39 | 0.6 (0.56, 0.64) | -0.0867 | 0.7022 |
| ｐ18613 | 42 | 0.63 (0.59, 0.67) | 39 | 0.6 (0.56, 0.64) | -0.0867 | 0.7022 |
| ｐ15744 | 42 | 0.42 (0.4, 0.45) | 39 | 0.4 (0.37, 0.43) | -0.0886 | 0.7022 |
| ｐ17158 | 42 | 0.29 (0.27, 0.31) | 39 | 0.27 (0.25, 0.29) | -0.0887 | 0.7022 |
| ｐ18207 | 42 | 0.34 (0.32, 0.36) | 39 | 0.32 (0.29, 0.34) | -0.0897 | 0.7022 |
| ｐ18790 | 42 | 0.43 (0.4, 0.46) | 39 | 0.4 (0.37, 0.44) | -0.1009 | 0.7022 |
| ｐ17454 | 42 | 0.23 (0.21, 0.25) | 39 | 0.21 (0.2, 0.23) | -0.1022 | 0.7022 |
| ｐ18703 | 42 | 0.28 (0.26, 0.3) | 39 | 0.26 (0.24, 0.28) | -0.1046 | 0.7022 |
| ｐ19629 | 42 | 0.36 (0.34, 0.39) | 39 | 0.34 (0.31, 0.37) | -0.1054 | 0.7022 |
| ｐ15877 | 42 | 0.52 (0.48, 0.56) | 39 | 0.49 (0.44, 0.53) | -0.1086 | 0.7022 |
| ｐ19211 | 42 | 0.41 (0.37, 0.44) | 39 | 0.37 (0.34, 0.41) | -0.1185 | 0.7022 |
| ｐ19171 | 42 | 0.51 (0.47, 0.56) | 39 | 0.47 (0.43, 0.52) | -0.1222 | 0.7022 |
| ｐ16847 | 42 | 0.39 (0.35, 0.42) | 39 | 0.35 (0.32, 0.39) | -0.1242 | 0.7022 |
| ｐ16663 | 42 | 0.29 (0.26, 0.32) | 39 | 0.27 (0.24, 0.29) | -0.1298 | 0.7022 |
| ｐ19940 | 42 | 0.48 (0.43, 0.53) | 39 | 0.43 (0.38, 0.49) | -0.1550 | 0.7022 |
| ｐ18102 | 42 | 0.24 (0.21, 0.26) | 39 | 0.21 (0.19, 0.24) | -0.1552 | 0.7022 |
| ｐ19936 | 42 | 0.24 (0.21, 0.26) | 39 | 0.21 (0.19, 0.24) | -0.1552 | 0.7022 |
| ｐ18251 | 42 | 0.18 (0.16, 0.21) | 39 | 0.16 (0.14, 0.19) | -0.1739 | 0.7022 |
| ｐ17398 | 42 | 0.3 (0.26, 0.33) | 39 | 0.26 (0.22, 0.3) | -0.1781 | 0.7022 |
| ｐ17033 | 42 | 0.25 (0.22, 0.28) | 39 | 0.22 (0.19, 0.25) | -0.1825 | 0.7022 |
| ｐ17522 | 42 | 0.27 (0.23, 0.31) | 39 | 0.24 (0.2, 0.28) | -0.2064 | 0.7022 |
| ｐ16262 | 42 | 0.19 (0.16, 0.22) | 39 | 0.17 (0.14, 0.2) | -0.2154 | 0.7022 |
| ｐ17816 | 42 | 0.12 (0.1, 0.14) | 39 | 0.1 (0.09, 0.12) | -0.2160 | 0.7022 |
| ｐ16958 | 42 | 0.21 (0.18, 0.25) | 39 | 0.18 (0.15, 0.22) | -0.2247 | 0.7022 |
| ｐ17661 | 42 | 0.28 (0.24, 0.32) | 39 | 0.24 (0.2, 0.29) | -0.2263 | 0.7022 |
| ｐ18716 | 42 | 0.63 (0.53, 0.73) | 39 | 0.54 (0.43, 0.64) | -0.2279 | 0.7022 |
| ｐ15024 | 42 | 0.27 (0.23, 0.31) | 39 | 0.23 (0.18, 0.27) | -0.2320 | 0.7022 |
| ｐ16984 | 42 | 0.2 (0.17, 0.24) | 39 | 0.17 (0.14, 0.21) | -0.2395 | 0.7022 |
| ｐ16015 | 42 | 0.21 (0.18, 0.25) | 39 | 0.18 (0.14, 0.22) | -0.2490 | 0.7022 |
| ｐ17718 | 42 | 0.27 (0.23, 0.32) | 39 | 0.23 (0.18, 0.28) | -0.2652 | 0.7022 |
| ｐ15907 | 42 | 0.29 (0.24, 0.35) | 39 | 0.24 (0.19, 0.3) | -0.2689 | 0.7022 |
| ｐ19168 | 42 | 0.35 (0.28, 0.42) | 39 | 0.29 (0.21, 0.36) | -0.2952 | 0.7022 |
| ｐ19765 | 42 | 0.09 (0.07, 0.11) | 39 | 0.07 (0.05, 0.09) | -0.3231 | 0.7022 |
| ｐ16609 | 42 | 0.25 (0.19, 0.31) | 39 | 0.2 (0.13, 0.26) | -0.3674 | 0.7022 |
| ｐ16158 | 42 | 0.27 (0.2, 0.33) | 39 | 0.21 (0.14, 0.27) | -0.3684 | 0.7022 |
| ｐ17949 | 42 | 0.62 (0.47, 0.77) | 39 | 0.48 (0.32, 0.63) | -0.3775 | 0.7022 |
| ｐ17354 | 42 | 0.08 (0.06, 0.1) | 39 | 0.06 (0.04, 0.08) | -0.3877 | 0.7022 |
| ｐ17751 | 42 | 0.16 (0.12, 0.21) | 39 | 0.12 (0.08, 0.17) | -0.4295 | 0.7022 |
| ｐ19931 | 42 | 0.1 (0.07, 0.14) | 39 | 0.07 (0.04, 0.11) | -0.4810 | 0.7022 |
| ｐ19733 | 42 | 0.15 (0.1, 0.2) | 39 | 0.11 (0.06, 0.16) | -0.4916 | 0.7022 |
| ｐ15854 | 42 | 0.17 (0.11, 0.22) | 39 | 0.11 (0.06, 0.17) | -0.5329 | 0.7022 |
| ｐ16027 | 42 | 0.17 (0.11, 0.22) | 39 | 0.11 (0.06, 0.17) | -0.5329 | 0.7022 |
| ｐ16073 | 42 | 0.04 (0.03, 0.05) | 39 | 0.03 (0.01, 0.04) | -0.5333 | 0.7022 |
| ｐ19242 | 42 | 0.15 (0.1, 0.2) | 39 | 0.1 (0.05, 0.16) | -0.5350 | 0.7022 |
| ｐ19853 | 42 | 0.08 (0.05, 0.11) | 39 | 0.05 (0.02, 0.08) | -0.6533 | 0.7022 |
| ｐ19851 | 42 | 0.34 (0.2, 0.47) | 39 | 0.21 (0.07, 0.35) | -0.6793 | 0.7022 |
| ｐ19933 | 42 | 0.06 (0.01, 0.1) | 39 | 0.02 (-0.03, 0.06) | -1.8536 | 0.7022 |
| ｐ18263 | 42 | 0.14 (0.01, 0.26) | 39 | 0.02 (-0.1, 0.15) | -2.6288 | 0.7022 |
| ｐ14818 | 42 | 0.61 (0.61, 0.61) | 39 | 0.61 (0.61, 0.61) | 0.0000 | 0.7022 |
| ｐ21451 | 42 | 1.04 (1.04, 1.04) | 39 | 1.04 (1.04, 1.04) | -0.0004 | 0.7022 |
| ｐ22871 | 42 | 2.08 (2.08, 2.08) | 39 | 2.08 (2.08, 2.08) | -0.0011 | 0.7022 |
| ｐ22923 | 42 | 0.82 (0.82, 0.82) | 39 | 0.82 (0.82, 0.82) | -0.0011 | 0.7022 |
| ｐ7601 | 42 | 1.11 (1.11, 1.11) | 39 | 1.11 (1.11, 1.11) | -0.0008 | 0.7022 |
| ｐ22463 | 42 | 1.37 (1.37, 1.37) | 39 | 1.37 (1.37, 1.37) | -0.0007 | 0.7022 |
| ｐ22567 | 42 | 1.01 (1, 1.01) | 39 | 1 (1, 1.01) | -0.0023 | 0.7022 |
| ｐ9390 | 42 | 0.83 (0.83, 0.84) | 39 | 0.83 (0.83, 0.84) | -0.0028 | 0.7022 |
| ｐ22274 | 42 | 1.69 (1.69, 1.69) | 39 | 1.69 (1.68, 1.69) | -0.0017 | 0.7022 |
| ｐ22496 | 42 | 0.99 (0.99, 0.99) | 39 | 0.99 (0.99, 0.99) | -0.0015 | 0.7022 |
| ｐ14080 | 42 | 0.99 (0.99, 0.99) | 39 | 0.99 (0.98, 0.99) | -0.0051 | 0.7022 |
| ｐ22912 | 42 | 0.51 (0.51, 0.51) | 39 | 0.51 (0.51, 0.51) | -0.0047 | 0.7022 |
| ｐ13798 | 42 | 0.93 (0.93, 0.93) | 39 | 0.93 (0.93, 0.93) | -0.0011 | 0.7022 |
| ｐ12323 | 42 | 1.13 (1.13, 1.13) | 39 | 1.13 (1.12, 1.13) | -0.0030 | 0.7022 |
| ｐ21962 | 42 | 1.12 (1.12, 1.12) | 39 | 1.12 (1.11, 1.12) | -0.0052 | 0.7022 |
| ｐ22887 | 42 | 0.58 (0.58, 0.59) | 39 | 0.58 (0.58, 0.58) | -0.0053 | 0.7022 |
| ｐ22909 | 42 | 1.21 (1.2, 1.21) | 39 | 1.2 (1.2, 1.21) | -0.0069 | 0.7022 |
| ｐ11486 | 42 | 0.74 (0.74, 0.75) | 39 | 0.74 (0.74, 0.74) | -0.0074 | 0.7022 |
| ｐ13750 | 42 | 2.21 (2.21, 2.22) | 39 | 2.21 (2.2, 2.21) | -0.0030 | 0.7022 |
| ｐ14836 | 42 | 1.18 (1.16, 1.19) | 39 | 1.16 (1.15, 1.18) | -0.0182 | 0.7022 |
| ｐ14688 | 42 | 0.24 (0.24, 0.25) | 39 | 0.24 (0.23, 0.24) | -0.0266 | 0.7022 |
| ｐ20188 | 42 | 0.43 (0.42, 0.44) | 39 | 0.42 (0.41, 0.43) | -0.0322 | 0.7022 |
| ｐ22649 | 42 | 3.19 (3.18, 3.19) | 39 | 3.18 (3.17, 3.19) | -0.0034 | 0.7022 |
| ｐ11763 | 42 | 0.76 (0.76, 0.77) | 39 | 0.76 (0.76, 0.76) | -0.0045 | 0.7022 |
| ｐ22472 | 42 | 0.87 (0.85, 0.89) | 39 | 0.85 (0.84, 0.87) | -0.0259 | 0.7022 |
| ｐ14792 | 42 | 0.79 (0.78, 0.8) | 39 | 0.78 (0.77, 0.79) | -0.0205 | 0.7022 |
| ｐ14794 | 42 | 0.59 (0.59, 0.59) | 39 | 0.59 (0.58, 0.59) | -0.0084 | 0.7022 |
| ｐ14815 | 42 | 0.58 (0.57, 0.58) | 39 | 0.57 (0.57, 0.58) | -0.0149 | 0.7022 |
| ｐ21758 | 42 | 1.41 (1.39, 1.43) | 39 | 1.4 (1.38, 1.42) | -0.0189 | 0.7022 |
| ｐ22642 | 42 | 1.24 (1.23, 1.25) | 39 | 1.23 (1.22, 1.24) | -0.0098 | 0.7022 |
| ｐ2386 | 42 | 0.4 (0.39, 0.41) | 39 | 0.39 (0.38, 0.4) | -0.0330 | 0.7022 |
| ｐ8479 | 42 | 1.03 (1, 1.05) | 39 | 1 (0.98, 1.03) | -0.0346 | 0.7022 |
| ｐ14800 | 42 | 0.37 (0.37, 0.37) | 39 | 0.37 (0.36, 0.37) | -0.0112 | 0.7022 |
| ｐ22910 | 42 | 0.57 (0.57, 0.58) | 39 | 0.57 (0.57, 0.58) | -0.0110 | 0.7022 |
| ｐ14822 | 42 | 0.75 (0.74, 0.75) | 39 | 0.74 (0.74, 0.75) | -0.0111 | 0.7022 |
| ｐ2803 | 42 | 1.25 (1.24, 1.26) | 39 | 1.24 (1.22, 1.25) | -0.0128 | 0.7022 |
| ｐ14841 | 42 | 0.93 (0.9, 0.96) | 39 | 0.9 (0.87, 0.94) | -0.0454 | 0.7022 |
| ｐ10840 | 42 | 0.46 (0.45, 0.48) | 39 | 0.45 (0.43, 0.47) | -0.0585 | 0.7022 |
| ｐ22178 | 42 | 0.52 (0.51, 0.53) | 39 | 0.51 (0.5, 0.52) | -0.0290 | 0.7022 |
| ｐ20982 | 42 | 0.28 (0.26, 0.29) | 39 | 0.26 (0.25, 0.28) | -0.0641 | 0.7022 |
| ｐ22913 | 42 | 0.77 (0.76, 0.77) | 39 | 0.76 (0.75, 0.77) | -0.0112 | 0.7022 |
| ｐ20110 | 42 | 0.63 (0.62, 0.65) | 39 | 0.62 (0.6, 0.63) | -0.0303 | 0.7022 |
| ｐ21759 | 42 | 0.9 (0.88, 0.92) | 39 | 0.88 (0.86, 0.9) | -0.0333 | 0.7022 |
| ｐ14899 | 42 | 0.5 (0.44, 0.56) | 39 | 0.44 (0.38, 0.51) | -0.1827 | 0.7022 |
| ｐ22637 | 42 | 1.13 (1.11, 1.15) | 39 | 1.11 (1.1, 1.13) | -0.0199 | 0.7022 |
| ｐ20562 | 42 | 1.17 (1.14, 1.19) | 39 | 1.14 (1.11, 1.17) | -0.0334 | 0.7022 |
| ｐ20548 | 42 | 0.33 (0.32, 0.33) | 39 | 0.32 (0.32, 0.33) | -0.0187 | 0.7022 |
| ｐ22635 | 42 | 1.37 (1.35, 1.39) | 39 | 1.35 (1.33, 1.37) | -0.0230 | 0.7022 |
| ｐ22488 | 42 | 0.61 (0.59, 0.63) | 39 | 0.59 (0.57, 0.61) | -0.0449 | 0.7022 |
| ｐ22882 | 42 | 0.62 (0.59, 0.65) | 39 | 0.59 (0.55, 0.62) | -0.0747 | 0.7022 |
| ｐ14675 | 42 | 0.24 (0.21, 0.26) | 39 | 0.21 (0.19, 0.24) | -0.1482 | 0.7022 |
| ｐ22544 | 42 | 0.29 (0.29, 0.29) | 39 | 0.29 (0.29, 0.29) | -0.0067 | 0.7022 |
| ｐ12878 | 42 | 1.01 (0.99, 1.04) | 39 | 0.99 (0.97, 1.02) | -0.0334 | 0.7022 |
| ｐ22906 | 42 | 0.42 (0.39, 0.45) | 39 | 0.39 (0.36, 0.42) | -0.0929 | 0.7022 |
| ｐ7849 | 42 | 0.32 (0.28, 0.36) | 39 | 0.29 (0.25, 0.32) | -0.1601 | 0.7022 |
| ｐ78 | 42 | 1.04 (1.04, 1.05) | 39 | 1.04 (1.04, 1.05) | -0.0057 | 0.7022 |
| ｐ22655 | 42 | 0.6 (0.6, 0.61) | 39 | 0.6 (0.59, 0.6) | -0.0115 | 0.7022 |
| ｐ14848 | 42 | 1.33 (1.31, 1.35) | 39 | 1.31 (1.29, 1.33) | -0.0209 | 0.7022 |
| ｐ13181 | 42 | 0.7 (0.69, 0.71) | 39 | 0.69 (0.68, 0.7) | -0.0227 | 0.7022 |
| ｐ9765 | 42 | 0.69 (0.68, 0.7) | 39 | 0.68 (0.67, 0.69) | -0.0237 | 0.7022 |
| ｐ12696 | 42 | 0.41 (0.38, 0.44) | 39 | 0.38 (0.35, 0.41) | -0.1065 | 0.7022 |
| ｐ22646 | 42 | 0.22 (0.2, 0.24) | 39 | 0.21 (0.19, 0.23) | -0.1223 | 0.7022 |
| ｐ14766 | 42 | 1.16 (1.13, 1.18) | 39 | 1.13 (1.11, 1.16) | -0.0283 | 0.7022 |
| ｐ21858 | 42 | 1.05 (1.02, 1.08) | 39 | 1.02 (0.99, 1.05) | -0.0383 | 0.7022 |
| ｐ22355 | 42 | 0.25 (0.23, 0.27) | 39 | 0.23 (0.21, 0.25) | -0.1073 | 0.7022 |
| ｐ21506 | 42 | 0.33 (0.3, 0.36) | 39 | 0.3 (0.27, 0.33) | -0.1327 | 0.7022 |
| ｐ22922 | 42 | 0.5 (0.49, 0.5) | 39 | 0.49 (0.48, 0.5) | -0.0165 | 0.7022 |
| ｐ22268 | 42 | 1.08 (1.07, 1.1) | 39 | 1.07 (1.05, 1.08) | -0.0186 | 0.7022 |
| ｐ22492 | 42 | 0.63 (0.62, 0.64) | 39 | 0.62 (0.62, 0.63) | -0.0194 | 0.7022 |
| ｐ22653 | 42 | 0.54 (0.53, 0.55) | 39 | 0.53 (0.52, 0.54) | -0.0276 | 0.7022 |
| ｐ23061 | 42 | 0.52 (0.51, 0.54) | 39 | 0.51 (0.5, 0.53) | -0.0302 | 0.7022 |
| ｐ22508 | 42 | 0.38 (0.36, 0.39) | 39 | 0.36 (0.35, 0.38) | -0.0583 | 0.7022 |
| ｐ22547 | 42 | 0.68 (0.65, 0.72) | 39 | 0.65 (0.61, 0.69) | -0.0764 | 0.7022 |
| ｐ22497 | 42 | 0.28 (0.26, 0.3) | 39 | 0.26 (0.24, 0.28) | -0.1018 | 0.7022 |
| ｐ4437 | 42 | 0.43 (0.39, 0.47) | 39 | 0.39 (0.35, 0.43) | -0.1366 | 0.7022 |
| ｐ12629 | 42 | 0.43 (0.37, 0.48) | 39 | 0.37 (0.31, 0.43) | -0.1986 | 0.7022 |
| ｐ10690 | 42 | 0.24 (0.2, 0.28) | 39 | 0.2 (0.16, 0.25) | -0.2568 | 0.7022 |
| ｐ21184 | 42 | 0.64 (0.63, 0.65) | 39 | 0.64 (0.63, 0.64) | -0.0170 | 0.7022 |
| ｐ11965 | 42 | 2.11 (2.06, 2.17) | 39 | 2.07 (2.01, 2.12) | -0.0331 | 0.7022 |
| ｐ22899 | 42 | 0.72 (0.71, 0.74) | 39 | 0.71 (0.69, 0.73) | -0.0344 | 0.7022 |
| ｐ1230 | 42 | 0.61 (0.59, 0.64) | 39 | 0.59 (0.57, 0.62) | -0.0489 | 0.7022 |
| ｐ10752 | 42 | 0.53 (0.51, 0.56) | 39 | 0.51 (0.48, 0.53) | -0.0650 | 0.7022 |
| ｐ22883 | 42 | 0.6 (0.56, 0.63) | 39 | 0.57 (0.53, 0.6) | -0.0803 | 0.7022 |
| ｐ20147 | 42 | 0.4 (0.37, 0.43) | 39 | 0.38 (0.35, 0.41) | -0.0990 | 0.7022 |
| ｐ22872 | 42 | 0.35 (0.32, 0.38) | 39 | 0.32 (0.3, 0.35) | -0.1116 | 0.7022 |
| ｐ22924 | 42 | 0.7 (0.64, 0.76) | 39 | 0.65 (0.59, 0.71) | -0.1157 | 0.7022 |
| ｐ22534 | 42 | 0.57 (0.51, 0.62) | 39 | 0.51 (0.46, 0.57) | -0.1412 | 0.7022 |
| ｐ14753 | 42 | 0.36 (0.33, 0.4) | 39 | 0.33 (0.29, 0.37) | -0.1532 | 0.7022 |
| ｐ21838 | 42 | 0.31 (0.26, 0.35) | 39 | 0.27 (0.22, 0.31) | -0.2148 | 0.7022 |
| ｐ14768 | 42 | 0.2 (0.15, 0.24) | 39 | 0.15 (0.11, 0.2) | -0.3502 | 0.7022 |
| ｐ22650 | 42 | 1.46 (1.44, 1.48) | 39 | 1.45 (1.42, 1.47) | -0.0186 | 0.7022 |
| ｐ22926 | 42 | 0.68 (0.67, 0.69) | 39 | 0.67 (0.66, 0.68) | -0.0238 | 0.7022 |
| ｐ4764 | 42 | 0.74 (0.72, 0.75) | 39 | 0.73 (0.71, 0.74) | -0.0247 | 0.7022 |
| ｐ14781 | 42 | 0.73 (0.72, 0.75) | 39 | 0.72 (0.7, 0.73) | -0.0317 | 0.7022 |
| ｐ103 | 42 | 0.74 (0.72, 0.76) | 39 | 0.72 (0.7, 0.74) | -0.0403 | 0.7022 |
| ｐ22874 | 42 | 0.65 (0.63, 0.68) | 39 | 0.63 (0.6, 0.65) | -0.0562 | 0.7022 |
| ｐ14772 | 42 | 1.16 (1.11, 1.21) | 39 | 1.12 (1.07, 1.17) | -0.0577 | 0.7022 |
| ｐ22907 | 42 | 0.46 (0.44, 0.48) | 39 | 0.44 (0.42, 0.46) | -0.0582 | 0.7022 |
| ｐ12084 | 42 | 0.37 (0.35, 0.38) | 39 | 0.35 (0.33, 0.37) | -0.0727 | 0.7022 |
| ｐ12453 | 42 | 0.5 (0.47, 0.53) | 39 | 0.48 (0.45, 0.5) | -0.0746 | 0.7022 |
| ｐ21895 | 42 | 0.26 (0.24, 0.28) | 39 | 0.24 (0.22, 0.26) | -0.0942 | 0.7022 |
| ｐ12935 | 42 | 0.76 (0.71, 0.82) | 39 | 0.72 (0.66, 0.77) | -0.0962 | 0.7022 |
| ｐ14957 | 42 | 0.32 (0.29, 0.34) | 39 | 0.29 (0.26, 0.32) | -0.1308 | 0.7022 |
| ｐ22927 | 42 | 0.17 (0.14, 0.2) | 39 | 0.14 (0.12, 0.17) | -0.2362 | 0.7022 |
| ｐ22903 | 42 | 0.23 (0.12, 0.34) | 39 | 0.12 (0.01, 0.24) | -0.8808 | 0.7022 |
| ｐ14842 | 42 | 0.7 (0.69, 0.71) | 39 | 0.69 (0.68, 0.7) | -0.0203 | 0.7022 |
| ｐ6904 | 42 | 0.61 (0.6, 0.62) | 39 | 0.6 (0.59, 0.61) | -0.0205 | 0.7022 |
| ｐ13258 | 42 | 1.17 (1.14, 1.2) | 39 | 1.14 (1.11, 1.17) | -0.0353 | 0.7022 |
| ｐ20871 | 42 | 1.07 (1.04, 1.1) | 39 | 1.04 (1.01, 1.07) | -0.0374 | 0.7022 |
| ｐ6372 | 42 | 0.59 (0.58, 0.61) | 39 | 0.58 (0.56, 0.59) | -0.0378 | 0.7022 |
| ｐ22911 | 42 | 1.03 (1, 1.07) | 39 | 1 (0.97, 1.04) | -0.0449 | 0.7022 |
| ｐ22639 | 42 | 0.71 (0.67, 0.74) | 39 | 0.67 (0.64, 0.71) | -0.0666 | 0.7022 |
| ｐ22644 | 42 | 0.67 (0.64, 0.71) | 39 | 0.64 (0.6, 0.68) | -0.0720 | 0.7022 |
| ｐ22660 | 42 | 0.88 (0.83, 0.93) | 39 | 0.83 (0.78, 0.89) | -0.0794 | 0.7022 |
| ｐ22538 | 42 | 0.47 (0.43, 0.5) | 39 | 0.44 (0.4, 0.47) | -0.0919 | 0.7022 |
| ｐ14790 | 42 | 1.14 (1, 1.28) | 39 | 1 (0.86, 1.15) | -0.1818 | 0.7022 |
| ｐ22897 | 42 | 0.2 (0.17, 0.23) | 39 | 0.17 (0.15, 0.2) | -0.1935 | 0.7022 |
| ｐ21627 | 42 | 0.76 (0.65, 0.87) | 39 | 0.66 (0.55, 0.77) | -0.2066 | 0.7022 |
| ｐ12982 | 42 | 0.63 (0.53, 0.73) | 39 | 0.54 (0.43, 0.64) | -0.2279 | 0.7022 |
| ｐ21352 | 42 | 0.09 (0.07, 0.11) | 39 | 0.07 (0.05, 0.09) | -0.3408 | 0.7022 |
| ｐ14707 | 42 | 0.86 (0.67, 1.06) | 39 | 0.68 (0.48, 0.88) | -0.3447 | 0.7022 |
| ｐ21412 | 42 | 0.19 (0.13, 0.24) | 39 | 0.14 (0.08, 0.19) | -0.4475 | 0.7022 |
| ｐ9810 | 42 | 0.17 (0.12, 0.22) | 39 | 0.12 (0.07, 0.17) | -0.4496 | 0.7022 |
| ｐ12697 | 42 | 0.4 (0.19, 0.61) | 39 | 0.2 (-0.02, 0.42) | -0.9831 | 0.7022 |
| ｐ22925 | 42 | 0.18 (0.06, 0.3) | 39 | 0.07 (-0.05, 0.19) | -1.3687 | 0.7022 |
| ｐ14895 | 42 | 0.8 (0.8, 0.81) | 39 | 0.8 (0.79, 0.8) | -0.0083 | 0.7022 |
| ｐ22876 | 42 | 0.83 (0.82, 0.85) | 39 | 0.82 (0.8, 0.83) | -0.0258 | 0.7022 |
| ｐ11124 | 42 | 0.15 (0.14, 0.17) | 39 | 0.14 (0.12, 0.16) | -0.1393 | 0.7022 |
| ｐ14896 | 42 | 0.15 (0.14, 0.17) | 39 | 0.14 (0.12, 0.16) | -0.1438 | 0.7022 |
| ｐ14869 | 42 | 0.15 (0.13, 0.17) | 39 | 0.13 (0.11, 0.15) | -0.1813 | 0.7022 |
| ｐ14714 | 42 | 0.46 (0.38, 0.54) | 39 | 0.38 (0.3, 0.47) | -0.2571 | 0.7022 |
| ｐ20290 | 42 | 0.27 (0.2, 0.33) | 39 | 0.21 (0.14, 0.27) | -0.3624 | 0.7022 |
| ｐ22663 | 42 | 0.18 (0.12, 0.25) | 39 | 0.12 (0.05, 0.19) | -0.5911 | 0.7022 |
| ｐ20179 | 42 | 0.12 (0.07, 0.18) | 39 | 0.07 (0.02, 0.13) | -0.7403 | 0.7022 |
| ｐ22894 | 42 | 0.09 (0.04, 0.14) | 39 | 0.04 (0, 0.09) | -0.9990 | 0.7022 |
| ｐ14708 | 42 | 0.33 (0.33, 0.33) | 39 | 0.33 (0.32, 0.33) | -0.0107 | 0.7022 |
| ｐ22668 | 42 | 2.08 (2.06, 2.1) | 39 | 2.06 (2.04, 2.08) | -0.0139 | 0.7022 |
| ｐ14857 | 42 | 1.53 (1.51, 1.55) | 39 | 1.51 (1.49, 1.53) | -0.0189 | 0.7022 |
| ｐ21116 | 42 | 1.08 (1.06, 1.1) | 39 | 1.07 (1.05, 1.08) | -0.0213 | 0.7022 |
| ｐ14693 | 42 | 0.69 (0.68, 0.7) | 39 | 0.68 (0.67, 0.69) | -0.0222 | 0.7022 |
| ｐ22931 | 42 | 0.82 (0.8, 0.84) | 39 | 0.8 (0.77, 0.82) | -0.0385 | 0.7022 |
| ｐ12506 | 42 | 0.62 (0.6, 0.64) | 39 | 0.6 (0.58, 0.62) | -0.0480 | 0.7022 |
| ｐ21817 | 42 | 0.34 (0.33, 0.35) | 39 | 0.33 (0.31, 0.34) | -0.0557 | 0.7022 |
| ｐ22374 | 42 | 0.53 (0.51, 0.56) | 39 | 0.51 (0.48, 0.54) | -0.0712 | 0.7022 |
| ｐ22921 | 42 | 0.41 (0.38, 0.43) | 39 | 0.39 (0.36, 0.41) | -0.0765 | 0.7022 |
| ｐ14692 | 42 | 1.47 (1.38, 1.56) | 39 | 1.39 (1.3, 1.48) | -0.0819 | 0.7022 |
| ｐ11629 | 42 | 0.68 (0.63, 0.74) | 39 | 0.63 (0.57, 0.69) | -0.1129 | 0.7022 |
| ｐ22661 | 42 | 0.4 (0.36, 0.44) | 39 | 0.36 (0.33, 0.4) | -0.1303 | 0.7022 |
| ｐ22551 | 42 | 0.43 (0.39, 0.47) | 39 | 0.39 (0.35, 0.43) | -0.1386 | 0.7022 |
| ｐ5030 | 42 | 0.29 (0.26, 0.33) | 39 | 0.26 (0.23, 0.3) | -0.1616 | 0.7022 |
| ｐ22572 | 42 | 0.2 (0.18, 0.23) | 39 | 0.18 (0.15, 0.2) | -0.1875 | 0.7022 |
| ｐ22641 | 42 | 0.62 (0.52, 0.71) | 39 | 0.53 (0.43, 0.63) | -0.2290 | 0.7022 |
| ｐ22893 | 42 | 0.38 (0.31, 0.45) | 39 | 0.32 (0.25, 0.39) | -0.2592 | 0.7022 |
| ｐ13383 | 42 | 0.64 (0.52, 0.75) | 39 | 0.53 (0.4, 0.65) | -0.2734 | 0.7022 |
| ｐ21133 | 42 | 0.24 (0.19, 0.3) | 39 | 0.2 (0.14, 0.25) | -0.3183 | 0.7022 |
| ｐ12769 | 42 | 0.29 (0.22, 0.36) | 39 | 0.23 (0.15, 0.3) | -0.3596 | 0.7022 |
| ｐ20840 | 42 | 0.35 (0.26, 0.44) | 39 | 0.27 (0.17, 0.36) | -0.4038 | 0.7022 |
| ｐ9705 | 42 | 0.14 (0.1, 0.19) | 39 | 0.1 (0.06, 0.15) | -0.4940 | 0.7022 |
| ｐ21719 | 42 | 0.06 (0.04, 0.08) | 39 | 0.04 (0.02, 0.06) | -0.5585 | 0.7022 |
| ｐ10087 | 42 | 0.07 (0.03, 0.1) | 39 | 0.03 (0, 0.07) | -0.9623 | 0.7022 |
| ｐ12446 | 42 | 0.07 (0.03, 0.1) | 39 | 0.03 (0, 0.07) | -0.9623 | 0.7022 |
| ｐ22896 | 42 | 0.03 (0.01, 0.04) | 39 | 0.01 (0, 0.03) | -0.9952 | 0.7022 |
| ｐ14798 | 42 | 0.1 (0.03, 0.16) | 39 | 0.04 (-0.03, 0.1) | -1.3516 | 0.7022 |
| ｐ8292 | 42 | 0.61 (0.6, 0.63) | 39 | 0.6 (0.59, 0.62) | -0.0305 | 0.7022 |
| ｐ5395 | 42 | 0.74 (0.72, 0.76) | 39 | 0.72 (0.7, 0.74) | -0.0357 | 0.7022 |
| ｐ12725 | 42 | 4.25 (4.13, 4.37) | 39 | 4.13 (4.01, 4.26) | -0.0392 | 0.7022 |
| ｐ14887 | 42 | 0.62 (0.6, 0.64) | 39 | 0.6 (0.58, 0.62) | -0.0419 | 0.7022 |
| ｐ20225 | 42 | 0.71 (0.69, 0.74) | 39 | 0.69 (0.66, 0.72) | -0.0529 | 0.7022 |
| ｐ14780 | 42 | 2.99 (2.87, 3.12) | 39 | 2.88 (2.75, 3.01) | -0.0581 | 0.7022 |
| ｐ14866 | 42 | 0.62 (0.59, 0.64) | 39 | 0.59 (0.56, 0.62) | -0.0619 | 0.7022 |
| ｐ21541 | 42 | 0.56 (0.53, 0.58) | 39 | 0.53 (0.51, 0.56) | -0.0629 | 0.7022 |
| ｐ22665 | 42 | 0.93 (0.88, 0.97) | 39 | 0.88 (0.84, 0.93) | -0.0682 | 0.7022 |
| ｐ14902 | 42 | 0.14 (0.13, 0.15) | 39 | 0.13 (0.12, 0.14) | -0.0686 | 0.7022 |
| ｐ21344 | 42 | 0.27 (0.25, 0.28) | 39 | 0.25 (0.24, 0.27) | -0.0834 | 0.7022 |
| ｐ12882 | 42 | 0.31 (0.29, 0.34) | 39 | 0.29 (0.27, 0.32) | -0.0988 | 0.7022 |
| ｐ22667 | 42 | 1.02 (0.94, 1.1) | 39 | 0.95 (0.87, 1.03) | -0.1066 | 0.7022 |
| ｐ22645 | 42 | 1.28 (1.17, 1.39) | 39 | 1.17 (1.05, 1.29) | -0.1266 | 0.7022 |
| ｐ20958 | 42 | 0.23 (0.2, 0.25) | 39 | 0.2 (0.18, 0.23) | -0.1481 | 0.7022 |
| ｐ14876 | 42 | 0.16 (0.15, 0.18) | 39 | 0.15 (0.13, 0.17) | -0.1557 | 0.7022 |
| ｐ12947 | 42 | 0.29 (0.24, 0.33) | 39 | 0.25 (0.2, 0.29) | -0.2157 | 0.7022 |
| ｐ14854 | 42 | 0.12 (0.11, 0.14) | 39 | 0.11 (0.09, 0.13) | -0.2192 | 0.7022 |
| ｐ22387 | 42 | 0.37 (0.3, 0.43) | 39 | 0.3 (0.23, 0.37) | -0.2781 | 0.7022 |
| ｐ22880 | 42 | 0.43 (0.34, 0.52) | 39 | 0.34 (0.25, 0.43) | -0.3172 | 0.7022 |
| ｐ14894 | 42 | 0.16 (0.12, 0.2) | 39 | 0.12 (0.08, 0.16) | -0.3866 | 0.7022 |
| ｐ10250 | 42 | 0.16 (0.11, 0.2) | 39 | 0.12 (0.07, 0.16) | -0.4464 | 0.7022 |
| ｐ14704 | 42 | 2.38 (2.35, 2.41) | 39 | 2.35 (2.31, 2.38) | -0.0189 | 0.7022 |
| ｐ23019 | 42 | 0.45 (0.44, 0.46) | 39 | 0.44 (0.43, 0.45) | -0.0318 | 0.7022 |
| ｐ22881 | 42 | 0.34 (0.3, 0.38) | 39 | 0.31 (0.27, 0.34) | -0.1524 | 0.7022 |
| ｐ11798 | 42 | 0.27 (0.21, 0.33) | 39 | 0.21 (0.15, 0.27) | -0.3450 | 0.7022 |
| ｐ13568 | 42 | 0.04 (0.01, 0.07) | 39 | 0.01 (-0.02, 0.04) | -2.0538 | 0.7022 |
| ｐ20883 | 42 | 0.36 (0.35, 0.38) | 39 | 0.36 (0.34, 0.37) | -0.0388 | 0.7022 |
| ｐ14826 | 42 | 0.69 (0.66, 0.72) | 39 | 0.67 (0.64, 0.69) | -0.0523 | 0.7022 |
| ｐ14419 | 42 | 0.35 (0.33, 0.38) | 39 | 0.33 (0.3, 0.35) | -0.0991 | 0.7022 |
| ｐ10435 | 42 | 0.44 (0.4, 0.47) | 39 | 0.4 (0.37, 0.44) | -0.1112 | 0.7022 |
| ｐ12863 | 42 | 0.39 (0.36, 0.43) | 39 | 0.36 (0.33, 0.4) | -0.1193 | 0.7022 |
| ｐ12972 | 42 | 0.16 (0.15, 0.17) | 39 | 0.15 (0.13, 0.16) | -0.1214 | 0.7022 |
| ｐ14808 | 42 | 0.31 (0.28, 0.34) | 39 | 0.28 (0.25, 0.31) | -0.1531 | 0.7022 |
| ｐ22480 | 42 | 0.74 (0.73, 0.75) | 39 | 0.73 (0.72, 0.74) | -0.0186 | 0.7022 |
| ｐ14743 | 42 | 0.21 (0.2, 0.22) | 39 | 0.2 (0.19, 0.21) | -0.0470 | 0.7022 |
| ｐ20694 | 42 | 0.46 (0.44, 0.48) | 39 | 0.45 (0.43, 0.46) | -0.0537 | 0.7022 |
| ｐ13794 | 42 | 0.32 (0.31, 0.34) | 39 | 0.31 (0.29, 0.32) | -0.0626 | 0.7022 |
| ｐ23073 | 42 | 0.48 (0.45, 0.5) | 39 | 0.46 (0.43, 0.48) | -0.0655 | 0.7022 |
| ｐ22898 | 42 | 0.27 (0.26, 0.29) | 39 | 0.26 (0.24, 0.28) | -0.0913 | 0.7022 |
| ｐ22870 | 42 | 0.61 (0.6, 0.63) | 39 | 0.6 (0.59, 0.62) | -0.0299 | 0.7022 |
| ｐ22918 | 42 | 2.74 (2.68, 2.8) | 39 | 2.68 (2.61, 2.75) | -0.0321 | 0.7022 |
| ｐ11608 | 42 | 4.27 (4.17, 4.38) | 39 | 4.17 (4.06, 4.28) | -0.0341 | 0.7022 |
| ｐ12989 | 42 | 0.69 (0.67, 0.72) | 39 | 0.67 (0.65, 0.7) | -0.0430 | 0.7022 |
| ｐ14703 | 42 | 1.31 (1.26, 1.36) | 39 | 1.26 (1.21, 1.31) | -0.0526 | 0.7022 |
| ｐ22885 | 42 | 0.64 (0.61, 0.67) | 39 | 0.62 (0.58, 0.65) | -0.0660 | 0.7022 |
| ｐ8019 | 42 | 0.49 (0.42, 0.56) | 39 | 0.43 (0.36, 0.5) | -0.1996 | 0.7022 |
| ｐ10534 | 42 | 0.65 (0.63, 0.67) | 39 | 0.63 (0.61, 0.65) | -0.0422 | 0.7022 |
| ｐ14889 | 42 | 0.25 (0.24, 0.26) | 39 | 0.24 (0.23, 0.25) | -0.0534 | 0.7022 |
| ｐ22879 | 42 | 0.51 (0.49, 0.53) | 39 | 0.49 (0.47, 0.51) | -0.0549 | 0.7022 |
| ｐ14694 | 42 | 0.85 (0.81, 0.89) | 39 | 0.81 (0.77, 0.85) | -0.0655 | 0.7022 |
| ｐ13060 | 42 | 0.4 (0.38, 0.43) | 39 | 0.38 (0.36, 0.41) | -0.0780 | 0.7022 |
| ｐ22869 | 42 | 0.55 (0.52, 0.59) | 39 | 0.52 (0.49, 0.56) | -0.0803 | 0.7022 |
| ｐ14882 | 42 | 0.74 (0.69, 0.79) | 39 | 0.69 (0.65, 0.74) | -0.0888 | 0.7022 |
| ｐ21501 | 42 | 0.31 (0.27, 0.34) | 39 | 0.27 (0.23, 0.31) | -0.1698 | 0.7022 |
| ｐ11435 | 42 | 1.1 (1.09, 1.12) | 39 | 1.09 (1.08, 1.11) | -0.0163 | 0.7022 |
| ｐ14900 | 42 | 2.86 (2.8, 2.93) | 39 | 2.8 (2.74, 2.87) | -0.0306 | 0.7022 |
| ｐ22888 | 42 | 0.66 (0.64, 0.67) | 39 | 0.64 (0.62, 0.66) | -0.0367 | 0.7022 |
| ｐ13041 | 42 | 0.75 (0.72, 0.78) | 39 | 0.72 (0.69, 0.75) | -0.0525 | 0.7022 |
| ｐ7099 | 42 | 0.61 (0.57, 0.64) | 39 | 0.57 (0.54, 0.61) | -0.0814 | 0.7022 |
| ｐ22878 | 42 | 0.45 (0.4, 0.49) | 39 | 0.41 (0.36, 0.45) | -0.1379 | 0.7022 |
| ｐ13178 | 42 | 0.22 (0.19, 0.25) | 39 | 0.2 (0.17, 0.22) | -0.1696 | 0.7022 |
| ｐ21200 | 42 | 0.89 (0.78, 1) | 39 | 0.79 (0.68, 0.9) | -0.1750 | 0.7022 |
| ｐ22892 | 42 | 0.94 (0.93, 0.95) | 39 | 0.93 (0.91, 0.94) | -0.0189 | 0.7022 |
| ｐ14873 | 42 | 0.79 (0.78, 0.81) | 39 | 0.78 (0.77, 0.79) | -0.0215 | 0.7022 |
| ｐ12584 | 42 | 0.84 (0.83, 0.86) | 39 | 0.83 (0.81, 0.84) | -0.0232 | 0.7022 |
| ｐ22664 | 42 | 0.62 (0.61, 0.63) | 39 | 0.61 (0.59, 0.62) | -0.0302 | 0.7022 |
| ｐ22900 | 42 | 0.49 (0.48, 0.5) | 39 | 0.48 (0.48, 0.49) | -0.0189 | 0.7022 |
| ｐ22877 | 42 | 1.09 (1.07, 1.11) | 39 | 1.08 (1.06, 1.09) | -0.0216 | 0.7022 |
| ｐ14410 | 42 | 0.46 (0.45, 0.47) | 39 | 0.45 (0.44, 0.46) | -0.0293 | 0.7022 |
| ｐ21750 | 42 | 1.96 (1.85, 2.07) | 39 | 1.86 (1.74, 1.97) | -0.0782 | 0.7022 |
| ｐ20120 | 42 | 0.5 (0.48, 0.53) | 39 | 0.48 (0.46, 0.51) | -0.0613 | 0.7022 |
| ｐ14843 | 42 | 0.48 (0.45, 0.52) | 39 | 0.45 (0.42, 0.49) | -0.0982 | 0.7022 |
| ｐ14833 | 42 | 0.91 (0.91, 0.92) | 39 | 0.91 (0.9, 0.91) | -0.0084 | 0.7022 |
| ｐ10241 | 42 | 0.56 (0.55, 0.56) | 39 | 0.55 (0.54, 0.56) | -0.0192 | 0.7022 |
| ｐ22905 | 42 | 0.3 (0.29, 0.31) | 39 | 0.29 (0.29, 0.3) | -0.0280 | 0.7022 |
| ｐ14717 | 42 | 0.63 (0.62, 0.65) | 39 | 0.62 (0.6, 0.64) | -0.0362 | 0.7022 |
| ｐ22908 | 42 | 1 (0.99, 1.02) | 39 | 0.99 (0.97, 1) | -0.0194 | 0.7022 |
| ｐ14878 | 42 | 0.65 (0.64, 0.66) | 39 | 0.64 (0.63, 0.65) | -0.0215 | 0.7022 |
| ｐ22550 | 42 | 0.91 (0.91, 0.92) | 39 | 0.91 (0.9, 0.91) | -0.0071 | 0.7022 |
| ｐ14750 | 42 | 2.75 (2.71, 2.8) | 39 | 2.71 (2.66, 2.76) | -0.0235 | 0.7022 |
| ｐ14623 | 42 | 1.06 (1.04, 1.09) | 39 | 1.04 (1.01, 1.07) | -0.0341 | 0.7022 |
| ｐ22915 | 42 | 0.81 (0.8, 0.82) | 39 | 0.81 (0.8, 0.81) | -0.0101 | 0.7022 |
| ｐ1741 | 42 | 0.22 (0.2, 0.24) | 39 | 0.2 (0.19, 0.22) | -0.1136 | 0.7022 |
| ｐ20400 | 42 | 0.75 (0.74, 0.75) | 39 | 0.74 (0.74, 0.75) | -0.0099 | 0.7022 |
| ｐ22861 | 42 | 0.46 (0.46, 0.47) | 39 | 0.46 (0.46, 0.46) | -0.0077 | 0.7022 |
| ｐ14884 | 42 | 0.29 (0.29, 0.3) | 39 | 0.29 (0.28, 0.29) | -0.0206 | 0.7022 |
| ｐ14844 | 42 | 0.69 (0.68, 0.7) | 39 | 0.68 (0.68, 0.69) | -0.0154 | 0.7022 |
| ｐ12794 | 42 | 4.41 (4.35, 4.47) | 39 | 4.36 (4.29, 4.42) | -0.0185 | 0.7022 |
| ｐ20615 | 42 | 0.81 (0.8, 0.83) | 39 | 0.8 (0.78, 0.81) | -0.0238 | 0.7022 |
| ｐ22917 | 42 | 0.97 (0.96, 0.99) | 39 | 0.96 (0.95, 0.98) | -0.0149 | 0.7022 |
| ｐ22873 | 42 | 1.26 (1.25, 1.27) | 39 | 1.25 (1.24, 1.26) | -0.0128 | 0.7022 |
| ｐ20035 | 42 | 0.51 (0.51, 0.52) | 39 | 0.51 (0.5, 0.51) | -0.0151 | 0.7022 |
| ｐ14816 | 42 | 1 (0.98, 1.03) | 39 | 0.98 (0.95, 1.01) | -0.0348 | 0.7022 |
| ｐ2995 | 42 | 0.92 (0.9, 0.94) | 39 | 0.9 (0.88, 0.92) | -0.0301 | 0.7022 |
| ｐ22643 | 42 | 0.9 (0.89, 0.91) | 39 | 0.89 (0.87, 0.9) | -0.0176 | 0.7022 |
| ｐ21608 | 42 | 0.27 (0.27, 0.27) | 39 | 0.27 (0.26, 0.27) | -0.0119 | 0.7022 |
| ｐ14796 | 42 | 0.59 (0.58, 0.6) | 39 | 0.58 (0.57, 0.59) | -0.0230 | 0.7022 |
| ｐ22904 | 42 | 0.39 (0.38, 0.39) | 39 | 0.38 (0.38, 0.39) | -0.0079 | 0.7022 |
| ｐ20119 | 42 | 0.76 (0.75, 0.77) | 39 | 0.75 (0.74, 0.76) | -0.0175 | 0.7022 |
| ｐ14802 | 42 | 0.67 (0.67, 0.68) | 39 | 0.67 (0.67, 0.67) | -0.0084 | 0.7022 |
| ｐ20134 | 42 | 1.16 (1.16, 1.16) | 39 | 1.16 (1.15, 1.16) | -0.0032 | 0.7022 |
| ｐ21916 | 42 | 0.91 (0.9, 0.91) | 39 | 0.9 (0.89, 0.91) | -0.0117 | 0.7022 |
| ｐ22914 | 42 | 1.05 (1.05, 1.05) | 39 | 1.05 (1.05, 1.05) | -0.0029 | 0.7022 |
| ｐ21662 | 42 | 1.39 (1.39, 1.39) | 39 | 1.39 (1.39, 1.39) | -0.0029 | 0.7022 |
| ｐ22481 | 42 | 0.95 (0.94, 0.95) | 39 | 0.94 (0.94, 0.95) | -0.0063 | 0.7022 |
| ｐ14832 | 42 | 1.69 (1.67, 1.7) | 39 | 1.67 (1.66, 1.69) | -0.0111 | 0.7022 |
| ｐ21214 | 42 | 0.9 (0.9, 0.9) | 39 | 0.9 (0.9, 0.9) | -0.0029 | 0.7022 |
| ｐ13846 | 42 | 0.84 (0.84, 0.84) | 39 | 0.84 (0.84, 0.84) | -0.0008 | 0.7022 |
| ｐ21207 | 42 | 1.1 (1.09, 1.1) | 39 | 1.09 (1.09, 1.1) | -0.0028 | 0.7022 |
| ｐ22886 | 42 | 0.29 (0.29, 0.29) | 39 | 0.29 (0.29, 0.29) | -0.0013 | 0.7022 |
| ｐ14765 | 42 | 0.8 (0.8, 0.8) | 39 | 0.8 (0.8, 0.8) | -0.0016 | 0.7022 |
| ｐ14686 | 42 | 1.96 (1.96, 1.96) | 39 | 1.96 (1.96, 1.96) | -0.0006 | 0.7022 |
| ｐ17483 | 42 | 0.3 (0.2, 0.39) | 39 | 0.38 (0.29, 0.48) | 0.3770 | 0.7020 |
| ｐ18600 | 42 | 0.66 (0.64, 0.69) | 39 | 0.64 (0.62, 0.67) | -0.0451 | 0.7020 |
| ｐ7714 | 42 | 0.3 (0.2, 0.39) | 39 | 0.38 (0.29, 0.48) | 0.3770 | 0.7020 |
| ｐ15205 | 42 | 0.66 (0.55, 0.78) | 39 | 0.56 (0.44, 0.67) | -0.2501 | 0.7009 |
| ｐ22895 | 42 | 0.54 (0.48, 0.59) | 39 | 0.48 (0.43, 0.54) | -0.1432 | 0.7007 |
| ｐ11465 | 42 | 0.52 (0.43, 0.62) | 39 | 0.43 (0.33, 0.53) | -0.2708 | 0.6989 |
| ｐ11172 | 42 | 0.12 (0.11, 0.13) | 39 | 0.13 (0.12, 0.14) | 0.1351 | 0.6986 |
| ｐ11866 | 42 | 0.09 (0.05, 0.14) | 39 | 0.05 (0.01, 0.1) | -0.7804 | 0.6973 |
| ｐ11948 | 42 | 0.79 (0.54, 1.03) | 39 | 0.56 (0.31, 0.81) | -0.4898 | 0.6967 |
| ｐ18247 | 42 | 0.31 (0.23, 0.39) | 39 | 0.24 (0.15, 0.32) | -0.3997 | 0.6966 |
| ｐ20052 | 42 | 0.77 (0.64, 0.91) | 39 | 0.9 (0.76, 1.04) | 0.2187 | 0.6955 |
| ｐ16122 | 42 | 0.23 (-0.22, 0.69) | 39 | 0.66 (0.19, 1.14) | 1.4981 | 0.6951 |
| ｐ12338 | 42 | 0.39 (0.23, 0.56) | 39 | 0.24 (0.07, 0.41) | -0.7222 | 0.6949 |
| ｐ15118 | 42 | 0.29 (0.17, 0.41) | 39 | 0.18 (0.06, 0.3) | -0.6931 | 0.6942 |
| ｐ15170 | 42 | 0.11 (-0.01, 0.22) | 39 | 0 (-0.12, 0.12) | NA | 0.6938 |
| ｐ21659 | 42 | 0.65 (0.53, 0.77) | 39 | 0.76 (0.63, 0.88) | 0.2296 | 0.6936 |
| ｐ15192 | 42 | 0.38 (0.3, 0.47) | 39 | 0.31 (0.23, 0.39) | -0.3120 | 0.6934 |
| ｐ16237 | 42 | 0.42 (0.16, 0.67) | 39 | 0.65 (0.39, 0.92) | 0.6555 | 0.6929 |
| ｐ15549 | 42 | 0.38 (0.35, 0.4) | 39 | 0.4 (0.38, 0.43) | 0.0913 | 0.6919 |
| ｐ14483 | 42 | 0.49 (0.25, 0.72) | 39 | 0.71 (0.46, 0.95) | 0.5307 | 0.6914 |
| ｐ15542 | 42 | 0.35 (0.17, 0.53) | 39 | 0.52 (0.33, 0.71) | 0.5638 | 0.6893 |
| ｐ18826 | 42 | 0.27 (0.2, 0.34) | 39 | 0.33 (0.26, 0.41) | 0.3069 | 0.6893 |
| ｐ4934 | 42 | 0.37 (0.19, 0.55) | 39 | 0.2 (0.02, 0.39) | -0.8680 | 0.6893 |
| ｐ22956 | 42 | 0.99 (0.42, 1.57) | 39 | 0.46 (-0.13, 1.06) | -1.0988 | 0.6891 |
| ｐ22486 | 42 | 0.68 (0.51, 0.84) | 39 | 0.52 (0.35, 0.7) | -0.3731 | 0.6890 |
| ｐ22958 | 42 | 0.13 (0.04, 0.23) | 39 | 0.22 (0.12, 0.33) | 0.7373 | 0.6876 |
| ｐ16760 | 42 | 0.39 (0.26, 0.52) | 39 | 0.27 (0.14, 0.41) | -0.5185 | 0.6863 |
| ｐ20500 | 42 | 0.58 (-0.25, 1.4) | 39 | 1.34 (0.48, 2.2) | 1.2169 | 0.6846 |
| ｐ11584 | 42 | 0.12 (0.04, 0.2) | 39 | 0.05 (-0.03, 0.13) | -1.3218 | 0.6806 |
| ｐ22093 | 42 | 0 (-0.06, 0.06) | 39 | 0.05 (-0.01, 0.11) | NA | 0.6803 |
| ｐ15267 | 42 | 0.29 (0.24, 0.35) | 39 | 0.24 (0.18, 0.3) | -0.2856 | 0.6761 |
| ｐ22069 | 41 | 0.55 (0.52, 0.59) | 36 | 0.52 (0.48, 0.56) | -0.0958 | 0.6750 |
| ｐ14892 | 42 | 0.18 (0.06, 0.31) | 39 | 0.3 (0.17, 0.43) | 0.6945 | 0.6716 |
| ｐ15499 | 42 | 0.4 (0.29, 0.5) | 39 | 0.49 (0.39, 0.6) | 0.3083 | 0.6708 |
| ｐ17326 | 42 | 0.6 (0.4, 0.81) | 39 | 0.79 (0.58, 1) | 0.3838 | 0.6696 |
| ｐ14691 | 42 | 0.1 (-0.01, 0.21) | 39 | 0 (-0.11, 0.11) | NA | 0.6695 |
| ｐ22510 | 42 | 0.27 (0.16, 0.37) | 39 | 0.36 (0.25, 0.47) | 0.4341 | 0.6690 |
| ｐ18298 | 42 | 0.02 (0, 0.04) | 39 | 0 (-0.02, 0.02) | NA | 0.6690 |
| ｐ11014 | 42 | 0.9 (0.18, 1.63) | 39 | 0.25 (-0.5, 1) | -1.8597 | 0.6674 |
| ｐ18265 | 42 | 0.61 (0.34, 0.89) | 39 | 0.86 (0.58, 1.15) | 0.4930 | 0.6670 |
| ｐ20616 | 42 | 0.75 (0.69, 0.8) | 39 | 0.8 (0.74, 0.86) | 0.0986 | 0.6668 |
| ｐ15300 | 42 | 0.38 (0.3, 0.46) | 39 | 0.3 (0.22, 0.39) | -0.3071 | 0.6659 |
| ｐ10716 | 42 | 0.74 (0.63, 0.85) | 39 | 0.84 (0.73, 0.95) | 0.1827 | 0.6652 |
| ｐ15221 | 42 | 0.06 (0.04, 0.09) | 39 | 0.04 (0.01, 0.06) | -0.6915 | 0.6647 |
| ｐ15656 | 42 | 0.52 (0.37, 0.67) | 39 | 0.38 (0.23, 0.54) | -0.4372 | 0.6635 |
| ｐ108 | 42 | 1.82 (0.67, 2.97) | 39 | 2.85 (1.66, 4.05) | 0.6502 | 0.6623 |
| ｐ19433 | 42 | 0.18 (0.14, 0.22) | 39 | 0.22 (0.17, 0.26) | 0.2803 | 0.6619 |
| ｐ944 | 42 | 0.57 (0.53, 0.61) | 39 | 0.61 (0.57, 0.65) | 0.0933 | 0.6603 |
| ｐ15712 | 42 | 0.12 (0.03, 0.21) | 39 | 0.2 (0.11, 0.29) | 0.7205 | 0.6596 |
| ｐ11242 | 42 | 1.41 (0.68, 2.15) | 39 | 0.75 (-0.01, 1.52) | -0.9067 | 0.6585 |
| ｐ20045 | 42 | 0.44 (0.29, 0.59) | 39 | 0.57 (0.42, 0.73) | 0.3856 | 0.6573 |
| ｐ16748 | 42 | 0.14 (0.07, 0.21) | 39 | 0.2 (0.13, 0.28) | 0.5317 | 0.6570 |
| ｐ22099 | 42 | 0.44 (0.32, 0.56) | 39 | 0.55 (0.42, 0.67) | 0.3134 | 0.6564 |
| ｐ22722 | 42 | 0.28 (-0.02, 0.59) | 39 | 0.56 (0.24, 0.87) | 0.9686 | 0.6556 |
| ｐ14791 | 42 | 0.72 (-0.99, 2.43) | 39 | 2.25 (0.47, 4.02) | 1.6390 | 0.6551 |
| ｐ15948 | 42 | 0.47 (0.31, 0.63) | 39 | 0.61 (0.45, 0.77) | 0.3749 | 0.6535 |
| ｐ22700 | 42 | 0.32 (-0.13, 0.77) | 39 | 0.72 (0.25, 1.18) | 1.1639 | 0.6534 |
| ｐ13854 | 42 | 0.21 (0.14, 0.29) | 39 | 0.15 (0.07, 0.22) | -0.5343 | 0.6518 |
| ｐ22047 | 42 | 0.18 (0.08, 0.27) | 39 | 0.26 (0.16, 0.35) | 0.5497 | 0.6509 |
| ｐ10772 | 42 | 0.33 (0.23, 0.43) | 39 | 0.42 (0.32, 0.52) | 0.3395 | 0.6503 |
| ｐ22949 | 42 | 0.55 (-0.63, 1.73) | 39 | 1.6 (0.37, 2.82) | 1.5361 | 0.6503 |
| ｐ21855 | 42 | 0.48 (0.33, 0.62) | 39 | 0.6 (0.45, 0.75) | 0.3443 | 0.6498 |
| ｐ15082 | 42 | 0.48 (0.33, 0.62) | 39 | 0.6 (0.45, 0.75) | 0.3443 | 0.6498 |
| ｐ14817 | 42 | 0.04 (-0.17, 0.25) | 39 | 0.23 (0.01, 0.45) | 2.3982 | 0.6495 |
| ｐ22053 | 42 | 0.14 (0.1, 0.17) | 39 | 0.16 (0.13, 0.2) | 0.2710 | 0.6493 |
| ｐ14947 | 42 | 0.62 (0.53, 0.7) | 39 | 0.54 (0.45, 0.63) | -0.1906 | 0.6479 |
| ｐ13963 | 42 | 0.47 (-0.06, 0.99) | 39 | 0.93 (0.38, 1.48) | 1.0014 | 0.6474 |
| ｐ15223 | 42 | 0.09 (0.05, 0.14) | 39 | 0.06 (0.01, 0.1) | -0.7487 | 0.6463 |
| ｐ20592 | 42 | 0.27 (0.24, 0.31) | 39 | 0.3 (0.27, 0.34) | 0.1526 | 0.6461 |
| ｐ6282 | 42 | 0.6 (0.5, 0.71) | 39 | 0.51 (0.4, 0.62) | -0.2478 | 0.6456 |
| ｐ18418 | 42 | 0.45 (0.33, 0.57) | 39 | 0.56 (0.43, 0.68) | 0.3059 | 0.6451 |
| ｐ20669 | 42 | 0.69 (0.66, 0.73) | 39 | 0.72 (0.69, 0.76) | 0.0637 | 0.6443 |
| ｐ10232 | 42 | 1.26 (0.45, 2.06) | 39 | 0.55 (-0.29, 1.38) | -1.2007 | 0.6429 |
| ｐ22694 | 42 | 0.31 (0.01, 0.62) | 39 | 0.58 (0.26, 0.9) | 0.8974 | 0.6423 |
| ｐ14867 | 42 | 0.35 (0.23, 0.48) | 39 | 0.24 (0.11, 0.37) | -0.5442 | 0.6413 |
| ｐ2832 | 42 | 0.8 (0.75, 0.84) | 39 | 0.75 (0.71, 0.8) | -0.0783 | 0.6411 |
| ｐ16297 | 42 | 0.27 (0.25, 0.3) | 39 | 0.25 (0.22, 0.28) | -0.1386 | 0.6407 |
| ｐ16780 | 42 | 0.27 (0.24, 0.3) | 39 | 0.24 (0.21, 0.27) | -0.1390 | 0.6402 |
| ｐ19177 | 42 | 0.37 (0.33, 0.41) | 39 | 0.33 (0.29, 0.37) | -0.1392 | 0.6400 |
| ｐ22561 | 42 | 1.27 (1.13, 1.4) | 39 | 1.15 (1.01, 1.29) | -0.1395 | 0.6395 |
| ｐ10955 | 42 | 0.3 (0.17, 0.42) | 39 | 0.19 (0.06, 0.32) | -0.6662 | 0.6394 |
| ｐ16117 | 42 | 0.56 (0.43, 0.69) | 39 | 0.44 (0.31, 0.58) | -0.3328 | 0.6394 |
| ｐ13728 | 42 | 0.06 (-0.01, 0.13) | 39 | 0 (-0.07, 0.07) | NA | 0.6392 |
| ｐ14845 | 42 | 0.27 (0.24, 0.3) | 39 | 0.25 (0.22, 0.28) | -0.1398 | 0.6391 |
| ｐ14784 | 42 | 0.5 (0.44, 0.55) | 39 | 0.45 (0.4, 0.51) | -0.1398 | 0.6391 |
| ｐ16719 | 42 | 0.28 (0.11, 0.46) | 39 | 0.44 (0.26, 0.62) | 0.6270 | 0.6388 |
| ｐ20523 | 42 | 0.84 (-0.06, 1.73) | 39 | 1.62 (0.69, 2.55) | 0.9519 | 0.6386 |
| ｐ14825 | 42 | 0.31 (0.28, 0.35) | 39 | 0.28 (0.25, 0.32) | -0.1405 | 0.6382 |
| ｐ6427 | 42 | 0.08 (0.07, 0.1) | 39 | 0.07 (0.05, 0.09) | -0.3160 | 0.6378 |
| ｐ15653 | 42 | 0.75 (0.67, 0.83) | 39 | 0.68 (0.59, 0.76) | -0.1409 | 0.6377 |
| ｐ21923 | 42 | 0.42 (0.37, 0.46) | 39 | 0.38 (0.33, 0.42) | -0.1410 | 0.6376 |
| ｐ16454 | 42 | 0.18 (0.16, 0.2) | 39 | 0.16 (0.14, 0.18) | -0.1412 | 0.6373 |
| ｐ15868 | 42 | 1.2 (0.84, 1.55) | 39 | 0.88 (0.51, 1.25) | -0.4355 | 0.6368 |
| ｐ14733 | 42 | 0.26 (0.23, 0.28) | 39 | 0.23 (0.2, 0.26) | -0.1421 | 0.6361 |
| ｐ17429 | 42 | 0.57 (0.51, 0.64) | 39 | 0.52 (0.46, 0.58) | -0.1421 | 0.6360 |
| ｐ21664 | 42 | 0.26 (0.19, 0.33) | 39 | 0.33 (0.25, 0.4) | 0.3043 | 0.6359 |
| ｐ4748 | 42 | 0.52 (0.43, 0.62) | 39 | 0.6 (0.51, 0.7) | 0.2090 | 0.6355 |
| ｐ17427 | 42 | 0.16 (0.14, 0.18) | 39 | 0.15 (0.13, 0.17) | -0.1431 | 0.6349 |
| ｐ10704 | 42 | 0.27 (0.24, 0.3) | 39 | 0.25 (0.22, 0.28) | -0.1432 | 0.6348 |
| ｐ15329 | 42 | 1.02 (0.75, 1.29) | 39 | 0.78 (0.5, 1.06) | -0.3832 | 0.6341 |
| ｐ14872 | 42 | 0.22 (0.19, 0.24) | 39 | 0.2 (0.17, 0.22) | -0.1437 | 0.6340 |
| ｐ22636 | 42 | 0.23 (0.2, 0.25) | 39 | 0.21 (0.18, 0.23) | -0.1441 | 0.6336 |
| ｐ14744 | 42 | 0.32 (0.29, 0.36) | 39 | 0.29 (0.26, 0.33) | -0.1447 | 0.6328 |
| ｐ12866 | 42 | 0.58 (0.38, 0.78) | 39 | 0.41 (0.2, 0.61) | -0.5121 | 0.6315 |
| ｐ14893 | 42 | 0.17 (0.15, 0.19) | 39 | 0.15 (0.13, 0.17) | -0.1459 | 0.6314 |
| ｐ22713 | 42 | 0.77 (0.08, 1.46) | 39 | 1.37 (0.65, 2.08) | 0.8330 | 0.6308 |
| ｐ15458 | 42 | 0.45 (0.4, 0.51) | 39 | 0.5 (0.45, 0.56) | 0.1410 | 0.6308 |
| ｐ14789 | 42 | 0.27 (0.24, 0.3) | 39 | 0.24 (0.21, 0.27) | -0.1465 | 0.6306 |
| ｐ22889 | 42 | 0.25 (0.22, 0.28) | 39 | 0.22 (0.2, 0.25) | -0.1471 | 0.6299 |
| ｐ14874 | 42 | 0.18 (0.16, 0.2) | 39 | 0.16 (0.14, 0.18) | -0.1472 | 0.6298 |
| ｐ12209 | 42 | 0.19 (0.17, 0.22) | 39 | 0.17 (0.15, 0.2) | -0.1472 | 0.6298 |
| ｐ2882 | 42 | 0.35 (0.31, 0.39) | 39 | 0.32 (0.28, 0.36) | -0.1478 | 0.6290 |
| ｐ11052 | 42 | 0.62 (0.37, 0.86) | 39 | 0.83 (0.57, 1.08) | 0.4238 | 0.6285 |
| ｐ21946 | 42 | 0.3 (0.27, 0.34) | 39 | 0.27 (0.24, 0.31) | -0.1487 | 0.6280 |
| ｐ22731 | 42 | 0.33 (-0.36, 1.02) | 39 | 0.92 (0.21, 1.64) | 1.4905 | 0.6263 |
| ｐ14698 | 42 | 2.86 (2.53, 3.18) | 39 | 2.57 (2.23, 2.91) | -0.1503 | 0.6261 |
| ｐ16766 | 42 | 0.33 (0.28, 0.39) | 39 | 0.28 (0.22, 0.34) | -0.2366 | 0.6253 |
| ｐ15014 | 42 | 0.14 (0.09, 0.2) | 39 | 0.1 (0.04, 0.15) | -0.5701 | 0.6253 |
| ｐ22920 | 42 | 0.6 (0.53, 0.66) | 39 | 0.54 (0.47, 0.61) | -0.1513 | 0.6250 |
| ｐ8149 | 42 | 0.71 (0.55, 0.87) | 39 | 0.57 (0.4, 0.74) | -0.3173 | 0.6246 |
| ｐ18996 | 42 | 0.27 (0.24, 0.3) | 39 | 0.24 (0.21, 0.27) | -0.1519 | 0.6243 |
| ｐ14542 | 42 | 0.27 (0.24, 0.3) | 39 | 0.24 (0.21, 0.27) | -0.1519 | 0.6243 |
| ｐ21934 | 42 | 0.4 (0.28, 0.53) | 39 | 0.29 (0.16, 0.43) | -0.4591 | 0.6236 |
| ｐ12197 | 42 | 1.11 (0.51, 1.71) | 39 | 0.59 (-0.03, 1.22) | -0.9039 | 0.6229 |
| ｐ19071 | 42 | 0.49 (0.43, 0.55) | 39 | 0.44 (0.38, 0.5) | -0.1534 | 0.6227 |
| ｐ14728 | 42 | 0.29 (0.26, 0.33) | 39 | 0.26 (0.23, 0.3) | -0.1534 | 0.6226 |
| ｐ21721 | 42 | 0.37 (0.33, 0.41) | 39 | 0.4 (0.36, 0.44) | 0.1185 | 0.6226 |
| ｐ22040 | 42 | 0.85 (0.81, 0.88) | 39 | 0.88 (0.84, 0.91) | 0.0512 | 0.6224 |
| ｐ16127 | 42 | 0.72 (0.64, 0.81) | 39 | 0.65 (0.56, 0.74) | -0.1543 | 0.6216 |
| ｐ14911 | 42 | 0.2 (0.11, 0.28) | 39 | 0.27 (0.18, 0.36) | 0.4643 | 0.6216 |
| ｐ15469 | 42 | 0.33 (0.27, 0.39) | 39 | 0.38 (0.32, 0.44) | 0.2008 | 0.6214 |
| ｐ22100 | 42 | 0.02 (0, 0.05) | 39 | 0 (-0.02, 0.03) | -3.1099 | 0.6209 |
| ｐ22322 | 42 | 0.55 (0.46, 0.64) | 39 | 0.47 (0.38, 0.57) | -0.2215 | 0.6206 |
| ｐ15816 | 41 | 0.29 (0.25, 0.33) | 39 | 0.26 (0.22, 0.3) | -0.1691 | 0.6205 |
| ｐ22936 | 42 | 0.56 (0.25, 0.87) | 39 | 0.82 (0.5, 1.15) | 0.5637 | 0.6204 |
| ｐ14208 | 42 | 0.6 (0.53, 0.67) | 39 | 0.54 (0.46, 0.61) | -0.1555 | 0.6203 |
| ｐ15337 | 42 | 0.03 (0, 0.06) | 39 | 0 (-0.03, 0.03) | NA | 0.6198 |
| ｐ9832 | 42 | 0.26 (0.12, 0.4) | 39 | 0.38 (0.23, 0.52) | 0.5451 | 0.6194 |
| ｐ6271 | 42 | 0.36 (0.32, 0.4) | 39 | 0.32 (0.28, 0.37) | -0.1567 | 0.6191 |
| ｐ15237 | 42 | 0.14 (0.05, 0.23) | 39 | 0.06 (-0.03, 0.16) | -1.1296 | 0.6185 |
| ｐ16014 | 42 | 0.24 (0.16, 0.32) | 39 | 0.18 (0.09, 0.26) | -0.4767 | 0.6183 |
| ｐ20140 | 42 | 1.24 (1.09, 1.39) | 39 | 1.11 (0.96, 1.27) | -0.1577 | 0.6180 |
| ｐ8022 | 42 | 1.29 (0.97, 1.61) | 39 | 1.02 (0.69, 1.35) | -0.3432 | 0.6170 |
| ｐ18355 | 42 | 1 (0.54, 1.46) | 39 | 0.61 (0.13, 1.08) | -0.7153 | 0.6167 |
| ｐ10635 | 42 | 0.42 (0.37, 0.47) | 39 | 0.38 (0.32, 0.43) | -0.1595 | 0.6161 |
| ｐ12719 | 42 | 0.24 (0.14, 0.33) | 39 | 0.32 (0.22, 0.42) | 0.4422 | 0.6158 |
| ｐ12590 | 42 | 0.46 (-0.03, 0.96) | 39 | 0.88 (0.37, 1.4) | 0.9330 | 0.6147 |
| ｐ16135 | 42 | 1 (0.88, 1.12) | 39 | 0.89 (0.77, 1.02) | -0.1610 | 0.6146 |
| ｐ14922 | 42 | 0.34 (0.01, 0.66) | 39 | 0.61 (0.28, 0.95) | 0.8605 | 0.6145 |
| ｐ10474 | 42 | 0.32 (0.28, 0.36) | 39 | 0.29 (0.25, 0.33) | -0.1614 | 0.6141 |
| ｐ14002 | 42 | 0.13 (0.02, 0.23) | 39 | 0.21 (0.11, 0.32) | 0.7657 | 0.6140 |
| ｐ21776 | 42 | 0.36 (0.31, 0.4) | 39 | 0.32 (0.27, 0.36) | -0.1628 | 0.6128 |
| ｐ20622 | 42 | 0.38 (0.33, 0.42) | 39 | 0.34 (0.29, 0.39) | -0.1628 | 0.6128 |
| ｐ11164 | 42 | 0.05 (-0.02, 0.12) | 39 | 0.11 (0.04, 0.19) | 1.1274 | 0.6128 |
| ｐ18796 | 42 | 0.38 (0.33, 0.43) | 39 | 0.34 (0.29, 0.39) | -0.1633 | 0.6123 |
| ｐ12099 | 42 | 0.21 (0.08, 0.33) | 39 | 0.1 (-0.03, 0.23) | -1.0463 | 0.6112 |
| ｐ14757 | 42 | 0.29 (0.25, 0.32) | 39 | 0.25 (0.22, 0.29) | -0.1648 | 0.6108 |
| ｐ20020 | 42 | 0.32 (0.28, 0.36) | 39 | 0.28 (0.24, 0.32) | -0.1651 | 0.6106 |
| ｐ22516 | 42 | 0.32 (0.25, 0.38) | 39 | 0.37 (0.31, 0.44) | 0.2259 | 0.6102 |
| ｐ22801 | 42 | 0.8 (0.3, 1.31) | 39 | 0.38 (-0.15, 0.9) | -1.0915 | 0.6094 |
| ｐ22049 | 42 | 0.31 (0.28, 0.34) | 39 | 0.34 (0.31, 0.37) | 0.1156 | 0.6093 |
| ｐ14858 | 42 | 0.68 (0.5, 0.86) | 39 | 0.53 (0.35, 0.72) | -0.3623 | 0.6089 |
| ｐ21558 | 42 | 1.06 (0.92, 1.2) | 39 | 0.94 (0.8, 1.09) | -0.1677 | 0.6081 |
| ｐ5380 | 42 | 0.74 (0.6, 0.89) | 39 | 0.62 (0.47, 0.77) | -0.2650 | 0.6073 |
| ｐ17015 | 42 | 0.22 (0.2, 0.25) | 39 | 0.2 (0.17, 0.23) | -0.1687 | 0.6071 |
| ｐ15523 | 42 | 0.2 (0.15, 0.26) | 39 | 0.25 (0.19, 0.3) | 0.2959 | 0.6068 |
| ｐ19104 | 42 | 0.48 (0.42, 0.54) | 39 | 0.43 (0.36, 0.49) | -0.1692 | 0.6066 |
| ｐ13045 | 42 | 0.7 (0.55, 0.85) | 39 | 0.82 (0.67, 0.98) | 0.2342 | 0.6065 |
| ｐ22024 | 42 | 0.58 (0.53, 0.63) | 39 | 0.62 (0.57, 0.68) | 0.1032 | 0.6052 |
| ｐ14131 | 42 | 0.4 (0.14, 0.66) | 39 | 0.62 (0.35, 0.89) | 0.6324 | 0.6052 |
| ｐ22151 | 42 | 0.53 (0.41, 0.66) | 39 | 0.43 (0.3, 0.56) | -0.3193 | 0.6050 |
| ｐ15102 | 42 | 0.55 (0.47, 0.62) | 39 | 0.49 (0.41, 0.56) | -0.1712 | 0.6048 |
| ｐ22794 | 42 | 0.58 (-0.74, 1.91) | 39 | 1.7 (0.32, 3.08) | 1.5437 | 0.6045 |
| ｐ13162 | 42 | 0.32 (0.27, 0.36) | 39 | 0.28 (0.24, 0.33) | -0.1720 | 0.6042 |
| ｐ15408 | 42 | 0.6 (0.52, 0.68) | 39 | 0.53 (0.45, 0.62) | -0.1720 | 0.6041 |
| ｐ11928 | 42 | 0.15 (0.13, 0.17) | 39 | 0.14 (0.11, 0.16) | -0.1730 | 0.6033 |
| ｐ4592 | 42 | 0.52 (0.44, 0.61) | 39 | 0.45 (0.36, 0.54) | -0.2138 | 0.6033 |
| ｐ11552 | 42 | 0.32 (0.28, 0.37) | 39 | 0.29 (0.24, 0.33) | -0.1732 | 0.6031 |
| ｐ22750 | 42 | 0.27 (0.12, 0.41) | 39 | 0.14 (-0.01, 0.3) | -0.8844 | 0.6028 |
| ｐ15148 | 42 | 0.52 (0.45, 0.59) | 39 | 0.46 (0.38, 0.53) | -0.1751 | 0.6015 |
| ｐ11804 | 42 | 0.7 (0.6, 0.79) | 39 | 0.62 (0.52, 0.71) | -0.1756 | 0.6011 |
| ｐ22003 | 42 | 0.56 (0.38, 0.74) | 39 | 0.71 (0.53, 0.9) | 0.3451 | 0.6005 |
| ｐ8227 | 42 | 0.32 (0.13, 0.51) | 39 | 0.16 (-0.03, 0.36) | -0.9774 | 0.6005 |
| ｐ13290 | 42 | 0.36 (0.23, 0.49) | 39 | 0.47 (0.34, 0.6) | 0.3716 | 0.6003 |
| ｐ18434 | 42 | 0.36 (0.23, 0.49) | 39 | 0.47 (0.34, 0.6) | 0.3716 | 0.6003 |
| ｐ15650 | 42 | 0.58 (0.5, 0.66) | 39 | 0.51 (0.43, 0.59) | -0.1768 | 0.6002 |
| ｐ2719 | 42 | 0.69 (0.59, 0.78) | 39 | 0.61 (0.51, 0.71) | -0.1768 | 0.6001 |
| ｐ21999 | 42 | 0.08 (0, 0.17) | 39 | 0.16 (0.07, 0.24) | 0.8844 | 0.5996 |
| ｐ17737 | 42 | 0.32 (0.27, 0.36) | 39 | 0.28 (0.23, 0.32) | -0.1780 | 0.5991 |
| ｐ9558 | 42 | 0.51 (0.44, 0.59) | 39 | 0.45 (0.38, 0.53) | -0.1806 | 0.5969 |
| ｐ22919 | 42 | 0.82 (0.7, 0.94) | 39 | 0.72 (0.6, 0.85) | -0.1812 | 0.5965 |
| ｐ11209 | 42 | 0.59 (0.51, 0.68) | 39 | 0.52 (0.44, 0.61) | -0.1812 | 0.5965 |
| ｐ16935 | 42 | 0.29 (0.25, 0.33) | 39 | 0.25 (0.21, 0.29) | -0.1817 | 0.5960 |
| ｐ17624 | 42 | 2.69 (1.9, 3.48) | 39 | 2.03 (1.21, 2.85) | -0.4052 | 0.5957 |
| ｐ13975 | 42 | 0.79 (-0.03, 1.62) | 39 | 1.48 (0.62, 2.34) | 0.8983 | 0.5951 |
| ｐ16185 | 42 | 0.94 (0.8, 1.08) | 39 | 0.83 (0.69, 0.97) | -0.1839 | 0.5945 |
| ｐ19111 | 42 | 0.94 (0.8, 1.08) | 39 | 0.83 (0.69, 0.97) | -0.1839 | 0.5945 |
| ｐ19295 | 42 | 0.94 (0.8, 1.08) | 39 | 0.83 (0.69, 0.97) | -0.1839 | 0.5945 |
| ｐ14814 | 42 | 0.46 (0.4, 0.53) | 39 | 0.41 (0.34, 0.48) | -0.1842 | 0.5941 |
| ｐ17545 | 42 | 0.2 (0.1, 0.29) | 39 | 0.27 (0.18, 0.37) | 0.4750 | 0.5938 |
| ｐ18564 | 42 | 0.11 (0.07, 0.15) | 39 | 0.08 (0.04, 0.12) | -0.4825 | 0.5933 |
| ｐ137 | 42 | 0.74 (0.6, 0.87) | 39 | 0.85 (0.71, 0.98) | 0.2003 | 0.5926 |
| ｐ12759 | 42 | 0.64 (0.54, 0.73) | 39 | 0.56 (0.46, 0.66) | -0.1863 | 0.5925 |
| ｐ20720 | 42 | 0.58 (0.49, 0.67) | 39 | 0.66 (0.56, 0.75) | 0.1738 | 0.5922 |
| ｐ1224 | 42 | 0.57 (0.31, 0.83) | 39 | 0.79 (0.51, 1.06) | 0.4675 | 0.5921 |
| ｐ173 | 42 | 0.21 (0.1, 0.32) | 39 | 0.12 (0.01, 0.23) | -0.8161 | 0.5920 |
| ｐ21096 | 42 | 0.14 (-0.1, 0.37) | 39 | 0.33 (0.09, 0.57) | 1.2843 | 0.5918 |
| ｐ14713 | 42 | 0.23 (0.19, 0.26) | 39 | 0.2 (0.16, 0.23) | -0.1874 | 0.5917 |
| ｐ22809 | 42 | 0.74 (0.68, 0.8) | 39 | 0.79 (0.73, 0.85) | 0.0897 | 0.5912 |
| ｐ15534 | 42 | 0.74 (0.69, 0.78) | 39 | 0.77 (0.73, 0.82) | 0.0701 | 0.5909 |
| ｐ22884 | 42 | 0.63 (0.54, 0.73) | 39 | 0.56 (0.46, 0.65) | -0.1888 | 0.5907 |
| ｐ16129 | 42 | 0.28 (0.24, 0.32) | 39 | 0.25 (0.2, 0.29) | -0.1893 | 0.5904 |
| ｐ12922 | 41 | 0.36 (0.17, 0.55) | 39 | 0.52 (0.32, 0.71) | 0.5132 | 0.5899 |
| ｐ7805 | 42 | 0.45 (0.38, 0.52) | 39 | 0.4 (0.33, 0.47) | -0.1905 | 0.5895 |
| ｐ22714 | 42 | 0.52 (-0.19, 1.23) | 39 | 1.11 (0.37, 1.84) | 1.0923 | 0.5894 |
| ｐ19097 | 42 | 0.23 (0.2, 0.27) | 39 | 0.2 (0.17, 0.24) | -0.1912 | 0.5891 |
| ｐ17378 | 42 | 0.67 (0.57, 0.77) | 39 | 0.59 (0.48, 0.69) | -0.1926 | 0.5880 |
| ｐ20429 | 42 | 0.2 (0.17, 0.23) | 39 | 0.18 (0.15, 0.21) | -0.1925 | 0.5880 |
| ｐ14797 | 42 | 0.29 (0.16, 0.43) | 39 | 0.18 (0.04, 0.32) | -0.7034 | 0.5876 |
| ｐ15177 | 42 | 0.02 (0, 0.04) | 39 | 0 (-0.02, 0.02) | NA | 0.5875 |
| ｐ15055 | 42 | 0.48 (0.4, 0.55) | 39 | 0.42 (0.34, 0.49) | -0.1934 | 0.5874 |
| ｐ22019 | 42 | 0.45 (0.33, 0.57) | 39 | 0.55 (0.43, 0.67) | 0.2804 | 0.5873 |
| ｐ21448 | 42 | 0.9 (0.76, 1.03) | 39 | 0.78 (0.64, 0.92) | -0.1942 | 0.5869 |
| ｐ21563 | 42 | 0.23 (0.17, 0.29) | 39 | 0.28 (0.22, 0.34) | 0.2801 | 0.5858 |
| ｐ16470 | 42 | 0.16 (-0.03, 0.35) | 39 | 0 (-0.2, 0.2) | NA | 0.5855 |
| ｐ14870 | 42 | 0.53 (0.45, 0.62) | 39 | 0.47 (0.38, 0.55) | -0.1969 | 0.5850 |
| ｐ19064 | 42 | 0.53 (0.45, 0.62) | 39 | 0.47 (0.38, 0.55) | -0.1969 | 0.5850 |
| ｐ21945 | 42 | 0.18 (0.15, 0.21) | 39 | 0.16 (0.13, 0.19) | -0.1980 | 0.5843 |
| ｐ14838 | 42 | 0.44 (0.37, 0.5) | 39 | 0.38 (0.31, 0.45) | -0.1981 | 0.5842 |
| ｐ12539 | 42 | 0.76 (0.44, 1.08) | 39 | 0.5 (0.17, 0.83) | -0.6100 | 0.5838 |
| ｐ11185 | 42 | 0.05 (0.01, 0.1) | 39 | 0.09 (0.04, 0.13) | 0.7282 | 0.5828 |
| ｐ7203 | 42 | 0.36 (0.18, 0.54) | 39 | 0.21 (0.02, 0.4) | -0.7726 | 0.5816 |
| ｐ17730 | 42 | 0.32 (0.27, 0.38) | 39 | 0.28 (0.23, 0.34) | -0.2029 | 0.5812 |
| ｐ22805 | 42 | 0.38 (-0.46, 1.22) | 39 | 1.07 (0.2, 1.94) | 1.4868 | 0.5811 |
| ｐ14676 | 41 | 1.35 (1.1, 1.59) | 39 | 1.15 (0.9, 1.4) | -0.2298 | 0.5782 |
| ｐ22673 | 42 | 0 (-0.08, 0.08) | 39 | 0.06 (-0.02, 0.14) | NA | 0.5780 |
| ｐ21539 | 42 | 0.18 (0.15, 0.21) | 39 | 0.16 (0.13, 0.19) | -0.2091 | 0.5773 |
| ｐ12747 | 42 | 0.46 (0.38, 0.54) | 39 | 0.4 (0.32, 0.48) | -0.2097 | 0.5770 |
| ｐ17651 | 42 | 0.24 (0.17, 0.32) | 39 | 0.3 (0.23, 0.38) | 0.3203 | 0.5768 |
| ｐ22788 | 42 | 0.58 (-1.11, 2.27) | 39 | 1.95 (0.2, 3.71) | 1.7525 | 0.5764 |
| ｐ17972 | 42 | 0.22 (0.15, 0.29) | 39 | 0.27 (0.2, 0.34) | 0.3293 | 0.5759 |
| ｐ12779 | 42 | 0.42 (0.35, 0.49) | 39 | 0.36 (0.29, 0.43) | -0.2118 | 0.5758 |
| ｐ22363 | 42 | 0.12 (0.07, 0.17) | 39 | 0.08 (0.03, 0.13) | -0.5519 | 0.5755 |
| ｐ15646 | 42 | 0.61 (0.51, 0.72) | 39 | 0.53 (0.42, 0.64) | -0.2139 | 0.5746 |
| ｐ17632 | 42 | 0.99 (0.82, 1.15) | 39 | 0.85 (0.67, 1.02) | -0.2146 | 0.5741 |
| ｐ15685 | 42 | 0.57 (0.47, 0.67) | 39 | 0.49 (0.39, 0.59) | -0.2146 | 0.5741 |
| ｐ14748 | 42 | 0.32 (0.27, 0.38) | 39 | 0.28 (0.22, 0.34) | -0.2158 | 0.5736 |
| ｐ19684 | 41 | 0.74 (0.68, 0.8) | 39 | 0.79 (0.73, 0.85) | 0.0887 | 0.5733 |
| ｐ12095 | 42 | 0.05 (0.04, 0.06) | 39 | 0.04 (0.03, 0.05) | -0.2167 | 0.5731 |
| ｐ15448 | 42 | 0.31 (0.27, 0.36) | 39 | 0.35 (0.3, 0.39) | 0.1595 | 0.5724 |
| ｐ17566 | 42 | 0.49 (0.46, 0.52) | 39 | 0.47 (0.43, 0.5) | -0.0800 | 0.5722 |
| ｐ15371 | 42 | 0.53 (0.44, 0.63) | 39 | 0.45 (0.35, 0.55) | -0.2260 | 0.5717 |
| ｐ14890 | 42 | 0.27 (0.22, 0.31) | 39 | 0.23 (0.18, 0.28) | -0.2193 | 0.5716 |
| ｐ14706 | 42 | 0.52 (0.43, 0.61) | 39 | 0.45 (0.35, 0.54) | -0.2194 | 0.5716 |
| ｐ15064 | 42 | 0.52 (0.43, 0.61) | 39 | 0.45 (0.35, 0.54) | -0.2196 | 0.5715 |
| ｐ22648 | 42 | 0.78 (0.64, 0.91) | 39 | 0.67 (0.53, 0.81) | -0.2206 | 0.5710 |
| ｐ12963 | 42 | 0.2 (0.12, 0.29) | 39 | 0.27 (0.18, 0.35) | 0.4047 | 0.5709 |
| ｐ22672 | 42 | 0 (-0.03, 0.03) | 39 | 0.02 (-0.01, 0.05) | NA | 0.5706 |
| ｐ20985 | 42 | 0.29 (0.22, 0.35) | 39 | 0.23 (0.16, 0.3) | -0.3044 | 0.5702 |
| ｐ11596 | 42 | 0.35 (0.29, 0.41) | 39 | 0.3 (0.23, 0.36) | -0.2226 | 0.5699 |
| ｐ15155 | 42 | 0.55 (0.41, 0.69) | 39 | 0.44 (0.29, 0.58) | -0.3332 | 0.5694 |
| ｐ8 | 42 | 1.8 (1.17, 2.43) | 39 | 2.3 (1.65, 2.96) | 0.3592 | 0.5694 |
| ｐ14622 | 39 | 0.53 (0.27, 0.8) | 39 | 0.32 (0.06, 0.59) | -0.7201 | 0.5693 |
| ｐ21824 | 42 | 0.56 (-1.86, 2.98) | 39 | 2.51 (-0.01, 5.02) | 2.1666 | 0.5693 |
| ｐ379 | 41 | 1.6 (0.15, 3.05) | 39 | 0.44 (-1.05, 1.93) | -1.8657 | 0.5690 |
| ｐ14761 | 42 | 0.91 (0.75, 1.08) | 39 | 0.78 (0.61, 0.95) | -0.2245 | 0.5689 |
| ｐ16229 | 42 | 0.1 (-0.06, 0.25) | 39 | 0.22 (0.06, 0.39) | 1.2079 | 0.5685 |
| ｐ22785 | 42 | 0.19 (-0.55, 0.92) | 39 | 0.78 (0.02, 1.54) | 2.0496 | 0.5681 |
| ｐ15219 | 42 | 0.41 (0.2, 0.62) | 39 | 0.24 (0.02, 0.46) | -0.7738 | 0.5672 |
| ｐ20854 | 42 | 0.8 (0.72, 0.88) | 39 | 0.87 (0.78, 0.95) | 0.1165 | 0.5650 |
| ｐ18408 | 42 | 0.44 (0.43, 0.45) | 39 | 0.43 (0.42, 0.44) | -0.0248 | 0.5648 |
| ｐ318 | 42 | 0.5 (0.31, 0.69) | 39 | 0.34 (0.14, 0.54) | -0.5352 | 0.5646 |
| ｐ5630 | 42 | 0.82 (0.49, 1.14) | 39 | 0.55 (0.21, 0.89) | -0.5591 | 0.5645 |
| ｐ18945 | 42 | 0.53 (0.32, 0.74) | 39 | 0.7 (0.48, 0.92) | 0.3986 | 0.5643 |
| ｐ17768 | 42 | 0.66 (0.65, 0.67) | 39 | 0.65 (0.64, 0.66) | -0.0187 | 0.5643 |
| ｐ22371 | 42 | 0.71 (0.71, 0.72) | 39 | 0.71 (0.7, 0.71) | -0.0109 | 0.5643 |
| ｐ11607 | 42 | 0.86 (0.49, 1.23) | 39 | 0.56 (0.17, 0.95) | -0.6174 | 0.5642 |
| ｐ22052 | 42 | 0.62 (0.48, 0.76) | 39 | 0.73 (0.59, 0.88) | 0.2413 | 0.5639 |
| ｐ363 | 42 | 1.12 (0.58, 1.66) | 39 | 0.69 (0.13, 1.25) | -0.6956 | 0.5629 |
| ｐ22960 | 42 | 0.19 (0.09, 0.3) | 39 | 0.28 (0.17, 0.39) | 0.5243 | 0.5623 |
| ｐ14702 | 42 | 0.16 (0.13, 0.19) | 39 | 0.14 (0.1, 0.17) | -0.2402 | 0.5615 |
| ｐ14839 | 42 | 0.61 (0.5, 0.73) | 39 | 0.52 (0.4, 0.64) | -0.2404 | 0.5614 |
| ｐ14364 | 42 | 0.72 (0.58, 0.86) | 39 | 0.61 (0.46, 0.75) | -0.2429 | 0.5603 |
| ｐ15047 | 42 | 1.53 (1, 2.06) | 39 | 1.11 (0.56, 1.66) | -0.4618 | 0.5599 |
| ｐ15593 | 42 | 0.29 (0.24, 0.35) | 39 | 0.25 (0.19, 0.31) | -0.2442 | 0.5597 |
| ｐ19589 | 42 | 0.04 (0.03, 0.04) | 39 | 0.03 (0.02, 0.04) | -0.2462 | 0.5589 |
| ｐ22045 | 42 | 0.36 (-0.02, 0.74) | 39 | 0.67 (0.27, 1.06) | 0.8815 | 0.5589 |
| ｐ10748 | 42 | 0.5 (0.4, 0.61) | 39 | 0.42 (0.32, 0.53) | -0.2486 | 0.5579 |
| ｐ14868 | 42 | 0.24 (0.07, 0.4) | 39 | 0.37 (0.2, 0.54) | 0.6408 | 0.5575 |
| ｐ17609 | 42 | 0.51 (0.41, 0.61) | 39 | 0.43 (0.32, 0.53) | -0.2499 | 0.5574 |
| ｐ19930 | 42 | 0.32 (0.23, 0.41) | 39 | 0.25 (0.16, 0.34) | -0.3576 | 0.5574 |
| ｐ11057 | 42 | 0.29 (0.06, 0.51) | 39 | 0.11 (-0.12, 0.34) | -1.3901 | 0.5568 |
| ｐ22023 | 42 | 0.32 (0.21, 0.43) | 39 | 0.4 (0.29, 0.52) | 0.3482 | 0.5554 |
| ｐ17031 | 42 | 0.36 (0.28, 0.43) | 39 | 0.3 (0.22, 0.37) | -0.2584 | 0.5541 |
| ｐ22647 | 42 | 0.29 (0.23, 0.35) | 39 | 0.24 (0.18, 0.3) | -0.2601 | 0.5534 |
| ｐ16862 | 42 | 0.56 (0.54, 0.58) | 39 | 0.58 (0.55, 0.6) | 0.0445 | 0.5533 |
| ｐ4739 | 41 | 0.21 (0.13, 0.29) | 39 | 0.27 (0.19, 0.36) | 0.3826 | 0.5533 |
| ｐ22944 | 42 | 0.66 (0.49, 0.84) | 39 | 0.8 (0.62, 0.98) | 0.2722 | 0.5530 |
| ｐ16646 | 42 | 0.52 (0.41, 0.63) | 39 | 0.43 (0.32, 0.55) | -0.2620 | 0.5527 |
| ｐ13152 | 42 | 0.21 (0.17, 0.26) | 39 | 0.18 (0.13, 0.22) | -0.2623 | 0.5526 |
| ｐ17956 | 42 | 0.54 (0.43, 0.66) | 39 | 0.45 (0.33, 0.57) | -0.2639 | 0.5521 |
| ｐ11818 | 42 | 0.3 (-0.63, 1.22) | 39 | 1.03 (0.07, 1.99) | 1.7783 | 0.5520 |
| ｐ18371 | 42 | 0.23 (0.18, 0.28) | 39 | 0.19 (0.14, 0.24) | -0.2664 | 0.5511 |
| ｐ15966 | 42 | 0.49 (0.41, 0.58) | 39 | 0.56 (0.47, 0.65) | 0.1868 | 0.5504 |
| ｐ22428 | 42 | 0.26 (0.16, 0.36) | 39 | 0.34 (0.24, 0.45) | 0.3867 | 0.5503 |
| ｐ11156 | 42 | 0.53 (0.03, 1.02) | 39 | 0.14 (-0.37, 0.65) | -1.9106 | 0.5490 |
| ｐ22029 | 42 | 1.98 (1.93, 2.02) | 39 | 2.01 (1.97, 2.06) | 0.0248 | 0.5489 |
| ｐ15524 | 42 | 0.33 (0.28, 0.38) | 39 | 0.37 (0.32, 0.42) | 0.1574 | 0.5488 |
| ｐ14865 | 42 | 0.78 (0.61, 0.96) | 39 | 0.65 (0.47, 0.83) | -0.2746 | 0.5483 |
| ｐ22634 | 42 | 0.97 (0.75, 1.18) | 39 | 0.8 (0.58, 1.02) | -0.2754 | 0.5480 |
| ｐ21053 | 42 | 0.34 (0.26, 0.42) | 39 | 0.28 (0.2, 0.36) | -0.2777 | 0.5473 |
| ｐ19080 | 42 | 0.22 (0.13, 0.31) | 39 | 0.15 (0.06, 0.24) | -0.5347 | 0.5465 |
| ｐ14179 | 42 | 0.42 (0.31, 0.52) | 39 | 0.5 (0.39, 0.61) | 0.2667 | 0.5464 |
| ｐ22180 | 42 | 0.39 (0.31, 0.48) | 39 | 0.32 (0.23, 0.42) | -0.2813 | 0.5461 |
| ｐ15555 | 42 | 0.57 (0.4, 0.74) | 39 | 0.44 (0.26, 0.62) | -0.3826 | 0.5453 |
| ｐ15435 | 42 | 0.32 (0.24, 0.39) | 39 | 0.26 (0.18, 0.34) | -0.2848 | 0.5450 |
| ｐ7732 | 42 | 0.92 (0.72, 1.12) | 39 | 1.08 (0.87, 1.29) | 0.2293 | 0.5437 |
| ｐ16382 | 42 | 0.1 (0.07, 0.12) | 39 | 0.08 (0.06, 0.1) | -0.2938 | 0.5424 |
| ｐ19556 | 42 | 0.12 (-0.06, 0.3) | 39 | 0.26 (0.07, 0.45) | 1.1238 | 0.5421 |
| ｐ15347 | 42 | 0.72 (0.24, 1.19) | 39 | 0.35 (-0.14, 0.84) | -1.0382 | 0.5414 |
| ｐ18444 | 42 | 0.53 (-0.57, 1.63) | 39 | 1.39 (0.24, 2.53) | 1.3840 | 0.5412 |
| ｐ10863 | 42 | 0.42 (0.29, 0.56) | 39 | 0.53 (0.39, 0.66) | 0.3145 | 0.5412 |
| ｐ15373 | 42 | 0.31 (0.27, 0.36) | 39 | 0.28 (0.23, 0.32) | -0.1656 | 0.5408 |
| ｐ17754 | 42 | 0.7 (0.53, 0.87) | 39 | 0.56 (0.39, 0.74) | -0.3039 | 0.5396 |
| ｐ17060 | 42 | 0.45 (0.37, 0.54) | 39 | 0.39 (0.3, 0.48) | -0.2203 | 0.5381 |
| ｐ14322 | 42 | 0.52 (0.39, 0.65) | 39 | 0.42 (0.28, 0.55) | -0.3105 | 0.5378 |
| ｐ13035 | 42 | 0.72 (0.41, 1.04) | 39 | 0.48 (0.15, 0.81) | -0.5986 | 0.5375 |
| ｐ13936 | 42 | 1.27 (0.95, 1.59) | 39 | 1.02 (0.69, 1.35) | -0.3119 | 0.5375 |
| ｐ17010 | 42 | 0.2 (0.15, 0.26) | 39 | 0.17 (0.11, 0.22) | -0.3119 | 0.5375 |
| ｐ22867 | 42 | 0.48 (0.37, 0.6) | 39 | 0.4 (0.28, 0.51) | -0.2844 | 0.5374 |
| ｐ16582 | 42 | 0.85 (0.63, 1.07) | 39 | 0.68 (0.46, 0.91) | -0.3146 | 0.5369 |
| ｐ13582 | 42 | 0.21 (0.15, 0.26) | 39 | 0.17 (0.11, 0.22) | -0.3156 | 0.5365 |
| ｐ20159 | 42 | 0.3 (0.11, 0.49) | 39 | 0.45 (0.25, 0.64) | 0.5650 | 0.5360 |
| ｐ4056 | 42 | 0.86 (0.74, 0.98) | 39 | 0.77 (0.65, 0.89) | -0.1578 | 0.5359 |
| ｐ22017 | 42 | 0.39 (0.34, 0.44) | 39 | 0.43 (0.38, 0.48) | 0.1284 | 0.5352 |
| ｐ16123 | 42 | 0.2 (0.1, 0.29) | 39 | 0.13 (0.03, 0.22) | -0.6554 | 0.5351 |
| ｐ8268 | 42 | 0.06 (0.04, 0.07) | 39 | 0.05 (0.03, 0.06) | -0.3222 | 0.5350 |
| ｐ22875 | 42 | 1.11 (0.33, 1.88) | 39 | 0.51 (-0.29, 1.32) | -1.1135 | 0.5347 |
| ｐ22032 | 42 | 0.12 (0.07, 0.17) | 39 | 0.16 (0.1, 0.21) | 0.4149 | 0.5341 |
| ｐ10700 | 42 | 0.4 (-0.04, 0.84) | 39 | 0.74 (0.28, 1.2) | 0.8828 | 0.5340 |
| ｐ21332 | 42 | 0.59 (0.43, 0.74) | 39 | 0.47 (0.31, 0.63) | -0.3271 | 0.5338 |
| ｐ18203 | 42 | 0.66 (0.49, 0.84) | 39 | 0.53 (0.35, 0.71) | -0.3279 | 0.5336 |
| ｐ1768 | 42 | 0.84 (0.01, 1.67) | 39 | 1.48 (0.62, 2.34) | 0.8102 | 0.5329 |
| ｐ6401 | 42 | 0.63 (0.46, 0.81) | 39 | 0.5 (0.33, 0.68) | -0.3328 | 0.5325 |
| ｐ1340 | 42 | 0.14 (0.02, 0.27) | 39 | 0.05 (-0.08, 0.18) | -1.5905 | 0.5306 |
| ｐ14720 | 42 | 0.58 (0.53, 0.64) | 39 | 0.54 (0.48, 0.6) | -0.1079 | 0.5293 |
| ｐ6391 | 42 | 0.24 (0.14, 0.34) | 39 | 0.16 (0.06, 0.27) | -0.5494 | 0.5293 |
| ｐ15439 | 42 | 0.2 (0.07, 0.33) | 39 | 0.3 (0.16, 0.43) | 0.5798 | 0.5271 |
| ｐ14385 | 42 | 0.59 (0.33, 0.84) | 39 | 0.39 (0.13, 0.66) | -0.5725 | 0.5268 |
| ｐ18518 | 42 | 0.13 (0, 0.25) | 39 | 0.22 (0.09, 0.35) | 0.8156 | 0.5265 |
| ｐ12395 | 42 | 0.41 (0.06, 0.77) | 39 | 0.68 (0.32, 1.05) | 0.7184 | 0.5258 |
| ｐ19169 | 42 | 0.41 (0.06, 0.77) | 39 | 0.68 (0.32, 1.05) | 0.7184 | 0.5258 |
| ｐ11462 | 42 | 0.44 (0.31, 0.57) | 39 | 0.34 (0.21, 0.48) | -0.3681 | 0.5255 |
| ｐ17942 | 42 | 0.2 (0.14, 0.26) | 39 | 0.16 (0.09, 0.22) | -0.3789 | 0.5236 |
| ｐ13224 | 42 | 0.37 (0.21, 0.52) | 39 | 0.25 (0.09, 0.41) | -0.5469 | 0.5219 |
| ｐ22928 | 42 | 0.55 (0.38, 0.72) | 39 | 0.42 (0.24, 0.6) | -0.3901 | 0.5218 |
| ｐ16285 | 42 | 0.16 (0.11, 0.21) | 39 | 0.12 (0.07, 0.17) | -0.3942 | 0.5211 |
| ｐ11313 | 42 | 1.62 (0.68, 2.57) | 39 | 0.91 (-0.07, 1.89) | -0.8305 | 0.5202 |
| ｐ21449 | 42 | 0.74 (0.5, 0.98) | 39 | 0.56 (0.31, 0.81) | -0.4039 | 0.5197 |
| ｐ15112 | 42 | 0.74 (0.5, 0.98) | 39 | 0.56 (0.31, 0.81) | -0.4039 | 0.5197 |
| ｐ18859 | 42 | 0 (-0.02, 0.02) | 39 | 0.02 (-0.01, 0.04) | NA | 0.5196 |
| ｐ22103 | 42 | 0 (-0.43, 0.43) | 39 | 0.33 (-0.12, 0.78) | NA | 0.5195 |
| ｐ819 | 42 | 0.59 (0.43, 0.74) | 39 | 0.47 (0.31, 0.63) | -0.3244 | 0.5194 |
| ｐ17472 | 42 | 0.71 (0.48, 0.95) | 39 | 0.54 (0.29, 0.78) | -0.4094 | 0.5190 |
| ｐ13195 | 42 | 0.36 (0.24, 0.48) | 39 | 0.27 (0.15, 0.39) | -0.4101 | 0.5188 |
| ｐ17697 | 42 | 0.35 (0.24, 0.47) | 39 | 0.27 (0.14, 0.39) | -0.4122 | 0.5186 |
| ｐ8600 | 42 | 0.35 (0.24, 0.47) | 39 | 0.27 (0.14, 0.39) | -0.4122 | 0.5185 |
| ｐ15828 | 42 | 0.29 (0.19, 0.38) | 39 | 0.21 (0.12, 0.31) | -0.4156 | 0.5181 |
| ｐ18117 | 42 | 0.66 (0.44, 0.88) | 39 | 0.5 (0.27, 0.73) | -0.4159 | 0.5180 |
| ｐ399 | 42 | 3.87 (2.35, 5.39) | 39 | 2.73 (1.16, 4.31) | -0.5024 | 0.5179 |
| ｐ22902 | 42 | 0.55 (0.37, 0.74) | 39 | 0.41 (0.22, 0.61) | -0.4206 | 0.5174 |
| ｐ15926 | 42 | 0.31 (0.2, 0.41) | 39 | 0.23 (0.12, 0.34) | -0.4235 | 0.5170 |
| ｐ22930 | 42 | 0.78 (0.51, 1.05) | 39 | 0.58 (0.3, 0.86) | -0.4292 | 0.5162 |
| ｐ10916 | 42 | 0.24 (0.17, 0.3) | 39 | 0.19 (0.12, 0.26) | -0.3254 | 0.5162 |
| ｐ19751 | 42 | 0.17 (0.11, 0.22) | 39 | 0.12 (0.06, 0.18) | -0.4330 | 0.5158 |
| ｐ12701 | 42 | 0.65 (-0.31, 1.62) | 39 | 1.37 (0.37, 2.37) | 1.0757 | 0.5147 |
| ｐ22985 | 42 | 0.34 (0.3, 0.38) | 39 | 0.37 (0.33, 0.41) | 0.1202 | 0.5145 |
| ｐ22901 | 42 | 0.45 (0.29, 0.61) | 39 | 0.33 (0.16, 0.49) | -0.4469 | 0.5141 |
| ｐ15386 | 42 | 0.55 (0.35, 0.75) | 39 | 0.7 (0.49, 0.91) | 0.3505 | 0.5133 |
| ｐ7280 | 42 | 0.8 (0.76, 0.84) | 39 | 0.77 (0.73, 0.82) | -0.0546 | 0.5114 |
| ｐ17741 | 42 | 0.73 (-0.11, 1.57) | 39 | 1.35 (0.48, 2.22) | 0.8870 | 0.5112 |
| ｐ17654 | 42 | 0.19 (0.06, 0.31) | 39 | 0.28 (0.15, 0.41) | 0.5894 | 0.5106 |
| ｐ22089 | 42 | 0.64 (0.62, 0.66) | 39 | 0.66 (0.64, 0.68) | 0.0360 | 0.5102 |
| ｐ12944 | 42 | 0.17 (0.11, 0.24) | 39 | 0.12 (0.05, 0.19) | -0.4872 | 0.5097 |
| ｐ22934 | 42 | 0.2 (-0.27, 0.67) | 39 | 0.55 (0.06, 1.04) | 1.4486 | 0.5094 |
| ｐ16182 | 42 | 0.2 (0.11, 0.29) | 39 | 0.26 (0.17, 0.36) | 0.4148 | 0.5079 |
| ｐ13614 | 42 | 0.85 (0.33, 1.36) | 39 | 1.23 (0.69, 1.76) | 0.5361 | 0.5077 |
| ｐ12736 | 42 | 0.58 (0.34, 0.81) | 39 | 0.4 (0.16, 0.65) | -0.5095 | 0.5076 |
| ｐ2187 | 42 | 0.1 (0.06, 0.15) | 39 | 0.14 (0.09, 0.18) | 0.3943 | 0.5071 |
| ｐ15012 | 42 | 0.22 (0.2, 0.24) | 39 | 0.21 (0.19, 0.23) | -0.0873 | 0.5064 |
| ｐ16428 | 42 | 0.76 (0.19, 1.32) | 39 | 0.34 (-0.25, 0.93) | -1.1652 | 0.5061 |
| ｐ5300 | 42 | 0.26 (0.11, 0.4) | 39 | 0.15 (-0.01, 0.3) | -0.7937 | 0.5053 |
| ｐ6001 | 42 | 0.22 (0.12, 0.31) | 39 | 0.15 (0.05, 0.24) | -0.5389 | 0.5051 |
| ｐ202 | 42 | 0.66 (0.47, 0.85) | 39 | 0.52 (0.32, 0.71) | -0.3481 | 0.5049 |
| ｐ22102 | 42 | 0.49 (0.33, 0.65) | 39 | 0.61 (0.44, 0.77) | 0.3134 | 0.5039 |
| ｐ21447 | 42 | 0.48 (-0.13, 1.09) | 39 | 0.93 (0.29, 1.56) | 0.9539 | 0.5027 |
| ｐ413 | 42 | 1.4 (0.1, 2.69) | 39 | 2.34 (1, 3.69) | 0.7485 | 0.5027 |
| ｐ14133 | 42 | 0.28 (-0.08, 0.64) | 39 | 0.55 (0.17, 0.92) | 0.9476 | 0.5026 |
| ｐ17217 | 42 | 0.52 (0.26, 0.78) | 39 | 0.71 (0.44, 0.97) | 0.4487 | 0.5025 |
| ｐ14695 | 42 | 0.72 (0.39, 1.04) | 39 | 0.48 (0.14, 0.81) | -0.5812 | 0.5020 |
| ｐ17859 | 42 | 0.39 (-0.05, 0.82) | 39 | 0.7 (0.25, 1.15) | 0.8645 | 0.5018 |
| ｐ114 | 42 | 0.26 (0.09, 0.43) | 39 | 0.39 (0.21, 0.56) | 0.5626 | 0.5006 |
| ｐ21756 | 42 | 0.15 (0.05, 0.24) | 39 | 0.21 (0.12, 0.31) | 0.5600 | 0.4999 |
| ｐ13847 | 42 | 0.22 (0.18, 0.26) | 39 | 0.25 (0.21, 0.29) | 0.1715 | 0.4995 |
| ｐ22094 | 42 | 0.01 (-0.06, 0.09) | 39 | 0.07 (-0.01, 0.15) | 2.2355 | 0.4985 |
| ｐ17801 | 42 | 0.76 (0.5, 1.02) | 39 | 0.57 (0.3, 0.84) | -0.4132 | 0.4980 |
| ｐ22026 | 42 | 0.47 (0.28, 0.65) | 39 | 0.6 (0.41, 0.8) | 0.3687 | 0.4975 |
| ｐ20583 | 42 | 0.19 (0.01, 0.37) | 39 | 0.32 (0.13, 0.5) | 0.7707 | 0.4973 |
| ｐ14809 | 42 | 0.24 (0.18, 0.3) | 39 | 0.2 (0.14, 0.26) | -0.2890 | 0.4972 |
| ｐ11495 | 42 | 1.16 (0.57, 1.75) | 39 | 0.73 (0.12, 1.34) | -0.6666 | 0.4970 |
| ｐ14631 | 42 | 0.94 (0.45, 1.43) | 39 | 0.59 (0.08, 1.09) | -0.6838 | 0.4961 |
| ｐ16597 | 42 | 0.14 (0.1, 0.17) | 39 | 0.11 (0.08, 0.15) | -0.2900 | 0.4961 |
| ｐ4940 | 42 | 0.17 (0.06, 0.27) | 39 | 0.24 (0.13, 0.35) | 0.5372 | 0.4960 |
| ｐ17197 | 42 | 0.36 (0.17, 0.55) | 39 | 0.22 (0.02, 0.42) | -0.7100 | 0.4949 |
| ｐ19719 | 42 | 0.66 (0.53, 0.78) | 39 | 0.75 (0.62, 0.87) | 0.1847 | 0.4943 |
| ｐ22070 | 42 | 0.13 (0.09, 0.18) | 39 | 0.17 (0.12, 0.21) | 0.2938 | 0.4942 |
| ｐ14718 | 42 | 0.63 (0.21, 1.05) | 39 | 0.33 (-0.1, 0.77) | -0.9387 | 0.4941 |
| ｐ6110 | 42 | 0.26 (0.15, 0.37) | 39 | 0.18 (0.07, 0.29) | -0.5205 | 0.4936 |
| ｐ22947 | 42 | 0.33 (0.18, 0.48) | 39 | 0.44 (0.28, 0.6) | 0.4196 | 0.4930 |
| ｐ20956 | 42 | 0.21 (0.09, 0.33) | 39 | 0.12 (0, 0.25) | -0.7680 | 0.4925 |
| ｐ15043 | 42 | 0.4 (0.32, 0.48) | 39 | 0.46 (0.37, 0.54) | 0.1988 | 0.4920 |
| ｐ16246 | 42 | 0.4 (0.32, 0.48) | 39 | 0.46 (0.37, 0.54) | 0.1988 | 0.4920 |
| ｐ347 | 42 | 1.01 (0.64, 1.37) | 39 | 0.75 (0.37, 1.12) | -0.4329 | 0.4917 |
| ｐ21636 | 42 | 0.37 (0.21, 0.54) | 39 | 0.49 (0.32, 0.66) | 0.3953 | 0.4911 |
| ｐ14682 | 39 | 0.17 (-0.06, 0.39) | 39 | 0.32 (0.1, 0.54) | 0.9575 | 0.4910 |
| ｐ18721 | 42 | 0.25 (0.2, 0.31) | 39 | 0.29 (0.24, 0.35) | 0.2019 | 0.4900 |
| ｐ14687 | 42 | 0.57 (0.22, 0.93) | 39 | 0.32 (-0.05, 0.68) | -0.8565 | 0.4896 |
| ｐ17833 | 42 | 0.16 (0.06, 0.26) | 39 | 0.23 (0.13, 0.33) | 0.5344 | 0.4888 |
| ｐ20063 | 42 | 0.22 (0.08, 0.36) | 39 | 0.12 (-0.03, 0.26) | -0.8909 | 0.4886 |
| ｐ8652 | 42 | 0.31 (0.26, 0.36) | 39 | 0.35 (0.3, 0.4) | 0.1562 | 0.4865 |
| ｐ14982 | 42 | 0.45 (-0.05, 0.94) | 39 | 0.8 (0.29, 1.31) | 0.8389 | 0.4856 |
| ｐ16983 | 42 | 0.2 (0.16, 0.23) | 39 | 0.17 (0.14, 0.21) | -0.1939 | 0.4851 |
| ｐ19750 | 42 | 0.2 (0.04, 0.35) | 39 | 0.09 (-0.07, 0.25) | -1.1603 | 0.4829 |
| ｐ13949 | 42 | 0.24 (0.11, 0.37) | 39 | 0.33 (0.19, 0.47) | 0.4785 | 0.4804 |
| ｐ9318 | 42 | 0.57 (0.53, 0.61) | 39 | 0.6 (0.56, 0.64) | 0.0720 | 0.4802 |
| ｐ15487 | 42 | 0.28 (0.2, 0.36) | 39 | 0.33 (0.25, 0.42) | 0.2601 | 0.4795 |
| ｐ22081 | 42 | 0.1 (0.04, 0.16) | 39 | 0.14 (0.08, 0.2) | 0.5227 | 0.4792 |
| ｐ22037 | 42 | 0.05 (0.01, 0.09) | 39 | 0.07 (0.03, 0.11) | 0.6791 | 0.4792 |
| ｐ15683 | 42 | 0.49 (0.34, 0.63) | 39 | 0.39 (0.24, 0.53) | -0.3279 | 0.4739 |
| ｐ2417 | 42 | 0.43 (0.28, 0.58) | 39 | 0.54 (0.38, 0.69) | 0.3138 | 0.4736 |
| ｐ6914 | 42 | 0.32 (0.19, 0.45) | 39 | 0.41 (0.28, 0.54) | 0.3613 | 0.4736 |
| ｐ15167 | 42 | 0.19 (-0.08, 0.46) | 39 | 0 (-0.28, 0.28) | NA | 0.4728 |
| ｐ23083 | 42 | 0.37 (0.2, 0.53) | 39 | 0.48 (0.31, 0.66) | 0.3953 | 0.4713 |
| ｐ16341 | 42 | 0.24 (0.15, 0.34) | 39 | 0.31 (0.21, 0.41) | 0.3498 | 0.4712 |
| ｐ427 | 42 | 0.46 (0.32, 0.6) | 39 | 0.37 (0.22, 0.51) | -0.3403 | 0.4712 |
| ｐ14677 | 42 | 0.04 (-0.02, 0.09) | 39 | 0 (-0.05, 0.05) | NA | 0.4706 |
| ｐ14719 | 42 | 0.03 (-0.01, 0.07) | 39 | 0 (-0.04, 0.04) | NA | 0.4706 |
| ｐ14756 | 42 | 0.05 (-0.02, 0.11) | 39 | 0 (-0.07, 0.07) | NA | 0.4706 |
| ｐ14820 | 42 | 0.04 (-0.02, 0.11) | 39 | 0 (-0.07, 0.07) | NA | 0.4706 |
| ｐ14801 | 42 | 0.03 (-0.01, 0.08) | 39 | 0 (-0.05, 0.05) | NA | 0.4706 |
| ｐ14834 | 42 | 0.11 (-0.05, 0.26) | 39 | 0 (-0.16, 0.16) | NA | 0.4706 |
| ｐ14853 | 42 | 0.02 (-0.01, 0.05) | 39 | 0 (-0.03, 0.03) | NA | 0.4706 |
| ｐ14901 | 42 | 0.05 (-0.02, 0.13) | 39 | 0 (-0.08, 0.08) | NA | 0.4706 |
| ｐ14821 | 42 | 0.01 (0, 0.03) | 39 | 0 (-0.02, 0.02) | NA | 0.4706 |
| ｐ14973 | 42 | 0.03 (-0.01, 0.08) | 39 | 0 (-0.05, 0.05) | NA | 0.4706 |
| ｐ14739 | 42 | 0 (0, 0.01) | 39 | 0 (-0.01, 0.01) | NA | 0.4706 |
| ｐ14819 | 42 | 0.02 (-0.01, 0.04) | 39 | 0 (-0.02, 0.02) | NA | 0.4706 |
| ｐ14891 | 42 | 0.05 (-0.02, 0.12) | 39 | 0 (-0.07, 0.07) | NA | 0.4706 |
| ｐ14697 | 42 | 0.02 (-0.01, 0.05) | 39 | 0 (-0.03, 0.03) | NA | 0.4706 |
| ｐ14849 | 42 | 0.01 (0, 0.02) | 39 | 0 (-0.01, 0.01) | NA | 0.4706 |
| ｐ14685 | 42 | 0.02 (-0.01, 0.05) | 39 | 0 (-0.03, 0.03) | NA | 0.4706 |
| ｐ15161 | 42 | 0.03 (-0.01, 0.07) | 39 | 0 (-0.04, 0.04) | NA | 0.4706 |
| ｐ15171 | 42 | 0.01 (0, 0.02) | 39 | 0 (-0.01, 0.01) | NA | 0.4706 |
| ｐ15263 | 42 | 0.07 (-0.03, 0.18) | 39 | 0 (-0.11, 0.11) | NA | 0.4706 |
| ｐ15326 | 42 | 0.01 (0, 0.03) | 39 | 0 (-0.02, 0.02) | NA | 0.4706 |
| ｐ16422 | 42 | 0.03 (-0.01, 0.06) | 39 | 0 (-0.04, 0.04) | NA | 0.4706 |
| ｐ16656 | 42 | 0.03 (-0.01, 0.06) | 39 | 0 (-0.04, 0.04) | NA | 0.4706 |
| ｐ16703 | 42 | 0.02 (-0.01, 0.05) | 39 | 0 (-0.03, 0.03) | NA | 0.4706 |
| ｐ17291 | 42 | 0.02 (-0.01, 0.06) | 39 | 0 (-0.03, 0.03) | NA | 0.4706 |
| ｐ18495 | 42 | 0.3 (0.21, 0.38) | 39 | 0.36 (0.27, 0.44) | 0.2589 | 0.4685 |
| ｐ17037 | 42 | 0.23 (0.14, 0.32) | 39 | 0.17 (0.08, 0.26) | -0.4537 | 0.4675 |
| ｐ22056 | 42 | 0.47 (0.36, 0.58) | 39 | 0.55 (0.43, 0.66) | 0.2163 | 0.4659 |
| ｐ15764 | 42 | 0.25 (0.22, 0.28) | 39 | 0.23 (0.2, 0.26) | -0.1199 | 0.4638 |
| ｐ22076 | 42 | 0.4 (0.36, 0.43) | 39 | 0.42 (0.39, 0.45) | 0.0759 | 0.4636 |
| ｐ23085 | 42 | 0.23 (0.12, 0.33) | 39 | 0.3 (0.19, 0.41) | 0.3994 | 0.4631 |
| ｐ8584 | 42 | 0.43 (0.38, 0.47) | 39 | 0.46 (0.41, 0.5) | 0.0981 | 0.4622 |
| ｐ15997 | 42 | 0.16 (0.09, 0.23) | 39 | 0.21 (0.14, 0.28) | 0.3817 | 0.4609 |
| ｐ14090 | 42 | 0.32 (0.23, 0.41) | 39 | 0.38 (0.29, 0.48) | 0.2615 | 0.4605 |
| ｐ10645 | 42 | 0.8 (0.56, 1.03) | 39 | 0.64 (0.39, 0.88) | -0.3256 | 0.4600 |
| ｐ16387 | 42 | 0.29 (-0.91, 1.49) | 39 | 1.11 (-0.13, 2.35) | 1.9394 | 0.4599 |
| ｐ14773 | 42 | 0.32 (0.26, 0.38) | 39 | 0.28 (0.21, 0.34) | -0.2084 | 0.4576 |
| ｐ12728 | 42 | 0.34 (0.11, 0.56) | 39 | 0.49 (0.25, 0.73) | 0.5501 | 0.4575 |
| ｐ15216 | 42 | 0.59 (0.37, 0.82) | 39 | 0.74 (0.51, 0.98) | 0.3301 | 0.4569 |
| ｐ16360 | 42 | 0.66 (0.24, 1.09) | 39 | 0.96 (0.51, 1.4) | 0.5245 | 0.4563 |
| ｐ17229 | 42 | 0.38 (0.25, 0.51) | 39 | 0.47 (0.34, 0.6) | 0.2949 | 0.4540 |
| ｐ15913 | 42 | 1.09 (0.35, 1.82) | 39 | 0.59 (-0.17, 1.35) | -0.8826 | 0.4527 |
| ｐ17612 | 42 | 0.54 (0.24, 0.85) | 39 | 0.34 (0.02, 0.65) | -0.6885 | 0.4520 |
| ｐ14778 | 42 | 0.68 (0.56, 0.79) | 39 | 0.75 (0.64, 0.87) | 0.1549 | 0.4519 |
| ｐ19034 | 42 | 0.36 (0.21, 0.51) | 39 | 0.46 (0.3, 0.62) | 0.3628 | 0.4503 |
| ｐ18999 | 42 | 0.19 (0.13, 0.26) | 39 | 0.24 (0.17, 0.3) | 0.2792 | 0.4501 |
| ｐ5140 | 42 | 0.45 (0.32, 0.59) | 39 | 0.54 (0.4, 0.69) | 0.2665 | 0.4469 |
| ｐ18559 | 42 | 0.07 (0, 0.15) | 39 | 0.12 (0.05, 0.2) | 0.7293 | 0.4458 |
| ｐ22939 | 42 | 0.34 (0.07, 0.61) | 39 | 0.52 (0.24, 0.8) | 0.6127 | 0.4457 |
| ｐ22007 | 42 | 1.09 (1.02, 1.16) | 39 | 1.14 (1.06, 1.22) | 0.0637 | 0.4452 |
| ｐ22549 | 42 | 0.48 (0.17, 0.78) | 39 | 0.68 (0.37, 1) | 0.5107 | 0.4437 |
| ｐ14633 | 42 | 0.17 (0.13, 0.2) | 39 | 0.15 (0.11, 0.18) | -0.2081 | 0.4437 |
| ｐ12842 | 42 | 0.39 (0.24, 0.53) | 39 | 0.48 (0.33, 0.64) | 0.3243 | 0.4434 |
| ｐ15940 | 42 | 0.14 (0.03, 0.25) | 39 | 0.21 (0.1, 0.33) | 0.6154 | 0.4432 |
| ｐ21621 | 42 | 0.14 (0.03, 0.25) | 39 | 0.21 (0.1, 0.33) | 0.6154 | 0.4432 |
| ｐ6398 | 42 | 0.83 (0.46, 1.19) | 39 | 1.07 (0.69, 1.45) | 0.3729 | 0.4428 |
| ｐ17182 | 42 | 0.54 (0.51, 0.57) | 39 | 0.52 (0.49, 0.55) | -0.0590 | 0.4409 |
| ｐ4743 | 42 | 0.15 (0.1, 0.21) | 39 | 0.12 (0.06, 0.17) | -0.3982 | 0.4405 |
| ｐ14897 | 42 | 0.15 (-0.05, 0.34) | 39 | 0.02 (-0.19, 0.22) | -3.1268 | 0.4405 |
| ｐ22074 | 42 | 0.91 (0.85, 0.97) | 39 | 0.95 (0.89, 1.02) | 0.0657 | 0.4388 |
| ｐ143 | 42 | 0.38 (0.12, 0.63) | 39 | 0.55 (0.28, 0.81) | 0.5331 | 0.4382 |
| ｐ15514 | 42 | 0.71 (0.36, 1.06) | 39 | 0.48 (0.12, 0.84) | -0.5637 | 0.4379 |
| ｐ13726 | 42 | 0.15 (-0.02, 0.32) | 39 | 0.26 (0.09, 0.44) | 0.7992 | 0.4358 |
| ｐ14920 | 42 | 0.47 (0.24, 0.69) | 39 | 0.32 (0.08, 0.55) | -0.5539 | 0.4354 |
| ｐ1783 | 42 | 0.21 (0.11, 0.31) | 39 | 0.28 (0.17, 0.38) | 0.3954 | 0.4352 |
| ｐ21419 | 42 | 0.57 (0.49, 0.64) | 39 | 0.62 (0.54, 0.7) | 0.1193 | 0.4348 |
| ｐ16633 | 42 | 0.45 (0.09, 0.8) | 39 | 0.68 (0.31, 1.05) | 0.6019 | 0.4337 |
| ｐ21008 | 42 | 0.17 (0.07, 0.27) | 39 | 0.24 (0.13, 0.34) | 0.4559 | 0.4319 |
| ｐ245 | 42 | 0.86 (0.77, 0.96) | 39 | 0.8 (0.7, 0.9) | -0.1057 | 0.4293 |
| ｐ2992 | 42 | 0.26 (0.19, 0.33) | 39 | 0.3 (0.23, 0.38) | 0.2362 | 0.4286 |
| ｐ14804 | 42 | 0.23 (0.12, 0.35) | 39 | 0.16 (0.03, 0.28) | -0.5732 | 0.4279 |
| ｐ15284 | 42 | 0.38 (0.3, 0.46) | 39 | 0.33 (0.25, 0.41) | -0.2077 | 0.4264 |
| ｐ19452 | 42 | 0.56 (0.45, 0.67) | 39 | 0.63 (0.52, 0.75) | 0.1713 | 0.4255 |
| ｐ11836 | 42 | 0.62 (0.39, 0.86) | 39 | 0.47 (0.22, 0.72) | -0.4058 | 0.4248 |
| ｐ16097 | 42 | 1.68 (0.02, 3.35) | 39 | 0.61 (-1.12, 2.34) | -1.4626 | 0.4240 |
| ｐ12430 | 42 | 0.63 (0.33, 0.92) | 39 | 0.81 (0.51, 1.12) | 0.3786 | 0.4231 |
| ｐ22096 | 42 | 0.24 (0.16, 0.33) | 39 | 0.3 (0.21, 0.38) | 0.2855 | 0.4223 |
| ｐ12358 | 42 | 0.77 (-0.87, 2.4) | 39 | 1.81 (0.12, 3.51) | 1.2386 | 0.4200 |
| ｐ19965 | 42 | 0.57 (0.45, 0.7) | 39 | 0.65 (0.52, 0.78) | 0.1891 | 0.4185 |
| ｐ22555 | 42 | 0.38 (0.27, 0.48) | 39 | 0.44 (0.33, 0.56) | 0.2427 | 0.4181 |
| ｐ16896 | 42 | 0.16 (0.06, 0.25) | 39 | 0.22 (0.12, 0.31) | 0.4696 | 0.4181 |
| ｐ13628 | 42 | 0.26 (0.23, 0.29) | 39 | 0.24 (0.21, 0.27) | -0.1008 | 0.4144 |
| ｐ13310 | 42 | 1.1 (0.25, 1.94) | 39 | 1.63 (0.75, 2.51) | 0.5729 | 0.4143 |
| ｐ14861 | 42 | 0.24 (0.16, 0.32) | 39 | 0.19 (0.11, 0.28) | -0.3423 | 0.4142 |
| ｐ10871 | 42 | 0.43 (0.26, 0.61) | 39 | 0.55 (0.36, 0.73) | 0.3286 | 0.4139 |
| ｐ66 | 42 | 2.17 (-2.02, 6.36) | 39 | 4.81 (0.47, 9.16) | 1.1503 | 0.4136 |
| ｐ18828 | 42 | 1.75 (1.41, 2.09) | 39 | 1.53 (1.18, 1.88) | -0.1893 | 0.4134 |
| ｐ15467 | 42 | 0.49 (0.39, 0.58) | 39 | 0.54 (0.45, 0.64) | 0.1624 | 0.4131 |
| ｐ14811 | 42 | 0.69 (0.46, 0.92) | 39 | 0.54 (0.3, 0.78) | -0.3431 | 0.4112 |
| ｐ17384 | 42 | 0.34 (0.27, 0.4) | 39 | 0.38 (0.31, 0.45) | 0.1737 | 0.4104 |
| ｐ16560 | 42 | 0.87 (0.78, 0.96) | 39 | 0.93 (0.84, 1.02) | 0.0871 | 0.4067 |
| ｐ21063 | 42 | 0.66 (0.36, 0.96) | 39 | 0.48 (0.16, 0.79) | -0.4785 | 0.4064 |
| ｐ10134 | 42 | 0.32 (0.19, 0.45) | 39 | 0.24 (0.11, 0.37) | -0.4147 | 0.4056 |
| ｐ4918 | 42 | 0.42 (0.35, 0.49) | 39 | 0.47 (0.39, 0.54) | 0.1437 | 0.4051 |
| ｐ22044 | 42 | 0.45 (0.39, 0.52) | 39 | 0.41 (0.34, 0.48) | -0.1428 | 0.4045 |
| ｐ9485 | 42 | 0.18 (0.13, 0.23) | 39 | 0.15 (0.1, 0.2) | -0.2680 | 0.4025 |
| ｐ17862 | 41 | 0.38 (0.27, 0.49) | 39 | 0.31 (0.2, 0.43) | -0.2846 | 0.4014 |
| ｐ15543 | 42 | 0.14 (0.11, 0.18) | 39 | 0.17 (0.13, 0.21) | 0.2151 | 0.3989 |
| ｐ22951 | 42 | 0.53 (0.5, 0.55) | 39 | 0.51 (0.49, 0.54) | -0.0398 | 0.3987 |
| ｐ18200 | 42 | 0.34 (0.23, 0.45) | 39 | 0.27 (0.16, 0.38) | -0.3180 | 0.3984 |
| ｐ14393 | 42 | 0.69 (0.61, 0.78) | 39 | 0.75 (0.66, 0.84) | 0.1096 | 0.3972 |
| ｐ9538 | 42 | 0.18 (0.1, 0.26) | 39 | 0.23 (0.15, 0.31) | 0.3397 | 0.3970 |
| ｐ709 | 42 | 0.05 (0.01, 0.09) | 39 | 0.02 (-0.02, 0.07) | -1.0545 | 0.3966 |
| ｐ12735 | 42 | 0.18 (-0.06, 0.42) | 39 | 0.33 (0.08, 0.58) | 0.8604 | 0.3964 |
| ｐ21661 | 42 | 0.76 (0.61, 0.92) | 39 | 0.86 (0.7, 1.02) | 0.1696 | 0.3950 |
| ｐ7599 | 42 | 1.49 (0.64, 2.35) | 39 | 2.01 (1.13, 2.9) | 0.4308 | 0.3943 |
| ｐ3119 | 42 | 0.13 (0.05, 0.21) | 39 | 0.18 (0.1, 0.26) | 0.4425 | 0.3924 |
| ｐ6327 | 42 | 0.81 (0.78, 0.84) | 39 | 0.79 (0.76, 0.82) | -0.0304 | 0.3917 |
| ｐ17844 | 42 | 0.66 (0.64, 0.68) | 39 | 0.67 (0.65, 0.7) | 0.0278 | 0.3917 |
| ｐ3579 | 42 | 0.59 (0.39, 0.8) | 39 | 0.72 (0.51, 0.92) | 0.2680 | 0.3905 |
| ｐ21614 | 42 | 0.21 (0.04, 0.38) | 39 | 0.1 (-0.07, 0.28) | -0.9908 | 0.3893 |
| ｐ20687 | 42 | 0.54 (0.46, 0.62) | 39 | 0.5 (0.42, 0.58) | -0.1293 | 0.3892 |
| ｐ22633 | 42 | 0.17 (0.12, 0.23) | 39 | 0.2 (0.15, 0.26) | 0.2518 | 0.3892 |
| ｐ17975 | 42 | 0.34 (0.31, 0.37) | 39 | 0.32 (0.29, 0.35) | -0.0791 | 0.3887 |
| ｐ19406 | 42 | 0.03 (-0.18, 0.23) | 39 | 0.15 (-0.06, 0.36) | 2.4352 | 0.3882 |
| ｐ221 | 42 | 0.54 (-0.45, 1.54) | 39 | 1.14 (0.11, 2.17) | 1.0667 | 0.3876 |
| ｐ15445 | 41 | 0.22 (-0.01, 0.44) | 39 | 0.35 (0.12, 0.58) | 0.6985 | 0.3872 |
| ｐ22033 | 42 | 0.56 (0.52, 0.6) | 39 | 0.58 (0.54, 0.63) | 0.0631 | 0.3855 |
| ｐ9766 | 42 | 0.7 (0.68, 0.72) | 39 | 0.69 (0.67, 0.71) | -0.0262 | 0.3854 |
| ｐ15144 | 42 | 0.33 (-0.03, 0.68) | 39 | 0.54 (0.17, 0.91) | 0.7216 | 0.3845 |
| ｐ15680 | 42 | 1.59 (1.23, 1.95) | 39 | 1.38 (1, 1.75) | -0.2084 | 0.3840 |
| ｐ15754 | 42 | 0.18 (0.09, 0.27) | 39 | 0.23 (0.14, 0.33) | 0.3850 | 0.3828 |
| ｐ22651 | 42 | 0.18 (0.07, 0.29) | 39 | 0.24 (0.13, 0.36) | 0.4417 | 0.3827 |
| ｐ14787 | 42 | 0.46 (0.35, 0.57) | 39 | 0.52 (0.41, 0.64) | 0.1900 | 0.3824 |
| ｐ11106 | 42 | 0.53 (0.48, 0.59) | 39 | 0.5 (0.45, 0.56) | -0.0895 | 0.3806 |
| ｐ16974 | 42 | 0.42 (0.39, 0.45) | 39 | 0.4 (0.36, 0.43) | -0.0702 | 0.3796 |
| ｐ17225 | 38 | 0.2 (0.04, 0.35) | 39 | 0.11 (-0.05, 0.26) | -0.8767 | 0.3791 |
| ｐ12940 | 42 | 0.41 (0.3, 0.51) | 39 | 0.34 (0.23, 0.45) | -0.2399 | 0.3770 |
| ｐ15795 | 42 | 0.12 (0.1, 0.15) | 39 | 0.11 (0.08, 0.13) | -0.1913 | 0.3770 |
| ｐ22950 | 42 | 0.32 (0.21, 0.43) | 39 | 0.39 (0.27, 0.51) | 0.2750 | 0.3767 |
| ｐ17393 | 42 | 1.13 (-2.03, 4.29) | 39 | 2.98 (-0.3, 6.25) | 1.3992 | 0.3756 |
| ｐ19142 | 42 | 0.32 (-0.68, 1.33) | 39 | 0.91 (-0.13, 1.95) | 1.4899 | 0.3754 |
| ｐ22843 | 42 | 0.58 (0.52, 0.65) | 39 | 0.62 (0.55, 0.69) | 0.0910 | 0.3729 |
| ｐ18728 | 42 | 0.23 (0.02, 0.44) | 39 | 0.35 (0.13, 0.57) | 0.6160 | 0.3704 |
| ｐ6277 | 42 | 0.35 (0.31, 0.4) | 39 | 0.33 (0.28, 0.37) | -0.1058 | 0.3698 |
| ｐ9574 | 42 | 0.58 (-0.43, 1.58) | 39 | 1.15 (0.11, 2.2) | 0.9969 | 0.3658 |
| ｐ18479 | 42 | 0.52 (0.45, 0.6) | 39 | 0.57 (0.49, 0.65) | 0.1149 | 0.3641 |
| ｐ6865 | 42 | 0.36 (0.31, 0.41) | 39 | 0.39 (0.34, 0.44) | 0.1146 | 0.3638 |
| ｐ11234 | 42 | 0.15 (0.08, 0.22) | 39 | 0.11 (0.04, 0.18) | -0.4451 | 0.3627 |
| ｐ14740 | 42 | 0.71 (0.38, 1.04) | 39 | 0.52 (0.18, 0.86) | -0.4413 | 0.3620 |
| ｐ16020 | 42 | 0.63 (0.59, 0.67) | 39 | 0.61 (0.56, 0.65) | -0.0543 | 0.3619 |
| ｐ14684 | 42 | 0.42 (0.28, 0.56) | 39 | 0.34 (0.19, 0.49) | -0.3095 | 0.3613 |
| ｐ9 | 42 | 1.2 (1.06, 1.34) | 39 | 1.28 (1.13, 1.43) | 0.0923 | 0.3590 |
| ｐ13653 | 42 | 0.35 (0.2, 0.51) | 39 | 0.44 (0.28, 0.6) | 0.3217 | 0.3577 |
| ｐ8634 | 42 | 0.47 (0.35, 0.59) | 39 | 0.54 (0.42, 0.67) | 0.1924 | 0.3570 |
| ｐ959 | 42 | 0.93 (0.8, 1.07) | 39 | 1.01 (0.87, 1.15) | 0.1123 | 0.3569 |
| ｐ21264 | 42 | 0.56 (0.45, 0.66) | 39 | 0.62 (0.5, 0.73) | 0.1481 | 0.3567 |
| ｐ14155 | 42 | 0.29 (0.16, 0.43) | 39 | 0.37 (0.23, 0.51) | 0.3324 | 0.3564 |
| ｐ12864 | 42 | 0.56 (0.19, 0.93) | 39 | 0.77 (0.38, 1.16) | 0.4587 | 0.3554 |
| ｐ13681 | 42 | 0.81 (0.76, 0.87) | 39 | 0.78 (0.73, 0.84) | -0.0541 | 0.3554 |
| ｐ327 | 42 | 0.7 (0.54, 0.86) | 39 | 0.61 (0.44, 0.78) | -0.1980 | 0.3551 |
| ｐ862 | 42 | 0.51 (0.38, 0.65) | 39 | 0.44 (0.31, 0.58) | -0.2208 | 0.3537 |
| ｐ15643 | 42 | 0.72 (0.59, 0.84) | 39 | 0.65 (0.52, 0.78) | -0.1458 | 0.3518 |
| ｐ21336 | 42 | 0.37 (0.28, 0.46) | 39 | 0.42 (0.33, 0.51) | 0.1771 | 0.3516 |
| ｐ14952 | 42 | 0.85 (0.82, 0.89) | 39 | 0.88 (0.84, 0.92) | 0.0360 | 0.3497 |
| ｐ20863 | 42 | 0.8 (0.73, 0.87) | 39 | 0.84 (0.77, 0.9) | 0.0639 | 0.3492 |
| ｐ21186 | 42 | 0.59 (0.57, 0.62) | 39 | 0.61 (0.58, 0.63) | 0.0322 | 0.3490 |
| ｐ22077 | 42 | 0.13 (0.01, 0.24) | 39 | 0.19 (0.07, 0.31) | 0.5979 | 0.3489 |
| ｐ18909 | 42 | 0.93 (0.47, 1.38) | 39 | 1.18 (0.7, 1.65) | 0.3472 | 0.3483 |
| ｐ10638 | 42 | 0.28 (0.23, 0.33) | 39 | 0.25 (0.2, 0.3) | -0.1408 | 0.3483 |
| ｐ8993 | 42 | 0.18 (0.12, 0.24) | 39 | 0.21 (0.15, 0.27) | 0.2462 | 0.3481 |
| ｐ10601 | 42 | 0.37 (0.25, 0.5) | 39 | 0.3 (0.17, 0.43) | -0.2958 | 0.3480 |
| ｐ1253 | 42 | 0.82 (-0.73, 2.37) | 39 | 1.67 (0.06, 3.28) | 1.0320 | 0.3479 |
| ｐ17434 | 42 | 0.45 (0.22, 0.68) | 39 | 0.58 (0.34, 0.81) | 0.3534 | 0.3475 |
| ｐ18015 | 42 | 0.52 (0.5, 0.53) | 39 | 0.51 (0.5, 0.52) | -0.0196 | 0.3471 |
| ｐ23041 | 42 | 0.45 (0.32, 0.57) | 39 | 0.51 (0.39, 0.64) | 0.2006 | 0.3469 |
| ｐ19934 | 42 | 0.72 (0.61, 0.83) | 39 | 0.78 (0.67, 0.89) | 0.1146 | 0.3468 |
| ｐ18109 | 42 | 1.41 (1.23, 1.6) | 39 | 1.51 (1.32, 1.7) | 0.0993 | 0.3453 |
| ｐ20421 | 42 | 0.66 (0.55, 0.77) | 39 | 0.6 (0.49, 0.72) | -0.1368 | 0.3437 |
| ｐ15554 | 42 | 0.13 (0.01, 0.25) | 39 | 0.19 (0.07, 0.32) | 0.5936 | 0.3433 |
| ｐ13466 | 42 | 0.76 (0.74, 0.78) | 39 | 0.77 (0.75, 0.79) | 0.0187 | 0.3432 |
| ｐ22955 | 42 | 0.64 (0.55, 0.74) | 39 | 0.69 (0.6, 0.79) | 0.1092 | 0.3425 |
| ｐ8685 | 42 | 0.54 (0.4, 0.69) | 39 | 0.62 (0.47, 0.77) | 0.1926 | 0.3411 |
| ｐ22400 | 42 | 0.52 (-0.09, 1.12) | 39 | 0.84 (0.22, 1.47) | 0.7076 | 0.3407 |
| ｐ12415 | 42 | 0.35 (0.24, 0.45) | 39 | 0.4 (0.29, 0.51) | 0.2198 | 0.3405 |
| ｐ20829 | 42 | 0.29 (0.24, 0.34) | 39 | 0.27 (0.22, 0.31) | -0.1303 | 0.3402 |
| ｐ16363 | 42 | 0.6 (0.32, 0.88) | 39 | 0.75 (0.46, 1.04) | 0.3204 | 0.3389 |
| ｐ21179 | 42 | 0.6 (0.32, 0.88) | 39 | 0.75 (0.46, 1.04) | 0.3204 | 0.3388 |
| ｐ17640 | 42 | 0.62 (0.41, 0.83) | 39 | 0.74 (0.52, 0.96) | 0.2414 | 0.3388 |
| ｐ12572 | 42 | 0.24 (0.1, 0.38) | 39 | 0.16 (0.02, 0.31) | -0.5560 | 0.3381 |
| ｐ14640 | 42 | 0.6 (0.18, 1.03) | 39 | 0.83 (0.39, 1.28) | 0.4648 | 0.3376 |
| ｐ15902 | 42 | 0.29 (0.23, 0.34) | 39 | 0.26 (0.19, 0.32) | -0.1676 | 0.3373 |
| ｐ22083 | 42 | 0.47 (0.45, 0.49) | 39 | 0.48 (0.46, 0.5) | 0.0330 | 0.3370 |
| ｐ16364 | 42 | 0.12 (-0.04, 0.28) | 39 | 0.21 (0.04, 0.37) | 0.7571 | 0.3349 |
| ｐ15279 | 42 | 0.31 (0.22, 0.39) | 39 | 0.26 (0.17, 0.35) | -0.2287 | 0.3339 |
| ｐ13560 | 42 | 0.31 (0.12, 0.5) | 39 | 0.21 (0.01, 0.4) | -0.5661 | 0.3334 |
| ｐ20658 | 42 | 0.42 (0.33, 0.51) | 39 | 0.37 (0.27, 0.46) | -0.1773 | 0.3326 |
| ｐ10667 | 42 | 0.17 (0.1, 0.25) | 39 | 0.13 (0.06, 0.21) | -0.3761 | 0.3325 |
| ｐ19189 | 42 | 0.39 (0.35, 0.44) | 39 | 0.42 (0.37, 0.47) | 0.0898 | 0.3318 |
| ｐ16687 | 42 | 0.47 (0.34, 0.59) | 39 | 0.53 (0.4, 0.66) | 0.1870 | 0.3313 |
| ｐ19047 | 42 | 0.5 (0.46, 0.54) | 39 | 0.52 (0.48, 0.57) | 0.0612 | 0.3310 |
| ｐ13622 | 42 | 0.4 (0.24, 0.55) | 39 | 0.31 (0.16, 0.47) | -0.3307 | 0.3295 |
| ｐ12657 | 42 | 0.14 (0.09, 0.2) | 39 | 0.11 (0.06, 0.17) | -0.3183 | 0.3290 |
| ｐ17501 | 42 | 0.55 (0.43, 0.67) | 39 | 0.49 (0.36, 0.61) | -0.1770 | 0.3285 |
| ｐ13068 | 42 | 0.52 (0.5, 0.55) | 39 | 0.51 (0.48, 0.54) | -0.0397 | 0.3279 |
| ｐ17042 | 42 | 0.48 (0.39, 0.57) | 39 | 0.53 (0.44, 0.62) | 0.1356 | 0.3274 |
| ｐ780 | 42 | 0.46 (0.3, 0.62) | 39 | 0.54 (0.38, 0.71) | 0.2437 | 0.3272 |
| ｐ9546 | 42 | 0.37 (0.32, 0.42) | 39 | 0.4 (0.34, 0.45) | 0.1035 | 0.3266 |
| ｐ16720 | 42 | 0.32 (0.28, 0.36) | 39 | 0.34 (0.3, 0.38) | 0.0864 | 0.3264 |
| ｐ22518 | 42 | 0.57 (0.54, 0.61) | 39 | 0.59 (0.55, 0.63) | 0.0489 | 0.3262 |
| ｐ10088 | 42 | 0.58 (-0.06, 1.22) | 39 | 0.91 (0.25, 1.57) | 0.6543 | 0.3261 |
| ｐ4296 | 42 | 0.24 (0.14, 0.34) | 39 | 0.19 (0.08, 0.29) | -0.3688 | 0.3253 |
| ｐ6882 | 42 | 0.92 (0.9, 0.94) | 39 | 0.93 (0.91, 0.95) | 0.0153 | 0.3243 |
| ｐ1236 | 42 | 0.45 (0.31, 0.59) | 39 | 0.38 (0.23, 0.52) | -0.2543 | 0.3236 |
| ｐ15952 | 42 | 1.24 (1.06, 1.42) | 39 | 1.15 (0.96, 1.33) | -0.1119 | 0.3235 |
| ｐ14970 | 42 | 0.36 (0.27, 0.44) | 39 | 0.31 (0.23, 0.4) | -0.1849 | 0.3228 |
| ｐ19076 | 42 | 0.36 (0.32, 0.4) | 39 | 0.38 (0.34, 0.42) | 0.0840 | 0.3225 |
| ｐ21796 | 42 | 0.05 (0.02, 0.08) | 39 | 0.04 (0.01, 0.06) | -0.4934 | 0.3215 |
| ｐ18606 | 42 | 1.04 (0.89, 1.18) | 39 | 0.96 (0.81, 1.11) | -0.1098 | 0.3214 |
| ｐ22981 | 42 | 0.25 (-0.01, 0.52) | 39 | 0.39 (0.11, 0.66) | 0.6278 | 0.3200 |
| ｐ2428 | 42 | 0.27 (0.25, 0.29) | 39 | 0.26 (0.24, 0.28) | -0.0542 | 0.3197 |
| ｐ14335 | 42 | 1.23 (-0.38, 2.83) | 39 | 2.05 (0.39, 3.72) | 0.7419 | 0.3194 |
| ｐ14191 | 42 | 0.42 (0.31, 0.52) | 39 | 0.36 (0.26, 0.47) | -0.1980 | 0.3188 |
| ｐ22452 | 42 | 0.46 (0.34, 0.58) | 39 | 0.52 (0.39, 0.64) | 0.1833 | 0.3185 |
| ｐ10631 | 42 | 0.24 (0.08, 0.41) | 39 | 0.33 (0.16, 0.5) | 0.4253 | 0.3176 |
| ｐ20675 | 42 | 0.64 (0.37, 0.92) | 39 | 0.79 (0.5, 1.07) | 0.2858 | 0.3161 |
| ｐ22511 | 42 | 0.17 (0.15, 0.19) | 39 | 0.18 (0.16, 0.21) | 0.0885 | 0.3152 |
| ｐ17655 | 42 | 0.41 (0.36, 0.45) | 39 | 0.43 (0.38, 0.48) | 0.0798 | 0.3152 |
| ｐ12884 | 42 | 0.62 (0.51, 0.73) | 39 | 0.68 (0.56, 0.79) | 0.1271 | 0.3149 |
| ｐ14759 | 42 | 0.54 (0.47, 0.61) | 39 | 0.5 (0.43, 0.58) | -0.1020 | 0.3139 |
| ｐ22020 | 42 | 0.06 (0.01, 0.11) | 39 | 0.08 (0.03, 0.13) | 0.5082 | 0.3133 |
| ｐ22038 | 42 | 0.51 (0.48, 0.54) | 39 | 0.53 (0.5, 0.56) | 0.0428 | 0.3132 |
| ｐ15562 | 42 | 0.55 (0.49, 0.61) | 39 | 0.58 (0.52, 0.64) | 0.0724 | 0.3130 |
| ｐ15314 | 42 | 0.46 (0.39, 0.52) | 39 | 0.49 (0.42, 0.56) | 0.1001 | 0.3102 |
| ｐ20595 | 42 | 0.15 (0.1, 0.2) | 39 | 0.18 (0.12, 0.23) | 0.2284 | 0.3094 |
| ｐ15878 | 42 | 0.3 (0.26, 0.34) | 39 | 0.28 (0.24, 0.32) | -0.0974 | 0.3072 |
| ｐ16511 | 42 | 0.78 (0.75, 0.82) | 39 | 0.8 (0.77, 0.83) | 0.0292 | 0.3057 |
| ｐ7360 | 42 | 0.78 (0.75, 0.82) | 39 | 0.8 (0.77, 0.83) | 0.0292 | 0.3057 |
| ｐ16266 | 42 | 0.08 (-0.08, 0.24) | 39 | 0.16 (0, 0.33) | 0.9578 | 0.3052 |
| ｐ10203 | 42 | 0.28 (0.18, 0.39) | 39 | 0.33 (0.23, 0.44) | 0.2357 | 0.3050 |
| ｐ22251 | 42 | 0.72 (0.53, 0.92) | 39 | 0.63 (0.42, 0.83) | -0.2077 | 0.3030 |
| ｐ835 | 42 | 0.76 (0.64, 0.88) | 39 | 0.7 (0.57, 0.83) | -0.1211 | 0.3025 |
| ｐ22961 | 42 | 0.54 (0.3, 0.78) | 39 | 0.42 (0.17, 0.67) | -0.3572 | 0.3009 |
| ｐ17516 | 42 | 0.27 (0.17, 0.38) | 39 | 0.33 (0.22, 0.44) | 0.2532 | 0.3009 |
| ｐ16592 | 41 | 0.24 (0.13, 0.35) | 36 | 0.19 (0.07, 0.3) | -0.3609 | 0.3003 |
| ｐ22021 | 42 | 0.26 (0.04, 0.49) | 39 | 0.15 (-0.08, 0.39) | -0.7797 | 0.2993 |
| ｐ1246 | 42 | 0.13 (0.1, 0.15) | 39 | 0.14 (0.11, 0.17) | 0.1362 | 0.2953 |
| ｐ20036 | 42 | 0.21 (0.09, 0.32) | 39 | 0.15 (0.04, 0.27) | -0.4353 | 0.2953 |
| ｐ13635 | 42 | 0.24 (0.15, 0.34) | 39 | 0.2 (0.11, 0.3) | -0.2816 | 0.2934 |
| ｐ22771 | 42 | 0.63 (0.56, 0.69) | 39 | 0.66 (0.59, 0.73) | 0.0715 | 0.2932 |
| ｐ19744 | 42 | 0.54 (0.44, 0.64) | 39 | 0.59 (0.48, 0.69) | 0.1227 | 0.2922 |
| ｐ16346 | 42 | 0.17 (0.08, 0.27) | 39 | 0.13 (0.03, 0.23) | -0.4425 | 0.2890 |
| ｐ20161 | 42 | 0.62 (0.52, 0.73) | 39 | 0.67 (0.56, 0.78) | 0.1089 | 0.2886 |
| ｐ16552 | 42 | 0.05 (0.02, 0.07) | 39 | 0.06 (0.04, 0.09) | 0.3139 | 0.2883 |
| ｐ20361 | 42 | 0.25 (0.18, 0.33) | 39 | 0.22 (0.14, 0.3) | -0.2157 | 0.2879 |
| ｐ16777 | 42 | 0.03 (0, 0.06) | 39 | 0.01 (-0.02, 0.04) | -1.1756 | 0.2879 |
| ｐ17889 | 42 | 0.53 (0.4, 0.66) | 39 | 0.59 (0.46, 0.73) | 0.1560 | 0.2877 |
| ｐ1252 | 42 | 0.52 (0.23, 0.81) | 39 | 0.65 (0.35, 0.95) | 0.3355 | 0.2854 |
| ｐ22829 | 42 | 0.4 (0.3, 0.51) | 39 | 0.45 (0.34, 0.56) | 0.1629 | 0.2845 |
| ｐ9150 | 42 | 0.32 (0.3, 0.34) | 39 | 0.31 (0.29, 0.33) | -0.0394 | 0.2840 |
| ｐ10391 | 42 | 0.26 (0.19, 0.33) | 39 | 0.29 (0.22, 0.37) | 0.1793 | 0.2819 |
| ｐ18195 | 42 | 0.26 (0.19, 0.33) | 39 | 0.29 (0.22, 0.37) | 0.1793 | 0.2819 |
| ｐ18964 | 42 | 0.26 (0.19, 0.33) | 39 | 0.29 (0.22, 0.37) | 0.1793 | 0.2819 |
| ｐ13342 | 42 | 0.34 (0.15, 0.53) | 39 | 0.43 (0.23, 0.63) | 0.3313 | 0.2801 |
| ｐ22002 | 42 | 0.4 (0.27, 0.54) | 39 | 0.47 (0.32, 0.61) | 0.2121 | 0.2798 |
| ｐ7200 | 42 | 0.25 (0.13, 0.37) | 39 | 0.2 (0.07, 0.32) | -0.3620 | 0.2797 |
| ｐ19079 | 42 | 0.17 (0.05, 0.29) | 39 | 0.11 (-0.01, 0.24) | -0.5774 | 0.2796 |
| ｐ17672 | 42 | 0.89 (-0.11, 1.88) | 39 | 1.35 (0.31, 2.38) | 0.5989 | 0.2782 |
| ｐ332 | 42 | 1.26 (0.67, 1.84) | 39 | 0.99 (0.38, 1.59) | -0.3458 | 0.2781 |
| ｐ17599 | 42 | 0.32 (0.16, 0.49) | 39 | 0.4 (0.23, 0.57) | 0.2978 | 0.2774 |
| ｐ17863 | 42 | 0.7 (0.44, 0.96) | 39 | 0.82 (0.55, 1.09) | 0.2245 | 0.2773 |
| ｐ2310 | 42 | 0.08 (0.02, 0.14) | 39 | 0.11 (0.05, 0.17) | 0.4036 | 0.2772 |
| ｐ18830 | 42 | 0.47 (0.4, 0.54) | 39 | 0.44 (0.37, 0.51) | -0.1034 | 0.2764 |
| ｐ10625 | 42 | 0.27 (0.14, 0.39) | 39 | 0.32 (0.19, 0.45) | 0.2785 | 0.2752 |
| ｐ44 | 42 | 0.4 (0.29, 0.51) | 39 | 0.35 (0.23, 0.46) | -0.1979 | 0.2748 |
| ｐ17554 | 42 | 0.48 (0.3, 0.67) | 39 | 0.56 (0.37, 0.76) | 0.2319 | 0.2747 |
| ｐ21700 | 42 | 0.68 (0.6, 0.75) | 39 | 0.64 (0.57, 0.72) | -0.0732 | 0.2717 |
| ｐ17744 | 42 | 0.44 (0.37, 0.51) | 39 | 0.41 (0.33, 0.48) | -0.1106 | 0.2699 |
| ｐ14383 | 42 | 0.59 (0.08, 1.1) | 33 | 0.83 (0.26, 1.4) | 0.4863 | 0.2689 |
| ｐ14355 | 42 | 0.38 (0.22, 0.54) | 39 | 0.45 (0.28, 0.61) | 0.2493 | 0.2685 |
| ｐ20261 | 42 | 0.38 (0.22, 0.54) | 39 | 0.45 (0.28, 0.61) | 0.2493 | 0.2685 |
| ｐ21930 | 42 | 0.92 (0.77, 1.08) | 39 | 0.85 (0.69, 1.01) | -0.1117 | 0.2667 |
| ｐ20180 | 42 | 0.08 (-0.03, 0.19) | 39 | 0.13 (0.02, 0.24) | 0.6752 | 0.2658 |
| ｐ20033 | 42 | 0.57 (0.49, 0.65) | 39 | 0.6 (0.52, 0.68) | 0.0883 | 0.2657 |
| ｐ14799 | 42 | 0.47 (0.38, 0.57) | 39 | 0.43 (0.33, 0.53) | -0.1328 | 0.2650 |
| ｐ14924 | 42 | 0.39 (0.3, 0.49) | 39 | 0.35 (0.26, 0.45) | -0.1568 | 0.2646 |
| ｐ11233 | 42 | 0.16 (0.08, 0.25) | 39 | 0.13 (0.04, 0.21) | -0.3655 | 0.2644 |
| ｐ13424 | 42 | 0.28 (0.09, 0.47) | 39 | 0.19 (0, 0.39) | -0.5178 | 0.2640 |
| ｐ4731 | 42 | 0.38 (0.27, 0.49) | 39 | 0.33 (0.22, 0.44) | -0.1917 | 0.2638 |
| ｐ1331 | 42 | 1.13 (-0.14, 2.4) | 39 | 1.69 (0.37, 3) | 0.5784 | 0.2638 |
| ｐ4483 | 42 | 0.19 (0.09, 0.29) | 39 | 0.24 (0.13, 0.34) | 0.2996 | 0.2635 |
| ｐ16279 | 42 | 0.37 (0.24, 0.51) | 39 | 0.31 (0.17, 0.46) | -0.2564 | 0.2633 |
| ｐ14941 | 42 | 1.07 (0.91, 1.24) | 39 | 1.15 (0.97, 1.32) | 0.0951 | 0.2624 |
| ｐ8234 | 42 | 0.4 (0.34, 0.45) | 39 | 0.42 (0.36, 0.48) | 0.0827 | 0.2597 |
| ｐ15123 | 42 | 0.71 (0.34, 1.08) | 39 | 0.55 (0.17, 0.93) | -0.3634 | 0.2573 |
| ｐ20191 | 42 | 0.39 (0.31, 0.48) | 39 | 0.43 (0.34, 0.52) | 0.1307 | 0.2569 |
| ｐ21770 | 42 | 0.05 (0.04, 0.06) | 39 | 0.04 (0.04, 0.05) | -0.1144 | 0.2568 |
| ｐ9579 | 42 | 0.5 (0.13, 0.88) | 39 | 0.66 (0.28, 1.05) | 0.4009 | 0.2565 |
| ｐ15187 | 42 | 0.43 (0.39, 0.48) | 39 | 0.42 (0.37, 0.46) | -0.0594 | 0.2565 |
| ｐ15359 | 42 | 0.4 (0.31, 0.49) | 39 | 0.44 (0.35, 0.54) | 0.1338 | 0.2563 |
| ｐ22654 | 42 | 0.4 (0.3, 0.5) | 39 | 0.44 (0.34, 0.55) | 0.1436 | 0.2553 |
| ｐ15922 | 42 | 0.23 (0.12, 0.34) | 39 | 0.28 (0.16, 0.39) | 0.2632 | 0.2546 |
| ｐ12765 | 42 | 0.39 (0.33, 0.45) | 39 | 0.36 (0.3, 0.43) | -0.1020 | 0.2537 |
| ｐ21668 | 42 | 0.47 (0.3, 0.63) | 39 | 0.54 (0.37, 0.71) | 0.2022 | 0.2532 |
| ｐ17312 | 42 | 0.28 (0.18, 0.39) | 39 | 0.33 (0.22, 0.44) | 0.2102 | 0.2520 |
| ｐ14812 | 42 | 0.66 (0.42, 0.89) | 39 | 0.56 (0.31, 0.8) | -0.2390 | 0.2519 |
| ｐ10236 | 41 | 0.91 (0.14, 1.68) | 39 | 0.59 (-0.2, 1.37) | -0.6307 | 0.2518 |
| ｐ19248 | 42 | 0.36 (0.25, 0.46) | 39 | 0.4 (0.29, 0.51) | 0.1750 | 0.2516 |
| ｐ15078 | 42 | 0.5 (0.15, 0.85) | 39 | 0.65 (0.29, 1.01) | 0.3689 | 0.2494 |
| ｐ14788 | 42 | 0.44 (0.23, 0.65) | 39 | 0.35 (0.13, 0.57) | -0.3263 | 0.2483 |
| ｐ7506 | 42 | 0.35 (0.27, 0.43) | 39 | 0.31 (0.23, 0.4) | -0.1470 | 0.2476 |
| ｐ95 | 41 | 0.89 (0.59, 1.18) | 36 | 0.76 (0.45, 1.08) | -0.2179 | 0.2466 |
| ｐ22073 | 42 | 0.32 (0.3, 0.35) | 39 | 0.33 (0.31, 0.36) | 0.0417 | 0.2449 |
| ｐ4845 | 42 | 0.23 (0.16, 0.31) | 39 | 0.26 (0.19, 0.34) | 0.1845 | 0.2446 |
| ｐ22426 | 42 | 0.09 (0.03, 0.15) | 39 | 0.12 (0.05, 0.18) | 0.3712 | 0.2443 |
| ｐ2410 | 42 | 0.41 (0.25, 0.58) | 39 | 0.35 (0.18, 0.52) | -0.2572 | 0.2441 |
| ｐ17955 | 42 | 0.32 (0.23, 0.4) | 39 | 0.28 (0.18, 0.37) | -0.1801 | 0.2434 |
| ｐ15622 | 42 | 0.31 (0.1, 0.52) | 39 | 0.39 (0.18, 0.61) | 0.3536 | 0.2426 |
| ｐ15659 | 41 | 2.81 (0.36, 5.26) | 39 | 1.81 (-0.7, 4.32) | -0.6304 | 0.2410 |
| ｐ2558 | 42 | 1.11 (0.84, 1.37) | 39 | 1 (0.73, 1.27) | -0.1471 | 0.2409 |
| ｐ15176 | 42 | 1.04 (-0.46, 2.55) | 39 | 1.66 (0.1, 3.22) | 0.6667 | 0.2408 |
| ｐ10929 | 42 | 0.42 (0.34, 0.49) | 39 | 0.39 (0.31, 0.46) | -0.1090 | 0.2407 |
| ｐ15519 | 42 | 0.56 (0.48, 0.63) | 39 | 0.59 (0.51, 0.67) | 0.0781 | 0.2393 |
| ｐ4699 | 42 | 0.74 (0.62, 0.85) | 39 | 0.69 (0.57, 0.81) | -0.0940 | 0.2391 |
| ｐ16694 | 42 | 0.92 (-0.22, 2.06) | 39 | 1.39 (0.2, 2.57) | 0.5856 | 0.2390 |
| ｐ10354 | 42 | 0.13 (0.06, 0.21) | 39 | 0.16 (0.08, 0.24) | 0.3031 | 0.2386 |
| ｐ19739 | 42 | 0.73 (0.48, 0.99) | 39 | 0.84 (0.57, 1.1) | 0.1882 | 0.2382 |
| ｐ12354 | 42 | 0.2 (0.12, 0.27) | 39 | 0.23 (0.15, 0.31) | 0.2122 | 0.2373 |
| ｐ19292 | 42 | 0.79 (0.74, 0.85) | 39 | 0.77 (0.72, 0.83) | -0.0399 | 0.2368 |
| ｐ13698 | 42 | 0.7 (0.57, 0.83) | 39 | 0.75 (0.62, 0.88) | 0.1018 | 0.2363 |
| ｐ16613 | 42 | 0.21 (0.15, 0.28) | 39 | 0.19 (0.12, 0.26) | -0.1835 | 0.2359 |
| ｐ14658 | 42 | 0.4 (0.24, 0.56) | 39 | 0.34 (0.17, 0.5) | -0.2472 | 0.2339 |
| ｐ11906 | 42 | 0.78 (0.45, 1.12) | 39 | 0.92 (0.57, 1.26) | 0.2251 | 0.2309 |
| ｐ14280 | 42 | 0.16 (0.13, 0.2) | 39 | 0.18 (0.14, 0.21) | 0.1153 | 0.2304 |
| ｐ14376 | 42 | 0.33 (0.12, 0.54) | 39 | 0.25 (0.03, 0.46) | -0.4157 | 0.2302 |
| ｐ11409 | 42 | 0.28 (0.15, 0.41) | 39 | 0.33 (0.2, 0.47) | 0.2414 | 0.2293 |
| ｐ21672 | 42 | 0.64 (0.43, 0.85) | 39 | 0.72 (0.5, 0.94) | 0.1751 | 0.2293 |
| ｐ867 | 42 | 0.11 (-0.01, 0.22) | 39 | 0.15 (0.03, 0.27) | 0.5001 | 0.2279 |
| ｐ22941 | 42 | 0.25 (0.17, 0.34) | 39 | 0.29 (0.2, 0.37) | 0.1711 | 0.2275 |
| ｐ163 | 42 | 0.54 (0.34, 0.74) | 39 | 0.46 (0.25, 0.67) | -0.2246 | 0.2266 |
| ｐ865 | 42 | 0.24 (0.11, 0.37) | 39 | 0.19 (0.06, 0.32) | -0.3263 | 0.2208 |
| ｐ15861 | 42 | 0.75 (0.67, 0.84) | 39 | 0.72 (0.63, 0.81) | -0.0630 | 0.2202 |
| ｐ22666 | 42 | 0.7 (0.68, 0.73) | 39 | 0.71 (0.69, 0.74) | 0.0194 | 0.2197 |
| ｐ8394 | 42 | 0.29 (0.11, 0.46) | 39 | 0.22 (0.04, 0.4) | -0.3809 | 0.2195 |
| ｐ15020 | 42 | 0.06 (0.01, 0.12) | 39 | 0.08 (0.03, 0.14) | 0.4113 | 0.2193 |
| ｐ18012 | 42 | 0.16 (0.11, 0.21) | 39 | 0.18 (0.13, 0.23) | 0.1582 | 0.2193 |
| ｐ18567 | 42 | 0.16 (0.12, 0.21) | 39 | 0.18 (0.13, 0.23) | 0.1392 | 0.2193 |
| ｐ17120 | 42 | 0.66 (0.57, 0.74) | 39 | 0.69 (0.6, 0.78) | 0.0685 | 0.2193 |
| ｐ15824 | 42 | 0.54 (0.48, 0.6) | 39 | 0.56 (0.5, 0.63) | 0.0582 | 0.2193 |
| ｐ15098 | 42 | 0.63 (0.62, 0.64) | 39 | 0.63 (0.62, 0.64) | 0.0093 | 0.2193 |
| ｐ19504 | 42 | 0.97 (0.96, 0.98) | 39 | 0.97 (0.97, 0.98) | 0.0037 | 0.2193 |
| ｐ15761 | 42 | 1.72 (1.72, 1.72) | 39 | 1.72 (1.72, 1.72) | 0.0003 | 0.2193 |
| ｐ10485 | 42 | 0.66 (0.66, 0.67) | 39 | 0.67 (0.66, 0.67) | 0.0045 | 0.2192 |
| ｐ14983 | 42 | 0.82 (0.8, 0.83) | 39 | 0.82 (0.81, 0.84) | 0.0085 | 0.2192 |
| ｐ12398 | 42 | 0.13 (0.05, 0.21) | 39 | 0.16 (0.07, 0.25) | 0.3135 | 0.2192 |
| ｐ8625 | 42 | 0.06 (0, 0.11) | 39 | 0.08 (0.02, 0.14) | 0.4471 | 0.2192 |
| ｐ10048 | 42 | 0.54 (0.52, 0.56) | 39 | 0.55 (0.53, 0.57) | 0.0182 | 0.2192 |
| ｐ19046 | 42 | 0.5 (0.27, 0.73) | 39 | 0.59 (0.34, 0.83) | 0.2330 | 0.2170 |
| ｐ18629 | 42 | 0.18 (0.09, 0.28) | 39 | 0.22 (0.12, 0.32) | 0.2607 | 0.2162 |
| ｐ12274 | 42 | 0.34 (0.22, 0.45) | 39 | 0.29 (0.18, 0.41) | -0.1936 | 0.2148 |
| ｐ7513 | 42 | 0.76 (0.14, 1.37) | 39 | 0.53 (-0.11, 1.17) | -0.5187 | 0.2148 |
| ｐ21998 | 42 | 0.02 (-0.01, 0.04) | 39 | 0.01 (-0.02, 0.03) | -1.2101 | 0.2124 |
| ｐ15719 | 41 | 0.68 (0.37, 1) | 39 | 0.8 (0.47, 1.12) | 0.2245 | 0.2111 |
| ｐ17781 | 42 | 0.07 (-0.01, 0.15) | 39 | 0.04 (-0.05, 0.12) | -0.8581 | 0.2108 |
| ｐ14810 | 42 | 0.49 (0.32, 0.66) | 39 | 0.55 (0.37, 0.73) | 0.1716 | 0.2105 |
| ｐ323 | 42 | 0.7 (0.54, 0.86) | 39 | 0.76 (0.59, 0.93) | 0.1172 | 0.2104 |
| ｐ15087 | 42 | 0.29 (0.26, 0.33) | 39 | 0.28 (0.25, 0.32) | -0.0601 | 0.2100 |
| ｐ6040 | 42 | 0.29 (0.16, 0.41) | 39 | 0.24 (0.11, 0.37) | -0.2457 | 0.2097 |
| ｐ21131 | 42 | 0.5 (0.47, 0.53) | 39 | 0.51 (0.48, 0.54) | 0.0296 | 0.2089 |
| ｐ11422 | 42 | 0.28 (0.16, 0.4) | 39 | 0.24 (0.11, 0.36) | -0.2410 | 0.2082 |
| ｐ9780 | 42 | 0.28 (0.07, 0.49) | 39 | 0.36 (0.14, 0.58) | 0.3494 | 0.2079 |
| ｐ15429 | 42 | 0.34 (0.19, 0.49) | 39 | 0.4 (0.24, 0.55) | 0.2065 | 0.2048 |
| ｐ11245 | 42 | 0.44 (0.25, 0.64) | 39 | 0.51 (0.31, 0.72) | 0.2128 | 0.2047 |
| ｐ9441 | 42 | 0.38 (0.23, 0.53) | 39 | 0.32 (0.17, 0.48) | -0.2250 | 0.2047 |
| ｐ8991 | 42 | 0.26 (0.16, 0.35) | 39 | 0.22 (0.12, 0.32) | -0.2046 | 0.2047 |
| ｐ16501 | 42 | 0.7 (0.54, 0.86) | 39 | 0.64 (0.48, 0.81) | -0.1233 | 0.2045 |
| ｐ320 | 42 | 0.93 (0.76, 1.09) | 39 | 0.87 (0.7, 1.04) | -0.0924 | 0.2030 |
| ｐ22589 | 42 | 0.71 (0.47, 0.96) | 39 | 0.8 (0.54, 1.06) | 0.1656 | 0.2021 |
| ｐ22945 | 42 | 0.42 (0.36, 0.49) | 39 | 0.4 (0.33, 0.47) | -0.0784 | 0.2017 |
| ｐ20944 | 42 | 0.2 (0.09, 0.31) | 39 | 0.24 (0.12, 0.36) | 0.2623 | 0.2009 |
| ｐ20868 | 42 | 0.5 (0.42, 0.57) | 39 | 0.52 (0.44, 0.6) | 0.0744 | 0.2001 |
| ｐ15289 | 42 | 0.25 (0.15, 0.35) | 39 | 0.22 (0.11, 0.32) | -0.2188 | 0.2000 |
| ｐ14769 | 42 | 0.38 (0.22, 0.54) | 39 | 0.32 (0.15, 0.49) | -0.2317 | 0.1983 |
| ｐ15512 | 42 | 0.42 (0.37, 0.47) | 39 | 0.41 (0.35, 0.46) | -0.0603 | 0.1980 |
| ｐ14758 | 42 | 0.18 (0.13, 0.23) | 39 | 0.2 (0.14, 0.25) | 0.1347 | 0.1975 |
| ｐ20682 | 42 | 0.8 (0.71, 0.89) | 39 | 0.83 (0.74, 0.93) | 0.0557 | 0.1969 |
| ｐ19027 | 42 | 0.15 (0.06, 0.24) | 39 | 0.12 (0.03, 0.22) | -0.3254 | 0.1966 |
| ｐ22080 | 42 | 0.68 (0.65, 0.72) | 39 | 0.67 (0.63, 0.71) | -0.0259 | 0.1965 |
| ｐ22512 | 42 | 0.54 (0.51, 0.57) | 39 | 0.53 (0.5, 0.56) | -0.0264 | 0.1957 |
| ｐ856 | 42 | 1.92 (0.47, 3.36) | 39 | 2.41 (0.91, 3.91) | 0.3316 | 0.1957 |
| ｐ127 | 39 | 0.47 (0.2, 0.74) | 39 | 0.38 (0.11, 0.65) | -0.3097 | 0.1956 |
| ｐ14292 | 42 | 0.39 (0.33, 0.45) | 39 | 0.41 (0.35, 0.47) | 0.0716 | 0.1955 |
| ｐ15810 | 42 | 0.19 (0.14, 0.23) | 39 | 0.17 (0.13, 0.22) | -0.1192 | 0.1954 |
| ｐ10234 | 42 | 0.76 (0.58, 0.95) | 39 | 0.7 (0.5, 0.89) | -0.1262 | 0.1939 |
| ｐ19048 | 42 | 0.24 (0.19, 0.3) | 39 | 0.26 (0.21, 0.32) | 0.1047 | 0.1939 |
| ｐ22071 | 42 | 0.76 (0.7, 0.82) | 39 | 0.78 (0.72, 0.84) | 0.0372 | 0.1939 |
| ｐ18368 | 42 | 0.64 (0.43, 0.85) | 39 | 0.71 (0.49, 0.93) | 0.1538 | 0.1934 |
| ｐ16807 | 42 | 0.17 (0.12, 0.22) | 39 | 0.15 (0.1, 0.2) | -0.1542 | 0.1934 |
| ｐ13668 | 42 | 0.34 (0.21, 0.47) | 39 | 0.38 (0.25, 0.52) | 0.1746 | 0.1933 |
| ｐ16467 | 42 | 0.23 (0.16, 0.3) | 39 | 0.21 (0.14, 0.28) | -0.1501 | 0.1888 |
| ｐ13394 | 42 | 0.23 (0.16, 0.3) | 39 | 0.21 (0.14, 0.28) | -0.1501 | 0.1888 |
| ｐ20013 | 42 | 0.23 (0.16, 0.3) | 39 | 0.21 (0.14, 0.28) | -0.1501 | 0.1888 |
| ｐ14722 | 42 | 0.57 (0.49, 0.65) | 39 | 0.54 (0.45, 0.63) | -0.0719 | 0.1887 |
| ｐ5270 | 42 | 0.51 (0.38, 0.63) | 39 | 0.47 (0.34, 0.59) | -0.1203 | 0.1881 |
| ｐ19917 | 42 | 0.39 (0.26, 0.52) | 39 | 0.43 (0.3, 0.57) | 0.1518 | 0.1880 |
| ｐ22104 | 42 | 0.19 (0.12, 0.25) | 39 | 0.21 (0.14, 0.27) | 0.1533 | 0.1878 |
| ｐ10357 | 42 | 0.65 (0.5, 0.81) | 39 | 0.71 (0.55, 0.87) | 0.1078 | 0.1867 |
| ｐ20030 | 42 | 0.68 (0.43, 0.93) | 39 | 0.76 (0.51, 1.02) | 0.1631 | 0.1864 |
| ｐ12247 | 42 | 1.3 (0.57, 2.03) | 39 | 1.54 (0.78, 2.3) | 0.2431 | 0.1856 |
| ｐ21689 | 42 | 0.42 (0.35, 0.49) | 39 | 0.44 (0.37, 0.52) | 0.0802 | 0.1852 |
| ｐ22682 | 42 | 0.28 (0.05, 0.51) | 39 | 0.2 (-0.04, 0.45) | -0.4591 | 0.1849 |
| ｐ18353 | 42 | 0.21 (0.13, 0.3) | 39 | 0.24 (0.16, 0.33) | 0.1720 | 0.1846 |
| ｐ21129 | 42 | 0.39 (0.29, 0.49) | 39 | 0.36 (0.26, 0.46) | -0.1189 | 0.1829 |
| ｐ15220 | 42 | 0.28 (0.2, 0.36) | 39 | 0.25 (0.17, 0.33) | -0.1373 | 0.1824 |
| ｐ875 | 42 | 0.28 (0.19, 0.37) | 39 | 0.31 (0.21, 0.4) | 0.1409 | 0.1815 |
| ｐ266 | 42 | 0.47 (0.24, 0.69) | 39 | 0.4 (0.16, 0.63) | -0.2411 | 0.1807 |
| ｐ15766 | 42 | 0.4 (0.21, 0.58) | 39 | 0.34 (0.15, 0.53) | -0.2289 | 0.1806 |
| ｐ22050 | 42 | 0.04 (-0.02, 0.1) | 39 | 0.06 (0, 0.12) | 0.5815 | 0.1804 |
| ｐ1220 | 42 | 0.42 (0.2, 0.64) | 39 | 0.49 (0.26, 0.72) | 0.2245 | 0.1800 |
| ｐ22686 | 42 | 0.7 (0.44, 0.96) | 39 | 0.62 (0.35, 0.89) | -0.1826 | 0.1800 |
| ｐ13959 | 42 | 0.47 (0.4, 0.55) | 39 | 0.45 (0.37, 0.53) | -0.0745 | 0.1799 |
| ｐ259 | 42 | 1.06 (0.87, 1.25) | 39 | 1 (0.8, 1.2) | -0.0865 | 0.1789 |
| ｐ16248 | 42 | 0.12 (0.08, 0.15) | 39 | 0.11 (0.07, 0.14) | -0.1293 | 0.1778 |
| ｐ20984 | 42 | 0.28 (0.22, 0.34) | 39 | 0.3 (0.24, 0.37) | 0.0986 | 0.1774 |
| ｐ17616 | 42 | 0.52 (0.46, 0.59) | 39 | 0.54 (0.47, 0.61) | 0.0548 | 0.1770 |
| ｐ13469 | 42 | 0.12 (0.08, 0.15) | 39 | 0.1 (0.07, 0.14) | -0.1371 | 0.1766 |
| ｐ6976 | 41 | 0.26 (0.07, 0.45) | 39 | 0.32 (0.12, 0.51) | 0.2937 | 0.1746 |
| ｐ18659 | 42 | 0.35 (0.29, 0.41) | 39 | 0.37 (0.31, 0.43) | 0.0734 | 0.1744 |
| ｐ21120 | 42 | 0.54 (0.46, 0.63) | 39 | 0.57 (0.48, 0.66) | 0.0685 | 0.1738 |
| ｐ15246 | 42 | 0.44 (0.25, 0.63) | 39 | 0.5 (0.3, 0.7) | 0.1824 | 0.1734 |
| ｐ10555 | 42 | 0.7 (-0.1, 1.5) | 39 | 0.95 (0.12, 1.78) | 0.4335 | 0.1729 |
| ｐ249 | 42 | 0.84 (0.54, 1.13) | 39 | 0.93 (0.62, 1.24) | 0.1487 | 0.1728 |
| ｐ19014 | 42 | 0.38 (0.25, 0.51) | 39 | 0.34 (0.2, 0.48) | -0.1639 | 0.1725 |
| ｐ21337 | 42 | 0.61 (0.46, 0.76) | 39 | 0.65 (0.5, 0.81) | 0.1042 | 0.1721 |
| ｐ12952 | 42 | 0.59 (0.5, 0.68) | 39 | 0.56 (0.47, 0.66) | -0.0711 | 0.1718 |
| ｐ15037 | 42 | 0.49 (0.27, 0.72) | 39 | 0.56 (0.33, 0.8) | 0.1892 | 0.1713 |
| ｐ18236 | 42 | 0.39 (0.23, 0.55) | 39 | 0.44 (0.28, 0.61) | 0.1692 | 0.1713 |
| ｐ16692 | 42 | 0.7 (0.7, 0.7) | 39 | 0.7 (0.69, 0.7) | -0.0018 | 0.1713 |
| ｐ15737 | 42 | 1.03 (1.02, 1.03) | 39 | 1.02 (1.02, 1.03) | -0.0030 | 0.1713 |
| ｐ19634 | 42 | 0.8 (0.79, 0.8) | 39 | 0.79 (0.79, 0.8) | -0.0036 | 0.1713 |
| ｐ16023 | 42 | 2.21 (2.18, 2.24) | 39 | 2.2 (2.17, 2.23) | -0.0058 | 0.1713 |
| ｐ15845 | 42 | 1.1 (1.07, 1.12) | 39 | 1.09 (1.07, 1.11) | -0.0089 | 0.1713 |
| ｐ19583 | 42 | 1.1 (1.07, 1.12) | 39 | 1.09 (1.07, 1.11) | -0.0089 | 0.1713 |
| ｐ18275 | 42 | 0.54 (0.52, 0.56) | 39 | 0.53 (0.51, 0.55) | -0.0176 | 0.1713 |
| ｐ15044 | 42 | 0.26 (0.24, 0.28) | 39 | 0.26 (0.24, 0.28) | -0.0335 | 0.1713 |
| ｐ17515 | 42 | 0.33 (0.2, 0.46) | 39 | 0.29 (0.16, 0.43) | -0.1853 | 0.1713 |
| ｐ5397 | 42 | 0.77 (0.76, 0.78) | 39 | 0.77 (0.76, 0.78) | -0.0058 | 0.1713 |
| ｐ6941 | 42 | 0.51 (0.49, 0.53) | 39 | 0.5 (0.49, 0.52) | -0.0146 | 0.1713 |
| ｐ22357 | 42 | 1.44 (1.34, 1.54) | 39 | 1.41 (1.31, 1.51) | -0.0305 | 0.1713 |
| ｐ22225 | 42 | 0.06 (-0.02, 0.13) | 39 | 0.03 (-0.04, 0.11) | -0.7208 | 0.1713 |
| ｐ8384 | 42 | 0.25 (0.21, 0.3) | 39 | 0.24 (0.19, 0.29) | -0.0835 | 0.1713 |
| ｐ12058 | 42 | 0.15 (0.12, 0.18) | 39 | 0.14 (0.11, 0.17) | -0.0980 | 0.1713 |
| ｐ21742 | 42 | 0.31 (0.24, 0.38) | 39 | 0.29 (0.21, 0.36) | -0.1036 | 0.1713 |
| ｐ6964 | 42 | 0.47 (0.45, 0.5) | 39 | 0.47 (0.44, 0.5) | -0.0275 | 0.1713 |
| ｐ11327 | 42 | 0.52 (0.48, 0.57) | 39 | 0.51 (0.47, 0.55) | -0.0360 | 0.1713 |
| ｐ7100 | 42 | 0.45 (0.43, 0.46) | 39 | 0.44 (0.43, 0.46) | -0.0135 | 0.1713 |
| ｐ14980 | 42 | 1.02 (1.01, 1.03) | 39 | 1.02 (1.01, 1.03) | -0.0048 | 0.1713 |
| ｐ14669 | 42 | 0.54 (0.54, 0.55) | 39 | 0.54 (0.54, 0.55) | -0.0018 | 0.1713 |
| ｐ21972 | 42 | 1.77 (1.76, 1.78) | 39 | 1.77 (1.76, 1.78) | -0.0023 | 0.1713 |
| ｐ22470 | 42 | 0.82 (0.7, 0.93) | 39 | 0.85 (0.73, 0.97) | 0.0621 | 0.1710 |
| ｐ22065 | 42 | 0.17 (0.07, 0.27) | 39 | 0.2 (0.1, 0.3) | 0.2354 | 0.1708 |
| ｐ241 | 42 | 0.77 (0.57, 0.97) | 39 | 0.83 (0.63, 1.04) | 0.1086 | 0.1707 |
| ｐ22075 | 42 | 0.34 (0.3, 0.38) | 39 | 0.33 (0.28, 0.37) | -0.0567 | 0.1689 |
| ｐ12741 | 42 | 0.73 (0.49, 0.97) | 39 | 0.81 (0.56, 1.06) | 0.1357 | 0.1688 |
| ｐ22670 | 42 | 0.22 (0.09, 0.35) | 39 | 0.18 (0.05, 0.32) | -0.2705 | 0.1660 |
| ｐ14990 | 42 | 0.48 (-0.05, 1.02) | 39 | 0.32 (-0.23, 0.88) | -0.5773 | 0.1653 |
| ｐ16145 | 42 | 0.3 (0.1, 0.5) | 39 | 0.24 (0.03, 0.45) | -0.3178 | 0.1646 |
| ｐ15250 | 42 | 0.3 (0.27, 0.33) | 39 | 0.29 (0.26, 0.32) | -0.0432 | 0.1644 |
| ｐ13512 | 42 | 0.45 (0.38, 0.53) | 39 | 0.43 (0.35, 0.51) | -0.0732 | 0.1643 |
| ｐ9200 | 42 | 0.21 (0.13, 0.29) | 39 | 0.23 (0.15, 0.32) | 0.1532 | 0.1635 |
| ｐ1900 | 42 | 0.41 (0.29, 0.53) | 39 | 0.44 (0.32, 0.57) | 0.1152 | 0.1614 |
| ｐ17383 | 42 | 0.48 (0.34, 0.62) | 39 | 0.52 (0.38, 0.67) | 0.1170 | 0.1610 |
| ｐ5289 | 42 | 0.12 (0.03, 0.21) | 39 | 0.15 (0.06, 0.24) | 0.2789 | 0.1610 |
| ｐ11451 | 42 | 0.48 (0.32, 0.65) | 39 | 0.53 (0.36, 0.7) | 0.1339 | 0.1590 |
| ｐ2175 | 42 | 0.62 (0.43, 0.82) | 39 | 0.57 (0.37, 0.77) | -0.1338 | 0.1589 |
| ｐ10413 | 42 | 0.12 (0.03, 0.21) | 39 | 0.15 (0.05, 0.24) | 0.2820 | 0.1584 |
| ｐ19844 | 42 | 0.85 (0.69, 1) | 39 | 0.8 (0.64, 0.97) | -0.0774 | 0.1581 |
| ｐ2167 | 41 | 0.46 (0.27, 0.65) | 33 | 0.52 (0.3, 0.73) | 0.1660 | 0.1574 |
| ｐ21864 | 42 | 0.53 (0.14, 0.91) | 39 | 0.42 (0.02, 0.81) | -0.3299 | 0.1560 |
| ｐ13063 | 42 | 0.68 (0.38, 0.99) | 39 | 0.77 (0.45, 1.08) | 0.1689 | 0.1542 |
| ｐ22027 | 42 | 0.92 (0.84, 1) | 39 | 0.95 (0.86, 1.03) | 0.0343 | 0.1537 |
| ｐ340 | 42 | 0.68 (0.56, 0.81) | 39 | 0.72 (0.59, 0.85) | 0.0697 | 0.1531 |
| ｐ13460 | 42 | 0.31 (0.24, 0.39) | 39 | 0.33 (0.26, 0.41) | 0.0922 | 0.1531 |
| ｐ14667 | 42 | 0.07 (0.03, 0.12) | 39 | 0.06 (0.01, 0.11) | -0.2820 | 0.1526 |
| ｐ19316 | 42 | 0.46 (0.29, 0.63) | 39 | 0.51 (0.33, 0.68) | 0.1405 | 0.1524 |
| ｐ11178 | 42 | 0.09 (0.05, 0.12) | 39 | 0.1 (0.06, 0.13) | 0.1569 | 0.1523 |
| ｐ22087 | 42 | 0.33 (0.18, 0.49) | 39 | 0.37 (0.21, 0.53) | 0.1751 | 0.1514 |
| ｐ19473 | 42 | 0.17 (0.12, 0.21) | 39 | 0.15 (0.11, 0.2) | -0.1126 | 0.1510 |
| ｐ4430 | 42 | 0.07 (-0.01, 0.14) | 39 | 0.05 (-0.03, 0.12) | -0.5048 | 0.1503 |
| ｐ22162 | 42 | 0.79 (0.74, 0.84) | 39 | 0.78 (0.72, 0.83) | -0.0264 | 0.1501 |
| ｐ15283 | 42 | 0.19 (0.08, 0.3) | 39 | 0.16 (0.05, 0.27) | -0.2416 | 0.1500 |
| ｐ22015 | 42 | 0.3 (0.21, 0.39) | 39 | 0.28 (0.19, 0.37) | -0.1214 | 0.1494 |
| ｐ18410 | 42 | 0.45 (0.4, 0.5) | 39 | 0.44 (0.39, 0.49) | -0.0436 | 0.1488 |
| ｐ17783 | 42 | 0.19 (0.08, 0.29) | 39 | 0.22 (0.11, 0.33) | 0.2039 | 0.1477 |
| ｐ15855 | 42 | 1.23 (0.24, 2.22) | 39 | 0.97 (-0.06, 1.99) | -0.3472 | 0.1464 |
| ｐ15193 | 42 | 0.13 (0.01, 0.26) | 33 | 0.17 (0.03, 0.31) | 0.3384 | 0.1462 |
| ｐ5949 | 42 | 0.56 (0.37, 0.75) | 39 | 0.51 (0.31, 0.7) | -0.1369 | 0.1454 |
| ｐ16427 | 42 | 0.55 (0.39, 0.71) | 39 | 0.51 (0.34, 0.67) | -0.1142 | 0.1454 |
| ｐ14403 | 42 | 0.44 (0.33, 0.55) | 39 | 0.47 (0.36, 0.59) | 0.0929 | 0.1444 |
| ｐ22489 | 42 | 0.07 (0.01, 0.14) | 39 | 0.09 (0.02, 0.16) | 0.3108 | 0.1418 |
| ｐ15655 | 42 | 0.12 (0.08, 0.15) | 39 | 0.12 (0.09, 0.16) | 0.1024 | 0.1418 |
| ｐ20940 | 42 | 0.46 (0.41, 0.52) | 39 | 0.48 (0.42, 0.54) | 0.0453 | 0.1409 |
| ｐ15002 | 42 | 0.66 (0.02, 1.3) | 39 | 0.82 (0.16, 1.48) | 0.3207 | 0.1409 |
| ｐ13617 | 42 | 0.18 (0.1, 0.26) | 39 | 0.2 (0.11, 0.28) | 0.1621 | 0.1406 |
| ｐ18231 | 42 | 0.1 (0.08, 0.12) | 39 | 0.11 (0.09, 0.12) | 0.0611 | 0.1404 |
| ｐ14779 | 42 | 0.38 (0.25, 0.5) | 39 | 0.34 (0.22, 0.47) | -0.1263 | 0.1404 |
| ｐ946 | 42 | 0.48 (0.3, 0.67) | 39 | 0.43 (0.24, 0.62) | -0.1501 | 0.1403 |
| ｐ21629 | 42 | 0.46 (0.31, 0.6) | 39 | 0.49 (0.34, 0.64) | 0.1118 | 0.1401 |
| ｐ20990 | 42 | 0.45 (0.36, 0.54) | 39 | 0.47 (0.38, 0.57) | 0.0734 | 0.1397 |
| ｐ15508 | 42 | 0.46 (0.31, 0.61) | 39 | 0.42 (0.26, 0.58) | -0.1259 | 0.1385 |
| ｐ17999 | 42 | 0.45 (0.43, 0.47) | 39 | 0.45 (0.43, 0.47) | 0.0165 | 0.1376 |
| ｐ19020 | 42 | 0.48 (0.41, 0.54) | 39 | 0.46 (0.4, 0.53) | -0.0488 | 0.1374 |
| ｐ22095 | 42 | 0.04 (-0.04, 0.11) | 39 | 0.06 (-0.02, 0.13) | 0.6105 | 0.1372 |
| ｐ22325 | 42 | 0.42 (0.32, 0.52) | 39 | 0.4 (0.3, 0.5) | -0.0849 | 0.1369 |
| ｐ18786 | 42 | 0.69 (0.61, 0.78) | 39 | 0.67 (0.58, 0.76) | -0.0467 | 0.1367 |
| ｐ22063 | 42 | 0.65 (0.56, 0.75) | 39 | 0.67 (0.58, 0.77) | 0.0516 | 0.1362 |
| ｐ10389 | 42 | 0.31 (0.1, 0.52) | 39 | 0.36 (0.14, 0.58) | 0.2213 | 0.1342 |
| ｐ407 | 42 | 0.67 (0.47, 0.88) | 39 | 0.72 (0.51, 0.94) | 0.1055 | 0.1342 |
| ｐ22380 | 42 | 0.23 (0.04, 0.43) | 39 | 0.28 (0.08, 0.48) | 0.2652 | 0.1341 |
| ｐ8650 | 42 | 0.3 (0.2, 0.41) | 39 | 0.33 (0.22, 0.44) | 0.1185 | 0.1330 |
| ｐ17802 | 41 | 0.37 (0.24, 0.51) | 39 | 0.34 (0.2, 0.48) | -0.1331 | 0.1327 |
| ｐ21430 | 42 | 0.57 (0.5, 0.63) | 39 | 0.55 (0.49, 0.62) | -0.0395 | 0.1311 |
| ｐ12808 | 42 | 0.36 (0.23, 0.49) | 39 | 0.39 (0.25, 0.52) | 0.1220 | 0.1311 |
| ｐ8009 | 41 | 0.72 (0.21, 1.23) | 39 | 0.84 (0.32, 1.36) | 0.2258 | 0.1311 |
| ｐ14732 | 42 | 1.08 (-0.01, 2.18) | 39 | 1.35 (0.21, 2.48) | 0.3146 | 0.1310 |
| ｐ17258 | 42 | 0.28 (0.23, 0.34) | 39 | 0.3 (0.24, 0.35) | 0.0655 | 0.1310 |
| ｐ14763 | 42 | 0.12 (-0.01, 0.25) | 39 | 0.15 (0.02, 0.28) | 0.3352 | 0.1309 |
| ｐ421 | 42 | 0.8 (0.67, 0.93) | 39 | 0.83 (0.69, 0.96) | 0.0552 | 0.1306 |
| ｐ20381 | 42 | 0.28 (0.15, 0.41) | 39 | 0.31 (0.17, 0.45) | 0.1548 | 0.1305 |
| ｐ21665 | 42 | 0.56 (0.46, 0.66) | 39 | 0.59 (0.48, 0.69) | 0.0623 | 0.1298 |
| ｐ17460 | 42 | 0.16 (0.06, 0.26) | 39 | 0.18 (0.08, 0.28) | 0.2059 | 0.1296 |
| ｐ20907 | 42 | 0.08 (0.01, 0.15) | 39 | 0.1 (0.03, 0.17) | 0.2646 | 0.1294 |
| ｐ17665 | 42 | 3.25 (-0.46, 6.96) | 39 | 4.14 (0.28, 7.99) | 0.3475 | 0.1291 |
| ｐ12714 | 41 | 0.69 (0.39, 0.99) | 39 | 0.62 (0.31, 0.92) | -0.1574 | 0.1283 |
| ｐ17048 | 42 | 0.08 (0.04, 0.12) | 39 | 0.07 (0.03, 0.11) | -0.1802 | 0.1280 |
| ｐ16632 | 42 | 0.19 (0.12, 0.26) | 39 | 0.18 (0.1, 0.25) | -0.1298 | 0.1278 |
| ｐ21619 | 42 | 0.43 (0.32, 0.53) | 39 | 0.45 (0.34, 0.56) | 0.0834 | 0.1277 |
| ｐ18463 | 42 | 0.36 (0.18, 0.53) | 39 | 0.4 (0.22, 0.57) | 0.1555 | 0.1277 |
| ｐ19799 | 42 | 0.35 (0.17, 0.54) | 39 | 0.4 (0.21, 0.59) | 0.1676 | 0.1275 |
| ｐ13140 | 42 | 0.43 (0.32, 0.54) | 39 | 0.41 (0.29, 0.52) | -0.0872 | 0.1263 |
| ｐ16116 | 42 | 0.4 (0.22, 0.57) | 39 | 0.36 (0.18, 0.54) | -0.1556 | 0.1258 |
| ｐ14984 | 42 | 0.4 (0.24, 0.56) | 39 | 0.44 (0.28, 0.61) | 0.1275 | 0.1257 |
| ｐ1301 | 42 | 0.8 (0.2, 1.4) | 39 | 0.66 (0.04, 1.28) | -0.2752 | 0.1253 |
| ｐ15245 | 42 | 0.39 (0.26, 0.53) | 39 | 0.36 (0.23, 0.5) | -0.1139 | 0.1238 |
| ｐ22068 | 42 | 0.5 (0.38, 0.62) | 39 | 0.53 (0.41, 0.65) | 0.0756 | 0.1237 |
| ｐ14650 | 42 | 0.43 (0.32, 0.55) | 39 | 0.41 (0.29, 0.53) | -0.0914 | 0.1219 |
| ｐ17940 | 42 | 0.3 (0.26, 0.33) | 39 | 0.31 (0.27, 0.34) | 0.0362 | 0.1218 |
| ｐ19555 | 42 | 0.24 (0.19, 0.28) | 39 | 0.23 (0.18, 0.27) | -0.0664 | 0.1212 |
| ｐ18543 | 42 | 1.18 (0.95, 1.41) | 39 | 1.23 (0.99, 1.47) | 0.0624 | 0.1210 |
| ｐ15382 | 42 | 0.66 (0.33, 0.98) | 39 | 0.73 (0.4, 1.07) | 0.1513 | 0.1209 |
| ｐ16090 | 42 | 0.66 (0.33, 0.98) | 39 | 0.73 (0.4, 1.07) | 0.1513 | 0.1209 |
| ｐ22018 | 42 | 0.02 (-0.02, 0.06) | 39 | 0.03 (-0.01, 0.07) | 0.5295 | 0.1206 |
| ｐ20041 | 42 | 0.67 (0.46, 0.88) | 39 | 0.62 (0.41, 0.84) | -0.1041 | 0.1201 |
| ｐ16348 | 42 | 0.36 (0.24, 0.47) | 39 | 0.33 (0.21, 0.45) | -0.1055 | 0.1196 |
| ｐ22043 | 42 | 0.09 (0.06, 0.13) | 39 | 0.1 (0.06, 0.14) | 0.1170 | 0.1189 |
| ｐ5284 | 42 | 0.1 (0.05, 0.15) | 39 | 0.11 (0.06, 0.17) | 0.1509 | 0.1187 |
| ｐ22559 | 42 | 0.44 (0.34, 0.55) | 39 | 0.47 (0.36, 0.57) | 0.0706 | 0.1183 |
| ｐ21815 | 42 | 0.5 (0.37, 0.63) | 39 | 0.47 (0.34, 0.6) | -0.0830 | 0.1183 |
| ｐ18156 | 42 | 0.3 (0.2, 0.41) | 39 | 0.33 (0.22, 0.44) | 0.1060 | 0.1171 |
| ｐ15068 | 42 | 1.59 (1.49, 1.68) | 39 | 1.57 (1.47, 1.67) | -0.0192 | 0.1163 |
| ｐ19059 | 42 | 0.34 (0.28, 0.41) | 39 | 0.36 (0.29, 0.42) | 0.0573 | 0.1158 |
| ｐ3965 | 42 | 0.5 (0.25, 0.75) | 39 | 0.56 (0.3, 0.82) | 0.1467 | 0.1149 |
| ｐ21942 | 42 | 0.25 (0.16, 0.33) | 39 | 0.26 (0.18, 0.35) | 0.0991 | 0.1129 |
| ｐ21398 | 42 | 0.69 (0.33, 1.04) | 39 | 0.61 (0.24, 0.98) | -0.1650 | 0.1116 |
| ｐ14737 | 42 | 0.52 (0.36, 0.67) | 39 | 0.48 (0.33, 0.64) | -0.0901 | 0.1095 |
| ｐ11840 | 42 | 0.5 (0.34, 0.67) | 39 | 0.47 (0.3, 0.64) | -0.1010 | 0.1093 |
| ｐ13742 | 42 | 0.37 (0.23, 0.5) | 39 | 0.34 (0.2, 0.48) | -0.1112 | 0.1089 |
| ｐ11439 | 42 | 0.54 (0.4, 0.67) | 39 | 0.56 (0.43, 0.7) | 0.0714 | 0.1079 |
| ｐ19758 | 42 | 0.55 (0.44, 0.66) | 39 | 0.58 (0.46, 0.69) | 0.0563 | 0.1070 |
| ｐ15 | 42 | 0.34 (0.22, 0.47) | 39 | 0.37 (0.24, 0.5) | 0.1031 | 0.1068 |
| ｐ22959 | 42 | 0.37 (0.27, 0.47) | 39 | 0.35 (0.25, 0.45) | -0.0794 | 0.1068 |
| ｐ16806 | 42 | 0.43 (0.22, 0.64) | 39 | 0.47 (0.25, 0.69) | 0.1367 | 0.1062 |
| ｐ4961 | 42 | 0.4 (0.36, 0.45) | 39 | 0.39 (0.34, 0.44) | -0.0344 | 0.1060 |
| ｐ17146 | 42 | 0.36 (0.23, 0.5) | 39 | 0.34 (0.2, 0.48) | -0.1099 | 0.1048 |
| ｐ18335 | 42 | 0.23 (0.13, 0.33) | 39 | 0.21 (0.11, 0.32) | -0.1260 | 0.1046 |
| ｐ14482 | 42 | 0.23 (0.13, 0.33) | 39 | 0.21 (0.11, 0.32) | -0.1260 | 0.1046 |
| ｐ15770 | 42 | 0.57 (0.53, 0.6) | 39 | 0.56 (0.52, 0.6) | -0.0182 | 0.1037 |
| ｐ11944 | 42 | 0.51 (0.47, 0.55) | 39 | 0.5 (0.46, 0.54) | -0.0203 | 0.1027 |
| ｐ17376 | 42 | 0.36 (0.26, 0.46) | 39 | 0.34 (0.24, 0.44) | -0.0782 | 0.1009 |
| ｐ20630 | 42 | 0.28 (0.16, 0.4) | 39 | 0.26 (0.13, 0.38) | -0.1250 | 0.0990 |
| ｐ6396 | 42 | 0.28 (0.16, 0.4) | 39 | 0.26 (0.13, 0.38) | -0.1250 | 0.0990 |
| ｐ10668 | 42 | 0.16 (0.1, 0.22) | 39 | 0.15 (0.09, 0.21) | -0.1039 | 0.0987 |
| ｐ22793 | 42 | 0.3 (0.19, 0.41) | 39 | 0.32 (0.21, 0.44) | 0.0969 | 0.0986 |
| ｐ6415 | 42 | 0.15 (0.09, 0.2) | 39 | 0.16 (0.1, 0.22) | 0.1054 | 0.0981 |
| ｐ6383 | 42 | 0.4 (0.27, 0.52) | 39 | 0.37 (0.24, 0.5) | -0.0886 | 0.0971 |
| ｐ14387 | 42 | 0.56 (0.38, 0.73) | 39 | 0.59 (0.41, 0.77) | 0.0813 | 0.0964 |
| ｐ21239 | 42 | 0.04 (0.01, 0.06) | 39 | 0.04 (0.02, 0.07) | 0.1665 | 0.0959 |
| ｐ17064 | 42 | 0.04 (0.01, 0.06) | 39 | 0.04 (0.02, 0.07) | 0.1665 | 0.0959 |
| ｐ22710 | 42 | 0.41 (0.28, 0.54) | 39 | 0.43 (0.3, 0.57) | 0.0786 | 0.0957 |
| ｐ2421 | 42 | 0.63 (0.54, 0.72) | 39 | 0.64 (0.55, 0.74) | 0.0374 | 0.0956 |
| ｐ14786 | 42 | 0.56 (0.38, 0.73) | 39 | 0.53 (0.34, 0.71) | -0.0837 | 0.0944 |
| ｐ22730 | 42 | 0.53 (0.49, 0.57) | 39 | 0.54 (0.49, 0.58) | 0.0198 | 0.0942 |
| ｐ18020 | 42 | 0.24 (0.21, 0.26) | 39 | 0.24 (0.21, 0.27) | 0.0296 | 0.0935 |
| ｐ816 | 42 | 0.81 (0.61, 1) | 39 | 0.77 (0.57, 0.97) | -0.0625 | 0.0928 |
| ｐ17623 | 42 | 0.62 (0.39, 0.86) | 39 | 0.58 (0.34, 0.82) | -0.0991 | 0.0923 |
| ｐ22607 | 42 | 0.45 (0.28, 0.61) | 39 | 0.42 (0.25, 0.59) | -0.0951 | 0.0922 |
| ｐ11073 | 42 | 0.55 (0.47, 0.64) | 39 | 0.57 (0.48, 0.65) | 0.0367 | 0.0920 |
| ｐ9388 | 42 | 0.32 (0.12, 0.52) | 39 | 0.29 (0.08, 0.49) | -0.1640 | 0.0906 |
| ｐ22462 | 42 | 0.32 (0.2, 0.44) | 39 | 0.34 (0.22, 0.47) | 0.0899 | 0.0900 |
| ｐ6642 | 42 | 0.58 (0.18, 0.98) | 39 | 0.64 (0.23, 1.06) | 0.1616 | 0.0892 |
| ｐ15260 | 42 | 0.45 (0.34, 0.55) | 39 | 0.43 (0.32, 0.54) | -0.0596 | 0.0891 |
| ｐ11372 | 42 | 0.14 (0.03, 0.24) | 39 | 0.15 (0.05, 0.26) | 0.1734 | 0.0881 |
| ｐ22756 | 42 | 0.23 (0.08, 0.37) | 39 | 0.25 (0.1, 0.4) | 0.1413 | 0.0846 |
| ｐ12270 | 42 | 0.41 (0.34, 0.47) | 39 | 0.4 (0.33, 0.47) | -0.0391 | 0.0843 |
| ｐ23076 | 42 | 0.87 (0.79, 0.94) | 39 | 0.85 (0.77, 0.93) | -0.0207 | 0.0836 |
| ｐ21912 | 42 | 0.6 (0.28, 0.93) | 39 | 0.66 (0.32, 1) | 0.1199 | 0.0835 |
| ｐ14749 | 42 | 0.48 (-0.3, 1.26) | 39 | 0.36 (-0.45, 1.16) | -0.4253 | 0.0816 |
| ｐ7185 | 42 | 0.34 (0.21, 0.47) | 39 | 0.36 (0.23, 0.49) | 0.0839 | 0.0816 |
| ｐ864 | 42 | 1.27 (0.63, 1.92) | 39 | 1.17 (0.5, 1.84) | -0.1194 | 0.0816 |
| ｐ15394 | 42 | 0.49 (0.4, 0.57) | 39 | 0.5 (0.41, 0.59) | 0.0393 | 0.0806 |
| ｐ13579 | 42 | 0.8 (0.64, 0.95) | 39 | 0.82 (0.66, 0.98) | 0.0421 | 0.0804 |
| ｐ2287 | 42 | 0.46 (0.29, 0.64) | 39 | 0.44 (0.25, 0.62) | -0.0888 | 0.0801 |
| ｐ861 | 42 | 0.45 (0.41, 0.49) | 39 | 0.46 (0.42, 0.5) | 0.0182 | 0.0796 |
| ｐ22456 | 42 | 0.31 (0.1, 0.51) | 39 | 0.28 (0.07, 0.49) | -0.1534 | 0.0792 |
| ｐ20271 | 42 | 0.15 (0.08, 0.22) | 39 | 0.16 (0.08, 0.23) | 0.1032 | 0.0790 |
| ｐ22491 | 42 | 0.38 (0.22, 0.54) | 39 | 0.41 (0.24, 0.57) | 0.0889 | 0.0786 |
| ｐ15268 | 42 | 0.69 (0.29, 1.09) | 39 | 0.63 (0.22, 1.05) | -0.1315 | 0.0783 |
| ｐ15876 | 42 | 0.17 (0.08, 0.25) | 39 | 0.18 (0.09, 0.27) | 0.1118 | 0.0776 |
| ｐ21098 | 42 | 0.22 (0.08, 0.37) | 39 | 0.24 (0.09, 0.39) | 0.1313 | 0.0763 |
| ｐ22962 | 42 | 0.5 (0.47, 0.53) | 39 | 0.5 (0.46, 0.53) | -0.0146 | 0.0754 |
| ｐ20131 | 42 | 0.51 (0.15, 0.86) | 39 | 0.56 (0.19, 0.92) | 0.1394 | 0.0754 |
| ｐ16547 | 42 | 0.31 (0.28, 0.35) | 39 | 0.31 (0.27, 0.35) | -0.0249 | 0.0753 |
| ｐ16259 | 42 | 0.06 (0, 0.12) | 39 | 0.05 (-0.01, 0.11) | -0.2438 | 0.0752 |
| ｐ21001 | 42 | 0.37 (0.25, 0.48) | 39 | 0.35 (0.23, 0.47) | -0.0678 | 0.0748 |
| ｐ828 | 42 | 0.65 (0.52, 0.78) | 39 | 0.67 (0.53, 0.81) | 0.0416 | 0.0739 |
| ｐ16580 | 42 | 0.41 (0.14, 0.68) | 39 | 0.44 (0.16, 0.72) | 0.1304 | 0.0732 |
| ｐ22796 | 42 | 0.42 (0.33, 0.5) | 39 | 0.43 (0.34, 0.52) | 0.0411 | 0.0729 |
| ｐ9537 | 42 | 0.15 (0.07, 0.22) | 39 | 0.14 (0.06, 0.22) | -0.1045 | 0.0714 |
| ｐ20000 | 42 | 0.15 (0.07, 0.22) | 39 | 0.14 (0.06, 0.22) | -0.1045 | 0.0714 |
| ｐ15215 | 42 | 0.5 (0.45, 0.56) | 39 | 0.5 (0.44, 0.55) | -0.0202 | 0.0709 |
| ｐ12686 | 42 | 0.23 (0.09, 0.38) | 39 | 0.25 (0.11, 0.4) | 0.1142 | 0.0702 |
| ｐ16083 | 42 | 0.72 (0.55, 0.88) | 39 | 0.69 (0.52, 0.87) | -0.0473 | 0.0700 |
| ｐ13727 | 42 | 0.37 (0.16, 0.58) | 39 | 0.4 (0.18, 0.62) | 0.1063 | 0.0698 |
| ｐ18342 | 42 | 0.7 (0.57, 0.83) | 39 | 0.72 (0.58, 0.85) | 0.0362 | 0.0693 |
| ｐ19180 | 42 | 0.29 (0.25, 0.33) | 39 | 0.28 (0.24, 0.33) | -0.0282 | 0.0692 |
| ｐ22652 | 42 | 0.64 (0.59, 0.68) | 39 | 0.64 (0.6, 0.69) | 0.0138 | 0.0686 |
| ｐ18994 | 42 | 0.28 (0.05, 0.5) | 39 | 0.25 (0.01, 0.48) | -0.1663 | 0.0683 |
| ｐ13873 | 42 | 0.28 (0.05, 0.5) | 39 | 0.25 (0.01, 0.48) | -0.1663 | 0.0683 |
| ｐ63 | 42 | 0.31 (0.06, 0.57) | 39 | 0.28 (0.01, 0.54) | -0.1647 | 0.0676 |
| ｐ17328 | 42 | 0.44 (0.36, 0.52) | 39 | 0.43 (0.35, 0.51) | -0.0346 | 0.0672 |
| ｐ21425 | 42 | 0.32 (0.23, 0.41) | 39 | 0.31 (0.21, 0.4) | -0.0556 | 0.0672 |
| ｐ21542 | 42 | 0.3 (0.22, 0.39) | 39 | 0.29 (0.2, 0.38) | -0.0535 | 0.0662 |
| ｐ14607 | 42 | 0.6 (0.58, 0.62) | 39 | 0.61 (0.59, 0.63) | 0.0062 | 0.0654 |
| ｐ13155 | 42 | 0.42 (0.28, 0.55) | 39 | 0.4 (0.26, 0.54) | -0.0596 | 0.0646 |
| ｐ372 | 42 | 0.85 (0.41, 1.3) | 39 | 0.8 (0.34, 1.26) | -0.0980 | 0.0646 |
| ｐ269 | 42 | 0.7 (0.62, 0.79) | 39 | 0.69 (0.6, 0.78) | -0.0219 | 0.0635 |
| ｐ19344 | 42 | 0.53 (0.34, 0.72) | 39 | 0.55 (0.35, 0.75) | 0.0642 | 0.0634 |
| ｐ19493 | 42 | 0.62 (0.6, 0.63) | 39 | 0.61 (0.6, 0.62) | -0.0031 | 0.0628 |
| ｐ15236 | 42 | 0.35 (0.28, 0.42) | 39 | 0.36 (0.29, 0.43) | 0.0344 | 0.0625 |
| ｐ16828 | 42 | 0.56 (0.51, 0.61) | 39 | 0.56 (0.51, 0.61) | 0.0149 | 0.0618 |
| ｐ20125 | 42 | 0.36 (0.25, 0.46) | 39 | 0.34 (0.23, 0.45) | -0.0538 | 0.0615 |
| ｐ14738 | 42 | 0.44 (0.28, 0.61) | 39 | 0.43 (0.26, 0.59) | -0.0651 | 0.0612 |
| ｐ1257 | 42 | 0.7 (0.45, 0.96) | 39 | 0.74 (0.47, 1) | 0.0616 | 0.0609 |
| ｐ14852 | 42 | 0.19 (0.04, 0.33) | 39 | 0.2 (0.05, 0.36) | 0.1294 | 0.0607 |
| ｐ19843 | 42 | 0.41 (0.26, 0.56) | 39 | 0.43 (0.27, 0.58) | 0.0600 | 0.0602 |
| ｐ18713 | 42 | 0.02 (0, 0.04) | 39 | 0.02 (0, 0.04) | -0.1466 | 0.0600 |
| ｐ18661 | 42 | 0.67 (0.37, 0.98) | 39 | 0.64 (0.32, 0.96) | -0.0796 | 0.0597 |
| ｐ612 | 42 | 0.74 (0.56, 0.93) | 39 | 0.72 (0.53, 0.92) | -0.0434 | 0.0596 |
| ｐ22935 | 42 | 0.42 (0.32, 0.53) | 39 | 0.41 (0.31, 0.52) | -0.0412 | 0.0593 |
| ｐ22082 | 42 | 0.04 (0.03, 0.05) | 39 | 0.04 (0.03, 0.05) | -0.0487 | 0.0591 |
| ｐ16413 | 42 | 0.63 (0.39, 0.88) | 39 | 0.6 (0.35, 0.86) | -0.0660 | 0.0586 |
| ｐ9447 | 42 | 0.48 (0.14, 0.83) | 39 | 0.45 (0.09, 0.8) | -0.1206 | 0.0578 |
| ｐ15451 | 42 | 0.58 (0.38, 0.78) | 39 | 0.6 (0.4, 0.81) | 0.0539 | 0.0567 |
| ｐ10465 | 42 | 0.56 (0.47, 0.65) | 39 | 0.57 (0.47, 0.66) | 0.0246 | 0.0545 |
| ｐ210 | 42 | 0.85 (0.67, 1.03) | 39 | 0.83 (0.65, 1.02) | -0.0324 | 0.0536 |
| ｐ7094 | 42 | 0.4 (0.24, 0.55) | 39 | 0.38 (0.22, 0.54) | -0.0603 | 0.0533 |
| ｐ14864 | 42 | 0.26 (0.19, 0.33) | 39 | 0.25 (0.18, 0.33) | -0.0435 | 0.0532 |
| ｐ24 | 42 | 0.48 (0.42, 0.54) | 39 | 0.49 (0.43, 0.55) | 0.0179 | 0.0525 |
| ｐ14330 | 42 | 0.4 (0.28, 0.53) | 39 | 0.39 (0.26, 0.52) | -0.0471 | 0.0519 |
| ｐ15762 | 42 | 0.07 (0, 0.14) | 39 | 0.08 (0, 0.15) | 0.1428 | 0.0518 |
| ｐ18222 | 42 | 0.31 (0.22, 0.4) | 39 | 0.32 (0.23, 0.41) | 0.0414 | 0.0518 |
| ｐ15590 | 42 | 0.92 (0.43, 1.4) | 39 | 0.87 (0.37, 1.37) | -0.0797 | 0.0514 |
| ｐ14860 | 42 | 0.41 (0.28, 0.54) | 39 | 0.42 (0.28, 0.55) | 0.0462 | 0.0513 |
| ｐ12287 | 42 | 0.64 (0.32, 0.95) | 39 | 0.67 (0.34, 1) | 0.0706 | 0.0510 |
| ｐ15110 | 42 | 0.47 (0.3, 0.65) | 39 | 0.46 (0.28, 0.64) | -0.0538 | 0.0499 |
| ｐ16024 | 42 | 0.46 (0.37, 0.56) | 39 | 0.45 (0.36, 0.55) | -0.0293 | 0.0492 |
| ｐ22943 | 42 | 0.2 (0.06, 0.33) | 39 | 0.21 (0.07, 0.35) | 0.0945 | 0.0489 |
| ｐ4409 | 42 | 0.36 (0.28, 0.45) | 39 | 0.37 (0.28, 0.47) | 0.0338 | 0.0489 |
| ｐ15294 | 42 | 0.24 (0.19, 0.29) | 39 | 0.24 (0.19, 0.29) | -0.0283 | 0.0486 |
| ｐ22782 | 42 | 0.35 (0.22, 0.48) | 39 | 0.37 (0.23, 0.5) | 0.0499 | 0.0476 |
| ｐ22631 | 42 | 0.73 (0.47, 0.98) | 39 | 0.7 (0.44, 0.97) | -0.0490 | 0.0476 |
| ｐ15722 | 42 | 1.69 (0.46, 2.93) | 39 | 1.58 (0.3, 2.86) | -0.1029 | 0.0475 |
| ｐ14281 | 42 | 0.81 (0.55, 1.06) | 39 | 0.83 (0.57, 1.09) | 0.0419 | 0.0472 |
| ｐ16331 | 42 | 0.8 (0.71, 0.89) | 39 | 0.81 (0.72, 0.91) | 0.0152 | 0.0471 |
| ｐ272 | 42 | 0.77 (0.65, 0.89) | 39 | 0.78 (0.65, 0.91) | 0.0215 | 0.0466 |
| ｐ13157 | 42 | 0.82 (0.29, 1.36) | 39 | 0.87 (0.32, 1.43) | 0.0840 | 0.0462 |
| ｐ17163 | 42 | 0.23 (0.15, 0.3) | 39 | 0.23 (0.15, 0.31) | 0.0433 | 0.0455 |
| ｐ14334 | 42 | 0.64 (0.24, 1.05) | 39 | 0.68 (0.26, 1.11) | 0.0800 | 0.0450 |
| ｐ15974 | 42 | 0.36 (0.21, 0.51) | 39 | 0.37 (0.22, 0.52) | 0.0516 | 0.0446 |
| ｐ11116 | 42 | 0.87 (0.73, 1) | 39 | 0.86 (0.72, 1) | -0.0200 | 0.0443 |
| ｐ15109 | 42 | 0.16 (0.08, 0.25) | 39 | 0.17 (0.09, 0.26) | 0.0615 | 0.0442 |
| ｐ260 | 42 | 1.01 (0.73, 1.28) | 39 | 1.03 (0.75, 1.32) | 0.0339 | 0.0436 |
| ｐ6023 | 42 | 0.24 (0.17, 0.31) | 39 | 0.25 (0.18, 0.32) | 0.0344 | 0.0434 |
| ｐ22932 | 42 | 0.64 (0.42, 0.85) | 39 | 0.62 (0.39, 0.84) | -0.0431 | 0.0434 |
| ｐ14451 | 42 | 0.63 (0.33, 0.94) | 39 | 0.61 (0.29, 0.92) | -0.0609 | 0.0431 |
| ｐ22090 | 42 | 0.02 (-0.01, 0.04) | 39 | 0.02 (-0.01, 0.05) | 0.2071 | 0.0431 |
| ｐ226 | 42 | 0.87 (0.48, 1.26) | 39 | 0.83 (0.43, 1.24) | -0.0566 | 0.0429 |
| ｐ6344 | 42 | 0.34 (0.27, 0.42) | 39 | 0.35 (0.27, 0.43) | 0.0268 | 0.0429 |
| ｐ19875 | 42 | 0.2 (0.12, 0.28) | 39 | 0.2 (0.12, 0.28) | 0.0465 | 0.0418 |
| ｐ25 | 42 | 0.5 (0.42, 0.57) | 39 | 0.49 (0.41, 0.57) | -0.0180 | 0.0406 |
| ｐ10864 | 42 | 0.16 (0.07, 0.24) | 39 | 0.17 (0.08, 0.25) | 0.0605 | 0.0405 |
| ｐ22088 | 42 | 0.24 (0.18, 0.31) | 39 | 0.24 (0.17, 0.3) | -0.0312 | 0.0404 |
| ｐ15325 | 42 | 0.2 (0.05, 0.35) | 39 | 0.19 (0.03, 0.35) | -0.0901 | 0.0404 |
| ｐ15402 | 42 | 0.79 (0.73, 0.85) | 39 | 0.79 (0.73, 0.85) | -0.0086 | 0.0402 |
| ｐ18829 | 42 | 0.1 (0.05, 0.14) | 39 | 0.1 (0.05, 0.15) | 0.0515 | 0.0379 |
| ｐ14619 | 42 | 0.54 (0.21, 0.88) | 39 | 0.57 (0.22, 0.91) | 0.0639 | 0.0366 |
| ｐ20155 | 42 | 0.42 (0.33, 0.51) | 39 | 0.43 (0.34, 0.52) | 0.0220 | 0.0363 |
| ｐ16569 | 42 | 0.34 (0.18, 0.5) | 39 | 0.35 (0.19, 0.52) | 0.0483 | 0.0360 |
| ｐ18216 | 42 | 0.34 (0.18, 0.5) | 39 | 0.35 (0.19, 0.52) | 0.0483 | 0.0360 |
| ｐ274 | 42 | 0.62 (0.47, 0.77) | 39 | 0.6 (0.45, 0.76) | -0.0257 | 0.0359 |
| ｐ19873 | 42 | 0.74 (0.5, 0.97) | 39 | 0.75 (0.51, 0.99) | 0.0318 | 0.0348 |
| ｐ15684 | 42 | 0.19 (0.02, 0.35) | 39 | 0.17 (0.01, 0.34) | -0.0891 | 0.0341 |
| ｐ15308 | 42 | 0.38 (0.29, 0.47) | 39 | 0.39 (0.29, 0.48) | 0.0240 | 0.0335 |
| ｐ13156 | 42 | 0.57 (0.53, 0.62) | 39 | 0.57 (0.52, 0.62) | -0.0079 | 0.0319 |
| ｐ22057 | 42 | 0.33 (0.23, 0.44) | 39 | 0.33 (0.22, 0.44) | -0.0290 | 0.0315 |
| ｐ18540 | 42 | 0.29 (0.26, 0.32) | 39 | 0.28 (0.25, 0.32) | -0.0095 | 0.0315 |
| ｐ14177 | 42 | 0.51 (0.42, 0.6) | 39 | 0.52 (0.43, 0.61) | 0.0155 | 0.0310 |
| ｐ10013 | 42 | 0.44 (0.35, 0.54) | 39 | 0.45 (0.35, 0.55) | 0.0188 | 0.0304 |
| ｐ8818 | 42 | 0.28 (0.19, 0.37) | 39 | 0.27 (0.18, 0.37) | -0.0297 | 0.0302 |
| ｐ21651 | 42 | 0.52 (0.46, 0.58) | 39 | 0.52 (0.47, 0.58) | 0.0093 | 0.0299 |
| ｐ10012 | 42 | 0.71 (0.66, 0.76) | 39 | 0.71 (0.66, 0.75) | -0.0055 | 0.0284 |
| ｐ22084 | 42 | 0.17 (0.13, 0.21) | 39 | 0.17 (0.13, 0.21) | -0.0185 | 0.0282 |
| ｐ2577 | 42 | 0.35 (0.18, 0.52) | 39 | 0.34 (0.16, 0.51) | -0.0405 | 0.0276 |
| ｐ11246 | 42 | 0.44 (0.3, 0.59) | 39 | 0.43 (0.29, 0.58) | -0.0263 | 0.0273 |
| ｐ22086 | 42 | 0.73 (0.47, 0.99) | 39 | 0.74 (0.47, 1.01) | 0.0275 | 0.0265 |
| ｐ17166 | 42 | 0.55 (0.54, 0.56) | 39 | 0.55 (0.54, 0.56) | -0.0012 | 0.0263 |
| ｐ15027 | 42 | 0.37 (0.25, 0.5) | 39 | 0.37 (0.23, 0.5) | -0.0258 | 0.0254 |
| ｐ18340 | 42 | 0.36 (0.29, 0.43) | 39 | 0.36 (0.3, 0.43) | 0.0132 | 0.0245 |
| ｐ14969 | 42 | 0.25 (0.19, 0.3) | 39 | 0.25 (0.19, 0.3) | 0.0152 | 0.0240 |
| ｐ17849 | 42 | 0.63 (0.59, 0.67) | 39 | 0.63 (0.58, 0.67) | -0.0043 | 0.0237 |
| ｐ21458 | 41 | 0.57 (0.13, 1.02) | 39 | 0.55 (0.1, 1.01) | -0.0523 | 0.0228 |
| ｐ12070 | 42 | 0.18 (0.07, 0.29) | 39 | 0.18 (0.07, 0.3) | 0.0398 | 0.0215 |
| ｐ17360 | 42 | 0.69 (0.64, 0.74) | 39 | 0.69 (0.64, 0.74) | 0.0046 | 0.0214 |
| ｐ17930 | 42 | 0.18 (0.11, 0.25) | 39 | 0.17 (0.1, 0.25) | -0.0254 | 0.0213 |
| ｐ18134 | 42 | 0.32 (0.18, 0.46) | 39 | 0.31 (0.17, 0.45) | -0.0266 | 0.0205 |
| ｐ411 | 42 | 0.99 (0.83, 1.16) | 39 | 1 (0.83, 1.17) | 0.0097 | 0.0197 |
| ｐ15617 | 42 | 0.54 (0.5, 0.59) | 39 | 0.54 (0.49, 0.59) | -0.0050 | 0.0197 |
| ｐ11869 | 42 | 0.44 (0.36, 0.52) | 39 | 0.45 (0.36, 0.53) | 0.0106 | 0.0196 |
| ｐ16367 | 42 | 0.28 (0.14, 0.43) | 39 | 0.28 (0.13, 0.43) | -0.0280 | 0.0184 |
| ｐ22449 | 42 | 0.12 (0.06, 0.17) | 39 | 0.12 (0.06, 0.17) | -0.0246 | 0.0181 |
| ｐ17898 | 42 | 0.32 (0.22, 0.42) | 39 | 0.32 (0.22, 0.43) | 0.0160 | 0.0171 |
| ｐ13403 | 42 | 0.09 (0.05, 0.14) | 39 | 0.1 (0.05, 0.15) | 0.0258 | 0.0170 |
| ｐ14953 | 42 | 0.07 (0.04, 0.1) | 39 | 0.07 (0.04, 0.1) | 0.0190 | 0.0159 |
| ｐ11688 | 42 | 0.35 (0.26, 0.44) | 39 | 0.35 (0.26, 0.44) | 0.0116 | 0.0152 |
| ｐ22952 | 42 | 0.36 (0.3, 0.41) | 39 | 0.36 (0.3, 0.41) | -0.0071 | 0.0147 |
| ｐ15065 | 42 | 0.54 (0.34, 0.74) | 39 | 0.54 (0.32, 0.75) | -0.0157 | 0.0139 |
| ｐ16630 | 42 | 0.04 (-0.01, 0.08) | 39 | 0.04 (-0.01, 0.08) | 0.0513 | 0.0139 |
| ｐ20168 | 42 | 0.38 (0.23, 0.53) | 39 | 0.37 (0.22, 0.53) | -0.0160 | 0.0136 |
| ｐ15425 | 42 | 0.14 (0.09, 0.2) | 39 | 0.14 (0.09, 0.2) | -0.0143 | 0.0135 |
| ｐ17625 | 42 | 0.46 (0.31, 0.61) | 39 | 0.46 (0.3, 0.61) | -0.0129 | 0.0133 |
| ｐ11489 | 42 | 0.53 (0.49, 0.56) | 39 | 0.53 (0.49, 0.57) | 0.0026 | 0.0125 |
| ｐ21023 | 42 | 0.38 (0.33, 0.43) | 39 | 0.38 (0.33, 0.43) | -0.0041 | 0.0108 |
| ｐ18491 | 42 | 0.5 (0.35, 0.65) | 39 | 0.49 (0.34, 0.65) | -0.0088 | 0.0099 |
| ｐ15293 | 42 | 0.43 (0.36, 0.5) | 39 | 0.43 (0.35, 0.5) | -0.0049 | 0.0097 |
| ｐ15579 | 42 | 0.51 (0.44, 0.59) | 39 | 0.52 (0.44, 0.59) | 0.0039 | 0.0091 |
| ｐ20942 | 42 | 0.59 (0.52, 0.67) | 39 | 0.59 (0.52, 0.67) | 0.0033 | 0.0088 |
| ｐ976 | 42 | 0.52 (0.43, 0.61) | 39 | 0.52 (0.43, 0.62) | 0.0044 | 0.0086 |
| ｐ15947 | 42 | 0.32 (0.22, 0.43) | 39 | 0.32 (0.21, 0.43) | -0.0080 | 0.0082 |
| ｐ20585 | 42 | 0.57 (0.54, 0.59) | 39 | 0.57 (0.54, 0.59) | -0.0010 | 0.0077 |
| ｐ14932 | 42 | 0.45 (0.28, 0.61) | 39 | 0.45 (0.28, 0.62) | 0.0082 | 0.0075 |
| ｐ15998 | 42 | 0.05 (-0.01, 0.1) | 39 | 0.05 (-0.02, 0.11) | 0.0284 | 0.0074 |
| ｐ10813 | 42 | 0.34 (0.31, 0.38) | 39 | 0.34 (0.31, 0.38) | -0.0022 | 0.0070 |
| ｐ14991 | 42 | 0.3 (0.21, 0.38) | 39 | 0.3 (0.21, 0.39) | 0.0057 | 0.0067 |
| ｐ14966 | 42 | 0.2 (0.15, 0.26) | 39 | 0.21 (0.15, 0.27) | 0.0054 | 0.0064 |
| ｐ19833 | 42 | 0.57 (0.43, 0.72) | 39 | 0.58 (0.42, 0.73) | 0.0046 | 0.0060 |
| ｐ20821 | 42 | 0.2 (0, 0.41) | 39 | 0.21 (-0.01, 0.42) | 0.0183 | 0.0060 |
| ｐ13406 | 42 | 0.39 (0.29, 0.49) | 39 | 0.39 (0.28, 0.5) | -0.0048 | 0.0060 |
| ｐ22066 | 42 | 0.03 (-0.02, 0.08) | 39 | 0.03 (-0.02, 0.08) | -0.0309 | 0.0060 |
| ｐ11407 | 42 | 0.41 (0.39, 0.44) | 39 | 0.41 (0.38, 0.44) | -0.0012 | 0.0058 |
| ｐ20714 | 41 | 0.22 (0, 0.44) | 39 | 0.22 (-0.01, 0.44) | 0.0172 | 0.0057 |
| ｐ16691 | 42 | 0.75 (0.35, 1.15) | 39 | 0.75 (0.33, 1.17) | 0.0092 | 0.0057 |
| ｐ18687 | 42 | 0.44 (0.32, 0.56) | 39 | 0.44 (0.32, 0.57) | 0.0047 | 0.0057 |
| ｐ16062 | 42 | 0.23 (0.17, 0.28) | 39 | 0.23 (0.17, 0.28) | -0.0040 | 0.0055 |
| ｐ10976 | 42 | 0.38 (0.23, 0.52) | 39 | 0.38 (0.23, 0.53) | 0.0061 | 0.0053 |
| ｐ17784 | 42 | 0.62 (0.56, 0.68) | 39 | 0.62 (0.56, 0.68) | -0.0014 | 0.0047 |
| ｐ16650 | 42 | 0.28 (0.2, 0.36) | 39 | 0.28 (0.2, 0.36) | 0.0038 | 0.0046 |
| ｐ2164 | 42 | 0.73 (0.73, 0.74) | 39 | 0.73 (0.73, 0.74) | -0.0001 | 0.0038 |
| ｐ11860 | 42 | 0.18 (0.11, 0.25) | 39 | 0.18 (0.1, 0.25) | -0.0040 | 0.0033 |
| ｐ22301 | 42 | 0.32 (0.28, 0.36) | 39 | 0.32 (0.28, 0.36) | -0.0012 | 0.0031 |
| ｐ15132 | 42 | 0.32 (0.28, 0.36) | 39 | 0.32 (0.28, 0.36) | -0.0012 | 0.0031 |
| ｐ15864 | 42 | 0.25 (0.18, 0.32) | 39 | 0.25 (0.18, 0.32) | -0.0026 | 0.0031 |
| ｐ15363 | 42 | 0.11 (0.05, 0.17) | 39 | 0.11 (0.05, 0.17) | 0.0047 | 0.0030 |
| ｐ15199 | 42 | 0.54 (0.34, 0.75) | 39 | 0.54 (0.33, 0.75) | -0.0033 | 0.0030 |
| ｐ8170 | 42 | 0.31 (0.02, 0.59) | 39 | 0.3 (0, 0.6) | -0.0076 | 0.0027 |
| ｐ10891 | 42 | 0.17 (0.08, 0.26) | 39 | 0.17 (0.08, 0.26) | -0.0036 | 0.0024 |
| ｐ22558 | 42 | 0.57 (0.23, 0.9) | 39 | 0.57 (0.22, 0.91) | 0.0024 | 0.0013 |
| ｐ17995 | 42 | 0.84 (0.5, 1.18) | 39 | 0.84 (0.49, 1.2) | 0.0012 | 0.0010 |
| ｐ22656 | 42 | 0.46 (0.37, 0.55) | 39 | 0.46 (0.37, 0.56) | 0.0004 | 0.0007 |
| ｐ10552 | 42 | 0.33 (0.22, 0.45) | 39 | 0.33 (0.21, 0.45) | -0.0002 | 0.0002 |
| ｐ6067 | 1 | 0.75 (0, 0) | 1 | 30.44 (0, 0) | 5.3379 | NA |
| ｐ3779 | 39 | 0.02 (-0.02, 0.06) | 42 | 0.66 (-0.54, 1.85) | 5.1064 | NA |
| ｐ3640 | 39 | 0.02 (-0.05, 0.09) | 42 | 0.54 (0.47, 0.6) | 5.0729 | NA |
| ｐ5221 | 39 | 0.04 (-0.33, 0.4) | 1 | 0.95 (0, 0) | 4.6515 | NA |
| ｐ8065 | 39 | 0.07 (0.03, 0.1) | 42 | 1.58 (0.86, 2.29) | 4.5852 | NA |
| ｐ9151 | 1 | 0.07 (0, 0) | 1 | 1.4 (0, 0) | 4.2737 | NA |
| ｐ7402 | 39 | 0.06 (0, 0.12) | 42 | 1.11 (0.93, 1.29) | 4.2150 | NA |
| ｐ7799 | 39 | 0.93 (0.65, 1.2) | 1 | 13.26 (0, 0) | 3.8392 | NA |
| ｐ1712 | 39 | 0.13 (0.08, 0.17) | 1 | 1.77 (0, 0) | 3.8149 | NA |
| ｐ9222 | 39 | 0.1 (0.07, 0.13) | 42 | 1.33 (1.17, 1.48) | 3.6784 | NA |
| ｐ5103 | 39 | 0.06 (0.01, 0.12) | 42 | 0.81 (0.62, 1) | 3.6742 | NA |
| ｐ657 | 39 | 0.07 (-0.05, 0.2) | 42 | 0.9 (0.76, 1.04) | 3.6330 | NA |
| ｐ1144 | 39 | 0.15 (0.05, 0.25) | 42 | 1.85 (1.78, 1.93) | 3.6274 | NA |
| ｐ2229 | 39 | 0.03 (-0.01, 0.08) | 42 | 0.42 (0.29, 0.55) | 3.6136 | NA |
| ｐ6230 | 39 | 0.1 (-0.07, 0.27) | 1 | 1.17 (0, 0) | 3.5581 | NA |
| ｐ506 | 39 | 0.09 (0.03, 0.16) | 42 | 1.01 (1.01, 1.02) | 3.4531 | NA |
| ｐ914 | 39 | 0.77 (0.74, 0.8) | 1 | 8.31 (0, 0) | 3.4297 | NA |
| ｐ5982 | 1 | 0.16 (0, 0) | 1 | 1.47 (0, 0) | 3.1752 | NA |
| ｐ1137 | 39 | 0.25 (0.21, 0.29) | 42 | 2.2 (-0.21, 4.61) | 3.1220 | NA |
| ｐ2341 | 39 | 0.04 (-0.07, 0.14) | 42 | 0.31 (0.25, 0.36) | 3.0862 | NA |
| ｐ1286 | 39 | 0.45 (0.4, 0.5) | 1 | 3.63 (0, 0) | 3.0176 | NA |
| ｐ2617 | 39 | 0.1 (0.02, 0.18) | 42 | 0.76 (0.73, 0.79) | 2.9509 | NA |
| ｐ6355 | 39 | 0.15 (0.12, 0.18) | 1 | 1.14 (0, 0) | 2.9283 | NA |
| ｐ5532 | 39 | 0.03 (-0.03, 0.09) | 42 | 0.21 (0.12, 0.31) | 2.8354 | NA |
| ｐ908 | 39 | 0.07 (0, 0.14) | 42 | 0.5 (0.28, 0.71) | 2.8276 | NA |
| ｐ4507 | 39 | 0.11 (0.04, 0.18) | 1 | 0.76 (0, 0) | 2.8104 | NA |
| ｐ8747 | 39 | 0.06 (-0.02, 0.14) | 42 | 0.42 (0.25, 0.59) | 2.7963 | NA |
| ｐ6006 | 1 | 0.4 (0, 0) | 1 | 2.71 (0, 0) | 2.7483 | NA |
| ｐ1848 | 39 | 0.12 (0.08, 0.16) | 42 | 0.77 (0.38, 1.16) | 2.6908 | NA |
| ｐ7978 | 39 | 0.14 (0.11, 0.18) | 42 | 0.92 (0.53, 1.32) | 2.6799 | NA |
| ｐ8139 | 39 | 0.18 (0.12, 0.24) | 1 | 1.13 (0, 0) | 2.6762 | NA |
| ｐ2871 | 39 | 0.1 (0.03, 0.18) | 42 | 0.62 (0.31, 0.92) | 2.5956 | NA |
| ｐ7346 | 39 | 0.21 (0.1, 0.32) | 1 | 1.23 (0, 0) | 2.5598 | NA |
| ｐ1075 | 39 | 0.35 (0.27, 0.44) | 42 | 2.08 (1.7, 2.47) | 2.5556 | NA |
| ｐ4370 | 1 | 0.66 (0, 0) | 1 | 3.87 (0, 0) | 2.5539 | NA |
| ｐ478 | 39 | 0.44 (0.28, 0.6) | 42 | 2.59 (0.54, 4.64) | 2.5503 | NA |
| ｐ5639 | 39 | 0.12 (0.06, 0.18) | 42 | 0.66 (0.61, 0.72) | 2.5103 | NA |
| ｐ8809 | 39 | 0.37 (0.28, 0.46) | 1 | 2.04 (0, 0) | 2.4575 | NA |
| ｐ6205 | 39 | 0.28 (0.24, 0.32) | 1 | 1.5 (0, 0) | 2.4249 | NA |
| ｐ635 | 39 | 0.13 (0.01, 0.24) | 42 | 0.66 (0.48, 0.84) | 2.3987 | NA |
| ｐ7696 | 39 | 0.14 (-0.37, 0.66) | 1 | 0.74 (0, 0) | 2.3440 | NA |
| ｐ1280 | 39 | 0.13 (0.09, 0.16) | 42 | 0.63 (0.57, 0.7) | 2.3268 | NA |
| ｐ7778 | 39 | 0.13 (0.02, 0.24) | 42 | 0.66 (0.39, 0.92) | 2.3184 | NA |
| ｐ557 | 39 | 0.24 (0.14, 0.33) | 42 | 1.15 (0.92, 1.37) | 2.2883 | NA |
| ｐ4573 | 39 | 0.13 (0.05, 0.2) | 42 | 0.61 (0.5, 0.71) | 2.2671 | NA |
| ｐ4070 | 39 | 0.14 (0.12, 0.16) | 42 | 0.66 (0.5, 0.82) | 2.2629 | NA |
| ｐ5078 | 39 | 0.22 (0.18, 0.26) | 42 | 1.04 (-1.05, 3.13) | 2.2518 | NA |
| ｐ8545 | 39 | 0.2 (0.09, 0.32) | 42 | 0.96 (0.94, 0.97) | 2.2263 | NA |
| ｐ5803 | 39 | 0.09 (0.01, 0.17) | 41 | 0.42 (0.36, 0.48) | 2.2125 | NA |
| ｐ454 | 39 | 0.18 (0.12, 0.23) | 42 | 0.78 (0.7, 0.87) | 2.1541 | NA |
| ｐ8202 | 39 | 0.13 (-0.11, 0.37) | 1 | 0.57 (0, 0) | 2.1404 | NA |
| ｐ7643 | 39 | 0.19 (0.06, 0.31) | 42 | 0.81 (0.79, 0.83) | 2.1309 | NA |
| ｐ2959 | 39 | 0.26 (0.22, 0.3) | 1 | 1.09 (0, 0) | 2.0749 | NA |
| ｐ8709 | 39 | 0.54 (0.41, 0.67) | 42 | 2.24 (2.24, 2.25) | 2.0605 | NA |
| ｐ4234 | 1 | 1 (0, 0) | 42 | 4.11 (1.55, 6.66) | 2.0386 | NA |
| ｐ7158 | 39 | 0.59 (0.46, 0.73) | 1 | 2.42 (0, 0) | 2.0337 | NA |
| ｐ1262 | 39 | 0.32 (0.29, 0.36) | 42 | 1.33 (0.31, 2.34) | 2.0315 | NA |
| ｐ7432 | 39 | 0.42 (0.39, 0.46) | 42 | 1.65 (1.59, 1.72) | 1.9618 | NA |
| ｐ5664 | 39 | 0.15 (0.06, 0.23) | 42 | 0.56 (0.44, 0.69) | 1.9543 | NA |
| ｐ4400 | 39 | 0.17 (0.07, 0.28) | 42 | 0.67 (0.53, 0.8) | 1.9425 | NA |
| ｐ1113 | 39 | 0.19 (0.17, 0.22) | 42 | 0.74 (0.27, 1.22) | 1.9422 | NA |
| ｐ5077 | 39 | 0.07 (0.02, 0.12) | 42 | 0.26 (0.22, 0.3) | 1.9265 | NA |
| ｐ6685 | 39 | 0.12 (0.08, 0.16) | 42 | 0.46 (0.43, 0.48) | 1.9196 | NA |
| ｐ538 | 39 | 0.16 (0.05, 0.28) | 42 | 0.61 (0.53, 0.7) | 1.9086 | NA |
| ｐ3482 | 39 | 0.1 (-0.07, 0.27) | 42 | 0.39 (0.23, 0.54) | 1.9071 | NA |
| ｐ2512 | 39 | 0.15 (0.08, 0.22) | 42 | 0.56 (0.53, 0.59) | 1.9002 | NA |
| ｐ6794 | 39 | 0.19 (0.14, 0.25) | 42 | 0.71 (0.69, 0.72) | 1.8744 | NA |
| ｐ4533 | 39 | 0.1 (0.02, 0.17) | 42 | 0.35 (0.25, 0.45) | 1.8613 | NA |
| ｐ4537 | 39 | 0.15 (0.04, 0.25) | 42 | 0.52 (0.25, 0.79) | 1.8472 | NA |
| ｐ9060 | 39 | 0.34 (0.14, 0.54) | 42 | 1.17 (1.15, 1.18) | 1.7894 | NA |
| ｐ6223 | 39 | 0.1 (0.05, 0.16) | 42 | 0.35 (0.29, 0.41) | 1.7536 | NA |
| ｐ9408 | 1 | 0.55 (0, 0) | 1 | 1.83 (0, 0) | 1.7384 | NA |
| ｐ5425 | 39 | 0.31 (0.3, 0.31) | 1 | 1 (0, 0) | 1.7102 | NA |
| ｐ9948 | 39 | 0.2 (0.17, 0.24) | 42 | 0.66 (0.6, 0.73) | 1.6920 | NA |
| ｐ2679 | 39 | 0.67 (0.65, 0.69) | 1 | 2.16 (0, 0) | 1.6880 | NA |
| ｐ9451 | 39 | 0.34 (0.21, 0.47) | 1 | 1.08 (0, 0) | 1.6761 | NA |
| ｐ4678 | 39 | 0.37 (0.04, 0.71) | 1 | 1.15 (0, 0) | 1.6368 | NA |
| ｐ5723 | 39 | 0.18 (-0.05, 0.41) | 42 | 0.55 (0.48, 0.62) | 1.6057 | NA |
| ｐ6182 | 39 | 0.24 (0.12, 0.35) | 42 | 0.72 (0.67, 0.77) | 1.6053 | NA |
| ｐ5965 | 1 | 0.47 (0, 0) | 1 | 1.43 (0, 0) | 1.6001 | NA |
| ｐ290 | 39 | 0.3 (0.21, 0.39) | 1 | 0.89 (0, 0) | 1.5959 | NA |
| ｐ3240 | 39 | 0.26 (0.17, 0.34) | 42 | 0.77 (0.71, 0.84) | 1.5912 | NA |
| ｐ9109 | 39 | 0.75 (0.53, 0.98) | 42 | 2.24 (2.18, 2.3) | 1.5680 | NA |
| ｐ3910 | 39 | 0.21 (-0.02, 0.43) | 42 | 0.61 (0.55, 0.67) | 1.5611 | NA |
| ｐ9898 | 39 | 0.2 (-0.32, 0.72) | 42 | 0.59 (0.51, 0.67) | 1.5529 | NA |
| ｐ6573 | 39 | 0.16 (0.03, 0.29) | 42 | 0.46 (0.42, 0.49) | 1.5501 | NA |
| ｐ8946 | 39 | 0.33 (0.15, 0.52) | 1 | 0.97 (0, 0) | 1.5415 | NA |
| ｐ492 | 39 | 0.41 (0.22, 0.6) | 42 | 1.19 (0.71, 1.66) | 1.5364 | NA |
| ｐ8764 | 39 | 0.28 (0.1, 0.46) | 1 | 0.79 (0, 0) | 1.4725 | NA |
| ｐ4495 | 39 | 0.31 (0.19, 0.44) | 42 | 0.87 (0.8, 0.94) | 1.4691 | NA |
| ｐ3155 | 39 | 0.3 (0.19, 0.42) | 42 | 0.84 (0.61, 1.08) | 1.4666 | NA |
| ｐ4581 | 39 | 0.27 (0.16, 0.38) | 1 | 0.75 (0, 0) | 1.4513 | NA |
| ｐ3812 | 39 | 0.18 (0.1, 0.27) | 42 | 0.5 (0.38, 0.61) | 1.4500 | NA |
| ｐ2937 | 39 | 0.21 (0.08, 0.33) | 42 | 0.56 (0.51, 0.61) | 1.4358 | NA |
| ｐ6060 | 39 | 0.4 (0.28, 0.52) | 1 | 1.07 (0, 0) | 1.4144 | NA |
| ｐ1665 | 1 | 0.42 (0, 0) | 1 | 1.13 (0, 0) | 1.4133 | NA |
| ｐ5700 | 39 | 0.14 (0.08, 0.2) | 42 | 0.37 (0.22, 0.51) | 1.3990 | NA |
| ｐ2346 | 39 | 0.21 (0.16, 0.27) | 42 | 0.56 (0.51, 0.6) | 1.3872 | NA |
| ｐ7152 | 39 | 0.17 (0.11, 0.24) | 42 | 0.45 (0.33, 0.58) | 1.3828 | NA |
| ｐ5084 | 39 | 0.31 (0.24, 0.39) | 42 | 0.8 (0.55, 1.04) | 1.3626 | NA |
| ｐ8700 | 39 | 0.25 (0.22, 0.28) | 42 | 0.64 (0.51, 0.76) | 1.3435 | NA |
| ｐ1525 | 39 | 0.82 (0.77, 0.88) | 1 | 2.06 (0, 0) | 1.3225 | NA |
| ｐ1434 | 39 | 0.4 (0.34, 0.45) | 42 | 0.99 (0.83, 1.15) | 1.3152 | NA |
| ｐ1521 | 39 | 0.29 (0.19, 0.39) | 42 | 0.72 (0.67, 0.78) | 1.3105 | NA |
| ｐ5149 | 33 | 0.39 (0.35, 0.44) | 42 | 0.97 (0.58, 1.36) | 1.3038 | NA |
| ｐ1000 | 39 | 0.49 (0.45, 0.52) | 42 | 1.17 (-1.1, 3.45) | 1.2729 | NA |
| ｐ5890 | 39 | 0.52 (0.34, 0.7) | 42 | 1.26 (0.28, 2.24) | 1.2717 | NA |
| ｐ7216 | 1 | 0.39 (0, 0) | 42 | 0.93 (0.93, 0.94) | 1.2495 | NA |
| ｐ1990 | 39 | 0.19 (0.06, 0.32) | 42 | 0.44 (0.41, 0.48) | 1.2367 | NA |
| ｐ8347 | 39 | 0.16 (0.1, 0.21) | 42 | 0.36 (0.3, 0.43) | 1.2333 | NA |
| ｐ6132 | 39 | 0.83 (0.57, 1.09) | 42 | 1.94 (1.52, 2.37) | 1.2327 | NA |
| ｐ5486 | 39 | 0.3 (0.16, 0.45) | 42 | 0.71 (0.7, 0.72) | 1.2305 | NA |
| ｐ5136 | 39 | 0.55 (0.18, 0.92) | 1 | 1.25 (0, 0) | 1.2003 | NA |
| ｐ2749 | 39 | 0.61 (0.18, 1.05) | 42 | 1.4 (1.4, 1.41) | 1.1950 | NA |
| ｐ8376 | 39 | 0.82 (0.74, 0.89) | 1 | 1.87 (0, 0) | 1.1894 | NA |
| ｐ8559 | 39 | 0.93 (0.92, 0.95) | 1 | 2.12 (0, 0) | 1.1842 | NA |
| ｐ5548 | 39 | 0.22 (0.14, 0.3) | 42 | 0.5 (0.41, 0.59) | 1.1742 | NA |
| ｐ9653 | 39 | 0.29 (0.27, 0.31) | 42 | 0.66 (0.64, 0.67) | 1.1566 | NA |
| ｐ4412 | 39 | 0.36 (0.3, 0.42) | 42 | 0.79 (0.57, 1.02) | 1.1513 | NA |
| ｐ6224 | 39 | 0.24 (0.18, 0.3) | 42 | 0.53 (0.37, 0.69) | 1.1491 | NA |
| ｐ3284 | 39 | 0.41 (0.26, 0.55) | 42 | 0.89 (0.61, 1.16) | 1.1302 | NA |
| ｐ1157 | 39 | 0.61 (0.48, 0.74) | 1 | 1.31 (0, 0) | 1.1095 | NA |
| ｐ2153 | 39 | 0.62 (0.48, 0.75) | 1 | 1.31 (0, 0) | 1.0950 | NA |
| ｐ599 | 39 | 0.38 (0.27, 0.49) | 1 | 0.8 (0, 0) | 1.0780 | NA |
| ｐ5496 | 39 | 0.3 (0.2, 0.4) | 42 | 0.64 (0.63, 0.65) | 1.0776 | NA |
| ｐ682 | 39 | 0.43 (0.38, 0.49) | 1 | 0.91 (0, 0) | 1.0769 | NA |
| ｐ2878 | 39 | 0.47 (0.15, 0.79) | 1 | 0.99 (0, 0) | 1.0755 | NA |
| ｐ665 | 39 | 0.34 (0.2, 0.47) | 42 | 0.71 (0.69, 0.72) | 1.0736 | NA |
| ｐ588 | 39 | 0.27 (0.21, 0.34) | 42 | 0.57 (0.46, 0.67) | 1.0416 | NA |
| ｐ1134 | 39 | 0.42 (-0.08, 0.91) | 42 | 0.86 (0.69, 1.02) | 1.0372 | NA |
| ｐ4417 | 39 | 0.4 (0.31, 0.49) | 1 | 0.82 (0, 0) | 1.0320 | NA |
| ｐ3360 | 39 | 0.43 (0.36, 0.49) | 42 | 0.86 (0.83, 0.9) | 1.0146 | NA |
| ｐ5963 | 1 | 0.43 (0, 0) | 1 | 0.87 (0, 0) | 1.0105 | NA |
| ｐ1907 | 1 | 0.83 (0, 0) | 1 | 1.67 (0, 0) | 1.0062 | NA |
| ｐ2671 | 39 | 0.35 (0.18, 0.51) | 42 | 0.69 (0.67, 0.71) | 0.9975 | NA |
| ｐ2352 | 39 | 0.62 (0.55, 0.69) | 42 | 1.23 (1.03, 1.44) | 0.9926 | NA |
| ｐ9360 | 39 | 0.15 (0.13, 0.17) | 42 | 0.29 (0.27, 0.32) | 0.9912 | NA |
| ｐ3260 | 39 | 0.47 (0.37, 0.57) | 42 | 0.93 (0.89, 0.98) | 0.9898 | NA |
| ｐ3458 | 39 | 0.38 (0.33, 0.43) | 42 | 0.76 (0.55, 0.97) | 0.9866 | NA |
| ｐ2073 | 39 | 0.41 (0.29, 0.54) | 42 | 0.81 (0.37, 1.26) | 0.9782 | NA |
| ｐ5464 | 1 | 0.27 (0, 0) | 42 | 0.53 (0.32, 0.74) | 0.9675 | NA |
| ｐ2136 | 39 | 0.3 (0.23, 0.37) | 42 | 0.58 (0.45, 0.71) | 0.9601 | NA |
| ｐ8902 | 39 | 0.34 (0.18, 0.5) | 1 | 0.66 (0, 0) | 0.9548 | NA |
| ｐ9610 | 39 | 0.54 (0.36, 0.72) | 42 | 1.03 (0.97, 1.09) | 0.9195 | NA |
| ｐ2357 | 39 | 0.81 (0.6, 1.02) | 1 | 1.53 (0, 0) | 0.9125 | NA |
| ｐ6088 | 39 | 0.51 (0.32, 0.7) | 1 | 0.95 (0, 0) | 0.9039 | NA |
| ｐ5121 | 39 | 0.37 (0.1, 0.64) | 42 | 0.68 (0.33, 1.04) | 0.8890 | NA |
| ｐ7991 | 39 | 0.66 (0.32, 0.99) | 1 | 1.21 (0, 0) | 0.8862 | NA |
| ｐ1320 | 39 | 0.44 (0.31, 0.56) | 42 | 0.81 (0.43, 1.18) | 0.8858 | NA |
| ｐ1050 | 39 | 1.81 (1.2, 2.41) | 41 | 3.32 (-0.53, 7.17) | 0.8791 | NA |
| ｐ3389 | 39 | 0.17 (0.14, 0.2) | 42 | 0.31 (0.19, 0.43) | 0.8777 | NA |
| ｐ3370 | 39 | 0.54 (0.44, 0.63) | 42 | 0.98 (0.96, 1.01) | 0.8753 | NA |
| ｐ3396 | 39 | 0.78 (0.66, 0.89) | 42 | 1.4 (1.36, 1.44) | 0.8537 | NA |
| ｐ4459 | 39 | 0.5 (0.49, 0.51) | 42 | 0.88 (-0.01, 1.78) | 0.8334 | NA |
| ｐ526 | 39 | 0.23 (0.13, 0.34) | 42 | 0.42 (0.25, 0.58) | 0.8306 | NA |
| ｐ1381 | 39 | 0.51 (0.37, 0.66) | 42 | 0.91 (0.77, 1.05) | 0.8274 | NA |
| ｐ747 | 39 | 0.24 (0.16, 0.33) | 42 | 0.43 (0.27, 0.59) | 0.8179 | NA |
| ｐ7226 | 39 | 1.16 (1.14, 1.18) | 1 | 2.04 (0, 0) | 0.8155 | NA |
| ｐ3202 | 39 | 0.23 (0.14, 0.31) | 42 | 0.4 (0.35, 0.45) | 0.8144 | NA |
| ｐ1436 | 39 | 0.58 (0.45, 0.71) | 42 | 1.02 (0.92, 1.12) | 0.8091 | NA |
| ｐ7135 | 39 | 0.39 (0.2, 0.59) | 42 | 0.69 (0.49, 0.88) | 0.7999 | NA |
| ｐ5868 | 39 | 0.43 (0.32, 0.55) | 42 | 0.75 (0.54, 0.97) | 0.7987 | NA |
| ｐ7460 | 39 | 1.63 (1.56, 1.7) | 1 | 2.84 (0, 0) | 0.7981 | NA |
| ｐ3186 | 39 | 0.38 (0.33, 0.43) | 42 | 0.66 (0.62, 0.7) | 0.7979 | NA |
| ｐ3250 | 39 | 0.43 (0.39, 0.46) | 42 | 0.73 (0.64, 0.82) | 0.7851 | NA |
| ｐ488 | 39 | 0.3 (0.23, 0.37) | 42 | 0.52 (0.42, 0.61) | 0.7822 | NA |
| ｐ1154 | 39 | 0.22 (0.13, 0.31) | 42 | 0.37 (0.25, 0.5) | 0.7655 | NA |
| ｐ4444 | 1 | 0.55 (0, 0) | 1 | 0.93 (0, 0) | 0.7654 | NA |
| ｐ3218 | 39 | 0.47 (0.37, 0.56) | 42 | 0.8 (0.77, 0.82) | 0.7637 | NA |
| ｐ3345 | 39 | 0.47 (0.38, 0.56) | 42 | 0.79 (0.47, 1.11) | 0.7587 | NA |
| ｐ2282 | 39 | 0.68 (0.47, 0.89) | 1 | 1.14 (0, 0) | 0.7495 | NA |
| ｐ15455 | 42 | 0.51 (0.4, 0.61) | 39 | 0.85 (0.74, 0.96) | 0.7439 | NA |
| ｐ9259 | 39 | 0.59 (0.54, 0.63) | 1 | 0.97 (0, 0) | 0.7339 | NA |
| ｐ2082 | 39 | 0.55 (0.09, 1.01) | 42 | 0.91 (0.9, 0.93) | 0.7272 | NA |
| ｐ1811 | 39 | 0.34 (0.31, 0.37) | 42 | 0.56 (0.49, 0.62) | 0.7252 | NA |
| ｐ3273 | 39 | 0.44 (0.34, 0.54) | 42 | 0.73 (0.51, 0.95) | 0.7233 | NA |
| ｐ6675 | 39 | 0.35 (0.22, 0.48) | 42 | 0.58 (0.48, 0.68) | 0.7217 | NA |
| ｐ8532 | 39 | 0.2 (0.14, 0.26) | 42 | 0.33 (0.21, 0.44) | 0.7118 | NA |
| ｐ4624 | 39 | 0.36 (0.22, 0.49) | 42 | 0.58 (0.2, 0.97) | 0.7070 | NA |
| ｐ3933 | 39 | 0.55 (0.5, 0.61) | 1 | 0.9 (0, 0) | 0.7040 | NA |
| ｐ4498 | 39 | 0.76 (0.69, 0.84) | 42 | 1.22 (0.87, 1.57) | 0.6764 | NA |
| ｐ1055 | 39 | 0.52 (0.29, 0.75) | 42 | 0.83 (0.33, 1.33) | 0.6715 | NA |
| ｐ5064 | 39 | 0.36 (0.21, 0.5) | 42 | 0.57 (0.54, 0.6) | 0.6659 | NA |
| ｐ2472 | 39 | 0.57 (0.38, 0.77) | 42 | 0.91 (0.76, 1.06) | 0.6652 | NA |
| ｐ4716 | 39 | 0.51 (0.5, 0.52) | 1 | 0.8 (0, 0) | 0.6550 | NA |
| ｐ3268 | 39 | 0.54 (0.48, 0.61) | 42 | 0.85 (0.75, 0.95) | 0.6492 | NA |
| ｐ1088 | 39 | 0.52 (0.41, 0.62) | 42 | 0.81 (0.39, 1.22) | 0.6436 | NA |
| ｐ9465 | 39 | 0.71 (0.6, 0.81) | 42 | 1.1 (1.09, 1.1) | 0.6376 | NA |
| ｐ3227 | 39 | 0.26 (0.25, 0.27) | 41 | 0.4 (0.19, 0.61) | 0.6342 | NA |
| ｐ8355 | 39 | 0.43 (0.36, 0.49) | 42 | 0.66 (0.59, 0.73) | 0.6306 | NA |
| ｐ9674 | 39 | 0.31 (0.25, 0.37) | 42 | 0.47 (0.39, 0.55) | 0.6081 | NA |
| ｐ9897 | 39 | 0.4 (0.26, 0.54) | 42 | 0.61 (0.11, 1.11) | 0.6034 | NA |
| ｐ2900 | 39 | 0.31 (0.25, 0.37) | 42 | 0.47 (0.27, 0.67) | 0.6024 | NA |
| ｐ3307 | 39 | 0.59 (0.55, 0.63) | 42 | 0.89 (0.87, 0.92) | 0.5967 | NA |
| ｐ3178 | 39 | 0.41 (0.31, 0.5) | 42 | 0.62 (0.59, 0.64) | 0.5933 | NA |
| ｐ989 | 1 | 0.35 (0, 0) | 42 | 0.52 (0.49, 0.56) | 0.5921 | NA |
| ｐ6560 | 39 | 0.27 (0.04, 0.49) | 42 | 0.4 (0.24, 0.57) | 0.5836 | NA |
| ｐ535 | 39 | 0.18 (0.11, 0.25) | 42 | 0.27 (0.16, 0.38) | 0.5831 | NA |
| ｐ2909 | 39 | 0.32 (0.3, 0.35) | 42 | 0.48 (0.41, 0.56) | 0.5762 | NA |
| ｐ4536 | 39 | 0.2 (0.09, 0.31) | 42 | 0.3 (0.2, 0.4) | 0.5699 | NA |
| ｐ1956 | 1 | 0.59 (0, 0) | 42 | 0.88 (0.87, 0.9) | 0.5692 | NA |
| ｐ6162 | 39 | 0.26 (-0.04, 0.56) | 42 | 0.38 (0.25, 0.51) | 0.5510 | NA |
| ｐ3353 | 39 | 0.42 (0.37, 0.47) | 42 | 0.61 (0.37, 0.84) | 0.5282 | NA |
| ｐ2814 | 1 | 1.36 (0, 0) | 1 | 1.95 (0, 0) | 0.5219 | NA |
| ｐ6713 | 39 | 0.55 (0.54, 0.56) | 42 | 0.79 (0.77, 0.8) | 0.5158 | NA |
| ｐ6163 | 39 | 0.33 (0.2, 0.47) | 42 | 0.48 (0.37, 0.58) | 0.5157 | NA |
| ｐ3494 | 39 | 0.46 (0.3, 0.62) | 1 | 0.66 (0, 0) | 0.5101 | NA |
| ｐ9940 | 39 | 0.18 (0.05, 0.3) | 42 | 0.25 (0.22, 0.29) | 0.5060 | NA |
| ｐ540 | 39 | 0.29 (0.2, 0.38) | 42 | 0.41 (0.38, 0.43) | 0.5011 | NA |
| ｐ5534 | 39 | 0.34 (0.24, 0.44) | 42 | 0.48 (0.44, 0.51) | 0.5005 | NA |
| ｐ1038 | 39 | 0.73 (0.67, 0.79) | 42 | 1.03 (0.45, 1.61) | 0.4958 | NA |
| ｐ6617 | 39 | 0.5 (0.36, 0.64) | 1 | 0.71 (0, 0) | 0.4951 | NA |
| ｐ1139 | 39 | 0.65 (-1.85, 3.15) | 42 | 0.91 (0.65, 1.17) | 0.4915 | NA |
| ｐ1214 | 39 | 0.33 (0.31, 0.35) | 42 | 0.46 (0.34, 0.58) | 0.4893 | NA |
| ｐ466 | 39 | 0.42 (0.29, 0.54) | 42 | 0.58 (0.57, 0.6) | 0.4845 | NA |
| ｐ6098 | 1 | 0.73 (0, 0) | 1 | 1.02 (0, 0) | 0.4790 | NA |
| ｐ5883 | 39 | 0.32 (0.09, 0.54) | 42 | 0.44 (0.27, 0.62) | 0.4780 | NA |
| ｐ9666 | 39 | 0.66 (0.64, 0.68) | 42 | 0.92 (0.56, 1.28) | 0.4707 | NA |
| ｐ4364 | 1 | 1.15 (0, 0) | 1 | 1.59 (0, 0) | 0.4689 | NA |
| ｐ5144 | 1 | 0.54 (0, 0) | 42 | 0.75 (0.35, 1.15) | 0.4599 | NA |
| ｐ5960 | 1 | 1 (0, 0) | 1 | 1.38 (0, 0) | 0.4591 | NA |
| ｐ9449 | 1 | 0.4 (0, 0) | 42 | 0.55 (0.52, 0.58) | 0.4559 | NA |
| ｐ6052 | 39 | 0.37 (0.24, 0.49) | 42 | 0.5 (0.39, 0.62) | 0.4555 | NA |
| ｐ6667 | 39 | 0.5 (0.48, 0.52) | 42 | 0.68 (0.56, 0.81) | 0.4548 | NA |
| ｐ5619 | 39 | 0.71 (0.33, 1.09) | 1 | 0.97 (0, 0) | 0.4516 | NA |
| ｐ3262 | 39 | 0.6 (0.44, 0.75) | 42 | 0.81 (0.31, 1.31) | 0.4388 | NA |
| ｐ3290 | 39 | 0.62 (0.52, 0.71) | 42 | 0.83 (0.66, 1.01) | 0.4275 | NA |
| ｐ3390 | 39 | 0.53 (0.4, 0.65) | 42 | 0.7 (0.66, 0.74) | 0.4085 | NA |
| ｐ161 | 39 | 0.65 (0.39, 0.92) | 1 | 0.87 (0, 0) | 0.4078 | NA |
| ｐ1845 | 39 | 0.15 (0.1, 0.2) | 42 | 0.2 (0.16, 0.23) | 0.4037 | NA |
| ｐ2934 | 39 | 0.29 (0.1, 0.49) | 42 | 0.39 (0.27, 0.5) | 0.3994 | NA |
| ｐ6688 | 39 | 0.42 (0.4, 0.45) | 42 | 0.56 (0.55, 0.56) | 0.3982 | NA |
| ｐ1510 | 39 | 0.23 (0.16, 0.3) | 42 | 0.31 (0.21, 0.41) | 0.3979 | NA |
| ｐ9863 | 1 | 0.83 (0, 0) | 42 | 1.07 (0.76, 1.39) | 0.3758 | NA |
| ｐ3883 | 39 | 0.28 (0.22, 0.35) | 42 | 0.36 (0.14, 0.58) | 0.3691 | NA |
| ｐ9099 | 39 | 0.37 (0.37, 0.37) | 42 | 0.47 (0.26, 0.68) | 0.3548 | NA |
| ｐ7219 | 39 | 0.93 (0.92, 0.94) | 42 | 1.18 (1.16, 1.21) | 0.3480 | NA |
| ｐ1831 | 39 | 0.49 (0.26, 0.71) | 42 | 0.62 (0.59, 0.65) | 0.3477 | NA |
| ｐ9294 | 39 | 0.66 (0.64, 0.68) | 1 | 0.84 (0, 0) | 0.3409 | NA |
| ｐ9544 | 1 | 0.72 (0, 0) | 1 | 0.9 (0, 0) | 0.3228 | NA |
| ｐ1374 | 39 | 0.34 (0.22, 0.47) | 42 | 0.43 (0.29, 0.57) | 0.3183 | NA |
| ｐ3198 | 39 | 0.62 (0.28, 0.96) | 42 | 0.77 (0.66, 0.87) | 0.3079 | NA |
| ｐ1304 | 39 | 0.22 (0.16, 0.28) | 42 | 0.27 (0.21, 0.34) | 0.2978 | NA |
| ｐ5524 | 39 | 0.1 (0.05, 0.15) | 42 | 0.13 (0.07, 0.18) | 0.2926 | NA |
| ｐ9963 | 39 | 0.8 (0.73, 0.86) | 1 | 0.97 (0, 0) | 0.2873 | NA |
| ｐ1217 | 39 | 0.3 (0.27, 0.34) | 1 | 0.37 (0, 0) | 0.2816 | NA |
| ｐ9680 | 39 | 0.52 (0.44, 0.6) | 1 | 0.63 (0, 0) | 0.2790 | NA |
| ｐ9040 | 39 | 0.18 (0.13, 0.23) | 42 | 0.22 (0.16, 0.28) | 0.2778 | NA |
| ｐ3432 | 39 | 0.63 (0.41, 0.85) | 42 | 0.76 (0.75, 0.77) | 0.2728 | NA |
| ｐ2349 | 39 | 0.48 (0.47, 0.5) | 42 | 0.58 (0.52, 0.65) | 0.2681 | NA |
| ｐ7717 | 1 | 0.74 (0, 0) | 1 | 0.89 (0, 0) | 0.2657 | NA |
| ｐ489 | 39 | 0.41 (0.31, 0.51) | 42 | 0.49 (0.31, 0.67) | 0.2641 | NA |
| ｐ5034 | 1 | 1 (0, 0) | 42 | 1.21 (1.2, 1.21) | 0.2630 | NA |
| ｐ3423 | 39 | 0.37 (0.34, 0.41) | 42 | 0.44 (0.23, 0.66) | 0.2442 | NA |
| ｐ5660 | 39 | 0.23 (0.14, 0.31) | 42 | 0.27 (0.19, 0.35) | 0.2410 | NA |
| ｐ1817 | 39 | 0.5 (0.43, 0.57) | 42 | 0.59 (0.37, 0.81) | 0.2337 | NA |
| ｐ2489 | 39 | 0.88 (0.73, 1.03) | 1 | 1.04 (0, 0) | 0.2334 | NA |
| ｐ2509 | 39 | 0.22 (0.15, 0.3) | 42 | 0.26 (0.19, 0.33) | 0.2256 | NA |
| ｐ3517 | 1 | 0.85 (0, 0) | 1 | 0.99 (0, 0) | 0.2194 | NA |
| ｐ4484 | 1 | 0.32 (0, 0) | 42 | 0.37 (0.25, 0.5) | 0.2166 | NA |
| ｐ1275 | 39 | 0.43 (0.34, 0.52) | 42 | 0.5 (0.41, 0.59) | 0.2039 | NA |
| ｐ1488 | 39 | 0.72 (0.55, 0.88) | 42 | 0.83 (0.67, 0.98) | 0.2024 | NA |
| ｐ3217 | 39 | 0.29 (0.2, 0.38) | 42 | 0.33 (0.24, 0.42) | 0.2023 | NA |
| ｐ1037 | 1 | 0.55 (0, 0) | 42 | 0.63 (0.57, 0.69) | 0.1987 | NA |
| ｐ7371 | 39 | 0.68 (0.29, 1.08) | 42 | 0.78 (0.75, 0.82) | 0.1916 | NA |
| ｐ2515 | 39 | 0.52 (0.49, 0.55) | 42 | 0.59 (0.55, 0.64) | 0.1817 | NA |
| ｐ2522 | 39 | 0.54 (0.49, 0.59) | 42 | 0.61 (0.28, 0.94) | 0.1807 | NA |
| ｐ3848 | 39 | 0.58 (0.39, 0.77) | 42 | 0.65 (0.57, 0.73) | 0.1686 | NA |
| ｐ2268 | 39 | 0.5 (0.33, 0.68) | 42 | 0.56 (0.37, 0.76) | 0.1644 | NA |
| ｐ5934 | 1 | 0.74 (0, 0) | 1 | 0.83 (0, 0) | 0.1638 | NA |
| ｐ546 | 39 | 0.37 (0.34, 0.4) | 42 | 0.41 (0.24, 0.59) | 0.1637 | NA |
| ｐ6606 | 39 | 0.41 (0.37, 0.45) | 42 | 0.46 (0.33, 0.59) | 0.1595 | NA |
| ｐ9118 | 39 | 2.24 (2.18, 2.3) | 1 | 2.5 (0, 0) | 0.1586 | NA |
| ｐ6115 | 1 | 1.29 (0, 0) | 1 | 1.43 (0, 0) | 0.1504 | NA |
| ｐ2911 | 39 | 0.61 (0.54, 0.68) | 42 | 0.67 (-0.16, 1.51) | 0.1430 | NA |
| ｐ3824 | 39 | 0.47 (0.36, 0.59) | 42 | 0.52 (0.26, 0.77) | 0.1258 | NA |
| ｐ5515 | 39 | 0.63 (0.62, 0.64) | 42 | 0.69 (0.65, 0.72) | 0.1158 | NA |
| ｐ513 | 39 | 0.72 (0.48, 0.95) | 42 | 0.78 (0.59, 0.96) | 0.1147 | NA |
| ｐ2453 | 1 | 1.02 (0, 0) | 1 | 1.1 (0, 0) | 0.1135 | NA |
| ｐ6762 | 39 | 0.35 (0.32, 0.39) | 42 | 0.38 (0.27, 0.49) | 0.1092 | NA |
| ｐ6773 | 39 | 0.34 (0.23, 0.46) | 42 | 0.36 (0.3, 0.43) | 0.0877 | NA |
| ｐ6827 | 39 | 0.69 (0.68, 0.71) | 1 | 0.73 (0, 0) | 0.0856 | NA |
| ｐ5335 | 1 | 0.83 (0, 0) | 1 | 0.88 (0, 0) | 0.0831 | NA |
| ｐ1997 | 39 | 0.51 (0.47, 0.55) | 42 | 0.54 (0.39, 0.7) | 0.0811 | NA |
| ｐ3137 | 39 | 0.28 (0.21, 0.34) | 42 | 0.29 (0.18, 0.4) | 0.0802 | NA |
| ｐ6155 | 39 | 0.46 (0.43, 0.5) | 42 | 0.49 (0.2, 0.78) | 0.0754 | NA |
| ｐ6744 | 39 | 0.35 (0.3, 0.39) | 42 | 0.36 (0.33, 0.39) | 0.0702 | NA |
| ｐ9706 | 1 | 1.14 (0, 0) | 1 | 1.19 (0, 0) | 0.0588 | NA |
| ｐ3409 | 39 | 0.45 (0.27, 0.64) | 1 | 0.47 (0, 0) | 0.0584 | NA |
| ｐ5823 | 39 | 0.31 (0.25, 0.37) | 42 | 0.32 (0.18, 0.46) | 0.0557 | NA |
| ｐ2347 | 39 | 0.47 (0.43, 0.52) | 42 | 0.49 (0.47, 0.5) | 0.0452 | NA |
| ｐ1509 | 39 | 0.18 (0.08, 0.28) | 42 | 0.19 (0.12, 0.25) | 0.0395 | NA |
| ｐ6151 | 39 | 0.49 (0.07, 0.9) | 42 | 0.5 (0.46, 0.53) | 0.0371 | NA |
| ｐ4548 | 39 | 0.37 (0.12, 0.62) | 42 | 0.38 (0.22, 0.54) | 0.0335 | NA |
| ｐ438 | 1 | 0.79 (0, 0) | 42 | 0.79 (0.68, 0.91) | 0.0081 | NA |
| ｐ6138 | 39 | 0.64 (0.47, 0.81) | 1 | 0.65 (0, 0) | 0.0077 | NA |
| ｐ2070 | 39 | 0.46 (0.38, 0.54) | 42 | 0.46 (0.34, 0.59) | -0.0008 | NA |
| ｐ2395 | 1 | 1.27 (0, 0) | 1 | 1.25 (0, 0) | -0.0166 | NA |
| ｐ9480 | 39 | 1.09 (1.09, 1.1) | 1 | 1.08 (0, 0) | -0.0168 | NA |
| ｐ2843 | 1 | 0.75 (0, 0) | 42 | 0.73 (0.72, 0.75) | -0.0261 | NA |
| ｐ1491 | 39 | 0.75 (0.58, 0.91) | 42 | 0.73 (0.64, 0.83) | -0.0281 | NA |
| ｐ1794 | 39 | 0.41 (0.37, 0.46) | 42 | 0.4 (0.34, 0.46) | -0.0326 | NA |
| ｐ5777 | 39 | 0.54 (0.47, 0.62) | 42 | 0.53 (0.32, 0.74) | -0.0338 | NA |
| ｐ3320 | 39 | 0.57 (0.56, 0.58) | 42 | 0.56 (0.53, 0.58) | -0.0437 | NA |
| ｐ3312 | 39 | 0.42 (0.39, 0.45) | 42 | 0.41 (-1.12, 1.93) | -0.0447 | NA |
| ｐ3379 | 39 | 0.7 (0.61, 0.79) | 42 | 0.67 (0.55, 0.8) | -0.0454 | NA |
| ｐ473 | 39 | 0.57 (0.56, 0.58) | 42 | 0.55 (0.4, 0.71) | -0.0464 | NA |
| ｐ6136 | 39 | 0.73 (0.72, 0.73) | 42 | 0.71 (0.54, 0.87) | -0.0468 | NA |
| ｐ3394 | 39 | 0.41 (0.14, 0.68) | 42 | 0.39 (0.29, 0.5) | -0.0480 | NA |
| ｐ1798 | 39 | 0.3 (0.24, 0.36) | 42 | 0.29 (0.26, 0.31) | -0.0604 | NA |
| ｐ6147 | 1 | 0.75 (0, 0) | 42 | 0.72 (0.32, 1.12) | -0.0610 | NA |
| ｐ3328 | 39 | 0.58 (0.45, 0.71) | 42 | 0.55 (0.53, 0.57) | -0.0806 | NA |
| ｐ4547 | 39 | 0.71 (0.49, 0.92) | 42 | 0.66 (0.42, 0.9) | -0.0955 | NA |
| ｐ7654 | 39 | 0.79 (0.77, 0.82) | 42 | 0.73 (-0.85, 2.31) | -0.1117 | NA |
| ｐ9652 | 39 | 0.28 (0.1, 0.47) | 42 | 0.26 (0.24, 0.28) | -0.1268 | NA |
| ｐ9922 | 39 | 0.5 (0.42, 0.58) | 42 | 0.45 (0.21, 0.7) | -0.1450 | NA |
| ｐ3223 | 39 | 0.51 (0.31, 0.72) | 42 | 0.46 (0.4, 0.53) | -0.1464 | NA |
| ｐ6186 | 39 | 0.67 (0.62, 0.72) | 42 | 0.6 (0.41, 0.8) | -0.1538 | NA |
| ｐ5565 | 39 | 0.46 (0.31, 0.6) | 42 | 0.41 (0.33, 0.49) | -0.1597 | NA |
| ｐ2335 | 39 | 0.53 (0.33, 0.72) | 42 | 0.47 (0.27, 0.67) | -0.1671 | NA |
| ｐ2894 | 39 | 0.42 (0.36, 0.47) | 42 | 0.37 (0.32, 0.43) | -0.1710 | NA |
| ｐ1004 | 39 | 1.2 (0.87, 1.53) | 1 | 1.06 (0, 0) | -0.1805 | NA |
| ｐ8128 | 39 | 0.26 (0.08, 0.44) | 42 | 0.23 (0.17, 0.29) | -0.1820 | NA |
| ｐ762 | 39 | 0.21 (0.17, 0.25) | 1 | 0.18 (0, 0) | -0.1835 | NA |
| ｐ1360 | 39 | 0.46 (0.35, 0.57) | 42 | 0.4 (0.3, 0.5) | -0.2004 | NA |
| ｐ4111 | 39 | 0.88 (0.72, 1.05) | 1 | 0.77 (0, 0) | -0.2005 | NA |
| ｐ2844 | 39 | 0.72 (0.7, 0.74) | 42 | 0.62 (0.13, 1.1) | -0.2135 | NA |
| ｐ2092 | 39 | 0.92 (0.9, 0.93) | 1 | 0.79 (0, 0) | -0.2146 | NA |
| ｐ4543 | 39 | 0.73 (0.45, 1.01) | 42 | 0.63 (0.51, 0.75) | -0.2162 | NA |
| ｐ4710 | 1 | 0.61 (0, 0) | 42 | 0.52 (0.5, 0.53) | -0.2267 | NA |
| ｐ5604 | 1 | 0.97 (0, 0) | 42 | 0.83 (0.46, 1.19) | -0.2288 | NA |
| ｐ3229 | 39 | 0.7 (0.49, 0.92) | 42 | 0.6 (0.55, 0.64) | -0.2321 | NA |
| ｐ3401 | 39 | 0.67 (0.58, 0.76) | 42 | 0.57 (0.48, 0.65) | -0.2403 | NA |
| ｐ2739 | 1 | 1.07 (0, 0) | 42 | 0.9 (0.48, 1.31) | -0.2508 | NA |
| ｐ4401 | 39 | 0.74 (0.6, 0.88) | 1 | 0.61 (0, 0) | -0.2741 | NA |
| ｐ5169 | 39 | 0.62 (0.22, 1.02) | 42 | 0.51 (0.44, 0.59) | -0.2772 | NA |
| ｐ647 | 39 | 0.79 (0.61, 0.98) | 42 | 0.65 (0.63, 0.68) | -0.2790 | NA |
| ｐ7981 | 39 | 0.73 (0.32, 1.14) | 42 | 0.6 (0.28, 0.92) | -0.2824 | NA |
| ｐ4546 | 39 | 0.5 (0.37, 0.63) | 42 | 0.41 (0.2, 0.62) | -0.2880 | NA |
| ｐ3365 | 39 | 0.52 (0.43, 0.61) | 42 | 0.43 (0.29, 0.56) | -0.2888 | NA |
| ｐ1789 | 39 | 0.47 (0.36, 0.59) | 42 | 0.38 (0.34, 0.43) | -0.3013 | NA |
| ｐ9370 | 39 | 0.26 (0.24, 0.29) | 1 | 0.21 (0, 0) | -0.3078 | NA |
| ｐ8028 | 1 | 0.76 (0, 0) | 42 | 0.61 (0.49, 0.73) | -0.3190 | NA |
| ｐ2758 | 39 | 1.4 (1.39, 1.4) | 42 | 1.12 (0.21, 2.02) | -0.3217 | NA |
| ｐ4470 | 39 | 1.19 (0.27, 2.12) | 1 | 0.95 (0, 0) | -0.3222 | NA |
| ｐ2003 | 39 | 0.34 (0.18, 0.5) | 42 | 0.26 (0.19, 0.34) | -0.3445 | NA |
| ｐ7749 | 1 | 1.07 (0, 0) | 42 | 0.85 (0.62, 1.07) | -0.3446 | NA |
| ｐ6188 | 39 | 0.64 (0.44, 0.84) | 42 | 0.5 (0.44, 0.56) | -0.3610 | NA |
| ｐ1092 | 39 | 0.38 (0.25, 0.51) | 42 | 0.3 (0.14, 0.45) | -0.3657 | NA |
| ｐ5656 | 39 | 0.36 (0.2, 0.53) | 42 | 0.28 (0.2, 0.36) | -0.3694 | NA |
| ｐ6652 | 1 | 0.67 (0, 0) | 42 | 0.52 (0.5, 0.54) | -0.3695 | NA |
| ｐ3376 | 39 | 1.03 (1, 1.05) | 42 | 0.8 (0.72, 0.87) | -0.3708 | NA |
| ｐ8045 | 39 | 0.88 (0.76, 1.01) | 42 | 0.68 (0.56, 0.8) | -0.3743 | NA |
| ｐ1272 | 39 | 0.53 (0.41, 0.66) | 42 | 0.41 (0.32, 0.5) | -0.3761 | NA |
| ｐ6991 | 39 | 0.43 (0.34, 0.51) | 42 | 0.33 (0.25, 0.41) | -0.3784 | NA |
| ｐ8743 | 39 | 0.23 (0.08, 0.37) | 42 | 0.17 (0.1, 0.25) | -0.3927 | NA |
| ｐ6025 | 1 | 1.44 (0, 0) | 1 | 1.09 (0, 0) | -0.3958 | NA |
| ｐ7379 | 39 | 0.1 (0.08, 0.12) | 42 | 0.08 (0.02, 0.14) | -0.3978 | NA |
| ｐ159 | 39 | 0.5 (0.34, 0.66) | 42 | 0.38 (0.25, 0.5) | -0.4005 | NA |
| ｐ4160 | 1 | 1.04 (0, 0) | 1 | 0.78 (0, 0) | -0.4078 | NA |
| ｐ3230 | 39 | 0.65 (0.6, 0.69) | 42 | 0.49 (0.45, 0.52) | -0.4160 | NA |
| ｐ3667 | 39 | 0.63 (0.56, 0.7) | 42 | 0.47 (0.2, 0.74) | -0.4255 | NA |
| ｐ9450 | 39 | 0.59 (0.55, 0.62) | 42 | 0.43 (0.31, 0.56) | -0.4451 | NA |
| ｐ2008 | 39 | 0.45 (0.37, 0.53) | 42 | 0.33 (0.09, 0.56) | -0.4493 | NA |
| ｐ1318 | 39 | 0.17 (0.03, 0.31) | 42 | 0.12 (0, 0.24) | -0.4589 | NA |
| ｐ6191 | 39 | 0.45 (0.38, 0.51) | 42 | 0.32 (0.28, 0.36) | -0.4641 | NA |
| ｐ1439 | 39 | 0.85 (0.75, 0.96) | 42 | 0.62 (0.48, 0.76) | -0.4663 | NA |
| ｐ9712 | 1 | 1.91 (0, 0) | 1 | 1.38 (0, 0) | -0.4700 | NA |
| ｐ5713 | 39 | 0.36 (0.32, 0.4) | 42 | 0.26 (0.04, 0.48) | -0.4706 | NA |
| ｐ8845 | 1 | 1.17 (0, 0) | 42 | 0.83 (0.82, 0.85) | -0.4953 | NA |
| ｐ2526 | 39 | 0.93 (0.59, 1.28) | 42 | 0.66 (0.6, 0.73) | -0.4964 | NA |
| ｐ3322 | 39 | 0.59 (0.57, 0.62) | 42 | 0.42 (0.29, 0.54) | -0.5102 | NA |
| ｐ7366 | 1 | 0.58 (0, 0) | 42 | 0.41 (0.03, 0.79) | -0.5117 | NA |
| ｐ7286 | 1 | 1.12 (0, 0) | 42 | 0.79 (0.78, 0.79) | -0.5123 | NA |
| ｐ2833 | 1 | 0.73 (0, 0) | 1 | 0.51 (0, 0) | -0.5312 | NA |
| ｐ528 | 39 | 0.3 (0.12, 0.47) | 42 | 0.2 (0.14, 0.27) | -0.5374 | NA |
| ｐ3166 | 39 | 0.64 (0.39, 0.88) | 42 | 0.44 (0.27, 0.61) | -0.5412 | NA |
| ｐ9333 | 1 | 0.99 (0, 0) | 42 | 0.67 (0.54, 0.8) | -0.5494 | NA |
| ｐ8934 | 39 | 0.53 (0.4, 0.66) | 42 | 0.36 (0.18, 0.54) | -0.5549 | NA |
| ｐ1393 | 39 | 0.68 (0.48, 0.88) | 42 | 0.46 (0.29, 0.63) | -0.5735 | NA |
| ｐ3270 | 39 | 0.82 (0.72, 0.93) | 42 | 0.55 (0.46, 0.64) | -0.5777 | NA |
| ｐ911 | 39 | 1.09 (0.87, 1.31) | 42 | 0.73 (0.7, 0.76) | -0.5837 | NA |
| ｐ3308 | 39 | 0.93 (0.91, 0.96) | 42 | 0.62 (0.56, 0.68) | -0.5857 | NA |
| ｐ1306 | 39 | 0.17 (0.1, 0.24) | 42 | 0.11 (-0.02, 0.24) | -0.5857 | NA |
| ｐ3330 | 39 | 0.58 (0.56, 0.61) | 42 | 0.39 (-0.22, 1) | -0.5890 | NA |
| ｐ6995 | 39 | 0.27 (0.18, 0.35) | 42 | 0.18 (0.15, 0.21) | -0.5950 | NA |
| ｐ9924 | 39 | 0.31 (0.06, 0.57) | 42 | 0.21 (0.09, 0.33) | -0.5982 | NA |
| ｐ3278 | 39 | 1.08 (0.85, 1.3) | 42 | 0.71 (0.68, 0.73) | -0.6060 | NA |
| ｐ1988 | 39 | 0.87 (0.85, 0.88) | 42 | 0.57 (0.23, 0.9) | -0.6073 | NA |
| ｐ5655 | 39 | 0.61 (0.55, 0.67) | 42 | 0.4 (0.24, 0.56) | -0.6101 | NA |
| ｐ1285 | 39 | 0.78 (0.71, 0.85) | 42 | 0.5 (0.46, 0.55) | -0.6356 | NA |
| ｐ5785 | 39 | 0.7 (0.48, 0.92) | 42 | 0.45 (0.37, 0.53) | -0.6374 | NA |
| ｐ2533 | 39 | 0.8 (0.73, 0.86) | 1 | 0.51 (0, 0) | -0.6374 | NA |
| ｐ551 | 39 | 0.41 (0.23, 0.59) | 42 | 0.26 (0.17, 0.35) | -0.6529 | NA |
| ｐ1422 | 39 | 0.77 (0.6, 0.95) | 42 | 0.49 (0.44, 0.54) | -0.6564 | NA |
| ｐ1205 | 1 | 1.42 (0, 0) | 42 | 0.9 (0.88, 0.91) | -0.6699 | NA |
| ｐ3350 | 39 | 0.53 (0.37, 0.7) | 42 | 0.34 (0.29, 0.38) | -0.6722 | NA |
| ｐ3380 | 39 | 0.96 (0.83, 1.1) | 42 | 0.6 (0.43, 0.78) | -0.6732 | NA |
| ｐ5484 | 39 | 0.68 (0.47, 0.9) | 42 | 0.42 (0.29, 0.56) | -0.6846 | NA |
| ｐ5997 | 1 | 1.66 (0, 0) | 1 | 1.01 (0, 0) | -0.7115 | NA |
| ｐ3340 | 39 | 1.18 (0.55, 1.81) | 42 | 0.71 (0.68, 0.74) | -0.7345 | NA |
| ｐ1445 | 39 | 0.93 (0.79, 1.08) | 42 | 0.56 (0.4, 0.72) | -0.7358 | NA |
| ｐ4553 | 39 | 0.54 (0.37, 0.71) | 42 | 0.32 (0.21, 0.43) | -0.7375 | NA |
| ｐ7128 | 1 | 0.93 (0, 0) | 42 | 0.56 (0.36, 0.75) | -0.7386 | NA |
| ｐ3125 | 1 | 0.79 (0, 0) | 42 | 0.47 (0.45, 0.49) | -0.7491 | NA |
| ｐ1784 | 1 | 0.79 (0, 0) | 42 | 0.47 (0.36, 0.58) | -0.7504 | NA |
| ｐ3377 | 39 | 0.92 (0.85, 1) | 42 | 0.54 (0.46, 0.63) | -0.7620 | NA |
| ｐ3454 | 39 | 0.75 (0.74, 0.76) | 42 | 0.44 (0.39, 0.49) | -0.7684 | NA |
| ｐ2951 | 39 | 0.5 (0.45, 0.55) | 42 | 0.29 (0.26, 0.33) | -0.7687 | NA |
| ｐ7681 | 1 | 1.03 (0, 0) | 42 | 0.6 (0.1, 1.1) | -0.7794 | NA |
| ｐ514 | 39 | 0.69 (0.5, 0.88) | 42 | 0.4 (0.3, 0.5) | -0.7806 | NA |
| ｐ8717 | 39 | 2.24 (2.24, 2.25) | 42 | 1.27 (1.27, 1.28) | -0.8154 | NA |
| ｐ5680 | 39 | 0.65 (0.51, 0.78) | 42 | 0.37 (0.22, 0.51) | -0.8187 | NA |
| ｐ1371 | 39 | 0.41 (0.31, 0.51) | 42 | 0.23 (0.11, 0.35) | -0.8313 | NA |
| ｐ5697 | 39 | 0.29 (0.14, 0.44) | 42 | 0.16 (0.11, 0.22) | -0.8420 | NA |
| ｐ3368 | 39 | 0.68 (0.53, 0.82) | 42 | 0.38 (0.29, 0.47) | -0.8429 | NA |
| ｐ3219 | 39 | 0.84 (0.81, 0.87) | 42 | 0.47 (0.27, 0.66) | -0.8433 | NA |
| ｐ8862 | 39 | 0.82 (0.81, 0.83) | 42 | 0.45 (0.3, 0.61) | -0.8508 | NA |
| ｐ1141 | 39 | 0.71 (0.44, 0.98) | 42 | 0.39 (0.32, 0.47) | -0.8535 | NA |
| ｐ5490 | 39 | 0.7 (0.69, 0.71) | 42 | 0.39 (0.29, 0.48) | -0.8573 | NA |
| ｐ3258 | 39 | 0.68 (0.59, 0.77) | 42 | 0.37 (0.28, 0.47) | -0.8582 | NA |
| ｐ3393 | 39 | 0.57 (0.43, 0.71) | 42 | 0.31 (0.05, 0.58) | -0.8644 | NA |
| ｐ464 | 39 | 0.56 (0.41, 0.7) | 42 | 0.31 (0.18, 0.43) | -0.8669 | NA |
| ｐ4661 | 39 | 0.74 (0.24, 1.23) | 42 | 0.4 (0.36, 0.45) | -0.8756 | NA |
| ｐ3282 | 39 | 0.74 (0.72, 0.77) | 42 | 0.4 (0.26, 0.54) | -0.8878 | NA |
| ｐ1108 | 39 | 0.4 (0.24, 0.56) | 42 | 0.21 (0.19, 0.24) | -0.9049 | NA |
| ｐ5560 | 39 | 0.47 (0.38, 0.57) | 42 | 0.25 (0.11, 0.39) | -0.9051 | NA |
| ｐ1435 | 39 | 1.03 (0.86, 1.19) | 42 | 0.55 (0.42, 0.68) | -0.9054 | NA |
| ｐ6715 | 39 | 0.79 (0.78, 0.81) | 42 | 0.42 (0.37, 0.46) | -0.9178 | NA |
| ｐ1083 | 39 | 0.77 (0.74, 0.81) | 42 | 0.41 (0.3, 0.51) | -0.9335 | NA |
| ｐ667 | 39 | 0.74 (0.72, 0.76) | 42 | 0.39 (0.34, 0.44) | -0.9344 | NA |
| ｐ2014 | 39 | 0.82 (0.58, 1.06) | 42 | 0.43 (0.35, 0.5) | -0.9432 | NA |
| ｐ9054 | 39 | 0.13 (0.07, 0.19) | 42 | 0.07 (-0.13, 0.26) | -0.9432 | NA |
| ｐ3310 | 39 | 0.72 (0.66, 0.79) | 42 | 0.37 (0.34, 0.4) | -0.9794 | NA |
| ｐ6081 | 1 | 1.26 (0, 0) | 42 | 0.64 (0.45, 0.82) | -0.9801 | NA |
| ｐ3171 | 39 | 0.74 (0.56, 0.91) | 42 | 0.37 (0.28, 0.46) | -0.9966 | NA |
| ｐ160 | 39 | 0.48 (0.35, 0.61) | 42 | 0.24 (-0.02, 0.49) | -1.0183 | NA |
| ｐ2845 | 39 | 1.24 (0.74, 1.74) | 1 | 0.61 (0, 0) | -1.0224 | NA |
| ｐ6785 | 39 | 0.38 (0.31, 0.44) | 42 | 0.18 (0.13, 0.24) | -1.0273 | NA |
| ｐ1216 | 39 | 0.56 (0.43, 0.68) | 42 | 0.27 (0.24, 0.31) | -1.0307 | NA |
| ｐ5547 | 39 | 0.44 (0.41, 0.47) | 42 | 0.21 (0.14, 0.29) | -1.0325 | NA |
| ｐ4652 | 1 | 1.08 (0, 0) | 42 | 0.52 (0.28, 0.76) | -1.0529 | NA |
| ｐ4416 | 39 | 0.76 (0.53, 0.99) | 42 | 0.36 (0.27, 0.45) | -1.0733 | NA |
| ｐ1135 | 39 | 0.61 (0.44, 0.78) | 42 | 0.29 (0.25, 0.33) | -1.0954 | NA |
| ｐ1849 | 39 | 1.1 (0.7, 1.5) | 1 | 0.51 (0, 0) | -1.0978 | NA |
| ｐ3249 | 39 | 0.8 (0.73, 0.87) | 42 | 0.37 (0.34, 0.4) | -1.1063 | NA |
| ｐ3363 | 39 | 0.92 (0.89, 0.95) | 42 | 0.43 (0.34, 0.51) | -1.1069 | NA |
| ｐ3187 | 39 | 0.73 (0.69, 0.78) | 42 | 0.34 (0.3, 0.38) | -1.1111 | NA |
| ｐ9235 | 39 | 1.3 (1.13, 1.46) | 42 | 0.6 (0.56, 0.64) | -1.1125 | NA |
| ｐ511 | 39 | 1.01 (1.01, 1.01) | 42 | 0.46 (0.24, 0.69) | -1.1222 | NA |
| ｐ7142 | 39 | 0.72 (0.52, 0.92) | 42 | 0.33 (-0.13, 0.79) | -1.1239 | NA |
| ｐ1080 | 39 | 1.77 (1.37, 2.17) | 42 | 0.81 (0.78, 0.85) | -1.1261 | NA |
| ｐ7329 | 39 | 0.78 (0.78, 0.79) | 42 | 0.36 (0.23, 0.49) | -1.1266 | NA |
| ｐ5710 | 39 | 0.72 (0.57, 0.87) | 42 | 0.33 (0.29, 0.37) | -1.1281 | NA |
| ｐ3179 | 39 | 0.66 (0.63, 0.69) | 42 | 0.3 (0.26, 0.35) | -1.1305 | NA |
| ｐ6568 | 39 | 0.67 (0.5, 0.84) | 42 | 0.3 (0.18, 0.43) | -1.1388 | NA |
| ｐ5200 | 39 | 0.69 (0.61, 0.77) | 42 | 0.31 (-0.04, 0.66) | -1.1479 | NA |
| ｐ6030 | 1 | 2.34 (0, 0) | 1 | 1.05 (0, 0) | -1.1514 | NA |
| ｐ5148 | 39 | 0.75 (0.33, 1.17) | 42 | 0.34 (0.3, 0.38) | -1.1551 | NA |
| ｐ9640 | 39 | 1.03 (0.97, 1.09) | 42 | 0.46 (0.29, 0.63) | -1.1637 | NA |
| ｐ2904 | 33 | 0.77 (0.55, 1) | 42 | 0.34 (0.32, 0.36) | -1.1694 | NA |
| ｐ4572 | 39 | 0.46 (0.32, 0.59) | 42 | 0.2 (0.13, 0.27) | -1.1775 | NA |
| ｐ8201 | 1 | 0.95 (0, 0) | 42 | 0.42 (0.19, 0.65) | -1.1797 | NA |
| ｐ3837 | 39 | 1.01 (0.75, 1.27) | 42 | 0.44 (0.25, 0.62) | -1.1987 | NA |
| ｐ4616 | 39 | 0.47 (0.31, 0.62) | 42 | 0.2 (0.07, 0.33) | -1.2031 | NA |
| ｐ4532 | 39 | 0.42 (0.4, 0.43) | 42 | 0.18 (0.11, 0.25) | -1.2040 | NA |
| ｐ1323 | 39 | 1.75 (1.36, 2.13) | 1 | 0.76 (0, 0) | -1.2062 | NA |
| ｐ3343 | 39 | 0.77 (0.73, 0.8) | 42 | 0.33 (0.25, 0.42) | -1.2073 | NA |
| ｐ3126 | 39 | 0.45 (0.43, 0.47) | 42 | 0.19 (0.13, 0.26) | -1.2186 | NA |
| ｐ4608 | 39 | 0.51 (0.37, 0.66) | 42 | 0.22 (0.07, 0.37) | -1.2205 | NA |
| ｐ3288 | 39 | 1.28 (1, 1.57) | 42 | 0.55 (0.46, 0.64) | -1.2264 | NA |
| ｐ5419 | 1 | 0.72 (0, 0) | 42 | 0.31 (0.31, 0.31) | -1.2288 | NA |
| ｐ7337 | 39 | 0.89 (0.75, 1.02) | 42 | 0.38 (0.27, 0.48) | -1.2301 | NA |
| ｐ3304 | 39 | 1.22 (0.75, 1.69) | 42 | 0.52 (0.47, 0.56) | -1.2344 | NA |
| ｐ5855 | 39 | 0.73 (0.59, 0.86) | 42 | 0.31 (0.2, 0.42) | -1.2367 | NA |
| ｐ3316 | 39 | 1.67 (0.09, 3.26) | 42 | 0.7 (0.29, 1.11) | -1.2507 | NA |
| ｐ1344 | 1 | 0.9 (0, 0) | 42 | 0.38 (0.19, 0.56) | -1.2540 | NA |
| ｐ6119 | 1 | 1.99 (0, 0) | 1 | 0.83 (0, 0) | -1.2575 | NA |
| ｐ3226 | 39 | 0.58 (0.51, 0.64) | 42 | 0.24 (0.23, 0.25) | -1.2724 | NA |
| ｐ460 | 39 | 0.82 (0.73, 0.91) | 42 | 0.34 (0.2, 0.48) | -1.2827 | NA |
| ｐ9459 | 1 | 0.9 (0, 0) | 42 | 0.37 (-0.65, 1.39) | -1.2866 | NA |
| ｐ8066 | 39 | 1.05 (0.31, 1.8) | 42 | 0.42 (0.25, 0.6) | -1.3135 | NA |
| ｐ4411 | 1 | 1.04 (0, 0) | 42 | 0.41 (0.35, 0.46) | -1.3570 | NA |
| ｐ3420 | 1 | 1.12 (0, 0) | 42 | 0.43 (0.39, 0.47) | -1.3770 | NA |
| ｐ2998 | 1 | 1.46 (0, 0) | 1 | 0.56 (0, 0) | -1.3824 | NA |
| ｐ5114 | 39 | 0.64 (0.44, 0.84) | 42 | 0.24 (-0.01, 0.5) | -1.3832 | NA |
| ｐ1303 | 1 | 0.74 (0, 0) | 42 | 0.28 (0.22, 0.34) | -1.4058 | NA |
| ｐ7405 | 39 | 1.22 (1.03, 1.41) | 42 | 0.45 (0.42, 0.49) | -1.4347 | NA |
| ｐ744 | 1 | 0.97 (0, 0) | 42 | 0.36 (0.27, 0.44) | -1.4432 | NA |
| ｐ3295 | 39 | 0.91 (0.73, 1.09) | 42 | 0.33 (-0.12, 0.78) | -1.4620 | NA |
| ｐ1842 | 39 | 0.61 (0.59, 0.64) | 42 | 0.22 (0.17, 0.27) | -1.4705 | NA |
| ｐ3261 | 39 | 1.01 (0.97, 1.05) | 42 | 0.36 (0.21, 0.51) | -1.4715 | NA |
| ｐ1261 | 1 | 1.02 (0, 0) | 42 | 0.36 (0.33, 0.4) | -1.4818 | NA |
| ｐ4626 | 39 | 0.93 (0.54, 1.33) | 40 | 0.33 (-1.76, 2.42) | -1.5009 | NA |
| ｐ3399 | 39 | 1.47 (1.43, 1.51) | 42 | 0.52 (0.43, 0.6) | -1.5050 | NA |
| ｐ3188 | 39 | 0.41 (0.37, 0.45) | 42 | 0.14 (-0.18, 0.47) | -1.5230 | NA |
| ｐ6682 | 39 | 0.5 (0.39, 0.6) | 42 | 0.17 (0.13, 0.21) | -1.5371 | NA |
| ｐ1210 | 39 | 0.89 (0.87, 0.91) | 42 | 0.31 (0.29, 0.33) | -1.5386 | NA |
| ｐ3211 | 39 | 0.38 (0.33, 0.43) | 42 | 0.13 (0.04, 0.22) | -1.5429 | NA |
| ｐ652 | 39 | 0.69 (0.67, 0.71) | 42 | 0.24 (0.12, 0.36) | -1.5455 | NA |
| ｐ3975 | 1 | 0.69 (0, 0) | 42 | 0.23 (0.07, 0.4) | -1.5689 | NA |
| ｐ3870 | 39 | 0.77 (0.68, 0.85) | 42 | 0.26 (0.2, 0.32) | -1.5691 | NA |
| ｐ1142 | 39 | 0.31 (0.23, 0.39) | 42 | 0.11 (0.01, 0.2) | -1.5702 | NA |
| ｐ8514 | 1 | 0.82 (0, 0) | 42 | 0.28 (0.22, 0.33) | -1.5742 | NA |
| ｐ756 | 39 | 0.52 (0.36, 0.69) | 42 | 0.18 (0.14, 0.21) | -1.5772 | NA |
| ｐ2889 | 1 | 1.54 (0, 0) | 42 | 0.51 (0.46, 0.57) | -1.5785 | NA |
| ｐ3317 | 39 | 1.66 (1.24, 2.09) | 42 | 0.55 (0.54, 0.56) | -1.5888 | NA |
| ｐ9285 | 1 | 2.09 (0, 0) | 42 | 0.69 (0.67, 0.71) | -1.6039 | NA |
| ｐ8326 | 1 | 2.46 (0, 0) | 42 | 0.8 (0.68, 0.92) | -1.6168 | NA |
| ｐ6047 | 1 | 1.01 (0, 0) | 42 | 0.33 (0.21, 0.45) | -1.6184 | NA |
| ｐ9090 | 39 | 1.16 (1.14, 1.18) | 42 | 0.37 (0.37, 0.37) | -1.6506 | NA |
| ｐ3391 | 39 | 0.78 (0.74, 0.81) | 42 | 0.25 (0.11, 0.38) | -1.6554 | NA |
| ｐ4593 | 1 | 1.21 (0, 0) | 42 | 0.38 (0.24, 0.52) | -1.6698 | NA |
| ｐ2925 | 39 | 1.34 (0.47, 2.21) | 42 | 0.41 (0.22, 0.61) | -1.6886 | NA |
| ｐ3349 | 39 | 1.24 (0.9, 1.57) | 42 | 0.38 (0.22, 0.54) | -1.6996 | NA |
| ｐ7618 | 1 | 1.13 (0, 0) | 42 | 0.35 (0.23, 0.47) | -1.7014 | NA |
| ｐ6135 | 39 | 2.44 (2, 2.89) | 42 | 0.73 (0.73, 0.74) | -1.7381 | NA |
| ｐ3266 | 39 | 1.44 (0.93, 1.96) | 42 | 0.43 (0.37, 0.49) | -1.7414 | NA |
| ｐ9883 | 39 | 0.8 (0.47, 1.12) | 42 | 0.23 (0.1, 0.37) | -1.7659 | NA |
| ｐ3462 | 39 | 0.96 (0.75, 1.18) | 42 | 0.28 (0.12, 0.45) | -1.7755 | NA |
| ｐ4660 | 39 | 1 (0.75, 1.25) | 42 | 0.29 (-0.18, 0.77) | -1.7772 | NA |
| ｐ9034 | 1 | 0.8 (0, 0) | 42 | 0.23 (0.19, 0.28) | -1.7785 | NA |
| ｐ8727 | 39 | 1.27 (1.27, 1.28) | 42 | 0.37 (0.23, 0.51) | -1.7907 | NA |
| ｐ4528 | 1 | 1.53 (0, 0) | 42 | 0.43 (0.42, 0.45) | -1.8213 | NA |
| ｐ5039 | 39 | 1.2 (1.19, 1.21) | 42 | 0.34 (0.2, 0.48) | -1.8365 | NA |
| ｐ8340 | 39 | 0.71 (0.59, 0.83) | 42 | 0.2 (0.14, 0.25) | -1.8609 | NA |
| ｐ1057 | 39 | 1.63 (1.12, 2.15) | 42 | 0.44 (0.37, 0.52) | -1.8784 | NA |
| ｐ155 | 1 | 1.44 (0, 0) | 42 | 0.39 (0.23, 0.54) | -1.8973 | NA |
| ｐ6131 | 1 | 2.87 (0, 0) | 42 | 0.77 (0.52, 1.02) | -1.9023 | NA |
| ｐ2459 | 1 | 2.33 (0, 0) | 42 | 0.62 (0.43, 0.8) | -1.9158 | NA |
| ｐ1278 | 39 | 0.6 (0.51, 0.69) | 42 | 0.16 (0.12, 0.19) | -1.9186 | NA |
| ｐ1349 | 39 | 0.57 (0.32, 0.82) | 42 | 0.15 (0.05, 0.26) | -1.9232 | NA |
| ｐ3405 | 39 | 0.72 (0.63, 0.81) | 42 | 0.19 (0.01, 0.37) | -1.9305 | NA |
| ｐ9588 | 1 | 1.34 (0, 0) | 42 | 0.35 (0.18, 0.52) | -1.9382 | NA |
| ｐ4006 | 39 | 0.51 (0.34, 0.68) | 1 | 0.13 (0, 0) | -1.9541 | NA |
| ｐ571 | 39 | 1.13 (0.9, 1.36) | 42 | 0.29 (0.23, 0.35) | -1.9668 | NA |
| ｐ5834 | 39 | 0.39 (0.25, 0.53) | 42 | 0.1 (-0.02, 0.22) | -1.9689 | NA |
| ｐ2865 | 1 | 2.72 (0, 0) | 42 | 0.68 (0.4, 0.96) | -1.9987 | NA |
| ｐ441 | 39 | 0.99 (0.87, 1.1) | 42 | 0.25 (0.19, 0.3) | -1.9995 | NA |
| ｐ1691 | 1 | 0.93 (0, 0) | 42 | 0.23 (0.19, 0.28) | -2.0128 | NA |
| ｐ5898 | 39 | 2.61 (1.59, 3.62) | 1 | 0.65 (0, 0) | -2.0128 | NA |
| ｐ8921 | 1 | 1.23 (0, 0) | 42 | 0.3 (0.18, 0.43) | -2.0232 | NA |
| ｐ3355 | 39 | 1.34 (1.09, 1.59) | 42 | 0.32 (0.26, 0.39) | -2.0465 | NA |
| ｐ9463 | 39 | 1.92 (0.86, 2.98) | 42 | 0.46 (0.36, 0.56) | -2.0550 | NA |
| ｐ3384 | 39 | 0.53 (0.35, 0.71) | 42 | 0.13 (0.09, 0.16) | -2.0696 | NA |
| ｐ9671 | 39 | 1.62 (1.25, 1.99) | 42 | 0.38 (0.32, 0.44) | -2.0967 | NA |
| ｐ1270 | 39 | 1.92 (0.89, 2.94) | 42 | 0.45 (0.33, 0.57) | -2.1019 | NA |
| ｐ1091 | 39 | 0.67 (0.24, 1.1) | 42 | 0.15 (0.03, 0.28) | -2.1172 | NA |
| ｐ7151 | 39 | 0.92 (0.44, 1.4) | 42 | 0.21 (0.15, 0.27) | -2.1266 | NA |
| ｐ4557 | 39 | 0.19 (0.08, 0.31) | 42 | 0.04 (-0.01, 0.1) | -2.1350 | NA |
| ｐ4398 | 1 | 1.07 (0, 0) | 42 | 0.24 (0.14, 0.34) | -2.1543 | NA |
| ｐ2192 | 1 | 5.95 (0, 0) | 42 | 1.3 (1.23, 1.36) | -2.1981 | NA |
| ｐ3231 | 39 | 0.54 (0.51, 0.58) | 42 | 0.12 (0.03, 0.2) | -2.2085 | NA |
| ｐ2134 | 1 | 1.69 (0, 0) | 42 | 0.36 (0.3, 0.43) | -2.2121 | NA |
| ｐ8694 | 39 | 1 (0.09, 1.92) | 42 | 0.22 (0.19, 0.24) | -2.2183 | NA |
| ｐ2336 | 39 | 0.91 (0.71, 1.12) | 42 | 0.19 (0.09, 0.29) | -2.2726 | NA |
| ｐ2767 | 39 | 3.85 (2.91, 4.78) | 1 | 0.76 (0, 0) | -2.3354 | NA |
| ｐ5635 | 1 | 1.05 (0, 0) | 42 | 0.2 (0.14, 0.26) | -2.3687 | NA |
| ｐ8687 | 1 | 1.3 (0, 0) | 42 | 0.25 (-0.63, 1.13) | -2.3742 | NA |
| ｐ1001 | 39 | 3.29 (0.93, 5.65) | 42 | 0.63 (0.31, 0.95) | -2.3848 | NA |
| ｐ7956 | 1 | 0.99 (0, 0) | 42 | 0.19 (0.16, 0.22) | -2.3952 | NA |
| ｐ1151 | 39 | 1.88 (1.81, 1.96) | 42 | 0.35 (0.27, 0.44) | -2.4143 | NA |
| ｐ6216 | 1 | 0.8 (0, 0) | 42 | 0.15 (0.1, 0.2) | -2.4229 | NA |
| ｐ9355 | 39 | 0.87 (0.74, 1.01) | 42 | 0.16 (0.14, 0.18) | -2.4301 | NA |
| ｐ4633 | 39 | 3.86 (1.74, 5.98) | 1 | 0.71 (0, 0) | -2.4384 | NA |
| ｐ4501 | 39 | 1.48 (1.12, 1.84) | 42 | 0.27 (0.2, 0.34) | -2.4447 | NA |
| ｐ2867 | 39 | 0.79 (0.5, 1.07) | 42 | 0.14 (0.07, 0.22) | -2.4460 | NA |
| ｐ3199 | 39 | 0.65 (0.54, 0.76) | 42 | 0.12 (0.03, 0.2) | -2.4840 | NA |
| ｐ5085 | 39 | 0.76 (0.51, 1.01) | 42 | 0.13 (0.08, 0.18) | -2.5222 | NA |
| ｐ2606 | 1 | 1.02 (0, 0) | 42 | 0.18 (0.1, 0.26) | -2.5274 | NA |
| ｐ6348 | 1 | 0.75 (0, 0) | 42 | 0.13 (0.1, 0.16) | -2.5410 | NA |
| ｐ6558 | 39 | 2.86 (1.72, 4.01) | 42 | 0.49 (0.27, 0.7) | -2.5578 | NA |
| ｐ4426 | 1 | 4.46 (0, 0) | 1 | 0.74 (0, 0) | -2.5918 | NA |
| ｐ7372 | 39 | 0.75 (0.71, 0.79) | 42 | 0.12 (0.11, 0.14) | -2.5968 | NA |
| ｐ4060 | 1 | 0.94 (0, 0) | 42 | 0.15 (0.14, 0.17) | -2.6073 | NA |
| ｐ3800 | 39 | 1.72 (0.48, 2.95) | 42 | 0.27 (0.19, 0.35) | -2.6494 | NA |
| ｐ2331 | 1 | 1.51 (0, 0) | 42 | 0.24 (0.05, 0.43) | -2.6508 | NA |
| ｐ5569 | 39 | 0.47 (0.39, 0.55) | 1 | 0.07 (0, 0) | -2.6857 | NA |
| ｐ8794 | 1 | 2.52 (0, 0) | 42 | 0.39 (0.3, 0.48) | -2.7059 | NA |
| ｐ616 | 1 | 1.66 (0, 0) | 42 | 0.24 (0.13, 0.35) | -2.7959 | NA |
| ｐ893 | 1 | 1.06 (0, 0) | 42 | 0.15 (0.08, 0.22) | -2.8144 | NA |
| ｐ4252 | 39 | 8.02 (5.38, 10.67) | 1 | 1.14 (0, 0) | -2.8196 | NA |
| ｐ493 | 39 | 2.08 (1.58, 2.57) | 42 | 0.29 (0.07, 0.51) | -2.8408 | NA |
| ｐ5801 | 39 | 0.38 (0.3, 0.47) | 42 | 0.05 (-0.02, 0.13) | -2.8668 | NA |
| ｐ1053 | 36 | 4.46 (0.35, 8.57) | 41 | 0.6 (0.37, 0.82) | -2.9008 | NA |
| ｐ8054 | 39 | 0.58 (0.45, 0.71) | 42 | 0.08 (0.04, 0.11) | -2.9091 | NA |
| ｐ1501 | 39 | 0.85 (0.75, 0.94) | 42 | 0.1 (0.01, 0.2) | -3.0314 | NA |
| ｐ1989 | 39 | 1.7 (1.36, 2.05) | 42 | 0.19 (0.06, 0.32) | -3.1606 | NA |
| ｐ661 | 39 | 0.94 (0.8, 1.08) | 42 | 0.1 (-0.03, 0.23) | -3.2172 | NA |
| ｐ9218 | 1 | 1.32 (0, 0) | 42 | 0.14 (0.11, 0.17) | -3.2347 | NA |
| ｐ6091 | 1 | 7.16 (0, 0) | 1 | 0.73 (0, 0) | -3.2955 | NA |
| ｐ7767 | 39 | 0.67 (0.44, 0.91) | 42 | 0.07 (-0.04, 0.17) | -3.3096 | NA |
| ｐ2648 | 39 | 0.77 (0.74, 0.8) | 42 | 0.07 (0.01, 0.14) | -3.3815 | NA |
| ｐ277 | 1 | 7.96 (0, 0) | 42 | 0.75 (0.67, 0.84) | -3.4007 | NA |
| ｐ1386 | 39 | 0.78 (0.63, 0.92) | 42 | 0.07 (-0.12, 0.26) | -3.4363 | NA |
| ｐ5519 | 39 | 0.69 (0.65, 0.73) | 42 | 0.06 (0.01, 0.11) | -3.5109 | NA |
| ｐ5080 | 33 | 2.87 (0.52, 5.23) | 42 | 0.24 (0.17, 0.32) | -3.5553 | NA |
| ｐ1269 | 39 | 2.45 (1.39, 3.5) | 42 | 0.2 (-0.78, 1.19) | -3.5894 | NA |
| ｐ6553 | 1 | 11.01 (0, 0) | 42 | 0.9 (-0.2, 2) | -3.6084 | NA |
| ｐ2501 | 1 | 2.39 (0, 0) | 42 | 0.18 (0.11, 0.25) | -3.7328 | NA |
| ｐ7667 | 39 | 4.44 (2.8, 6.08) | 42 | 0.33 (-0.03, 0.68) | -3.7666 | NA |
| ｐ6988 | 1 | 5.17 (0, 0) | 42 | 0.38 (0.29, 0.46) | -3.7831 | NA |
| ｐ479 | 39 | 7.47 (5.34, 9.6) | 42 | 0.43 (0.36, 0.5) | -4.1328 | NA |
| ｐ2218 | 39 | 1.38 (1.32, 1.45) | 42 | 0.08 (0.03, 0.12) | -4.1694 | NA |
| ｐ3719 | 39 | 0.84 (0.56, 1.13) | 42 | 0.03 (-0.01, 0.06) | -4.9692 | NA |
| ｐ4453 | 1 | 16.86 (0, 0) | 42 | 0.51 (0.5, 0.52) | -5.0567 | NA |
| ｐ3593 | 1 | 3.07 (0, 0) | 42 | 0.08 (0.02, 0.15) | -5.2242 | NA |
| ｐ1346 | 39 | 0.24 (0.05, 0.43) | 42 | 0 (-0.24, 0.24) | -6.8328 | NA |
| ｐ5072 | 39 | 0.53 (0.5, 0.56) | 42 | 0 (-0.05, 0.05) | -11.3896 | NA |
| ｐ498 | 39 | 0.41 (0.18, 0.63) | 42 | 0 (-0.06, 0.06) | NA | NA |
| ｐ2259 | 39 | 0 (-0.14, 0.14) | 42 | 0.57 (0.41, 0.74) | NA | NA |
| ｐ2652 | 39 | 0 (-0.06, 0.06) | 42 | 0.42 (0.26, 0.58) | NA | NA |
| ｐ4558 | 39 | 0 (-0.05, 0.05) | 42 | 0.45 (0.32, 0.57) | NA | NA |
| ｐ4670 | 39 | 0.35 (0.31, 0.4) | 42 | 0 (-0.32, 0.32) | NA | NA |
| ｐ5001 | 1 | 0.91 (0, 0) | 1 | 0 (0, 0) | NA | NA |
| ｐ5228 | 1 | 0 (0, 0) | 1 | 0.69 (0, 0) | NA | NA |
| ｐ5837 | 39 | 0 (-0.13, 0.13) | 42 | 0.73 (0.6, 0.86) | NA | NA |
| ｐ7025 | 39 | 0.15 (0.12, 0.18) | 1 | 0 (0, 0) | NA | NA |
| ｐ7668 | 39 | 0 (-0.37, 0.37) | 1 | 1.25 (0, 0) | NA | NA |
| ｐ8406 | 1 | 0 (0, 0) | 1 | 0.05 (0, 0) | NA | NA |
| ｐ10161 | 1 | 0.35 (0, 0) | 0 |  | NA | NA |
| ｐ10200 | 1 | 1.26 (0, 0) | 0 |  | NA | NA |
| ｐ10243 | 1 | 8.22 (0, 0) | 0 |  | NA | NA |
| ｐ10394 | 1 | 0.31 (0, 0) | 0 |  | NA | NA |
| ｐ10422 | 1 | 0.83 (0, 0) | 0 |  | NA | NA |
| ｐ10434 | 1 | 0.91 (0, 0) | 0 |  | NA | NA |
| ｐ10473 | 1 | 1.11 (0, 0) | 0 |  | NA | NA |
| ｐ10486 | 1 | 1.31 (0, 0) | 0 |  | NA | NA |
| ｐ10527 | 1 | 0.44 (0, 0) | 0 |  | NA | NA |
| ｐ10602 | 1 | 1.39 (0, 0) | 0 |  | NA | NA |
| ｐ10605 | 1 | 0.92 (0, 0) | 0 |  | NA | NA |
| ｐ10606 | 1 | 0.97 (0, 0) | 0 |  | NA | NA |
| ｐ10649 | 1 | 1.91 (0, 0) | 0 |  | NA | NA |
| ｐ10760 | 1 | 1.59 (0, 0) | 0 |  | NA | NA |
| ｐ10812 | 1 | 7.84 (0, 0) | 0 |  | NA | NA |
| ｐ10823 | 1 | 0.31 (0, 0) | 0 |  | NA | NA |
| ｐ10869 | 1 | 1.12 (0, 0) | 0 |  | NA | NA |
| ｐ10870 | 1 | 1.98 (0, 0) | 0 |  | NA | NA |
| ｐ10904 | 1 | 2.23 (0, 0) | 0 |  | NA | NA |
| ｐ10932 | 1 | 4.24 (0, 0) | 0 |  | NA | NA |
| ｐ10934 | 1 | 2.55 (0, 0) | 0 |  | NA | NA |
| ｐ10950 | 1 | 0.64 (0, 0) | 0 |  | NA | NA |
| ｐ10989 | 1 | 1.11 (0, 0) | 0 |  | NA | NA |
| ｐ11083 | 1 | 0.52 (0, 0) | 0 |  | NA | NA |
| ｐ11150 | 1 | 0.18 (0, 0) | 0 |  | NA | NA |
| ｐ11170 | 1 | 0.56 (0, 0) | 0 |  | NA | NA |
| ｐ11199 | 1 | 1.11 (0, 0) | 0 |  | NA | NA |
| ｐ11211 | 1 | 0.79 (0, 0) | 0 |  | NA | NA |
| ｐ11223 | 1 | 1.17 (0, 0) | 0 |  | NA | NA |
| ｐ11226 | 1 | 0.62 (0, 0) | 0 |  | NA | NA |
| ｐ11228 | 1 | 1.21 (0, 0) | 0 |  | NA | NA |
| ｐ11278 | 1 | 8.9 (0, 0) | 0 |  | NA | NA |
| ｐ11306 | 1 | 0.99 (0, 0) | 0 |  | NA | NA |
| ｐ11318 | 1 | 0.69 (0, 0) | 0 |  | NA | NA |
| ｐ11329 | 1 | 1.09 (0, 0) | 0 |  | NA | NA |
| ｐ11369 | 1 | 1.41 (0, 0) | 0 |  | NA | NA |
| ｐ11404 | 1 | 3.75 (0, 0) | 0 |  | NA | NA |
| ｐ11436 | 1 | 4.22 (0, 0) | 0 |  | NA | NA |
| ｐ11474 | 1 | 1.36 (0, 0) | 0 |  | NA | NA |
| ｐ11496 | 1 | 0.84 (0, 0) | 0 |  | NA | NA |
| ｐ11500 | 1 | 2.99 (0, 0) | 0 |  | NA | NA |
| ｐ11512 | 1 | 1.61 (0, 0) | 0 |  | NA | NA |
| ｐ11522 | 1 | 0 (0, 0) | 0 |  | NA | NA |
| ｐ11523 | 1 | 2.57 (0, 0) | 0 |  | NA | NA |
| ｐ11528 | 1 | 0.78 (0, 0) | 0 |  | NA | NA |
| ｐ11537 | 1 | 0 (0, 0) | 0 |  | NA | NA |
| ｐ11538 | 1 | 0 (0, 0) | 0 |  | NA | NA |
| ｐ11543 | 1 | 0.49 (0, 0) | 0 |  | NA | NA |
| ｐ11549 | 1 | 24.48 (0, 0) | 0 |  | NA | NA |
| ｐ11556 | 1 | 6.52 (0, 0) | 0 |  | NA | NA |
| ｐ11558 | 1 | 1.74 (0, 0) | 0 |  | NA | NA |
| ｐ11559 | 1 | 0.28 (0, 0) | 0 |  | NA | NA |
| ｐ11565 | 1 | 0.73 (0, 0) | 0 |  | NA | NA |
| ｐ11569 | 1 | 0.68 (0, 0) | 0 |  | NA | NA |
| ｐ11576 | 1 | 1.92 (0, 0) | 0 |  | NA | NA |
| ｐ11577 | 1 | 1.05 (0, 0) | 0 |  | NA | NA |
| ｐ11583 | 1 | 0.9 (0, 0) | 0 |  | NA | NA |
| ｐ11590 | 1 | 0 (0, 0) | 0 |  | NA | NA |
| ｐ11591 | 1 | 0.37 (0, 0) | 0 |  | NA | NA |
| ｐ11593 | 1 | 3.04 (0, 0) | 0 |  | NA | NA |
| ｐ11594 | 1 | 0 (0, 0) | 0 |  | NA | NA |
| ｐ11597 | 1 | 0.16 (0, 0) | 0 |  | NA | NA |
| ｐ11599 | 1 | 0 (0, 0) | 0 |  | NA | NA |
| ｐ11600 | 1 | 4.12 (0, 0) | 0 |  | NA | NA |
| ｐ11603 | 1 | 0.64 (0, 0) | 0 |  | NA | NA |
| ｐ11609 | 1 | 4.52 (0, 0) | 0 |  | NA | NA |
| ｐ11610 | 1 | 1.11 (0, 0) | 0 |  | NA | NA |
| ｐ11612 | 1 | 4.02 (0, 0) | 0 |  | NA | NA |
| ｐ11613 | 1 | 0.48 (0, 0) | 0 |  | NA | NA |
| ｐ11614 | 1 | 0.76 (0, 0) | 0 |  | NA | NA |
| ｐ11615 | 1 | 0.7 (0, 0) | 0 |  | NA | NA |
| ｐ11618 | 1 | 0.86 (0, 0) | 0 |  | NA | NA |
| ｐ11620 | 1 | 0 (0, 0) | 0 |  | NA | NA |
| ｐ11623 | 1 | 0 (0, 0) | 0 |  | NA | NA |
| ｐ11624 | 1 | 0.38 (0, 0) | 0 |  | NA | NA |
| ｐ11626 | 1 | 0.82 (0, 0) | 0 |  | NA | NA |
| ｐ11628 | 1 | 0 (0, 0) | 0 |  | NA | NA |
| ｐ11634 | 1 | 0.53 (0, 0) | 0 |  | NA | NA |
| ｐ11643 | 1 | 0.41 (0, 0) | 0 |  | NA | NA |
| ｐ11648 | 1 | 0 (0, 0) | 0 |  | NA | NA |
| ｐ11650 | 1 | 2.02 (0, 0) | 0 |  | NA | NA |
| ｐ11654 | 1 | 4.53 (0, 0) | 0 |  | NA | NA |
| ｐ11655 | 1 | 0.49 (0, 0) | 0 |  | NA | NA |
| ｐ11658 | 1 | 0.8 (0, 0) | 0 |  | NA | NA |
| ｐ11659 | 1 | 0.95 (0, 0) | 0 |  | NA | NA |
| ｐ11661 | 1 | 1.04 (0, 0) | 0 |  | NA | NA |
| ｐ11671 | 1 | 0.83 (0, 0) | 0 |  | NA | NA |
| ｐ11679 | 1 | 0.47 (0, 0) | 0 |  | NA | NA |
| ｐ11690 | 1 | 0.59 (0, 0) | 0 |  | NA | NA |
| ｐ11720 | 1 | 2.52 (0, 0) | 0 |  | NA | NA |
| ｐ11776 | 1 | 1.58 (0, 0) | 0 |  | NA | NA |
| ｐ11855 | 1 | 2.7 (0, 0) | 0 |  | NA | NA |
| ｐ11867 | 1 | 0.73 (0, 0) | 0 |  | NA | NA |
| ｐ11883 | 1 | 7.81 (0, 0) | 0 |  | NA | NA |
| ｐ11885 | 1 | 1.04 (0, 0) | 0 |  | NA | NA |
| ｐ11899 | 1 | 2.32 (0, 0) | 0 |  | NA | NA |
| ｐ11982 | 1 | 0.65 (0, 0) | 0 |  | NA | NA |
| ｐ11989 | 1 | 1.86 (0, 0) | 0 |  | NA | NA |
| ｐ11991 | 1 | 0.45 (0, 0) | 0 |  | NA | NA |
| ｐ12062 | 1 | 1.25 (0, 0) | 0 |  | NA | NA |
| ｐ12082 | 1 | 0.67 (0, 0) | 0 |  | NA | NA |
| ｐ12163 | 1 | 1.16 (0, 0) | 0 |  | NA | NA |
| ｐ12171 | 1 | 1.59 (0, 0) | 0 |  | NA | NA |
| ｐ12269 | 1 | 0.38 (0, 0) | 0 |  | NA | NA |
| ｐ12280 | 1 | 0.48 (0, 0) | 0 |  | NA | NA |
| ｐ12314 | 1 | 0.8 (0, 0) | 0 |  | NA | NA |
| ｐ12359 | 1 | 1 (0, 0) | 0 |  | NA | NA |
| ｐ12385 | 1 | 0.08 (0, 0) | 0 |  | NA | NA |
| ｐ12406 | 1 | 0.71 (0, 0) | 0 |  | NA | NA |
| ｐ12425 | 1 | 2.91 (0, 0) | 0 |  | NA | NA |
| ｐ12437 | 1 | 1.12 (0, 0) | 0 |  | NA | NA |
| ｐ12463 | 1 | 0.53 (0, 0) | 0 |  | NA | NA |
| ｐ12474 | 1 | 1.9 (0, 0) | 0 |  | NA | NA |
| ｐ12476 | 1 | 0.81 (0, 0) | 0 |  | NA | NA |
| ｐ12487 | 1 | 1.76 (0, 0) | 0 |  | NA | NA |
| ｐ12517 | 1 | 0.55 (0, 0) | 0 |  | NA | NA |
| ｐ12531 | 1 | 1.07 (0, 0) | 0 |  | NA | NA |
| ｐ12542 | 1 | 0.89 (0, 0) | 0 |  | NA | NA |
| ｐ12565 | 1 | 1.18 (0, 0) | 0 |  | NA | NA |
| ｐ12591 | 1 | 1.62 (0, 0) | 0 |  | NA | NA |
| ｐ12633 | 1 | 1.62 (0, 0) | 0 |  | NA | NA |
| ｐ12636 | 1 | 0.92 (0, 0) | 0 |  | NA | NA |
| ｐ12637 | 1 | 1.34 (0, 0) | 0 |  | NA | NA |
| ｐ12661 | 1 | 1.34 (0, 0) | 0 |  | NA | NA |
| ｐ12672 | 1 | 1.76 (0, 0) | 0 |  | NA | NA |
| ｐ12680 | 1 | 31.52 (0, 0) | 0 |  | NA | NA |
| ｐ12684 | 1 | 0 (0, 0) | 0 |  | NA | NA |
| ｐ12691 | 1 | 0 (0, 0) | 0 |  | NA | NA |
| ｐ12702 | 1 | 10.13 (0, 0) | 0 |  | NA | NA |
| ｐ12703 | 1 | 1.6 (0, 0) | 0 |  | NA | NA |
| ｐ12713 | 1 | 0.91 (0, 0) | 0 |  | NA | NA |
| ｐ12718 | 1 | 3.38 (0, 0) | 0 |  | NA | NA |
| ｐ12721 | 1 | 0.27 (0, 0) | 0 |  | NA | NA |
| ｐ12729 | 1 | 1.37 (0, 0) | 0 |  | NA | NA |
| ｐ12730 | 1 | 0.65 (0, 0) | 0 |  | NA | NA |
| ｐ12739 | 1 | 1.22 (0, 0) | 0 |  | NA | NA |
| ｐ12740 | 1 | 1.31 (0, 0) | 0 |  | NA | NA |
| ｐ12748 | 1 | 0.63 (0, 0) | 0 |  | NA | NA |
| ｐ12750 | 1 | 2.9 (0, 0) | 0 |  | NA | NA |
| ｐ12754 | 1 | 1 (0, 0) | 0 |  | NA | NA |
| ｐ12755 | 1 | 15.09 (0, 0) | 0 |  | NA | NA |
| ｐ12777 | 1 | 0.7 (0, 0) | 0 |  | NA | NA |
| ｐ12785 | 1 | 1.38 (0, 0) | 0 |  | NA | NA |
| ｐ12789 | 1 | 4.07 (0, 0) | 0 |  | NA | NA |
| ｐ12790 | 1 | 5.44 (0, 0) | 0 |  | NA | NA |
| ｐ12791 | 1 | 9.9 (0, 0) | 0 |  | NA | NA |
| ｐ12795 | 1 | 2.68 (0, 0) | 0 |  | NA | NA |
| ｐ12799 | 1 | 1.22 (0, 0) | 0 |  | NA | NA |
| ｐ12802 | 1 | 1.14 (0, 0) | 0 |  | NA | NA |
| ｐ12804 | 1 | 3.26 (0, 0) | 0 |  | NA | NA |
| ｐ12805 | 1 | 0.94 (0, 0) | 0 |  | NA | NA |
| ｐ12806 | 1 | 0.79 (0, 0) | 0 |  | NA | NA |
| ｐ12810 | 1 | 0.34 (0, 0) | 0 |  | NA | NA |
| ｐ12909 | 1 | 1 (0, 0) | 0 |  | NA | NA |
| ｐ12920 | 1 | 0 (0, 0) | 0 |  | NA | NA |
| ｐ12923 | 1 | 0.98 (0, 0) | 0 |  | NA | NA |
| ｐ12925 | 1 | 2.42 (0, 0) | 0 |  | NA | NA |
| ｐ12932 | 1 | 1.78 (0, 0) | 0 |  | NA | NA |
| ｐ12933 | 1 | 0.92 (0, 0) | 0 |  | NA | NA |
| ｐ12937 | 1 | 0.57 (0, 0) | 0 |  | NA | NA |
| ｐ12942 | 1 | 2.94 (0, 0) | 0 |  | NA | NA |
| ｐ12943 | 1 | 2.63 (0, 0) | 0 |  | NA | NA |
| ｐ12945 | 1 | 0 (0, 0) | 0 |  | NA | NA |
| ｐ12946 | 1 | 0.45 (0, 0) | 0 |  | NA | NA |
| ｐ12948 | 1 | 0.68 (0, 0) | 0 |  | NA | NA |
| ｐ12950 | 1 | 2.61 (0, 0) | 0 |  | NA | NA |
| ｐ12951 | 1 | 0.46 (0, 0) | 0 |  | NA | NA |
| ｐ12956 | 1 | 2.23 (0, 0) | 0 |  | NA | NA |
| ｐ12960 | 1 | 3.46 (0, 0) | 0 |  | NA | NA |
| ｐ12973 | 1 | 0.77 (0, 0) | 0 |  | NA | NA |
| ｐ12974 | 1 | 0.57 (0, 0) | 0 |  | NA | NA |
| ｐ12975 | 1 | 0.41 (0, 0) | 0 |  | NA | NA |
| ｐ12994 | 1 | 1.07 (0, 0) | 0 |  | NA | NA |
| ｐ13009 | 1 | 1.09 (0, 0) | 0 |  | NA | NA |
| ｐ13022 | 1 | 1.39 (0, 0) | 0 |  | NA | NA |
| ｐ13027 | 1 | 1.07 (0, 0) | 0 |  | NA | NA |
| ｐ13039 | 1 | 0.86 (0, 0) | 0 |  | NA | NA |
| ｐ13040 | 1 | 0.35 (0, 0) | 0 |  | NA | NA |
| ｐ13089 | 1 | 0.72 (0, 0) | 0 |  | NA | NA |
| ｐ13130 | 1 | 0 (0, 0) | 0 |  | NA | NA |
| ｐ13134 | 1 | 0.95 (0, 0) | 0 |  | NA | NA |
| ｐ13135 | 1 | 0.39 (0, 0) | 0 |  | NA | NA |
| ｐ13138 | 1 | 1.25 (0, 0) | 0 |  | NA | NA |
| ｐ13143 | 1 | 0.94 (0, 0) | 0 |  | NA | NA |
| ｐ13147 | 1 | 0 (0, 0) | 0 |  | NA | NA |
| ｐ13149 | 1 | 2.4 (0, 0) | 0 |  | NA | NA |
| ｐ13150 | 1 | 0 (0, 0) | 0 |  | NA | NA |
| ｐ13153 | 1 | 2.9 (0, 0) | 0 |  | NA | NA |
| ｐ13160 | 1 | 0 (0, 0) | 0 |  | NA | NA |
| ｐ13161 | 1 | 0.83 (0, 0) | 0 |  | NA | NA |
| ｐ13167 | 1 | 1.03 (0, 0) | 0 |  | NA | NA |
| ｐ13173 | 1 | 2.66 (0, 0) | 0 |  | NA | NA |
| ｐ13175 | 1 | 4.13 (0, 0) | 0 |  | NA | NA |
| ｐ13182 | 1 | 0.56 (0, 0) | 0 |  | NA | NA |
| ｐ13186 | 1 | 0.73 (0, 0) | 0 |  | NA | NA |
| ｐ13202 | 1 | 0 (0, 0) | 0 |  | NA | NA |
| ｐ13203 | 1 | 1.15 (0, 0) | 0 |  | NA | NA |
| ｐ13211 | 1 | 1.71 (0, 0) | 0 |  | NA | NA |
| ｐ13212 | 1 | 1.76 (0, 0) | 0 |  | NA | NA |
| ｐ13215 | 1 | 4.44 (0, 0) | 0 |  | NA | NA |
| ｐ13218 | 1 | 0.78 (0, 0) | 0 |  | NA | NA |
| ｐ13225 | 1 | 0.98 (0, 0) | 0 |  | NA | NA |
| ｐ13231 | 1 | 0.42 (0, 0) | 0 |  | NA | NA |
| ｐ13233 | 1 | 0.67 (0, 0) | 0 |  | NA | NA |
| ｐ13236 | 1 | 0.94 (0, 0) | 0 |  | NA | NA |
| ｐ13237 | 1 | 0.02 (0, 0) | 0 |  | NA | NA |
| ｐ13239 | 1 | 1.1 (0, 0) | 0 |  | NA | NA |
| ｐ13241 | 1 | 0.91 (0, 0) | 0 |  | NA | NA |
| ｐ13388 | 1 | 0.83 (0, 0) | 0 |  | NA | NA |
| ｐ13431 | 1 | 2.14 (0, 0) | 0 |  | NA | NA |
| ｐ13432 | 1 | 0.98 (0, 0) | 0 |  | NA | NA |
| ｐ13517 | 1 | 1.03 (0, 0) | 0 |  | NA | NA |
| ｐ13535 | 1 | 1.31 (0, 0) | 0 |  | NA | NA |
| ｐ13604 | 1 | 0.49 (0, 0) | 0 |  | NA | NA |
| ｐ13647 | 1 | 2.8 (0, 0) | 0 |  | NA | NA |
| ｐ13685 | 1 | 22.67 (0, 0) | 0 |  | NA | NA |
| ｐ13712 | 1 | 0 (0, 0) | 0 |  | NA | NA |
| ｐ13751 | 1 | 0.8 (0, 0) | 0 |  | NA | NA |
| ｐ13934 | 1 | 2.21 (0, 0) | 0 |  | NA | NA |
| ｐ13954 | 1 | 1.45 (0, 0) | 0 |  | NA | NA |
| ｐ13956 | 1 | 1.08 (0, 0) | 0 |  | NA | NA |
| ｐ13962 | 1 | 5.34 (0, 0) | 0 |  | NA | NA |
| ｐ13992 | 1 | 1.11 (0, 0) | 0 |  | NA | NA |
| ｐ14145 | 1 | 0.62 (0, 0) | 0 |  | NA | NA |
| ｐ14227 | 1 | 1.09 (0, 0) | 0 |  | NA | NA |
| ｐ14230 | 1 | 1.22 (0, 0) | 0 |  | NA | NA |
| ｐ14248 | 1 | 1.18 (0, 0) | 0 |  | NA | NA |
| ｐ14339 | 1 | 0.81 (0, 0) | 0 |  | NA | NA |
| ｐ14359 | 1 | 2.13 (0, 0) | 0 |  | NA | NA |
| ｐ14381 | 1 | 1.8 (0, 0) | 0 |  | NA | NA |
| ｐ14409 | 1 | 0.85 (0, 0) | 0 |  | NA | NA |
| ｐ14431 | 1 | 1.36 (0, 0) | 0 |  | NA | NA |
| ｐ14463 | 1 | 0.86 (0, 0) | 0 |  | NA | NA |
| ｐ14467 | 1 | 2.14 (0, 0) | 0 |  | NA | NA |
| ｐ14487 | 1 | 0.77 (0, 0) | 0 |  | NA | NA |
| ｐ14493 | 1 | 0.97 (0, 0) | 0 |  | NA | NA |
| ｐ14529 | 1 | 1.61 (0, 0) | 0 |  | NA | NA |
| ｐ14533 | 1 | 16.28 (0, 0) | 0 |  | NA | NA |
| ｐ14544 | 1 | 0.43 (0, 0) | 0 |  | NA | NA |
| ｐ14557 | 1 | 0.41 (0, 0) | 0 |  | NA | NA |
| ｐ14678 | 2 | 0 (0, 0) | 0 |  | NA | NA |
| ｐ14690 | 2 | 0 (0, 0) | 0 |  | NA | NA |
| ｐ14711 | 2 | 0 (0, 0) | 0 |  | NA | NA |
| ｐ14729 | 2 | 0 (0, 0) | 0 |  | NA | NA |
| ｐ14898 | 42 | 0 (0, 0) | 39 | 0 (0, 0) | NA | NA |
| ｐ14907 | 1 | 1.23 (0, 0) | 0 |  | NA | NA |
| ｐ14912 | 1 | 0.16 (0, 0) | 0 |  | NA | NA |
| ｐ14913 | 1 | 0.42 (0, 0) | 0 |  | NA | NA |
| ｐ14923 | 1 | 1.24 (0, 0) | 0 |  | NA | NA |
| ｐ14930 | 1 | 0.64 (0, 0) | 0 |  | NA | NA |
| ｐ14931 | 1 | 0.96 (0, 0) | 0 |  | NA | NA |
| ｐ14938 | 1 | 0.26 (0, 0) | 0 |  | NA | NA |
| ｐ14945 | 1 | 1.07 (0, 0) | 0 |  | NA | NA |
| ｐ14949 | 1 | 1.34 (0, 0) | 0 |  | NA | NA |
| ｐ14987 | 1 | 1.73 (0, 0) | 0 |  | NA | NA |
| ｐ14992 | 1 | 0.61 (0, 0) | 0 |  | NA | NA |
| ｐ14996 | 1 | 5.75 (0, 0) | 0 |  | NA | NA |
| ｐ14997 | 1 | 2.35 (0, 0) | 0 |  | NA | NA |
| ｐ15008 | 1 | 0.75 (0, 0) | 0 |  | NA | NA |
| ｐ15017 | 1 | 1.37 (0, 0) | 0 |  | NA | NA |
| ｐ15045 | 1 | 2.3 (0, 0) | 0 |  | NA | NA |
| ｐ15049 | 1 | 0.82 (0, 0) | 0 |  | NA | NA |
| ｐ15054 | 1 | 0.92 (0, 0) | 0 |  | NA | NA |
| ｐ15066 | 1 | 2.47 (0, 0) | 0 |  | NA | NA |
| ｐ15075 | 1 | 7.98 (0, 0) | 0 |  | NA | NA |
| ｐ15076 | 1 | 1.14 (0, 0) | 0 |  | NA | NA |
| ｐ15079 | 1 | 1.17 (0, 0) | 0 |  | NA | NA |
| ｐ15084 | 1 | 0.71 (0, 0) | 0 |  | NA | NA |
| ｐ15091 | 1 | 0.39 (0, 0) | 0 |  | NA | NA |
| ｐ15092 | 1 | 4.97 (0, 0) | 0 |  | NA | NA |
| ｐ15117 | 1 | 5.1 (0, 0) | 0 |  | NA | NA |
| ｐ15119 | 1 | 1.06 (0, 0) | 0 |  | NA | NA |
| ｐ15124 | 1 | 0.5 (0, 0) | 0 |  | NA | NA |
| ｐ15125 | 1 | 2.73 (0, 0) | 0 |  | NA | NA |
| ｐ15129 | 1 | 0.6 (0, 0) | 0 |  | NA | NA |
| ｐ15160 | 3 | 0 (0, 0) | 0 |  | NA | NA |
| ｐ15327 | 3 | 0 (0, 0) | 0 |  | NA | NA |
| ｐ15342 | 1 | 0.53 (0, 0) | 0 |  | NA | NA |
| ｐ15343 | 1 | 0.69 (0, 0) | 0 |  | NA | NA |
| ｐ15356 | 1 | 0.69 (0, 0) | 0 |  | NA | NA |
| ｐ15357 | 1 | 1.22 (0, 0) | 0 |  | NA | NA |
| ｐ15360 | 1 | 1.28 (0, 0) | 0 |  | NA | NA |
| ｐ15367 | 1 | 0.9 (0, 0) | 0 |  | NA | NA |
| ｐ15376 | 1 | 0.57 (0, 0) | 0 |  | NA | NA |
| ｐ15414 | 1 | 1.26 (0, 0) | 0 |  | NA | NA |
| ｐ15417 | 1 | 0.79 (0, 0) | 0 |  | NA | NA |
| ｐ15420 | 1 | 1.91 (0, 0) | 0 |  | NA | NA |
| ｐ15421 | 1 | 1.24 (0, 0) | 0 |  | NA | NA |
| ｐ15427 | 1 | 1.1 (0, 0) | 0 |  | NA | NA |
| ｐ15594 | 1 | 1.35 (0, 0) | 0 |  | NA | NA |
| ｐ15597 | 1 | 0.65 (0, 0) | 0 |  | NA | NA |
| ｐ15600 | 1 | 0.93 (0, 0) | 0 |  | NA | NA |
| ｐ15608 | 1 | 9.6 (0, 0) | 0 |  | NA | NA |
| ｐ15644 | 1 | 1.41 (0, 0) | 0 |  | NA | NA |
| ｐ15665 | 1 | 0.39 (0, 0) | 0 |  | NA | NA |
| ｐ15668 | 1 | 0.69 (0, 0) | 0 |  | NA | NA |
| ｐ15670 | 1 | 0.74 (0, 0) | 0 |  | NA | NA |
| ｐ15689 | 1 | 0.5 (0, 0) | 0 |  | NA | NA |
| ｐ15694 | 1 | 2.34 (0, 0) | 0 |  | NA | NA |
| ｐ15695 | 1 | 1.29 (0, 0) | 0 |  | NA | NA |
| ｐ15696 | 1 | 1.8 (0, 0) | 0 |  | NA | NA |
| ｐ15697 | 1 | 1.17 (0, 0) | 0 |  | NA | NA |
| ｐ15713 | 1 | 1.75 (0, 0) | 0 |  | NA | NA |
| ｐ15736 | 1 | 0.82 (0, 0) | 0 |  | NA | NA |
| ｐ15758 | 1 | 10.91 (0, 0) | 0 |  | NA | NA |
| ｐ15763 | 1 | 0.41 (0, 0) | 0 |  | NA | NA |
| ｐ15775 | 1 | 2.77 (0, 0) | 0 |  | NA | NA |
| ｐ15776 | 1 | 0.44 (0, 0) | 0 |  | NA | NA |
| ｐ15777 | 1 | 0.86 (0, 0) | 0 |  | NA | NA |
| ｐ15787 | 1 | 1.73 (0, 0) | 0 |  | NA | NA |
| ｐ15788 | 1 | 1.39 (0, 0) | 0 |  | NA | NA |
| ｐ15789 | 1 | 1.14 (0, 0) | 0 |  | NA | NA |
| ｐ15791 | 1 | 4.74 (0, 0) | 0 |  | NA | NA |
| ｐ15796 | 1 | 0.8 (0, 0) | 0 |  | NA | NA |
| ｐ15797 | 1 | 2.02 (0, 0) | 0 |  | NA | NA |
| ｐ15804 | 1 | 2.45 (0, 0) | 0 |  | NA | NA |
| ｐ15809 | 1 | 0.17 (0, 0) | 0 |  | NA | NA |
| ｐ15812 | 1 | 0.72 (0, 0) | 0 |  | NA | NA |
| ｐ15815 | 1 | 0.8 (0, 0) | 0 |  | NA | NA |
| ｐ15817 | 1 | 0.7 (0, 0) | 0 |  | NA | NA |
| ｐ15860 | 1 | 0.14 (0, 0) | 0 |  | NA | NA |
| ｐ15873 | 1 | 4.44 (0, 0) | 0 |  | NA | NA |
| ｐ15881 | 1 | 0.67 (0, 0) | 0 |  | NA | NA |
| ｐ15896 | 1 | 0.31 (0, 0) | 0 |  | NA | NA |
| ｐ15904 | 1 | 2.69 (0, 0) | 0 |  | NA | NA |
| ｐ15916 | 1 | 0.94 (0, 0) | 0 |  | NA | NA |
| ｐ15950 | 1 | 4.59 (0, 0) | 0 |  | NA | NA |
| ｐ15962 | 1 | 0.93 (0, 0) | 0 |  | NA | NA |
| ｐ15967 | 1 | 0.3 (0, 0) | 0 |  | NA | NA |
| ｐ15990 | 1 | 1.13 (0, 0) | 0 |  | NA | NA |
| ｐ15999 | 1 | 1.23 (0, 0) | 0 |  | NA | NA |
| ｐ16013 | 1 | 1.01 (0, 0) | 0 |  | NA | NA |
| ｐ16022 | 1 | 1.23 (0, 0) | 0 |  | NA | NA |
| ｐ16043 | 1 | 1.03 (0, 0) | 0 |  | NA | NA |
| ｐ16047 | 1 | 5.56 (0, 0) | 0 |  | NA | NA |
| ｐ16050 | 1 | 1.41 (0, 0) | 0 |  | NA | NA |
| ｐ16051 | 1 | 0.31 (0, 0) | 0 |  | NA | NA |
| ｐ16053 | 1 | 0.9 (0, 0) | 0 |  | NA | NA |
| ｐ16054 | 1 | 0.4 (0, 0) | 0 |  | NA | NA |
| ｐ16065 | 1 | 0.79 (0, 0) | 0 |  | NA | NA |
| ｐ16069 | 1 | 0.89 (0, 0) | 0 |  | NA | NA |
| ｐ16071 | 1 | 0.17 (0, 0) | 0 |  | NA | NA |
| ｐ16076 | 1 | 0.86 (0, 0) | 0 |  | NA | NA |
| ｐ16082 | 1 | 1.15 (0, 0) | 0 |  | NA | NA |
| ｐ16101 | 1 | 0.75 (0, 0) | 0 |  | NA | NA |
| ｐ16118 | 1 | 0.62 (0, 0) | 0 |  | NA | NA |
| ｐ16139 | 1 | 1.07 (0, 0) | 0 |  | NA | NA |
| ｐ16162 | 1 | 1.25 (0, 0) | 0 |  | NA | NA |
| ｐ16199 | 1 | 3.01 (0, 0) | 0 |  | NA | NA |
| ｐ16200 | 1 | 0.95 (0, 0) | 0 |  | NA | NA |
| ｐ16232 | 1 | 0.97 (0, 0) | 0 |  | NA | NA |
| ｐ16241 | 1 | 1.71 (0, 0) | 0 |  | NA | NA |
| ｐ16274 | 1 | 0.41 (0, 0) | 0 |  | NA | NA |
| ｐ16291 | 1 | 0.59 (0, 0) | 0 |  | NA | NA |
| ｐ16296 | 1 | 6.45 (0, 0) | 0 |  | NA | NA |
| ｐ16312 | 1 | 5.11 (0, 0) | 0 |  | NA | NA |
| ｐ16326 | 1 | 0.32 (0, 0) | 0 |  | NA | NA |
| ｐ16343 | 1 | 5.57 (0, 0) | 0 |  | NA | NA |
| ｐ16349 | 1 | 1.26 (0, 0) | 0 |  | NA | NA |
| ｐ16351 | 1 | 0.27 (0, 0) | 0 |  | NA | NA |
| ｐ16352 | 1 | 0.39 (0, 0) | 0 |  | NA | NA |
| ｐ16359 | 1 | 1.07 (0, 0) | 0 |  | NA | NA |
| ｐ16373 | 1 | 2.29 (0, 0) | 0 |  | NA | NA |
| ｐ16393 | 1 | 0.89 (0, 0) | 0 |  | NA | NA |
| ｐ16408 | 1 | 2.94 (0, 0) | 0 |  | NA | NA |
| ｐ16415 | 1 | 1.32 (0, 0) | 0 |  | NA | NA |
| ｐ16416 | 1 | 1.51 (0, 0) | 0 |  | NA | NA |
| ｐ16420 | 1 | 0.87 (0, 0) | 0 |  | NA | NA |
| ｐ16423 | 1 | 1.41 (0, 0) | 0 |  | NA | NA |
| ｐ16424 | 1 | 0.82 (0, 0) | 0 |  | NA | NA |
| ｐ16426 | 1 | 0.65 (0, 0) | 0 |  | NA | NA |
| ｐ16429 | 1 | 0.51 (0, 0) | 0 |  | NA | NA |
| ｐ16457 | 1 | 1.77 (0, 0) | 0 |  | NA | NA |
| ｐ16471 | 1 | 0.53 (0, 0) | 0 |  | NA | NA |
| ｐ16474 | 1 | 0.37 (0, 0) | 0 |  | NA | NA |
| ｐ16476 | 1 | 0.53 (0, 0) | 0 |  | NA | NA |
| ｐ16521 | 1 | 1.08 (0, 0) | 0 |  | NA | NA |
| ｐ16567 | 1 | 0.99 (0, 0) | 0 |  | NA | NA |
| ｐ16576 | 1 | 0 (0, 0) | 0 |  | NA | NA |
| ｐ16578 | 1 | 0.1 (0, 0) | 0 |  | NA | NA |
| ｐ16579 | 1 | 0 (0, 0) | 0 |  | NA | NA |
| ｐ16581 | 1 | 0.16 (0, 0) | 0 |  | NA | NA |
| ｐ16583 | 1 | 2.13 (0, 0) | 0 |  | NA | NA |
| ｐ16584 | 1 | 1.64 (0, 0) | 0 |  | NA | NA |
| ｐ16585 | 1 | 0.64 (0, 0) | 0 |  | NA | NA |
| ｐ16588 | 1 | 1.44 (0, 0) | 0 |  | NA | NA |
| ｐ16589 | 1 | 0 (0, 0) | 0 |  | NA | NA |
| ｐ16590 | 1 | 4.58 (0, 0) | 0 |  | NA | NA |
| ｐ16593 | 1 | 0.55 (0, 0) | 0 |  | NA | NA |
| ｐ16598 | 1 | 1.92 (0, 0) | 0 |  | NA | NA |
| ｐ16599 | 1 | 2.34 (0, 0) | 0 |  | NA | NA |
| ｐ16602 | 1 | 1.99 (0, 0) | 0 |  | NA | NA |
| ｐ16605 | 1 | 1.94 (0, 0) | 0 |  | NA | NA |
| ｐ16608 | 1 | 8.4 (0, 0) | 0 |  | NA | NA |
| ｐ16616 | 1 | 0.78 (0, 0) | 0 |  | NA | NA |
| ｐ16617 | 1 | 2.7 (0, 0) | 0 |  | NA | NA |
| ｐ16618 | 1 | 0 (0, 0) | 0 |  | NA | NA |
| ｐ16620 | 1 | 2.69 (0, 0) | 0 |  | NA | NA |
| ｐ16621 | 1 | 0.31 (0, 0) | 0 |  | NA | NA |
| ｐ16626 | 1 | 1.96 (0, 0) | 0 |  | NA | NA |
| ｐ16628 | 1 | 1.52 (0, 0) | 0 |  | NA | NA |
| ｐ16629 | 1 | 1.76 (0, 0) | 0 |  | NA | NA |
| ｐ16631 | 1 | 0.42 (0, 0) | 0 |  | NA | NA |
| ｐ16634 | 1 | 2.24 (0, 0) | 0 |  | NA | NA |
| ｐ16658 | 1 | 0.63 (0, 0) | 0 |  | NA | NA |
| ｐ16665 | 1 | 1.37 (0, 0) | 0 |  | NA | NA |
| ｐ16669 | 1 | 0.57 (0, 0) | 0 |  | NA | NA |
| ｐ16693 | 1 | 0.74 (0, 0) | 0 |  | NA | NA |
| ｐ16701 | 1 | 1.4 (0, 0) | 0 |  | NA | NA |
| ｐ16702 | 1 | 0.61 (0, 0) | 0 |  | NA | NA |
| ｐ16732 | 1 | 1.1 (0, 0) | 0 |  | NA | NA |
| ｐ16750 | 1 | 0.78 (0, 0) | 0 |  | NA | NA |
| ｐ16751 | 1 | 2 (0, 0) | 0 |  | NA | NA |
| ｐ16781 | 1 | 0.62 (0, 0) | 0 |  | NA | NA |
| ｐ16804 | 1 | 0.78 (0, 0) | 0 |  | NA | NA |
| ｐ16812 | 1 | 1.01 (0, 0) | 0 |  | NA | NA |
| ｐ16822 | 1 | 0.71 (0, 0) | 0 |  | NA | NA |
| ｐ16871 | 1 | 1.09 (0, 0) | 0 |  | NA | NA |
| ｐ16891 | 1 | 0.76 (0, 0) | 0 |  | NA | NA |
| ｐ16903 | 1 | 1.08 (0, 0) | 0 |  | NA | NA |
| ｐ16920 | 1 | 1.23 (0, 0) | 0 |  | NA | NA |
| ｐ16931 | 1 | 1.01 (0, 0) | 0 |  | NA | NA |
| ｐ16940 | 1 | 2.29 (0, 0) | 0 |  | NA | NA |
| ｐ16949 | 1 | 0.62 (0, 0) | 0 |  | NA | NA |
| ｐ16953 | 1 | 0.38 (0, 0) | 0 |  | NA | NA |
| ｐ16960 | 1 | 1.29 (0, 0) | 0 |  | NA | NA |
| ｐ16961 | 1 | 1.05 (0, 0) | 0 |  | NA | NA |
| ｐ16962 | 1 | 1.5 (0, 0) | 0 |  | NA | NA |
| ｐ16963 | 1 | 1.11 (0, 0) | 0 |  | NA | NA |
| ｐ16965 | 1 | 1.19 (0, 0) | 0 |  | NA | NA |
| ｐ16966 | 1 | 0.97 (0, 0) | 0 |  | NA | NA |
| ｐ16967 | 1 | 2.91 (0, 0) | 0 |  | NA | NA |
| ｐ16968 | 1 | 0.78 (0, 0) | 0 |  | NA | NA |
| ｐ16969 | 1 | 2.12 (0, 0) | 0 |  | NA | NA |
| ｐ16970 | 1 | 3.36 (0, 0) | 0 |  | NA | NA |
| ｐ16971 | 1 | 2.12 (0, 0) | 0 |  | NA | NA |
| ｐ16975 | 1 | 1.35 (0, 0) | 0 |  | NA | NA |
| ｐ16976 | 1 | 0.7 (0, 0) | 0 |  | NA | NA |
| ｐ16977 | 1 | 0.52 (0, 0) | 0 |  | NA | NA |
| ｐ16978 | 1 | 0.8 (0, 0) | 0 |  | NA | NA |
| ｐ16979 | 1 | 0.7 (0, 0) | 0 |  | NA | NA |
| ｐ16980 | 1 | 1.12 (0, 0) | 0 |  | NA | NA |
| ｐ16981 | 1 | 1 (0, 0) | 0 |  | NA | NA |
| ｐ16985 | 1 | 2.87 (0, 0) | 0 |  | NA | NA |
| ｐ16986 | 1 | 0.91 (0, 0) | 0 |  | NA | NA |
| ｐ16987 | 1 | 1.96 (0, 0) | 0 |  | NA | NA |
| ｐ16989 | 1 | 2.43 (0, 0) | 0 |  | NA | NA |
| ｐ16991 | 1 | 2.41 (0, 0) | 0 |  | NA | NA |
| ｐ16993 | 1 | 1.24 (0, 0) | 0 |  | NA | NA |
| ｐ16994 | 1 | 4.4 (0, 0) | 0 |  | NA | NA |
| ｐ16996 | 1 | 0.95 (0, 0) | 0 |  | NA | NA |
| ｐ16997 | 1 | 0.98 (0, 0) | 0 |  | NA | NA |
| ｐ16998 | 1 | 0.67 (0, 0) | 0 |  | NA | NA |
| ｐ17003 | 1 | 0.82 (0, 0) | 0 |  | NA | NA |
| ｐ17004 | 1 | 0.73 (0, 0) | 0 |  | NA | NA |
| ｐ17006 | 1 | 0.59 (0, 0) | 0 |  | NA | NA |
| ｐ17007 | 1 | 1.05 (0, 0) | 0 |  | NA | NA |
| ｐ17008 | 1 | 0.98 (0, 0) | 0 |  | NA | NA |
| ｐ17009 | 1 | 0.85 (0, 0) | 0 |  | NA | NA |
| ｐ17011 | 1 | 1.54 (0, 0) | 0 |  | NA | NA |
| ｐ17012 | 1 | 0.78 (0, 0) | 0 |  | NA | NA |
| ｐ17014 | 1 | 0.77 (0, 0) | 0 |  | NA | NA |
| ｐ17017 | 1 | 0.51 (0, 0) | 0 |  | NA | NA |
| ｐ17018 | 1 | 1.02 (0, 0) | 0 |  | NA | NA |
| ｐ17019 | 1 | 1 (0, 0) | 0 |  | NA | NA |
| ｐ17020 | 1 | 0.96 (0, 0) | 0 |  | NA | NA |
| ｐ17021 | 1 | 0.98 (0, 0) | 0 |  | NA | NA |
| ｐ17022 | 1 | 0.88 (0, 0) | 0 |  | NA | NA |
| ｐ17023 | 1 | 1.38 (0, 0) | 0 |  | NA | NA |
| ｐ17024 | 1 | 0.8 (0, 0) | 0 |  | NA | NA |
| ｐ17025 | 1 | 0.9 (0, 0) | 0 |  | NA | NA |
| ｐ17027 | 1 | 0.33 (0, 0) | 0 |  | NA | NA |
| ｐ17041 | 1 | 1.26 (0, 0) | 0 |  | NA | NA |
| ｐ17046 | 1 | 5.1 (0, 0) | 0 |  | NA | NA |
| ｐ17053 | 1 | 1.33 (0, 0) | 0 |  | NA | NA |
| ｐ17056 | 1 | 0.72 (0, 0) | 0 |  | NA | NA |
| ｐ17068 | 1 | 0.93 (0, 0) | 0 |  | NA | NA |
| ｐ17071 | 1 | 0.36 (0, 0) | 0 |  | NA | NA |
| ｐ17085 | 1 | 0.87 (0, 0) | 0 |  | NA | NA |
| ｐ17089 | 1 | 0.39 (0, 0) | 0 |  | NA | NA |
| ｐ17095 | 1 | 1.3 (0, 0) | 0 |  | NA | NA |
| ｐ17106 | 1 | 0.81 (0, 0) | 0 |  | NA | NA |
| ｐ17113 | 1 | 1.19 (0, 0) | 0 |  | NA | NA |
| ｐ17129 | 1 | 1.07 (0, 0) | 0 |  | NA | NA |
| ｐ17131 | 1 | 1.12 (0, 0) | 0 |  | NA | NA |
| ｐ17132 | 1 | 0.98 (0, 0) | 0 |  | NA | NA |
| ｐ17139 | 1 | 0.51 (0, 0) | 0 |  | NA | NA |
| ｐ17143 | 1 | 2.97 (0, 0) | 0 |  | NA | NA |
| ｐ17144 | 1 | 0.75 (0, 0) | 0 |  | NA | NA |
| ｐ17152 | 1 | 0.68 (0, 0) | 0 |  | NA | NA |
| ｐ17153 | 1 | 1.12 (0, 0) | 0 |  | NA | NA |
| ｐ17154 | 1 | 0 (0, 0) | 0 |  | NA | NA |
| ｐ17156 | 1 | 0.21 (0, 0) | 0 |  | NA | NA |
| ｐ17157 | 1 | 0.11 (0, 0) | 0 |  | NA | NA |
| ｐ17159 | 1 | 0 (0, 0) | 0 |  | NA | NA |
| ｐ17160 | 1 | 1.39 (0, 0) | 0 |  | NA | NA |
| ｐ17161 | 1 | 0.27 (0, 0) | 0 |  | NA | NA |
| ｐ17162 | 1 | 1.12 (0, 0) | 0 |  | NA | NA |
| ｐ17164 | 1 | 1.75 (0, 0) | 0 |  | NA | NA |
| ｐ17165 | 1 | 1.38 (0, 0) | 0 |  | NA | NA |
| ｐ17167 | 1 | 0.49 (0, 0) | 0 |  | NA | NA |
| ｐ17168 | 1 | 0.09 (0, 0) | 0 |  | NA | NA |
| ｐ17169 | 1 | 0.98 (0, 0) | 0 |  | NA | NA |
| ｐ17170 | 1 | 0.42 (0, 0) | 0 |  | NA | NA |
| ｐ17171 | 1 | 1.02 (0, 0) | 0 |  | NA | NA |
| ｐ17172 | 1 | 6.49 (0, 0) | 0 |  | NA | NA |
| ｐ17173 | 1 | 1.33 (0, 0) | 0 |  | NA | NA |
| ｐ17175 | 1 | 1.65 (0, 0) | 0 |  | NA | NA |
| ｐ17176 | 1 | 0.44 (0, 0) | 0 |  | NA | NA |
| ｐ17178 | 1 | 0 (0, 0) | 0 |  | NA | NA |
| ｐ17179 | 1 | 0.32 (0, 0) | 0 |  | NA | NA |
| ｐ17180 | 1 | 0.87 (0, 0) | 0 |  | NA | NA |
| ｐ17183 | 1 | 0.31 (0, 0) | 0 |  | NA | NA |
| ｐ17184 | 1 | 1.57 (0, 0) | 0 |  | NA | NA |
| ｐ17185 | 1 | 2.92 (0, 0) | 0 |  | NA | NA |
| ｐ17186 | 1 | 0.57 (0, 0) | 0 |  | NA | NA |
| ｐ17189 | 1 | 1.95 (0, 0) | 0 |  | NA | NA |
| ｐ17191 | 1 | 1.23 (0, 0) | 0 |  | NA | NA |
| ｐ17193 | 1 | 2.43 (0, 0) | 0 |  | NA | NA |
| ｐ17194 | 1 | 2.81 (0, 0) | 0 |  | NA | NA |
| ｐ17196 | 1 | 1.36 (0, 0) | 0 |  | NA | NA |
| ｐ17198 | 1 | 2.31 (0, 0) | 0 |  | NA | NA |
| ｐ17199 | 1 | 4.98 (0, 0) | 0 |  | NA | NA |
| ｐ17200 | 1 | 0.59 (0, 0) | 0 |  | NA | NA |
| ｐ17202 | 1 | 1.04 (0, 0) | 0 |  | NA | NA |
| ｐ17203 | 1 | 0.38 (0, 0) | 0 |  | NA | NA |
| ｐ17204 | 1 | 0.9 (0, 0) | 0 |  | NA | NA |
| ｐ17205 | 1 | 0.89 (0, 0) | 0 |  | NA | NA |
| ｐ17206 | 1 | 1.18 (0, 0) | 0 |  | NA | NA |
| ｐ17207 | 1 | 1.05 (0, 0) | 0 |  | NA | NA |
| ｐ17209 | 1 | 0.34 (0, 0) | 0 |  | NA | NA |
| ｐ17211 | 1 | 1.09 (0, 0) | 0 |  | NA | NA |
| ｐ17212 | 1 | 1.61 (0, 0) | 0 |  | NA | NA |
| ｐ17213 | 1 | 0.74 (0, 0) | 0 |  | NA | NA |
| ｐ17214 | 1 | 1.94 (0, 0) | 0 |  | NA | NA |
| ｐ17215 | 1 | 1.87 (0, 0) | 0 |  | NA | NA |
| ｐ17218 | 1 | 0.29 (0, 0) | 0 |  | NA | NA |
| ｐ17219 | 1 | 1.41 (0, 0) | 0 |  | NA | NA |
| ｐ17221 | 1 | 0.77 (0, 0) | 0 |  | NA | NA |
| ｐ17223 | 1 | 0 (0, 0) | 0 |  | NA | NA |
| ｐ17236 | 1 | 0.93 (0, 0) | 0 |  | NA | NA |
| ｐ17243 | 1 | 0.65 (0, 0) | 0 |  | NA | NA |
| ｐ17245 | 1 | 0.84 (0, 0) | 0 |  | NA | NA |
| ｐ17255 | 1 | 0.71 (0, 0) | 0 |  | NA | NA |
| ｐ17262 | 1 | 1.67 (0, 0) | 0 |  | NA | NA |
| ｐ17274 | 1 | 0.78 (0, 0) | 0 |  | NA | NA |
| ｐ17280 | 1 | 1.21 (0, 0) | 0 |  | NA | NA |
| ｐ17286 | 1 | 4.76 (0, 0) | 0 |  | NA | NA |
| ｐ17287 | 1 | 0.89 (0, 0) | 0 |  | NA | NA |
| ｐ17292 | 1 | 1.12 (0, 0) | 0 |  | NA | NA |
| ｐ17295 | 1 | 0.56 (0, 0) | 0 |  | NA | NA |
| ｐ17315 | 1 | 1.24 (0, 0) | 0 |  | NA | NA |
| ｐ17327 | 1 | 0.5 (0, 0) | 0 |  | NA | NA |
| ｐ17343 | 1 | 0.96 (0, 0) | 0 |  | NA | NA |
| ｐ17344 | 1 | 0.66 (0, 0) | 0 |  | NA | NA |
| ｐ17373 | 1 | 0.52 (0, 0) | 0 |  | NA | NA |
| ｐ17388 | 1 | 1.18 (0, 0) | 0 |  | NA | NA |
| ｐ17389 | 1 | 0.5 (0, 0) | 0 |  | NA | NA |
| ｐ17402 | 1 | 0.71 (0, 0) | 0 |  | NA | NA |
| ｐ17409 | 1 | 3.85 (0, 0) | 0 |  | NA | NA |
| ｐ17414 | 1 | 0.99 (0, 0) | 0 |  | NA | NA |
| ｐ17435 | 1 | 1.7 (0, 0) | 0 |  | NA | NA |
| ｐ17441 | 1 | 2.16 (0, 0) | 0 |  | NA | NA |
| ｐ17458 | 1 | 0 (0, 0) | 0 |  | NA | NA |
| ｐ17477 | 1 | 0.78 (0, 0) | 0 |  | NA | NA |
| ｐ17489 | 1 | 1.09 (0, 0) | 0 |  | NA | NA |
| ｐ17498 | 1 | 0.65 (0, 0) | 0 |  | NA | NA |
| ｐ17511 | 1 | 0.65 (0, 0) | 0 |  | NA | NA |
| ｐ17523 | 1 | 0.65 (0, 0) | 0 |  | NA | NA |
| ｐ17532 | 1 | 0.91 (0, 0) | 0 |  | NA | NA |
| ｐ17542 | 1 | 0.76 (0, 0) | 0 |  | NA | NA |
| ｐ17555 | 1 | 0.64 (0, 0) | 0 |  | NA | NA |
| ｐ17564 | 1 | 1.03 (0, 0) | 0 |  | NA | NA |
| ｐ17578 | 1 | 1.11 (0, 0) | 0 |  | NA | NA |
| ｐ17581 | 1 | 0.62 (0, 0) | 0 |  | NA | NA |
| ｐ17589 | 1 | 0.42 (0, 0) | 0 |  | NA | NA |
| ｐ17591 | 1 | 4.98 (0, 0) | 0 |  | NA | NA |
| ｐ17592 | 1 | 0.99 (0, 0) | 0 |  | NA | NA |
| ｐ17600 | 1 | 2.14 (0, 0) | 0 |  | NA | NA |
| ｐ17631 | 1 | 1.23 (0, 0) | 0 |  | NA | NA |
| ｐ17650 | 1 | 0.91 (0, 0) | 0 |  | NA | NA |
| ｐ17675 | 1 | 1.86 (0, 0) | 0 |  | NA | NA |
| ｐ17690 | 1 | 1.88 (0, 0) | 0 |  | NA | NA |
| ｐ17695 | 1 | 1.86 (0, 0) | 0 |  | NA | NA |
| ｐ17698 | 1 | 13.85 (0, 0) | 0 |  | NA | NA |
| ｐ17709 | 1 | 0.49 (0, 0) | 0 |  | NA | NA |
| ｐ17722 | 1 | 1.13 (0, 0) | 0 |  | NA | NA |
| ｐ17726 | 1 | 1 (0, 0) | 0 |  | NA | NA |
| ｐ17727 | 1 | 1.18 (0, 0) | 0 |  | NA | NA |
| ｐ17729 | 1 | 2.34 (0, 0) | 0 |  | NA | NA |
| ｐ17731 | 1 | 1.5 (0, 0) | 0 |  | NA | NA |
| ｐ17734 | 1 | 0.79 (0, 0) | 0 |  | NA | NA |
| ｐ17735 | 1 | 0.97 (0, 0) | 0 |  | NA | NA |
| ｐ17736 | 1 | 1.05 (0, 0) | 0 |  | NA | NA |
| ｐ17738 | 1 | 1.25 (0, 0) | 0 |  | NA | NA |
| ｐ17739 | 1 | 1.05 (0, 0) | 0 |  | NA | NA |
| ｐ17742 | 1 | 0.45 (0, 0) | 0 |  | NA | NA |
| ｐ17745 | 1 | 0.63 (0, 0) | 0 |  | NA | NA |
| ｐ17746 | 1 | 1.08 (0, 0) | 0 |  | NA | NA |
| ｐ17747 | 1 | 2.05 (0, 0) | 0 |  | NA | NA |
| ｐ17748 | 1 | 0.83 (0, 0) | 0 |  | NA | NA |
| ｐ17750 | 1 | 1.4 (0, 0) | 0 |  | NA | NA |
| ｐ17752 | 1 | 0.78 (0, 0) | 0 |  | NA | NA |
| ｐ17753 | 1 | 0.72 (0, 0) | 0 |  | NA | NA |
| ｐ17756 | 1 | 1.25 (0, 0) | 0 |  | NA | NA |
| ｐ17757 | 1 | 1.62 (0, 0) | 0 |  | NA | NA |
| ｐ17758 | 1 | 0.56 (0, 0) | 0 |  | NA | NA |
| ｐ17760 | 1 | 0.7 (0, 0) | 0 |  | NA | NA |
| ｐ17761 | 1 | 1.38 (0, 0) | 0 |  | NA | NA |
| ｐ17762 | 1 | 0.21 (0, 0) | 0 |  | NA | NA |
| ｐ17763 | 1 | 0.75 (0, 0) | 0 |  | NA | NA |
| ｐ17764 | 1 | 1.1 (0, 0) | 0 |  | NA | NA |
| ｐ17766 | 1 | 1.02 (0, 0) | 0 |  | NA | NA |
| ｐ17769 | 1 | 0.85 (0, 0) | 0 |  | NA | NA |
| ｐ17770 | 1 | 1.26 (0, 0) | 0 |  | NA | NA |
| ｐ17771 | 1 | 4.16 (0, 0) | 0 |  | NA | NA |
| ｐ17772 | 1 | 0.7 (0, 0) | 0 |  | NA | NA |
| ｐ17773 | 1 | 0.63 (0, 0) | 0 |  | NA | NA |
| ｐ17775 | 1 | 0.51 (0, 0) | 0 |  | NA | NA |
| ｐ17776 | 1 | 0.64 (0, 0) | 0 |  | NA | NA |
| ｐ17778 | 1 | 0.61 (0, 0) | 0 |  | NA | NA |
| ｐ17779 | 1 | 0.84 (0, 0) | 0 |  | NA | NA |
| ｐ17780 | 1 | 0.04 (0, 0) | 0 |  | NA | NA |
| ｐ17785 | 1 | 0.66 (0, 0) | 0 |  | NA | NA |
| ｐ17788 | 1 | 0.54 (0, 0) | 0 |  | NA | NA |
| ｐ17789 | 1 | 0.54 (0, 0) | 0 |  | NA | NA |
| ｐ17790 | 1 | 0.67 (0, 0) | 0 |  | NA | NA |
| ｐ17791 | 1 | 1.53 (0, 0) | 0 |  | NA | NA |
| ｐ17792 | 1 | 0.54 (0, 0) | 0 |  | NA | NA |
| ｐ17795 | 1 | 1.65 (0, 0) | 0 |  | NA | NA |
| ｐ17796 | 1 | 0 (0, 0) | 0 |  | NA | NA |
| ｐ17797 | 1 | 10.58 (0, 0) | 0 |  | NA | NA |
| ｐ17798 | 1 | 3.07 (0, 0) | 0 |  | NA | NA |
| ｐ17800 | 1 | 0.82 (0, 0) | 0 |  | NA | NA |
| ｐ17804 | 1 | 1.73 (0, 0) | 0 |  | NA | NA |
| ｐ17805 | 1 | 0 (0, 0) | 0 |  | NA | NA |
| ｐ17806 | 1 | 2.86 (0, 0) | 0 |  | NA | NA |
| ｐ17807 | 1 | 2.08 (0, 0) | 0 |  | NA | NA |
| ｐ17808 | 1 | 1.84 (0, 0) | 0 |  | NA | NA |
| ｐ17809 | 1 | 2.42 (0, 0) | 0 |  | NA | NA |
| ｐ17811 | 1 | 1.81 (0, 0) | 0 |  | NA | NA |
| ｐ17812 | 1 | 2.06 (0, 0) | 0 |  | NA | NA |
| ｐ17814 | 1 | 0.58 (0, 0) | 0 |  | NA | NA |
| ｐ17815 | 1 | 2.99 (0, 0) | 0 |  | NA | NA |
| ｐ17817 | 1 | 0 (0, 0) | 0 |  | NA | NA |
| ｐ17818 | 1 | 1.29 (0, 0) | 0 |  | NA | NA |
| ｐ17819 | 1 | 1.55 (0, 0) | 0 |  | NA | NA |
| ｐ17821 | 1 | 1.14 (0, 0) | 0 |  | NA | NA |
| ｐ17822 | 1 | 0.62 (0, 0) | 0 |  | NA | NA |
| ｐ17823 | 1 | 0.64 (0, 0) | 0 |  | NA | NA |
| ｐ17824 | 1 | 0.46 (0, 0) | 0 |  | NA | NA |
| ｐ17825 | 1 | 0 (0, 0) | 0 |  | NA | NA |
| ｐ17827 | 1 | 3.72 (0, 0) | 0 |  | NA | NA |
| ｐ17828 | 1 | 5.9 (0, 0) | 0 |  | NA | NA |
| ｐ17829 | 1 | 0.57 (0, 0) | 0 |  | NA | NA |
| ｐ17831 | 1 | 1.46 (0, 0) | 0 |  | NA | NA |
| ｐ17832 | 1 | 1.53 (0, 0) | 0 |  | NA | NA |
| ｐ17834 | 1 | 0.64 (0, 0) | 0 |  | NA | NA |
| ｐ17835 | 1 | 2.19 (0, 0) | 0 |  | NA | NA |
| ｐ17836 | 1 | 0.63 (0, 0) | 0 |  | NA | NA |
| ｐ17837 | 1 | 0 (0, 0) | 0 |  | NA | NA |
| ｐ17843 | 1 | 0.58 (0, 0) | 0 |  | NA | NA |
| ｐ17846 | 1 | 2.79 (0, 0) | 0 |  | NA | NA |
| ｐ17847 | 1 | 0.2 (0, 0) | 0 |  | NA | NA |
| ｐ17848 | 1 | 4.23 (0, 0) | 0 |  | NA | NA |
| ｐ17850 | 1 | 0.52 (0, 0) | 0 |  | NA | NA |
| ｐ17852 | 1 | 1.41 (0, 0) | 0 |  | NA | NA |
| ｐ17853 | 1 | 1.11 (0, 0) | 0 |  | NA | NA |
| ｐ17855 | 1 | 1.32 (0, 0) | 0 |  | NA | NA |
| ｐ17856 | 1 | 2.93 (0, 0) | 0 |  | NA | NA |
| ｐ17858 | 1 | 0.57 (0, 0) | 0 |  | NA | NA |
| ｐ17861 | 1 | 0.83 (0, 0) | 0 |  | NA | NA |
| ｐ17865 | 1 | 2.1 (0, 0) | 0 |  | NA | NA |
| ｐ17867 | 1 | 0.56 (0, 0) | 0 |  | NA | NA |
| ｐ17870 | 1 | 2.07 (0, 0) | 0 |  | NA | NA |
| ｐ17871 | 1 | 0 (0, 0) | 0 |  | NA | NA |
| ｐ17872 | 1 | 1.77 (0, 0) | 0 |  | NA | NA |
| ｐ17873 | 1 | 0.26 (0, 0) | 0 |  | NA | NA |
| ｐ17874 | 1 | 2.27 (0, 0) | 0 |  | NA | NA |
| ｐ17875 | 1 | 1.6 (0, 0) | 0 |  | NA | NA |
| ｐ17876 | 1 | 0.5 (0, 0) | 0 |  | NA | NA |
| ｐ17877 | 1 | 0.72 (0, 0) | 0 |  | NA | NA |
| ｐ17878 | 1 | 0.54 (0, 0) | 0 |  | NA | NA |
| ｐ17879 | 1 | 0.37 (0, 0) | 0 |  | NA | NA |
| ｐ17882 | 1 | 0.86 (0, 0) | 0 |  | NA | NA |
| ｐ17883 | 1 | 3 (0, 0) | 0 |  | NA | NA |
| ｐ17884 | 1 | 0.79 (0, 0) | 0 |  | NA | NA |
| ｐ17885 | 1 | 1.29 (0, 0) | 0 |  | NA | NA |
| ｐ17886 | 1 | 1.22 (0, 0) | 0 |  | NA | NA |
| ｐ17887 | 1 | 1.16 (0, 0) | 0 |  | NA | NA |
| ｐ17888 | 1 | 1.74 (0, 0) | 0 |  | NA | NA |
| ｐ17890 | 1 | 1.35 (0, 0) | 0 |  | NA | NA |
| ｐ17891 | 1 | 1 (0, 0) | 0 |  | NA | NA |
| ｐ17892 | 1 | 0.59 (0, 0) | 0 |  | NA | NA |
| ｐ17893 | 1 | 0.88 (0, 0) | 0 |  | NA | NA |
| ｐ17894 | 1 | 2.93 (0, 0) | 0 |  | NA | NA |
| ｐ17896 | 1 | 0.59 (0, 0) | 0 |  | NA | NA |
| ｐ17899 | 1 | 0 (0, 0) | 0 |  | NA | NA |
| ｐ17900 | 1 | 1.49 (0, 0) | 0 |  | NA | NA |
| ｐ17901 | 1 | 1.02 (0, 0) | 0 |  | NA | NA |
| ｐ17903 | 1 | 0 (0, 0) | 0 |  | NA | NA |
| ｐ17904 | 1 | 0.28 (0, 0) | 0 |  | NA | NA |
| ｐ17905 | 1 | 0.85 (0, 0) | 0 |  | NA | NA |
| ｐ17906 | 1 | 0.95 (0, 0) | 0 |  | NA | NA |
| ｐ17907 | 1 | 2.24 (0, 0) | 0 |  | NA | NA |
| ｐ17908 | 1 | 0.92 (0, 0) | 0 |  | NA | NA |
| ｐ17909 | 1 | 1.29 (0, 0) | 0 |  | NA | NA |
| ｐ17911 | 1 | 0.95 (0, 0) | 0 |  | NA | NA |
| ｐ17912 | 1 | 1.62 (0, 0) | 0 |  | NA | NA |
| ｐ17913 | 1 | 0.96 (0, 0) | 0 |  | NA | NA |
| ｐ17914 | 1 | 1.58 (0, 0) | 0 |  | NA | NA |
| ｐ17915 | 1 | 1.35 (0, 0) | 0 |  | NA | NA |
| ｐ17917 | 1 | 1.57 (0, 0) | 0 |  | NA | NA |
| ｐ17919 | 1 | 0.97 (0, 0) | 0 |  | NA | NA |
| ｐ17920 | 1 | 1.36 (0, 0) | 0 |  | NA | NA |
| ｐ17921 | 1 | 0.57 (0, 0) | 0 |  | NA | NA |
| ｐ17923 | 1 | 1.12 (0, 0) | 0 |  | NA | NA |
| ｐ17924 | 1 | 1.11 (0, 0) | 0 |  | NA | NA |
| ｐ17925 | 1 | 1.11 (0, 0) | 0 |  | NA | NA |
| ｐ17926 | 1 | 0.3 (0, 0) | 0 |  | NA | NA |
| ｐ17927 | 1 | 1.49 (0, 0) | 0 |  | NA | NA |
| ｐ17928 | 1 | 1.17 (0, 0) | 0 |  | NA | NA |
| ｐ17929 | 1 | 0.92 (0, 0) | 0 |  | NA | NA |
| ｐ17931 | 1 | 2.97 (0, 0) | 0 |  | NA | NA |
| ｐ17932 | 1 | 0.67 (0, 0) | 0 |  | NA | NA |
| ｐ17933 | 1 | 0.33 (0, 0) | 0 |  | NA | NA |
| ｐ17934 | 1 | 1.34 (0, 0) | 0 |  | NA | NA |
| ｐ17937 | 1 | 2.26 (0, 0) | 0 |  | NA | NA |
| ｐ17938 | 1 | 2.28 (0, 0) | 0 |  | NA | NA |
| ｐ17939 | 1 | 0.36 (0, 0) | 0 |  | NA | NA |
| ｐ17943 | 1 | 0.85 (0, 0) | 0 |  | NA | NA |
| ｐ17944 | 1 | 4.23 (0, 0) | 0 |  | NA | NA |
| ｐ17948 | 1 | 0.4 (0, 0) | 0 |  | NA | NA |
| ｐ17950 | 1 | 0 (0, 0) | 0 |  | NA | NA |
| ｐ17961 | 1 | 1.45 (0, 0) | 0 |  | NA | NA |
| ｐ17964 | 1 | 0.41 (0, 0) | 0 |  | NA | NA |
| ｐ17971 | 1 | 0.81 (0, 0) | 0 |  | NA | NA |
| ｐ17977 | 1 | 1.5 (0, 0) | 0 |  | NA | NA |
| ｐ17987 | 1 | 1.46 (0, 0) | 0 |  | NA | NA |
| ｐ18024 | 1 | 1.06 (0, 0) | 0 |  | NA | NA |
| ｐ18026 | 1 | 1.35 (0, 0) | 0 |  | NA | NA |
| ｐ18028 | 1 | 0.54 (0, 0) | 0 |  | NA | NA |
| ｐ18070 | 1 | 1.02 (0, 0) | 0 |  | NA | NA |
| ｐ18113 | 1 | 0.86 (0, 0) | 0 |  | NA | NA |
| ｐ18121 | 1 | 0.96 (0, 0) | 0 |  | NA | NA |
| ｐ18122 | 1 | 1.54 (0, 0) | 0 |  | NA | NA |
| ｐ18140 | 1 | 2.94 (0, 0) | 0 |  | NA | NA |
| ｐ18146 | 1 | 0 (0, 0) | 0 |  | NA | NA |
| ｐ18177 | 1 | 1.22 (0, 0) | 0 |  | NA | NA |
| ｐ18186 | 1 | 0.57 (0, 0) | 0 |  | NA | NA |
| ｐ18187 | 1 | 0.97 (0, 0) | 0 |  | NA | NA |
| ｐ18225 | 1 | 0.72 (0, 0) | 0 |  | NA | NA |
| ｐ18235 | 1 | 0.97 (0, 0) | 0 |  | NA | NA |
| ｐ18240 | 1 | 0.92 (0, 0) | 0 |  | NA | NA |
| ｐ18274 | 1 | 1.23 (0, 0) | 0 |  | NA | NA |
| ｐ18329 | 1 | 0.85 (0, 0) | 0 |  | NA | NA |
| ｐ18336 | 1 | 1.22 (0, 0) | 0 |  | NA | NA |
| ｐ18338 | 1 | 0.8 (0, 0) | 0 |  | NA | NA |
| ｐ18341 | 1 | 1.66 (0, 0) | 0 |  | NA | NA |
| ｐ18348 | 1 | 1.02 (0, 0) | 0 |  | NA | NA |
| ｐ18385 | 1 | 1.01 (0, 0) | 0 |  | NA | NA |
| ｐ18399 | 1 | 0.8 (0, 0) | 0 |  | NA | NA |
| ｐ18404 | 1 | 0.81 (0, 0) | 0 |  | NA | NA |
| ｐ18417 | 1 | 1.42 (0, 0) | 0 |  | NA | NA |
| ｐ18425 | 1 | 1.53 (0, 0) | 0 |  | NA | NA |
| ｐ18468 | 1 | 0.43 (0, 0) | 0 |  | NA | NA |
| ｐ18474 | 1 | 0.75 (0, 0) | 0 |  | NA | NA |
| ｐ18475 | 1 | 1.82 (0, 0) | 0 |  | NA | NA |
| ｐ18500 | 1 | 1.25 (0, 0) | 0 |  | NA | NA |
| ｐ18501 | 1 | 0.5 (0, 0) | 0 |  | NA | NA |
| ｐ18565 | 1 | 0.57 (0, 0) | 0 |  | NA | NA |
| ｐ18582 | 1 | 1.14 (0, 0) | 0 |  | NA | NA |
| ｐ18612 | 1 | 1.09 (0, 0) | 0 |  | NA | NA |
| ｐ18617 | 1 | 1.18 (0, 0) | 0 |  | NA | NA |
| ｐ18626 | 1 | 0.98 (0, 0) | 0 |  | NA | NA |
| ｐ18628 | 1 | 1.77 (0, 0) | 0 |  | NA | NA |
| ｐ18656 | 1 | 1.06 (0, 0) | 0 |  | NA | NA |
| ｐ18674 | 1 | 0.78 (0, 0) | 0 |  | NA | NA |
| ｐ18679 | 1 | 0.62 (0, 0) | 0 |  | NA | NA |
| ｐ18693 | 1 | 0.78 (0, 0) | 0 |  | NA | NA |
| ｐ18730 | 1 | 3.16 (0, 0) | 0 |  | NA | NA |
| ｐ18746 | 1 | 1 (0, 0) | 0 |  | NA | NA |
| ｐ18756 | 1 | 1.07 (0, 0) | 0 |  | NA | NA |
| ｐ18763 | 1 | 0.89 (0, 0) | 0 |  | NA | NA |
| ｐ18764 | 1 | 4.24 (0, 0) | 0 |  | NA | NA |
| ｐ18788 | 1 | 1.08 (0, 0) | 0 |  | NA | NA |
| ｐ18814 | 1 | 0.44 (0, 0) | 0 |  | NA | NA |
| ｐ18834 | 1 | 0.33 (0, 0) | 0 |  | NA | NA |
| ｐ18839 | 1 | 0.68 (0, 0) | 0 |  | NA | NA |
| ｐ18840 | 1 | 1.71 (0, 0) | 0 |  | NA | NA |
| ｐ18847 | 1 | 1.5 (0, 0) | 0 |  | NA | NA |
| ｐ18848 | 1 | 0.91 (0, 0) | 0 |  | NA | NA |
| ｐ18892 | 1 | 1.03 (0, 0) | 0 |  | NA | NA |
| ｐ18918 | 1 | 1.51 (0, 0) | 0 |  | NA | NA |
| ｐ18953 | 1 | 1.19 (0, 0) | 0 |  | NA | NA |
| ｐ18957 | 1 | 0.98 (0, 0) | 0 |  | NA | NA |
| ｐ18961 | 1 | 0.68 (0, 0) | 0 |  | NA | NA |
| ｐ18978 | 1 | 0.74 (0, 0) | 0 |  | NA | NA |
| ｐ19029 | 1 | 0.77 (0, 0) | 0 |  | NA | NA |
| ｐ19037 | 1 | 2.37 (0, 0) | 0 |  | NA | NA |
| ｐ19049 | 1 | 0.99 (0, 0) | 0 |  | NA | NA |
| ｐ19073 | 1 | 1.72 (0, 0) | 0 |  | NA | NA |
| ｐ19077 | 1 | 0.45 (0, 0) | 0 |  | NA | NA |
| ｐ19085 | 1 | 1.48 (0, 0) | 0 |  | NA | NA |
| ｐ19087 | 1 | 1.03 (0, 0) | 0 |  | NA | NA |
| ｐ19096 | 1 | 0.45 (0, 0) | 0 |  | NA | NA |
| ｐ19109 | 1 | 1.48 (0, 0) | 0 |  | NA | NA |
| ｐ19123 | 1 | 0.9 (0, 0) | 0 |  | NA | NA |
| ｐ19135 | 1 | 0.23 (0, 0) | 0 |  | NA | NA |
| ｐ19144 | 1 | 7.67 (0, 0) | 0 |  | NA | NA |
| ｐ19149 | 1 | 1.53 (0, 0) | 0 |  | NA | NA |
| ｐ19155 | 1 | 2.74 (0, 0) | 0 |  | NA | NA |
| ｐ19162 | 1 | 1.23 (0, 0) | 0 |  | NA | NA |
| ｐ19175 | 1 | 1.22 (0, 0) | 0 |  | NA | NA |
| ｐ19191 | 1 | 1.11 (0, 0) | 0 |  | NA | NA |
| ｐ19199 | 1 | 1.09 (0, 0) | 0 |  | NA | NA |
| ｐ19222 | 1 | 1.7 (0, 0) | 0 |  | NA | NA |
| ｐ19224 | 1 | 0.4 (0, 0) | 0 |  | NA | NA |
| ｐ19229 | 1 | 1.19 (0, 0) | 0 |  | NA | NA |
| ｐ19233 | 1 | 0.62 (0, 0) | 0 |  | NA | NA |
| ｐ19259 | 1 | 1.24 (0, 0) | 0 |  | NA | NA |
| ｐ19265 | 1 | 0.81 (0, 0) | 0 |  | NA | NA |
| ｐ19293 | 1 | 0.33 (0, 0) | 0 |  | NA | NA |
| ｐ19332 | 1 | 0.47 (0, 0) | 0 |  | NA | NA |
| ｐ19362 | 1 | 1.1 (0, 0) | 0 |  | NA | NA |
| ｐ19567 | 1 | 4.26 (0, 0) | 0 |  | NA | NA |
| ｐ19586 | 1 | 1.33 (0, 0) | 0 |  | NA | NA |
| ｐ19622 | 1 | 1.25 (0, 0) | 0 |  | NA | NA |
| ｐ19635 | 1 | 1.24 (0, 0) | 0 |  | NA | NA |
| ｐ19646 | 1 | 0.6 (0, 0) | 0 |  | NA | NA |
| ｐ19666 | 1 | 4.63 (0, 0) | 0 |  | NA | NA |
| ｐ19685 | 1 | 0 (0, 0) | 0 |  | NA | NA |
| ｐ19686 | 1 | 0 (0, 0) | 0 |  | NA | NA |
| ｐ19687 | 1 | 17.34 (0, 0) | 0 |  | NA | NA |
| ｐ19688 | 1 | 2.01 (0, 0) | 0 |  | NA | NA |
| ｐ19689 | 1 | 2.62 (0, 0) | 0 |  | NA | NA |
| ｐ19690 | 1 | 1.18 (0, 0) | 0 |  | NA | NA |
| ｐ19691 | 1 | 0.46 (0, 0) | 0 |  | NA | NA |
| ｐ19692 | 1 | 1.79 (0, 0) | 0 |  | NA | NA |
| ｐ19694 | 1 | 6.63 (0, 0) | 0 |  | NA | NA |
| ｐ19696 | 1 | 1.11 (0, 0) | 0 |  | NA | NA |
| ｐ19697 | 1 | 0.4 (0, 0) | 0 |  | NA | NA |
| ｐ19698 | 1 | 2.78 (0, 0) | 0 |  | NA | NA |
| ｐ19699 | 1 | 5.72 (0, 0) | 0 |  | NA | NA |
| ｐ19701 | 1 | 0.35 (0, 0) | 0 |  | NA | NA |
| ｐ19702 | 1 | 2.31 (0, 0) | 0 |  | NA | NA |
| ｐ19704 | 1 | 0.83 (0, 0) | 0 |  | NA | NA |
| ｐ19706 | 1 | 1.26 (0, 0) | 0 |  | NA | NA |
| ｐ19707 | 1 | 1.99 (0, 0) | 0 |  | NA | NA |
| ｐ19709 | 1 | 0.72 (0, 0) | 0 |  | NA | NA |
| ｐ19710 | 1 | 1.53 (0, 0) | 0 |  | NA | NA |
| ｐ19711 | 1 | 1.81 (0, 0) | 0 |  | NA | NA |
| ｐ19714 | 1 | 0.48 (0, 0) | 0 |  | NA | NA |
| ｐ19715 | 1 | 1.13 (0, 0) | 0 |  | NA | NA |
| ｐ19716 | 1 | 0.6 (0, 0) | 0 |  | NA | NA |
| ｐ19717 | 1 | 0.88 (0, 0) | 0 |  | NA | NA |
| ｐ19721 | 1 | 0.98 (0, 0) | 0 |  | NA | NA |
| ｐ19722 | 1 | 1.33 (0, 0) | 0 |  | NA | NA |
| ｐ19724 | 1 | 1 (0, 0) | 0 |  | NA | NA |
| ｐ19725 | 1 | 0.96 (0, 0) | 0 |  | NA | NA |
| ｐ19726 | 1 | 1.23 (0, 0) | 0 |  | NA | NA |
| ｐ19727 | 1 | 2.13 (0, 0) | 0 |  | NA | NA |
| ｐ19729 | 1 | 0.8 (0, 0) | 0 |  | NA | NA |
| ｐ19730 | 1 | 1.05 (0, 0) | 0 |  | NA | NA |
| ｐ19731 | 1 | 0.71 (0, 0) | 0 |  | NA | NA |
| ｐ19736 | 1 | 0.2 (0, 0) | 0 |  | NA | NA |
| ｐ19738 | 1 | 1.44 (0, 0) | 0 |  | NA | NA |
| ｐ19740 | 1 | 0.98 (0, 0) | 0 |  | NA | NA |
| ｐ19742 | 1 | 1.42 (0, 0) | 0 |  | NA | NA |
| ｐ19743 | 1 | 2.18 (0, 0) | 0 |  | NA | NA |
| ｐ19746 | 1 | 0.88 (0, 0) | 0 |  | NA | NA |
| ｐ19747 | 1 | 0 (0, 0) | 0 |  | NA | NA |
| ｐ19748 | 1 | 1.05 (0, 0) | 0 |  | NA | NA |
| ｐ19749 | 1 | 1.34 (0, 0) | 0 |  | NA | NA |
| ｐ19752 | 1 | 0.21 (0, 0) | 0 |  | NA | NA |
| ｐ19753 | 1 | 0.67 (0, 0) | 0 |  | NA | NA |
| ｐ19754 | 1 | 0.79 (0, 0) | 0 |  | NA | NA |
| ｐ19756 | 1 | 1.61 (0, 0) | 0 |  | NA | NA |
| ｐ19757 | 1 | 1.03 (0, 0) | 0 |  | NA | NA |
| ｐ19759 | 1 | 2.25 (0, 0) | 0 |  | NA | NA |
| ｐ19760 | 1 | 5.8 (0, 0) | 0 |  | NA | NA |
| ｐ19762 | 1 | 1.74 (0, 0) | 0 |  | NA | NA |
| ｐ19763 | 1 | 0.72 (0, 0) | 0 |  | NA | NA |
| ｐ19764 | 1 | 1.97 (0, 0) | 0 |  | NA | NA |
| ｐ19766 | 1 | 0.79 (0, 0) | 0 |  | NA | NA |
| ｐ19767 | 1 | 0.54 (0, 0) | 0 |  | NA | NA |
| ｐ19768 | 1 | 0.61 (0, 0) | 0 |  | NA | NA |
| ｐ19769 | 1 | 1.17 (0, 0) | 0 |  | NA | NA |
| ｐ19770 | 1 | 1.8 (0, 0) | 0 |  | NA | NA |
| ｐ19772 | 1 | 2.67 (0, 0) | 0 |  | NA | NA |
| ｐ19773 | 1 | 0.21 (0, 0) | 0 |  | NA | NA |
| ｐ19777 | 1 | 0.91 (0, 0) | 0 |  | NA | NA |
| ｐ19779 | 1 | 12.61 (0, 0) | 0 |  | NA | NA |
| ｐ19781 | 1 | 1.24 (0, 0) | 0 |  | NA | NA |
| ｐ19784 | 1 | 2.04 (0, 0) | 0 |  | NA | NA |
| ｐ19785 | 1 | 0.27 (0, 0) | 0 |  | NA | NA |
| ｐ19786 | 1 | 0.95 (0, 0) | 0 |  | NA | NA |
| ｐ19787 | 1 | 0.28 (0, 0) | 0 |  | NA | NA |
| ｐ19788 | 1 | 0.4 (0, 0) | 0 |  | NA | NA |
| ｐ19789 | 1 | 0.3 (0, 0) | 0 |  | NA | NA |
| ｐ19790 | 1 | 3.08 (0, 0) | 0 |  | NA | NA |
| ｐ19791 | 1 | 0 (0, 0) | 0 |  | NA | NA |
| ｐ19792 | 1 | 0 (0, 0) | 0 |  | NA | NA |
| ｐ19793 | 1 | 0 (0, 0) | 0 |  | NA | NA |
| ｐ19794 | 1 | 0 (0, 0) | 0 |  | NA | NA |
| ｐ19795 | 1 | 0.43 (0, 0) | 0 |  | NA | NA |
| ｐ19797 | 1 | 0.28 (0, 0) | 0 |  | NA | NA |
| ｐ19798 | 1 | 0.35 (0, 0) | 0 |  | NA | NA |
| ｐ19800 | 1 | 0.81 (0, 0) | 0 |  | NA | NA |
| ｐ19824 | 1 | 1.02 (0, 0) | 0 |  | NA | NA |
| ｐ19848 | 1 | 0.51 (0, 0) | 0 |  | NA | NA |
| ｐ19855 | 1 | 1.08 (0, 0) | 0 |  | NA | NA |
| ｐ19870 | 1 | 0.59 (0, 0) | 0 |  | NA | NA |
| ｐ19885 | 1 | 0 (0, 0) | 0 |  | NA | NA |
| ｐ19886 | 1 | 1.25 (0, 0) | 0 |  | NA | NA |
| ｐ19900 | 1 | 1.07 (0, 0) | 0 |  | NA | NA |
| ｐ19916 | 1 | 1.73 (0, 0) | 0 |  | NA | NA |
| ｐ19942 | 1 | 1.11 (0, 0) | 0 |  | NA | NA |
| ｐ19946 | 1 | 0.37 (0, 0) | 0 |  | NA | NA |
| ｐ19956 | 1 | 0.52 (0, 0) | 0 |  | NA | NA |
| ｐ19962 | 1 | 1.3 (0, 0) | 0 |  | NA | NA |
| ｐ19963 | 1 | 2.86 (0, 0) | 0 |  | NA | NA |
| ｐ19964 | 1 | 1.04 (0, 0) | 0 |  | NA | NA |
| ｐ19968 | 1 | 0.87 (0, 0) | 0 |  | NA | NA |
| ｐ19983 | 1 | 1 (0, 0) | 0 |  | NA | NA |
| ｐ20014 | 1 | 0.33 (0, 0) | 0 |  | NA | NA |
| ｐ20021 | 1 | 1.16 (0, 0) | 0 |  | NA | NA |
| ｐ20032 | 1 | 1.12 (0, 0) | 0 |  | NA | NA |
| ｐ20038 | 1 | 0.78 (0, 0) | 0 |  | NA | NA |
| ｐ20050 | 1 | 0.9 (0, 0) | 0 |  | NA | NA |
| ｐ20075 | 1 | 0 (0, 0) | 0 |  | NA | NA |
| ｐ20076 | 1 | 0.58 (0, 0) | 0 |  | NA | NA |
| ｐ20082 | 1 | 1.09 (0, 0) | 0 |  | NA | NA |
| ｐ20083 | 1 | 0 (0, 0) | 0 |  | NA | NA |
| ｐ20084 | 1 | 0 (0, 0) | 0 |  | NA | NA |
| ｐ20085 | 1 | 0.43 (0, 0) | 0 |  | NA | NA |
| ｐ20088 | 1 | 0.31 (0, 0) | 0 |  | NA | NA |
| ｐ20089 | 1 | 0.73 (0, 0) | 0 |  | NA | NA |
| ｐ20090 | 1 | 1.74 (0, 0) | 0 |  | NA | NA |
| ｐ20091 | 1 | 1.79 (0, 0) | 0 |  | NA | NA |
| ｐ20092 | 1 | 0.03 (0, 0) | 0 |  | NA | NA |
| ｐ20093 | 1 | 0.34 (0, 0) | 0 |  | NA | NA |
| ｐ20094 | 1 | 2.37 (0, 0) | 0 |  | NA | NA |
| ｐ20095 | 1 | 6.98 (0, 0) | 0 |  | NA | NA |
| ｐ20096 | 1 | 0.43 (0, 0) | 0 |  | NA | NA |
| ｐ20098 | 1 | 1.43 (0, 0) | 0 |  | NA | NA |
| ｐ20099 | 1 | 1.17 (0, 0) | 0 |  | NA | NA |
| ｐ20100 | 1 | 2.06 (0, 0) | 0 |  | NA | NA |
| ｐ20101 | 1 | 1.2 (0, 0) | 0 |  | NA | NA |
| ｐ20102 | 1 | 0.37 (0, 0) | 0 |  | NA | NA |
| ｐ20103 | 1 | 2.12 (0, 0) | 0 |  | NA | NA |
| ｐ20104 | 1 | 1.36 (0, 0) | 0 |  | NA | NA |
| ｐ20106 | 1 | 1.11 (0, 0) | 0 |  | NA | NA |
| ｐ20107 | 1 | 0.14 (0, 0) | 0 |  | NA | NA |
| ｐ20108 | 1 | 0.76 (0, 0) | 0 |  | NA | NA |
| ｐ20109 | 1 | 0.82 (0, 0) | 0 |  | NA | NA |
| ｐ20111 | 1 | 2.34 (0, 0) | 0 |  | NA | NA |
| ｐ20113 | 1 | 0.84 (0, 0) | 0 |  | NA | NA |
| ｐ20114 | 1 | 1.18 (0, 0) | 0 |  | NA | NA |
| ｐ20115 | 1 | 1.46 (0, 0) | 0 |  | NA | NA |
| ｐ20116 | 1 | 4.84 (0, 0) | 0 |  | NA | NA |
| ｐ20117 | 1 | 0.62 (0, 0) | 0 |  | NA | NA |
| ｐ20118 | 1 | 0.74 (0, 0) | 0 |  | NA | NA |
| ｐ20121 | 1 | 0.72 (0, 0) | 0 |  | NA | NA |
| ｐ20122 | 1 | 0.44 (0, 0) | 0 |  | NA | NA |
| ｐ20123 | 1 | 1.43 (0, 0) | 0 |  | NA | NA |
| ｐ20124 | 1 | 0.95 (0, 0) | 0 |  | NA | NA |
| ｐ20126 | 1 | 0.29 (0, 0) | 0 |  | NA | NA |
| ｐ20128 | 1 | 0.47 (0, 0) | 0 |  | NA | NA |
| ｐ20129 | 1 | 0.58 (0, 0) | 0 |  | NA | NA |
| ｐ20130 | 1 | 0.55 (0, 0) | 0 |  | NA | NA |
| ｐ20132 | 1 | 1.17 (0, 0) | 0 |  | NA | NA |
| ｐ20133 | 1 | 0.09 (0, 0) | 0 |  | NA | NA |
| ｐ20135 | 1 | 0.6 (0, 0) | 0 |  | NA | NA |
| ｐ20136 | 1 | 0.71 (0, 0) | 0 |  | NA | NA |
| ｐ20137 | 1 | 1.21 (0, 0) | 0 |  | NA | NA |
| ｐ20138 | 1 | 3.44 (0, 0) | 0 |  | NA | NA |
| ｐ20139 | 1 | 0.49 (0, 0) | 0 |  | NA | NA |
| ｐ20141 | 1 | 1.41 (0, 0) | 0 |  | NA | NA |
| ｐ20142 | 1 | 1.59 (0, 0) | 0 |  | NA | NA |
| ｐ20143 | 1 | 0.83 (0, 0) | 0 |  | NA | NA |
| ｐ20144 | 1 | 1.37 (0, 0) | 0 |  | NA | NA |
| ｐ20145 | 1 | 0.73 (0, 0) | 0 |  | NA | NA |
| ｐ20146 | 1 | 2.04 (0, 0) | 0 |  | NA | NA |
| ｐ20148 | 1 | 0.98 (0, 0) | 0 |  | NA | NA |
| ｐ20149 | 1 | 0.46 (0, 0) | 0 |  | NA | NA |
| ｐ20151 | 1 | 1.74 (0, 0) | 0 |  | NA | NA |
| ｐ20152 | 1 | 1.39 (0, 0) | 0 |  | NA | NA |
| ｐ20153 | 1 | 0 (0, 0) | 0 |  | NA | NA |
| ｐ20154 | 1 | 0.81 (0, 0) | 0 |  | NA | NA |
| ｐ20156 | 1 | 1.41 (0, 0) | 0 |  | NA | NA |
| ｐ20158 | 1 | 0.62 (0, 0) | 0 |  | NA | NA |
| ｐ20160 | 1 | 0.82 (0, 0) | 0 |  | NA | NA |
| ｐ20163 | 1 | 0.63 (0, 0) | 0 |  | NA | NA |
| ｐ20164 | 1 | 0.69 (0, 0) | 0 |  | NA | NA |
| ｐ20165 | 1 | 1.18 (0, 0) | 0 |  | NA | NA |
| ｐ20166 | 1 | 0.86 (0, 0) | 0 |  | NA | NA |
| ｐ20167 | 1 | 1.25 (0, 0) | 0 |  | NA | NA |
| ｐ20172 | 1 | 0.83 (0, 0) | 0 |  | NA | NA |
| ｐ20174 | 1 | 0 (0, 0) | 0 |  | NA | NA |
| ｐ20175 | 1 | 0.66 (0, 0) | 0 |  | NA | NA |
| ｐ20176 | 1 | 0.89 (0, 0) | 0 |  | NA | NA |
| ｐ20177 | 1 | 0 (0, 0) | 0 |  | NA | NA |
| ｐ20178 | 1 | 1.86 (0, 0) | 0 |  | NA | NA |
| ｐ20181 | 1 | 0.57 (0, 0) | 0 |  | NA | NA |
| ｐ20182 | 1 | 4.67 (0, 0) | 0 |  | NA | NA |
| ｐ20183 | 1 | 0.87 (0, 0) | 0 |  | NA | NA |
| ｐ20184 | 1 | 2.83 (0, 0) | 0 |  | NA | NA |
| ｐ20185 | 1 | 0.43 (0, 0) | 0 |  | NA | NA |
| ｐ20186 | 1 | 0 (0, 0) | 0 |  | NA | NA |
| ｐ20192 | 1 | 0.83 (0, 0) | 0 |  | NA | NA |
| ｐ20193 | 1 | 1.49 (0, 0) | 0 |  | NA | NA |
| ｐ20194 | 1 | 2.77 (0, 0) | 0 |  | NA | NA |
| ｐ20195 | 1 | 0.72 (0, 0) | 0 |  | NA | NA |
| ｐ20196 | 1 | 2.17 (0, 0) | 0 |  | NA | NA |
| ｐ20199 | 1 | 3.39 (0, 0) | 0 |  | NA | NA |
| ｐ20201 | 1 | 2.04 (0, 0) | 0 |  | NA | NA |
| ｐ20203 | 1 | 0 (0, 0) | 0 |  | NA | NA |
| ｐ20204 | 1 | 0.69 (0, 0) | 0 |  | NA | NA |
| ｐ20205 | 1 | 0.76 (0, 0) | 0 |  | NA | NA |
| ｐ20206 | 1 | 0.26 (0, 0) | 0 |  | NA | NA |
| ｐ20208 | 1 | 0 (0, 0) | 0 |  | NA | NA |
| ｐ20209 | 1 | 2.21 (0, 0) | 0 |  | NA | NA |
| ｐ20210 | 1 | 2.63 (0, 0) | 0 |  | NA | NA |
| ｐ20212 | 1 | 0.79 (0, 0) | 0 |  | NA | NA |
| ｐ20213 | 1 | 0.56 (0, 0) | 0 |  | NA | NA |
| ｐ20214 | 1 | 0.68 (0, 0) | 0 |  | NA | NA |
| ｐ20215 | 1 | 1.62 (0, 0) | 0 |  | NA | NA |
| ｐ20254 | 1 | 0.05 (0, 0) | 0 |  | NA | NA |
| ｐ20264 | 1 | 0.44 (0, 0) | 0 |  | NA | NA |
| ｐ20272 | 1 | 1.3 (0, 0) | 0 |  | NA | NA |
| ｐ20282 | 1 | 0.91 (0, 0) | 0 |  | NA | NA |
| ｐ20288 | 1 | 1.42 (0, 0) | 0 |  | NA | NA |
| ｐ20300 | 1 | 1.1 (0, 0) | 0 |  | NA | NA |
| ｐ20311 | 1 | 0.82 (0, 0) | 0 |  | NA | NA |
| ｐ20316 | 1 | 5.95 (0, 0) | 0 |  | NA | NA |
| ｐ20325 | 1 | 1.05 (0, 0) | 0 |  | NA | NA |
| ｐ20343 | 1 | 1.64 (0, 0) | 0 |  | NA | NA |
| ｐ20395 | 1 | 0.88 (0, 0) | 0 |  | NA | NA |
| ｐ20419 | 1 | 0.65 (0, 0) | 0 |  | NA | NA |
| ｐ20424 | 1 | 0.37 (0, 0) | 0 |  | NA | NA |
| ｐ20487 | 1 | 1.08 (0, 0) | 0 |  | NA | NA |
| ｐ20529 | 1 | 3.06 (0, 0) | 0 |  | NA | NA |
| ｐ20533 | 1 | 1.35 (0, 0) | 0 |  | NA | NA |
| ｐ20571 | 1 | 1.42 (0, 0) | 0 |  | NA | NA |
| ｐ20579 | 1 | 1.25 (0, 0) | 0 |  | NA | NA |
| ｐ20582 | 1 | 1 (0, 0) | 0 |  | NA | NA |
| ｐ20613 | 1 | 3.24 (0, 0) | 0 |  | NA | NA |
| ｐ20627 | 1 | 0.8 (0, 0) | 0 |  | NA | NA |
| ｐ20631 | 1 | 0.36 (0, 0) | 0 |  | NA | NA |
| ｐ20693 | 1 | 2 (0, 0) | 0 |  | NA | NA |
| ｐ20712 | 1 | 0 (0, 0) | 0 |  | NA | NA |
| ｐ20718 | 0 |  | 39 | 0.45 (0.28, 0.63) | NA | NA |
| ｐ20746 | 1 | 0.48 (0, 0) | 0 |  | NA | NA |
| ｐ20747 | 1 | 0.57 (0, 0) | 0 |  | NA | NA |
| ｐ20752 | 1 | 3.06 (0, 0) | 0 |  | NA | NA |
| ｐ20754 | 1 | 1.34 (0, 0) | 0 |  | NA | NA |
| ｐ20772 | 1 | 1.03 (0, 0) | 0 |  | NA | NA |
| ｐ20781 | 1 | 1.07 (0, 0) | 0 |  | NA | NA |
| ｐ20801 | 1 | 0.47 (0, 0) | 0 |  | NA | NA |
| ｐ20808 | 1 | 0 (0, 0) | 0 |  | NA | NA |
| ｐ20814 | 1 | 1.11 (0, 0) | 0 |  | NA | NA |
| ｐ20831 | 1 | 1.06 (0, 0) | 0 |  | NA | NA |
| ｐ20833 | 1 | 3.22 (0, 0) | 0 |  | NA | NA |
| ｐ20834 | 1 | 0.53 (0, 0) | 0 |  | NA | NA |
| ｐ20842 | 1 | 0.65 (0, 0) | 0 |  | NA | NA |
| ｐ20886 | 1 | 0.94 (0, 0) | 0 |  | NA | NA |
| ｐ20902 | 1 | 0.56 (0, 0) | 0 |  | NA | NA |
| ｐ20926 | 1 | 1.2 (0, 0) | 0 |  | NA | NA |
| ｐ20946 | 1 | 1.11 (0, 0) | 0 |  | NA | NA |
| ｐ20957 | 1 | 1.1 (0, 0) | 0 |  | NA | NA |
| ｐ20963 | 1 | 0 (0, 0) | 0 |  | NA | NA |
| ｐ20986 | 1 | 1.26 (0, 0) | 0 |  | NA | NA |
| ｐ21016 | 1 | 0.52 (0, 0) | 0 |  | NA | NA |
| ｐ21029 | 1 | 0.89 (0, 0) | 0 |  | NA | NA |
| ｐ21035 | 1 | 2.17 (0, 0) | 0 |  | NA | NA |
| ｐ21062 | 1 | 0.8 (0, 0) | 0 |  | NA | NA |
| ｐ21077 | 1 | 0.83 (0, 0) | 0 |  | NA | NA |
| ｐ21090 | 1 | 1.03 (0, 0) | 0 |  | NA | NA |
| ｐ21104 | 1 | 0.86 (0, 0) | 0 |  | NA | NA |
| ｐ21132 | 1 | 49.23 (0, 0) | 0 |  | NA | NA |
| ｐ21139 | 1 | 0.58 (0, 0) | 0 |  | NA | NA |
| ｐ21180 | 1 | 0.73 (0, 0) | 0 |  | NA | NA |
| ｐ21188 | 1 | 0.54 (0, 0) | 0 |  | NA | NA |
| ｐ21241 | 1 | 0.95 (0, 0) | 0 |  | NA | NA |
| ｐ21245 | 1 | 17 (0, 0) | 0 |  | NA | NA |
| ｐ21247 | 1 | 0.65 (0, 0) | 0 |  | NA | NA |
| ｐ21262 | 1 | 0.5 (0, 0) | 0 |  | NA | NA |
| ｐ21263 | 1 | 1.26 (0, 0) | 0 |  | NA | NA |
| ｐ21265 | 1 | 0.2 (0, 0) | 0 |  | NA | NA |
| ｐ21267 | 1 | 8.63 (0, 0) | 0 |  | NA | NA |
| ｐ21268 | 1 | 2.6 (0, 0) | 0 |  | NA | NA |
| ｐ21269 | 1 | 1.51 (0, 0) | 0 |  | NA | NA |
| ｐ21270 | 1 | 2.57 (0, 0) | 0 |  | NA | NA |
| ｐ21272 | 1 | 1.33 (0, 0) | 0 |  | NA | NA |
| ｐ21273 | 1 | 3.38 (0, 0) | 0 |  | NA | NA |
| ｐ21274 | 1 | 1.24 (0, 0) | 0 |  | NA | NA |
| ｐ21299 | 1 | 4.08 (0, 0) | 0 |  | NA | NA |
| ｐ21304 | 1 | 1.24 (0, 0) | 0 |  | NA | NA |
| ｐ21308 | 1 | 1.03 (0, 0) | 0 |  | NA | NA |
| ｐ21326 | 1 | 0.72 (0, 0) | 0 |  | NA | NA |
| ｐ21358 | 1 | 0.98 (0, 0) | 0 |  | NA | NA |
| ｐ21361 | 1 | 0.56 (0, 0) | 0 |  | NA | NA |
| ｐ21363 | 1 | 0.67 (0, 0) | 0 |  | NA | NA |
| ｐ21387 | 1 | 0.76 (0, 0) | 0 |  | NA | NA |
| ｐ21454 | 1 | 0.92 (0, 0) | 0 |  | NA | NA |
| ｐ21473 | 1 | 0.83 (0, 0) | 0 |  | NA | NA |
| ｐ21475 | 1 | 0.97 (0, 0) | 0 |  | NA | NA |
| ｐ21503 | 1 | 0.97 (0, 0) | 0 |  | NA | NA |
| ｐ21538 | 1 | 0.53 (0, 0) | 0 |  | NA | NA |
| ｐ21540 | 1 | 0.69 (0, 0) | 0 |  | NA | NA |
| ｐ21545 | 1 | 1.46 (0, 0) | 0 |  | NA | NA |
| ｐ21546 | 1 | 0.85 (0, 0) | 0 |  | NA | NA |
| ｐ21564 | 1 | 0.57 (0, 0) | 0 |  | NA | NA |
| ｐ21616 | 1 | 1.01 (0, 0) | 0 |  | NA | NA |
| ｐ21634 | 1 | 0.83 (0, 0) | 0 |  | NA | NA |
| ｐ21656 | 1 | 1.05 (0, 0) | 0 |  | NA | NA |
| ｐ21682 | 1 | 0.64 (0, 0) | 0 |  | NA | NA |
| ｐ21685 | 1 | 1.25 (0, 0) | 0 |  | NA | NA |
| ｐ21690 | 1 | 0.87 (0, 0) | 0 |  | NA | NA |
| ｐ21692 | 1 | 1.39 (0, 0) | 0 |  | NA | NA |
| ｐ21709 | 1 | 0.79 (0, 0) | 0 |  | NA | NA |
| ｐ21714 | 1 | 2.52 (0, 0) | 0 |  | NA | NA |
| ｐ21760 | 1 | 1.02 (0, 0) | 0 |  | NA | NA |
| ｐ21808 | 1 | 1.24 (0, 0) | 0 |  | NA | NA |
| ｐ21884 | 1 | 3.41 (0, 0) | 0 |  | NA | NA |
| ｐ21885 | 1 | 1.36 (0, 0) | 0 |  | NA | NA |
| ｐ21886 | 1 | 3.35 (0, 0) | 0 |  | NA | NA |
| ｐ21887 | 1 | 0.39 (0, 0) | 0 |  | NA | NA |
| ｐ21891 | 1 | 0.89 (0, 0) | 0 |  | NA | NA |
| ｐ21892 | 1 | 0 (0, 0) | 0 |  | NA | NA |
| ｐ21893 | 1 | 2.39 (0, 0) | 0 |  | NA | NA |
| ｐ21894 | 1 | 0.87 (0, 0) | 0 |  | NA | NA |
| ｐ21896 | 1 | 2.2 (0, 0) | 0 |  | NA | NA |
| ｐ21898 | 1 | 0.81 (0, 0) | 0 |  | NA | NA |
| ｐ21900 | 1 | 0 (0, 0) | 0 |  | NA | NA |
| ｐ21901 | 1 | 0 (0, 0) | 0 |  | NA | NA |
| ｐ21902 | 1 | 1.72 (0, 0) | 0 |  | NA | NA |
| ｐ21903 | 1 | 0 (0, 0) | 0 |  | NA | NA |
| ｐ21906 | 1 | 4.34 (0, 0) | 0 |  | NA | NA |
| ｐ21907 | 1 | 2.48 (0, 0) | 0 |  | NA | NA |
| ｐ21908 | 1 | 0.27 (0, 0) | 0 |  | NA | NA |
| ｐ21909 | 1 | 0.83 (0, 0) | 0 |  | NA | NA |
| ｐ21911 | 1 | 0.62 (0, 0) | 0 |  | NA | NA |
| ｐ21913 | 1 | 0.33 (0, 0) | 0 |  | NA | NA |
| ｐ21914 | 1 | 3.26 (0, 0) | 0 |  | NA | NA |
| ｐ21915 | 1 | 0.6 (0, 0) | 0 |  | NA | NA |
| ｐ21917 | 1 | 0.49 (0, 0) | 0 |  | NA | NA |
| ｐ21918 | 1 | 0 (0, 0) | 0 |  | NA | NA |
| ｐ21919 | 1 | 1.56 (0, 0) | 0 |  | NA | NA |
| ｐ21920 | 1 | 0.37 (0, 0) | 0 |  | NA | NA |
| ｐ21921 | 1 | 1.45 (0, 0) | 0 |  | NA | NA |
| ｐ21922 | 1 | 1.16 (0, 0) | 0 |  | NA | NA |
| ｐ21925 | 1 | 0.72 (0, 0) | 0 |  | NA | NA |
| ｐ21926 | 1 | 2.34 (0, 0) | 0 |  | NA | NA |
| ｐ21928 | 1 | 2.71 (0, 0) | 0 |  | NA | NA |
| ｐ21929 | 1 | 0.81 (0, 0) | 0 |  | NA | NA |
| ｐ21931 | 1 | 1.3 (0, 0) | 0 |  | NA | NA |
| ｐ21932 | 1 | 0.77 (0, 0) | 0 |  | NA | NA |
| ｐ21933 | 1 | 3.93 (0, 0) | 0 |  | NA | NA |
| ｐ21935 | 1 | 1.3 (0, 0) | 0 |  | NA | NA |
| ｐ21936 | 1 | 1.63 (0, 0) | 0 |  | NA | NA |
| ｐ21937 | 1 | 1.76 (0, 0) | 0 |  | NA | NA |
| ｐ21938 | 1 | 0.22 (0, 0) | 0 |  | NA | NA |
| ｐ21939 | 1 | 0.15 (0, 0) | 0 |  | NA | NA |
| ｐ21940 | 1 | 0.43 (0, 0) | 0 |  | NA | NA |
| ｐ21941 | 1 | 11.45 (0, 0) | 0 |  | NA | NA |
| ｐ21943 | 1 | 0.34 (0, 0) | 0 |  | NA | NA |
| ｐ21944 | 1 | 0.57 (0, 0) | 0 |  | NA | NA |
| ｐ21947 | 1 | 1.02 (0, 0) | 0 |  | NA | NA |
| ｐ21948 | 1 | 0.21 (0, 0) | 0 |  | NA | NA |
| ｐ21949 | 1 | 0.35 (0, 0) | 0 |  | NA | NA |
| ｐ21951 | 1 | 0.34 (0, 0) | 0 |  | NA | NA |
| ｐ21952 | 1 | 0.31 (0, 0) | 0 |  | NA | NA |
| ｐ21953 | 1 | 0.06 (0, 0) | 0 |  | NA | NA |
| ｐ21954 | 1 | 0.39 (0, 0) | 0 |  | NA | NA |
| ｐ21977 | 1 | 0.49 (0, 0) | 0 |  | NA | NA |
| ｐ21978 | 1 | 0.66 (0, 0) | 0 |  | NA | NA |
| ｐ21981 | 1 | 1.74 (0, 0) | 0 |  | NA | NA |
| ｐ21987 | 1 | 1.14 (0, 0) | 0 |  | NA | NA |
| ｐ22140 | 1 | 1.4 (0, 0) | 0 |  | NA | NA |
| ｐ22145 | 1 | 0.96 (0, 0) | 0 |  | NA | NA |
| ｐ22150 | 1 | 2.09 (0, 0) | 0 |  | NA | NA |
| ｐ22212 | 1 | 0.9 (0, 0) | 0 |  | NA | NA |
| ｐ22226 | 1 | 0.89 (0, 0) | 0 |  | NA | NA |
| ｐ22314 | 1 | 0.39 (0, 0) | 0 |  | NA | NA |
| ｐ22331 | 1 | 0.94 (0, 0) | 0 |  | NA | NA |
| ｐ22362 | 1 | 5.96 (0, 0) | 0 |  | NA | NA |
| ｐ22392 | 1 | 0 (0, 0) | 0 |  | NA | NA |
| ｐ22458 | 0 |  | 6 | 0 (0, 0) | NA | NA |
| ｐ22460 | 0 |  | 6 | 0 (0, 0) | NA | NA |
| ｐ22461 | 1 | 1.18 (0, 0) | 0 |  | NA | NA |
| ｐ22464 | 1 | 2.56 (0, 0) | 0 |  | NA | NA |
| ｐ22465 | 1 | 1.33 (0, 0) | 0 |  | NA | NA |
| ｐ22466 | 1 | 0.69 (0, 0) | 0 |  | NA | NA |
| ｐ22467 | 1 | 0.54 (0, 0) | 0 |  | NA | NA |
| ｐ22468 | 1 | 0.63 (0, 0) | 0 |  | NA | NA |
| ｐ22469 | 1 | 1.27 (0, 0) | 0 |  | NA | NA |
| ｐ22471 | 1 | 0.77 (0, 0) | 0 |  | NA | NA |
| ｐ22475 | 1 | 0.69 (0, 0) | 0 |  | NA | NA |
| ｐ22476 | 1 | 1.27 (0, 0) | 0 |  | NA | NA |
| ｐ22479 | 1 | 0.31 (0, 0) | 0 |  | NA | NA |
| ｐ22482 | 1 | 0.8 (0, 0) | 0 |  | NA | NA |
| ｐ22483 | 1 | 3.04 (0, 0) | 0 |  | NA | NA |
| ｐ22484 | 1 | 0.59 (0, 0) | 0 |  | NA | NA |
| ｐ22485 | 1 | 0.47 (0, 0) | 0 |  | NA | NA |
| ｐ22487 | 1 | 0.73 (0, 0) | 0 |  | NA | NA |
| ｐ22490 | 1 | 2.34 (0, 0) | 0 |  | NA | NA |
| ｐ22493 | 1 | 4.13 (0, 0) | 0 |  | NA | NA |
| ｐ22494 | 1 | 0.57 (0, 0) | 0 |  | NA | NA |
| ｐ22495 | 1 | 0.76 (0, 0) | 0 |  | NA | NA |
| ｐ22498 | 1 | 0.72 (0, 0) | 0 |  | NA | NA |
| ｐ22499 | 1 | 0.71 (0, 0) | 0 |  | NA | NA |
| ｐ22500 | 1 | 0.69 (0, 0) | 0 |  | NA | NA |
| ｐ22501 | 1 | 0.8 (0, 0) | 0 |  | NA | NA |
| ｐ22502 | 1 | 0.56 (0, 0) | 0 |  | NA | NA |
| ｐ22503 | 1 | 0.8 (0, 0) | 0 |  | NA | NA |
| ｐ22504 | 1 | 0.84 (0, 0) | 0 |  | NA | NA |
| ｐ22505 | 1 | 1.17 (0, 0) | 0 |  | NA | NA |
| ｐ22506 | 1 | 0.75 (0, 0) | 0 |  | NA | NA |
| ｐ22507 | 1 | 0.66 (0, 0) | 0 |  | NA | NA |
| ｐ22514 | 1 | 0.59 (0, 0) | 0 |  | NA | NA |
| ｐ22517 | 1 | 1.18 (0, 0) | 0 |  | NA | NA |
| ｐ22520 | 1 | 0.52 (0, 0) | 0 |  | NA | NA |
| ｐ22521 | 1 | 0.65 (0, 0) | 0 |  | NA | NA |
| ｐ22523 | 1 | 0.89 (0, 0) | 0 |  | NA | NA |
| ｐ22524 | 1 | 1.35 (0, 0) | 0 |  | NA | NA |
| ｐ22529 | 1 | 0.84 (0, 0) | 0 |  | NA | NA |
| ｐ22530 | 1 | 1.11 (0, 0) | 0 |  | NA | NA |
| ｐ22531 | 1 | 0.82 (0, 0) | 0 |  | NA | NA |
| ｐ22533 | 1 | 0.97 (0, 0) | 0 |  | NA | NA |
| ｐ22535 | 1 | 0.46 (0, 0) | 0 |  | NA | NA |
| ｐ22536 | 1 | 1.36 (0, 0) | 0 |  | NA | NA |
| ｐ22537 | 1 | 1.11 (0, 0) | 0 |  | NA | NA |
| ｐ22539 | 1 | 0.66 (0, 0) | 0 |  | NA | NA |
| ｐ22540 | 1 | 0.5 (0, 0) | 0 |  | NA | NA |
| ｐ22541 | 1 | 0.74 (0, 0) | 0 |  | NA | NA |
| ｐ22546 | 1 | 0.7 (0, 0) | 0 |  | NA | NA |
| ｐ22548 | 1 | 1.31 (0, 0) | 0 |  | NA | NA |
| ｐ22554 | 1 | 1.27 (0, 0) | 0 |  | NA | NA |
| ｐ22556 | 1 | 0.69 (0, 0) | 0 |  | NA | NA |
| ｐ22557 | 1 | 0.64 (0, 0) | 0 |  | NA | NA |
| ｐ22560 | 1 | 1.3 (0, 0) | 0 |  | NA | NA |
| ｐ22563 | 1 | 0.86 (0, 0) | 0 |  | NA | NA |
| ｐ22564 | 1 | 3.16 (0, 0) | 0 |  | NA | NA |
| ｐ22565 | 1 | 1.25 (0, 0) | 0 |  | NA | NA |
| ｐ22566 | 1 | 2.01 (0, 0) | 0 |  | NA | NA |
| ｐ22568 | 1 | 0.9 (0, 0) | 0 |  | NA | NA |
| ｐ22569 | 1 | 0.78 (0, 0) | 0 |  | NA | NA |
| ｐ22570 | 1 | 0.67 (0, 0) | 0 |  | NA | NA |
| ｐ22571 | 1 | 0.94 (0, 0) | 0 |  | NA | NA |
| ｐ22573 | 1 | 0.81 (0, 0) | 0 |  | NA | NA |
| ｐ22575 | 1 | 0.78 (0, 0) | 0 |  | NA | NA |
| ｐ22576 | 1 | 0 (0, 0) | 0 |  | NA | NA |
| ｐ22577 | 1 | 2.3 (0, 0) | 0 |  | NA | NA |
| ｐ22578 | 1 | 0.89 (0, 0) | 0 |  | NA | NA |
| ｐ22579 | 1 | 0.99 (0, 0) | 0 |  | NA | NA |
| ｐ22580 | 1 | 1.56 (0, 0) | 0 |  | NA | NA |
| ｐ22581 | 1 | 1.27 (0, 0) | 0 |  | NA | NA |
| ｐ22582 | 1 | 1.19 (0, 0) | 0 |  | NA | NA |
| ｐ22583 | 1 | 1.02 (0, 0) | 0 |  | NA | NA |
| ｐ22584 | 1 | 1.02 (0, 0) | 0 |  | NA | NA |
| ｐ22585 | 1 | 1.3 (0, 0) | 0 |  | NA | NA |
| ｐ22586 | 1 | 1.39 (0, 0) | 0 |  | NA | NA |
| ｐ22587 | 1 | 1.7 (0, 0) | 0 |  | NA | NA |
| ｐ22588 | 1 | 1.25 (0, 0) | 0 |  | NA | NA |
| ｐ22590 | 1 | 1.26 (0, 0) | 0 |  | NA | NA |
| ｐ22591 | 1 | 1.03 (0, 0) | 0 |  | NA | NA |
| ｐ22592 | 1 | 1.18 (0, 0) | 0 |  | NA | NA |
| ｐ22593 | 1 | 0.67 (0, 0) | 0 |  | NA | NA |
| ｐ22594 | 1 | 2.16 (0, 0) | 0 |  | NA | NA |
| ｐ22595 | 1 | 0.86 (0, 0) | 0 |  | NA | NA |
| ｐ22596 | 1 | 0.78 (0, 0) | 0 |  | NA | NA |
| ｐ22597 | 1 | 4.49 (0, 0) | 0 |  | NA | NA |
| ｐ22598 | 1 | 0.9 (0, 0) | 0 |  | NA | NA |
| ｐ22599 | 1 | 1.85 (0, 0) | 0 |  | NA | NA |
| ｐ22600 | 1 | 1.12 (0, 0) | 0 |  | NA | NA |
| ｐ22601 | 1 | 0.68 (0, 0) | 0 |  | NA | NA |
| ｐ22602 | 1 | 1.01 (0, 0) | 0 |  | NA | NA |
| ｐ22603 | 1 | 1.66 (0, 0) | 0 |  | NA | NA |
| ｐ22604 | 1 | 0.56 (0, 0) | 0 |  | NA | NA |
| ｐ22606 | 1 | 1.05 (0, 0) | 0 |  | NA | NA |
| ｐ22608 | 1 | 1 (0, 0) | 0 |  | NA | NA |
| ｐ22609 | 1 | 1.69 (0, 0) | 0 |  | NA | NA |
| ｐ22610 | 1 | 1.19 (0, 0) | 0 |  | NA | NA |
| ｐ22611 | 1 | 1.54 (0, 0) | 0 |  | NA | NA |
| ｐ22612 | 1 | 1.14 (0, 0) | 0 |  | NA | NA |
| ｐ22613 | 1 | 1.21 (0, 0) | 0 |  | NA | NA |
| ｐ22614 | 1 | 1.11 (0, 0) | 0 |  | NA | NA |
| ｐ22616 | 1 | 0.95 (0, 0) | 0 |  | NA | NA |
| ｐ22617 | 1 | 0.7 (0, 0) | 0 |  | NA | NA |
| ｐ22618 | 1 | 0.83 (0, 0) | 0 |  | NA | NA |
| ｐ22620 | 1 | 0.97 (0, 0) | 0 |  | NA | NA |
| ｐ22622 | 1 | 0.59 (0, 0) | 0 |  | NA | NA |
| ｐ22623 | 1 | 1.53 (0, 0) | 0 |  | NA | NA |
| ｐ22624 | 1 | 1.18 (0, 0) | 0 |  | NA | NA |
| ｐ22625 | 1 | 0.98 (0, 0) | 0 |  | NA | NA |
| ｐ22626 | 1 | 0.72 (0, 0) | 0 |  | NA | NA |
| ｐ22627 | 1 | 1.14 (0, 0) | 0 |  | NA | NA |
| ｐ22628 | 1 | 1.34 (0, 0) | 0 |  | NA | NA |
| ｐ22629 | 1 | 0.97 (0, 0) | 0 |  | NA | NA |
| ｐ22630 | 1 | 1.3 (0, 0) | 0 |  | NA | NA |
| ｐ22632 | 1 | 0.8 (0, 0) | 0 |  | NA | NA |
| ｐ22839 | 1 | 0.51 (0, 0) | 0 |  | NA | NA |
| ｐ22842 | 1 | 1.11 (0, 0) | 0 |  | NA | NA |
| ｐ22976 | 1 | 0.75 (0, 0) | 0 |  | NA | NA |
| ｐ22994 | 1 | 1.47 (0, 0) | 0 |  | NA | NA |
| ｐ23043 | 1 | 1.54 (0, 0) | 0 |  | NA | NA |
| ｐ23084 | 1 | 0.89 (0, 0) | 0 |  | NA | NA |

Abbreviations: PDAC, pancreatic ductal adenocarcinoma; CI, confidence interval; FC, fold change with respect to the mean value in healthy subjects; NA, not available.

The P-value was calculated using a two-tailed Student’s t test.

## Supplementary Table S3. Comparison of urinary protein fragments between healthy volunteers and patients with PDAC in the validation cohort

| Fragments | Healthy volunteers | | PDAC patients | | Log2 [FC] | -Log10 [P value] |
| --- | --- | --- | --- | --- | --- | --- |
|  | N | Mean (95%CI) | N | Mean (95%CI) |  |  |
| pep_no_13684 | 36 | 0.96 (0.88, 1.05) | 28 | 1.64 (1.35, 1.92) | 0.761 | 5.491 |
| pep_no_3781 | 1 | 5.23 (0, 0) | 3 | 0.93 (0.64, 1.23) | -2.487 | 2.987 |
| pep_no_399 | 36 | 1.3 (1.16, 1.44) | 28 | 1.75 (1.48, 2.02) | 0.427 | 2.664 |
| pep_no_254 | 34 | 0.81 (0.64, 0.97) | 27 | 1.31 (1.01, 1.62) | 0.700 | 2.593 |
| pep_no_12919 | 11 | 3.7 (1.03, 6.37) | 20 | 0.89 (0.72, 1.07) | -2.050 | 2.448 |
| pep_no_318 | 17 | 2.17 (1.75, 2.58) | 16 | 1.44 (1.19, 1.68) | -0.592 | 2.440 |
| pep_no_14815 | 36 | 0.71 (0.58, 0.85) | 28 | 1.11 (0.85, 1.38) | 0.643 | 2.390 |
| pep_no_3409 | 2 | 7.53 (-26.19, 41.25) | 6 | 1.44 (0.66, 2.23) | -2.383 | 2.359 |
| pep_no_943 | 15 | 0.52 (0.37, 0.67) | 14 | 0.94 (0.69, 1.2) | 0.854 | 2.357 |
| pep_no_661 | 36 | 0.68 (0.59, 0.77) | 28 | 0.93 (0.77, 1.09) | 0.446 | 2.275 |
| pep_no_12407 | 1 | 0.62 (0, 0) | 3 | 0.92 (0.87, 0.97) | 0.581 | 2.212 |
| pep_no_959 | 34 | 1.04 (0.8, 1.28) | 27 | 1.62 (1.26, 1.99) | 0.647 | 2.200 |
| pep_no_274 | 36 | 1.51 (1.09, 1.94) | 28 | 0.83 (0.7, 0.97) | -0.858 | 2.135 |
| pep_no_15400 | 7 | 0.45 (0.36, 0.55) | 21 | 0.8 (0.66, 0.95) | 0.825 | 2.038 |
| pep_no_13224 | 13 | 1.3 (0.96, 1.63) | 21 | 0.9 (0.76, 1.04) | -0.530 | 1.965 |
| pep_no_13311 | 8 | 1.24 (0.8, 1.67) | 11 | 1.96 (1.56, 2.36) | 0.663 | 1.850 |
| pep_no_4483 | 32 | 0.68 (0.33, 1.02) | 24 | 4.84 (0.9, 8.78) | 2.836 | 1.825 |
| pep_no_1236 | 33 | 1.32 (1.07, 1.58) | 19 | 0.88 (0.7, 1.06) | -0.598 | 1.791 |
| pep_no_19858 | 15 | 0.66 (0.46, 0.87) | 2 | 2.35 (-23.71, 28.41) | 1.825 | 1.785 |
| pep_no_106 | 14 | 2.7 (1.5, 3.89) | 13 | 1.19 (0.93, 1.45) | -1.177 | 1.777 |
| pep_no_4548 | 35 | 1.21 (1.06, 1.36) | 25 | 0.93 (0.76, 1.11) | -0.375 | 1.728 |
| pep_no_260 | 22 | 1.34 (1.09, 1.58) | 4 | 0.64 (0.4, 0.87) | -1.067 | 1.717 |
| pep_no_647 | 25 | 1.41 (1.17, 1.66) | 25 | 1.07 (0.9, 1.24) | -0.404 | 1.670 |
| pep_no_2577 | 8 | 3.8 (1.12, 6.48) | 11 | 1.36 (1.12, 1.6) | -1.485 | 1.666 |
| pep_no_12298 | 31 | 1.09 (1.01, 1.17) | 18 | 1.28 (1.1, 1.47) | 0.236 | 1.621 |
| pep_no_22928 | 9 | 0.59 (0.5, 0.69) | 3 | 1.05 (-0.23, 2.32) | 0.816 | 1.611 |
| pep_no_12985 | 6 | 1.3 (0.2, 2.4) | 18 | 0.64 (0.48, 0.81) | -1.016 | 1.604 |
| pep_no_24988 | 5 | 0.63 (0.45, 0.81) | 3 | 0.36 (0.21, 0.51) | -0.816 | 1.604 |
| pep_no_9554 | 16 | 0.7 (0.6, 0.81) | 16 | 0.92 (0.75, 1.09) | 0.387 | 1.585 |
| pep_no_6568 | 20 | 1.24 (0.98, 1.49) | 23 | 0.88 (0.68, 1.08) | -0.487 | 1.576 |
| pep_no_25295 | 1 | 3.04 (0, 0) | 3 | 1.6 (1.07, 2.12) | -0.930 | 1.569 |
| pep_no_49 | 2 | 7.14 (-45.3, 59.58) | 6 | 1.41 (0.82, 2) | -2.340 | 1.552 |
| pep_no_780 | 31 | 2.16 (1.77, 2.54) | 13 | 1.4 (0.86, 1.94) | -0.624 | 1.546 |
| pep_no_24934 | 1 | 2.11 (0, 0) | 3 | 0.86 (0.39, 1.33) | -1.294 | 1.533 |
| pep_no_14363 | 21 | 0.79 (0.62, 0.96) | 8 | 1.13 (0.88, 1.37) | 0.516 | 1.531 |
| pep_no_210 | 34 | 0.83 (0.73, 0.94) | 27 | 1.08 (0.86, 1.3) | 0.374 | 1.507 |
| pep_no_1269 | 29 | 1.44 (1.2, 1.67) | 12 | 1.03 (0.88, 1.18) | -0.479 | 1.494 |
| pep_no_3579 | 17 | 1.43 (0.75, 2.12) | 14 | 0.61 (0.4, 0.82) | -1.230 | 1.476 |
| pep_no_14972 | 22 | 1.25 (0.96, 1.53) | 21 | 0.91 (0.79, 1.03) | -0.451 | 1.476 |
| pep_no_987 | 34 | 1.11 (0.89, 1.33) | 27 | 0.83 (0.74, 0.93) | -0.411 | 1.453 |
| pep_no_12572 | 31 | 1.06 (0.89, 1.23) | 26 | 0.81 (0.65, 0.97) | -0.383 | 1.434 |
| pep_no_10234 | 16 | 0.93 (0.85, 1.02) | 7 | 1.13 (0.89, 1.38) | 0.281 | 1.426 |
| pep_no_11116 | 35 | 0.71 (0.53, 0.89) | 25 | 1.4 (0.65, 2.15) | 0.980 | 1.423 |
| pep_no_921 | 31 | 0.87 (0.67, 1.06) | 18 | 1.19 (0.96, 1.41) | 0.453 | 1.413 |
| pep_no_22691 | 1 | 3.06 (0, 0) | 3 | 1.59 (0.93, 2.25) | -0.947 | 1.391 |
| pep_no_22073 | 5 | 0.72 (0.55, 0.88) | 2 | 1.06 (-0.87, 2.98) | 0.561 | 1.335 |
| pep_no_4660 | 29 | 0.86 (0.75, 0.96) | 25 | 1.06 (0.88, 1.25) | 0.313 | 1.334 |
| pep_no_14610 | 5 | 0.57 (0.29, 0.85) | 4 | 1.26 (0.3, 2.21) | 1.135 | 1.312 |
| pep_no_27411 | 5 | 0.96 (0.63, 1.29) | 2 | 1.49 (-0.43, 3.42) | 0.639 | 1.262 |
| pep_no_163 | 26 | 0.88 (0.73, 1.03) | 27 | 1.2 (0.9, 1.5) | 0.448 | 1.254 |
| pep_no_3837 | 34 | 0.88 (0.78, 0.99) | 27 | 1.09 (0.88, 1.3) | 0.305 | 1.252 |
| pep_no_22992 | 2 | 1.02 (0.74, 1.3) | 6 | 1.32 (1.14, 1.5) | 0.371 | 1.242 |
| pep_no_12668 | 19 | 1.28 (0.8, 1.75) | 8 | 0.56 (0.19, 0.92) | -1.199 | 1.236 |
| pep_no_24893 | 2 | 1.33 (0.77, 1.9) | 6 | 0.97 (0.75, 1.19) | -0.466 | 1.233 |
| pep_no_25561 | 1 | 0.64 (0, 0) | 3 | 1.48 (1.02, 1.94) | 1.206 | 1.223 |
| pep_no_15144 | 19 | 0.83 (0.52, 1.13) | 5 | 2.12 (-1.33, 5.58) | 1.359 | 1.200 |
| pep_no_411 | 31 | 1.99 (1.51, 2.47) | 26 | 1.44 (1.12, 1.76) | -0.470 | 1.196 |
| pep_no_175 | 34 | 1.12 (0.93, 1.31) | 27 | 0.9 (0.77, 1.02) | -0.324 | 1.195 |
| pep_no_10415 | 28 | 0.94 (0.82, 1.06) | 16 | 1.15 (0.92, 1.38) | 0.297 | 1.182 |
| pep_no_514 | 36 | 0.74 (0.61, 0.87) | 28 | 0.95 (0.75, 1.15) | 0.364 | 1.180 |
| pep_no_432 | 36 | 1.12 (1, 1.23) | 28 | 0.97 (0.86, 1.08) | -0.205 | 1.178 |
| pep_no_74 | 29 | 1.56 (0.61, 2.52) | 24 | 0.6 (0.41, 0.79) | -1.373 | 1.157 |
| pep_no_22684 | 2 | 2.08 (-7.87, 12.02) | 6 | 1.15 (0.83, 1.46) | -0.858 | 1.134 |
| pep_no_15561 | 9 | 0.96 (0.79, 1.13) | 10 | 1.18 (0.98, 1.39) | 0.301 | 1.131 |
| pep_no_13745 | 33 | 0.73 (0.62, 0.85) | 24 | 0.96 (0.7, 1.23) | 0.394 | 1.128 |
| pep_no_160 | 23 | 1.29 (1.04, 1.54) | 14 | 0.98 (0.79, 1.17) | -0.400 | 1.128 |
| pep_no_18374 | 3 | 0.7 (0.02, 1.37) | 9 | 1.32 (0.93, 1.71) | 0.922 | 1.119 |
| pep_no_22511 | 4 | 1.93 (-0.85, 4.72) | 12 | 0.95 (0.69, 1.22) | -1.019 | 1.109 |
| pep_no_10394 | 29 | 1.11 (0.97, 1.25) | 20 | 1.31 (1.12, 1.51) | 0.241 | 1.105 |
| pep_no_1311 | 19 | 1.06 (0.81, 1.31) | 14 | 0.79 (0.65, 0.92) | -0.431 | 1.102 |
| pep_no_25004 | 4 | 0.77 (0.45, 1.09) | 12 | 1.19 (0.92, 1.47) | 0.634 | 1.077 |
| pep_no_4533 | 36 | 1.23 (1, 1.46) | 28 | 0.98 (0.83, 1.12) | -0.331 | 1.065 |
| pep_no_2357 | 36 | 1.2 (1.01, 1.38) | 28 | 0.99 (0.84, 1.14) | -0.278 | 1.058 |
| pep_no_47 | 27 | 2.35 (0.71, 3.99) | 26 | 0.92 (0.57, 1.26) | -1.354 | 1.045 |
| pep_no_24748 | 8 | 1.04 (0.6, 1.47) | 5 | 1.63 (0.85, 2.41) | 0.655 | 1.044 |
| pep_no_479 | 36 | 0.83 (0.48, 1.19) | 28 | 1.49 (0.73, 2.25) | 0.839 | 1.040 |
| pep_no_2418 | 23 | 1.37 (1.1, 1.64) | 20 | 1.09 (0.91, 1.28) | -0.330 | 1.040 |
| pep_no_25380 | 6 | 0.73 (0.57, 0.88) | 6 | 0.93 (0.7, 1.16) | 0.354 | 1.037 |
| pep_no_10051 | 5 | 0.83 (0.54, 1.12) | 15 | 1.23 (0.96, 1.5) | 0.578 | 1.032 |
| pep_no_10210 | 23 | 0.93 (0.82, 1.04) | 15 | 1.11 (0.88, 1.34) | 0.266 | 1.031 |
| pep_no_1091 | 5 | 0.74 (0.18, 1.3) | 2 | 1.48 (-1.5, 4.46) | 1.005 | 1.028 |
| pep_no_25585 | 4 | 0.76 (0.73, 0.8) | 7 | 1.13 (0.78, 1.49) | 0.566 | 1.026 |
| pep_no_108 | 19 | 1.93 (1.06, 2.79) | 20 | 1.22 (1.01, 1.43) | -0.656 | 1.010 |
| pep_no_14568 | 13 | 1.45 (1.21, 1.68) | 2 | 0.95 (0.61, 1.28) | -0.615 | 1.003 |
| pep_no_4452 | 34 | 1.12 (0.91, 1.33) | 22 | 0.87 (0.66, 1.07) | -0.367 | 0.994 |
| pep_no_25289 | 1 | 1.39 (0, 0) | 3 | 0.55 (-0.07, 1.17) | -1.330 | 0.989 |
| pep_no_27143 | 4 | 0.66 (0.35, 0.97) | 1 | 1.15 (0, 0) | 0.808 | 0.960 |
| pep_no_24616 | 3 | 1.72 (0.71, 2.72) | 9 | 1.16 (0.79, 1.54) | -0.561 | 0.959 |
| pep_no_20795 | 9 | 1.14 (0.73, 1.55) | 10 | 1.59 (1.15, 2.02) | 0.478 | 0.958 |
| pep_no_8512 | 8 | 0.8 (0.55, 1.05) | 1 | 1.37 (0, 0) | 0.781 | 0.953 |
| pep_no_2282 | 14 | 0.83 (0.54, 1.11) | 5 | 1.38 (0.18, 2.58) | 0.744 | 0.952 |
| pep_no_3824 | 8 | 2.95 (0.1, 5.8) | 11 | 1.23 (0.94, 1.52) | -1.264 | 0.949 |
| pep_no_27338 | 5 | 1.08 (0.51, 1.65) | 2 | 2.52 (-13.57, 18.61) | 1.224 | 0.945 |
| pep_no_27394 | 5 | 1.16 (0.89, 1.42) | 2 | 1.51 (-0.67, 3.68) | 0.383 | 0.943 |
| pep_no_1257 | 15 | 1.09 (0.76, 1.42) | 21 | 0.85 (0.72, 0.97) | -0.360 | 0.943 |
| pep_no_191 | 32 | 2.45 (1.28, 3.62) | 21 | 1.3 (0.96, 1.63) | -0.919 | 0.940 |
| pep_no_24331 | 1 | 1.8 (0, 0) | 3 | 1.08 (0.49, 1.66) | -0.743 | 0.933 |
| pep_no_1439 | 5 | 1.19 (0.87, 1.51) | 4 | 0.93 (0.67, 1.18) | -0.362 | 0.932 |
| pep_no_1320 | 22 | 1.29 (1.12, 1.47) | 23 | 1.14 (1.02, 1.25) | -0.188 | 0.926 |
| pep_no_221 | 22 | 1.12 (0.72, 1.51) | 10 | 1.91 (0.53, 3.28) | 0.773 | 0.923 |
| pep_no_15310 | 20 | 0.88 (0.72, 1.05) | 20 | 1.05 (0.91, 1.19) | 0.247 | 0.923 |
| pep_no_11304 | 4 | 0.84 (0.56, 1.11) | 7 | 1.03 (0.86, 1.2) | 0.301 | 0.919 |
| pep_no_25723 | 10 | 1.32 (1.08, 1.56) | 5 | 1.03 (0.71, 1.36) | -0.350 | 0.917 |
| pep_no_14086 | 20 | 0.53 (0.41, 0.64) | 19 | 0.66 (0.53, 0.78) | 0.315 | 0.914 |
| pep_no_27145 | 4 | 0.62 (0.28, 0.97) | 1 | 1.14 (0, 0) | 0.871 | 0.903 |
| pep_no_24864 | 3 | 0.64 (-0.44, 1.73) | 9 | 1.08 (0.79, 1.38) | 0.757 | 0.901 |
| pep_no_27334 | 5 | 0.8 (0.56, 1.05) | 2 | 2.04 (-13.77, 17.86) | 1.346 | 0.901 |
| pep_no_271 | 32 | 1.62 (1.19, 2.05) | 27 | 1.23 (0.99, 1.47) | -0.402 | 0.896 |
| pep_no_19357 | 2 | 1.43 (-8.63, 11.5) | 6 | 0.74 (0.57, 0.92) | -0.948 | 0.886 |
| pep_no_4234 | 36 | 1.04 (0.83, 1.24) | 28 | 1.31 (0.99, 1.63) | 0.335 | 0.879 |
| pep_no_24489 | 2 | 2.12 (-14.75, 19) | 6 | 0.91 (0.48, 1.34) | -1.220 | 0.877 |
| pep_no_14857 | 7 | 0.9 (0.79, 1) | 2 | 1.3 (-5.35, 7.96) | 0.537 | 0.870 |
| pep_no_11158 | 4 | 0.36 (-0.19, 0.91) | 1 | 1.14 (0, 0) | 1.676 | 0.868 |
| pep_no_26989 | 9 | 1.17 (0.91, 1.44) | 2 | 1.92 (-11.22, 15.07) | 0.712 | 0.862 |
| pep_no_8886 | 5 | 0.79 (0.56, 1.02) | 2 | 2.12 (-15.64, 19.87) | 1.422 | 0.860 |
| pep_no_4581 | 36 | 0.81 (0.65, 0.96) | 28 | 1.01 (0.77, 1.24) | 0.320 | 0.854 |
| pep_no_24807 | 3 | 0.2 (-0.15, 0.56) | 9 | 0.4 (0.25, 0.55) | 0.964 | 0.841 |
| pep_no_12611 | 5 | 0.7 (0.29, 1.11) | 2 | 2.09 (-16.71, 20.88) | 1.580 | 0.822 |
| pep_no_894 | 2 | 0.89 (-2.15, 3.93) | 6 | 1.39 (0.99, 1.79) | 0.644 | 0.813 |
| pep_no_26066 | 6 | 1.01 (0.8, 1.22) | 2 | 1.25 (0.85, 1.66) | 0.312 | 0.798 |
| pep_no_6030 | 29 | 1.03 (0.82, 1.23) | 7 | 0.73 (0.43, 1.02) | -0.502 | 0.794 |
| pep_no_22997 | 9 | 0.7 (0.47, 0.92) | 2 | 1.04 (-1.41, 3.5) | 0.579 | 0.788 |
| pep_no_25384 | 8 | 1.12 (0.81, 1.43) | 5 | 0.85 (0.58, 1.11) | -0.401 | 0.784 |
| pep_no_14830 | 21 | 1.2 (1.01, 1.4) | 16 | 1.39 (1.19, 1.59) | 0.210 | 0.782 |
| pep_no_27368 | 5 | 0.84 (0.57, 1.11) | 2 | 1.14 (-1.14, 3.43) | 0.446 | 0.773 |
| pep_no_15078 | 36 | 0.94 (0.68, 1.2) | 28 | 1.2 (0.93, 1.46) | 0.347 | 0.770 |
| pep_no_11456 | 36 | 0.78 (0.62, 0.95) | 28 | 1 (0.71, 1.28) | 0.344 | 0.769 |
| pep_no_1323 | 9 | 0.94 (0.74, 1.15) | 8 | 0.79 (0.66, 0.92) | -0.257 | 0.765 |
| pep_no_5102 | 5 | 1.21 (0.78, 1.65) | 2 | 2.02 (-8.49, 12.53) | 0.735 | 0.756 |
| pep_no_5221 | 7 | 1.52 (0.2, 2.84) | 8 | 0.75 (0.34, 1.16) | -1.016 | 0.756 |
| pep_no_7142 | 25 | 1.12 (0.89, 1.35) | 21 | 0.93 (0.77, 1.09) | -0.273 | 0.755 |
| pep_no_25237 | 1 | 0.87 (0, 0) | 3 | 1.17 (0.85, 1.5) | 0.439 | 0.753 |
| pep_no_27406 | 5 | 1.1 (0.73, 1.47) | 2 | 1.69 (-5.6, 8.98) | 0.622 | 0.752 |
| pep_no_27284 | 5 | 1.33 (0.01, 2.64) | 2 | 5.59 (-57.3, 68.48) | 2.077 | 0.745 |
| pep_no_1129 | 9 | 0.78 (0.52, 1.03) | 4 | 1.03 (0.74, 1.32) | 0.408 | 0.741 |
| pep_no_868 | 36 | 1.14 (0.91, 1.37) | 28 | 0.94 (0.79, 1.1) | -0.274 | 0.741 |
| pep_no_1435 | 32 | 1.17 (1.07, 1.28) | 16 | 1.3 (1.12, 1.47) | 0.148 | 0.740 |
| pep_no_26078 | 15 | 0.77 (0.6, 0.94) | 4 | 1.26 (-0.9, 3.43) | 0.717 | 0.737 |
| pep_no_1321 | 5 | 1.03 (0.68, 1.38) | 2 | 2.15 (-14.59, 18.88) | 1.060 | 0.731 |
| pep_no_118 | 25 | 0.96 (0.6, 1.33) | 13 | 0.62 (0.42, 0.82) | -0.635 | 0.719 |
| pep_no_14779 | 36 | 0.94 (0.79, 1.09) | 28 | 0.78 (0.56, 1) | -0.282 | 0.715 |
| pep_no_11672 | 6 | 0.88 (0.67, 1.09) | 1 | 1.21 (0, 0) | 0.450 | 0.710 |
| pep_no_474 | 10 | 1.07 (0.86, 1.28) | 4 | 1.46 (0.18, 2.73) | 0.440 | 0.705 |
| pep_no_23691 | 4 | 1.18 (0.46, 1.91) | 4 | 2.75 (-0.62, 6.12) | 1.218 | 0.705 |
| pep_no_68 | 15 | 1.06 (0.56, 1.55) | 3 | 0.35 (-0.19, 0.88) | -1.601 | 0.693 |
| pep_no_26198 | 11 | 1.38 (0.91, 1.86) | 3 | 0.81 (0.12, 1.49) | -0.781 | 0.692 |
| pep_no_7173 | 5 | 0.78 (0.5, 1.06) | 2 | 1.23 (-4.88, 7.33) | 0.651 | 0.692 |
| pep_no_25463 | 1 | 0.74 (0, 0) | 3 | 1.38 (0.63, 2.14) | 0.910 | 0.687 |
| pep_no_15351 | 16 | 1.12 (0.94, 1.3) | 5 | 1.32 (1.2, 1.44) | 0.241 | 0.683 |
| pep_no_3845 | 7 | 1.8 (0.02, 3.57) | 8 | 0.89 (0.6, 1.18) | -1.015 | 0.678 |
| pep_no_5855 | 31 | 0.85 (0.74, 0.97) | 25 | 0.97 (0.81, 1.13) | 0.188 | 0.675 |
| pep_no_27256 | 5 | 1.14 (0.38, 1.9) | 2 | 3.2 (-30.12, 36.53) | 1.487 | 0.662 |
| pep_no_460 | 36 | 0.99 (0.91, 1.07) | 28 | 1.07 (0.97, 1.17) | 0.109 | 0.659 |
| pep_no_24834 | 19 | 1.01 (0.65, 1.37) | 8 | 0.65 (0.26, 1.04) | -0.628 | 0.654 |
| pep_no_12477 | 34 | 0.96 (0.77, 1.15) | 27 | 1.12 (0.94, 1.29) | 0.220 | 0.647 |
| pep_no_21207 | 1 | 0.94 (0, 0) | 3 | 0.69 (0.39, 1) | -0.433 | 0.645 |
| pep_no_2160 | 3 | 1.27 (-0.31, 2.84) | 9 | 0.95 (0.75, 1.16) | -0.412 | 0.635 |
| pep_no_1237 | 14 | 1.18 (0.99, 1.37) | 14 | 1.01 (0.78, 1.25) | -0.221 | 0.614 |
| pep_no_20800 | 5 | 0.72 (0.43, 1.01) | 4 | 1.01 (0.3, 1.72) | 0.490 | 0.614 |
| pep_no_1340 | 17 | 1.08 (0.77, 1.39) | 2 | 0.55 (0.46, 0.65) | -0.967 | 0.612 |
| pep_no_14531 | 36 | 0.91 (0.8, 1.03) | 28 | 1.02 (0.86, 1.19) | 0.166 | 0.610 |
| pep_no_478 | 8 | 1.05 (0.76, 1.34) | 5 | 1.43 (0.46, 2.41) | 0.447 | 0.608 |
| pep_no_14745 | 34 | 1.04 (0.75, 1.34) | 27 | 0.8 (0.51, 1.09) | -0.380 | 0.606 |
| pep_no_11187 | 20 | 0.84 (0.67, 1) | 17 | 0.97 (0.79, 1.15) | 0.218 | 0.602 |
| pep_no_12620 | 5 | 1.17 (0.78, 1.56) | 2 | 1.77 (-8.02, 11.57) | 0.600 | 0.591 |
| pep_no_15126 | 5 | 1.16 (0.94, 1.38) | 2 | 1.91 (-11.98, 15.81) | 0.722 | 0.585 |
| pep_no_15358 | 29 | 0.75 (0.56, 0.95) | 20 | 0.97 (0.59, 1.35) | 0.365 | 0.584 |
| pep_no_828 | 23 | 1.21 (1.09, 1.34) | 12 | 1.1 (0.95, 1.25) | -0.139 | 0.583 |
| pep_no_25941 | 7 | 0.59 (0.29, 0.9) | 4 | 0.91 (0.01, 1.81) | 0.619 | 0.581 |
| pep_no_14982 | 18 | 0.72 (0.53, 0.9) | 21 | 0.58 (0.41, 0.76) | -0.307 | 0.580 |
| pep_no_19778 | 10 | 0.83 (0.65, 1.01) | 7 | 0.97 (0.75, 1.19) | 0.225 | 0.579 |
| pep_no_24519 | 1 | 0.64 (0, 0) | 3 | 0.95 (0.52, 1.38) | 0.562 | 0.576 |
| pep_no_27333 | 5 | 0.81 (0.59, 1.03) | 2 | 1.15 (-4.58, 6.88) | 0.509 | 0.575 |
| pep_no_22525 | 3 | 1.01 (0.62, 1.4) | 9 | 1.5 (0.96, 2.03) | 0.567 | 0.570 |
| pep_no_26195 | 6 | 1.73 (0.72, 2.74) | 1 | 0.44 (0, 0) | -1.978 | 0.569 |
| pep_no_14887 | 6 | 0.72 (0.47, 0.97) | 1 | 1.04 (0, 0) | 0.523 | 0.564 |
| pep_no_14334 | 6 | 1.63 (0.35, 2.91) | 5 | 2.98 (-0.14, 6.1) | 0.868 | 0.564 |
| pep_no_27238 | 5 | 1.33 (1.08, 1.58) | 2 | 1.54 (-0.62, 3.71) | 0.217 | 0.563 |
| pep_no_3355 | 23 | 1.39 (1.02, 1.77) | 19 | 1.14 (0.88, 1.4) | -0.290 | 0.562 |
| pep_no_26 | 16 | 1.18 (0.26, 2.11) | 5 | 0.3 (-0.03, 0.62) | -2.003 | 0.557 |
| pep_no_25871 | 1 | 0.45 (0, 0) | 3 | 0.92 (0.24, 1.61) | 1.028 | 0.555 |
| pep_no_22010 | 13 | 0.75 (0.51, 0.99) | 21 | 1.02 (0.65, 1.39) | 0.443 | 0.555 |
| pep_no_24816 | 24 | 0.86 (0.71, 1.01) | 24 | 0.97 (0.83, 1.11) | 0.172 | 0.549 |
| pep_no_27385 | 5 | 0.76 (0.53, 1) | 2 | 1.19 (-6.73, 9.11) | 0.646 | 0.546 |
| pep_no_819 | 31 | 0.98 (0.8, 1.16) | 13 | 0.82 (0.61, 1.03) | -0.258 | 0.545 |
| pep_no_407 | 36 | 1.02 (0.88, 1.17) | 28 | 0.93 (0.82, 1.03) | -0.147 | 0.545 |
| pep_no_19998 | 5 | 0.89 (0.69, 1.09) | 2 | 1.24 (-5.18, 7.66) | 0.478 | 0.541 |
| pep_no_10601 | 7 | 1.27 (0.79, 1.76) | 10 | 2.35 (0.54, 4.15) | 0.882 | 0.538 |
| pep_no_15984 | 12 | 0.72 (0.56, 0.89) | 7 | 0.96 (0.32, 1.61) | 0.413 | 0.538 |
| pep_no_1270 | 16 | 0.94 (0.58, 1.29) | 12 | 1.32 (0.56, 2.08) | 0.491 | 0.535 |
| pep_no_396 | 5 | 1.13 (0.65, 1.61) | 2 | 1.91 (-12.49, 16.3) | 0.753 | 0.528 |
| pep_no_7849 | 31 | 0.99 (0.91, 1.08) | 13 | 1.08 (0.89, 1.28) | 0.125 | 0.526 |
| pep_no_2417 | 15 | 0.82 (0.56, 1.07) | 22 | 1.04 (0.71, 1.38) | 0.355 | 0.519 |
| pep_no_58 | 5 | 0.22 (0.01, 0.43) | 2 | 0.07 (-0.81, 0.95) | -1.668 | 0.516 |
| pep_no_1157 | 10 | 1.05 (0.7, 1.4) | 6 | 1.3 (0.88, 1.72) | 0.306 | 0.504 |
| pep_no_6004 | 8 | 0.9 (0.63, 1.17) | 6 | 1.12 (0.64, 1.6) | 0.312 | 0.504 |
| pep_no_21075 | 3 | 0.76 (0.37, 1.15) | 1 | 0.52 (0, 0) | -0.546 | 0.503 |
| pep_no_17106 | 14 | 0.87 (0.68, 1.05) | 14 | 1.16 (0.57, 1.75) | 0.421 | 0.502 |
| pep_no_13390 | 16 | 1.13 (0.47, 1.8) | 5 | 0.54 (0.08, 1.01) | -1.067 | 0.502 |
| pep_no_571 | 36 | 1.07 (0.85, 1.29) | 28 | 0.93 (0.79, 1.07) | -0.203 | 0.501 |
| pep_no_15092 | 7 | 0.92 (0.39, 1.45) | 3 | 1.99 (-4.81, 8.78) | 1.107 | 0.495 |
| pep_no_13085 | 3 | 0.89 (0.57, 1.21) | 9 | 1.01 (0.87, 1.16) | 0.188 | 0.494 |
| pep_no_1272 | 5 | 0.75 (0.59, 0.91) | 2 | 0.91 (-1.82, 3.65) | 0.284 | 0.493 |
| pep_no_421 | 14 | 1.32 (1.02, 1.61) | 4 | 1.6 (0.95, 2.24) | 0.280 | 0.489 |
| pep_no_7732 | 3 | 2.65 (-1.52, 6.82) | 3 | 1.54 (0.64, 2.45) | -0.779 | 0.485 |
| pep_no_498 | 36 | 0.96 (0.73, 1.2) | 28 | 1.15 (0.84, 1.46) | 0.252 | 0.483 |
| pep_no_25594 | 7 | 0.45 (-0.04, 0.94) | 9 | 1.08 (-0.12, 2.28) | 1.257 | 0.483 |
| pep_no_26877 | 13 | 0.75 (0.63, 0.87) | 2 | 0.91 (-1.38, 3.19) | 0.275 | 0.483 |
| pep_no_24869 | 6 | 1.11 (0.76, 1.46) | 6 | 1.32 (0.94, 1.69) | 0.245 | 0.482 |
| pep_no_12614 | 5 | 0.85 (0.71, 1) | 2 | 1.27 (-7.75, 10.28) | 0.572 | 0.481 |
| pep_no_10001 | 10 | 1.19 (0.47, 1.91) | 5 | 2.48 (-2.42, 7.38) | 1.060 | 0.477 |
| pep_no_10465 | 34 | 1.11 (1, 1.23) | 22 | 1.04 (0.94, 1.13) | -0.105 | 0.468 |
| pep_no_4942 | 36 | 1.3 (0.97, 1.63) | 28 | 1.07 (0.75, 1.4) | -0.271 | 0.468 |
| pep_no_27287 | 5 | 0.99 (0.71, 1.26) | 2 | 1.43 (-8.04, 10.91) | 0.541 | 0.466 |
| pep_no_26717 | 3 | 1.47 (1.06, 1.88) | 1 | 1.24 (0, 0) | -0.250 | 0.464 |
| pep_no_20734 | 10 | 1.34 (0.66, 2.03) | 7 | 0.97 (0.59, 1.34) | -0.475 | 0.462 |
| pep_no_20246 | 4 | 0.91 (0.46, 1.35) | 6 | 1.09 (0.79, 1.4) | 0.268 | 0.460 |
| pep_no_27160 | 4 | 0.9 (0.67, 1.13) | 1 | 0.72 (0, 0) | -0.321 | 0.458 |
| pep_no_25578 | 6 | 1.83 (-0.07, 3.74) | 5 | 1.02 (0.62, 1.42) | -0.848 | 0.454 |
| pep_no_23770 | 9 | 0.8 (-0.23, 1.84) | 15 | 0.47 (0.34, 0.61) | -0.767 | 0.447 |
| pep_no_25013 | 7 | 0.95 (0.62, 1.28) | 10 | 1.21 (0.74, 1.69) | 0.350 | 0.446 |
| pep_no_25583 | 6 | 0.76 (0.55, 0.97) | 7 | 0.89 (0.64, 1.14) | 0.226 | 0.445 |
| pep_no_27290 | 5 | 1.04 (0.83, 1.25) | 2 | 1.46 (-8.13, 11.05) | 0.491 | 0.443 |
| pep_no_276 | 9 | 0.85 (0.62, 1.09) | 2 | 0.64 (-1.22, 2.49) | -0.421 | 0.436 |
| pep_no_17185 | 2 | 1.25 (-3.99, 6.49) | 6 | 0.99 (0.74, 1.25) | -0.333 | 0.434 |
| pep_no_27378 | 5 | 1.56 (0.93, 2.2) | 2 | 2.29 (-12.77, 17.35) | 0.551 | 0.434 |
| pep_no_46 | 20 | 0.72 (0.2, 1.24) | 5 | 0.25 (-0.03, 0.54) | -1.504 | 0.434 |
| pep_no_27183 | 5 | 0.5 (0.04, 0.95) | 2 | 0.22 (-1.06, 1.5) | -1.171 | 0.434 |
| pep_no_528 | 36 | 0.92 (0.77, 1.07) | 28 | 0.83 (0.68, 0.97) | -0.154 | 0.430 |
| pep_no_15899 | 14 | 0.96 (0.82, 1.1) | 17 | 1.09 (0.85, 1.34) | 0.181 | 0.430 |
| pep_no_2472 | 36 | 0.92 (0.81, 1.04) | 28 | 0.86 (0.76, 0.95) | -0.112 | 0.427 |
| pep_no_9574 | 13 | 1.01 (0.77, 1.25) | 3 | 1.3 (-0.74, 3.34) | 0.356 | 0.426 |
| pep_no_166 | 34 | 1.57 (0.69, 2.44) | 27 | 1.13 (0.95, 1.31) | -0.473 | 0.425 |
| pep_no_22849 | 22 | 0.92 (0.79, 1.06) | 5 | 1.07 (0.45, 1.7) | 0.222 | 0.425 |
| pep_no_24424 | 17 | 1.11 (0.83, 1.39) | 10 | 0.94 (0.7, 1.18) | -0.245 | 0.424 |
| pep_no_79 | 26 | 1.31 (0.31, 2.31) | 15 | 0.72 (0.31, 1.14) | -0.854 | 0.420 |
| pep_no_26178 | 6 | 1.06 (0.29, 1.83) | 1 | 0.3 (0, 0) | -1.822 | 0.419 |
| pep_no_25006 | 19 | 1.13 (0.93, 1.33) | 13 | 1.31 (0.86, 1.77) | 0.217 | 0.418 |
| pep_no_22154 | 2 | 1.03 (-3.83, 5.9) | 6 | 1.68 (0.74, 2.62) | 0.703 | 0.416 |
| pep_no_20442 | 5 | 1.1 (0.89, 1.31) | 2 | 1.24 (-0.36, 2.83) | 0.167 | 0.414 |
| pep_no_10645 | 36 | 0.73 (0.63, 0.83) | 28 | 0.8 (0.67, 0.94) | 0.132 | 0.411 |
| pep_no_4252 | 36 | 0.56 (0.38, 0.74) | 28 | 0.66 (0.5, 0.83) | 0.248 | 0.409 |
| pep_no_13742 | 20 | 1.77 (0.24, 3.3) | 5 | 0.49 (0.17, 0.81) | -1.857 | 0.399 |
| pep_no_303 | 10 | 1.3 (0.73, 1.88) | 4 | 2.04 (-1.91, 5.99) | 0.647 | 0.399 |
| pep_no_22695 | 2 | 1.05 (-1.02, 3.12) | 1 | 1.44 (0, 0) | 0.452 | 0.396 |
| pep_no_12632 | 6 | 0.94 (0.67, 1.2) | 1 | 1.19 (0, 0) | 0.338 | 0.394 |
| pep_no_25448 | 2 | 1.27 (-0.12, 2.66) | 6 | 0.97 (0.49, 1.44) | -0.394 | 0.393 |
| pep_no_835 | 28 | 1.36 (1.18, 1.54) | 15 | 1.24 (1.02, 1.46) | -0.133 | 0.392 |
| pep_no_71 | 10 | 0.35 (0.17, 0.52) | 4 | 0.23 (-0.01, 0.48) | -0.575 | 0.391 |
| pep_no_15243 | 7 | 1.68 (0.82, 2.54) | 3 | 1.18 (0.42, 1.95) | -0.507 | 0.390 |
| pep_no_67 | 20 | 1.51 (0.09, 2.93) | 5 | 0.34 (-0.01, 0.7) | -2.133 | 0.389 |
| pep_no_7209 | 5 | 0.88 (0.46, 1.31) | 2 | 1.27 (-7.13, 9.68) | 0.527 | 0.387 |
| pep_no_6188 | 36 | 1 (0.81, 1.19) | 28 | 0.89 (0.71, 1.07) | -0.166 | 0.385 |
| pep_no_13072 | 1 | 0.94 (0, 0) | 3 | 1.38 (0.46, 2.31) | 0.555 | 0.385 |
| pep_no_2169 | 8 | 0.7 (0.12, 1.27) | 5 | 1.13 (-0.35, 2.61) | 0.706 | 0.383 |
| pep_no_27248 | 5 | 1.69 (0.67, 2.72) | 2 | 2.67 (-19.34, 24.69) | 0.660 | 0.382 |
| pep_no_3350 | 34 | 1.17 (0.83, 1.51) | 22 | 1.41 (0.86, 1.96) | 0.273 | 0.379 |
| pep_no_11382 | 11 | 1.14 (0.98, 1.3) | 10 | 1.25 (0.99, 1.51) | 0.133 | 0.375 |
| pep_no_24979 | 1 | 0.66 (0, 0) | 3 | 1.64 (-0.48, 3.76) | 1.321 | 0.373 |
| pep_no_3594 | 5 | 0.14 (-0.04, 0.31) | 2 | 0.04 (-0.23, 0.32) | -1.624 | 0.371 |
| pep_no_24342 | 6 | 2.28 (0.58, 3.98) | 1 | 0.77 (0, 0) | -1.567 | 0.369 |
| pep_no_12270 | 36 | 1.06 (0.9, 1.22) | 28 | 1.18 (0.89, 1.48) | 0.160 | 0.368 |
| pep_no_12621 | 7 | 0.96 (0.67, 1.26) | 3 | 1.26 (-0.88, 3.39) | 0.384 | 0.367 |
| pep_no_21459 | 2 | 0.8 (0.1, 1.5) | 1 | 0.92 (0, 0) | 0.198 | 0.364 |
| pep_no_6094 | 8 | 0.63 (0.23, 1.03) | 1 | 1.05 (0, 0) | 0.740 | 0.361 |
| pep_no_16575 | 8 | 0.85 (0.66, 1.04) | 6 | 0.95 (0.69, 1.21) | 0.165 | 0.361 |
| pep_no_1301 | 29 | 0.65 (0.47, 0.83) | 25 | 0.81 (0.42, 1.2) | 0.310 | 0.357 |
| pep_no_7740 | 34 | 1.07 (0.96, 1.18) | 28 | 1.14 (0.98, 1.31) | 0.095 | 0.356 |
| pep_no_12760 | 36 | 0.91 (0.7, 1.11) | 28 | 1.05 (0.71, 1.38) | 0.208 | 0.353 |
| pep_no_10921 | 6 | 1.12 (0.51, 1.73) | 12 | 1.31 (1.04, 1.58) | 0.223 | 0.352 |
| pep_no_25386 | 8 | 0.93 (0.32, 1.55) | 12 | 0.75 (0.58, 0.93) | -0.308 | 0.348 |
| pep_no_3389 | 12 | 1.13 (0.86, 1.4) | 7 | 1.28 (0.91, 1.65) | 0.183 | 0.347 |
| pep_no_6617 | 21 | 0.75 (0.59, 0.9) | 14 | 0.88 (0.5, 1.25) | 0.227 | 0.347 |
| pep_no_26174 | 6 | 1.96 (0.93, 3) | 1 | 1.1 (0, 0) | -0.843 | 0.344 |
| pep_no_147 | 30 | 0.86 (0.66, 1.06) | 15 | 0.74 (0.46, 1.02) | -0.229 | 0.343 |
| pep_no_12940 | 23 | 1.21 (1.11, 1.31) | 5 | 1.12 (0.87, 1.38) | -0.106 | 0.339 |
| pep_no_13796 | 14 | 0.73 (0.54, 0.92) | 17 | 0.82 (0.65, 0.99) | 0.166 | 0.335 |
| pep_no_15313 | 5 | 1.13 (0.88, 1.38) | 2 | 1.01 (0.31, 1.71) | -0.161 | 0.332 |
| pep_no_25 | 13 | 1.48 (0.01, 2.94) | 13 | 0.95 (0.48, 1.43) | -0.632 | 0.331 |
| pep_no_7005 | 5 | 0.9 (0.48, 1.33) | 2 | 0.69 (-1.02, 2.4) | -0.381 | 0.331 |
| pep_no_5590 | 15 | 6.13 (4.32, 7.94) | 17 | 4.88 (1.87, 7.89) | -0.330 | 0.328 |
| pep_no_19371 | 14 | 0.65 (-0.06, 1.37) | 5 | 0.24 (-0.01, 0.48) | -1.466 | 0.327 |
| pep_no_25392 | 1 | 1.36 (0, 0) | 3 | 1.14 (0.6, 1.68) | -0.256 | 0.326 |
| pep_no_17923 | 4 | 1.18 (0.71, 1.64) | 1 | 0.91 (0, 0) | -0.372 | 0.321 |
| pep_no_1053 | 16 | 2.07 (0.79, 3.34) | 22 | 1.65 (1.16, 2.14) | -0.324 | 0.321 |
| pep_no_249 | 36 | 0.72 (0.52, 0.92) | 28 | 0.96 (0.21, 1.71) | 0.417 | 0.317 |
| pep_no_95 | 34 | 0.79 (0.69, 0.9) | 27 | 0.87 (0.65, 1.09) | 0.136 | 0.316 |
| pep_no_26087 | 6 | 4.01 (0.56, 7.46) | 1 | 1.33 (0, 0) | -1.589 | 0.315 |
| pep_no_2844 | 7 | 1.52 (0.87, 2.17) | 2 | 1.94 (-4.51, 8.38) | 0.346 | 0.312 |
| pep_no_24562 | 2 | 1.06 (-0.38, 2.49) | 6 | 0.96 (0.78, 1.14) | -0.142 | 0.305 |
| pep_no_2489 | 36 | 0.95 (0.8, 1.1) | 28 | 1.03 (0.87, 1.19) | 0.108 | 0.303 |
| pep_no_9502 | 5 | 1.1 (0.32, 1.89) | 2 | 1.45 (1.43, 1.47) | 0.392 | 0.302 |
| pep_no_1038 | 26 | 1.01 (0.78, 1.24) | 15 | 0.89 (0.66, 1.12) | -0.175 | 0.301 |
| pep_no_15578 | 18 | 1.13 (0.8, 1.45) | 11 | 1.29 (0.89, 1.69) | 0.195 | 0.298 |
| pep_no_27371 | 5 | 1.43 (0.47, 2.39) | 2 | 2.04 (-12.62, 16.69) | 0.510 | 0.297 |
| pep_no_4528 | 5 | 0.27 (0.11, 0.44) | 4 | 0.34 (0.13, 0.54) | 0.288 | 0.297 |
| pep_no_220 | 36 | 0.73 (0.52, 0.94) | 28 | 0.89 (0.4, 1.39) | 0.287 | 0.295 |
| pep_no_24037 | 7 | 0.71 (0.44, 0.98) | 1 | 0.49 (0, 0) | -0.525 | 0.289 |
| pep_no_7284 | 5 | 1.49 (0.46, 2.52) | 2 | 2.03 (-9.2, 13.26) | 0.447 | 0.285 |
| pep_no_25954 | 1 | 0.83 (0, 0) | 3 | 1.06 (0.41, 1.7) | 0.356 | 0.284 |
| pep_no_12636 | 11 | 1.05 (0.89, 1.22) | 3 | 1.19 (-0.28, 2.67) | 0.182 | 0.281 |
| pep_no_332 | 1 | 2.98 (0, 0) | 3 | 5.89 (-2.27, 14.04) | 0.981 | 0.281 |
| pep_no_4401 | 36 | 1.08 (0.84, 1.32) | 28 | 1.2 (0.92, 1.47) | 0.145 | 0.281 |
| pep_no_22675 | 6 | 1.69 (-0.27, 3.65) | 1 | 0.32 (0, 0) | -2.424 | 0.280 |
| pep_no_9730 | 5 | 1.1 (0.73, 1.47) | 2 | 1.32 (-4.44, 7.09) | 0.268 | 0.280 |
| pep_no_884 | 17 | 1.45 (1.2, 1.71) | 8 | 1.64 (0.79, 2.5) | 0.180 | 0.278 |
| pep_no_14570 | 34 | 0.72 (0.57, 0.87) | 27 | 0.82 (0.51, 1.14) | 0.191 | 0.278 |
| pep_no_25892 | 1 | 1.31 (0, 0) | 3 | 1.76 (0.48, 3.05) | 0.427 | 0.277 |
| pep_no_27379 | 5 | 1.46 (1.15, 1.77) | 2 | 1.72 (-6.35, 9.78) | 0.235 | 0.275 |
| pep_no_26181 | 6 | 4.89 (-0.61, 10.39) | 1 | 1.09 (0, 0) | -2.169 | 0.275 |
| pep_no_4592 | 10 | 1.08 (0.79, 1.38) | 4 | 1.23 (0.74, 1.72) | 0.184 | 0.274 |
| pep_no_26072 | 6 | 1.01 (0.79, 1.24) | 1 | 0.86 (0, 0) | -0.243 | 0.274 |
| pep_no_25034 | 1 | 0.5 (0, 0) | 3 | 0.86 (-0.18, 1.91) | 0.786 | 0.272 |
| pep_no_190 | 28 | 0.87 (0.65, 1.1) | 22 | 0.79 (0.63, 0.94) | -0.151 | 0.270 |
| pep_no_3454 | 20 | 1.12 (0.98, 1.26) | 12 | 1.05 (0.89, 1.22) | -0.087 | 0.269 |
| pep_no_141 | 34 | 0.83 (0.53, 1.12) | 27 | 0.7 (0.38, 1.01) | -0.248 | 0.267 |
| pep_no_17643 | 1 | 0.75 (0, 0) | 3 | 1.24 (-0.21, 2.7) | 0.723 | 0.264 |
| pep_no_22774 | 1 | 0.65 (0, 0) | 3 | 0.84 (0.26, 1.43) | 0.380 | 0.261 |
| pep_no_26177 | 15 | 0.82 (0.3, 1.34) | 3 | 0.48 (-0.52, 1.49) | -0.771 | 0.256 |
| pep_no_320 | 36 | 1.05 (0.94, 1.16) | 28 | 0.99 (0.85, 1.14) | -0.074 | 0.255 |
| pep_no_12741 | 10 | 1.35 (0.84, 1.87) | 14 | 1.21 (0.97, 1.46) | -0.155 | 0.250 |
| pep_no_8595 | 3 | 1.09 (-0.4, 2.58) | 9 | 0.98 (0.88, 1.07) | -0.160 | 0.248 |
| pep_no_24727 | 3 | 0.86 (0.45, 1.27) | 9 | 0.96 (0.76, 1.16) | 0.156 | 0.247 |
| pep_no_27535 | 5 | 0.95 (0.18, 1.71) | 2 | 0.66 (-1.6, 2.92) | -0.523 | 0.246 |
| pep_no_27352 | 5 | 1.31 (0.49, 2.14) | 2 | 1.65 (-4.41, 7.72) | 0.332 | 0.244 |
| pep_no_22516 | 3 | 1.04 (-0.23, 2.32) | 4 | 0.83 (0.15, 1.51) | -0.329 | 0.242 |
| pep_no_893 | 14 | 1.21 (1.04, 1.38) | 6 | 1.29 (1.05, 1.52) | 0.088 | 0.242 |
| pep_no_3290 | 22 | 0.98 (0.83, 1.14) | 24 | 1.04 (0.89, 1.19) | 0.084 | 0.242 |
| pep_no_18168 | 5 | 1.5 (0.95, 2.04) | 2 | 1.29 (-0.39, 2.98) | -0.209 | 0.241 |
| pep_no_944 | 33 | 1 (0.9, 1.11) | 24 | 1.07 (0.83, 1.3) | 0.089 | 0.238 |
| pep_no_7672 | 5 | 1.17 (0.63, 1.7) | 15 | 1.06 (0.87, 1.25) | -0.137 | 0.237 |
| pep_no_15966 | 17 | 0.95 (0.77, 1.13) | 4 | 1.07 (0.34, 1.79) | 0.165 | 0.235 |
| pep_no_27085 | 4 | 1.35 (0.59, 2.1) | 1 | 1.02 (0, 0) | -0.396 | 0.233 |
| pep_no_26172 | 13 | 0.9 (0.45, 1.35) | 2 | 0.6 (-3.11, 4.31) | -0.592 | 0.230 |
| pep_no_7667 | 25 | 0.96 (0.52, 1.4) | 11 | 0.78 (0.5, 1.05) | -0.302 | 0.228 |
| pep_no_27375 | 5 | 1.22 (0.86, 1.59) | 2 | 1.35 (1.14, 1.57) | 0.142 | 0.227 |
| pep_no_8066 | 5 | 1.08 (0.14, 2.01) | 3 | 0.81 (-0.11, 1.73) | -0.409 | 0.225 |
| pep_no_3273 | 24 | 1.36 (1.16, 1.57) | 9 | 1.27 (0.94, 1.6) | -0.106 | 0.221 |
| pep_no_19248 | 5 | 1.02 (0.34, 1.7) | 2 | 1.39 (-11.25, 14.04) | 0.449 | 0.220 |
| pep_no_24986 | 12 | 1.17 (0.77, 1.57) | 7 | 1.03 (0.63, 1.43) | -0.189 | 0.219 |
| pep_no_9975 | 5 | 1.22 (0.62, 1.82) | 2 | 1.01 (-1.74, 3.77) | -0.267 | 0.215 |
| pep_no_6088 | 8 | 1 (0.78, 1.21) | 18 | 0.95 (0.86, 1.05) | -0.067 | 0.209 |
| pep_no_27390 | 5 | 0.98 (0.78, 1.19) | 2 | 1.06 (-0.69, 2.81) | 0.108 | 0.208 |
| pep_no_24814 | 2 | 1 (-1.77, 3.77) | 6 | 0.89 (0.62, 1.15) | -0.172 | 0.206 |
| pep_no_8347 | 3 | 1.27 (0.53, 2) | 9 | 1.48 (0.94, 2.01) | 0.226 | 0.205 |
[truncated: 12,785 more chars]
